# Sperm competition risk drives plasticity in seminal fluid composition

**DOI:** 10.1186/s12915-015-0197-2

**Published:** 2015-10-27

**Authors:** Steven A. Ramm, Dominic A. Edward, Amy J. Claydon, Dean E. Hammond, Philip Brownridge, Jane L. Hurst, Robert J. Beynon, Paula Stockley

**Affiliations:** 1 Mammalian Behaviour and Evolution Group, Institute of Integrative Biology, University of Liverpool, Leahurst Campus, Chester High Road, Neston, CH64 7TE UK; 2 Evolutionary Biology, Bielefeld University, Morgenbreede 45, 33615 Bielefeld, Germany; 3 Centre for Proteome Research, Institute of Integrative Biology, University of Liverpool, Biosciences Building, Crown Street, Liverpool, L69 7ZB UK

**Keywords:** Evolution, Phenotypic plasticity, Proteomics, Random Forest analysis, Seminal fluid, Sperm competition

## Abstract

**Background:**

Ejaculates contain a diverse mixture of sperm and seminal fluid proteins, the combination of which is crucial to male reproductive success under competitive conditions. Males should therefore tailor the production of different ejaculate components according to their social environment, with particular sensitivity to cues of sperm competition risk (i.e. how likely it is that females will mate promiscuously). Here we test this hypothesis using an established vertebrate model system, the house mouse (*Mus musculus domesticus*), combining experimental data with a quantitative proteomics analysis of seminal fluid composition. Our study tests for the first time how both sperm and seminal fluid components of the ejaculate are tailored to the social environment.

**Results:**

Our quantitative proteomics analysis reveals that the relative production of different proteins found in seminal fluid – i.e. seminal fluid proteome composition – differs significantly according to cues of sperm competition risk. Using a conservative analytical approach to identify differential expression of individual seminal fluid components, at least seven of 31 secreted seminal fluid proteins examined showed consistent differences in relative abundance under high versus low sperm competition conditions. Notably three important proteins with potential roles in sperm competition – SVS 6, SVS 5 and CEACAM 10 – were more abundant in the high competition treatment groups. Total investment in both sperm and seminal fluid production also increased with cues of heightened sperm competition risk in the social environment. By contrast, relative investment in different ejaculate components was unaffected by cues of mating opportunities.

**Conclusions:**

Our study reveals significant plasticity in different ejaculate components, with the production of both sperm and non-sperm fractions of the ejaculate strongly influenced by the social environment. Sperm competition risk is thus shown to be a key factor in male ejaculate production decisions, including driving plasticity in seminal fluid composition.

**Electronic supplementary material:**

The online version of this article (doi:10.1186/s12915-015-0197-2) contains supplementary material, which is available to authorized users.

## Background

Ejaculates are a complex mixture of sperm and seminal fluid components, many features of which are thought to have been shaped by sperm competition [1, 2]. For example, much recent research has focused on the optimal allocation of limited sperm reserves to maximise fertilisation success [3–5]. A related but separate question concerns the extent to which overall investment in ejaculate production varies with sperm competition. There is widespread support for the prediction that higher levels of sperm competition select for greater investment in sperm production, both across [6–9] and within [10–14] species, including the ability to plastically adjust sperm production to match the prevailing competitive conditions [15–19]. However, seminal fluid contains a diverse array of proteins that exert various influences on female reproductive biology, and it is likely that varying levels of sperm competition could also impact on optimal seminal fluid production and/or composition [20–24]. A further important consideration is that investment in ejaculate production might also be influenced by the number of females available for mating [25, 26]. This complicates interpretation of previous studies because sperm competition and mating rate will often be confounded [27, 28] and under certain conditions, mating rate might predominate in determining optimal male sperm production strategies [27].

Here we use an established vertebrate model system, the house mouse (*Mus musculus domesticus*), to investigate plasticity in production of different ejaculate components in relation to cues of sperm competition risk and mating rate. House mice have a relatively low overall investment in sperm production compared to many other rodents [29]. However, males typically experience a significant but variable risk of sperm competition [30, 31], which previously has been linked to phenotypic plasticity in sperm production [17, 32]. Moreover, we have shown that there is a rapid turnover of seminal fluid proteins in this species, notably more rapid than spermatogenesis [33], suggesting potential for short-term plasticity in responses of non-sperm ejaculate components. Mating opportunities are also likely to vary substantially for male house mice in natural populations, depending on the number of resident females in the territory of a particular male and the proximity of neighbouring territories [30, 34].

To determine the relative importance of sperm competition risk and anticipated mating rates on phenotypic plasticity in ejaculate production requires a controlled experiment with independent manipulation of appropriate cues used to assess these two factors. In rodents, scent represents a crucial sensory modality used in a variety of reproductive contexts [35]. For example, male rodents are sensitive to cues of sperm competition conveyed by conspecific male odours [17, 21, 36] and are capable of sophisticated discrimination of odours from female conspecifics [37, 38]. The perception of sperm competition risk and mating opportunities (hereafter ‘mating rate’) should therefore be influenced by exposure to the odours of adult male and female conspecifics, respectively, in addition to direct encounters with them. That is, a high encounter rate with multiple males and/or their odours should indicate a high risk of sperm competition, whereas a high encounter rate with multiple females and/or their odours should indicate a high potential mating rate.

To test whether male house mice vary investment in the production of different ejaculate components according to social conditions, we used a 2 × 2 factorial experimental design and independently manipulated cues of sperm competition risk and potential mating rate. Combined with detailed quantitative proteomics analysis, our experiment reveals that the relative abundance of different proteins found in seminal fluid (i.e. seminal fluid composition) and sperm production both differ according to cues of sperm competition risk. By contrast, relative investment in different ejaculate components was unaffected by cues of potential mating rate. Sperm competition risk is thus shown to be a key factor influencing male ejaculate production decisions that drives plasticity in seminal fluid composition.

## Results

The experiment simultaneously manipulated subjects’ social experience of both male and female conspecifics in a 2 × 2 factorial design to simulate both high or low sperm competition risk (HSC/LSC; subjects were provided with cues from three or one male conspecifics, respectively) and high or low anticipated mating rates (HMR/LMR; subjects were provided with cues from four or two female conspecifics, respectively – see detailed description in the Methods).

### Sperm production

**Fig. 1:**
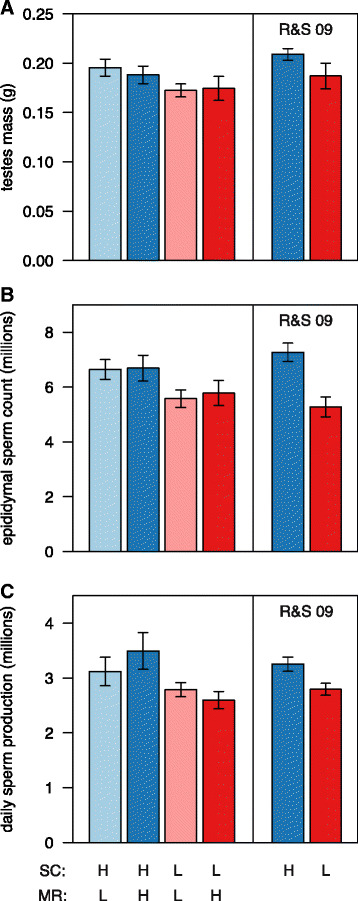
Summary of sperm production parameters under variable cues of sperm competition risk and likely mating rate. Mean (± s.e.m.) sperm production parameters: **a** testis size, **b** epididymal sperm numbers and **c** daily sperm production rate. We simultaneously manipulated social cues of sperm competition risk (SC) and potential mating rate (MR), consistently finding that only the former impacts on sperm production parameters (all *P* < 0.05, see main text for test statistics). Included for comparison in each *right-hand panel* are previously published data from Ramm and Stockley (R&S 09, [17]), who reported a complementary experiment in which mating rate cues were held constant at a low level and only sperm competition cues were varied. Note that overall these experiments point to a primary influence of sperm competition cues on plasticity in sperm production parameters, with the mean parameter estimates for the three treatment groups classified as high sperm competition (blue shaded bars) differing significantly from those for the three treatment groups classified as low sperm competition (red shaded bars) for two out of three sperm production parameters (testis mass: *t*_4_ = 2.63, *P* = 0.058; epididymal sperm numbers: *t*_4_ = 5.94, *P* = 0.004; daily sperm production: *t*_4_ = 3.70, *P* = 0.02). *HSC* high sperm competition, *LSC* low sperm competition, *HMR* high mating rate, *LMR* low mating rate

We measured three sperm production parameters: testes mass, epididymal sperm numbers, and daily sperm production rate (see Methods). There was substantial variation in all three sperm production parameters across the experimental treatment groups (Table 1). Separate analyses for the two static measures of testis size (Fig. 1a) and epididymal sperm numbers (Fig. 1b), as well as for the dynamic measure of daily sperm production (Fig. 1c), consistently revealed the same pattern. Cues of sperm competition (number of males encountered) significantly influenced sperm production parameters but there was no effect of mating rate cues (number of females encountered) on any of these parameters, and no significant interaction effects (Table 2). Males in high competition treatment groups typically had 12 % larger testes, and 20 % higher epididymal sperm numbers and daily sperm production rates, compared to males in the low competition treatment groups.

**Table 1:** Mean sperm production parameters under contrasting cues of sperm competition risk and mating opportunities

Sperm competition (SC)	Mating rate (MR)	Paired testes mass (g)	Epididymal sperm count (x10^6^)	Daily sperm production (x10^6^)	Seminal vesicles mass (g)
High	Low	0.196 ± 0.007	6.72 ± 0.32	3.04 ± 0.24	0.151 ± 0.015
High	High	0.188 ± 0.009	6.69 ± 0.47	3.50 ± 0.33	0.139 ± 0.013
Low	Low	0.169 ± 0.006	5.36 ± 0.27	2.82 ± 0.14	0.117 ± 0.012
Low	High	0.174 ± 0.012	5.78 ± 0.46	2.60 ± 0.16	0.115 ± 0.012

Mean (± s.e.m.) sperm production parameters of male house mice exposed to different social cues of sperm competition risk and potential mating rate. Sperm competition risk (SC) was manipulated by altering the number of male competitors regularly encountered (one = low; three = high) and potential mating rate (MR) was manipulated by altering the number of females regularly encountered (two = low; four = high). Testes mass is the mass of both testes (in g); epididymal sperm count is the number of sperm recovered from the caput of the right epididymis (x 10^6^) and daily sperm production rate is the number of sperm produced per testis per day, based on spermatid head counts from testicular homogenates (x 10^6^). (n = 8 in each group, except for daily sperm production rate in the low SC, high MR treatment group where n = 7)

**Table 2:** Sperm competition risk but not potential mating rate impacts on ejaculate production

Term	Paired testes mass		Epididymal sperm count		Daily sperm production		Seminal vesicles mass	
	*F*	*P*	*F*	*P*	*F*	*P*	*F*	*P*
SC	5.81	0.02	8.48	0.007	5.22	0.03	4.37	0.046
MR	0.06	0.81	0.12	0.73	0.09	0.76	0.21	0.65
SC × MR	0.48	0.49	0.32	0.58	1.96	0.17	0.06	0.81

Summary of ANOVAs testing for the effects of sperm competition risk (SC) and potential mating rate (MR) treatments on four ejaculate production parameters: paired testes mass, epididymal sperm count and daily sperm production (all sperm production measures), plus seminal vesicles mass (a measure of seminal fluid production, see Table 1 for details). In all cases, the degrees of freedom for each *F* test, d.f. = 1,28 except for daily sperm production where one subject was excluded and so d.f. = 1,27.

*ANOVA* analysis of variance

These results are supported by mixed model analyses taking into account potential non-independence of males from cages grouped within the same enclosure. The effects of competition remained either significant (epididymal sperm numbers: χ^2^ = 4.07, d.f. = 1, *P* = 0.044) or very nearly so (daily sperm production: χ^2^ = 3.57, d.f. = 1, *P* = 0.059; testis mass: χ^2^ = 3.44, d.f. = 1, *P* = 0.063). Taken together with our earlier findings [17], these data strongly support the conclusion that sperm competition cues significantly impact upon sperm production parameters (Fig. 1), and that this effect is most pronounced on epididymal sperm numbers and daily sperm production rates, and somewhat weaker on the size of the testes.

Neither sperm competition nor mating rate manipulations affected measures of ejaculate quality, such as percentage sperm motility (treatment group effect: *F*_3,28_ = 2.19, *P* = 0.1), curvilinear sperm velocity (*F*_3,28_ = 1.11, *P* = 0.4) or straight-line sperm velocity (*F*_3,28_ = 1.72, *P* = 0.2).

### Seminal fluid production

**Fig. 2:**
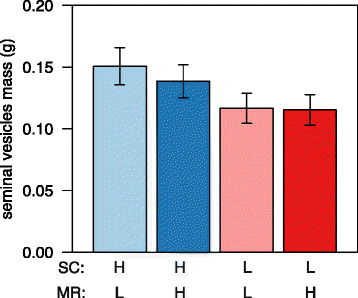
Seminal fluid production under variable cues of sperm competition risk and potential mating rate. Mean (± s.e.m.) seminal vesicles mass of male house mice exposed to different social cues of sperm competition risk (SC) and potential mating rate (MR). *HSC* high sperm competition, *LSC* low sperm competition *HMR* high mating rate, *LMR* low mating rate

In addition to effects on sperm production parameters, there was also a marginally significant effect of sperm competition risk (but not potential mating rate) on the size of the seminal vesicles, suggesting these to be on average 25 % larger in males from high competition risk treatment groups (Fig. 2), HSC, LMR: 0.151 ± 0.015 g; HSC, HMR: 0.139 ± 0.013 g; LSC, LMR: 0.117 ± 0.012 g; LSC, HMR: 0.115 ± 0.012 g; sperm competition: *F*_1,28_ = 4.37, *P* = 0.046; mating rate: *F*_1,28_ = 0.21, *P* = 0.65; sperm competition x mating rate: *F*_1,28_ = 0.06, *P* = 0.81 Table 2). However, the effect of sperm competition risk on the size of seminal vesicles was marginally non-significant in the mixed model taking potential non-independence due to enclosure effects into account (χ^2^ = 3.08, d.f. = 1, *P* = 0.07).

### Seminal fluid composition

**Fig. 3:**
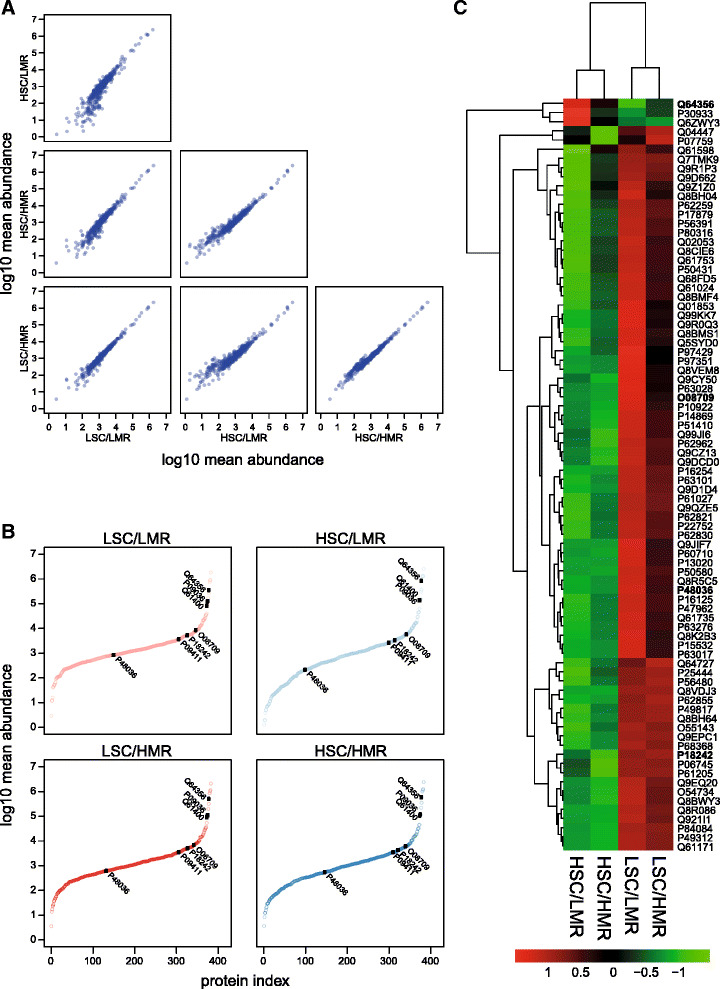
Summary of the proteomics data. **a** Consistency of measurements of normalised mean label-free abundances (log10 transformed), between treatment groups. The matrix plots show how well the seminal fluid proteomes of each treatment group correlate in pairwise comparisons. **b** Ranked mean log10 abundance curves for each of the four treatment group seminal vesicle proteomes, with those proteins found to be important for classifying samples according to the sperm competition risk treatment (from Random Forest modelling), labelled as black points with their accession number. **c** A heatmap of the mean abundance of proteins in the seminal vesicle proteomes across the four treatment groups. Un-transformed normalised abundances for those proteins present at significantly different levels between the mating groups (according to ANOVA in Progenesis QI; p < 0.05) were averaged, based on prior analyses of how well their proteomes correlate with one another between biological replicates, within each treatment group (data not shown). Mean abundances were then centred and scaled based on protein identity (row) and hierarchically clustered according to both treatment group (*column*) and protein (*row*) data. Euclidean distance measures and the “complete” linkage method for hierarchical clustering were used and the result plotted as a heatmap (using the R package ‘pheatmap’). Clear differences in the abundance of seminal vesicle proteins are apparent. Proteins found to be important for classifying samples according to the sperm competition risk treatment (from Random Forest modelling), are highlighted with bold accession numbers. Abbreviations of treatment groups are as follows: *HSC* high sperm competition, *LSC* low sperm competition, *HMR* high mating rate, *LMR* low mating rate

We next investigated the protein composition of seminal fluid, and specifically the protein secretion of the seminal vesicles – which is the major accessory reproductive gland in mice – using standard proteomics workflows. We obtained profiles that were qualitatively similar over all four treatment groups (Fig. 3a), extending to 383 proteins, and covering approximately six orders of magnitude of dynamic range (Fig. 3b). The overall profiles of each of the treatment groups were very similar, permitting comparison, at a protein level, of all treatment groups. To gain an overview of the variation in protein expression patterns revealed by these label-free proteomics data, entire protein profiles were used to direct a hierarchical clustering (Fig. 3c), restricting our analysis to those proteins for which we obtained a minimum of three high confidence peptides for quantification (see Methods). The clustering analysis indicated the strongest separation between high and low sperm competition treatments, which we tested formally using two approaches: 1) using the standard proteomics output on relative abundances for each protein obtained from Progenesis QI; and 2) a more conservative Random Forest (RF) analysis of proteome composition, focusing on a subset of (independently) known *secreted* seminal vesicle proteins, and controlling for potential differences between treatments in the proportion of secreted proteins present in each sample (for an in depth description of RF models see Methods).

#### Progenesis QI analysis of individual proteins

**Fig. 4:**
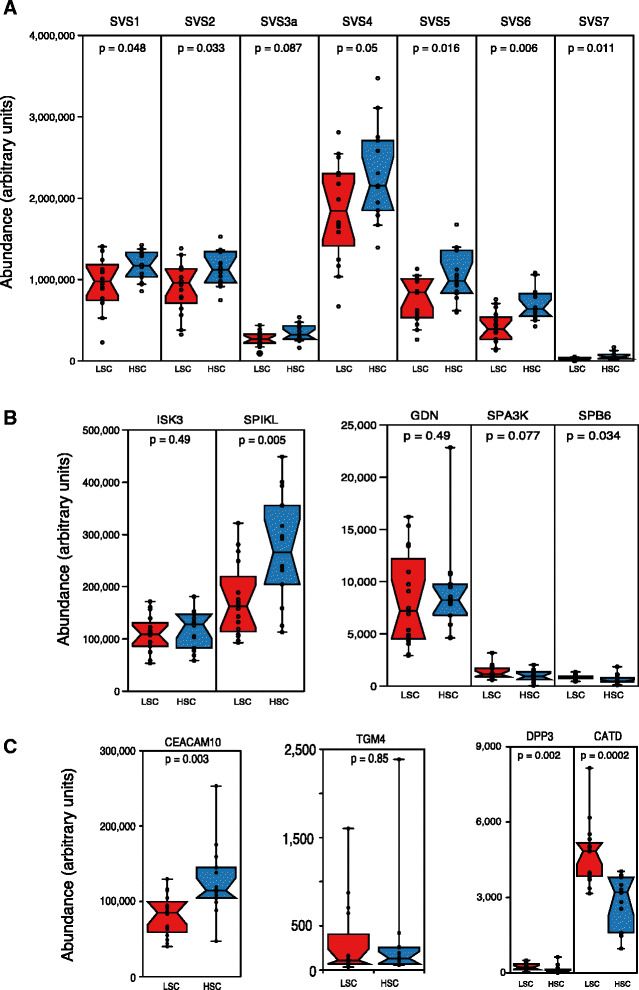
Proteomics analysis of key secreted seminal vesicle proteins. Combined data from all HSC and LSC mice were used in a label-free quantitative proteomics analysis of seminal vesicle proteins (Additional file 1). From these data, requiring at least three unique peptides for quantification and identification, the expression of the proteins was recovered and plotted on a protein-by-protein basis. Panel (**a**) ‘classical’ SVS proteins 1 to 7, panel (**b**) serine protease inhibitors and (**c**) other proteins relevant to coagulation, plug formation and survival, or sperm motility. Data are presented as notched box and whisker plots where the midline is the median, box extremes define 25^th^ and 75^th^ percentiles, and the notches are calculated as ([75^th^-25^th^ percentile value] * 1.57/sqrt(N); whiskers define extreme data points. N = 14 for HSC and 16 for LSC. *HSC* high sperm competition, *LSC* low sperm competition

We began with a broad analysis, comparing all proteins using Progenesis QI, a standard package for analysis of label-free quantitative proteomics data. For this analysis, all 383 proteins were used to normalise the samples (Additional file 1), and we focused on exploring responses of subject males to cues of sperm competition risk (number of rival males encountered). We filtered the data according to *P* < 0.05 (to detect statistically significant differential expression according to sperm competition risk treatment), and a minimum of three unique peptides used for the identification and quantification of each protein (to ensure only high quality data contributed to the analysis). Using these criteria, 49 proteins were differentially expressed, 10 of which were significantly up-regulated under high competition conditions. Of those proteins that were down-regulated, the majority were intracellular cytoskeletal or metabolic proteins, the significance of which is unclear. Although only 8 % (31/383) of proteins identified in our study are known to be secreted (based on [39]), secreted proteins make up 31 % (15/49) of proteins that are differentially expressed according to sperm competition risk. Of particular interest in the present study are the responses of secreted proteins with likely potential functions in post-copulatory sexual selection. These include a major family of seminal vesicle proteins that are known to be secreted by this accessory reproductive gland (SVS 1–7 Fig. 4a), six of which – SVS 1 (Q6WIZ7), SVS 2 (Q62216), SVS 4 (P18419), SVS 5 (P30933), SVS 6 (Q64356), and SVS 7 (Q09098) – were at or below the individual significance threshold for differential expression of *P* = 0.05 in this initial Progenesis QI analysis, all being more abundant under high competition (with a trend for SVS 3 (Q8VI13) at *P* = 0.08 to also vary in the same direction). Serine protease inhibitors (Fig. 4b) are also of interest because recently mated females increase intravaginal endopeptidase production thought to aid plug dissolution, and there is good evidence for a protease: antiprotease system in sexual conflict [40, 41]. Of five serine protease inhibitors identified in our Progenesis QI analyses, two were unchanged: Serpin Kazal-type 3 (ISK3, P09036) and Glia-derived nexin (GDN, Q07235). However, one abundant protease inhibitor Serpin kazal-like protein (SPIKL, Q8CEK3) was significantly up-regulated (1.6 fold) under high competition conditions. Two other low abundance inhibitors, Serpin A3K (SPA3K, P07759) and Serpin B6 (SPB6, Q60854) were slightly down regulated in high competition groups (1.2 and 1.4 fold, respectively) (Fig. 3b). The label-free quantification confirms a broad dynamic range of expression of the individual inhibitors (at least 10-fold), which would be consistent with their targeting of different proteases. We also explored changes in the coagulum cross-linking transglutaminase TGM4 (Q8BZH1) and CEACAM 10 (Q61400), a glycoprotein commonly secreted by seminal vesicles in rodents [42] and implicated in sperm motility (see Discussion). Of these, only CEACAM 10 was significantly up-regulated under high sperm competition risk. Two peptidases – DPP3 (Q99KK7) and cathepsin D (CATD, P18242) – both exhibit lowered expression under high competition (Fig. 4c).

#### Random Forest analysis of proteome composition

Next, we employed a more stringent approach to analysing the seminal vesicle proteome, performing a single analysis of proteome composition and focusing solely on that proportion of the proteome that is secreted and that could thus have functions relevant to sperm competition outcomes within the female reproductive tract. To do so, we implemented a Random Forest (RF) classification model for proteins secreted by the seminal vesicles (see Methods). In contrast to the standard Progenesis QI analysis reported above (see Additional file 1), for this analysis we used only known ejaculated proteins (from [39]) to normalise the samples, again using a minimum of three unique peptides per protein for identification and quantification (see Additional file 2). Normalising to ejaculated proteins is a more conservative approach that controls for any potential differences between treatments in the proportion of secreted proteins present in each sample (see Methods). This might occur, for example, if larger seminal vesicles associated with high competition were to contain a higher ratio of lumen to tissue, and hence a higher proportion of ejaculated to non-ejaculated proteins. We used this approach for analysis of all four treatment groups, to investigate effects of both sperm competition risk (number of males encountered) and potential mating rate (number of females encountered).

**Fig. 5:**
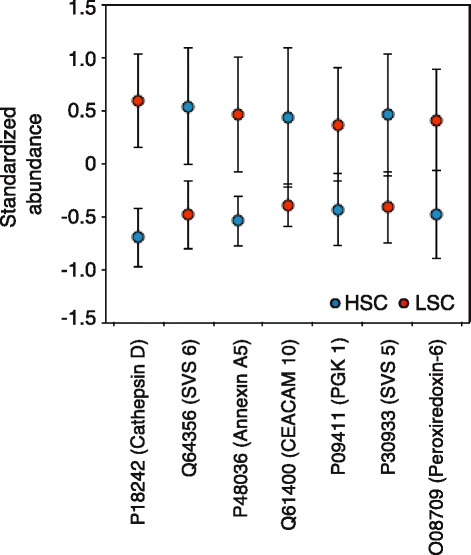
Random forest analysis of seminal fluid composition under variable sperm competition risk. The figure plots the seven secreted seminal vesicle proteins important for classifying samples according to the sperm competition risk treatment, based on variable importance scores. Standardised abundance scores of each protein are shown (blue symbols: high sperm competition (HSC); red symbols: low sperm competition (LSC); mean ± 95 % confidence intervals). Proteins are ordered according to their variable importance score as per Table 3 (see Table 3 for UniProt IDs). Analysis is based on data requiring at least three unique peptides for quantification and identification, with normalisation to ejaculated proteins (see Methods)

**Fig. 6:**
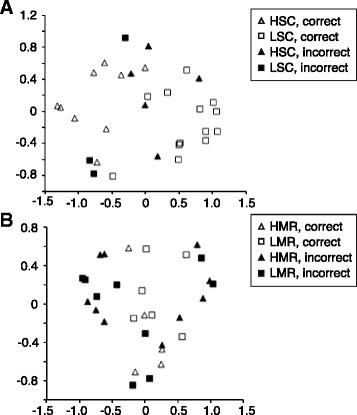
Multidimensional scaling (MDS) plots illustrating seminal fluid compositional differences under variable sperm competition risk and potential mating rate. MDS plots showing the distance between samples in two dimensions, MDS1 (*x*) and MDS2 (*y*). Plots are produced from the proximity matrix generated in Random Forest (RF) models that were used to classify samples according to (**a**) the number of males and (**b**) number of females to which each male was exposed. Square symbols are individuals that were exposed to the “low” treatment, i.e. either one male (in **a**) or two females (in **b**). Triangular symbols are individuals exposed to the “high” treatment in each analysis, i.e. either three males (in **a**) or four females (in **b**). Filled symbols indicate samples that were incorrectly predicted by the RF model, illustrating the much better ability of the RF analysis to classify samples correctly based on cues of likely sperm competition (**a**; 22/30 classified correctly) rather than cues of potential mating opportunities (**b**; 11/30 classified correctly; see main text for test statistics). HSC: high sperm competition; LSC: low sperm competition; HMR: high mating rate; LMR: low mating rate.

The results of the RF model indicate that secreted seminal vesicle protein composition indeed differs systematically according to sperm competition risk treatment. The RF model had a classification error rate of 26.67 % (22 correct: 8 incorrect), meaning that it was unlikely to have been achieved by chance (21 of 1,000 bootstrap replicates achieved this error rate or lower; *P* = 0.021). This level of accuracy in the classification of samples was achieved because of differences in the abundances of individual proteins between treatments. Of 31 proteins analysed, seven had an important influence on the classification accuracy of the RF model, including four also identified as significantly different between treatment groups in our initial Progenesis QI analysis (SVS 5, SVS 6, CEACAM 10, and cathepsin D). Notably, three important proteins with potential roles in sperm competition – SVS 6, SVS 5, and CEACAM 10 – were more abundant in the high competition treatment groups. Two other proteins – cathepsin D and annexin A5 (P48036) – were down-regulated under conditions of high sperm competition, and two – peroxiredoxin-6 (O08709) and phosphoglycerate kinase 1 (P09411) – are likely to be cytosolic proteins, the precise role of which remains uncertain. Differences in the abundance of all important proteins are depicted in Fig. 5, and the results of RF models are summarised in Table 3, alongside a series of independent t-tests for each individual protein as an alternative method of analysis more akin to the initial Progenesis QI protein-by-protein analysis, but here controlling for both potential differences between treatments in the proportion of secreted proteins and statistically for multiple hypothesis testing. The results of the RF model are largely consistent with those based on q-values obtained from these multiple t-tests, that is, p-values corrected for multiple comparisons (but see Methods for an explanation of why the RF model approach is preferable). The separation of samples using the RF approach with respect to sperm competition treatment is summarised as a multidimensional scaling plot in Fig. 6a.

**Table 3:** Random forest analysis of plasticity in seminal fluid composition

Protein	Name	Importance	Importance	T-Test	
			95 % CI		
				*p*	q
P18242	Cathepsin D	46.398	45.14 - 47.63	0.000	0.002
Q64356	SVS 6	30.231	28.99 - 31.76	0.003	0.038
P48036	Annexin A5	28.445	26.60 - 29.61	0.004	0.038
Q61400	CEACAM 10	12.745	10.64 - 14.67	0.019	0.088
P09411	Phosphoglycerate kinase	12.138	10.16 - 13.86	0.025	0.096
P30933	SVS 5	10.058	7.95 - 12.80	0.014	0.084
O08709	Peroxiredoxin-6	9.683	7.75 - 12.02	0.011	0.082
Q09098	PATE 4 / SVS 7	6.589	4.52 - 8.51	0.033	0.114
Q8CEK3	Spikl	6.043	3.56 - 8.09	0.020	0.088
P07759	Serine protease inhibitor A3K	5.887	3.21 - 7.92	0.062	0.138
P21460	Cystatin-C	4.772	2.65 - 7.28	0.061	0.138
P45376	Aldose reductase	4.673	2.19 - 6.88	0.717	0.793
P12032	Metalloproteinase inhibitor 1	2.288	−0.17 - 4.98	0.160	0.261
Q8BZH1	Transglutaminase 4	2.171	−0.16 - 4.01	0.992	0.992
P01887	Beta-2-microglobulin	2.140	−0.15 - 4.67	0.040	0.123
Q6WIZ7	SVS 1	1.240	−1.16 - 3.92	0.052	0.135
Q3SXH3	SVA	0.291	−1.48 - 2.25	0.111	0.214
P35700	Peroxiredoxin-1	0.199	−2.11 - 2.15	0.649	0.775
P18419	SVS 4	−0.169	−2.44 - 2.50	0.050	0.135
Q62216	SVS 2	−0.467	−2.70 - 2.07	0.070	0.144
Q9QY48	Deoxyribonuclease-2-beta	−0.644	−2.24 - 0.99	0.125	0.228
Q8VI13	SVS 3	−1.856	−3.77 - 0.16	0.135	0.232
Q01768	Nucleoside diphosphate kinase B	−1.949	−3.71 - 0.30	0.314	0.482
P81117	Nucleobindin-2	−2.434	−4.80 - 0.14	0.419	0.564
P20029	78 kDa glucose-regulated protein	−2.990	−5.36 - -1.08	0.446	0.576
P14152	Malate dehydrogenase	−3.549	−5.83 - -1.85	0.822	0.879
Q8BND	Sulfhydryl oxidase	−4.700	−6.64 - -2.85	0.327	0.482
P08228	Superoxide dismutase	−5.600	−7.48 - -3.23	0.370	0.522
Q07235	Glia-derived nexin	−5.685	−7.88 - -3.58	0.650	0.775
P07724	Serum albumin	−5.803	−8.10 - -3.49	0.913	0.943
P09036	Spink 3	−6.798	−8.7 - -5.00	0.703	0.793

The table lists variable importance scores for each secreted seminal vesicle protein from the RF model used to classify samples according to sperm competition risk experienced by subject males. Proteins that differ between treatments will make a greater contribution to the accurate classification of samples in RF models and thus have higher variable importance scores. Proteins are arranged in descending order of variable importance. The first seven proteins listed are defined as being important for classifying samples according to the sperm competition risk treatment. These are proteins that have a score greater than 6.798, which is the absolute value of the lowest variable importance score of all proteins. The results of multiple t-tests are also provided for comparison to the results of RF models. P-values resulting from t-tests were corrected for multiple comparisons by the FDR using the Benjamini-Hochberg method to yield q-values.

Abbreviations: *SVS* seminal vesicle secretory protein, *CEACAM* carcinoembryonic antigen-related cell adhesion molecule, *Spikl* serine protease inhibitor kazal-like, *PATE4* prostate and testes expressed protein, *Spink* serine protease inhibitor kazal-type, *RF* Random Forest, *FDR* false discovery rate)

To determine whether males from the same enclosure tended to be more similar, and thus whether our results may be affected by a common environment effect, we also generated a RF classification model that analysed differences in protein abundance that might have arisen between enclosures. The model performed poorly at classifying males to the correct enclosure, having a classification error rate of 100 %. Additionally, when analysing the differences between males described in the proximity matrix of this model, males were not more similar to their enclosure partners than individuals from other enclosures.

Finally, we investigated the protein composition of seminal fluid using an RF classification model that analysed differences in protein abundances between samples according to the potential mating rate treatment (number of females encountered). This RF model had a classification error rate of 63.3 % (11 correct: 19 incorrect) that could easily have been achieved by chance (968 of 1,000 bootstrap replicates achieved this error rate or lower; *P* = 0.97). This shows that cues of potential mating rate did not influence the relative abundance of seminal vesicle proteins, as can be visually inferred from the absence of separation of samples with respect to mating rate treatment in the multidimensional scaling plot summarising the RF analysis (Fig. 6b).

## Discussion

Our findings reveal that social cues of sperm competition risk are a critical factor in determining male investment in ejaculate traits. Whilst previous studies have largely focused on the impact of social conditions on ejaculate allocation [4, 5, 20, 43, 44] (but see, e.g. [15–17]), we demonstrate here the importance of the social environment on male ejaculate production strategies. We show that: (1) sperm competition risk cues strongly influence production of both seminal fluid proteins and sperm; but (2) cues of potential mating opportunities do not appear to influence ejaculate production in this species.

### Sperm competition risk drives plasticity in seminal fluid composition and investment

Using a proteomics approach to identify and quantify seminal vesicle proteins, we found strong evidence that cues of sperm competition risk influence seminal fluid proteome composition. The response of SVS proteins to cues of sperm competition risk are of particular interest. Collectively, the SVS proteins make up the vast majority of the seminal vesicle secretion; they are likely to be important to male competitive fertilisation success due to established roles in copulatory plug formation, sperm transport and sperm motility (reviewed in [42, 45]), and many exhibit interspecific evidence of rapid evolution [46–48]. Although caution is required in interpreting results of our Progenesis QI analyses, overall these suggest a consistent trend of up-regulated SVS proteins under high competition conditions. By contrast, the 24 other ejaculated proteins that we investigated showed no such directional bias (15 were more abundant in the high competition treatment, and 9 in the low competition treatment). This pattern is supported by results of our Random Forest analyses showing significant differences in secreted seminal fluid proteome composition, with SVS 5 and SVS 6 among the seven proteins identified as important for classifying samples according to sperm competition treatment group. Although a role in sperm competition for SVS 5 and SVS 6 appears likely, elucidating their precise function requires further investigation. Roles in relation to copulatory plugs could be one possibility. However, neither SVS 5 nor SVS 6 contain transglutaminase substrate domains, and so are unlikely to be actively involved in copulatory plug formation [49]. Interestingly, these proteins have also been suggested to be serine protease inhibitors [47], based on their proximity in the genome to other WFDC proteins, some of which have this activity. However, SVS 5 and SVS 6 do not possess the classic four disulphide bond potential, and homology modelling to predict a three dimensional protein structure [50] leads to low confidence models that do not map to known serine protease inhibitors. There has been no direct demonstration of anti-protease activity of these proteins, and although the role of SVS 5 in copulatory plug survival has recently been questioned [41], this does not preclude other functions in sperm competition unrelated to plug formation or dissolution. Also, in relation to copulatory plug formation, we note that the coagulum transglutaminase TGM4 – which catalyses the cross-linking of SVS proteins – was not itself significantly up-regulated under high sperm competition risk according to our initial Progenesis QI analysis.

Although we found limited evidence for an influence of the social environment on proteins related to copulatory plug formation, there is some evidence for an up-regulation of proteins linked to sperm motility under conditions of high sperm competition risk. For example, according to our initial Progenesis QI analysis, SVS 7 (caltrin, PATE4), a protein that enhances sperm motility [51], was significantly up-regulated in our high competition treatment groups. Further support for this idea comes from more robust evidence of increased CEACAM 10 production under high competition conditions. CEACAM 10 is a glycoprotein that enhances sperm motility [52], and its increased production under high sperm competition risk is also confirmed by our Random Forest analysis.

Our Random Forest analysis indicates that two other proteins – cathepsin D, an aspartic protease, and annexin A5 (function unknown) – were down-regulated under conditions of high sperm competition (in addition DPP3 was down-regulated under high competition according to our initial Progenesis QI analysis). These responses are less easily explained, although it is possible that reduction of the potential proteolytic environment of the seminal vesicle may exert other effects influencing reproductive success under sperm competition [53].

In summary, these findings based on quantitative protein analysis in a model vertebrate provide direct and broad-scale evidence of plasticity in seminal fluid protein production linked to sperm competition risk. We are aware of only one previous experimental study that attempted to measure plasticity in seminal fluid production in response to sperm competition cues, and this measured gene rather than protein expression levels. Fedorka *et al.* [22] manipulated the social environment of male fruit flies (*Drosophila melanogaster*), and found evidence for plastic expression of two out of three seminal fluid protein-coding genes investigated – Acp26Aa and Acp62f (but not Acp70A) – both of which were down-regulated 72 h after eclosion under high competition conditions.

A further novel finding of the present study is that male house mice appear to adjust investment in the overall size of the seminal vesicles according to cues of average sperm competition risk. The marginal significance of this response means the result should be interpreted with caution, especially because no similar response was found in our previous study [17]. Nevertheless, it seems likely that altered male investment in the size of the seminal vesicles, and thus presumably in the total amount of seminal fluid production, could be adaptive under varying sperm competition risk. Indeed, recent evidence points to an influence of seminal vesicle mass on competitive male reproductive success in semi-natural house mouse populations [54], as would be expected if variation in seminal fluid production is relevant to sperm competition outcomes. Socially-mediated plasticity in seminal vesicle size in a second rodent species, the bank vole *Myodes glareolus* [21], the correlated evolution of sperm and seminal fluid investment across rodent species [8], and the rapid evolutionary dynamics of specific seminal fluid components [42, 46–48], all serve to further emphasize the likely importance of non-sperm ejaculate components in mammalian sperm competition. Future experimental work should now focus on the fitness consequences of variation in total and individual seminal fluid protein production (cf. [20]).

### Sperm competition risk drives sperm production plasticity

Male house mice in this study adjusted overall investment in sperm production according to cues of average sperm competition risk experienced during sexual development. Specifically, those males that were repeatedly exposed to social cues from three rival males had higher daily sperm production rates and higher epididymal sperm counts compared to those that had encountered social cues from just one rival male. Importantly, these changes occurred irrespective of whether subject males were regularly also exposed to social cues from two or four potential mates, suggesting that cues of likely mating rate at this level do not affect sperm production plasticity in this species (see below).

Our findings are generally consistent with results from two previous studies in which social experience of male house mice with male (but not female) conspecifics was manipulated to vary cues of average sperm competition level, and where sperm production was also higher following regular encounters with more male conspecifics [17, 32]. In the present study, and contrary to previous findings [17, 32], we additionally report a moderate but significant influence of male social experience on the testis mass of male house mice, consistent with widely held assumptions linking testis size to sperm production rates (see [55] and references therein). Nonetheless, we also found a disproportionate increase in the other sperm production parameters (epididymal sperm numbers and daily sperm production based on spermatid head counts from testicular homogenates) relative to testis size, a pattern that is highly consistent across studies [17]. This implies that an increased testis size alone cannot explain all of the sperm production plasticity response seen in male house mice. Similar evidence for invertebrates suggests that this may be a common feature of phenotypic plasticity in sperm production (e.g. [56]), although the precise adjustments within the testis that enable males to plastically increase investment in sperm production under heightened sperm competition remain to be elucidated (reviewed in [57]; see also [58–62] for evolutionary responses to sperm competition besides gross testis size).

In addition to sperm production parameters, we also investigated a measure of sperm quality. Despite recent evidence that mouse sperm motility parameters respond to different post-copulatory sexual selection regimes under experimental evolution [60], we found no evidence of plasticity in sperm motility parameters in response to cues of either sperm competition risk or likely mating rate, at least under *in vitro* conditions, consistent with earlier findings in this species [32].

### Female-mediated cues do not impact on ejaculate production

Although male house mice in our study adjusted overall investment in ejaculate production according to their encounters with male conspecifics, we found no evidence that ejaculate investment increased as predicted in response to encounters with female conspecifics. Here we predicted a potential response to likely mating rates: males that regularly encounter more females might perceive more potential mating opportunities and increase their sperm and seminal fluid production accordingly. Since no such effect was observed, our findings suggest that variation in mating rate may not be as important as sperm competition in determining optimal ejaculate investment decisions for male house mice, at least at the relatively modest but naturalistic levels of variation in potential mating opportunities presented within our experimental treatment groups. In general, male house mice appear relatively constrained in the number of ejaculates they are able to produce within a given 24 hour period [63–65], such that some degree of sperm limitation may be expected to occur where two or more females within their territory are simultaneously receptive [66]. Cues encountered from four females might, therefore, reasonably be regarded as offering a potentially high mating rate for male house mice, although we cannot rule out the possibility that more extreme variation in female encounter rates might reveal a significant response in sperm production traits. For example, an experimental evolution study in *Drosophila melanogaster* revealed that mating rate can significantly impact on sperm production parameters under relatively extreme female-biased sex ratios [27] (cf. [67]). Similar effects could also explain why some other experimental evolution studies have not observed an evolutionary response in testis size in male-biased populations, where sperm competition is elevated but mating rate is simultaneously reduced [68]. In contrast to these previous studies however, the cues of likely sperm competition and mating rate that we manipulated in the present study were indirect; hence, it is possible that repeated emptying and re-filling of sperm reserves under a realised elevation in mating rate might affect sperm production parameters differently (see also [16, 55]).

## Conclusions

In conclusion, our study reveals significant plasticity in different ejaculate components, with both the relative abundances of different seminal fluid proteins and sperm production strongly influenced by the social environment. Moreover, we show that experience with male conspecifics – and thus the perceived level of sperm competition – predominates over experience with female conspecifics in determining variable investment in ejaculate components. This confirms sperm competition risk as a key factor in male ejaculate production decisions. We also show here for the first time that this risk drives plasticity in seminal fluid composition as well as sperm numbers. Our results, therefore, emphasise the importance of considering the whole ejaculate when seeking to understand male responses to sperm competition [2, 69].

## Methods

### Experimental design

To distinguish the potential effects of sperm competition risk and potential mating rate cues on sperm production plasticity, we exposed recently-weaned experimental subjects to long-term treatments that manipulated their social experience with both male conspecifics (as a cue of sperm competition risk) and female conspecifics (as a cue of anticipated mating rate). The manipulations were achieved by closely controlling the social experience of subjects, including controlled physical encounters with both male and female conspecifics and regular exposure to conspecific odours.

### Subjects

Subject males (n = 32) were from a colony of wild house mice that had been outbred for six or fewer generations in captivity and originally derived from local populations in Cheshire. Each male was individually housed in a 48 cm × 11.5 cm × 12 cm cage for the duration of the experiment (M3, North Kent Plastic Cages Ltd., UK), with Corn Cob Absorb 10/14 substrate and paper-wool nest material, with *ad libitum* access to food (LabDiet 5002) and water. Subjects were maintained under controlled environmental conditions: temperature 20–21 °C, relative humidity 45–65 % and a reversed 12:12 h light cycle (lights off at 08.00). Females used for the experiments (n = 36) were unrelated, sexually mature individuals from the same colony, housed in (unrelated) pairs in M3 cages distributed on a rack in the same room as the experimental males. All social experience manipulations were performed during the dark period.

### Cues of sperm competition risk and potential mating rate

The experiment was designed to simultaneously manipulate cues of sperm competition risk and potential mating rate. This was achieved by a 2 × 2 factorial design incorporating contrasting levels of both sperm competition (high sperm competition (HSC) vs. low sperm competition (LSC)) and mating rate (high mating rate (HMR) vs. low mating rate (LMR)), with n = 8 males per block (n = 32 in total) and social experience manipulated over a period of ten weeks. Recently weaned subject male mice (age at start of experiment: mean 32.9 days, range 31-33 days) were randomly allocated to treatment groups, whilst ensuring brothers were not grouped together and with minor modifications to ensure body masses were balanced at the start of the experiment (mean ± SEM; HSC, LMR: 15.76 ± 0.74 g HSC, HMR: 16.31 ± 0.78 g; LSC, LMR: 15.88 ± 0.56 g; LSC, HMR: 16.18 ± 0.42 g). Males did not differ in body mass between treatment groups at the end of the experiment (mean ± SEM; HSC, LMR: 20.14 ± 0.83 g; HSC, HMR: 20.11 ± 0.89 g; LSC, LMR: 19.83 ± 0.68 g; LSC, HMR: 18.99 ± 0.78 g; *F*_3,28_ = 0.44, *P* = 0.7).

Each experimental male was placed (within its home cage) inside a high-sided enclosure (each 1.25 m × 0.6 m × 0.8 m high) with either one (LSC treatment groups) or three (HSC treatment groups) other males present in the same enclosure (also within their home cages). All males had the opportunity to interact with the other males in their enclosure during weeks 1, 3 and 5 of the experiment. To do this we released each male in turn from their home cage for a period of 30 min each. This ensured that males were always separated by cage bars to prevent escalation of any aggressive interactions. In HSC treatment groups, this contact experience was supplemented by the transfer of odour cues (approximately 25 g soiled bedding) among experimental cages three times a week for all ten weeks of the experiment. This followed a weekly schedule whereby each experimental male received one odour cue from each of the three other experimental males housed in the same enclosure. A similar schedule was followed for the LSC treatment groups except that odour cues from the competitor male were transferred once a week. On the other two transfer days each male received a sham odour transfer from their own cage to control for cage handling between treatments.

Cues of likely mating rate were manipulated by transferring female odour cues (approximately 25 g soiled bedding) to subject male cages. Odour cues came from either one cage containing a pair of females (LMR) or from two cages each containing a pair of females (HMR). Transfers were carried out on two days each week, each time with odour cues from two females transferred in the LMR treatment, and from four females in the HMR treatment. Female odour cues were transferred on different days to the transfer of male odour cues, for the full ten weeks of the experiment. Each pair of females contributed odour cues consistently to only one male enclosure (containing either two or four males, depending on the SC treatment). We also allowed males to interact with females by placing the female cages within the high-sided enclosures and releasing each male in turn from its home cage, during which time the other male cages were removed. This was done for a period of 30 min per male in weeks 2, 4 and 6 of the experiment. Male and female cages were cleaned (including replacement of all soiled substrate and nest material with fresh substrate and nest material) in weeks 2, 4, 6, 8, and 10. Ejaculate production parameters were assessed by removing subjects from the experiment in a randomised order at the end of week 11 and during week 12.

### Seminal fluid investment

The seminal vesicles are the largest of the accessory reproductive glands in rodents and the major site of seminal fluid production [8]. At the end of the experiment, we therefore recorded the mass of the left and right seminal vesicles, as a measure of investment in seminal fluid production. Samples were then frozen at −20 °C for later analysis.

### Proteomics and label-free quantification of seminal fluid

Whole seminal vesicles were thawed then homogenised on ice in 20x volume 50 mM ammonium bicarbonate (based on fresh weights). Each homogenate was assayed for protein concentration; 50 μg of protein per sample was proteolysed with trypsin, after treatment with dithiothreitol and iodoacetamide to reduce and alkylate cysteine residues. The final digestion volume was 200 μL. Digests were further diluted to ensure an approximate concentration of 0.8 μg/μL. Samples were analysed for 30 of the 32 experimental subjects (samples for two subjects suffered degradation as a result of being defrosted prematurely and refrozen).

To quantify the abundance of specific seminal fluid proteins, mass spectrometry was performed as described previously [33]. Briefly, the samples, as prepared above, were analysed as tryptic peptides, with 1 μL of the final dilution resolved by reversed-phase (C18) ultra performance liquid chromatography (Waters nanoAcquity) over a 30 min linear organic gradient of 3–40 % buffer B (0.1 % formic acid in acetonitrile), prior to tandem mass spectrometry using a LTQ-Orbitrap Velos (Thermo Scientific, Waltham, MA, USA). High resolution, accurate mass data were acquired in a data-dependent manner, with the top 20 most intense peptides in each MS scan selected for fragmentation.

The raw data were processed using Progenesis QI (v2, Nonlinear Dynamics) to determine protein abundances. The data from all raw files were automatically aligned according to retention time to produce an aggregate spectrum, from which charge states +1 and > +4 were excluded. This aggregated spectrum contains tandem MS fragmentation data from all aligned runs enabling maximal protein identifications across all samples. Data were then separated into four experimental treatment groups, with eight males acting as biological replicates for both LSC groups and seven males for the HSC conditions. The aggregate peak list file (.mgf) was created using standard default settings and specified to contain only data relating to peptides ranked 1–4. This was then searched against a protein database containing reviewed UniProt entries for *Mus musculus* plus a small number of unreviewed entries relating to proteins identified elsewhere [42], using Mascot (v 2.5.1). A 10 ppm peptide tolerance and 0.5 Da MS/MS tolerance were set, with modifications of fixed cysteinyl carbamidomethylation and variable oxidation of methionine. Trypsin was the specified enzyme, allowing for one miscleavage. The .xml file generated by Mascot was imported back into Progenesis QI for feature assignment, normalisation and relative quantification. Data were normalised 1) to all proteins identified in the seminal vesicle samples (see Additional file 1 – data are split by treatment groups according to high or low sperm competition risk), and 2) to ejaculated protein(s) in the list identified by Dean *et al.* [39] (see Additional file 2 – data are split by treatment groups according to high or low sperm competition risk, and high or low potential mating rate). Normalisation to all proteins is the default approach; however, in the latter case we normalised to ejaculated proteins because there was a trend for males in our high competition treatment groups to develop larger seminal vesicles than those in the low competition groups. This is important to take into account because even though we analysed an equivalent total amount of protein for each male, larger seminal vesicles might contain a different ratio of lumen to tissue, and hence a different ratio of ejaculated to non-ejaculated proteins. Protein quantification was based on averaging the individual abundances of the top three most abundant peptides for each protein and then comparing them relatively across runs. Summaries of the quantitative proteomics data and Progenesis QI analyses are provided in Fig. 1 and Additional files 1 and 2, respectively.

### Statistical analyses of seminal fluid composition

A total of 383 proteins were identified in the seminal vesicles from 30 experimental subjects, although our analysis considers only 147 of these that were quantified using at least three peptides (see Additional files 1 and 2). The seminal vesicle samples analysed contain a complex mixture of proteins at widely varying concentrations. In addition to proteins found in the lumen of the seminal vesicle, these 147 proteins also include seminal vesicle tissue proteins. So that only ejaculated proteins were considered, we compared our 147 proteins against the list of 69 male-derived proteins identified by Dean *et al.* [39] that are contained in the male ejaculate, and limited further analysis to the 31 proteins common to both datasets. Note that we do not expect to identify all 69 ejaculated proteins identified by Dean *et al.* [39] as these will also be sourced from other reproductive tissues, such as the prostate. By limiting our analysis to 31 proteins known to be transferred to the female at ejaculation we therefore focus here on proteins that could potentially influence male success in sperm competition.

Abundances were analysed using Random Forest (RF) classification models [70]. RF is a classification algorithm highly suited to the analysis of high-dimensional proteomics and other -omics datasets [71]. In brief, RF creates an ensemble of decision trees (a forest) in which each tree is trained to classify a set of samples to different classes (e.g. experimental treatment groups) using a set of variables (e.g. protein abundances). If the abundance of a protein, or proteins, differs between experimental treatment groups, the RF model will be more accurate at classifying samples to the correct class. Each tree in the forest is trained using a different random subset of samples and proteins from the entire dataset. Remaining samples not used in the training stage are termed ‘out of bag’ (OOB) samples. After training, each tree is used to predict the experimental treatment of each OOB sample and these are subsequently averaged across all trees to give an overall classification for each sample. The predicted classifications are then compared to the actual experimental treatment of each sample to determine the overall accuracy of the RF model. If the RF model can accurately predict the experimental treatment of each sample, this indicates differences in the abundance of proteins between treatments.

The results of the RF model can be used to identify which proteins differ most between classes or experimental treatments. This is done by extracting variable importance scores for each protein. These importance scores describe the contribution of each protein to the accurate classification of samples across all the trees in the forest. Proteins that differ most between treatments will make a greater contribution to the accurate classification of samples and consequently have higher variable importance scores.

RF classification models were implemented using the randomForest package [71] in R v2.15.1 (R Foundation for Statistical Computing, Vienna, Austria). Each RF model contained 10,000 trees. The number of variables sampled in each tree was determined using the tuneRF function in randomForest. Outliers were defined as samples that consistently achieved an outlier score greater than 10 [71].

Two RF models were generated to test for differences based on treatment group. The first model classified samples according to the number of males encountered by subjects in each group whilst the second model classified samples according to the number of females encountered by subjects in each group. As RF is a stochastic modelling approach, that is, each tree is based on a random subset of samples and variables, each RF model was generated 1,000 times. OOB classification errors are reported as the median error rate across all 1,000 replicates of each model. The significance of OOB classification errors was determined by performing a further 1,000 bootstrap replicates of each model with random permutations of the classification variable.

Variable importance (VI) scores are reported as the mean decrease in classification accuracy attributable to each protein. VI scores were averaged and 95 % confidence intervals estimated from the 1,000 replicates of each RF model. Variables are defined as being important for differentiating samples only when the VI score is greater than the absolute value of the lowest VI score. VI scores were only reported for a model if the OOB classification error rate of the model was significantly greater than expected by chance.

RF models are highly suited to the analysis of proteomics datasets [70]. Advantages of this approach include the ability to deal with a larger number of variables relative to sample size, the test is non-parametric, models are not sensitive to outliers, overfitting is not a problem, and variable importances and sample proximities are easily generated. An alternative method of analysis to identify significant differences in individual protein abundances between treatments would be to perform a series of statistical tests, for example t-tests, with subsequent correction for multiple comparisons. However, there is considerable debate over the correct method to adopt to control for multiple comparisons. Broadly speaking, methods control either for the familywise error rate (e.g. Bonferroni, Holm-Bonferroni, Hochberg-Bonferonni) or false discovery rate (FDR; e.g. Benjamini-Hochberg, Benjamini-Hochberg-Yekutieli). The chance of type I error, that is, falsely rejecting the null hypothesis, will depend on the method adopted. To validate our analysis and provide a comparison of different methods available to analyse our data, we have also conducted multiple t-tests to analyse differences according to the sperm competition treatment (number of males). P-values resulting from t-tests were corrected for multiple comparisons by the FDR using the Benjamini-Hochberg method to yield q-values.

### Sperm production

At the end of the experiment we determined three sperm production parameters: testes mass (immediately, combined mass of both testes), epididymal sperm numbers (immediately, based on the caput of the right epididymis), and daily sperm production rate (using frozen samples of the right testis), as previously described [17]. The latter measure is based on spermatid head counts from testicular homogenates which, because the timing of spermatogenesis in mice is known, can be converted into a dynamic measure of sperm output per testis per day (see [17, 72]). One male was excluded from the analysis of daily sperm production rate due to problems processing frozen material. We also recorded male body mass at the beginning and end of the experiment. All measurements were taken blind to the treatment group of the experimental subject being measured.

### Sperm motility

We estimated sperm motility parameters for subject males based on sperm recovered from the left epididymis, as described by Lemaître *et al*. [21]. Briefly, at the end of the experiment sperm were collected by making ten incisions in the cauda epididymis with a scalpel blade into 200 μL BWW medium [73]. This was incubated for 15 min at 37 °C and a 20 μl sample then transferred to a glass slide and a cover slip applied. For each sample we obtained two video recordings of sperm, each of 2 s duration and captured at 75 frames/s using a Leica DM1000 microscope with heated stage set to 37 °C coupled to a Point Grey Flea2 (FL2-03S2M-C) 1394b camera and FlyCapture® software (Point Grey Research Inc., Richmond, BC, Canada). Videos were analysed in ImageJ [74] with the CASA plugin [75] using parameters optimised for mouse sperm.

### Statistical analyses of sperm data

Effects of the two experimental factors (sperm competition risk, potential mating rate) and their interaction were assessed using general linear models, with data log-transformed as appropriate. We also conducted mixed model analyses to control for potential non-independence of subjects whose home cages were grouped within the same enclosure (i.e. with enclosure ID fitted as a random effect). The significance of individual fixed terms was assessed using likelihood ratio tests comparing models with and without the term of interest. Analyses were performed in JMP v10 and using the lme4 package in R v2.15.1 (R Foundation for Statistical Computing, Vienna, Austria).

### Ethical statement

This research adhered to the Association for the Study of Animal Behaviour/Animal Behaviour Society Guidelines for the Use of Animals in Research, the legal requirements of the country in which the work was carried out and all institutional guidelines.

### Availability of data and materials

The mass spectrometry proteomics data have been deposited to the ProteomeXchange Consortium [76] via the PRIDE partner repository with the dataset identifier PXD002900. Morphological data are provided as supplementary information in Additional file 3.

## Additional files

Additional file 1: **A summary of the proteomics data analysis from Progenesis QI with abundances normalized using all 383 proteins.** Progenesis QI html report file for the proteins identified and quantified across the four treatment groups. At the top of the file is a summary table of the protein-level average normalised abundances, ranked according to Mascot protein database search score. This is followed by peptide-level abundances, in tabular form, for each protein, on a protein-byprotein basis. Data are split by treatment groups according to high or low sperm competition risk. At the bottom of the report file are plots summarizing the between treatment group abundance data, at protein level. Those proteins ‘tagged’ with a red or green circle are those that were significantly changing in abundance between the treatment groups, according to ANOVA tests at p < 0.05 or p < 0.01 (respectively). Also included are the Top3 protein abundances, normalised to all proteins, in a .csv file. (ZIP 4169 kb)

Additional file 2: **A summary of the proteomics data analysis from Progenesis QI with abundances normalised using only 31 ejaculated proteins.** Progenesis QI html report file for the proteins identified and quantified across the four treatment groups. At the top of the file is a summary table of the protein-level average normalised abundances, ranked according to Mascot protein database search score. This is followed by peptide-level abundances, in tabular form, for each protein, on a protein-by-protein basis. Data are split by treatment groups according to high or low sperm competition risk, and high or low potential mating rate. At the bottom of the report file are plots summarizing the between treatment group abundance data, at protein level. Those proteins ‘tagged’ with a red or green circle are those that were significantly changing in abundance between the treatment groups, according to ANOVA tests at p < 0.05 or p < 0.01 (respectively). Also included are the Top3 protein abundances, normalised to the 31 ejaculated proteins, in a .csv file. (ZIP 4957 kb)

**Morphological data.** Body mass, testes mass, seminal vesicles mass, daily sperm production rate and epididymal sperm numbers data for subject male house mice, classified according to experimental treatment group: high sperm competition risk, high potential mating rate (4m4f); low sperm competition risk, low potential mating rate (2m2f); high sperm competition risk, low potential mating rate (4m2f); low sperm competition risk, high potential mating rate (2m4f). (XLSX 45 kb)
