# Supplementary figures and images for: Sperm competition risk drives plasticity in seminal fluid composition (part 2 of 4)
Source: BMC Biol. 2015 Oct 27;13:87. doi: 10.1186/s12915-015-0197-2 (PMC4624372; doi:10.1186/s12915-015-0197-2)

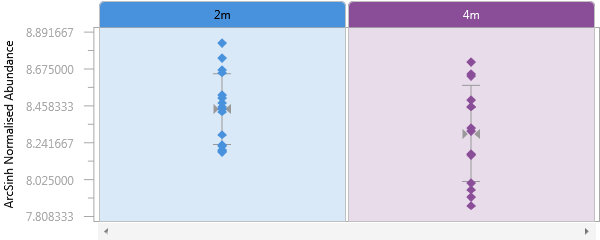

Supplement: Supplementary file 1 — A summary of the proteomics data analysis from Progenesis QI with abundances normalized using all 383 proteins. Progenesis QI html report file for the proteins identified and quantified across the four treatment groups. At the top of the file is a summary table of the protein-level average normalised abundances, ranked according to Mascot protein database search score. This is followed by peptide-level abundances, in tabular form, for each protein, on a protein-byprotein basis. Data are split by treatment groups according to high or low sperm competition risk. At the bottom of the report file are plots summarizing the between treatment group abundance data, at protein level. Those proteins ‘tagged’ with a red or green circle are those that were significantly changing in abundance between the treatment groups, according to ANOVA tests at p < 0.05 or p < 0.01 (respectively). Also included are the Top3 protein abundances, normalised to all proteins, in a .csv file. (ZIP 4169 kb) [file 12915_2015_197_MOESM1_ESM.zip › Additional File 1_2-way/Additional File 1_2-way analysis_Ramm et al_files/protein189_graph.png]

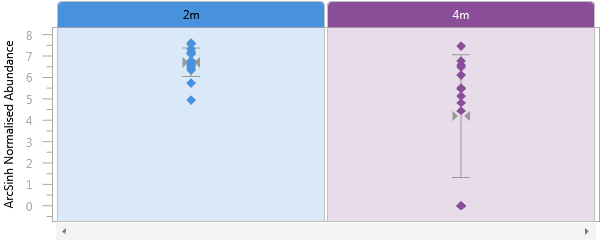

Supplement: Supplementary file 1 — A summary of the proteomics data analysis from Progenesis QI with abundances normalized using all 383 proteins. Progenesis QI html report file for the proteins identified and quantified across the four treatment groups. At the top of the file is a summary table of the protein-level average normalised abundances, ranked according to Mascot protein database search score. This is followed by peptide-level abundances, in tabular form, for each protein, on a protein-byprotein basis. Data are split by treatment groups according to high or low sperm competition risk. At the bottom of the report file are plots summarizing the between treatment group abundance data, at protein level. Those proteins ‘tagged’ with a red or green circle are those that were significantly changing in abundance between the treatment groups, according to ANOVA tests at p < 0.05 or p < 0.01 (respectively). Also included are the Top3 protein abundances, normalised to all proteins, in a .csv file. (ZIP 4169 kb) [file 12915_2015_197_MOESM1_ESM.zip › Additional File 1_2-way/Additional File 1_2-way analysis_Ramm et al_files/protein18_graph.png]

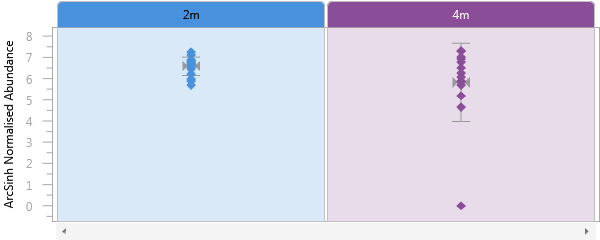

Supplement: Supplementary file 1 — A summary of the proteomics data analysis from Progenesis QI with abundances normalized using all 383 proteins. Progenesis QI html report file for the proteins identified and quantified across the four treatment groups. At the top of the file is a summary table of the protein-level average normalised abundances, ranked according to Mascot protein database search score. This is followed by peptide-level abundances, in tabular form, for each protein, on a protein-byprotein basis. Data are split by treatment groups according to high or low sperm competition risk. At the bottom of the report file are plots summarizing the between treatment group abundance data, at protein level. Those proteins ‘tagged’ with a red or green circle are those that were significantly changing in abundance between the treatment groups, according to ANOVA tests at p < 0.05 or p < 0.01 (respectively). Also included are the Top3 protein abundances, normalised to all proteins, in a .csv file. (ZIP 4169 kb) [file 12915_2015_197_MOESM1_ESM.zip › Additional File 1_2-way/Additional File 1_2-way analysis_Ramm et al_files/protein190_graph.png]

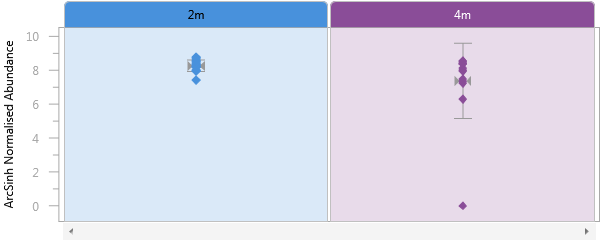

Supplement: Supplementary file 1 — A summary of the proteomics data analysis from Progenesis QI with abundances normalized using all 383 proteins. Progenesis QI html report file for the proteins identified and quantified across the four treatment groups. At the top of the file is a summary table of the protein-level average normalised abundances, ranked according to Mascot protein database search score. This is followed by peptide-level abundances, in tabular form, for each protein, on a protein-byprotein basis. Data are split by treatment groups according to high or low sperm competition risk. At the bottom of the report file are plots summarizing the between treatment group abundance data, at protein level. Those proteins ‘tagged’ with a red or green circle are those that were significantly changing in abundance between the treatment groups, according to ANOVA tests at p < 0.05 or p < 0.01 (respectively). Also included are the Top3 protein abundances, normalised to all proteins, in a .csv file. (ZIP 4169 kb) [file 12915_2015_197_MOESM1_ESM.zip › Additional File 1_2-way/Additional File 1_2-way analysis_Ramm et al_files/protein191_graph.png]

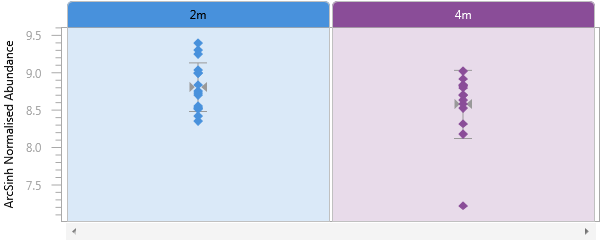

Supplement: Supplementary file 1 — A summary of the proteomics data analysis from Progenesis QI with abundances normalized using all 383 proteins. Progenesis QI html report file for the proteins identified and quantified across the four treatment groups. At the top of the file is a summary table of the protein-level average normalised abundances, ranked according to Mascot protein database search score. This is followed by peptide-level abundances, in tabular form, for each protein, on a protein-byprotein basis. Data are split by treatment groups according to high or low sperm competition risk. At the bottom of the report file are plots summarizing the between treatment group abundance data, at protein level. Those proteins ‘tagged’ with a red or green circle are those that were significantly changing in abundance between the treatment groups, according to ANOVA tests at p < 0.05 or p < 0.01 (respectively). Also included are the Top3 protein abundances, normalised to all proteins, in a .csv file. (ZIP 4169 kb) [file 12915_2015_197_MOESM1_ESM.zip › Additional File 1_2-way/Additional File 1_2-way analysis_Ramm et al_files/protein192_graph.png]

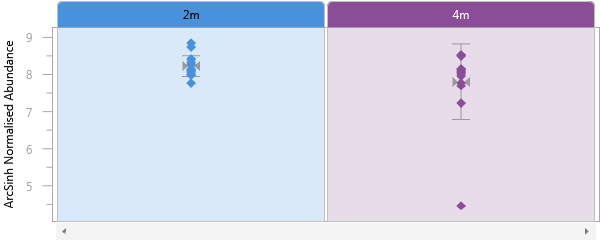

Supplement: Supplementary file 1 — A summary of the proteomics data analysis from Progenesis QI with abundances normalized using all 383 proteins. Progenesis QI html report file for the proteins identified and quantified across the four treatment groups. At the top of the file is a summary table of the protein-level average normalised abundances, ranked according to Mascot protein database search score. This is followed by peptide-level abundances, in tabular form, for each protein, on a protein-byprotein basis. Data are split by treatment groups according to high or low sperm competition risk. At the bottom of the report file are plots summarizing the between treatment group abundance data, at protein level. Those proteins ‘tagged’ with a red or green circle are those that were significantly changing in abundance between the treatment groups, according to ANOVA tests at p < 0.05 or p < 0.01 (respectively). Also included are the Top3 protein abundances, normalised to all proteins, in a .csv file. (ZIP 4169 kb) [file 12915_2015_197_MOESM1_ESM.zip › Additional File 1_2-way/Additional File 1_2-way analysis_Ramm et al_files/protein193_graph.png]

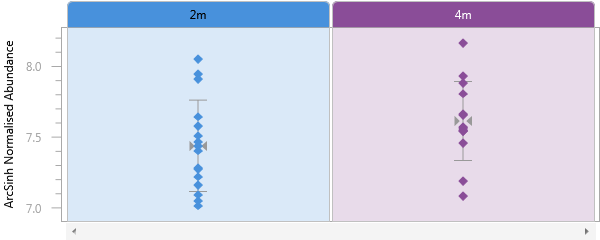

Supplement: Supplementary file 1 — A summary of the proteomics data analysis from Progenesis QI with abundances normalized using all 383 proteins. Progenesis QI html report file for the proteins identified and quantified across the four treatment groups. At the top of the file is a summary table of the protein-level average normalised abundances, ranked according to Mascot protein database search score. This is followed by peptide-level abundances, in tabular form, for each protein, on a protein-byprotein basis. Data are split by treatment groups according to high or low sperm competition risk. At the bottom of the report file are plots summarizing the between treatment group abundance data, at protein level. Those proteins ‘tagged’ with a red or green circle are those that were significantly changing in abundance between the treatment groups, according to ANOVA tests at p < 0.05 or p < 0.01 (respectively). Also included are the Top3 protein abundances, normalised to all proteins, in a .csv file. (ZIP 4169 kb) [file 12915_2015_197_MOESM1_ESM.zip › Additional File 1_2-way/Additional File 1_2-way analysis_Ramm et al_files/protein194_graph.png]

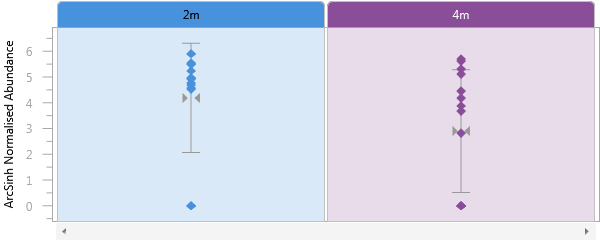

Supplement: Supplementary file 1 — A summary of the proteomics data analysis from Progenesis QI with abundances normalized using all 383 proteins. Progenesis QI html report file for the proteins identified and quantified across the four treatment groups. At the top of the file is a summary table of the protein-level average normalised abundances, ranked according to Mascot protein database search score. This is followed by peptide-level abundances, in tabular form, for each protein, on a protein-byprotein basis. Data are split by treatment groups according to high or low sperm competition risk. At the bottom of the report file are plots summarizing the between treatment group abundance data, at protein level. Those proteins ‘tagged’ with a red or green circle are those that were significantly changing in abundance between the treatment groups, according to ANOVA tests at p < 0.05 or p < 0.01 (respectively). Also included are the Top3 protein abundances, normalised to all proteins, in a .csv file. (ZIP 4169 kb) [file 12915_2015_197_MOESM1_ESM.zip › Additional File 1_2-way/Additional File 1_2-way analysis_Ramm et al_files/protein195_graph.png]

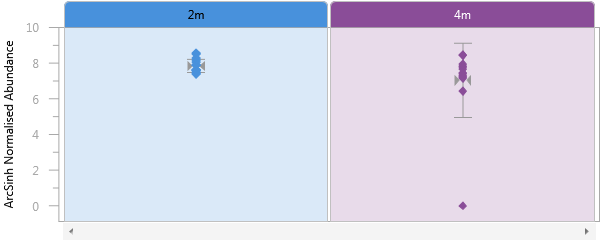

Supplement: Supplementary file 1 — A summary of the proteomics data analysis from Progenesis QI with abundances normalized using all 383 proteins. Progenesis QI html report file for the proteins identified and quantified across the four treatment groups. At the top of the file is a summary table of the protein-level average normalised abundances, ranked according to Mascot protein database search score. This is followed by peptide-level abundances, in tabular form, for each protein, on a protein-byprotein basis. Data are split by treatment groups according to high or low sperm competition risk. At the bottom of the report file are plots summarizing the between treatment group abundance data, at protein level. Those proteins ‘tagged’ with a red or green circle are those that were significantly changing in abundance between the treatment groups, according to ANOVA tests at p < 0.05 or p < 0.01 (respectively). Also included are the Top3 protein abundances, normalised to all proteins, in a .csv file. (ZIP 4169 kb) [file 12915_2015_197_MOESM1_ESM.zip › Additional File 1_2-way/Additional File 1_2-way analysis_Ramm et al_files/protein196_graph.png]

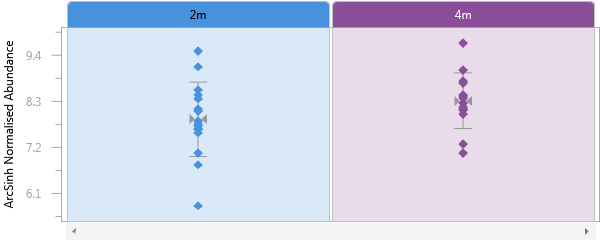

Supplement: Supplementary file 1 — A summary of the proteomics data analysis from Progenesis QI with abundances normalized using all 383 proteins. Progenesis QI html report file for the proteins identified and quantified across the four treatment groups. At the top of the file is a summary table of the protein-level average normalised abundances, ranked according to Mascot protein database search score. This is followed by peptide-level abundances, in tabular form, for each protein, on a protein-byprotein basis. Data are split by treatment groups according to high or low sperm competition risk. At the bottom of the report file are plots summarizing the between treatment group abundance data, at protein level. Those proteins ‘tagged’ with a red or green circle are those that were significantly changing in abundance between the treatment groups, according to ANOVA tests at p < 0.05 or p < 0.01 (respectively). Also included are the Top3 protein abundances, normalised to all proteins, in a .csv file. (ZIP 4169 kb) [file 12915_2015_197_MOESM1_ESM.zip › Additional File 1_2-way/Additional File 1_2-way analysis_Ramm et al_files/protein197_graph.png]

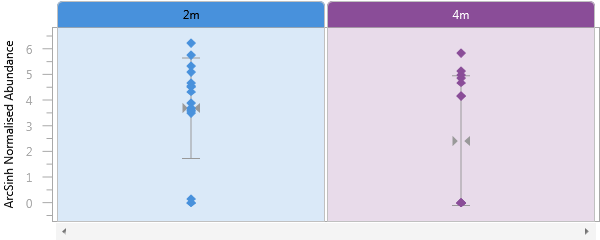

Supplement: Supplementary file 1 — A summary of the proteomics data analysis from Progenesis QI with abundances normalized using all 383 proteins. Progenesis QI html report file for the proteins identified and quantified across the four treatment groups. At the top of the file is a summary table of the protein-level average normalised abundances, ranked according to Mascot protein database search score. This is followed by peptide-level abundances, in tabular form, for each protein, on a protein-byprotein basis. Data are split by treatment groups according to high or low sperm competition risk. At the bottom of the report file are plots summarizing the between treatment group abundance data, at protein level. Those proteins ‘tagged’ with a red or green circle are those that were significantly changing in abundance between the treatment groups, according to ANOVA tests at p < 0.05 or p < 0.01 (respectively). Also included are the Top3 protein abundances, normalised to all proteins, in a .csv file. (ZIP 4169 kb) [file 12915_2015_197_MOESM1_ESM.zip › Additional File 1_2-way/Additional File 1_2-way analysis_Ramm et al_files/protein198_graph.png]

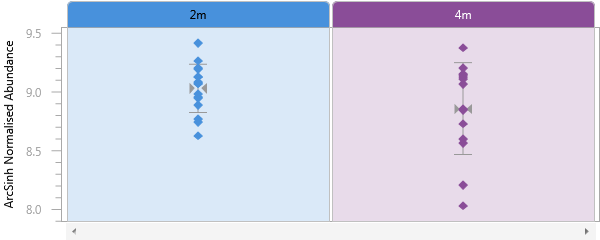

Supplement: Supplementary file 1 — A summary of the proteomics data analysis from Progenesis QI with abundances normalized using all 383 proteins. Progenesis QI html report file for the proteins identified and quantified across the four treatment groups. At the top of the file is a summary table of the protein-level average normalised abundances, ranked according to Mascot protein database search score. This is followed by peptide-level abundances, in tabular form, for each protein, on a protein-byprotein basis. Data are split by treatment groups according to high or low sperm competition risk. At the bottom of the report file are plots summarizing the between treatment group abundance data, at protein level. Those proteins ‘tagged’ with a red or green circle are those that were significantly changing in abundance between the treatment groups, according to ANOVA tests at p < 0.05 or p < 0.01 (respectively). Also included are the Top3 protein abundances, normalised to all proteins, in a .csv file. (ZIP 4169 kb) [file 12915_2015_197_MOESM1_ESM.zip › Additional File 1_2-way/Additional File 1_2-way analysis_Ramm et al_files/protein199_graph.png]

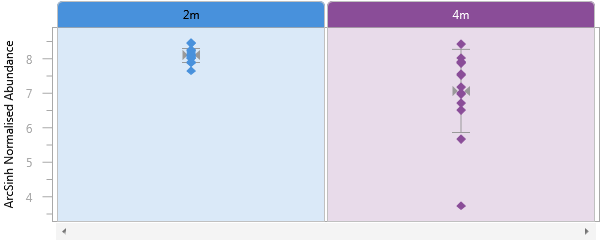

Supplement: Supplementary file 1 — A summary of the proteomics data analysis from Progenesis QI with abundances normalized using all 383 proteins. Progenesis QI html report file for the proteins identified and quantified across the four treatment groups. At the top of the file is a summary table of the protein-level average normalised abundances, ranked according to Mascot protein database search score. This is followed by peptide-level abundances, in tabular form, for each protein, on a protein-byprotein basis. Data are split by treatment groups according to high or low sperm competition risk. At the bottom of the report file are plots summarizing the between treatment group abundance data, at protein level. Those proteins ‘tagged’ with a red or green circle are those that were significantly changing in abundance between the treatment groups, according to ANOVA tests at p < 0.05 or p < 0.01 (respectively). Also included are the Top3 protein abundances, normalised to all proteins, in a .csv file. (ZIP 4169 kb) [file 12915_2015_197_MOESM1_ESM.zip › Additional File 1_2-way/Additional File 1_2-way analysis_Ramm et al_files/protein19_graph.png]

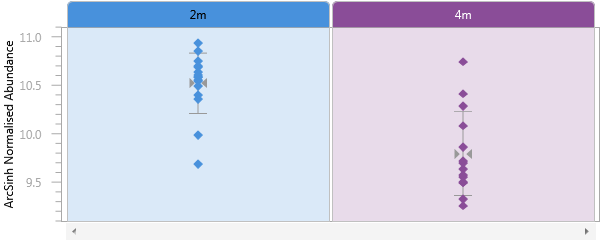

Supplement: Supplementary file 1 — A summary of the proteomics data analysis from Progenesis QI with abundances normalized using all 383 proteins. Progenesis QI html report file for the proteins identified and quantified across the four treatment groups. At the top of the file is a summary table of the protein-level average normalised abundances, ranked according to Mascot protein database search score. This is followed by peptide-level abundances, in tabular form, for each protein, on a protein-byprotein basis. Data are split by treatment groups according to high or low sperm competition risk. At the bottom of the report file are plots summarizing the between treatment group abundance data, at protein level. Those proteins ‘tagged’ with a red or green circle are those that were significantly changing in abundance between the treatment groups, according to ANOVA tests at p < 0.05 or p < 0.01 (respectively). Also included are the Top3 protein abundances, normalised to all proteins, in a .csv file. (ZIP 4169 kb) [file 12915_2015_197_MOESM1_ESM.zip › Additional File 1_2-way/Additional File 1_2-way analysis_Ramm et al_files/protein1_graph.png]

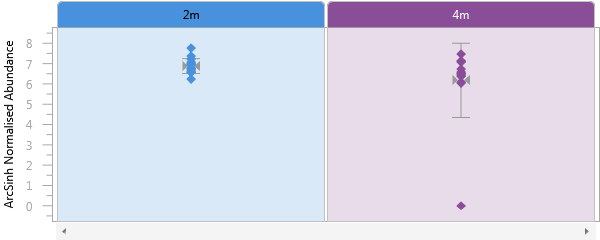

Supplement: Supplementary file 1 — A summary of the proteomics data analysis from Progenesis QI with abundances normalized using all 383 proteins. Progenesis QI html report file for the proteins identified and quantified across the four treatment groups. At the top of the file is a summary table of the protein-level average normalised abundances, ranked according to Mascot protein database search score. This is followed by peptide-level abundances, in tabular form, for each protein, on a protein-byprotein basis. Data are split by treatment groups according to high or low sperm competition risk. At the bottom of the report file are plots summarizing the between treatment group abundance data, at protein level. Those proteins ‘tagged’ with a red or green circle are those that were significantly changing in abundance between the treatment groups, according to ANOVA tests at p < 0.05 or p < 0.01 (respectively). Also included are the Top3 protein abundances, normalised to all proteins, in a .csv file. (ZIP 4169 kb) [file 12915_2015_197_MOESM1_ESM.zip › Additional File 1_2-way/Additional File 1_2-way analysis_Ramm et al_files/protein200_graph.png]

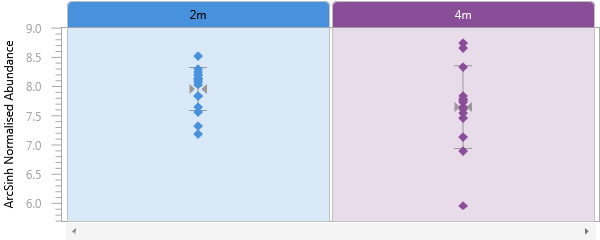

Supplement: Supplementary file 1 — A summary of the proteomics data analysis from Progenesis QI with abundances normalized using all 383 proteins. Progenesis QI html report file for the proteins identified and quantified across the four treatment groups. At the top of the file is a summary table of the protein-level average normalised abundances, ranked according to Mascot protein database search score. This is followed by peptide-level abundances, in tabular form, for each protein, on a protein-byprotein basis. Data are split by treatment groups according to high or low sperm competition risk. At the bottom of the report file are plots summarizing the between treatment group abundance data, at protein level. Those proteins ‘tagged’ with a red or green circle are those that were significantly changing in abundance between the treatment groups, according to ANOVA tests at p < 0.05 or p < 0.01 (respectively). Also included are the Top3 protein abundances, normalised to all proteins, in a .csv file. (ZIP 4169 kb) [file 12915_2015_197_MOESM1_ESM.zip › Additional File 1_2-way/Additional File 1_2-way analysis_Ramm et al_files/protein201_graph.png]

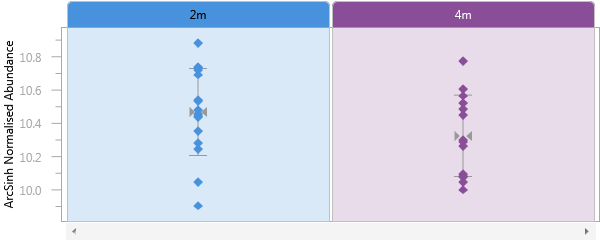

Supplement: Supplementary file 1 — A summary of the proteomics data analysis from Progenesis QI with abundances normalized using all 383 proteins. Progenesis QI html report file for the proteins identified and quantified across the four treatment groups. At the top of the file is a summary table of the protein-level average normalised abundances, ranked according to Mascot protein database search score. This is followed by peptide-level abundances, in tabular form, for each protein, on a protein-byprotein basis. Data are split by treatment groups according to high or low sperm competition risk. At the bottom of the report file are plots summarizing the between treatment group abundance data, at protein level. Those proteins ‘tagged’ with a red or green circle are those that were significantly changing in abundance between the treatment groups, according to ANOVA tests at p < 0.05 or p < 0.01 (respectively). Also included are the Top3 protein abundances, normalised to all proteins, in a .csv file. (ZIP 4169 kb) [file 12915_2015_197_MOESM1_ESM.zip › Additional File 1_2-way/Additional File 1_2-way analysis_Ramm et al_files/protein202_graph.png]

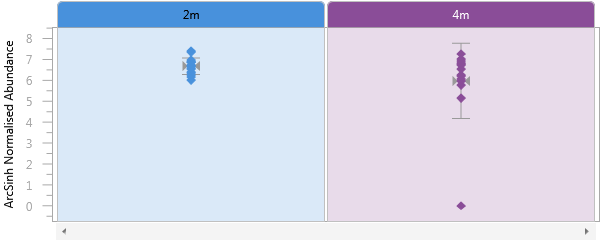

Supplement: Supplementary file 1 — A summary of the proteomics data analysis from Progenesis QI with abundances normalized using all 383 proteins. Progenesis QI html report file for the proteins identified and quantified across the four treatment groups. At the top of the file is a summary table of the protein-level average normalised abundances, ranked according to Mascot protein database search score. This is followed by peptide-level abundances, in tabular form, for each protein, on a protein-byprotein basis. Data are split by treatment groups according to high or low sperm competition risk. At the bottom of the report file are plots summarizing the between treatment group abundance data, at protein level. Those proteins ‘tagged’ with a red or green circle are those that were significantly changing in abundance between the treatment groups, according to ANOVA tests at p < 0.05 or p < 0.01 (respectively). Also included are the Top3 protein abundances, normalised to all proteins, in a .csv file. (ZIP 4169 kb) [file 12915_2015_197_MOESM1_ESM.zip › Additional File 1_2-way/Additional File 1_2-way analysis_Ramm et al_files/protein203_graph.png]

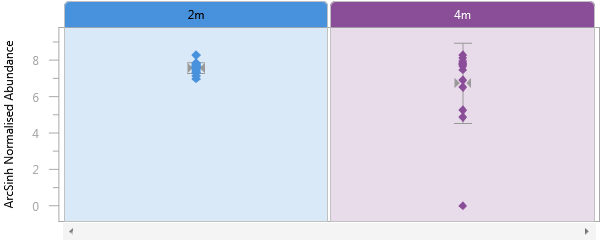

Supplement: Supplementary file 1 — A summary of the proteomics data analysis from Progenesis QI with abundances normalized using all 383 proteins. Progenesis QI html report file for the proteins identified and quantified across the four treatment groups. At the top of the file is a summary table of the protein-level average normalised abundances, ranked according to Mascot protein database search score. This is followed by peptide-level abundances, in tabular form, for each protein, on a protein-byprotein basis. Data are split by treatment groups according to high or low sperm competition risk. At the bottom of the report file are plots summarizing the between treatment group abundance data, at protein level. Those proteins ‘tagged’ with a red or green circle are those that were significantly changing in abundance between the treatment groups, according to ANOVA tests at p < 0.05 or p < 0.01 (respectively). Also included are the Top3 protein abundances, normalised to all proteins, in a .csv file. (ZIP 4169 kb) [file 12915_2015_197_MOESM1_ESM.zip › Additional File 1_2-way/Additional File 1_2-way analysis_Ramm et al_files/protein204_graph.png]

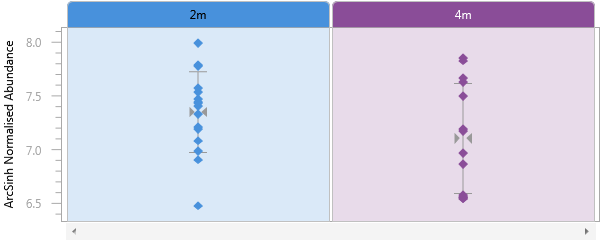

Supplement: Supplementary file 1 — A summary of the proteomics data analysis from Progenesis QI with abundances normalized using all 383 proteins. Progenesis QI html report file for the proteins identified and quantified across the four treatment groups. At the top of the file is a summary table of the protein-level average normalised abundances, ranked according to Mascot protein database search score. This is followed by peptide-level abundances, in tabular form, for each protein, on a protein-byprotein basis. Data are split by treatment groups according to high or low sperm competition risk. At the bottom of the report file are plots summarizing the between treatment group abundance data, at protein level. Those proteins ‘tagged’ with a red or green circle are those that were significantly changing in abundance between the treatment groups, according to ANOVA tests at p < 0.05 or p < 0.01 (respectively). Also included are the Top3 protein abundances, normalised to all proteins, in a .csv file. (ZIP 4169 kb) [file 12915_2015_197_MOESM1_ESM.zip › Additional File 1_2-way/Additional File 1_2-way analysis_Ramm et al_files/protein205_graph.png]

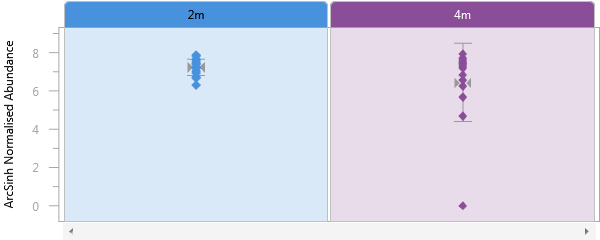

Supplement: Supplementary file 1 — A summary of the proteomics data analysis from Progenesis QI with abundances normalized using all 383 proteins. Progenesis QI html report file for the proteins identified and quantified across the four treatment groups. At the top of the file is a summary table of the protein-level average normalised abundances, ranked according to Mascot protein database search score. This is followed by peptide-level abundances, in tabular form, for each protein, on a protein-byprotein basis. Data are split by treatment groups according to high or low sperm competition risk. At the bottom of the report file are plots summarizing the between treatment group abundance data, at protein level. Those proteins ‘tagged’ with a red or green circle are those that were significantly changing in abundance between the treatment groups, according to ANOVA tests at p < 0.05 or p < 0.01 (respectively). Also included are the Top3 protein abundances, normalised to all proteins, in a .csv file. (ZIP 4169 kb) [file 12915_2015_197_MOESM1_ESM.zip › Additional File 1_2-way/Additional File 1_2-way analysis_Ramm et al_files/protein206_graph.png]

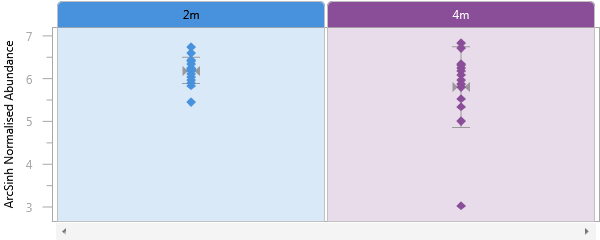

Supplement: Supplementary file 1 — A summary of the proteomics data analysis from Progenesis QI with abundances normalized using all 383 proteins. Progenesis QI html report file for the proteins identified and quantified across the four treatment groups. At the top of the file is a summary table of the protein-level average normalised abundances, ranked according to Mascot protein database search score. This is followed by peptide-level abundances, in tabular form, for each protein, on a protein-byprotein basis. Data are split by treatment groups according to high or low sperm competition risk. At the bottom of the report file are plots summarizing the between treatment group abundance data, at protein level. Those proteins ‘tagged’ with a red or green circle are those that were significantly changing in abundance between the treatment groups, according to ANOVA tests at p < 0.05 or p < 0.01 (respectively). Also included are the Top3 protein abundances, normalised to all proteins, in a .csv file. (ZIP 4169 kb) [file 12915_2015_197_MOESM1_ESM.zip › Additional File 1_2-way/Additional File 1_2-way analysis_Ramm et al_files/protein207_graph.png]

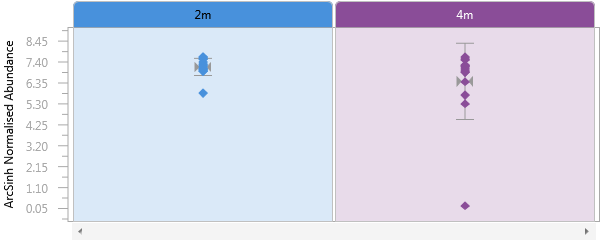

Supplement: Supplementary file 1 — A summary of the proteomics data analysis from Progenesis QI with abundances normalized using all 383 proteins. Progenesis QI html report file for the proteins identified and quantified across the four treatment groups. At the top of the file is a summary table of the protein-level average normalised abundances, ranked according to Mascot protein database search score. This is followed by peptide-level abundances, in tabular form, for each protein, on a protein-byprotein basis. Data are split by treatment groups according to high or low sperm competition risk. At the bottom of the report file are plots summarizing the between treatment group abundance data, at protein level. Those proteins ‘tagged’ with a red or green circle are those that were significantly changing in abundance between the treatment groups, according to ANOVA tests at p < 0.05 or p < 0.01 (respectively). Also included are the Top3 protein abundances, normalised to all proteins, in a .csv file. (ZIP 4169 kb) [file 12915_2015_197_MOESM1_ESM.zip › Additional File 1_2-way/Additional File 1_2-way analysis_Ramm et al_files/protein208_graph.png]

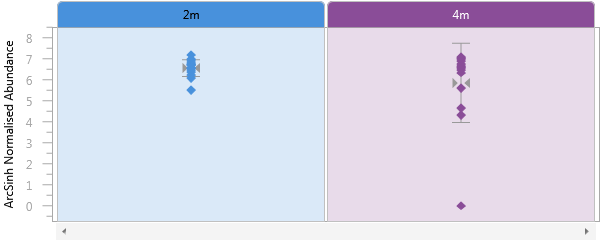

Supplement: Supplementary file 1 — A summary of the proteomics data analysis from Progenesis QI with abundances normalized using all 383 proteins. Progenesis QI html report file for the proteins identified and quantified across the four treatment groups. At the top of the file is a summary table of the protein-level average normalised abundances, ranked according to Mascot protein database search score. This is followed by peptide-level abundances, in tabular form, for each protein, on a protein-byprotein basis. Data are split by treatment groups according to high or low sperm competition risk. At the bottom of the report file are plots summarizing the between treatment group abundance data, at protein level. Those proteins ‘tagged’ with a red or green circle are those that were significantly changing in abundance between the treatment groups, according to ANOVA tests at p < 0.05 or p < 0.01 (respectively). Also included are the Top3 protein abundances, normalised to all proteins, in a .csv file. (ZIP 4169 kb) [file 12915_2015_197_MOESM1_ESM.zip › Additional File 1_2-way/Additional File 1_2-way analysis_Ramm et al_files/protein209_graph.png]

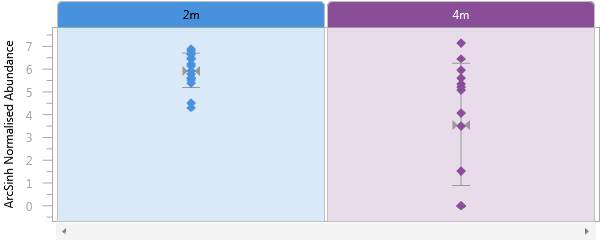

Supplement: Supplementary file 1 — A summary of the proteomics data analysis from Progenesis QI with abundances normalized using all 383 proteins. Progenesis QI html report file for the proteins identified and quantified across the four treatment groups. At the top of the file is a summary table of the protein-level average normalised abundances, ranked according to Mascot protein database search score. This is followed by peptide-level abundances, in tabular form, for each protein, on a protein-byprotein basis. Data are split by treatment groups according to high or low sperm competition risk. At the bottom of the report file are plots summarizing the between treatment group abundance data, at protein level. Those proteins ‘tagged’ with a red or green circle are those that were significantly changing in abundance between the treatment groups, according to ANOVA tests at p < 0.05 or p < 0.01 (respectively). Also included are the Top3 protein abundances, normalised to all proteins, in a .csv file. (ZIP 4169 kb) [file 12915_2015_197_MOESM1_ESM.zip › Additional File 1_2-way/Additional File 1_2-way analysis_Ramm et al_files/protein20_graph.png]

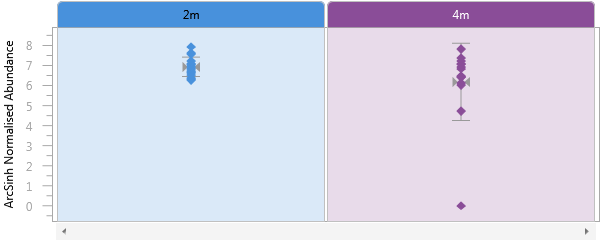

Supplement: Supplementary file 1 — A summary of the proteomics data analysis from Progenesis QI with abundances normalized using all 383 proteins. Progenesis QI html report file for the proteins identified and quantified across the four treatment groups. At the top of the file is a summary table of the protein-level average normalised abundances, ranked according to Mascot protein database search score. This is followed by peptide-level abundances, in tabular form, for each protein, on a protein-byprotein basis. Data are split by treatment groups according to high or low sperm competition risk. At the bottom of the report file are plots summarizing the between treatment group abundance data, at protein level. Those proteins ‘tagged’ with a red or green circle are those that were significantly changing in abundance between the treatment groups, according to ANOVA tests at p < 0.05 or p < 0.01 (respectively). Also included are the Top3 protein abundances, normalised to all proteins, in a .csv file. (ZIP 4169 kb) [file 12915_2015_197_MOESM1_ESM.zip › Additional File 1_2-way/Additional File 1_2-way analysis_Ramm et al_files/protein210_graph.png]

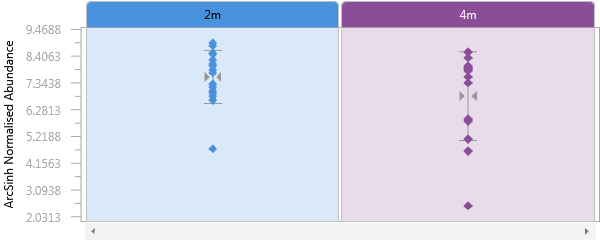

Supplement: Supplementary file 1 — A summary of the proteomics data analysis from Progenesis QI with abundances normalized using all 383 proteins. Progenesis QI html report file for the proteins identified and quantified across the four treatment groups. At the top of the file is a summary table of the protein-level average normalised abundances, ranked according to Mascot protein database search score. This is followed by peptide-level abundances, in tabular form, for each protein, on a protein-byprotein basis. Data are split by treatment groups according to high or low sperm competition risk. At the bottom of the report file are plots summarizing the between treatment group abundance data, at protein level. Those proteins ‘tagged’ with a red or green circle are those that were significantly changing in abundance between the treatment groups, according to ANOVA tests at p < 0.05 or p < 0.01 (respectively). Also included are the Top3 protein abundances, normalised to all proteins, in a .csv file. (ZIP 4169 kb) [file 12915_2015_197_MOESM1_ESM.zip › Additional File 1_2-way/Additional File 1_2-way analysis_Ramm et al_files/protein211_graph.png]

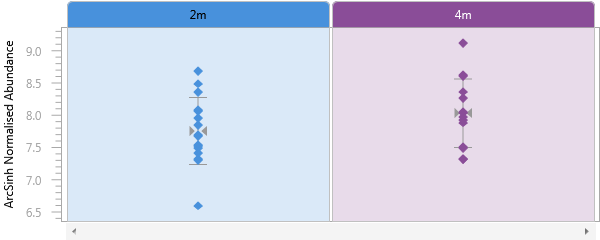

Supplement: Supplementary file 1 — A summary of the proteomics data analysis from Progenesis QI with abundances normalized using all 383 proteins. Progenesis QI html report file for the proteins identified and quantified across the four treatment groups. At the top of the file is a summary table of the protein-level average normalised abundances, ranked according to Mascot protein database search score. This is followed by peptide-level abundances, in tabular form, for each protein, on a protein-byprotein basis. Data are split by treatment groups according to high or low sperm competition risk. At the bottom of the report file are plots summarizing the between treatment group abundance data, at protein level. Those proteins ‘tagged’ with a red or green circle are those that were significantly changing in abundance between the treatment groups, according to ANOVA tests at p < 0.05 or p < 0.01 (respectively). Also included are the Top3 protein abundances, normalised to all proteins, in a .csv file. (ZIP 4169 kb) [file 12915_2015_197_MOESM1_ESM.zip › Additional File 1_2-way/Additional File 1_2-way analysis_Ramm et al_files/protein212_graph.png]

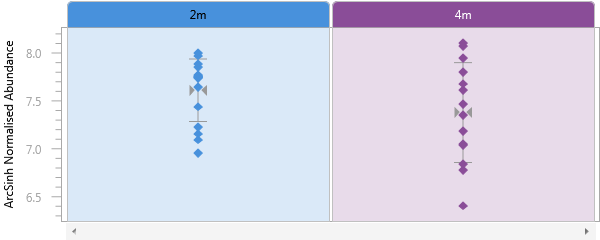

Supplement: Supplementary file 1 — A summary of the proteomics data analysis from Progenesis QI with abundances normalized using all 383 proteins. Progenesis QI html report file for the proteins identified and quantified across the four treatment groups. At the top of the file is a summary table of the protein-level average normalised abundances, ranked according to Mascot protein database search score. This is followed by peptide-level abundances, in tabular form, for each protein, on a protein-byprotein basis. Data are split by treatment groups according to high or low sperm competition risk. At the bottom of the report file are plots summarizing the between treatment group abundance data, at protein level. Those proteins ‘tagged’ with a red or green circle are those that were significantly changing in abundance between the treatment groups, according to ANOVA tests at p < 0.05 or p < 0.01 (respectively). Also included are the Top3 protein abundances, normalised to all proteins, in a .csv file. (ZIP 4169 kb) [file 12915_2015_197_MOESM1_ESM.zip › Additional File 1_2-way/Additional File 1_2-way analysis_Ramm et al_files/protein213_graph.png]

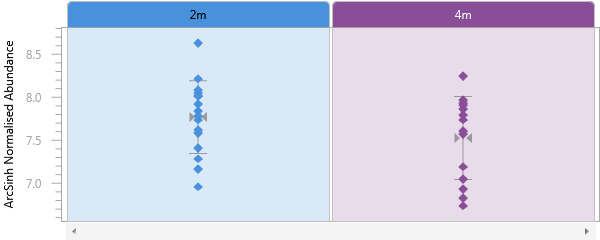

Supplement: Supplementary file 1 — A summary of the proteomics data analysis from Progenesis QI with abundances normalized using all 383 proteins. Progenesis QI html report file for the proteins identified and quantified across the four treatment groups. At the top of the file is a summary table of the protein-level average normalised abundances, ranked according to Mascot protein database search score. This is followed by peptide-level abundances, in tabular form, for each protein, on a protein-byprotein basis. Data are split by treatment groups according to high or low sperm competition risk. At the bottom of the report file are plots summarizing the between treatment group abundance data, at protein level. Those proteins ‘tagged’ with a red or green circle are those that were significantly changing in abundance between the treatment groups, according to ANOVA tests at p < 0.05 or p < 0.01 (respectively). Also included are the Top3 protein abundances, normalised to all proteins, in a .csv file. (ZIP 4169 kb) [file 12915_2015_197_MOESM1_ESM.zip › Additional File 1_2-way/Additional File 1_2-way analysis_Ramm et al_files/protein214_graph.png]

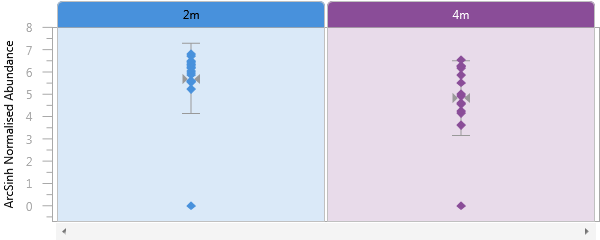

Supplement: Supplementary file 1 — A summary of the proteomics data analysis from Progenesis QI with abundances normalized using all 383 proteins. Progenesis QI html report file for the proteins identified and quantified across the four treatment groups. At the top of the file is a summary table of the protein-level average normalised abundances, ranked according to Mascot protein database search score. This is followed by peptide-level abundances, in tabular form, for each protein, on a protein-byprotein basis. Data are split by treatment groups according to high or low sperm competition risk. At the bottom of the report file are plots summarizing the between treatment group abundance data, at protein level. Those proteins ‘tagged’ with a red or green circle are those that were significantly changing in abundance between the treatment groups, according to ANOVA tests at p < 0.05 or p < 0.01 (respectively). Also included are the Top3 protein abundances, normalised to all proteins, in a .csv file. (ZIP 4169 kb) [file 12915_2015_197_MOESM1_ESM.zip › Additional File 1_2-way/Additional File 1_2-way analysis_Ramm et al_files/protein215_graph.png]

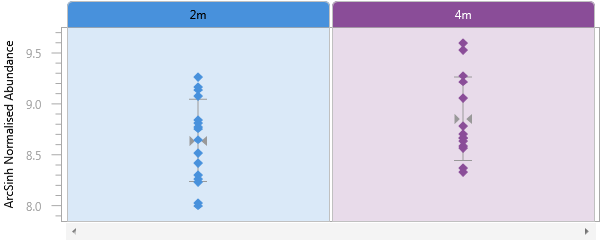

Supplement: Supplementary file 1 — A summary of the proteomics data analysis from Progenesis QI with abundances normalized using all 383 proteins. Progenesis QI html report file for the proteins identified and quantified across the four treatment groups. At the top of the file is a summary table of the protein-level average normalised abundances, ranked according to Mascot protein database search score. This is followed by peptide-level abundances, in tabular form, for each protein, on a protein-byprotein basis. Data are split by treatment groups according to high or low sperm competition risk. At the bottom of the report file are plots summarizing the between treatment group abundance data, at protein level. Those proteins ‘tagged’ with a red or green circle are those that were significantly changing in abundance between the treatment groups, according to ANOVA tests at p < 0.05 or p < 0.01 (respectively). Also included are the Top3 protein abundances, normalised to all proteins, in a .csv file. (ZIP 4169 kb) [file 12915_2015_197_MOESM1_ESM.zip › Additional File 1_2-way/Additional File 1_2-way analysis_Ramm et al_files/protein216_graph.png]

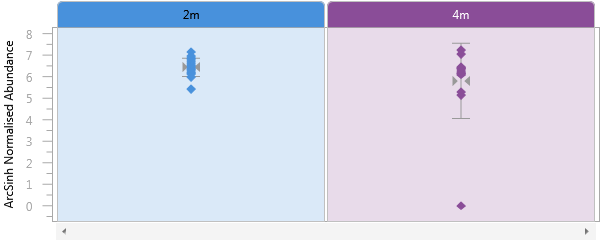

Supplement: Supplementary file 1 — A summary of the proteomics data analysis from Progenesis QI with abundances normalized using all 383 proteins. Progenesis QI html report file for the proteins identified and quantified across the four treatment groups. At the top of the file is a summary table of the protein-level average normalised abundances, ranked according to Mascot protein database search score. This is followed by peptide-level abundances, in tabular form, for each protein, on a protein-byprotein basis. Data are split by treatment groups according to high or low sperm competition risk. At the bottom of the report file are plots summarizing the between treatment group abundance data, at protein level. Those proteins ‘tagged’ with a red or green circle are those that were significantly changing in abundance between the treatment groups, according to ANOVA tests at p < 0.05 or p < 0.01 (respectively). Also included are the Top3 protein abundances, normalised to all proteins, in a .csv file. (ZIP 4169 kb) [file 12915_2015_197_MOESM1_ESM.zip › Additional File 1_2-way/Additional File 1_2-way analysis_Ramm et al_files/protein217_graph.png]

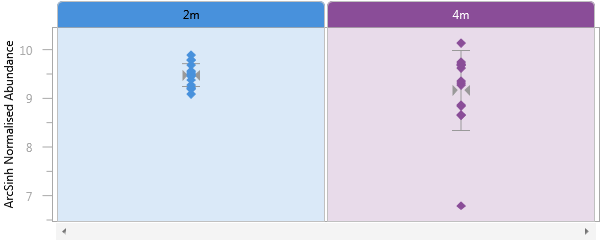

Supplement: Supplementary file 1 — A summary of the proteomics data analysis from Progenesis QI with abundances normalized using all 383 proteins. Progenesis QI html report file for the proteins identified and quantified across the four treatment groups. At the top of the file is a summary table of the protein-level average normalised abundances, ranked according to Mascot protein database search score. This is followed by peptide-level abundances, in tabular form, for each protein, on a protein-byprotein basis. Data are split by treatment groups according to high or low sperm competition risk. At the bottom of the report file are plots summarizing the between treatment group abundance data, at protein level. Those proteins ‘tagged’ with a red or green circle are those that were significantly changing in abundance between the treatment groups, according to ANOVA tests at p < 0.05 or p < 0.01 (respectively). Also included are the Top3 protein abundances, normalised to all proteins, in a .csv file. (ZIP 4169 kb) [file 12915_2015_197_MOESM1_ESM.zip › Additional File 1_2-way/Additional File 1_2-way analysis_Ramm et al_files/protein218_graph.png]

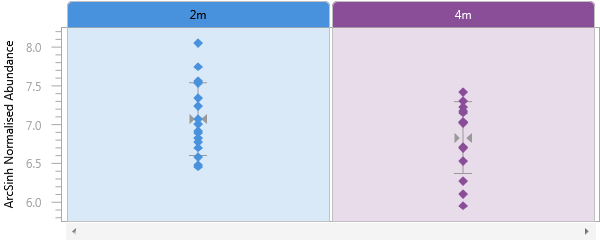

Supplement: Supplementary file 1 — A summary of the proteomics data analysis from Progenesis QI with abundances normalized using all 383 proteins. Progenesis QI html report file for the proteins identified and quantified across the four treatment groups. At the top of the file is a summary table of the protein-level average normalised abundances, ranked according to Mascot protein database search score. This is followed by peptide-level abundances, in tabular form, for each protein, on a protein-byprotein basis. Data are split by treatment groups according to high or low sperm competition risk. At the bottom of the report file are plots summarizing the between treatment group abundance data, at protein level. Those proteins ‘tagged’ with a red or green circle are those that were significantly changing in abundance between the treatment groups, according to ANOVA tests at p < 0.05 or p < 0.01 (respectively). Also included are the Top3 protein abundances, normalised to all proteins, in a .csv file. (ZIP 4169 kb) [file 12915_2015_197_MOESM1_ESM.zip › Additional File 1_2-way/Additional File 1_2-way analysis_Ramm et al_files/protein219_graph.png]

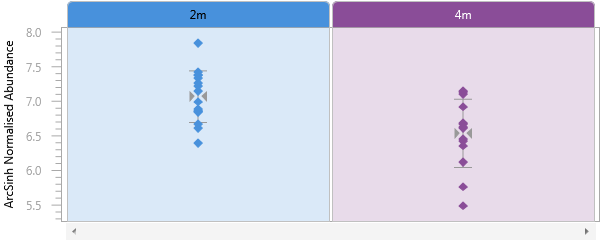

Supplement: Supplementary file 1 — A summary of the proteomics data analysis from Progenesis QI with abundances normalized using all 383 proteins. Progenesis QI html report file for the proteins identified and quantified across the four treatment groups. At the top of the file is a summary table of the protein-level average normalised abundances, ranked according to Mascot protein database search score. This is followed by peptide-level abundances, in tabular form, for each protein, on a protein-byprotein basis. Data are split by treatment groups according to high or low sperm competition risk. At the bottom of the report file are plots summarizing the between treatment group abundance data, at protein level. Those proteins ‘tagged’ with a red or green circle are those that were significantly changing in abundance between the treatment groups, according to ANOVA tests at p < 0.05 or p < 0.01 (respectively). Also included are the Top3 protein abundances, normalised to all proteins, in a .csv file. (ZIP 4169 kb) [file 12915_2015_197_MOESM1_ESM.zip › Additional File 1_2-way/Additional File 1_2-way analysis_Ramm et al_files/protein21_graph.png]

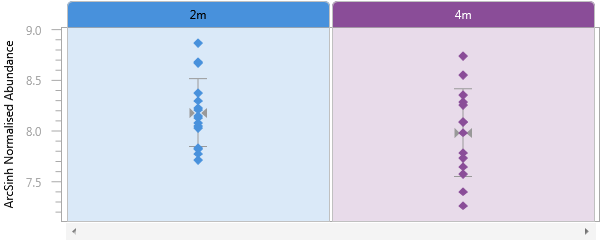

Supplement: Supplementary file 1 — A summary of the proteomics data analysis from Progenesis QI with abundances normalized using all 383 proteins. Progenesis QI html report file for the proteins identified and quantified across the four treatment groups. At the top of the file is a summary table of the protein-level average normalised abundances, ranked according to Mascot protein database search score. This is followed by peptide-level abundances, in tabular form, for each protein, on a protein-byprotein basis. Data are split by treatment groups according to high or low sperm competition risk. At the bottom of the report file are plots summarizing the between treatment group abundance data, at protein level. Those proteins ‘tagged’ with a red or green circle are those that were significantly changing in abundance between the treatment groups, according to ANOVA tests at p < 0.05 or p < 0.01 (respectively). Also included are the Top3 protein abundances, normalised to all proteins, in a .csv file. (ZIP 4169 kb) [file 12915_2015_197_MOESM1_ESM.zip › Additional File 1_2-way/Additional File 1_2-way analysis_Ramm et al_files/protein220_graph.png]

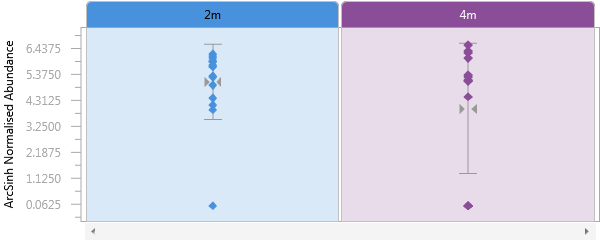

Supplement: Supplementary file 1 — A summary of the proteomics data analysis from Progenesis QI with abundances normalized using all 383 proteins. Progenesis QI html report file for the proteins identified and quantified across the four treatment groups. At the top of the file is a summary table of the protein-level average normalised abundances, ranked according to Mascot protein database search score. This is followed by peptide-level abundances, in tabular form, for each protein, on a protein-byprotein basis. Data are split by treatment groups according to high or low sperm competition risk. At the bottom of the report file are plots summarizing the between treatment group abundance data, at protein level. Those proteins ‘tagged’ with a red or green circle are those that were significantly changing in abundance between the treatment groups, according to ANOVA tests at p < 0.05 or p < 0.01 (respectively). Also included are the Top3 protein abundances, normalised to all proteins, in a .csv file. (ZIP 4169 kb) [file 12915_2015_197_MOESM1_ESM.zip › Additional File 1_2-way/Additional File 1_2-way analysis_Ramm et al_files/protein221_graph.png]

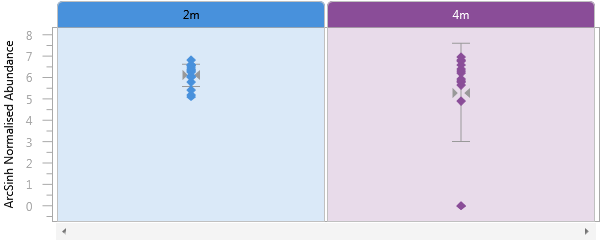

Supplement: Supplementary file 1 — A summary of the proteomics data analysis from Progenesis QI with abundances normalized using all 383 proteins. Progenesis QI html report file for the proteins identified and quantified across the four treatment groups. At the top of the file is a summary table of the protein-level average normalised abundances, ranked according to Mascot protein database search score. This is followed by peptide-level abundances, in tabular form, for each protein, on a protein-byprotein basis. Data are split by treatment groups according to high or low sperm competition risk. At the bottom of the report file are plots summarizing the between treatment group abundance data, at protein level. Those proteins ‘tagged’ with a red or green circle are those that were significantly changing in abundance between the treatment groups, according to ANOVA tests at p < 0.05 or p < 0.01 (respectively). Also included are the Top3 protein abundances, normalised to all proteins, in a .csv file. (ZIP 4169 kb) [file 12915_2015_197_MOESM1_ESM.zip › Additional File 1_2-way/Additional File 1_2-way analysis_Ramm et al_files/protein222_graph.png]

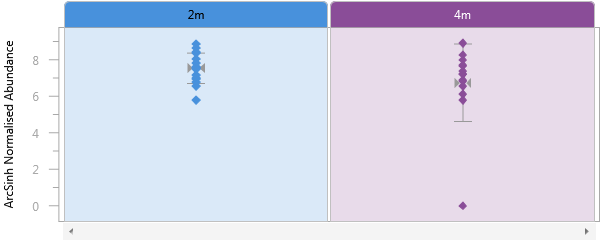

Supplement: Supplementary file 1 — A summary of the proteomics data analysis from Progenesis QI with abundances normalized using all 383 proteins. Progenesis QI html report file for the proteins identified and quantified across the four treatment groups. At the top of the file is a summary table of the protein-level average normalised abundances, ranked according to Mascot protein database search score. This is followed by peptide-level abundances, in tabular form, for each protein, on a protein-byprotein basis. Data are split by treatment groups according to high or low sperm competition risk. At the bottom of the report file are plots summarizing the between treatment group abundance data, at protein level. Those proteins ‘tagged’ with a red or green circle are those that were significantly changing in abundance between the treatment groups, according to ANOVA tests at p < 0.05 or p < 0.01 (respectively). Also included are the Top3 protein abundances, normalised to all proteins, in a .csv file. (ZIP 4169 kb) [file 12915_2015_197_MOESM1_ESM.zip › Additional File 1_2-way/Additional File 1_2-way analysis_Ramm et al_files/protein223_graph.png]

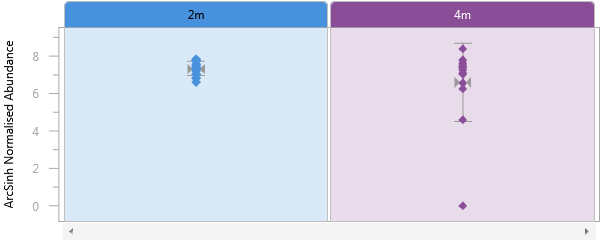

Supplement: Supplementary file 1 — A summary of the proteomics data analysis from Progenesis QI with abundances normalized using all 383 proteins. Progenesis QI html report file for the proteins identified and quantified across the four treatment groups. At the top of the file is a summary table of the protein-level average normalised abundances, ranked according to Mascot protein database search score. This is followed by peptide-level abundances, in tabular form, for each protein, on a protein-byprotein basis. Data are split by treatment groups according to high or low sperm competition risk. At the bottom of the report file are plots summarizing the between treatment group abundance data, at protein level. Those proteins ‘tagged’ with a red or green circle are those that were significantly changing in abundance between the treatment groups, according to ANOVA tests at p < 0.05 or p < 0.01 (respectively). Also included are the Top3 protein abundances, normalised to all proteins, in a .csv file. (ZIP 4169 kb) [file 12915_2015_197_MOESM1_ESM.zip › Additional File 1_2-way/Additional File 1_2-way analysis_Ramm et al_files/protein224_graph.png]

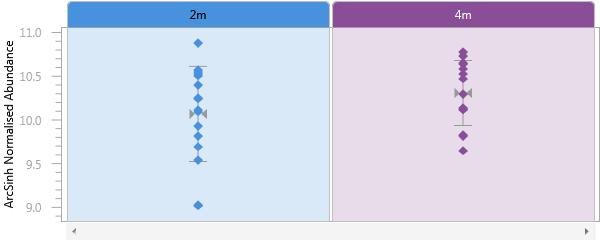

Supplement: Supplementary file 1 — A summary of the proteomics data analysis from Progenesis QI with abundances normalized using all 383 proteins. Progenesis QI html report file for the proteins identified and quantified across the four treatment groups. At the top of the file is a summary table of the protein-level average normalised abundances, ranked according to Mascot protein database search score. This is followed by peptide-level abundances, in tabular form, for each protein, on a protein-byprotein basis. Data are split by treatment groups according to high or low sperm competition risk. At the bottom of the report file are plots summarizing the between treatment group abundance data, at protein level. Those proteins ‘tagged’ with a red or green circle are those that were significantly changing in abundance between the treatment groups, according to ANOVA tests at p < 0.05 or p < 0.01 (respectively). Also included are the Top3 protein abundances, normalised to all proteins, in a .csv file. (ZIP 4169 kb) [file 12915_2015_197_MOESM1_ESM.zip › Additional File 1_2-way/Additional File 1_2-way analysis_Ramm et al_files/protein225_graph.png]

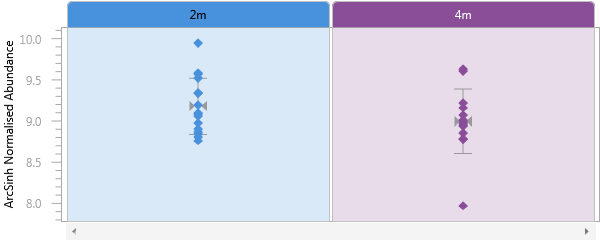

Supplement: Supplementary file 1 — A summary of the proteomics data analysis from Progenesis QI with abundances normalized using all 383 proteins. Progenesis QI html report file for the proteins identified and quantified across the four treatment groups. At the top of the file is a summary table of the protein-level average normalised abundances, ranked according to Mascot protein database search score. This is followed by peptide-level abundances, in tabular form, for each protein, on a protein-byprotein basis. Data are split by treatment groups according to high or low sperm competition risk. At the bottom of the report file are plots summarizing the between treatment group abundance data, at protein level. Those proteins ‘tagged’ with a red or green circle are those that were significantly changing in abundance between the treatment groups, according to ANOVA tests at p < 0.05 or p < 0.01 (respectively). Also included are the Top3 protein abundances, normalised to all proteins, in a .csv file. (ZIP 4169 kb) [file 12915_2015_197_MOESM1_ESM.zip › Additional File 1_2-way/Additional File 1_2-way analysis_Ramm et al_files/protein226_graph.png]

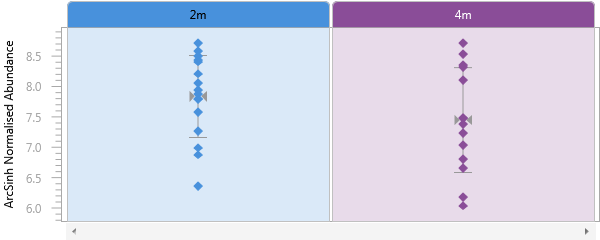

Supplement: Supplementary file 1 — A summary of the proteomics data analysis from Progenesis QI with abundances normalized using all 383 proteins. Progenesis QI html report file for the proteins identified and quantified across the four treatment groups. At the top of the file is a summary table of the protein-level average normalised abundances, ranked according to Mascot protein database search score. This is followed by peptide-level abundances, in tabular form, for each protein, on a protein-byprotein basis. Data are split by treatment groups according to high or low sperm competition risk. At the bottom of the report file are plots summarizing the between treatment group abundance data, at protein level. Those proteins ‘tagged’ with a red or green circle are those that were significantly changing in abundance between the treatment groups, according to ANOVA tests at p < 0.05 or p < 0.01 (respectively). Also included are the Top3 protein abundances, normalised to all proteins, in a .csv file. (ZIP 4169 kb) [file 12915_2015_197_MOESM1_ESM.zip › Additional File 1_2-way/Additional File 1_2-way analysis_Ramm et al_files/protein227_graph.png]

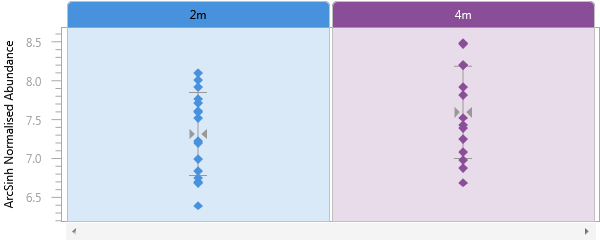

Supplement: Supplementary file 1 — A summary of the proteomics data analysis from Progenesis QI with abundances normalized using all 383 proteins. Progenesis QI html report file for the proteins identified and quantified across the four treatment groups. At the top of the file is a summary table of the protein-level average normalised abundances, ranked according to Mascot protein database search score. This is followed by peptide-level abundances, in tabular form, for each protein, on a protein-byprotein basis. Data are split by treatment groups according to high or low sperm competition risk. At the bottom of the report file are plots summarizing the between treatment group abundance data, at protein level. Those proteins ‘tagged’ with a red or green circle are those that were significantly changing in abundance between the treatment groups, according to ANOVA tests at p < 0.05 or p < 0.01 (respectively). Also included are the Top3 protein abundances, normalised to all proteins, in a .csv file. (ZIP 4169 kb) [file 12915_2015_197_MOESM1_ESM.zip › Additional File 1_2-way/Additional File 1_2-way analysis_Ramm et al_files/protein228_graph.png]

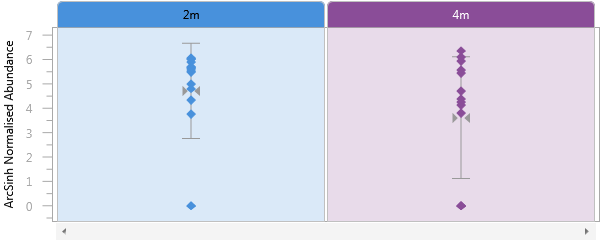

Supplement: Supplementary file 1 — A summary of the proteomics data analysis from Progenesis QI with abundances normalized using all 383 proteins. Progenesis QI html report file for the proteins identified and quantified across the four treatment groups. At the top of the file is a summary table of the protein-level average normalised abundances, ranked according to Mascot protein database search score. This is followed by peptide-level abundances, in tabular form, for each protein, on a protein-byprotein basis. Data are split by treatment groups according to high or low sperm competition risk. At the bottom of the report file are plots summarizing the between treatment group abundance data, at protein level. Those proteins ‘tagged’ with a red or green circle are those that were significantly changing in abundance between the treatment groups, according to ANOVA tests at p < 0.05 or p < 0.01 (respectively). Also included are the Top3 protein abundances, normalised to all proteins, in a .csv file. (ZIP 4169 kb) [file 12915_2015_197_MOESM1_ESM.zip › Additional File 1_2-way/Additional File 1_2-way analysis_Ramm et al_files/protein229_graph.png]

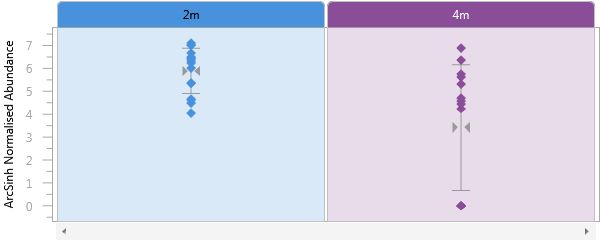

Supplement: Supplementary file 1 — A summary of the proteomics data analysis from Progenesis QI with abundances normalized using all 383 proteins. Progenesis QI html report file for the proteins identified and quantified across the four treatment groups. At the top of the file is a summary table of the protein-level average normalised abundances, ranked according to Mascot protein database search score. This is followed by peptide-level abundances, in tabular form, for each protein, on a protein-byprotein basis. Data are split by treatment groups according to high or low sperm competition risk. At the bottom of the report file are plots summarizing the between treatment group abundance data, at protein level. Those proteins ‘tagged’ with a red or green circle are those that were significantly changing in abundance between the treatment groups, according to ANOVA tests at p < 0.05 or p < 0.01 (respectively). Also included are the Top3 protein abundances, normalised to all proteins, in a .csv file. (ZIP 4169 kb) [file 12915_2015_197_MOESM1_ESM.zip › Additional File 1_2-way/Additional File 1_2-way analysis_Ramm et al_files/protein22_graph.png]

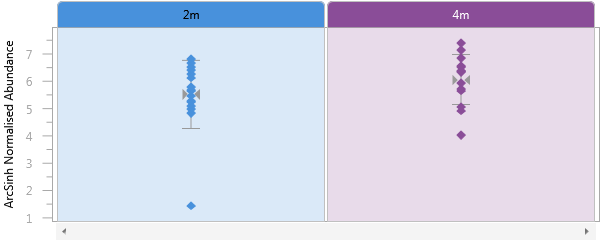

Supplement: Supplementary file 1 — A summary of the proteomics data analysis from Progenesis QI with abundances normalized using all 383 proteins. Progenesis QI html report file for the proteins identified and quantified across the four treatment groups. At the top of the file is a summary table of the protein-level average normalised abundances, ranked according to Mascot protein database search score. This is followed by peptide-level abundances, in tabular form, for each protein, on a protein-byprotein basis. Data are split by treatment groups according to high or low sperm competition risk. At the bottom of the report file are plots summarizing the between treatment group abundance data, at protein level. Those proteins ‘tagged’ with a red or green circle are those that were significantly changing in abundance between the treatment groups, according to ANOVA tests at p < 0.05 or p < 0.01 (respectively). Also included are the Top3 protein abundances, normalised to all proteins, in a .csv file. (ZIP 4169 kb) [file 12915_2015_197_MOESM1_ESM.zip › Additional File 1_2-way/Additional File 1_2-way analysis_Ramm et al_files/protein230_graph.png]

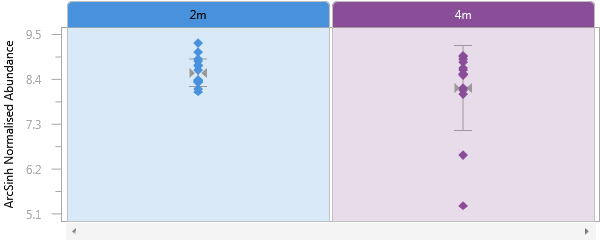

Supplement: Supplementary file 1 — A summary of the proteomics data analysis from Progenesis QI with abundances normalized using all 383 proteins. Progenesis QI html report file for the proteins identified and quantified across the four treatment groups. At the top of the file is a summary table of the protein-level average normalised abundances, ranked according to Mascot protein database search score. This is followed by peptide-level abundances, in tabular form, for each protein, on a protein-byprotein basis. Data are split by treatment groups according to high or low sperm competition risk. At the bottom of the report file are plots summarizing the between treatment group abundance data, at protein level. Those proteins ‘tagged’ with a red or green circle are those that were significantly changing in abundance between the treatment groups, according to ANOVA tests at p < 0.05 or p < 0.01 (respectively). Also included are the Top3 protein abundances, normalised to all proteins, in a .csv file. (ZIP 4169 kb) [file 12915_2015_197_MOESM1_ESM.zip › Additional File 1_2-way/Additional File 1_2-way analysis_Ramm et al_files/protein231_graph.png]

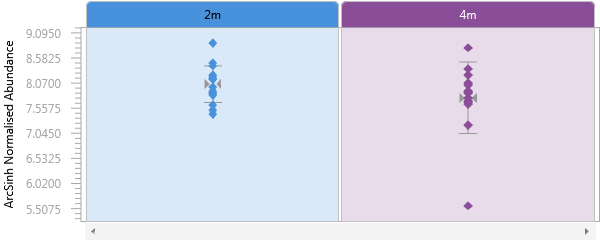

Supplement: Supplementary file 1 — A summary of the proteomics data analysis from Progenesis QI with abundances normalized using all 383 proteins. Progenesis QI html report file for the proteins identified and quantified across the four treatment groups. At the top of the file is a summary table of the protein-level average normalised abundances, ranked according to Mascot protein database search score. This is followed by peptide-level abundances, in tabular form, for each protein, on a protein-byprotein basis. Data are split by treatment groups according to high or low sperm competition risk. At the bottom of the report file are plots summarizing the between treatment group abundance data, at protein level. Those proteins ‘tagged’ with a red or green circle are those that were significantly changing in abundance between the treatment groups, according to ANOVA tests at p < 0.05 or p < 0.01 (respectively). Also included are the Top3 protein abundances, normalised to all proteins, in a .csv file. (ZIP 4169 kb) [file 12915_2015_197_MOESM1_ESM.zip › Additional File 1_2-way/Additional File 1_2-way analysis_Ramm et al_files/protein232_graph.png]

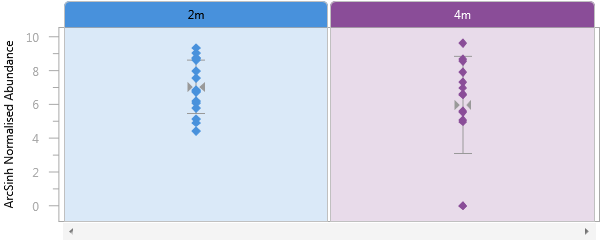

Supplement: Supplementary file 1 — A summary of the proteomics data analysis from Progenesis QI with abundances normalized using all 383 proteins. Progenesis QI html report file for the proteins identified and quantified across the four treatment groups. At the top of the file is a summary table of the protein-level average normalised abundances, ranked according to Mascot protein database search score. This is followed by peptide-level abundances, in tabular form, for each protein, on a protein-byprotein basis. Data are split by treatment groups according to high or low sperm competition risk. At the bottom of the report file are plots summarizing the between treatment group abundance data, at protein level. Those proteins ‘tagged’ with a red or green circle are those that were significantly changing in abundance between the treatment groups, according to ANOVA tests at p < 0.05 or p < 0.01 (respectively). Also included are the Top3 protein abundances, normalised to all proteins, in a .csv file. (ZIP 4169 kb) [file 12915_2015_197_MOESM1_ESM.zip › Additional File 1_2-way/Additional File 1_2-way analysis_Ramm et al_files/protein233_graph.png]

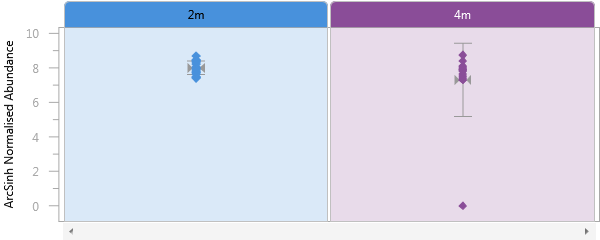

Supplement: Supplementary file 1 — A summary of the proteomics data analysis from Progenesis QI with abundances normalized using all 383 proteins. Progenesis QI html report file for the proteins identified and quantified across the four treatment groups. At the top of the file is a summary table of the protein-level average normalised abundances, ranked according to Mascot protein database search score. This is followed by peptide-level abundances, in tabular form, for each protein, on a protein-byprotein basis. Data are split by treatment groups according to high or low sperm competition risk. At the bottom of the report file are plots summarizing the between treatment group abundance data, at protein level. Those proteins ‘tagged’ with a red or green circle are those that were significantly changing in abundance between the treatment groups, according to ANOVA tests at p < 0.05 or p < 0.01 (respectively). Also included are the Top3 protein abundances, normalised to all proteins, in a .csv file. (ZIP 4169 kb) [file 12915_2015_197_MOESM1_ESM.zip › Additional File 1_2-way/Additional File 1_2-way analysis_Ramm et al_files/protein234_graph.png]

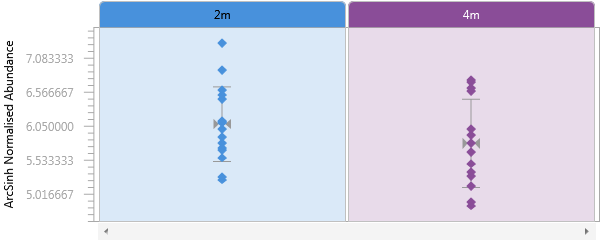

Supplement: Supplementary file 1 — A summary of the proteomics data analysis from Progenesis QI with abundances normalized using all 383 proteins. Progenesis QI html report file for the proteins identified and quantified across the four treatment groups. At the top of the file is a summary table of the protein-level average normalised abundances, ranked according to Mascot protein database search score. This is followed by peptide-level abundances, in tabular form, for each protein, on a protein-byprotein basis. Data are split by treatment groups according to high or low sperm competition risk. At the bottom of the report file are plots summarizing the between treatment group abundance data, at protein level. Those proteins ‘tagged’ with a red or green circle are those that were significantly changing in abundance between the treatment groups, according to ANOVA tests at p < 0.05 or p < 0.01 (respectively). Also included are the Top3 protein abundances, normalised to all proteins, in a .csv file. (ZIP 4169 kb) [file 12915_2015_197_MOESM1_ESM.zip › Additional File 1_2-way/Additional File 1_2-way analysis_Ramm et al_files/protein235_graph.png]

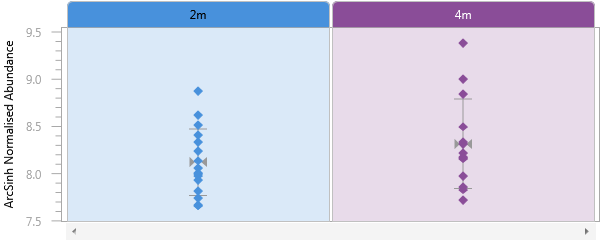

Supplement: Supplementary file 1 — A summary of the proteomics data analysis from Progenesis QI with abundances normalized using all 383 proteins. Progenesis QI html report file for the proteins identified and quantified across the four treatment groups. At the top of the file is a summary table of the protein-level average normalised abundances, ranked according to Mascot protein database search score. This is followed by peptide-level abundances, in tabular form, for each protein, on a protein-byprotein basis. Data are split by treatment groups according to high or low sperm competition risk. At the bottom of the report file are plots summarizing the between treatment group abundance data, at protein level. Those proteins ‘tagged’ with a red or green circle are those that were significantly changing in abundance between the treatment groups, according to ANOVA tests at p < 0.05 or p < 0.01 (respectively). Also included are the Top3 protein abundances, normalised to all proteins, in a .csv file. (ZIP 4169 kb) [file 12915_2015_197_MOESM1_ESM.zip › Additional File 1_2-way/Additional File 1_2-way analysis_Ramm et al_files/protein236_graph.png]

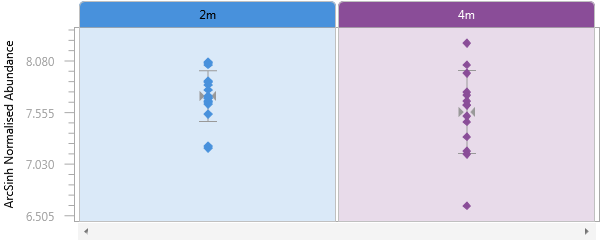

Supplement: Supplementary file 1 — A summary of the proteomics data analysis from Progenesis QI with abundances normalized using all 383 proteins. Progenesis QI html report file for the proteins identified and quantified across the four treatment groups. At the top of the file is a summary table of the protein-level average normalised abundances, ranked according to Mascot protein database search score. This is followed by peptide-level abundances, in tabular form, for each protein, on a protein-byprotein basis. Data are split by treatment groups according to high or low sperm competition risk. At the bottom of the report file are plots summarizing the between treatment group abundance data, at protein level. Those proteins ‘tagged’ with a red or green circle are those that were significantly changing in abundance between the treatment groups, according to ANOVA tests at p < 0.05 or p < 0.01 (respectively). Also included are the Top3 protein abundances, normalised to all proteins, in a .csv file. (ZIP 4169 kb) [file 12915_2015_197_MOESM1_ESM.zip › Additional File 1_2-way/Additional File 1_2-way analysis_Ramm et al_files/protein237_graph.png]

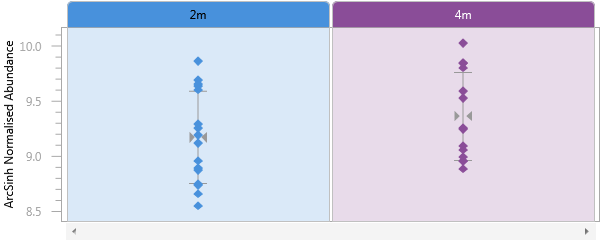

Supplement: Supplementary file 1 — A summary of the proteomics data analysis from Progenesis QI with abundances normalized using all 383 proteins. Progenesis QI html report file for the proteins identified and quantified across the four treatment groups. At the top of the file is a summary table of the protein-level average normalised abundances, ranked according to Mascot protein database search score. This is followed by peptide-level abundances, in tabular form, for each protein, on a protein-byprotein basis. Data are split by treatment groups according to high or low sperm competition risk. At the bottom of the report file are plots summarizing the between treatment group abundance data, at protein level. Those proteins ‘tagged’ with a red or green circle are those that were significantly changing in abundance between the treatment groups, according to ANOVA tests at p < 0.05 or p < 0.01 (respectively). Also included are the Top3 protein abundances, normalised to all proteins, in a .csv file. (ZIP 4169 kb) [file 12915_2015_197_MOESM1_ESM.zip › Additional File 1_2-way/Additional File 1_2-way analysis_Ramm et al_files/protein238_graph.png]

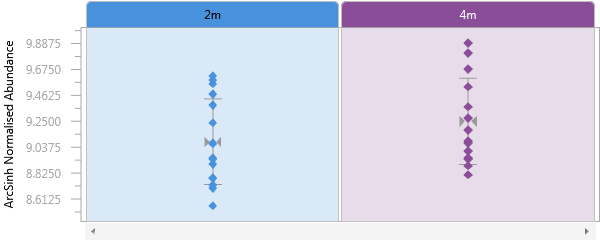

Supplement: Supplementary file 1 — A summary of the proteomics data analysis from Progenesis QI with abundances normalized using all 383 proteins. Progenesis QI html report file for the proteins identified and quantified across the four treatment groups. At the top of the file is a summary table of the protein-level average normalised abundances, ranked according to Mascot protein database search score. This is followed by peptide-level abundances, in tabular form, for each protein, on a protein-byprotein basis. Data are split by treatment groups according to high or low sperm competition risk. At the bottom of the report file are plots summarizing the between treatment group abundance data, at protein level. Those proteins ‘tagged’ with a red or green circle are those that were significantly changing in abundance between the treatment groups, according to ANOVA tests at p < 0.05 or p < 0.01 (respectively). Also included are the Top3 protein abundances, normalised to all proteins, in a .csv file. (ZIP 4169 kb) [file 12915_2015_197_MOESM1_ESM.zip › Additional File 1_2-way/Additional File 1_2-way analysis_Ramm et al_files/protein239_graph.png]

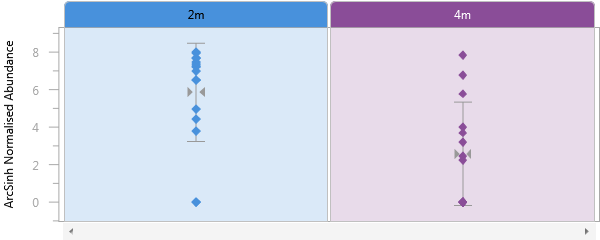

Supplement: Supplementary file 1 — A summary of the proteomics data analysis from Progenesis QI with abundances normalized using all 383 proteins. Progenesis QI html report file for the proteins identified and quantified across the four treatment groups. At the top of the file is a summary table of the protein-level average normalised abundances, ranked according to Mascot protein database search score. This is followed by peptide-level abundances, in tabular form, for each protein, on a protein-byprotein basis. Data are split by treatment groups according to high or low sperm competition risk. At the bottom of the report file are plots summarizing the between treatment group abundance data, at protein level. Those proteins ‘tagged’ with a red or green circle are those that were significantly changing in abundance between the treatment groups, according to ANOVA tests at p < 0.05 or p < 0.01 (respectively). Also included are the Top3 protein abundances, normalised to all proteins, in a .csv file. (ZIP 4169 kb) [file 12915_2015_197_MOESM1_ESM.zip › Additional File 1_2-way/Additional File 1_2-way analysis_Ramm et al_files/protein23_graph.png]

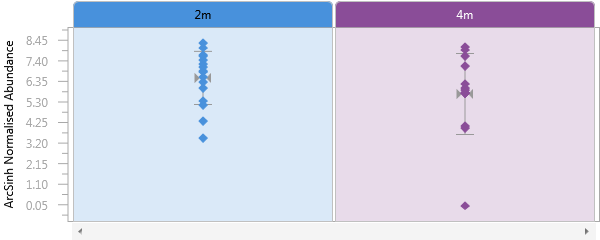

Supplement: Supplementary file 1 — A summary of the proteomics data analysis from Progenesis QI with abundances normalized using all 383 proteins. Progenesis QI html report file for the proteins identified and quantified across the four treatment groups. At the top of the file is a summary table of the protein-level average normalised abundances, ranked according to Mascot protein database search score. This is followed by peptide-level abundances, in tabular form, for each protein, on a protein-byprotein basis. Data are split by treatment groups according to high or low sperm competition risk. At the bottom of the report file are plots summarizing the between treatment group abundance data, at protein level. Those proteins ‘tagged’ with a red or green circle are those that were significantly changing in abundance between the treatment groups, according to ANOVA tests at p < 0.05 or p < 0.01 (respectively). Also included are the Top3 protein abundances, normalised to all proteins, in a .csv file. (ZIP 4169 kb) [file 12915_2015_197_MOESM1_ESM.zip › Additional File 1_2-way/Additional File 1_2-way analysis_Ramm et al_files/protein240_graph.png]

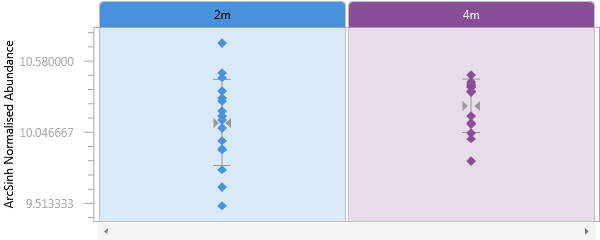

Supplement: Supplementary file 1 — A summary of the proteomics data analysis from Progenesis QI with abundances normalized using all 383 proteins. Progenesis QI html report file for the proteins identified and quantified across the four treatment groups. At the top of the file is a summary table of the protein-level average normalised abundances, ranked according to Mascot protein database search score. This is followed by peptide-level abundances, in tabular form, for each protein, on a protein-byprotein basis. Data are split by treatment groups according to high or low sperm competition risk. At the bottom of the report file are plots summarizing the between treatment group abundance data, at protein level. Those proteins ‘tagged’ with a red or green circle are those that were significantly changing in abundance between the treatment groups, according to ANOVA tests at p < 0.05 or p < 0.01 (respectively). Also included are the Top3 protein abundances, normalised to all proteins, in a .csv file. (ZIP 4169 kb) [file 12915_2015_197_MOESM1_ESM.zip › Additional File 1_2-way/Additional File 1_2-way analysis_Ramm et al_files/protein241_graph.png]

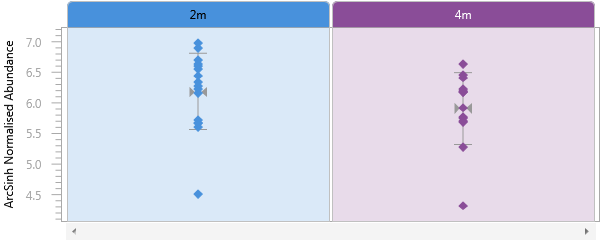

Supplement: Supplementary file 1 — A summary of the proteomics data analysis from Progenesis QI with abundances normalized using all 383 proteins. Progenesis QI html report file for the proteins identified and quantified across the four treatment groups. At the top of the file is a summary table of the protein-level average normalised abundances, ranked according to Mascot protein database search score. This is followed by peptide-level abundances, in tabular form, for each protein, on a protein-byprotein basis. Data are split by treatment groups according to high or low sperm competition risk. At the bottom of the report file are plots summarizing the between treatment group abundance data, at protein level. Those proteins ‘tagged’ with a red or green circle are those that were significantly changing in abundance between the treatment groups, according to ANOVA tests at p < 0.05 or p < 0.01 (respectively). Also included are the Top3 protein abundances, normalised to all proteins, in a .csv file. (ZIP 4169 kb) [file 12915_2015_197_MOESM1_ESM.zip › Additional File 1_2-way/Additional File 1_2-way analysis_Ramm et al_files/protein242_graph.png]

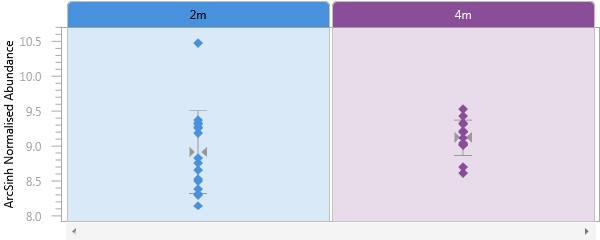

Supplement: Supplementary file 1 — A summary of the proteomics data analysis from Progenesis QI with abundances normalized using all 383 proteins. Progenesis QI html report file for the proteins identified and quantified across the four treatment groups. At the top of the file is a summary table of the protein-level average normalised abundances, ranked according to Mascot protein database search score. This is followed by peptide-level abundances, in tabular form, for each protein, on a protein-byprotein basis. Data are split by treatment groups according to high or low sperm competition risk. At the bottom of the report file are plots summarizing the between treatment group abundance data, at protein level. Those proteins ‘tagged’ with a red or green circle are those that were significantly changing in abundance between the treatment groups, according to ANOVA tests at p < 0.05 or p < 0.01 (respectively). Also included are the Top3 protein abundances, normalised to all proteins, in a .csv file. (ZIP 4169 kb) [file 12915_2015_197_MOESM1_ESM.zip › Additional File 1_2-way/Additional File 1_2-way analysis_Ramm et al_files/protein243_graph.png]

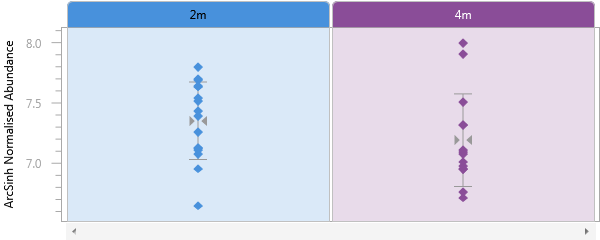

Supplement: Supplementary file 1 — A summary of the proteomics data analysis from Progenesis QI with abundances normalized using all 383 proteins. Progenesis QI html report file for the proteins identified and quantified across the four treatment groups. At the top of the file is a summary table of the protein-level average normalised abundances, ranked according to Mascot protein database search score. This is followed by peptide-level abundances, in tabular form, for each protein, on a protein-byprotein basis. Data are split by treatment groups according to high or low sperm competition risk. At the bottom of the report file are plots summarizing the between treatment group abundance data, at protein level. Those proteins ‘tagged’ with a red or green circle are those that were significantly changing in abundance between the treatment groups, according to ANOVA tests at p < 0.05 or p < 0.01 (respectively). Also included are the Top3 protein abundances, normalised to all proteins, in a .csv file. (ZIP 4169 kb) [file 12915_2015_197_MOESM1_ESM.zip › Additional File 1_2-way/Additional File 1_2-way analysis_Ramm et al_files/protein244_graph.png]

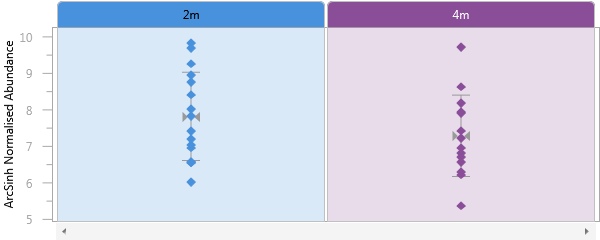

Supplement: Supplementary file 1 — A summary of the proteomics data analysis from Progenesis QI with abundances normalized using all 383 proteins. Progenesis QI html report file for the proteins identified and quantified across the four treatment groups. At the top of the file is a summary table of the protein-level average normalised abundances, ranked according to Mascot protein database search score. This is followed by peptide-level abundances, in tabular form, for each protein, on a protein-byprotein basis. Data are split by treatment groups according to high or low sperm competition risk. At the bottom of the report file are plots summarizing the between treatment group abundance data, at protein level. Those proteins ‘tagged’ with a red or green circle are those that were significantly changing in abundance between the treatment groups, according to ANOVA tests at p < 0.05 or p < 0.01 (respectively). Also included are the Top3 protein abundances, normalised to all proteins, in a .csv file. (ZIP 4169 kb) [file 12915_2015_197_MOESM1_ESM.zip › Additional File 1_2-way/Additional File 1_2-way analysis_Ramm et al_files/protein245_graph.png]

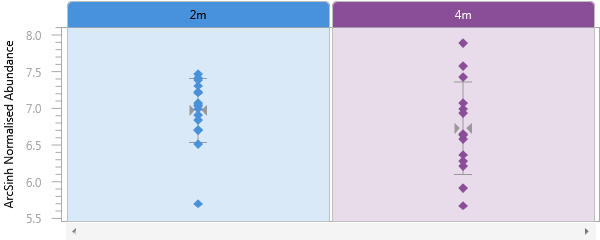

Supplement: Supplementary file 1 — A summary of the proteomics data analysis from Progenesis QI with abundances normalized using all 383 proteins. Progenesis QI html report file for the proteins identified and quantified across the four treatment groups. At the top of the file is a summary table of the protein-level average normalised abundances, ranked according to Mascot protein database search score. This is followed by peptide-level abundances, in tabular form, for each protein, on a protein-byprotein basis. Data are split by treatment groups according to high or low sperm competition risk. At the bottom of the report file are plots summarizing the between treatment group abundance data, at protein level. Those proteins ‘tagged’ with a red or green circle are those that were significantly changing in abundance between the treatment groups, according to ANOVA tests at p < 0.05 or p < 0.01 (respectively). Also included are the Top3 protein abundances, normalised to all proteins, in a .csv file. (ZIP 4169 kb) [file 12915_2015_197_MOESM1_ESM.zip › Additional File 1_2-way/Additional File 1_2-way analysis_Ramm et al_files/protein246_graph.png]

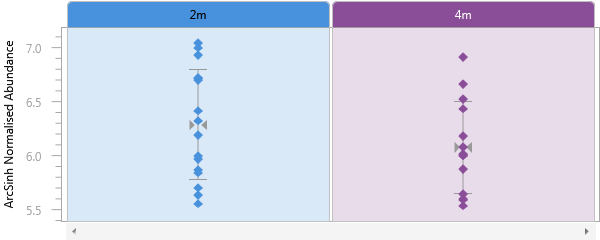

Supplement: Supplementary file 1 — A summary of the proteomics data analysis from Progenesis QI with abundances normalized using all 383 proteins. Progenesis QI html report file for the proteins identified and quantified across the four treatment groups. At the top of the file is a summary table of the protein-level average normalised abundances, ranked according to Mascot protein database search score. This is followed by peptide-level abundances, in tabular form, for each protein, on a protein-byprotein basis. Data are split by treatment groups according to high or low sperm competition risk. At the bottom of the report file are plots summarizing the between treatment group abundance data, at protein level. Those proteins ‘tagged’ with a red or green circle are those that were significantly changing in abundance between the treatment groups, according to ANOVA tests at p < 0.05 or p < 0.01 (respectively). Also included are the Top3 protein abundances, normalised to all proteins, in a .csv file. (ZIP 4169 kb) [file 12915_2015_197_MOESM1_ESM.zip › Additional File 1_2-way/Additional File 1_2-way analysis_Ramm et al_files/protein247_graph.png]

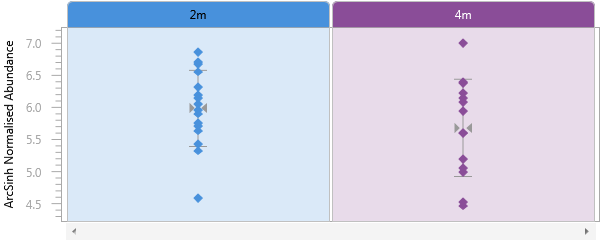

Supplement: Supplementary file 1 — A summary of the proteomics data analysis from Progenesis QI with abundances normalized using all 383 proteins. Progenesis QI html report file for the proteins identified and quantified across the four treatment groups. At the top of the file is a summary table of the protein-level average normalised abundances, ranked according to Mascot protein database search score. This is followed by peptide-level abundances, in tabular form, for each protein, on a protein-byprotein basis. Data are split by treatment groups according to high or low sperm competition risk. At the bottom of the report file are plots summarizing the between treatment group abundance data, at protein level. Those proteins ‘tagged’ with a red or green circle are those that were significantly changing in abundance between the treatment groups, according to ANOVA tests at p < 0.05 or p < 0.01 (respectively). Also included are the Top3 protein abundances, normalised to all proteins, in a .csv file. (ZIP 4169 kb) [file 12915_2015_197_MOESM1_ESM.zip › Additional File 1_2-way/Additional File 1_2-way analysis_Ramm et al_files/protein248_graph.png]

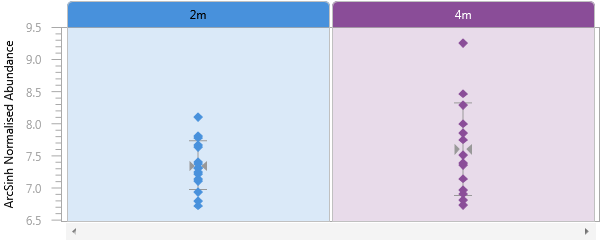

Supplement: Supplementary file 1 — A summary of the proteomics data analysis from Progenesis QI with abundances normalized using all 383 proteins. Progenesis QI html report file for the proteins identified and quantified across the four treatment groups. At the top of the file is a summary table of the protein-level average normalised abundances, ranked according to Mascot protein database search score. This is followed by peptide-level abundances, in tabular form, for each protein, on a protein-byprotein basis. Data are split by treatment groups according to high or low sperm competition risk. At the bottom of the report file are plots summarizing the between treatment group abundance data, at protein level. Those proteins ‘tagged’ with a red or green circle are those that were significantly changing in abundance between the treatment groups, according to ANOVA tests at p < 0.05 or p < 0.01 (respectively). Also included are the Top3 protein abundances, normalised to all proteins, in a .csv file. (ZIP 4169 kb) [file 12915_2015_197_MOESM1_ESM.zip › Additional File 1_2-way/Additional File 1_2-way analysis_Ramm et al_files/protein249_graph.png]

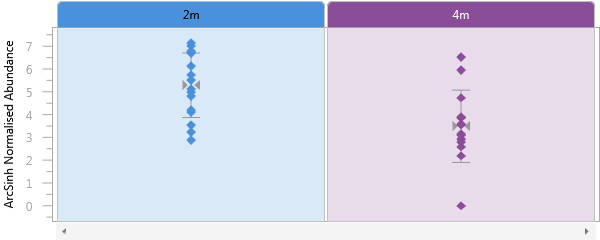

Supplement: Supplementary file 1 — A summary of the proteomics data analysis from Progenesis QI with abundances normalized using all 383 proteins. Progenesis QI html report file for the proteins identified and quantified across the four treatment groups. At the top of the file is a summary table of the protein-level average normalised abundances, ranked according to Mascot protein database search score. This is followed by peptide-level abundances, in tabular form, for each protein, on a protein-byprotein basis. Data are split by treatment groups according to high or low sperm competition risk. At the bottom of the report file are plots summarizing the between treatment group abundance data, at protein level. Those proteins ‘tagged’ with a red or green circle are those that were significantly changing in abundance between the treatment groups, according to ANOVA tests at p < 0.05 or p < 0.01 (respectively). Also included are the Top3 protein abundances, normalised to all proteins, in a .csv file. (ZIP 4169 kb) [file 12915_2015_197_MOESM1_ESM.zip › Additional File 1_2-way/Additional File 1_2-way analysis_Ramm et al_files/protein24_graph.png]

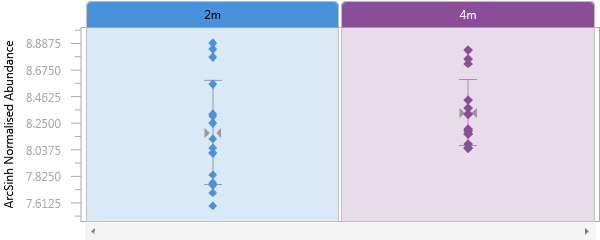

Supplement: Supplementary file 1 — A summary of the proteomics data analysis from Progenesis QI with abundances normalized using all 383 proteins. Progenesis QI html report file for the proteins identified and quantified across the four treatment groups. At the top of the file is a summary table of the protein-level average normalised abundances, ranked according to Mascot protein database search score. This is followed by peptide-level abundances, in tabular form, for each protein, on a protein-byprotein basis. Data are split by treatment groups according to high or low sperm competition risk. At the bottom of the report file are plots summarizing the between treatment group abundance data, at protein level. Those proteins ‘tagged’ with a red or green circle are those that were significantly changing in abundance between the treatment groups, according to ANOVA tests at p < 0.05 or p < 0.01 (respectively). Also included are the Top3 protein abundances, normalised to all proteins, in a .csv file. (ZIP 4169 kb) [file 12915_2015_197_MOESM1_ESM.zip › Additional File 1_2-way/Additional File 1_2-way analysis_Ramm et al_files/protein250_graph.png]

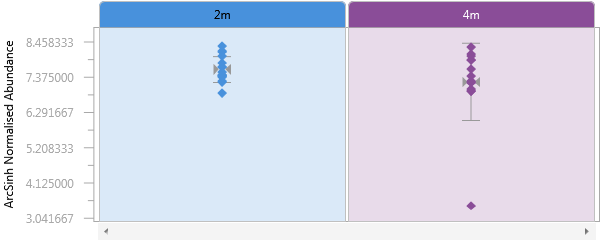

Supplement: Supplementary file 1 — A summary of the proteomics data analysis from Progenesis QI with abundances normalized using all 383 proteins. Progenesis QI html report file for the proteins identified and quantified across the four treatment groups. At the top of the file is a summary table of the protein-level average normalised abundances, ranked according to Mascot protein database search score. This is followed by peptide-level abundances, in tabular form, for each protein, on a protein-byprotein basis. Data are split by treatment groups according to high or low sperm competition risk. At the bottom of the report file are plots summarizing the between treatment group abundance data, at protein level. Those proteins ‘tagged’ with a red or green circle are those that were significantly changing in abundance between the treatment groups, according to ANOVA tests at p < 0.05 or p < 0.01 (respectively). Also included are the Top3 protein abundances, normalised to all proteins, in a .csv file. (ZIP 4169 kb) [file 12915_2015_197_MOESM1_ESM.zip › Additional File 1_2-way/Additional File 1_2-way analysis_Ramm et al_files/protein251_graph.png]

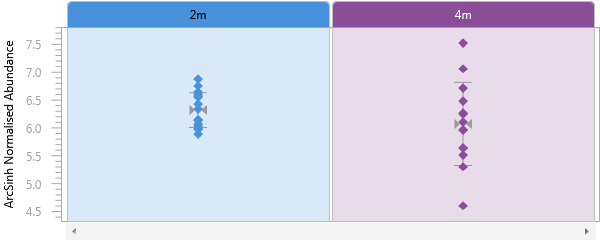

Supplement: Supplementary file 1 — A summary of the proteomics data analysis from Progenesis QI with abundances normalized using all 383 proteins. Progenesis QI html report file for the proteins identified and quantified across the four treatment groups. At the top of the file is a summary table of the protein-level average normalised abundances, ranked according to Mascot protein database search score. This is followed by peptide-level abundances, in tabular form, for each protein, on a protein-byprotein basis. Data are split by treatment groups according to high or low sperm competition risk. At the bottom of the report file are plots summarizing the between treatment group abundance data, at protein level. Those proteins ‘tagged’ with a red or green circle are those that were significantly changing in abundance between the treatment groups, according to ANOVA tests at p < 0.05 or p < 0.01 (respectively). Also included are the Top3 protein abundances, normalised to all proteins, in a .csv file. (ZIP 4169 kb) [file 12915_2015_197_MOESM1_ESM.zip › Additional File 1_2-way/Additional File 1_2-way analysis_Ramm et al_files/protein252_graph.png]

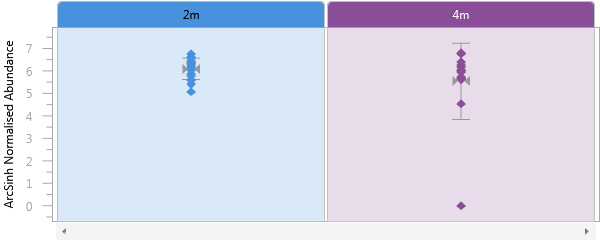

Supplement: Supplementary file 1 — A summary of the proteomics data analysis from Progenesis QI with abundances normalized using all 383 proteins. Progenesis QI html report file for the proteins identified and quantified across the four treatment groups. At the top of the file is a summary table of the protein-level average normalised abundances, ranked according to Mascot protein database search score. This is followed by peptide-level abundances, in tabular form, for each protein, on a protein-byprotein basis. Data are split by treatment groups according to high or low sperm competition risk. At the bottom of the report file are plots summarizing the between treatment group abundance data, at protein level. Those proteins ‘tagged’ with a red or green circle are those that were significantly changing in abundance between the treatment groups, according to ANOVA tests at p < 0.05 or p < 0.01 (respectively). Also included are the Top3 protein abundances, normalised to all proteins, in a .csv file. (ZIP 4169 kb) [file 12915_2015_197_MOESM1_ESM.zip › Additional File 1_2-way/Additional File 1_2-way analysis_Ramm et al_files/protein253_graph.png]

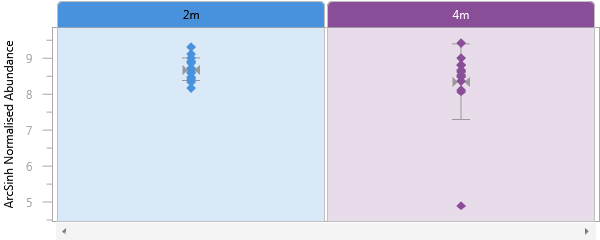

Supplement: Supplementary file 1 — A summary of the proteomics data analysis from Progenesis QI with abundances normalized using all 383 proteins. Progenesis QI html report file for the proteins identified and quantified across the four treatment groups. At the top of the file is a summary table of the protein-level average normalised abundances, ranked according to Mascot protein database search score. This is followed by peptide-level abundances, in tabular form, for each protein, on a protein-byprotein basis. Data are split by treatment groups according to high or low sperm competition risk. At the bottom of the report file are plots summarizing the between treatment group abundance data, at protein level. Those proteins ‘tagged’ with a red or green circle are those that were significantly changing in abundance between the treatment groups, according to ANOVA tests at p < 0.05 or p < 0.01 (respectively). Also included are the Top3 protein abundances, normalised to all proteins, in a .csv file. (ZIP 4169 kb) [file 12915_2015_197_MOESM1_ESM.zip › Additional File 1_2-way/Additional File 1_2-way analysis_Ramm et al_files/protein254_graph.png]

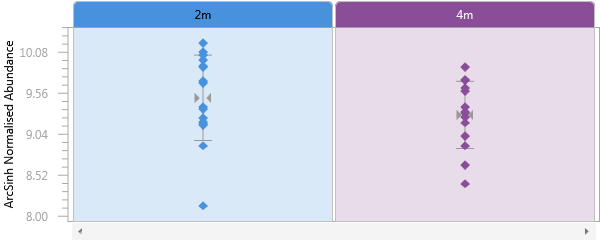

Supplement: Supplementary file 1 — A summary of the proteomics data analysis from Progenesis QI with abundances normalized using all 383 proteins. Progenesis QI html report file for the proteins identified and quantified across the four treatment groups. At the top of the file is a summary table of the protein-level average normalised abundances, ranked according to Mascot protein database search score. This is followed by peptide-level abundances, in tabular form, for each protein, on a protein-byprotein basis. Data are split by treatment groups according to high or low sperm competition risk. At the bottom of the report file are plots summarizing the between treatment group abundance data, at protein level. Those proteins ‘tagged’ with a red or green circle are those that were significantly changing in abundance between the treatment groups, according to ANOVA tests at p < 0.05 or p < 0.01 (respectively). Also included are the Top3 protein abundances, normalised to all proteins, in a .csv file. (ZIP 4169 kb) [file 12915_2015_197_MOESM1_ESM.zip › Additional File 1_2-way/Additional File 1_2-way analysis_Ramm et al_files/protein255_graph.png]

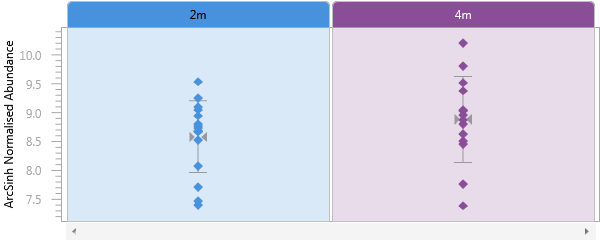

Supplement: Supplementary file 1 — A summary of the proteomics data analysis from Progenesis QI with abundances normalized using all 383 proteins. Progenesis QI html report file for the proteins identified and quantified across the four treatment groups. At the top of the file is a summary table of the protein-level average normalised abundances, ranked according to Mascot protein database search score. This is followed by peptide-level abundances, in tabular form, for each protein, on a protein-byprotein basis. Data are split by treatment groups according to high or low sperm competition risk. At the bottom of the report file are plots summarizing the between treatment group abundance data, at protein level. Those proteins ‘tagged’ with a red or green circle are those that were significantly changing in abundance between the treatment groups, according to ANOVA tests at p < 0.05 or p < 0.01 (respectively). Also included are the Top3 protein abundances, normalised to all proteins, in a .csv file. (ZIP 4169 kb) [file 12915_2015_197_MOESM1_ESM.zip › Additional File 1_2-way/Additional File 1_2-way analysis_Ramm et al_files/protein256_graph.png]

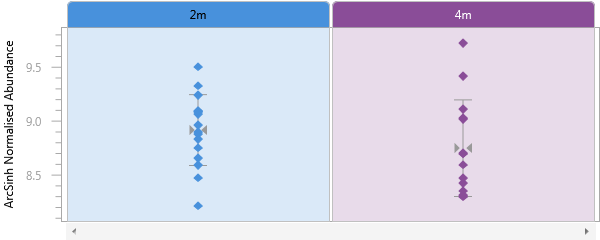

Supplement: Supplementary file 1 — A summary of the proteomics data analysis from Progenesis QI with abundances normalized using all 383 proteins. Progenesis QI html report file for the proteins identified and quantified across the four treatment groups. At the top of the file is a summary table of the protein-level average normalised abundances, ranked according to Mascot protein database search score. This is followed by peptide-level abundances, in tabular form, for each protein, on a protein-byprotein basis. Data are split by treatment groups according to high or low sperm competition risk. At the bottom of the report file are plots summarizing the between treatment group abundance data, at protein level. Those proteins ‘tagged’ with a red or green circle are those that were significantly changing in abundance between the treatment groups, according to ANOVA tests at p < 0.05 or p < 0.01 (respectively). Also included are the Top3 protein abundances, normalised to all proteins, in a .csv file. (ZIP 4169 kb) [file 12915_2015_197_MOESM1_ESM.zip › Additional File 1_2-way/Additional File 1_2-way analysis_Ramm et al_files/protein257_graph.png]

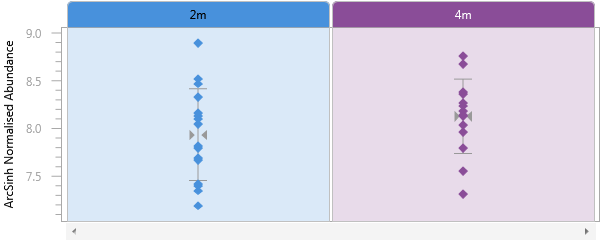

Supplement: Supplementary file 1 — A summary of the proteomics data analysis from Progenesis QI with abundances normalized using all 383 proteins. Progenesis QI html report file for the proteins identified and quantified across the four treatment groups. At the top of the file is a summary table of the protein-level average normalised abundances, ranked according to Mascot protein database search score. This is followed by peptide-level abundances, in tabular form, for each protein, on a protein-byprotein basis. Data are split by treatment groups according to high or low sperm competition risk. At the bottom of the report file are plots summarizing the between treatment group abundance data, at protein level. Those proteins ‘tagged’ with a red or green circle are those that were significantly changing in abundance between the treatment groups, according to ANOVA tests at p < 0.05 or p < 0.01 (respectively). Also included are the Top3 protein abundances, normalised to all proteins, in a .csv file. (ZIP 4169 kb) [file 12915_2015_197_MOESM1_ESM.zip › Additional File 1_2-way/Additional File 1_2-way analysis_Ramm et al_files/protein258_graph.png]

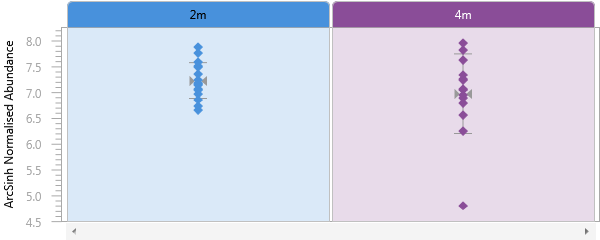

Supplement: Supplementary file 1 — A summary of the proteomics data analysis from Progenesis QI with abundances normalized using all 383 proteins. Progenesis QI html report file for the proteins identified and quantified across the four treatment groups. At the top of the file is a summary table of the protein-level average normalised abundances, ranked according to Mascot protein database search score. This is followed by peptide-level abundances, in tabular form, for each protein, on a protein-byprotein basis. Data are split by treatment groups according to high or low sperm competition risk. At the bottom of the report file are plots summarizing the between treatment group abundance data, at protein level. Those proteins ‘tagged’ with a red or green circle are those that were significantly changing in abundance between the treatment groups, according to ANOVA tests at p < 0.05 or p < 0.01 (respectively). Also included are the Top3 protein abundances, normalised to all proteins, in a .csv file. (ZIP 4169 kb) [file 12915_2015_197_MOESM1_ESM.zip › Additional File 1_2-way/Additional File 1_2-way analysis_Ramm et al_files/protein259_graph.png]

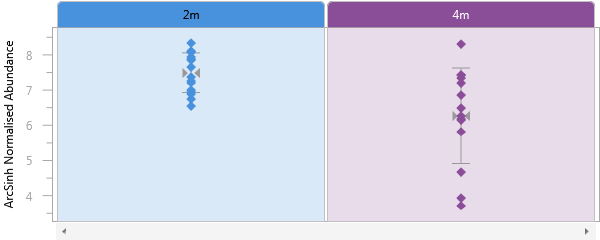

Supplement: Supplementary file 1 — A summary of the proteomics data analysis from Progenesis QI with abundances normalized using all 383 proteins. Progenesis QI html report file for the proteins identified and quantified across the four treatment groups. At the top of the file is a summary table of the protein-level average normalised abundances, ranked according to Mascot protein database search score. This is followed by peptide-level abundances, in tabular form, for each protein, on a protein-byprotein basis. Data are split by treatment groups according to high or low sperm competition risk. At the bottom of the report file are plots summarizing the between treatment group abundance data, at protein level. Those proteins ‘tagged’ with a red or green circle are those that were significantly changing in abundance between the treatment groups, according to ANOVA tests at p < 0.05 or p < 0.01 (respectively). Also included are the Top3 protein abundances, normalised to all proteins, in a .csv file. (ZIP 4169 kb) [file 12915_2015_197_MOESM1_ESM.zip › Additional File 1_2-way/Additional File 1_2-way analysis_Ramm et al_files/protein25_graph.png]

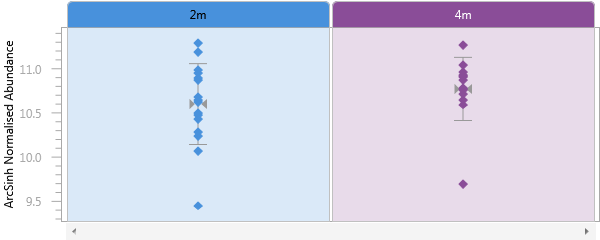

Supplement: Supplementary file 1 — A summary of the proteomics data analysis from Progenesis QI with abundances normalized using all 383 proteins. Progenesis QI html report file for the proteins identified and quantified across the four treatment groups. At the top of the file is a summary table of the protein-level average normalised abundances, ranked according to Mascot protein database search score. This is followed by peptide-level abundances, in tabular form, for each protein, on a protein-byprotein basis. Data are split by treatment groups according to high or low sperm competition risk. At the bottom of the report file are plots summarizing the between treatment group abundance data, at protein level. Those proteins ‘tagged’ with a red or green circle are those that were significantly changing in abundance between the treatment groups, according to ANOVA tests at p < 0.05 or p < 0.01 (respectively). Also included are the Top3 protein abundances, normalised to all proteins, in a .csv file. (ZIP 4169 kb) [file 12915_2015_197_MOESM1_ESM.zip › Additional File 1_2-way/Additional File 1_2-way analysis_Ramm et al_files/protein260_graph.png]

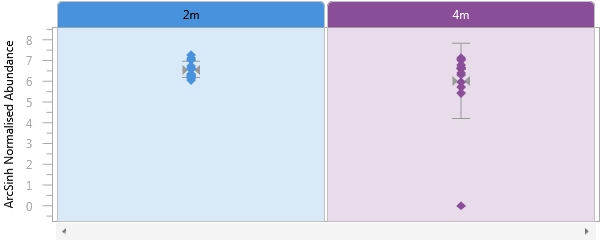

Supplement: Supplementary file 1 — A summary of the proteomics data analysis from Progenesis QI with abundances normalized using all 383 proteins. Progenesis QI html report file for the proteins identified and quantified across the four treatment groups. At the top of the file is a summary table of the protein-level average normalised abundances, ranked according to Mascot protein database search score. This is followed by peptide-level abundances, in tabular form, for each protein, on a protein-byprotein basis. Data are split by treatment groups according to high or low sperm competition risk. At the bottom of the report file are plots summarizing the between treatment group abundance data, at protein level. Those proteins ‘tagged’ with a red or green circle are those that were significantly changing in abundance between the treatment groups, according to ANOVA tests at p < 0.05 or p < 0.01 (respectively). Also included are the Top3 protein abundances, normalised to all proteins, in a .csv file. (ZIP 4169 kb) [file 12915_2015_197_MOESM1_ESM.zip › Additional File 1_2-way/Additional File 1_2-way analysis_Ramm et al_files/protein261_graph.png]

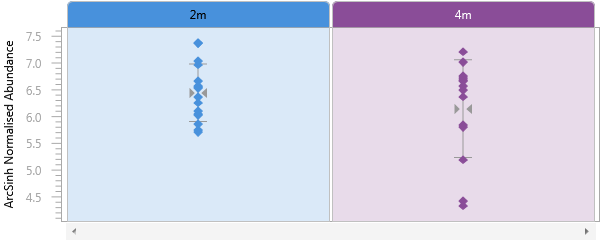

Supplement: Supplementary file 1 — A summary of the proteomics data analysis from Progenesis QI with abundances normalized using all 383 proteins. Progenesis QI html report file for the proteins identified and quantified across the four treatment groups. At the top of the file is a summary table of the protein-level average normalised abundances, ranked according to Mascot protein database search score. This is followed by peptide-level abundances, in tabular form, for each protein, on a protein-byprotein basis. Data are split by treatment groups according to high or low sperm competition risk. At the bottom of the report file are plots summarizing the between treatment group abundance data, at protein level. Those proteins ‘tagged’ with a red or green circle are those that were significantly changing in abundance between the treatment groups, according to ANOVA tests at p < 0.05 or p < 0.01 (respectively). Also included are the Top3 protein abundances, normalised to all proteins, in a .csv file. (ZIP 4169 kb) [file 12915_2015_197_MOESM1_ESM.zip › Additional File 1_2-way/Additional File 1_2-way analysis_Ramm et al_files/protein262_graph.png]

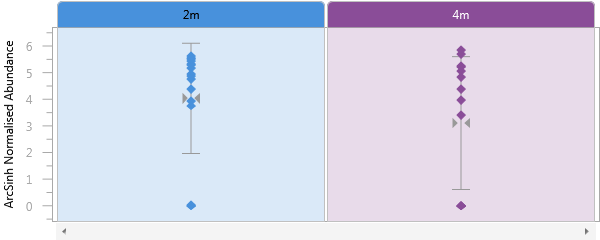

Supplement: Supplementary file 1 — A summary of the proteomics data analysis from Progenesis QI with abundances normalized using all 383 proteins. Progenesis QI html report file for the proteins identified and quantified across the four treatment groups. At the top of the file is a summary table of the protein-level average normalised abundances, ranked according to Mascot protein database search score. This is followed by peptide-level abundances, in tabular form, for each protein, on a protein-byprotein basis. Data are split by treatment groups according to high or low sperm competition risk. At the bottom of the report file are plots summarizing the between treatment group abundance data, at protein level. Those proteins ‘tagged’ with a red or green circle are those that were significantly changing in abundance between the treatment groups, according to ANOVA tests at p < 0.05 or p < 0.01 (respectively). Also included are the Top3 protein abundances, normalised to all proteins, in a .csv file. (ZIP 4169 kb) [file 12915_2015_197_MOESM1_ESM.zip › Additional File 1_2-way/Additional File 1_2-way analysis_Ramm et al_files/protein263_graph.png]

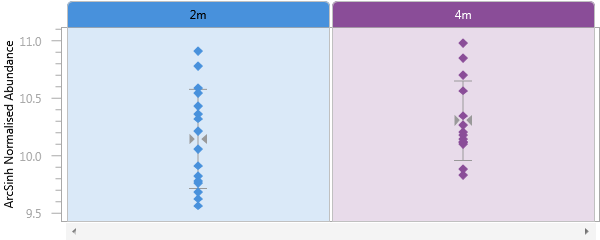

Supplement: Supplementary file 1 — A summary of the proteomics data analysis from Progenesis QI with abundances normalized using all 383 proteins. Progenesis QI html report file for the proteins identified and quantified across the four treatment groups. At the top of the file is a summary table of the protein-level average normalised abundances, ranked according to Mascot protein database search score. This is followed by peptide-level abundances, in tabular form, for each protein, on a protein-byprotein basis. Data are split by treatment groups according to high or low sperm competition risk. At the bottom of the report file are plots summarizing the between treatment group abundance data, at protein level. Those proteins ‘tagged’ with a red or green circle are those that were significantly changing in abundance between the treatment groups, according to ANOVA tests at p < 0.05 or p < 0.01 (respectively). Also included are the Top3 protein abundances, normalised to all proteins, in a .csv file. (ZIP 4169 kb) [file 12915_2015_197_MOESM1_ESM.zip › Additional File 1_2-way/Additional File 1_2-way analysis_Ramm et al_files/protein264_graph.png]

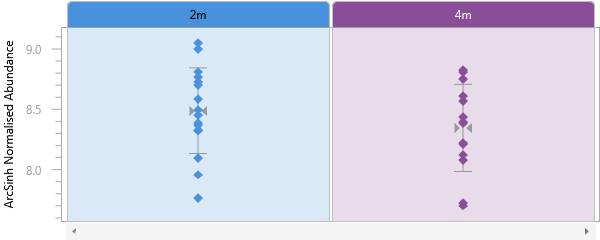

Supplement: Supplementary file 1 — A summary of the proteomics data analysis from Progenesis QI with abundances normalized using all 383 proteins. Progenesis QI html report file for the proteins identified and quantified across the four treatment groups. At the top of the file is a summary table of the protein-level average normalised abundances, ranked according to Mascot protein database search score. This is followed by peptide-level abundances, in tabular form, for each protein, on a protein-byprotein basis. Data are split by treatment groups according to high or low sperm competition risk. At the bottom of the report file are plots summarizing the between treatment group abundance data, at protein level. Those proteins ‘tagged’ with a red or green circle are those that were significantly changing in abundance between the treatment groups, according to ANOVA tests at p < 0.05 or p < 0.01 (respectively). Also included are the Top3 protein abundances, normalised to all proteins, in a .csv file. (ZIP 4169 kb) [file 12915_2015_197_MOESM1_ESM.zip › Additional File 1_2-way/Additional File 1_2-way analysis_Ramm et al_files/protein265_graph.png]

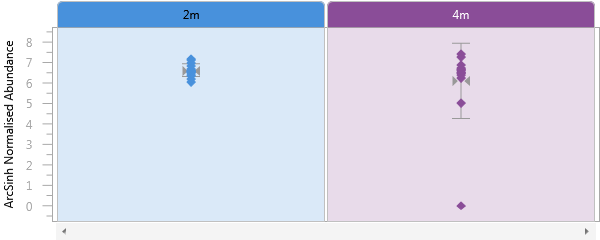

Supplement: Supplementary file 1 — A summary of the proteomics data analysis from Progenesis QI with abundances normalized using all 383 proteins. Progenesis QI html report file for the proteins identified and quantified across the four treatment groups. At the top of the file is a summary table of the protein-level average normalised abundances, ranked according to Mascot protein database search score. This is followed by peptide-level abundances, in tabular form, for each protein, on a protein-byprotein basis. Data are split by treatment groups according to high or low sperm competition risk. At the bottom of the report file are plots summarizing the between treatment group abundance data, at protein level. Those proteins ‘tagged’ with a red or green circle are those that were significantly changing in abundance between the treatment groups, according to ANOVA tests at p < 0.05 or p < 0.01 (respectively). Also included are the Top3 protein abundances, normalised to all proteins, in a .csv file. (ZIP 4169 kb) [file 12915_2015_197_MOESM1_ESM.zip › Additional File 1_2-way/Additional File 1_2-way analysis_Ramm et al_files/protein266_graph.png]

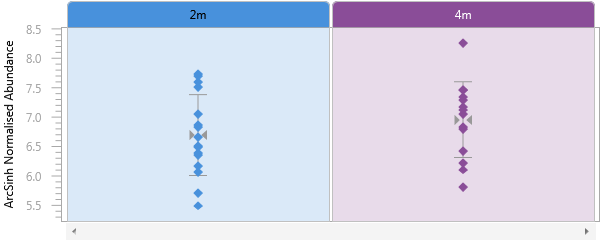

Supplement: Supplementary file 1 — A summary of the proteomics data analysis from Progenesis QI with abundances normalized using all 383 proteins. Progenesis QI html report file for the proteins identified and quantified across the four treatment groups. At the top of the file is a summary table of the protein-level average normalised abundances, ranked according to Mascot protein database search score. This is followed by peptide-level abundances, in tabular form, for each protein, on a protein-byprotein basis. Data are split by treatment groups according to high or low sperm competition risk. At the bottom of the report file are plots summarizing the between treatment group abundance data, at protein level. Those proteins ‘tagged’ with a red or green circle are those that were significantly changing in abundance between the treatment groups, according to ANOVA tests at p < 0.05 or p < 0.01 (respectively). Also included are the Top3 protein abundances, normalised to all proteins, in a .csv file. (ZIP 4169 kb) [file 12915_2015_197_MOESM1_ESM.zip › Additional File 1_2-way/Additional File 1_2-way analysis_Ramm et al_files/protein267_graph.png]

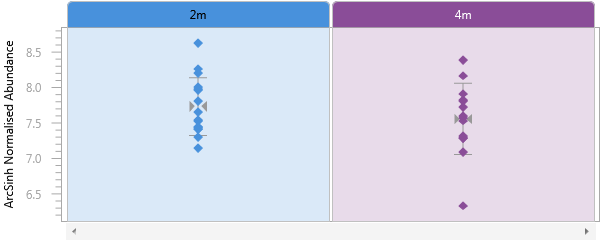

Supplement: Supplementary file 1 — A summary of the proteomics data analysis from Progenesis QI with abundances normalized using all 383 proteins. Progenesis QI html report file for the proteins identified and quantified across the four treatment groups. At the top of the file is a summary table of the protein-level average normalised abundances, ranked according to Mascot protein database search score. This is followed by peptide-level abundances, in tabular form, for each protein, on a protein-byprotein basis. Data are split by treatment groups according to high or low sperm competition risk. At the bottom of the report file are plots summarizing the between treatment group abundance data, at protein level. Those proteins ‘tagged’ with a red or green circle are those that were significantly changing in abundance between the treatment groups, according to ANOVA tests at p < 0.05 or p < 0.01 (respectively). Also included are the Top3 protein abundances, normalised to all proteins, in a .csv file. (ZIP 4169 kb) [file 12915_2015_197_MOESM1_ESM.zip › Additional File 1_2-way/Additional File 1_2-way analysis_Ramm et al_files/protein268_graph.png]

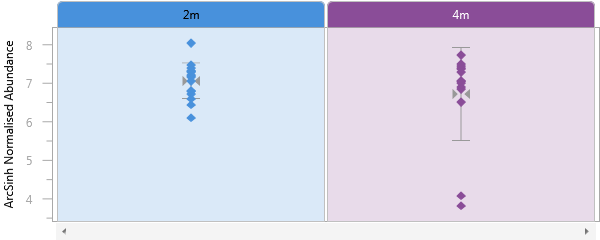

Supplement: Supplementary file 1 — A summary of the proteomics data analysis from Progenesis QI with abundances normalized using all 383 proteins. Progenesis QI html report file for the proteins identified and quantified across the four treatment groups. At the top of the file is a summary table of the protein-level average normalised abundances, ranked according to Mascot protein database search score. This is followed by peptide-level abundances, in tabular form, for each protein, on a protein-byprotein basis. Data are split by treatment groups according to high or low sperm competition risk. At the bottom of the report file are plots summarizing the between treatment group abundance data, at protein level. Those proteins ‘tagged’ with a red or green circle are those that were significantly changing in abundance between the treatment groups, according to ANOVA tests at p < 0.05 or p < 0.01 (respectively). Also included are the Top3 protein abundances, normalised to all proteins, in a .csv file. (ZIP 4169 kb) [file 12915_2015_197_MOESM1_ESM.zip › Additional File 1_2-way/Additional File 1_2-way analysis_Ramm et al_files/protein269_graph.png]

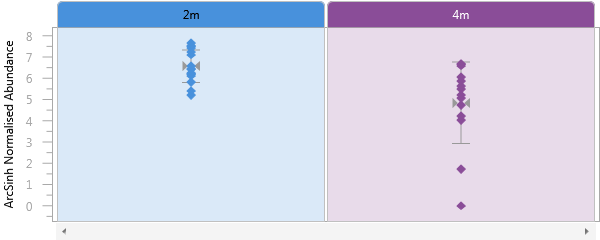

Supplement: Supplementary file 1 — A summary of the proteomics data analysis from Progenesis QI with abundances normalized using all 383 proteins. Progenesis QI html report file for the proteins identified and quantified across the four treatment groups. At the top of the file is a summary table of the protein-level average normalised abundances, ranked according to Mascot protein database search score. This is followed by peptide-level abundances, in tabular form, for each protein, on a protein-byprotein basis. Data are split by treatment groups according to high or low sperm competition risk. At the bottom of the report file are plots summarizing the between treatment group abundance data, at protein level. Those proteins ‘tagged’ with a red or green circle are those that were significantly changing in abundance between the treatment groups, according to ANOVA tests at p < 0.05 or p < 0.01 (respectively). Also included are the Top3 protein abundances, normalised to all proteins, in a .csv file. (ZIP 4169 kb) [file 12915_2015_197_MOESM1_ESM.zip › Additional File 1_2-way/Additional File 1_2-way analysis_Ramm et al_files/protein26_graph.png]

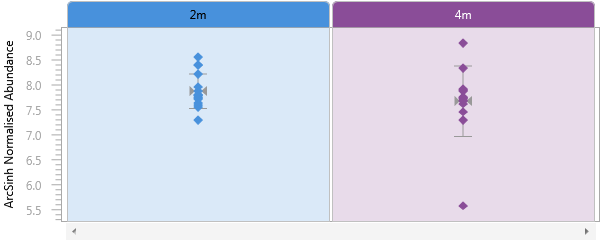

Supplement: Supplementary file 1 — A summary of the proteomics data analysis from Progenesis QI with abundances normalized using all 383 proteins. Progenesis QI html report file for the proteins identified and quantified across the four treatment groups. At the top of the file is a summary table of the protein-level average normalised abundances, ranked according to Mascot protein database search score. This is followed by peptide-level abundances, in tabular form, for each protein, on a protein-byprotein basis. Data are split by treatment groups according to high or low sperm competition risk. At the bottom of the report file are plots summarizing the between treatment group abundance data, at protein level. Those proteins ‘tagged’ with a red or green circle are those that were significantly changing in abundance between the treatment groups, according to ANOVA tests at p < 0.05 or p < 0.01 (respectively). Also included are the Top3 protein abundances, normalised to all proteins, in a .csv file. (ZIP 4169 kb) [file 12915_2015_197_MOESM1_ESM.zip › Additional File 1_2-way/Additional File 1_2-way analysis_Ramm et al_files/protein270_graph.png]

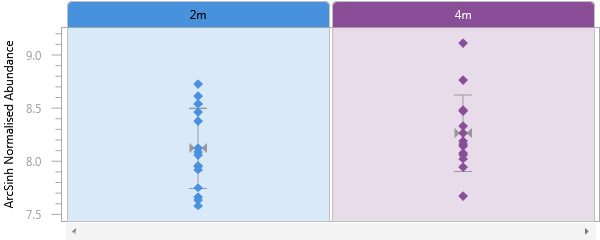

Supplement: Supplementary file 1 — A summary of the proteomics data analysis from Progenesis QI with abundances normalized using all 383 proteins. Progenesis QI html report file for the proteins identified and quantified across the four treatment groups. At the top of the file is a summary table of the protein-level average normalised abundances, ranked according to Mascot protein database search score. This is followed by peptide-level abundances, in tabular form, for each protein, on a protein-byprotein basis. Data are split by treatment groups according to high or low sperm competition risk. At the bottom of the report file are plots summarizing the between treatment group abundance data, at protein level. Those proteins ‘tagged’ with a red or green circle are those that were significantly changing in abundance between the treatment groups, according to ANOVA tests at p < 0.05 or p < 0.01 (respectively). Also included are the Top3 protein abundances, normalised to all proteins, in a .csv file. (ZIP 4169 kb) [file 12915_2015_197_MOESM1_ESM.zip › Additional File 1_2-way/Additional File 1_2-way analysis_Ramm et al_files/protein271_graph.png]

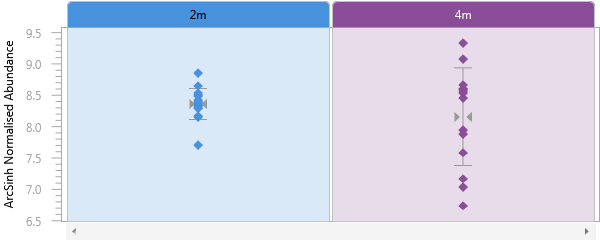

Supplement: Supplementary file 1 — A summary of the proteomics data analysis from Progenesis QI with abundances normalized using all 383 proteins. Progenesis QI html report file for the proteins identified and quantified across the four treatment groups. At the top of the file is a summary table of the protein-level average normalised abundances, ranked according to Mascot protein database search score. This is followed by peptide-level abundances, in tabular form, for each protein, on a protein-byprotein basis. Data are split by treatment groups according to high or low sperm competition risk. At the bottom of the report file are plots summarizing the between treatment group abundance data, at protein level. Those proteins ‘tagged’ with a red or green circle are those that were significantly changing in abundance between the treatment groups, according to ANOVA tests at p < 0.05 or p < 0.01 (respectively). Also included are the Top3 protein abundances, normalised to all proteins, in a .csv file. (ZIP 4169 kb) [file 12915_2015_197_MOESM1_ESM.zip › Additional File 1_2-way/Additional File 1_2-way analysis_Ramm et al_files/protein272_graph.png]

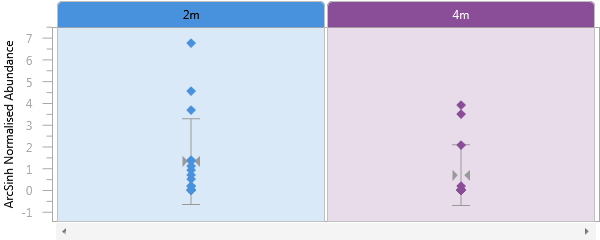

Supplement: Supplementary file 1 — A summary of the proteomics data analysis from Progenesis QI with abundances normalized using all 383 proteins. Progenesis QI html report file for the proteins identified and quantified across the four treatment groups. At the top of the file is a summary table of the protein-level average normalised abundances, ranked according to Mascot protein database search score. This is followed by peptide-level abundances, in tabular form, for each protein, on a protein-byprotein basis. Data are split by treatment groups according to high or low sperm competition risk. At the bottom of the report file are plots summarizing the between treatment group abundance data, at protein level. Those proteins ‘tagged’ with a red or green circle are those that were significantly changing in abundance between the treatment groups, according to ANOVA tests at p < 0.05 or p < 0.01 (respectively). Also included are the Top3 protein abundances, normalised to all proteins, in a .csv file. (ZIP 4169 kb) [file 12915_2015_197_MOESM1_ESM.zip › Additional File 1_2-way/Additional File 1_2-way analysis_Ramm et al_files/protein273_graph.png]

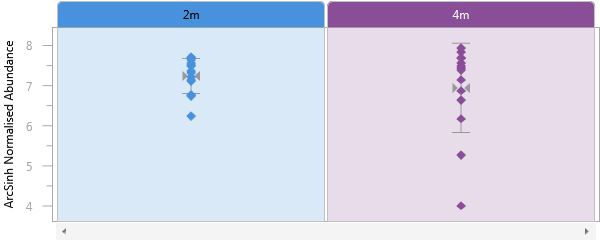

Supplement: Supplementary file 1 — A summary of the proteomics data analysis from Progenesis QI with abundances normalized using all 383 proteins. Progenesis QI html report file for the proteins identified and quantified across the four treatment groups. At the top of the file is a summary table of the protein-level average normalised abundances, ranked according to Mascot protein database search score. This is followed by peptide-level abundances, in tabular form, for each protein, on a protein-byprotein basis. Data are split by treatment groups according to high or low sperm competition risk. At the bottom of the report file are plots summarizing the between treatment group abundance data, at protein level. Those proteins ‘tagged’ with a red or green circle are those that were significantly changing in abundance between the treatment groups, according to ANOVA tests at p < 0.05 or p < 0.01 (respectively). Also included are the Top3 protein abundances, normalised to all proteins, in a .csv file. (ZIP 4169 kb) [file 12915_2015_197_MOESM1_ESM.zip › Additional File 1_2-way/Additional File 1_2-way analysis_Ramm et al_files/protein274_graph.png]

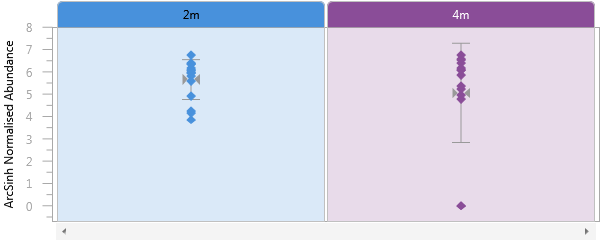

Supplement: Supplementary file 1 — A summary of the proteomics data analysis from Progenesis QI with abundances normalized using all 383 proteins. Progenesis QI html report file for the proteins identified and quantified across the four treatment groups. At the top of the file is a summary table of the protein-level average normalised abundances, ranked according to Mascot protein database search score. This is followed by peptide-level abundances, in tabular form, for each protein, on a protein-byprotein basis. Data are split by treatment groups according to high or low sperm competition risk. At the bottom of the report file are plots summarizing the between treatment group abundance data, at protein level. Those proteins ‘tagged’ with a red or green circle are those that were significantly changing in abundance between the treatment groups, according to ANOVA tests at p < 0.05 or p < 0.01 (respectively). Also included are the Top3 protein abundances, normalised to all proteins, in a .csv file. (ZIP 4169 kb) [file 12915_2015_197_MOESM1_ESM.zip › Additional File 1_2-way/Additional File 1_2-way analysis_Ramm et al_files/protein275_graph.png]

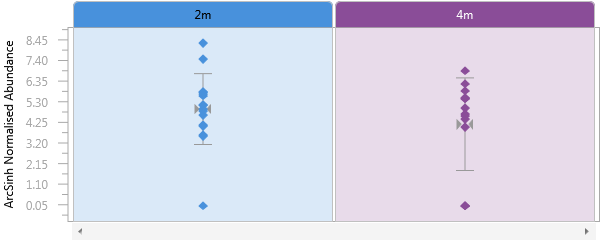

Supplement: Supplementary file 1 — A summary of the proteomics data analysis from Progenesis QI with abundances normalized using all 383 proteins. Progenesis QI html report file for the proteins identified and quantified across the four treatment groups. At the top of the file is a summary table of the protein-level average normalised abundances, ranked according to Mascot protein database search score. This is followed by peptide-level abundances, in tabular form, for each protein, on a protein-byprotein basis. Data are split by treatment groups according to high or low sperm competition risk. At the bottom of the report file are plots summarizing the between treatment group abundance data, at protein level. Those proteins ‘tagged’ with a red or green circle are those that were significantly changing in abundance between the treatment groups, according to ANOVA tests at p < 0.05 or p < 0.01 (respectively). Also included are the Top3 protein abundances, normalised to all proteins, in a .csv file. (ZIP 4169 kb) [file 12915_2015_197_MOESM1_ESM.zip › Additional File 1_2-way/Additional File 1_2-way analysis_Ramm et al_files/protein276_graph.png]

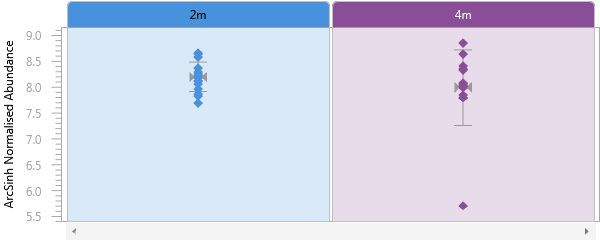

Supplement: Supplementary file 1 — A summary of the proteomics data analysis from Progenesis QI with abundances normalized using all 383 proteins. Progenesis QI html report file for the proteins identified and quantified across the four treatment groups. At the top of the file is a summary table of the protein-level average normalised abundances, ranked according to Mascot protein database search score. This is followed by peptide-level abundances, in tabular form, for each protein, on a protein-byprotein basis. Data are split by treatment groups according to high or low sperm competition risk. At the bottom of the report file are plots summarizing the between treatment group abundance data, at protein level. Those proteins ‘tagged’ with a red or green circle are those that were significantly changing in abundance between the treatment groups, according to ANOVA tests at p < 0.05 or p < 0.01 (respectively). Also included are the Top3 protein abundances, normalised to all proteins, in a .csv file. (ZIP 4169 kb) [file 12915_2015_197_MOESM1_ESM.zip › Additional File 1_2-way/Additional File 1_2-way analysis_Ramm et al_files/protein277_graph.png]

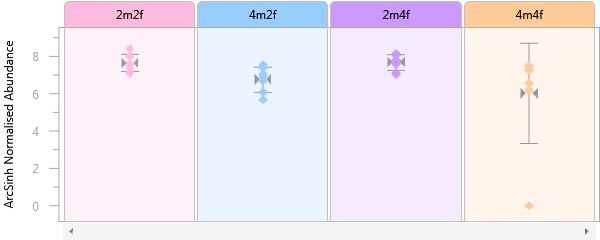

Supplement: Supplementary file 2 — A summary of the proteomics data analysis from Progenesis QI with abundances normalised using only 31 ejaculated proteins. Progenesis QI html report file for the proteins identified and quantified across the four treatment groups. At the top of the file is a summary table of the protein-level average normalised abundances, ranked according to Mascot protein database search score. This is followed by peptide-level abundances, in tabular form, for each protein, on a protein-by-protein basis. Data are split by treatment groups according to high or low sperm competition risk, and high or low potential mating rate. At the bottom of the report file are plots summarizing the between treatment group abundance data, at protein level. Those proteins ‘tagged’ with a red or green circle are those that were significantly changing in abundance between the treatment groups, according to ANOVA tests at p < 0.05 or p < 0.01 (respectively). Also included are the Top3 protein abundances, normalised to the 31 ejaculated proteins, in a .csv file. (ZIP 4957 kb) [file 12915_2015_197_MOESM2_ESM.zip › Additional File 2_4-way/Additional File 2_4-way analysis_Ramm et al_files/protein100_graph.png]
