# Supplementary material for: Sperm competition risk drives plasticity in seminal fluid composition
Source: BMC Biol. 2015 Oct 27;13:87. doi: 10.1186/s12915-015-0197-2 (PMC4624372; doi:10.1186/s12915-015-0197-2)
Supplement: Supplementary file 2 — A summary of the proteomics data analysis from Progenesis QI with abundances normalised using only 31 ejaculated proteins. Progenesis QI html report file for the proteins identified and quantified across the four treatment groups. At the top of the file is a summary table of the protein-level average normalised abundances, ranked according to Mascot protein database search score. This is followed by peptide-level abundances, in tabular form, for each protein, on a protein-by-protein basis. Data are split by treatment groups according to high or low sperm competition risk, and high or low potential mating rate. At the bottom of the report file are plots summarizing the between treatment group abundance data, at protein level. Those proteins ‘tagged’ with a red or green circle are those that were significantly changing in abundance between the treatment groups, according to ANOVA tests at p < 0.05 or p < 0.01 (respectively). Also included are the Top3 protein abundances, normalised to the 31 ejaculated proteins, in a .csv file. (ZIP 4957 kb) [file 12915_2015_197_MOESM2_ESM.zip › Additional File 2_4-way/Additional File 2_4-way analysis_Ramm et al.htm]

Ramm1ul\_v4


# Ramm1ul\_v4

  

## Experiment: Ramm1ul\_v4

## Report created: 03/02/2015 14:20:15

## Proteins

### Protein building options

|  |  |
| --- | --- |
| Protein grouping | **Group similar proteins** |
| Protein quantitation | **Relative Quantitation using Hi-3** |

  

| Accession | Peptides | Score | Anova (p)\* | Fold | Tags | Description | Average Normalised Abundances | | | |
| --- | --- | --- | --- | --- | --- | --- | --- | --- | --- | --- |
|  |  |  |  |  |  |  | 2m2f | 4m2f | 2m4f | 4m4f |
| O08638 | 52 (39) | 3026.24 | 0.20 | 1.76 |  | MYH11\_MOUSE Myosin-11 OS=Mus musculus GN=Myh11 PE=1 SV=1 | 6168.17 | 3511.72 | 5974.34 | 3600.27 |
| Q6WIZ7 | 40 (40) | 2866.64 | 0.10 | 1.39 |  | Q6WIZ7\_MOUSE PSv-2 OS=Mus musculus GN=Svs1 PE=2 SV=1 | 9.45e+005 | 1.31e+006 | 1.17e+006 | 1.26e+006 |
| Q62216 | 26 (26) | 2463.65 | 0.26 | 1.38 |  | Q62216\_MOUSE Semenoclotin OS=Mus musculus GN=Semg1 PE=2 SV=1 | 9.53e+005 | 1.32e+006 | 1.07e+006 | 1.19e+006 |
| Q8BTM8 | 31 (27) | 1806.10 | 0.67 | 1.25 |  | FLNA\_MOUSE Filamin-A OS=Mus musculus GN=Flna PE=1 SV=4 | 2110.25 | 1832.35 | 2288.18 | 2210.69 |
| P07724 | 25 (25) | 1561.67 | 0.93 | 1.05 |  | ALBU\_MOUSE Serum albumin OS=Mus musculus GN=Alb PE=1 SV=3 | 2.23e+004 | 2.24e+004 | 2.22e+004 | 2.14e+004 |
| Q8VI13 | 19 (19) | 1440.86 | 0.47 | 1.41 |  | Q8VI13\_MOUSE Seminal vesicle secretion III OS Mus musculus GN Svs3a PE 2 SV 1 | 2.84e+005 | 4.01e+005 | 3.37e+005 | 3.90e+005 |
| P20029 | 21 (19) | 1438.45 | 0.81 | 1.30 |  | GRP78\_MOUSE 78 kDa glucose-regulated protein OS=Mus musculus GN=Hspa5 PE=1 SV=3 | 1.48e+004 | 1.92e+004 | 1.66e+004 | 1.63e+004 |
| P31001 | 16 (13) | 1245.35 | 0.22 | 2.98 |  | DESM\_MOUSE Desmin OS=Mus musculus GN=Des PE=1 SV=3 | 3459.58 | 1240.36 | 3696.17 | 2214.79 |
| Q8BND5 | 18 (18) | 1026.37 | 0.62 | 1.20 |  | QSOX1\_MOUSE Sulfhydryl oxidase 1 OS=Mus musculus GN=Qsox1 PE=2 SV=1 | 1.40e+004 | 1.67e+004 | 1.48e+004 | 1.50e+004 |
| P37804 | 14 (14) | 1014.01 | 0.44 | 1.51 |  | TAGL\_MOUSE Transgelin OS=Mus musculus GN=Tagln PE=1 SV=3 | 3.28e+004 | 4.97e+004 | 3.66e+004 | 3.65e+004 |
| P62737 | 15 (1) | 1001.69 | 0.15 | 5.08 |  | ACTA\_MOUSE Actin, aortic smooth muscle OS=Mus musculus GN=Acta2 PE=1 SV=1 | 3569.78 | 702.90 | 2345.91 | 2160.90 |
| P68033 | 15 (0) | 996.51 | --- | --- |  | ACTC\_MOUSE Actin, alpha cardiac muscle 1 OS=Mus musculus GN=Actc1 PE=1 SV=1 | --- | --- | --- | --- |
| P02088 | 8 (5) | 903.87 | 0.25 | 1.43 |  | HBB1\_MOUSE Hemoglobin subunit beta-1 OS=Mus musculus GN=Hbb-b1 PE=1 SV=2 | 4.20e+004 | 4.74e+004 | 5.75e+004 | 4.02e+004 |
| P18419 | 9 (9) | 864.70 | 0.15 | 1.39 |  | SVS4\_MOUSE Seminal vesicle secretory protein 4 OS=Mus musculus GN=Svs4 PE=1 SV=2 | 1.91e+006 | 2.65e+006 | 2.17e+006 | 2.34e+006 |
| P60710 | 13 (4) | 812.01 | 1.15e-004 | 2.43 |  | ACTB\_MOUSE Actin, cytoplasmic 1 OS=Mus musculus GN=Actb PE=1 SV=1 | 2.52e+004 | 1.08e+004 | 1.94e+004 | 1.03e+004 |
| P09103 | 12 (12) | 799.70 | 0.99 | 1.04 |  | PDIA1\_MOUSE Protein disulfide-isomerase OS=Mus musculus GN=P4hb PE=1 SV=1 | 8977.34 | 8958.78 | 9214.14 | 8824.68 |
| P30933 | 9 (9) | 741.65 | 4.91e-003 | 1.76 |  | SVS5\_MOUSE Seminal vesicle secretory protein 5 OS=Mus musculus GN=Svs5 PE=2 SV=1 | 7.81e+005 | 1.38e+006 | 8.79e+005 | 9.01e+005 |
| P11679 | 11 (9) | 734.01 | 0.81 | 1.17 |  | K2C8\_MOUSE Keratin, type II cytoskeletal 8 OS=Mus musculus GN=Krt8 PE=1 SV=4 | 5211.54 | 4672.82 | 5463.75 | 5003.71 |
| O08709 | 13 (13) | 724.08 | 0.03 | 1.51 |  | PRDX6\_MOUSE Peroxiredoxin-6 OS=Mus musculus GN=Prdx6 PE=1 SV=3 | 8702.13 | 5886.83 | 7304.93 | 5750.12 |
| P81117 | 13 (13) | 676.34 | 0.53 | 1.31 |  | NUCB2\_MOUSE Nucleobindin-2 OS=Mus musculus GN=Nucb2 PE=1 SV=2 | 2.22e+004 | 2.92e+004 | 2.66e+004 | 2.54e+004 |
| Q91YQ5 | 11 (11) | 673.57 | 0.91 | 1.11 |  | RPN1\_MOUSE Dolichyl-diphosphooligosaccharide--protein glycosyltransferase subunit 1 OS=Mus musculus GN=Rpn1 PE=2 SV=1 | 3463.05 | 3153.20 | 3126.02 | 3266.44 |
| P63017 | 10 (7) | 649.56 | 0.04 | 1.59 |  | HSP7C\_MOUSE Heat shock cognate 71 kDa protein OS=Mus musculus GN=Hspa8 PE=1 SV=1 | 5850.66 | 3690.85 | 5018.73 | 3854.84 |
| P58774 | 9 (4) | 646.38 | 0.18 | 1.99 |  | TPM2\_MOUSE Tropomyosin beta chain OS=Mus musculus GN=Tpm2 PE=1 SV=1 | 9373.24 | 4713.07 | 8691.97 | 7997.98 |
| Q07235 | 10 (10) | 638.19 | 0.61 | 1.33 |  | GDN\_MOUSE Glia-derived nexin OS=Mus musculus GN=Serpine2 PE=2 SV=2 | 8352.60 | 1.11e+004 | 9709.38 | 8401.10 |
| Q8VDD5 | 11 (2) | 635.84 | 0.52 | 1.41 |  | MYH9\_MOUSE Myosin-9 OS=Mus musculus GN=Myh9 PE=1 SV=4 | 399.00 | 289.56 | 284.73 | 401.50 |
| P08113 | 10 (10) | 601.67 | 0.93 | 1.10 |  | ENPL\_MOUSE Endoplasmin OS=Mus musculus GN=Hsp90b1 PE=1 SV=2 | 4244.33 | 3859.15 | 4059.19 | 4028.51 |
| P58771 | 8 (3) | 587.21 | 0.33 | 1.82 |  | TPM1\_MOUSE Tropomyosin alpha-1 chain OS=Mus musculus GN=Tpm1 PE=1 SV=1 | 1.28e+004 | 7016.10 | 1.07e+004 | 1.23e+004 |
| Q9D819 | 9 (9) | 558.06 | 0.78 | 1.68 |  | IPYR\_MOUSE Inorganic pyrophosphatase OS=Mus musculus GN=Ppa1 PE=1 SV=1 | 3695.81 | 5443.57 | 3249.27 | 4805.37 |
| Q7TPR4 | 9 (9) | 549.68 | 0.06 | 2.70 |  | ACTN1\_MOUSE Alpha-actinin-1 OS=Mus musculus GN=Actn1 PE=2 SV=1 | 2033.44 | 753.07 | 1885.81 | 1136.97 |
| Q9DBG6 | 9 (9) | 548.38 | 0.18 | 1.73 |  | RPN2\_MOUSE Dolichyl-diphosphooligosaccharide--protein glycosyltransferase subunit 2 OS=Mus musculus GN=Rpn2 PE=2 SV=1 | 4625.65 | 2666.41 | 3780.04 | 3585.43 |
| P62806 | 7 (7) | 543.30 | 0.84 | 1.18 |  | H4\_MOUSE Histone H4 OS=Mus musculus GN=Hist1h4a PE=1 SV=2 | 3.66e+004 | 3.13e+004 | 3.70e+004 | 3.56e+004 |
| P45376 | 8 (8) | 533.92 | 0.84 | 1.14 |  | ALDR\_MOUSE Aldose reductase OS=Mus musculus GN=Akr1b1 PE=1 SV=3 | 1.13e+004 | 1.11e+004 | 9986.22 | 1.12e+004 |
| P15626 | 10 (9) | 509.76 | 0.34 | 1.52 |  | GSTM2\_MOUSE Glutathione S-transferase Mu 2 OS=Mus musculus GN=Gstm2 PE=1 SV=2 | 5876.35 | 5072.37 | 3863.31 | 5324.61 |
| P10126 | 8 (8) | 499.79 | 0.98 | 1.13 |  | EF1A1\_MOUSE Elongation factor 1-alpha 1 OS=Mus musculus GN=Eef1a1 PE=1 SV=3 | 8316.77 | 7796.73 | 8035.19 | 8814.83 |
| P56480 | 9 (9) | 499.67 | 0.04 | 1.84 |  | ATPB\_MOUSE ATP synthase subunit beta, mitochondrial OS=Mus musculus GN=Atp5b PE=1 SV=2 | 4795.34 | 2715.88 | 4995.92 | 3259.00 |
| P14152 | 7 (7) | 495.15 | 0.86 | 1.22 |  | MDHC\_MOUSE Malate dehydrogenase, cytoplasmic OS=Mus musculus GN=Mdh1 PE=1 SV=3 | 7789.48 | 8560.17 | 7248.48 | 7020.28 |
| P62908 | 9 (8) | 493.17 | 0.84 | 1.10 |  | RS3\_MOUSE 40S ribosomal protein S3 OS=Mus musculus GN=Rps3 PE=1 SV=1 | 4112.61 | 3777.53 | 4030.94 | 3733.01 |
| P08249 | 10 (10) | 492.38 | 0.51 | 1.29 |  | MDHM\_MOUSE Malate dehydrogenase, mitochondrial OS=Mus musculus GN=Mdh2 PE=1 SV=3 | 4967.73 | 4292.14 | 4037.56 | 3850.21 |
| P27773 | 10 (10) | 491.38 | 0.06 | 1.55 |  | PDIA3\_MOUSE Protein disulfide-isomerase A3 OS=Mus musculus GN=Pdia3 PE=1 SV=2 | 6579.03 | 4500.31 | 5686.29 | 4238.53 |
| Q03265 | 7 (7) | 487.33 | 0.09 | 1.49 |  | ATPA\_MOUSE ATP synthase subunit alpha, mitochondrial OS=Mus musculus GN=Atp5a1 PE=1 SV=1 | 5854.82 | 4446.65 | 5940.93 | 3998.22 |
| Q04447 | 6 (6) | 472.10 | 0.03 | 1.56 |  | KCRB\_MOUSE Creatine kinase B-type OS=Mus musculus GN=Ckb PE=1 SV=1 | 5739.66 | 4990.44 | 6163.29 | 3944.73 |
| P97315 | 8 (8) | 466.61 | 0.90 | 1.09 |  | CSRP1\_MOUSE Cysteine and glycine-rich protein 1 OS=Mus musculus GN=Csrp1 PE=1 SV=3 | 1.42e+004 | 1.37e+004 | 1.50e+004 | 1.38e+004 |
| Q61879 | 8 (0) | 466.38 | --- | --- |  | MYH10\_MOUSE Myosin-10 OS=Mus musculus GN=Myh10 PE=1 SV=2 | --- | --- | --- | --- |
| Q08091 | 9 (9) | 465.86 | 0.96 | 1.12 |  | CNN1\_MOUSE Calponin-1 OS=Mus musculus GN=Cnn1 PE=2 SV=1 | 1.15e+004 | 1.08e+004 | 1.15e+004 | 1.03e+004 |
| P58252 | 6 (6) | 462.62 | 0.10 | 1.60 |  | EF2\_MOUSE Elongation factor 2 OS=Mus musculus GN=Eef2 PE=1 SV=2 | 4086.31 | 2741.70 | 3218.00 | 2553.83 |
| Q6ZWY9 | 7 (7) | 453.47 | 0.30 | 1.27 |  | H2B1C\_MOUSE Histone H2B type 1-C/E/G OS=Mus musculus GN=Hist1h2bc PE=1 SV=3 | 2.21e+004 | 1.76e+004 | 1.91e+004 | 1.74e+004 |
| Q922R8 | 6 (6) | 450.06 | 0.13 | 1.80 |  | PDIA6\_MOUSE Protein disulfide-isomerase A6 OS=Mus musculus GN=Pdia6 PE=1 SV=3 | 4935.67 | 2748.82 | 4527.12 | 3732.07 |
| P62962 | 6 (6) | 433.30 | 0.03 | 1.81 |  | PROF1\_MOUSE Profilin-1 OS=Mus musculus GN=Pfn1 PE=1 SV=2 | 9821.69 | 6301.43 | 8679.57 | 5416.50 |
| P51881 | 8 (4) | 430.60 | 0.83 | 1.19 |  | ADT2\_MOUSE ADP/ATP translocase 2 OS=Mus musculus GN=Slc25a5 PE=1 SV=3 | 2916.41 | 3351.01 | 2816.66 | 2950.96 |
| Q64356 | 5 (5) | 416.77 | 2.41e-003 | 2.38 |  | SVS6\_MOUSE Seminal vesicle secretory protein 6 OS=Mus musculus GN=Svs6 PE=2 SV=1 | 3.79e+005 | 9.01e+005 | 5.43e+005 | 6.78e+005 |
| P68372 | 8 (1) | 407.30 | 0.92 | 1.18 |  | TBB2C\_MOUSE Tubulin beta-2C chain OS=Mus musculus GN=Tubb2c PE=1 SV=1 | 2345.46 | 2356.98 | 2616.51 | 2214.15 |
| P63101 | 7 (4) | 392.92 | 1.01e-003 | 3.05 |  | 1433Z\_MOUSE 14-3-3 protein zeta/delta OS=Mus musculus GN=Ywhaz PE=1 SV=1 | 6077.69 | 1989.90 | 5122.26 | 2495.94 |
| Q05186 | 5 (5) | 387.91 | 0.88 | 1.15 |  | RCN1\_MOUSE Reticulocalbin-1 OS=Mus musculus GN=Rcn1 PE=1 SV=1 | 5656.15 | 5201.49 | 5989.24 | 5648.30 |
| P52480 | 7 (7) | 384.35 | 0.69 | 1.23 |  | KPYM\_MOUSE Pyruvate kinase isozymes M1/M2 OS=Mus musculus GN=Pkm2 PE=1 SV=4 | 2954.72 | 2596.64 | 2439.07 | 2991.25 |
| P99024 | 7 (0) | 370.72 | --- | --- |  | TBB5\_MOUSE Tubulin beta-5 chain OS=Mus musculus GN=Tubb5 PE=1 SV=1 | --- | --- | --- | --- |
| P24369 | 7 (7) | 367.93 | 0.85 | 1.22 |  | PPIB\_MOUSE Peptidyl-prolyl cis-trans isomerase B OS=Mus musculus GN=Ppib PE=2 SV=2 | 2067.51 | 2518.84 | 2329.77 | 2340.59 |
| Q64727 | 7 (7) | 364.35 | 0.02 | 1.87 |  | VINC\_MOUSE Vinculin OS=Mus musculus GN=Vcl PE=1 SV=4 | 858.48 | 500.81 | 938.25 | 594.70 |
| Q9CQ19 | 6 (6) | 363.56 | 0.28 | 2.21 |  | MYL9\_MOUSE Myosin regulatory light polypeptide 9 OS=Mus musculus GN=Myl9 PE=1 SV=3 | 2697.75 | 1221.50 | 2394.59 | 2127.13 |
| P19001 | 6 (4) | 359.08 | 0.72 | 1.31 |  | K1C19\_MOUSE Keratin, type I cytoskeletal 19 OS=Mus musculus GN=Krt19 PE=2 SV=1 | 1784.21 | 1532.29 | 1498.42 | 1961.18 |
| P68369 | 6 (1) | 356.18 | 0.28 | 1.45 |  | TBA1A\_MOUSE Tubulin alpha-1A chain OS=Mus musculus GN=Tuba1a PE=1 SV=1 | 2810.92 | 1938.93 | 2219.70 | 2730.08 |
| P05213 | 6 (0) | 355.20 | --- | --- |  | TBA1B\_MOUSE Tubulin alpha-1B chain OS=Mus musculus GN=Tuba1b PE=1 SV=2 | --- | --- | --- | --- |
| O54734 | 5 (5) | 353.04 | 0.03 | 1.72 |  | OST48\_MOUSE Dolichyl-diphosphooligosaccharide--protein glycosyltransferase 48 kDa subunit OS=Mus musculus GN=Ddost PE=1 SV=1 | 2859.92 | 1818.75 | 2609.86 | 1659.34 |
| Q60605 | 6 (6) | 352.25 | 0.22 | 2.06 |  | MYL6\_MOUSE Myosin light polypeptide 6 OS=Mus musculus GN=Myl6 PE=1 SV=3 | 5898.66 | 2864.34 | 5260.86 | 4461.69 |
| P17751 | 7 (7) | 351.57 | 0.36 | 1.31 |  | TPIS\_MOUSE Triosephosphate isomerase OS=Mus musculus GN=Tpi1 PE=1 SV=3 | 5274.28 | 4078.99 | 4590.20 | 4019.24 |
| P02089 | 4 (1) | 350.75 | 0.49 | 1.40 |  | HBB2\_MOUSE Hemoglobin subunit beta-2 OS=Mus musculus GN=Hbb-b2 PE=1 SV=2 | 1404.71 | 1960.14 | 1835.20 | 1646.26 |
| P35980 | 5 (5) | 347.96 | 0.84 | 1.15 |  | RL18\_MOUSE 60S ribosomal protein L18 OS=Mus musculus GN=Rpl18 PE=2 SV=3 | 5698.14 | 6258.54 | 6580.64 | 6224.62 |
| Q9ERD7 | 7 (0) | 334.50 | --- | --- |  | TBB3\_MOUSE Tubulin beta-3 chain OS=Mus musculus GN=Tubb3 PE=1 SV=1 | --- | --- | --- | --- |
| P16858 | 6 (6) | 331.40 | 0.61 | 1.23 |  | G3P\_MOUSE Glyceraldehyde-3-phosphate dehydrogenase OS=Mus musculus GN=Gapdh PE=1 SV=2 | 5045.38 | 5422.25 | 5915.44 | 4822.25 |
| Q9R0P5 | 5 (5) | 329.58 | 0.09 | 1.46 |  | DEST\_MOUSE Destrin OS=Mus musculus GN=Dstn PE=1 SV=3 | 9285.00 | 7091.91 | 9182.40 | 6373.63 |
| Q99KI0 | 6 (6) | 319.47 | 0.09 | 1.63 |  | ACON\_MOUSE Aconitate hydratase, mitochondrial OS=Mus musculus GN=Aco2 PE=1 SV=1 | 1443.84 | 1000.21 | 1161.94 | 888.51 |
| Q8VCT3 | 5 (4) | 311.22 | 0.60 | 1.19 |  | AMPB\_MOUSE Aminopeptidase B OS=Mus musculus GN=Rnpep PE=2 SV=1 | 2242.74 | 1891.09 | 2044.95 | 2197.69 |
| Q8CEK3 | 3 (3) | 293.84 | 0.09 | 1.91 |  | SPIKL\_MOUSE Serine protease inhibitor kazal-like protein, minor form OS=Mus musculus PE=1 SV=1 | 1.78e+005 | 3.40e+005 | 2.18e+005 | 2.80e+005 |
| Q68FD5 | 7 (7) | 283.46 | 0.02 | 3.19 |  | CLH\_MOUSE Clathrin heavy chain 1 OS=Mus musculus GN=Cltc PE=1 SV=3 | 995.01 | 312.09 | 760.63 | 503.84 |
| Q9JKR6 | 6 (6) | 282.16 | 0.08 | 2.08 |  | HYOU1\_MOUSE Hypoxia up-regulated protein 1 OS=Mus musculus GN=Hyou1 PE=1 SV=1 | 2657.07 | 1290.39 | 2689.71 | 1968.14 |
| Q99K85 | 5 (5) | 279.25 | 0.66 | 1.36 |  | SERC\_MOUSE Phosphoserine aminotransferase OS=Mus musculus GN=Psat1 PE=1 SV=1 | 1027.01 | 1114.61 | 983.57 | 818.11 |
| P61205 | 5 (1) | 275.53 | 5.75e-003 | 2.20 |  | ARF3\_MOUSE ADP-ribosylation factor 3 OS=Mus musculus GN=Arf3 PE=2 SV=2 | 3922.66 | 2529.05 | 3896.25 | 1782.17 |
| P09411 | 6 (6) | 272.97 | 0.11 | 1.78 |  | PGK1\_MOUSE Phosphoglycerate kinase 1 OS=Mus musculus GN=Pgk1 PE=1 SV=4 | 4240.70 | 2895.82 | 3798.76 | 2383.24 |
| Q61400 | 5 (5) | 271.49 | 0.08 | 1.72 |  | CEAMA\_MOUSE Carcinoembryonic antigen-related cell adhesion molecule 10 OS=Mus musculus GN=Ceacam10 PE=1 SV=1 | 8.56e+004 | 1.47e+005 | 9.52e+004 | 1.44e+005 |
| P20152 | 5 (2) | 270.98 | 0.09 | 2.17 |  | VIME\_MOUSE Vimentin OS=Mus musculus GN=Vim PE=1 SV=3 | 509.14 | 234.32 | 430.03 | 435.50 |
| P17742 | 5 (5) | 270.04 | 0.80 | 1.29 |  | PPIA\_MOUSE Peptidyl-prolyl cis-trans isomerase A OS=Mus musculus GN=Ppia PE=1 SV=2 | 6095.48 | 7469.85 | 5803.03 | 7001.67 |
| Q8BZH1 | 5 (5) | 268.54 | 0.39 | 2.81 |  | TGM4\_MOUSE Protein-glutamine gamma-glutamyltransferase 4 OS=Mus musculus GN=Tgm4 PE=1 SV=2 | 487.41 | 210.38 | 173.61 | 446.86 |
| P62983 | 4 (4) | 268.19 | 0.73 | 1.26 |  | RS27A\_MOUSE Ubiquitin-40S ribosomal protein S27a OS=Mus musculus GN=Rps27a PE=1 SV=2 | 1.65e+004 | 1.73e+004 | 1.67e+004 | 1.37e+004 |
| Q8BFZ3 | 5 (0) | 261.49 | --- | --- |  | ACTBL\_MOUSE Beta-actin-like protein 2 OS=Mus musculus GN=Actbl2 PE=1 SV=1 | --- | --- | --- | --- |
| P62259 | 5 (3) | 261.23 | 0.05 | 2.17 |  | 1433E\_MOUSE 14-3-3 protein epsilon OS=Mus musculus GN=Ywhae PE=1 SV=1 | 6831.49 | 3142.42 | 6124.03 | 4511.86 |
| P68254 | 5 (1) | 261.01 | 0.23 | 1.58 |  | 1433T\_MOUSE 14-3-3 protein theta OS=Mus musculus GN=Ywhaq PE=1 SV=1 | 631.21 | 406.92 | 494.94 | 641.74 |
| P14211 | 4 (4) | 260.63 | 0.82 | 1.26 |  | CALR\_MOUSE Calreticulin OS=Mus musculus GN=Calr PE=1 SV=1 | 5641.03 | 6257.39 | 4999.40 | 6303.71 |
| P68368 | 5 (1) | 259.44 | 0.04 | 2.84 |  | TBA4A\_MOUSE Tubulin alpha-4A chain OS=Mus musculus GN=Tuba4a PE=1 SV=1 | 1653.43 | 583.10 | 1539.66 | 726.00 |
| Q6ZWN5 | 6 (6) | 259.41 | 0.97 | 1.11 |  | RS9\_MOUSE 40S ribosomal protein S9 OS=Mus musculus GN=Rps9 PE=2 SV=3 | 1120.53 | 1089.60 | 1067.51 | 1185.28 |
| P84084 | 5 (1) | 256.37 | 5.32e-004 | 2.41 |  | ARF5\_MOUSE ADP-ribosylation factor 5 OS=Mus musculus GN=Arf5 PE=2 SV=2 | 1397.02 | 644.18 | 1330.09 | 578.48 |
| P62242 | 4 (4) | 255.46 | 0.47 | 1.24 |  | RS8\_MOUSE 40S ribosomal protein S8 OS=Mus musculus GN=Rps8 PE=1 SV=2 | 2222.17 | 2009.61 | 2092.02 | 1787.38 |
| P09036 | 4 (4) | 252.63 | 0.71 | 1.16 |  | ISK3\_MOUSE Serine protease inhibitor Kazal-type 3 OS=Mus musculus GN=Spink3 PE=1 SV=1 | 1.31e+005 | 1.42e+005 | 1.22e+005 | 1.25e+005 |
| P17182 | 4 (4) | 252.50 | 0.05 | 1.76 |  | ENOA\_MOUSE Alpha-enolase OS=Mus musculus GN=Eno1 PE=1 SV=3 | 2749.44 | 2447.51 | 2717.40 | 1562.87 |
| Q8BG05 | 4 (4) | 245.91 | 0.93 | 1.31 |  | ROA3\_MOUSE Heterogeneous nuclear ribonucleoprotein A3 OS=Mus musculus GN=Hnrnpa3 PE=1 SV=1 | 1527.62 | 1479.49 | 1628.77 | 1940.69 |
| P99027 | 4 (4) | 243.32 | 0.11 | 1.46 |  | RLA2\_MOUSE 60S acidic ribosomal protein P2 OS=Mus musculus GN=Rplp2 PE=1 SV=3 | 5959.11 | 4091.17 | 5860.10 | 4860.28 |
| P51410 | 5 (5) | 233.26 | 0.02 | 2.09 |  | RL9\_MOUSE 60S ribosomal protein L9 OS=Mus musculus GN=Rpl9 PE=2 SV=2 | 3220.98 | 1779.41 | 2672.13 | 1544.72 |
| P14206 | 5 (5) | 232.45 | 0.18 | 1.48 |  | RSSA\_MOUSE 40S ribosomal protein SA OS=Mus musculus GN=Rpsa PE=1 SV=4 | 3237.28 | 2215.48 | 3287.75 | 2934.39 |
| P25444 | 5 (5) | 225.01 | 0.01 | 1.66 |  | RS2\_MOUSE 40S ribosomal protein S2 OS=Mus musculus GN=Rps2 PE=1 SV=3 | 3727.06 | 2296.95 | 3817.26 | 2787.27 |
| P62754 | 3 (3) | 221.78 | 0.39 | 1.31 |  | RS6\_MOUSE 40S ribosomal protein S6 OS=Mus musculus GN=Rps6 PE=1 SV=1 | 1737.98 | 1365.14 | 1784.63 | 1410.89 |
| P68040 | 5 (5) | 220.39 | 0.92 | 1.16 |  | GBLP\_MOUSE Guanine nucleotide-binding protein subunit beta-2-like 1 OS=Mus musculus GN=Gnb2l1 PE=1 SV=3 | 2074.74 | 2279.96 | 2038.92 | 2356.58 |
| P62204 | 4 (4) | 215.67 | 0.16 | 1.29 |  | CALM\_MOUSE Calmodulin OS=Mus musculus GN=Calm1 PE=1 SV=2 | 2894.26 | 2248.76 | 2449.41 | 2280.04 |
| P05064 | 4 (3) | 211.97 | 0.73 | 1.32 |  | ALDOA\_MOUSE Fructose-bisphosphate aldolase A OS=Mus musculus GN=Aldoa PE=1 SV=2 | 2103.45 | 1925.90 | 1588.64 | 1777.02 |
| P47911 | 4 (3) | 207.96 | 0.35 | 1.49 |  | RL6\_MOUSE 60S ribosomal protein L6 OS=Mus musculus GN=Rpl6 PE=1 SV=3 | 2268.07 | 1717.82 | 2076.55 | 1522.83 |
| P09405 | 4 (4) | 207.86 | 0.75 | 1.64 |  | NUCL\_MOUSE Nucleolin OS=Mus musculus GN=Ncl PE=1 SV=2 | 895.53 | 1466.92 | 998.81 | 1327.63 |
| P08228 | 4 (4) | 206.87 | 0.89 | 1.21 |  | SODC\_MOUSE Superoxide dismutase [Cu-Zn] OS=Mus musculus GN=Sod1 PE=1 SV=2 | 4326.99 | 5152.16 | 4258.84 | 4495.88 |
| P61982 | 4 (1) | 204.20 | 0.25 | 1.51 |  | 1433G\_MOUSE 14-3-3 protein gamma OS=Mus musculus GN=Ywhag PE=1 SV=2 | 699.38 | 464.36 | 588.74 | 524.86 |
| P12032 | 4 (4) | 203.53 | 0.47 | 2.34 |  | TIMP1\_MOUSE Metalloproteinase inhibitor 1 OS=Mus musculus GN=Timp1 PE=2 SV=2 | 1770.36 | 1364.17 | 1389.84 | 754.98 |
| P35564 | 3 (3) | 199.87 | 0.09 | 1.57 |  | CALX\_MOUSE Calnexin OS=Mus musculus GN=Canx PE=1 SV=1 | 1551.47 | 1016.36 | 1594.21 | 1163.31 |
| P48962 | 4 (0) | 195.45 | --- | --- |  | ADT1\_MOUSE ADP/ATP translocase 1 OS=Mus musculus GN=Slc25a4 PE=1 SV=4 | --- | --- | --- | --- |
| P48036 | 3 (3) | 194.02 | 0.03 | 3.93 |  | ANXA5\_MOUSE Annexin A5 OS=Mus musculus GN=Anxa5 PE=1 SV=1 | 1083.25 | 275.40 | 786.04 | 324.98 |
| Q09098 | 3 (3) | 193.31 | 0.07 | 2.73 |  | PATE4\_MOUSE Prostate and testis expressed protein 4 OS=Mus musculus GN=Pate4 PE=1 SV=3 | 2.79e+004 | 7.62e+004 | 3.92e+004 | 6.95e+004 |
| P02762 | 3 (3) | 190.49 | 0.97 | 2.94 |  | MUP6\_MOUSE Major urinary protein 6 OS=Mus musculus GN=Mup6 PE=1 SV=2 | 536.35 | 303.04 | 890.18 | 581.09 |
| Q9D8E6 | 4 (4) | 190.02 | 0.28 | 1.36 |  | RL4\_MOUSE 60S ribosomal protein L4 OS=Mus musculus GN=Rpl4 PE=1 SV=3 | 3955.76 | 3253.38 | 4021.27 | 2951.80 |
| Q01853 | 4 (4) | 186.56 | 4.88e-003 | 4.91 |  | TERA\_MOUSE Transitional endoplasmic reticulum ATPase OS=Mus musculus GN=Vcp PE=1 SV=4 | 2766.75 | 563.29 | 1750.51 | 1114.71 |
| P21460 | 3 (3) | 185.71 | 0.34 | 1.50 |  | CYTC\_MOUSE Cystatin-C OS=Mus musculus GN=Cst3 PE=2 SV=2 | 2546.68 | 3680.30 | 2452.41 | 2849.92 |
| Q9D1G1 | 4 (1) | 185.65 | 0.07 | 1.81 |  | RAB1B\_MOUSE Ras-related protein Rab-1B OS=Mus musculus GN=Rab1b PE=1 SV=1 | 701.07 | 387.99 | 615.69 | 415.33 |
| Q8VHX6 | 4 (0) | 185.50 | --- | --- |  | FLNC\_MOUSE Filamin-C OS=Mus musculus GN=Flnc PE=1 SV=3 | --- | --- | --- | --- |
| P62821 | 4 (1) | 185.00 | 0.04 | 1.80 |  | RAB1A\_MOUSE Ras-related protein Rab-1A OS=Mus musculus GN=Rab1A PE=1 SV=3 | 3682.98 | 2045.60 | 3291.40 | 2432.98 |
| Q9QY48 | 4 (4) | 184.09 | 0.48 | 1.65 |  | DNS2B\_MOUSE Deoxyribonuclease-2-beta OS=Mus musculus GN=Dnase2b PE=2 SV=1 | 1627.97 | 1319.49 | 1800.31 | 1090.91 |
| Q78PY7 | 3 (3) | 182.24 | 0.62 | 1.23 |  | SND1\_MOUSE Staphylococcal nuclease domain-containing protein 1 OS=Mus musculus GN=Snd1 PE=1 SV=1 | 1580.28 | 1367.83 | 1393.07 | 1279.98 |
| Q921I1 | 4 (4) | 181.56 | 0.04 | 2.06 |  | TRFE\_MOUSE Serotransferrin OS=Mus musculus GN=Tf PE=1 SV=1 | 1003.94 | 514.46 | 902.04 | 488.50 |
| Q01768 | 4 (2) | 180.67 | 0.57 | 1.66 |  | NDKB\_MOUSE Nucleoside diphosphate kinase B OS=Mus musculus GN=Nme2 PE=1 SV=1 | 2182.56 | 2334.81 | 1882.46 | 3121.44 |
| Q3SXH3 | 3 (3) | 176.21 | 0.31 | 1.54 |  | Q3SXH3\_MOUSE Seminal vesicle antigen OS=Mus musculus GN=Sva PE=2 SV=1 | 1.07e+005 | 1.65e+005 | 1.21e+005 | 1.40e+005 |
| P01942 | 3 (3) | 176.17 | 0.92 | 1.12 |  | HBA\_MOUSE Hemoglobin subunit alpha OS=Mus musculus GN=Hba PE=1 SV=2 | 1.35e+004 | 1.42e+004 | 1.51e+004 | 1.40e+004 |
| P29341 | 4 (4) | 170.60 | 0.16 | 1.96 |  | PABP1\_MOUSE Polyadenylate-binding protein 1 OS=Mus musculus GN=Pabpc1 PE=1 SV=1 | 1586.57 | 809.66 | 1194.29 | 986.62 |
| P0C0S6 | 3 (1) | 170.46 | 0.98 | 1.07 |  | H2AZ\_MOUSE Histone H2A.Z OS=Mus musculus GN=H2afz PE=1 SV=2 | 688.20 | 739.67 | 723.60 | 693.61 |
| Q9DCD0 | 3 (3) | 167.82 | 0.02 | 2.62 |  | 6PGD\_MOUSE 6-phosphogluconate dehydrogenase, decarboxylating OS=Mus musculus GN=Pgd PE=2 SV=3 | 2496.72 | 1203.95 | 2052.74 | 952.29 |
| P47963 | 3 (3) | 167.58 | 0.85 | 1.21 |  | RL13\_MOUSE 60S ribosomal protein L13 OS=Mus musculus GN=Rpl13 PE=2 SV=3 | 1489.28 | 1320.00 | 1360.86 | 1598.27 |
| P14824 | 3 (3) | 166.28 | 0.76 | 1.19 |  | ANXA6\_MOUSE Annexin A6 OS=Mus musculus GN=Anxa6 PE=1 SV=2 | 438.06 | 403.14 | 481.72 | 411.56 |
| P14148 | 3 (3) | 163.09 | 0.50 | 1.29 |  | RL7\_MOUSE 60S ribosomal protein L7 OS=Mus musculus GN=Rpl7 PE=2 SV=2 | 3050.64 | 2370.75 | 2666.78 | 3060.88 |
| Q9DBF1 | 2 (2) | 162.26 | 0.08 | 2.28 |  | AL7A1\_MOUSE Alpha-aminoadipic semialdehyde dehydrogenase OS=Mus musculus GN=Aldh7a1 PE=1 SV=4 | 1156.37 | 514.26 | 1173.58 | 522.66 |
| P05202 | 3 (3) | 161.45 | 0.30 | 1.47 |  | AATM\_MOUSE Aspartate aminotransferase, mitochondrial OS=Mus musculus GN=Got2 PE=1 SV=1 | 2356.00 | 1602.33 | 2173.39 | 1674.64 |
| P35700 | 3 (3) | 161.05 | 0.96 | 1.10 |  | PRDX1\_MOUSE Peroxiredoxin-1 OS=Mus musculus GN=Prdx1 PE=1 SV=1 | 2052.62 | 2234.64 | 2041.38 | 2236.25 |
| P22752 | 3 (1) | 160.11 | 1.52e-003 | 2.64 |  | H2A1\_MOUSE Histone H2A type 1 OS=Mus musculus GN=Hist1h2ab PE=1 SV=3 | 3.72e+004 | 1.41e+004 | 3.12e+004 | 1.84e+004 |
| P17879 | 3 (1) | 157.98 | 0.04 | 2.26 |  | HS71B\_MOUSE Heat shock 70 kDa protein 1B OS=Mus musculus GN=Hspa1b PE=1 SV=3 | 363.34 | 160.43 | 313.97 | 219.86 |
| P43274 | 2 (2) | 153.91 | 0.92 | 1.20 |  | H14\_MOUSE Histone H1.4 OS=Mus musculus GN=Hist1h1e PE=1 SV=2 | 7594.78 | 8528.48 | 7095.32 | 8041.77 |
| Q6PDN3 | 3 (3) | 153.12 | 0.48 | 1.30 |  | MYLK\_MOUSE Myosin light chain kinase, smooth muscle OS=Mus musculus GN=Mylk PE=1 SV=3 | 4060.94 | 3644.75 | 4632.02 | 4727.00 |
| P01887 | 3 (3) | 152.23 | 0.09 | 1.90 |  | B2MG\_MOUSE Beta-2-microglobulin OS=Mus musculus GN=B2m PE=1 SV=1 | 2.41e+004 | 4.58e+004 | 3.18e+004 | 4.48e+004 |
| P62702 | 3 (3) | 150.69 | 0.06 | 1.78 |  | RS4X\_MOUSE 40S ribosomal protein S4, X isoform OS=Mus musculus GN=Rps4x PE=2 SV=2 | 973.26 | 547.91 | 922.01 | 904.13 |
| P62270 | 3 (3) | 149.60 | 0.43 | 1.31 |  | RS18\_MOUSE 40S ribosomal protein S18 OS=Mus musculus GN=Rps18 PE=2 SV=3 | 1424.59 | 1086.52 | 1250.33 | 1163.11 |
| P32921 | 4 (4) | 147.78 | 0.96 | 1.35 |  | SYWC\_MOUSE Tryptophanyl-tRNA synthetase, cytoplasmic OS=Mus musculus GN=Wars PE=1 SV=2 | 1124.57 | 1520.78 | 1284.57 | 1221.63 |
| P00329 | 2 (2) | 147.19 | 0.87 | 1.17 |  | ADH1\_MOUSE Alcohol dehydrogenase 1 OS=Mus musculus GN=Adh1 PE=2 SV=2 | 1495.57 | 1276.07 | 1479.71 | 1313.87 |
| P70296 | 2 (2) | 144.60 | 0.97 | 1.12 |  | PEBP1\_MOUSE Phosphatidylethanolamine-binding protein 1 OS=Mus musculus GN=Pebp1 PE=1 SV=3 | 2515.23 | 2337.85 | 2250.11 | 2257.07 |
| Q61171 | 2 (2) | 143.36 | 0.04 | 1.74 |  | PRDX2\_MOUSE Peroxiredoxin-2 OS=Mus musculus GN=Prdx2 PE=1 SV=3 | 2143.82 | 1288.07 | 2026.96 | 1229.46 |
| Q8BGZ7 | 2 (1) | 142.26 | 0.18 | 1.57 |  | K2C75\_MOUSE Keratin, type II cytoskeletal 75 OS=Mus musculus GN=Krt75 PE=1 SV=1 | 1331.88 | 1076.67 | 1097.16 | 849.82 |
| P08003 | 3 (3) | 139.31 | 0.20 | 1.69 |  | PDIA4\_MOUSE Protein disulfide-isomerase A4 OS=Mus musculus GN=Pdia4 PE=1 SV=2 | 845.29 | 501.65 | 670.70 | 630.68 |
| P62082 | 3 (3) | 137.91 | 0.10 | 1.74 |  | RS7\_MOUSE 40S ribosomal protein S7 OS=Mus musculus GN=Rps7 PE=2 SV=1 | 3231.86 | 1854.21 | 3108.54 | 2641.75 |
| P07356 | 2 (2) | 137.84 | 0.14 | 1.98 |  | ANXA2\_MOUSE Annexin A2 OS=Mus musculus GN=Anxa2 PE=1 SV=2 | 1088.50 | 548.53 | 1005.83 | 709.44 |
| Q60854 | 3 (3) | 135.77 | 0.15 | 1.42 |  | SPB6\_MOUSE Serpin B6 OS=Mus musculus GN=Serpinb6 PE=2 SV=1 | 1011.72 | 713.08 | 945.97 | 750.43 |
| O70251 | 2 (2) | 135.66 | 0.33 | 1.31 |  | EF1B\_MOUSE Elongation factor 1-beta OS=Mus musculus GN=Eef1b PE=1 SV=5 | 3148.41 | 2400.04 | 2636.93 | 2626.35 |
| P07901 | 3 (3) | 133.44 | 0.65 | 1.33 |  | HS90A\_MOUSE Heat shock protein HSP 90-alpha OS=Mus musculus GN=Hsp90aa1 PE=1 SV=4 | 1052.02 | 789.86 | 856.78 | 824.89 |
| Q61598 | 2 (2) | 132.65 | 0.02 | 1.69 |  | GDIB\_MOUSE Rab GDP dissociation inhibitor beta OS=Mus musculus GN=Gdi2 PE=1 SV=1 | 1897.90 | 1123.88 | 1765.74 | 1698.42 |
| P05784 | 3 (1) | 131.05 | 0.90 | 1.11 |  | K1C18\_MOUSE Keratin, type I cytoskeletal 18 OS=Mus musculus GN=Krt18 PE=1 SV=5 | 741.62 | 676.60 | 719.22 | 669.11 |
| Q9D1D4 | 2 (2) | 130.92 | 0.02 | 2.02 |  | TMEDA\_MOUSE Transmembrane emp24 domain-containing protein 10 OS=Mus musculus GN=Tmed10 PE=2 SV=1 | 1883.27 | 930.23 | 1664.83 | 1013.80 |
| P15532 | 3 (1) | 130.90 | 2.68e-003 | 2.60 |  | NDKA\_MOUSE Nucleoside diphosphate kinase A OS=Mus musculus GN=Nme1 PE=1 SV=1 | 1169.24 | 450.37 | 865.37 | 500.71 |
| Q8BP67 | 2 (2) | 129.89 | 0.68 | 1.24 |  | RL24\_MOUSE 60S ribosomal protein L24 OS=Mus musculus GN=Rpl24 PE=2 SV=2 | 889.78 | 900.98 | 1102.18 | 934.30 |
| P97351 | 3 (3) | 128.80 | 7.24e-004 | 2.91 |  | RS3A\_MOUSE 40S ribosomal protein S3a OS=Mus musculus GN=Rps3a PE=1 SV=3 | 1399.63 | 480.31 | 871.04 | 518.17 |
| Q9QZE5 | 3 (2) | 127.87 | 0.01 | 3.06 |  | COPG\_MOUSE Coatomer subunit gamma OS=Mus musculus GN=Copg PE=2 SV=1 | 1252.63 | 409.05 | 1107.06 | 586.03 |
| P62889 | 2 (2) | 127.62 | 0.17 | 1.43 |  | RL30\_MOUSE 60S ribosomal protein L30 OS=Mus musculus GN=Rpl30 PE=2 SV=2 | 3525.25 | 3261.64 | 3468.09 | 2469.25 |
| Q91YR9 | 3 (3) | 123.93 | 0.08 | 2.22 |  | PTGR1\_MOUSE Prostaglandin reductase 1 OS=Mus musculus GN=Ptgr1 PE=2 SV=2 | 822.07 | 422.22 | 565.88 | 370.65 |
| P14131 | 2 (2) | 123.55 | 0.74 | 1.23 |  | RS16\_MOUSE 40S ribosomal protein S16 OS=Mus musculus GN=Rps16 PE=2 SV=4 | 3642.96 | 3133.28 | 3624.48 | 3852.28 |
| P63038 | 2 (2) | 123.52 | 0.47 | 1.45 |  | CH60\_MOUSE 60 kDa heat shock protein, mitochondrial OS=Mus musculus GN=Hspd1 PE=1 SV=1 | 713.38 | 490.42 | 608.22 | 671.14 |
| Q9CY50 | 2 (2) | 123.34 | 6.66e-003 | 2.50 |  | SSRA\_MOUSE Translocon-associated protein subunit alpha OS=Mus musculus GN=Ssr1 PE=1 SV=1 | 1645.50 | 812.45 | 1174.13 | 656.95 |
| P62855 | 2 (2) | 123.19 | 0.03 | 1.63 |  | RS26\_MOUSE 40S ribosomal protein S26 OS=Mus musculus GN=Rps26 PE=2 SV=3 | 5698.60 | 3616.94 | 5892.38 | 3753.38 |
| P45878 | 3 (3) | 122.04 | 0.91 | 1.13 |  | FKBP2\_MOUSE Peptidyl-prolyl cis-trans isomerase FKBP2 OS=Mus musculus GN=Fkbp2 PE=1 SV=1 | 1887.43 | 1920.49 | 1704.00 | 1795.28 |
| Q9CXW4 | 2 (2) | 121.46 | 0.55 | 1.20 |  | RL11\_MOUSE 60S ribosomal protein L11 OS=Mus musculus GN=Rpl11 PE=1 SV=4 | 1627.97 | 1356.62 | 1606.29 | 1427.51 |
| P62830 | 3 (3) | 120.79 | 0.05 | 1.59 |  | RL23\_MOUSE 60S ribosomal protein L23 OS=Mus musculus GN=Rpl23 PE=2 SV=1 | 2820.72 | 1773.93 | 2580.09 | 1984.01 |
| Q9EQ20 | 3 (3) | 119.35 | 0.04 | 1.57 |  | MMSA\_MOUSE Methylmalonate-semialdehyde dehydrogenase [acylating], mitochondrial OS=Mus musculus GN=Aldh6a1 PE=1 SV=1 | 510.36 | 344.90 | 478.17 | 324.29 |
| P67984 | 2 (2) | 117.14 | 0.76 | 1.27 |  | RL22\_MOUSE 60S ribosomal protein L22 OS=Mus musculus GN=Rpl22 PE=2 SV=2 | 2928.32 | 3458.82 | 3724.96 | 3524.27 |
| Q9JIF7 | 2 (2) | 116.58 | 0.03 | 2.93 |  | COPB\_MOUSE Coatomer subunit beta OS=Mus musculus GN=Copb1 PE=1 SV=1 | 757.69 | 260.15 | 546.04 | 258.87 |
| P35979 | 1 (1) | 116.11 | 0.06 | 1.68 |  | RL12\_MOUSE 60S ribosomal protein L12 OS=Mus musculus GN=Rpl12 PE=1 SV=2 | 2516.04 | 1500.02 | 2219.49 | 1972.40 |
| Q60930 | 2 (2) | 114.98 | 0.96 | 1.17 |  | VDAC2\_MOUSE Voltage-dependent anion-selective channel protein 2 OS=Mus musculus GN=Vdac2 PE=1 SV=2 | 592.75 | 693.15 | 621.94 | 664.39 |
| Q99PL5 | 3 (3) | 114.11 | 0.77 | 1.85 |  | RRBP1\_MOUSE Ribosome-binding protein 1 OS=Mus musculus GN=Rrbp1 PE=2 SV=2 | 564.84 | 715.43 | 532.45 | 984.59 |
| Q06890 | 2 (2) | 113.76 | 0.25 | 2.13 |  | CLUS\_MOUSE Clusterin OS=Mus musculus GN=Clu PE=1 SV=1 | 1574.63 | 1233.00 | 1173.56 | 739.30 |
| P07759 | 3 (3) | 113.08 | 0.03 | 2.12 |  | SPA3K\_MOUSE Serine protease inhibitor A3K OS=Mus musculus GN=Serpina3k PE=1 SV=2 | 1363.68 | 1263.63 | 1678.46 | 793.40 |
| P62849 | 2 (2) | 113.00 | 0.33 | 1.74 |  | RS24\_MOUSE 40S ribosomal protein S24 OS=Mus musculus GN=Rps24 PE=1 SV=1 | 2307.15 | 1376.75 | 2401.00 | 1759.01 |
| P84099 | 2 (2) | 111.75 | 0.16 | 1.44 |  | RL19\_MOUSE 60S ribosomal protein L19 OS=Mus musculus GN=Rpl19 PE=1 SV=1 | 1485.33 | 1032.06 | 1338.92 | 1179.07 |
| Q64433 | 2 (2) | 111.29 | 0.06 | 1.79 |  | CH10\_MOUSE 10 kDa heat shock protein, mitochondrial OS=Mus musculus GN=Hspe1 PE=1 SV=2 | 717.71 | 457.27 | 468.13 | 401.22 |
| Q3UN54 | 2 (2) | 111.20 | 0.51 | 1.33 |  | SSLP1\_MOUSE Secreted seminal-vesicle Ly-6 protein 1 OS=Mus musculus GN=Sslp1 PE=1 SV=1 | 4711.40 | 5990.76 | 5140.57 | 6253.91 |
| Q9DBJ1 | 3 (3) | 110.07 | 0.27 | 2.07 |  | PGAM1\_MOUSE Phosphoglycerate mutase 1 OS=Mus musculus GN=Pgam1 PE=1 SV=3 | 2552.99 | 1231.70 | 2116.86 | 1501.26 |
| P57776 | 2 (2) | 109.85 | 0.12 | 1.43 |  | EF1D\_MOUSE Elongation factor 1-delta OS=Mus musculus GN=Eef1d PE=1 SV=3 | 5899.49 | 5744.70 | 7299.22 | 8232.02 |
| P13020 | 2 (2) | 109.23 | 0.04 | 1.78 |  | GELS\_MOUSE Gelsolin OS=Mus musculus GN=Gsn PE=1 SV=3 | 1299.35 | 731.70 | 1083.54 | 739.32 |
| P61255 | 3 (3) | 107.66 | 0.51 | 1.56 |  | RL26\_MOUSE 60S ribosomal protein L26 OS=Mus musculus GN=Rpl26 PE=2 SV=1 | 350.77 | 235.42 | 245.61 | 367.75 |
| Q8CGC7 | 2 (2) | 106.73 | 0.39 | 1.67 |  | SYEP\_MOUSE Bifunctional aminoacyl-tRNA synthetase OS=Mus musculus GN=Eprs PE=2 SV=3 | 434.98 | 283.59 | 472.83 | 380.63 |
| Q9CZX8 | 2 (2) | 105.68 | 0.49 | 1.36 |  | RS19\_MOUSE 40S ribosomal protein S19 OS=Mus musculus GN=Rps19 PE=1 SV=3 | 1876.16 | 1463.67 | 1989.87 | 1570.00 |
| P40142 | 2 (2) | 104.66 | 0.06 | 2.40 |  | TKT\_MOUSE Transketolase OS=Mus musculus GN=Tkt PE=1 SV=1 | 801.43 | 333.82 | 801.78 | 339.38 |
| P00687 | 2 (2) | 104.29 | 0.92 | 1.88 |  | AMY1\_MOUSE Alpha-amylase 1 OS=Mus musculus GN=Amy1 PE=1 SV=1 | 752.89 | 1409.98 | 748.02 | 859.50 |
| P48774 | 2 (1) | 104.19 | 0.98 | 1.70 |  | GSTM5\_MOUSE Glutathione S-transferase Mu 5 OS=Mus musculus GN=Gstm5 PE=1 SV=1 | 91.47 | 53.92 | 66.47 | 64.34 |
| P14115 | 2 (2) | 103.63 | 0.85 | 1.69 |  | RL27A\_MOUSE 60S ribosomal protein L27a OS=Mus musculus GN=Rpl27a PE=2 SV=5 | 2534.04 | 2902.85 | 2545.60 | 4294.82 |
| P14869 | 2 (2) | 103.33 | 0.01 | 3.45 |  | RLA0\_MOUSE 60S acidic ribosomal protein P0 OS=Mus musculus GN=Rplp0 PE=1 SV=3 | 3144.29 | 1160.92 | 2396.03 | 910.56 |
| Q9D8V7 | 2 (2) | 102.91 | 0.27 | 2.28 |  | SC11C\_MOUSE Signal peptidase complex catalytic subunit SEC11C OS=Mus musculus GN=Sec11c PE=2 SV=3 | 846.02 | 370.84 | 781.60 | 618.15 |
| P11087 | 1 (1) | 102.90 | 0.51 | 3.04 |  | CO1A1\_MOUSE Collagen alpha-1(I) chain OS=Mus musculus GN=Col1a1 PE=1 SV=4 | 2096.20 | 778.01 | 1567.66 | 2361.68 |
| P62717 | 2 (2) | 102.24 | 0.27 | 1.41 |  | RL18A\_MOUSE 60S ribosomal protein L18a OS=Mus musculus GN=Rpl18a PE=1 SV=1 | 1820.62 | 1506.53 | 1677.54 | 1293.47 |
| P15105 | 2 (2) | 100.92 | 0.61 | 1.20 |  | GLNA\_MOUSE Glutamine synthetase OS=Mus musculus GN=Glul PE=1 SV=6 | 861.83 | 718.52 | 781.73 | 715.96 |
| P18242 | 3 (3) | 100.72 | 2.26e-004 | 2.07 |  | CATD\_MOUSE Cathepsin D OS=Mus musculus GN=Ctsd PE=1 SV=1 | 5355.67 | 3390.65 | 5476.01 | 2650.79 |
| Q9CPU0 | 2 (2) | 100.02 | 0.49 | 1.83 |  | LGUL\_MOUSE Lactoylglutathione lyase OS=Mus musculus GN=Glo1 PE=1 SV=3 | 991.77 | 722.71 | 718.67 | 542.27 |
| Q9CR57 | 1 (1) | 99.94 | 0.09 | 1.84 |  | RL14\_MOUSE 60S ribosomal protein L14 OS=Mus musculus GN=Rpl14 PE=2 SV=3 | 3646.51 | 3017.74 | 4439.28 | 2406.34 |
| P63028 | 2 (2) | 98.76 | 8.54e-003 | 2.16 |  | TCTP\_MOUSE Translationally-controlled tumor protein OS=Mus musculus GN=Tpt1 PE=1 SV=1 | 1378.76 | 676.45 | 991.15 | 637.21 |
| P61027 | 2 (1) | 98.14 | 9.82e-003 | 2.80 |  | RAB10\_MOUSE Ras-related protein Rab-10 OS=Mus musculus GN=Rab10 PE=1 SV=1 | 438.16 | 156.58 | 386.78 | 211.31 |
| P99029 | 2 (2) | 93.21 | 0.06 | 4.48 |  | PRDX5\_MOUSE Peroxiredoxin-5, mitochondrial OS=Mus musculus GN=Prdx5 PE=1 SV=2 | 1194.73 | 266.40 | 657.01 | 381.23 |
| Q62186 | 2 (2) | 93.16 | 0.08 | 1.68 |  | SSRD\_MOUSE Translocon-associated protein subunit delta OS=Mus musculus GN=Ssr4 PE=2 SV=1 | 2890.68 | 1717.00 | 2719.08 | 2011.71 |
| Q921F2 | 2 (2) | 91.67 | 0.40 | 3.53 |  | TADBP\_MOUSE TAR DNA-binding protein 43 OS=Mus musculus GN=Tardbp PE=1 SV=1 | 282.44 | 79.98 | 250.69 | 175.03 |
| P47962 | 1 (1) | 91.52 | 0.04 | 3.07 |  | RL5\_MOUSE 60S ribosomal protein L5 OS=Mus musculus GN=Rpl5 PE=1 SV=3 | 1780.50 | 580.61 | 1400.10 | 767.68 |
| Q9CYN2 | 2 (2) | 91.22 | 0.98 | 1.11 |  | SPCS2\_MOUSE Signal peptidase complex subunit 2 OS=Mus musculus GN=Spcs2 PE=2 SV=1 | 997.02 | 948.35 | 897.24 | 958.81 |
| P42125 | 2 (2) | 90.91 | 0.62 | 1.50 |  | D3D2\_MOUSE 3,2-trans-enoyl-CoA isomerase, mitochondrial OS=Mus musculus GN=Dci PE=2 SV=1 | 432.09 | 288.46 | 412.01 | 356.78 |
| P18760 | 2 (2) | 90.64 | 0.77 | 1.19 |  | COF1\_MOUSE Cofilin-1 OS=Mus musculus GN=Cfl1 PE=1 SV=3 | 3486.20 | 2981.52 | 3537.46 | 2996.54 |
| O55029 | 2 (2) | 88.59 | 0.24 | 1.84 |  | COPB2\_MOUSE Coatomer subunit beta' OS=Mus musculus GN=Copb2 PE=2 SV=2 | 724.41 | 466.13 | 536.78 | 392.83 |
| Q8VDN2 | 2 (2) | 88.08 | 0.09 | 2.94 |  | AT1A1\_MOUSE Sodium/potassium-transporting ATPase subunit alpha-1 OS=Mus musculus GN=Atp1a1 PE=1 SV=1 | 488.60 | 166.45 | 361.96 | 207.27 |
| Q8R086 | 2 (2) | 87.18 | 0.02 | 2.53 |  | SUOX\_MOUSE Sulfite oxidase, mitochondrial OS=Mus musculus GN=Suox PE=1 SV=2 | 755.32 | 312.20 | 681.85 | 299.05 |
| Q9QYB1 | 1 (1) | 85.98 | 0.41 | 1.90 |  | CLIC4\_MOUSE Chloride intracellular channel protein 4 OS=Mus musculus GN=Clic4 PE=1 SV=3 | 261.19 | 260.41 | 332.53 | 175.34 |
| P15327 | 2 (2) | 84.85 | 0.20 | 1.52 |  | PMGE\_MOUSE Bisphosphoglycerate mutase OS=Mus musculus GN=Bpgm PE=2 SV=2 | 2036.06 | 1401.75 | 2096.61 | 1376.97 |
| Q64310 | 1 (1) | 84.58 | 0.09 | 2.71 |  | SURF4\_MOUSE Surfeit locus protein 4 OS=Mus musculus GN=Surf4 PE=2 SV=1 | 3393.65 | 1251.08 | 3171.49 | 1664.28 |
| Q91V61 | 2 (2) | 84.09 | 0.82 | 1.06 |  | SFXN3\_MOUSE Sideroflexin-3 OS=Mus musculus GN=Sfxn3 PE=1 SV=1 | 1293.17 | 1273.16 | 1345.89 | 1279.54 |
| P26040 | 2 (2) | 82.76 | 0.51 | 1.32 |  | EZRI\_MOUSE Ezrin OS=Mus musculus GN=Ezr PE=1 SV=3 | 1199.59 | 911.80 | 1140.96 | 1162.79 |
| Q9D8N0 | 1 (1) | 82.42 | 0.60 | 1.64 |  | EF1G\_MOUSE Elongation factor 1-gamma OS=Mus musculus GN=Eef1g PE=1 SV=3 | 1606.99 | 1875.71 | 1405.61 | 2302.53 |
| P62301 | 2 (2) | 82.11 | 0.69 | 1.21 |  | RS13\_MOUSE 40S ribosomal protein S13 OS=Mus musculus GN=Rps13 PE=1 SV=2 | 900.88 | 742.35 | 804.72 | 881.24 |
| Q9CYH2 | 2 (2) | 82.05 | 0.05 | 3.04 |  | CJ058\_MOUSE UPF0765 protein C10orf58 homolog OS=Mus musculus PE=1 SV=2 | 926.62 | 575.08 | 474.00 | 304.55 |
| P62858 | 1 (1) | 80.16 | 0.46 | 1.42 |  | RS28\_MOUSE 40S ribosomal protein S28 OS=Mus musculus GN=Rps28 PE=2 SV=1 | 5362.69 | 4503.51 | 4991.21 | 3785.22 |
| P62267 | 1 (1) | 79.62 | 0.64 | 1.54 |  | RS23\_MOUSE 40S ribosomal protein S23 OS=Mus musculus GN=Rps23 PE=2 SV=3 | 3098.69 | 4762.69 | 3717.96 | 4007.35 |
| Q91YW3 | 2 (1) | 78.38 | 0.37 | 1.29 |  | DNJC3\_MOUSE DnaJ homolog subfamily C member 3 OS=Mus musculus GN=Dnajc3 PE=1 SV=1 | 153.45 | 121.01 | 149.98 | 119.40 |
| Q9DB20 | 2 (2) | 77.95 | 0.30 | 1.92 |  | ATPO\_MOUSE ATP synthase subunit O, mitochondrial OS=Mus musculus GN=Atp5o PE=1 SV=1 | 1161.96 | 606.61 | 995.22 | 854.30 |
| O88569 | 1 (1) | 77.88 | 0.07 | 4.45 |  | ROA2\_MOUSE Heterogeneous nuclear ribonucleoproteins A2/B1 OS=Mus musculus GN=Hnrnpa2b1 PE=1 SV=2 | 1512.83 | 400.88 | 1170.66 | 340.27 |
| Q9D0I9 | 2 (2) | 77.19 | 0.62 | 1.29 |  | SYRC\_MOUSE Arginyl-tRNA synthetase, cytoplasmic OS=Mus musculus GN=Rars PE=2 SV=2 | 1.36e+004 | 1.71e+004 | 1.65e+004 | 1.75e+004 |
| P60867 | 2 (2) | 77.17 | 0.26 | 1.55 |  | RS20\_MOUSE 40S ribosomal protein S20 OS=Mus musculus GN=Rps20 PE=1 SV=1 | 955.43 | 617.06 | 757.82 | 883.97 |
| Q99MN1 | 2 (2) | 76.45 | 0.09 | 1.80 |  | SYK\_MOUSE Lysyl-tRNA synthetase OS=Mus musculus GN=Kars PE=1 SV=1 | 367.96 | 216.98 | 362.37 | 204.58 |
| Q99LX0 | 2 (2) | 76.13 | 0.72 | 1.17 |  | PARK7\_MOUSE Protein DJ-1 OS=Mus musculus GN=Park7 PE=1 SV=1 | 755.13 | 658.29 | 654.03 | 644.44 |
| P68433 | 2 (2) | 75.69 | 0.86 | 1.13 |  | H31\_MOUSE Histone H3.1 OS=Mus musculus GN=Hist1h3a PE=1 SV=2 | 9361.95 | 9367.83 | 8254.56 | 9033.53 |
| O55143 | 2 (2) | 75.48 | 0.02 | 5.93 |  | AT2A2\_MOUSE Sarcoplasmic/endoplasmic reticulum calcium ATPase 2 OS=Mus musculus GN=Atp2a2 PE=1 SV=2 | 564.73 | 95.27 | 525.32 | 214.64 |
| Q9CZU6 | 2 (2) | 75.39 | 0.37 | 1.39 |  | CISY\_MOUSE Citrate synthase, mitochondrial OS=Mus musculus GN=Cs PE=1 SV=1 | 814.91 | 633.95 | 762.49 | 584.76 |
| Q61735 | 1 (1) | 75.35 | 7.16e-003 | 10.26 |  | CD47\_MOUSE Leukocyte surface antigen CD47 OS=Mus musculus GN=Cd47 PE=1 SV=2 | 830.71 | 80.95 | 556.32 | 193.92 |
| P61804 | 1 (1) | 75.18 | 0.87 | 1.15 |  | DAD1\_MOUSE Dolichyl-diphosphooligosaccharide--protein glycosyltransferase subunit DAD1 OS=Mus musculus GN=Dad1 PE=2 SV=3 | 2793.77 | 2966.43 | 2974.74 | 2591.77 |
| P12970 | 2 (2) | 74.26 | 0.49 | 1.28 |  | RL7A\_MOUSE 60S ribosomal protein L7a OS=Mus musculus GN=Rpl7a PE=2 SV=2 | 975.20 | 815.73 | 885.10 | 759.67 |
| P06745 | 2 (2) | 73.40 | 2.39e-003 | 3.04 |  | G6PI\_MOUSE Glucose-6-phosphate isomerase OS=Mus musculus GN=Gpi PE=1 SV=4 | 1994.85 | 1153.80 | 1933.30 | 655.30 |
| Q8BH04 | 1 (1) | 73.37 | 0.05 | 3.61 |  | PCKGM\_MOUSE Phosphoenolpyruvate carboxykinase [GTP], mitochondrial OS=Mus musculus GN=Pck2 PE=2 SV=1 | 453.95 | 125.86 | 327.90 | 254.79 |
| Q62095 | 2 (2) | 72.75 | 0.07 | 2.76 |  | DDX3Y\_MOUSE ATP-dependent RNA helicase DDX3Y OS=Mus musculus GN=Ddx3y PE=1 SV=2 | 619.36 | 224.52 | 549.38 | 291.14 |
| Q8R2E9 | 2 (2) | 72.61 | 0.41 | 1.39 |  | ERO1B\_MOUSE ERO1-like protein beta OS=Mus musculus GN=Ero1lb PE=2 SV=1 | 916.81 | 1053.82 | 1275.76 | 960.88 |
| P13707 | 2 (2) | 72.53 | 0.15 | 1.51 |  | GPDA\_MOUSE Glycerol-3-phosphate dehydrogenase [NAD+], cytoplasmic OS=Mus musculus GN=Gpd1 PE=1 SV=3 | 312.54 | 258.09 | 264.81 | 207.41 |
| P06869 | 2 (2) | 72.31 | 0.17 | 2.23 |  | UROK\_MOUSE Urokinase-type plasminogen activator OS=Mus musculus GN=Plau PE=1 SV=1 | 161.00 | 359.64 | 258.19 | 323.05 |
| P02468 | 1 (1) | 72.18 | 0.66 | 8.64 |  | LAMC1\_MOUSE Laminin subunit gamma-1 OS=Mus musculus GN=Lamc1 PE=1 SV=2 | 452.39 | 52.38 | 286.25 | 294.99 |
| Q921U8 | 1 (1) | 72.15 | 0.53 | 6.75 |  | SMTN\_MOUSE Smoothelin OS=Mus musculus GN=Smtn PE=2 SV=2 | 278.51 | 41.25 | 188.60 | 186.82 |
| Q922Q4 | 1 (1) | 71.31 | 0.55 | 1.56 |  | P5CR2\_MOUSE Pyrroline-5-carboxylate reductase 2 OS=Mus musculus GN=Pycr2 PE=2 SV=1 | 553.73 | 384.78 | 353.84 | 442.98 |
| P97447 | 1 (1) | 71.02 | 0.81 | 3.81 |  | FHL1\_MOUSE Four and a half LIM domains protein 1 OS=Mus musculus GN=Fhl1 PE=2 SV=3 | 796.44 | 208.97 | 456.14 | 295.33 |
| P26039 | 2 (2) | 70.46 | 0.14 | 4.39 |  | TLN1\_MOUSE Talin-1 OS=Mus musculus GN=Tln1 PE=1 SV=1 | 454.22 | 103.57 | 447.32 | 202.33 |
| P27659 | 2 (2) | 68.89 | 0.91 | 1.16 |  | RL3\_MOUSE 60S ribosomal protein L3 OS=Mus musculus GN=Rpl3 PE=2 SV=2 | 1086.05 | 939.38 | 968.16 | 943.57 |
| P57759 | 2 (2) | 67.90 | 0.57 | 1.48 |  | ERP29\_MOUSE Endoplasmic reticulum resident protein 29 OS=Mus musculus GN=Erp29 PE=1 SV=2 | 1266.95 | 853.58 | 1219.74 | 1166.06 |
| Q922Q8 | 2 (2) | 67.68 | 0.33 | 1.69 |  | LRC59\_MOUSE Leucine-rich repeat-containing protein 59 OS=Mus musculus GN=Lrrc59 PE=2 SV=1 | 1542.15 | 930.29 | 1574.50 | 1374.12 |
| O09159 | 1 (1) | 67.58 | 0.72 | 1.20 |  | MA2B1\_MOUSE Lysosomal alpha-mannosidase OS=Mus musculus GN=Man2b1 PE=2 SV=3 | 514.24 | 472.66 | 429.41 | 491.68 |
| Q91V04 | 1 (1) | 67.16 | 0.13 | 2.04 |  | TRAM1\_MOUSE Translocating chain-associated membrane protein 1 OS=Mus musculus GN=Tram1 PE=1 SV=3 | 1263.51 | 727.69 | 1049.09 | 617.86 |
| Q9CQR2 | 1 (1) | 66.95 | 0.99 | 1.14 |  | RS21\_MOUSE 40S ribosomal protein S21 OS=Mus musculus GN=Rps21 PE=2 SV=1 | 2350.06 | 2502.82 | 2213.05 | 2196.20 |
| P60334 | 2 (2) | 66.02 | 0.84 | 1.24 |  | CDO1\_MOUSE Cysteine dioxygenase type 1 OS=Mus musculus GN=Cdo1 PE=1 SV=1 | 750.99 | 683.90 | 755.22 | 849.51 |
| Q8VEM8 | 1 (1) | 65.83 | 1.43e-003 | 7.55 |  | MPCP\_MOUSE Phosphate carrier protein, mitochondrial OS=Mus musculus GN=Slc25a3 PE=1 SV=1 | 468.00 | 62.00 | 259.66 | 84.57 |
| Q99LC5 | 2 (2) | 65.76 | 0.09 | 3.76 |  | ETFA\_MOUSE Electron transfer flavoprotein subunit alpha, mitochondrial OS=Mus musculus GN=Etfa PE=1 SV=2 | 772.93 | 205.58 | 708.64 | 493.62 |
| Q00896 | 2 (2) | 65.48 | 0.20 | 1.38 |  | A1AT3\_MOUSE Alpha-1-antitrypsin 1-3 OS=Mus musculus GN=Serpina1c PE=1 SV=2 | 801.71 | 801.28 | 858.21 | 621.78 |
| Q8BP47 | 2 (2) | 64.98 | 0.09 | 1.79 |  | SYNC\_MOUSE Asparaginyl-tRNA synthetase, cytoplasmic OS=Mus musculus GN=Nars PE=1 SV=2 | 480.27 | 313.42 | 561.21 | 380.56 |
| P62900 | 1 (1) | 64.03 | 0.38 | 1.25 |  | RL31\_MOUSE 60S ribosomal protein L31 OS=Mus musculus GN=Rpl31 PE=2 SV=1 | 7721.79 | 6182.24 | 7534.07 | 6176.39 |
| P61979 | 1 (1) | 63.65 | 0.07 | 1.79 |  | HNRPK\_MOUSE Heterogeneous nuclear ribonucleoprotein K OS=Mus musculus GN=Hnrnpk PE=1 SV=1 | 2144.90 | 1199.78 | 2050.55 | 1372.46 |
| A3KMP2 | 1 (1) | 62.81 | 0.40 | 2.10 |  | TTC38\_MOUSE Tetratricopeptide repeat protein 38 OS=Mus musculus GN=Ttc38 PE=2 SV=2 | 125.26 | 66.09 | 138.57 | 93.90 |
| P62911 | 1 (1) | 62.02 | 0.19 | 1.69 |  | RL32\_MOUSE 60S ribosomal protein L32 OS=Mus musculus GN=Rpl32 PE=2 SV=2 | 356.19 | 345.08 | 421.37 | 249.31 |
| Q9Z1Z0 | 1 (1) | 61.22 | 0.04 | 5.93 |  | USO1\_MOUSE General vesicular transport factor p115 OS=Mus musculus GN=Uso1 PE=1 SV=2 | 455.31 | 76.84 | 344.17 | 263.07 |
| P49312 | 1 (1) | 61.21 | 0.02 | 3.52 |  | ROA1\_MOUSE Heterogeneous nuclear ribonucleoprotein A1 OS=Mus musculus GN=Hnrnpa1 PE=1 SV=2 | 325.44 | 109.05 | 297.08 | 92.45 |
| Q99JI6 | 1 (1) | 60.41 | 0.02 | 5.27 |  | RAP1B\_MOUSE Ras-related protein Rap-1b OS=Mus musculus GN=Rap1b PE=2 SV=2 | 1600.60 | 593.02 | 1187.10 | 303.80 |
| Q61024 | 2 (2) | 59.83 | 5.59e-003 | 3.78 |  | ASNS\_MOUSE Asparagine synthetase [glutamine-hydrolyzing] OS=Mus musculus GN=Asns PE=2 SV=3 | 677.33 | 179.39 | 521.81 | 317.97 |
| Q5XJY5 | 1 (1) | 59.74 | 0.47 | 1.59 |  | COPD\_MOUSE Coatomer subunit delta OS=Mus musculus GN=Arcn1 PE=2 SV=1 | 998.06 | 625.80 | 838.89 | 919.98 |
| P62852 | 1 (1) | 58.98 | 0.88 | 1.15 |  | RS25\_MOUSE 40S ribosomal protein S25 OS=Mus musculus GN=Rps25 PE=2 SV=1 | 2573.62 | 2236.10 | 2308.64 | 2508.71 |
| Q62425 | 1 (1) | 57.94 | 0.32 | 2.05 |  | NDUA4\_MOUSE NADH dehydrogenase [ubiquinone] 1 alpha subcomplex subunit 4 OS=Mus musculus GN=Ndufa4 PE=1 SV=2 | 2245.51 | 1095.95 | 2121.18 | 1892.27 |
| P10107 | 1 (1) | 57.50 | 0.08 | 7.13 |  | ANXA1\_MOUSE Annexin A1 OS=Mus musculus GN=Anxa1 PE=1 SV=2 | 357.50 | 50.16 | 245.25 | 75.71 |
| Q9R0P6 | 1 (1) | 57.37 | 0.08 | 2.70 |  | SC11A\_MOUSE Signal peptidase complex catalytic subunit SEC11A OS=Mus musculus GN=Sec11a PE=2 SV=1 | 1059.60 | 392.94 | 860.74 | 427.16 |
| P97429 | 1 (1) | 57.08 | 4.13e-003 | 8.14 |  | ANXA4\_MOUSE Annexin A4 OS=Mus musculus GN=Anxa4 PE=2 SV=3 | 1994.56 | 245.13 | 921.61 | 505.44 |
| Q8CIE6 | 1 (1) | 56.57 | 0.02 | 4.46 |  | COPA\_MOUSE Coatomer subunit alpha OS=Mus musculus GN=Copa PE=1 SV=1 | 1157.26 | 259.71 | 898.12 | 553.84 |
| P34884 | 1 (1) | 56.53 | 0.64 | 1.40 |  | MIF\_MOUSE Macrophage migration inhibitory factor OS=Mus musculus GN=Mif PE=1 SV=2 | 792.64 | 673.37 | 648.30 | 566.93 |
| P49817 | 1 (1) | 56.33 | 0.03 | 92.84 |  | CAV1\_MOUSE Caveolin-1 OS=Mus musculus GN=Cav1 PE=1 SV=1 | 869.78 | 9.55 | 886.21 | 199.14 |
| Q01149 | 1 (1) | 56.29 | 0.17 | 72.12 |  | CO1A2\_MOUSE Collagen alpha-2(I) chain OS=Mus musculus GN=Col1a2 PE=2 SV=2 | 942.27 | 13.06 | 538.81 | 188.13 |
| P10630 | 1 (1) | 56.16 | 0.70 | 1.33 |  | IF4A2\_MOUSE Eukaryotic initiation factor 4A-II OS=Mus musculus GN=Eif4a2 PE=2 SV=2 | 2201.13 | 2160.16 | 2863.66 | 2227.50 |
| P14602 | 1 (1) | 56.13 | 0.61 | 2.35 |  | HSPB1\_MOUSE Heat shock protein beta-1 OS=Mus musculus GN=Hspb1 PE=1 SV=3 | 872.95 | 1199.00 | 1073.29 | 2053.48 |
| P80316 | 1 (1) | 56.08 | 0.02 | 6.37 |  | TCPE\_MOUSE T-complex protein 1 subunit epsilon OS=Mus musculus GN=Cct5 PE=1 SV=1 | 208.01 | 32.63 | 164.77 | 84.73 |
| P47955 | 1 (1) | 56.06 | 0.15 | 2.66 |  | RLA1\_MOUSE 60S acidic ribosomal protein P1 OS=Mus musculus GN=Rplp1 PE=1 SV=1 | 1.86e+004 | 6991.37 | 1.61e+004 | 1.29e+004 |
| Q60932 | 1 (1) | 55.81 | 0.88 | 1.10 |  | VDAC1\_MOUSE Voltage-dependent anion-selective channel protein 1 OS=Mus musculus GN=Vdac1 PE=1 SV=3 | 1046.21 | 1050.66 | 1147.19 | 1046.34 |
| P20108 | 1 (1) | 55.45 | 0.38 | 1.64 |  | PRDX3\_MOUSE Thioredoxin-dependent peroxide reductase, mitochondrial OS=Mus musculus GN=Prdx3 PE=1 SV=1 | 912.26 | 554.75 | 772.65 | 736.81 |
| P62264 | 1 (1) | 54.34 | 0.44 | 1.51 |  | RS14\_MOUSE 40S ribosomal protein S14 OS=Mus musculus GN=Rps14 PE=2 SV=3 | 3318.66 | 2199.87 | 2647.98 | 2706.08 |
| Q62465 | 1 (1) | 54.20 | 0.38 | 1.67 |  | VAT1\_MOUSE Synaptic vesicle membrane protein VAT-1 homolog OS=Mus musculus GN=Vat1 PE=1 SV=3 | 494.63 | 296.33 | 471.06 | 392.43 |
| Q78XF5 | 1 (1) | 54.09 | 0.44 | 1.70 |  | OSTC\_MOUSE Oligosaccharyltransferase complex subunit OSTC OS=Mus musculus GN=Ostc PE=2 SV=1 | 1292.27 | 761.80 | 992.57 | 1120.78 |
| Q6ZWY3 | 2 (2) | 53.55 | 0.04 | 1.47 |  | RS27L\_MOUSE 40S ribosomal protein S27-like OS=Mus musculus GN=Rps27l PE=2 SV=3 | 1416.85 | 2006.34 | 1368.33 | 1572.92 |
| P10922 | 1 (1) | 53.11 | 0.05 | 2.06 |  | H10\_MOUSE Histone H1.0 OS=Mus musculus GN=H1f0 PE=2 SV=4 | 2134.76 | 1134.46 | 1679.12 | 1038.06 |
| Q7TMK9 | 1 (1) | 52.73 | 0.05 | 4.19 |  | HNRPQ\_MOUSE Heterogeneous nuclear ribonucleoprotein Q OS=Mus musculus GN=Syncrip PE=1 SV=2 | 939.87 | 224.51 | 895.12 | 524.43 |
| P50580 | 1 (1) | 51.99 | 0.04 | 2.70 |  | PA2G4\_MOUSE Proliferation-associated protein 2G4 OS=Mus musculus GN=Pa2g4 PE=1 SV=3 | 954.81 | 367.48 | 763.78 | 353.87 |
| P29387 | 1 (1) | 51.61 | 0.11 | 5.36 |  | GBB4\_MOUSE Guanine nucleotide-binding protein subunit beta-4 OS=Mus musculus GN=Gnb4 PE=2 SV=4 | 265.67 | 49.57 | 142.32 | 82.02 |
| Q8BH64 | 1 (1) | 51.31 | 0.05 | 3.80 |  | EHD2\_MOUSE EH domain-containing protein 2 OS=Mus musculus GN=Ehd2 PE=1 SV=1 | 353.07 | 95.48 | 362.57 | 138.51 |
| P14069 | 1 (1) | 51.27 | 0.64 | 1.21 |  | S10A6\_MOUSE Protein S100-A6 OS=Mus musculus GN=S100a6 PE=1 SV=3 | 3161.94 | 3838.94 | 3315.63 | 3711.59 |
| Q9CZM2 | 1 (1) | 51.14 | 0.59 | 1.34 |  | RL15\_MOUSE 60S ribosomal protein L15 OS=Mus musculus GN=Rpl15 PE=2 SV=4 | 2540.17 | 2704.16 | 2011.93 | 2228.79 |
| Q8K2B3 | 1 (1) | 50.52 | 0.03 | 6.93 |  | DHSA\_MOUSE Succinate dehydrogenase [ubiquinone] flavoprotein subunit, mitochondrial OS=Mus musculus GN=Sdha PE=1 SV=1 | 255.35 | 36.83 | 164.37 | 63.55 |
| Q9JJI8 | 1 (1) | 50.43 | 0.49 | 1.45 |  | RL38\_MOUSE 60S ribosomal protein L38 OS=Mus musculus GN=Rpl38 PE=2 SV=3 | 1269.02 | 1233.27 | 1590.41 | 1097.98 |
| P16254 | 1 (1) | 50.39 | 0.02 | 3.50 |  | SRP14\_MOUSE Signal recognition particle 14 kDa protein OS=Mus musculus GN=Srp14 PE=1 SV=1 | 624.59 | 178.60 | 517.37 | 197.36 |
| P00920 | 1 (1) | 50.05 | 0.19 | 2.41 |  | CAH2\_MOUSE Carbonic anhydrase 2 OS=Mus musculus GN=Ca2 PE=1 SV=4 | 1601.55 | 664.62 | 1465.95 | 899.29 |
| P48678 | 1 (1) | 49.80 | 0.40 | 1.72 |  | LMNA\_MOUSE Prelamin-A/C OS=Mus musculus GN=Lmna PE=1 SV=2 | 426.81 | 248.12 | 293.29 | 273.27 |
| Q9CXS4 | 1 (1) | 49.51 | 0.74 | 1.23 |  | CENPV\_MOUSE Centromere protein V OS=Mus musculus GN=Cenpv PE=2 SV=2 | 237.18 | 224.66 | 208.13 | 193.53 |
| Q8R5C5 | 1 (1) | 49.45 | 0.02 | 6.96 |  | ACTY\_MOUSE Beta-centractin OS=Mus musculus GN=Actr1b PE=1 SV=1 | 292.47 | 42.05 | 207.41 | 59.69 |
| P26638 | 1 (1) | 49.45 | 0.37 | 1.47 |  | SYSC\_MOUSE Seryl-tRNA synthetase, cytoplasmic OS=Mus musculus GN=Sars PE=2 SV=3 | 1735.90 | 1291.25 | 1896.05 | 1466.38 |
| P16125 | 1 (1) | 49.45 | 4.24e-003 | 9.99 |  | LDHB\_MOUSE L-lactate dehydrogenase B chain OS=Mus musculus GN=Ldhb PE=1 SV=2 | 1048.71 | 104.97 | 757.74 | 257.37 |
| Q9Z1Q5 | 1 (1) | 49.29 | 0.13 | 2.03 |  | CLIC1\_MOUSE Chloride intracellular channel protein 1 OS=Mus musculus GN=Clic1 PE=1 SV=3 | 926.49 | 455.45 | 917.59 | 578.25 |
| P54071 | 1 (1) | 49.04 | 0.42 | 1.29 |  | IDHP\_MOUSE Isocitrate dehydrogenase [NADP], mitochondrial OS=Mus musculus GN=Idh2 PE=1 SV=3 | 453.25 | 352.36 | 404.91 | 367.12 |
| Q99020 | 1 (1) | 48.01 | 0.50 | 1.53 |  | ROAA\_MOUSE Heterogeneous nuclear ribonucleoprotein A/B OS=Mus musculus GN=Hnrnpab PE=1 SV=1 | 1366.86 | 974.80 | 1158.96 | 1486.80 |
| Q9CXI5 | 1 (1) | 47.97 | 0.20 | 1.78 |  | MANF\_MOUSE Mesencephalic astrocyte-derived neurotrophic factor OS=Mus musculus GN=Manf PE=1 SV=1 | 6296.17 | 3539.29 | 5743.15 | 4991.23 |
| Q61207 | 1 (0) | 47.96 | --- | --- |  | SAP\_MOUSE Sulfated glycoprotein 1 OS=Mus musculus GN=Psap PE=1 SV=2 | --- | --- | --- | --- |
| P63323 | 1 (1) | 47.93 | 0.73 | 1.17 |  | RS12\_MOUSE 40S ribosomal protein S12 OS=Mus musculus GN=Rps12 PE=1 SV=2 | 1100.57 | 1044.71 | 936.84 | 1020.56 |
| Q9CZD3 | 1 (1) | 47.76 | 0.08 | 3.45 |  | SYG\_MOUSE Glycyl-tRNA synthetase OS=Mus musculus GN=Gars PE=1 SV=1 | 635.24 | 184.07 | 611.72 | 409.08 |
| Q61753 | 1 (1) | 47.46 | 3.22e-003 | 2.33 |  | SERA\_MOUSE D-3-phosphoglycerate dehydrogenase OS=Mus musculus GN=Phgdh PE=1 SV=3 | 786.43 | 338.03 | 657.39 | 499.62 |
| Q8CHP8 | 1 (1) | 46.41 | 0.21 | 1.71 |  | PGP\_MOUSE Phosphoglycolate phosphatase OS=Mus musculus GN=Pgp PE=2 SV=1 | 901.65 | 614.10 | 910.16 | 532.63 |
| O88844 | 1 (1) | 46.39 | 0.13 | 3.93 |  | IDHC\_MOUSE Isocitrate dehydrogenase [NADP] cytoplasmic OS=Mus musculus GN=Idh1 PE=1 SV=1 | 110.78 | 55.82 | 67.56 | 28.21 |
| Q9QUI0 | 1 (1) | 46.16 | 0.86 | 1.18 |  | RHOA\_MOUSE Transforming protein RhoA OS=Mus musculus GN=Rhoa PE=1 SV=1 | 572.82 | 660.06 | 605.39 | 560.29 |
| Q8CIB5 | 1 (1) | 46.15 | 0.06 | 3.86 |  | FERM2\_MOUSE Fermitin family homolog 2 OS=Mus musculus GN=Fermt2 PE=1 SV=1 | 295.76 | 86.51 | 334.05 | 179.36 |
| Q99KK7 | 1 (1) | 46.10 | 0.01 | 3.54 |  | DPP3\_MOUSE Dipeptidyl peptidase 3 OS=Mus musculus GN=Dpp3 PE=2 SV=1 | 335.03 | 94.64 | 225.91 | 133.67 |
| Q99PT1 | 1 (1) | 45.59 | 0.40 | 1.40 |  | GDIR1\_MOUSE Rho GDP-dissociation inhibitor 1 OS=Mus musculus GN=Arhgdia PE=1 SV=3 | 1570.44 | 1564.56 | 1366.23 | 1125.33 |
| Q93092 | 1 (1) | 45.19 | 0.19 | 2.49 |  | TALDO\_MOUSE Transaldolase OS=Mus musculus GN=Taldo1 PE=1 SV=2 | 632.74 | 301.24 | 370.46 | 253.95 |
| Q9CY27 | 1 (1) | 45.07 | 0.47 | 1.64 |  | TECR\_MOUSE Trans-2,3-enoyl-CoA reductase OS=Mus musculus GN=Tecr PE=1 SV=1 | 440.60 | 269.44 | 423.63 | 398.74 |
| Q9CZ13 | 1 (1) | 44.86 | 4.64e-003 | 5.23 |  | QCR1\_MOUSE Cytochrome b-c1 complex subunit 1, mitochondrial OS=Mus musculus GN=Uqcrc1 PE=1 SV=1 | 1752.97 | 535.89 | 1350.70 | 335.47 |
| Q9DCX2 | 1 (1) | 43.99 | 0.10 | 1.93 |  | ATP5H\_MOUSE ATP synthase subunit d, mitochondrial OS=Mus musculus GN=Atp5h PE=1 SV=3 | 390.94 | 202.10 | 367.30 | 253.95 |
| Q9D1M7 | 1 (1) | 43.94 | 0.30 | 1.79 |  | FKB11\_MOUSE Peptidyl-prolyl cis-trans isomerase FKBP11 OS=Mus musculus GN=Fkbp11 PE=2 SV=1 | 516.76 | 288.28 | 443.88 | 331.83 |
| Q3UPH1 | 1 (1) | 43.77 | 0.54 | 2.21 |  | PRRC1\_MOUSE Protein PRRC1 OS=Mus musculus GN=Prrc1 PE=2 SV=1 | 263.81 | 119.50 | 241.94 | 214.44 |
| P19253 | 1 (1) | 43.74 | 0.63 | 1.33 |  | RL13A\_MOUSE 60S ribosomal protein L13a OS=Mus musculus GN=Rpl13a PE=1 SV=4 | 2348.49 | 1805.42 | 1788.91 | 1766.46 |
| P56391 | 1 (1) | 43.72 | 0.02 | 5.99 |  | CX6B1\_MOUSE Cytochrome c oxidase subunit 6B1 OS=Mus musculus GN=Cox6b1 PE=1 SV=2 | 1466.84 | 244.87 | 1152.02 | 622.45 |
| P42932 | 1 (0) | 43.62 | --- | --- |  | TCPQ\_MOUSE T-complex protein 1 subunit theta OS=Mus musculus GN=Cct8 PE=1 SV=3 | --- | --- | --- | --- |
| Q9R0Q3 | 1 (1) | 43.47 | 0.03 | 8.20 |  | TMED2\_MOUSE Transmembrane emp24 domain-containing protein 2 OS=Mus musculus GN=Tmed2 PE=1 SV=1 | 227.78 | 27.77 | 133.73 | 59.83 |
| Q9CQC9 | 1 (1) | 43.42 | 0.38 | 1.58 |  | SAR1B\_MOUSE GTP-binding protein SAR1b OS=Mus musculus GN=Sar1b PE=1 SV=1 | 1245.84 | 1152.35 | 1675.82 | 1817.88 |
| P26350 | 1 (1) | 43.16 | 0.43 | 1.47 |  | PTMA\_MOUSE Prothymosin alpha OS=Mus musculus GN=Ptma PE=1 SV=2 | 1589.14 | 1157.97 | 1310.99 | 1078.55 |
| P06151 | 1 (1) | 43.14 | 0.61 | 1.42 |  | LDHA\_MOUSE L-lactate dehydrogenase A chain OS=Mus musculus GN=Ldha PE=1 SV=3 | 641.57 | 669.54 | 715.04 | 503.47 |
| O35350 | 1 (1) | 43.03 | 0.13 | 2.05 |  | CAN1\_MOUSE Calpain-1 catalytic subunit OS=Mus musculus GN=Capn1 PE=2 SV=1 | 255.23 | 129.76 | 192.13 | 265.73 |
| P10639 | 1 (1) | 42.89 | 0.52 | 1.36 |  | THIO\_MOUSE Thioredoxin OS=Mus musculus GN=Txn PE=1 SV=3 | 2273.33 | 1828.15 | 2078.52 | 1673.44 |
| Q8VDJ3 | 1 (1) | 42.86 | 0.03 | 2.50 |  | VIGLN\_MOUSE Vigilin OS=Mus musculus GN=Hdlbp PE=1 SV=1 | 623.87 | 255.70 | 624.92 | 250.44 |
| P50431 | 1 (1) | 42.84 | 0.01 | 7.10 |  | GLYC\_MOUSE Serine hydroxymethyltransferase, cytosolic OS=Mus musculus GN=Shmt1 PE=1 SV=2 | 496.95 | 70.01 | 368.50 | 217.19 |
| Q9JLR1 | 1 (1) | 42.69 | 0.68 | 1.29 |  | S61A2\_MOUSE Protein transport protein Sec61 subunit alpha isoform 2 OS=Mus musculus GN=Sec61a2 PE=2 SV=3 | 1098.69 | 1236.69 | 955.78 | 1101.05 |
| P61514 | 1 (1) | 42.61 | 0.19 | 1.68 |  | RL37A\_MOUSE 60S ribosomal protein L37a OS=Mus musculus GN=Rpl37a PE=2 SV=2 | 1752.80 | 1155.32 | 1458.49 | 1045.46 |
| P49962 | 1 (1) | 41.45 | 0.29 | 1.70 |  | SRP09\_MOUSE Signal recognition particle 9 kDa protein OS=Mus musculus GN=Srp9 PE=1 SV=2 | 410.06 | 308.43 | 524.52 | 339.35 |
| Q9R099 | 1 (1) | 40.92 | 0.21 | 2.38 |  | TBL2\_MOUSE Transducin beta-like protein 2 OS=Mus musculus GN=Tbl2 PE=2 SV=1 | 278.73 | 125.17 | 298.24 | 128.37 |
| Q5SYD0 | 1 (1) | 40.79 | 0.03 | 5.44 |  | MYO1D\_MOUSE Myosin-Id OS=Mus musculus GN=Myo1d PE=1 SV=1 | 453.04 | 83.34 | 297.64 | 169.18 |
| Q3TDQ1 | 1 (1) | 40.52 | 0.72 | 1.32 |  | STT3B\_MOUSE Dolichyl-diphosphooligosaccharide--protein glycosyltransferase subunit STT3B OS=Mus musculus GN=Stt3b PE=1 SV=2 | 932.77 | 909.28 | 1198.33 | 1072.77 |
| P62862 | 1 (1) | 40.39 | 0.60 | 1.46 |  | RS30\_MOUSE 40S ribosomal protein S30 OS=Mus musculus GN=Fau PE=3 SV=1 | 666.15 | 641.80 | 491.03 | 714.81 |
| Q9CPY7 | 1 (1) | 39.82 | 0.80 | 1.31 |  | AMPL\_MOUSE Cytosol aminopeptidase OS=Mus musculus GN=Lap3 PE=1 SV=3 | 250.20 | 193.78 | 214.20 | 191.10 |
| P62827 | 1 (1) | 39.57 | 0.65 | 1.42 |  | RAN\_MOUSE GTP-binding nuclear protein Ran OS=Mus musculus GN=Ran PE=1 SV=3 | 863.39 | 946.65 | 664.35 | 886.06 |
| P61961 | 1 (1) | 39.37 | 0.45 | 1.43 |  | UFM1\_MOUSE Ubiquitin-fold modifier 1 OS=Mus musculus GN=Ufm1 PE=1 SV=1 | 3209.92 | 2248.50 | 2787.29 | 2498.22 |
| Q91VR2 | 1 (1) | 39.22 | 0.55 | 1.37 |  | ATPG\_MOUSE ATP synthase subunit gamma, mitochondrial OS=Mus musculus GN=Atp5c1 PE=1 SV=1 | 308.51 | 241.60 | 330.97 | 303.76 |
| P00405 | 1 (1) | 39.16 | 0.07 | 1.89 |  | COX2\_MOUSE Cytochrome c oxidase subunit 2 OS=Mus musculus GN=Mtco2 PE=1 SV=1 | 614.15 | 325.69 | 519.96 | 396.99 |
| P47758 | 1 (1) | 38.05 | 0.16 | 2.14 |  | SRPRB\_MOUSE Signal recognition particle receptor subunit beta OS=Mus musculus GN=Srprb PE=1 SV=1 | 296.21 | 138.74 | 239.42 | 261.89 |
| P47738 | 1 (1) | 38.05 | 0.20 | 1.68 |  | ALDH2\_MOUSE Aldehyde dehydrogenase, mitochondrial OS=Mus musculus GN=Aldh2 PE=1 SV=1 | 514.00 | 305.09 | 506.82 | 344.09 |
| Q62393 | 1 (1) | 37.43 | 0.35 | 1.90 |  | TPD52\_MOUSE Tumor protein D52 OS=Mus musculus GN=Tpd52 PE=1 SV=2 | 595.31 | 325.35 | 619.18 | 476.97 |
| Q60597 | 1 (1) | 37.39 | 0.22 | 5.21 |  | ODO1\_MOUSE 2-oxoglutarate dehydrogenase, mitochondrial OS=Mus musculus GN=Ogdh PE=1 SV=3 | 114.97 | 22.06 | 29.27 | 51.99 |
| Q91W90 | 1 (1) | 37.31 | 0.10 | 2.60 |  | TXND5\_MOUSE Thioredoxin domain-containing protein 5 OS=Mus musculus GN=Txndc5 PE=1 SV=2 | 479.42 | 184.28 | 388.65 | 339.02 |
| Q63805 | 1 (1) | 37.16 | 0.12 | 4.75 |  | A1AG3\_MOUSE Alpha-1-acid glycoprotein 3 OS=Mus musculus GN=Orm3 PE=2 SV=1 | 209.10 | 146.89 | 338.03 | 71.21 |
| Q9JII6 | 1 (1) | 36.92 | 0.50 | 1.62 |  | AK1A1\_MOUSE Alcohol dehydrogenase [NADP+] OS=Mus musculus GN=Akr1a1 PE=1 SV=3 | 333.07 | 205.07 | 245.30 | 284.30 |
| Q9Z0J0 | 1 (1) | 36.82 | 0.29 | 1.72 |  | NPC2\_MOUSE Epididymal secretory protein E1 OS=Mus musculus GN=Npc2 PE=2 SV=1 | 927.16 | 595.02 | 671.64 | 540.51 |
| Q6ZWV3 | 1 (1) | 36.80 | 0.23 | 1.61 |  | RL10\_MOUSE 60S ribosomal protein L10 OS=Mus musculus GN=Rpl10 PE=2 SV=3 | 548.59 | 341.12 | 430.03 | 363.40 |
| Q9CPT4 | 1 (1) | 36.53 | 0.45 | 1.18 |  | CS010\_MOUSE UPF0556 protein C19orf10 homolog OS=Mus musculus GN=D17Wsu104e PE=2 SV=1 | 461.10 | 404.16 | 444.52 | 389.24 |
| Q9CQS8 | 1 (1) | 36.22 | 0.96 | 1.09 |  | SC61B\_MOUSE Protein transport protein Sec61 subunit beta OS=Mus musculus GN=Sec61b PE=1 SV=3 | 2462.58 | 2359.34 | 2269.41 | 2284.75 |
| Q64442 | 1 (1) | 35.67 | 0.48 | 1.64 |  | DHSO\_MOUSE Sorbitol dehydrogenase OS=Mus musculus GN=Sord PE=1 SV=3 | 362.66 | 223.02 | 303.57 | 221.55 |
| Q8BMF4 | 1 (1) | 35.52 | 0.03 | 9.21 |  | ODP2\_MOUSE Dihydrolipoyllysine-residue acetyltransferase component of pyruvate dehydrogenase complex, mitochondrial OS=Mus musculus GN=Dlat PE=1 SV=2 | 399.86 | 43.42 | 287.02 | 131.01 |
| P41105 | 1 (1) | 35.37 | 0.83 | 1.28 |  | RL28\_MOUSE 60S ribosomal protein L28 OS=Mus musculus GN=Rpl28 PE=1 SV=2 | 1479.09 | 1241.80 | 1425.99 | 1584.84 |
| P60335 | 1 (1) | 35.15 | 0.37 | 1.61 |  | PCBP1\_MOUSE Poly(rC)-binding protein 1 OS=Mus musculus GN=Pcbp1 PE=1 SV=1 | 878.50 | 544.97 | 863.97 | 676.61 |
| P24527 | 1 (1) | 35.05 | 0.52 | 1.58 |  | LKHA4\_MOUSE Leukotriene A-4 hydrolase OS=Mus musculus GN=Lta4h PE=1 SV=3 | 311.72 | 197.30 | 248.25 | 239.45 |
| O88487 | 1 (1) | 34.98 | 0.66 | 1.31 |  | DC1I2\_MOUSE Cytoplasmic dynein 1 intermediate chain 2 OS=Mus musculus GN=Dync1i2 PE=2 SV=1 | 1551.34 | 2036.63 | 2022.79 | 1963.08 |
| Q9D051 | 1 (1) | 34.75 | 0.23 | 2.05 |  | ODPB\_MOUSE Pyruvate dehydrogenase E1 component subunit beta, mitochondrial OS=Mus musculus GN=Pdhb PE=1 SV=1 | 437.12 | 213.19 | 294.86 | 269.39 |
| Q9EPC1 | 1 (1) | 34.01 | 0.02 | 7.15 |  | PARVA\_MOUSE Alpha-parvin OS=Mus musculus GN=Parva PE=1 SV=1 | 349.65 | 48.91 | 340.42 | 92.92 |
| Q9R1P0 | 1 (1) | 33.71 | 0.22 | 2.03 |  | PSA4\_MOUSE Proteasome subunit alpha type-4 OS=Mus musculus GN=Psma4 PE=1 SV=1 | 691.73 | 355.12 | 720.70 | 464.10 |
| Q9D662 | 1 (1) | 33.51 | 0.02 | 6.65 |  | SC23B\_MOUSE Protein transport protein Sec23B OS=Mus musculus GN=Sec23b PE=2 SV=1 | 1251.38 | 188.30 | 1096.09 | 657.50 |
| Q91YL7 | 1 (1) | 33.43 | 0.13 | 7.36 |  | PG2IP\_MOUSE PGAP2-interacting protein OS=Mus musculus GN=Cwh43 PE=1 SV=1 | 418.37 | 56.82 | 309.91 | 172.51 |
| P23591 | 1 (0) | 33.42 | --- | --- |  | FCL\_MOUSE GDP-L-fucose synthase OS=Mus musculus GN=Tsta3 PE=2 SV=3 | --- | --- | --- | --- |
| Q3UPL0 | 1 (1) | 32.23 | 0.22 | 1.58 |  | SC31A\_MOUSE Protein transport protein Sec31A OS=Mus musculus GN=Sec31a PE=1 SV=2 | 658.60 | 423.33 | 668.85 | 482.61 |
| P63276 | 1 (1) | 32.10 | 0.03 | 13.21 |  | RS17\_MOUSE 40S ribosomal protein S17 OS=Mus musculus GN=Rps17 PE=1 SV=2 | 1157.71 | 87.65 | 790.21 | 223.07 |
| P01868 | 1 (1) | 31.73 | 0.61 | 1.43 |  | IGHG1\_MOUSE Ig gamma-1 chain C region secreted form OS=Mus musculus GN=Ighg1 PE=1 SV=1 | 444.07 | 473.72 | 331.39 | 351.43 |
| P15501 | 1 (1) | 31.73 | 0.62 | 118.72 |  | SPBP\_MOUSE Prostatic spermine-binding protein OS=Mus musculus GN=Sbp PE=2 SV=1 | 12.87 | 0.54 | 64.32 | 6.26 |
| P80314 | 1 (1) | 31.39 | 0.32 | 4.33 |  | TCPB\_MOUSE T-complex protein 1 subunit beta OS=Mus musculus GN=Cct2 PE=1 SV=4 | 145.26 | 33.53 | 138.81 | 41.84 |
| Q02053 | 1 (1) | 30.78 | 0.01 | 4.16 |  | UBA1\_MOUSE Ubiquitin-like modifier-activating enzyme 1 OS=Mus musculus GN=Uba1 PE=1 SV=1 | 491.10 | 117.96 | 371.78 | 242.05 |
| Q9DC29 | 1 (0) | 30.26 | --- | --- |  | ABCB6\_MOUSE ATP-binding cassette sub-family B member 6, mitochondrial OS=Mus musculus GN=Abcb6 PE=1 SV=1 | --- | --- | --- | --- |
| Q8BGY2 | 1 (1) | 29.84 | 0.23 | 2.49 |  | IF5A2\_MOUSE Eukaryotic translation initiation factor 5A-2 OS=Mus musculus GN=Eif5a2 PE=2 SV=3 | 191.24 | 92.34 | 230.10 | 205.38 |
| Q1W617 | 1 (1) | 29.63 | 0.85 | 17.47 |  | SHRM4\_MOUSE Protein Shroom4 OS=Mus musculus GN=Shroom4 PE=1 SV=1 | 529.75 | 30.33 | 78.18 | 144.20 |
| Q9Z172 | 1 (1) | 29.63 | 0.83 | 1.94 |  | SUMO3\_MOUSE Small ubiquitin-related modifier 3 OS=Mus musculus GN=Sumo3 PE=2 SV=1 | 608.53 | 770.76 | 556.34 | 1076.89 |
| P62320 | 1 (1) | 29.45 | 0.18 | 2.78 |  | SMD3\_MOUSE Small nuclear ribonucleoprotein Sm D3 OS=Mus musculus GN=Snrpd3 PE=1 SV=1 | 275.25 | 99.18 | 231.15 | 172.54 |
| Q8BWY3 | 1 (1) | 29.42 | 0.05 | 4.37 |  | ERF1\_MOUSE Eukaryotic peptide chain release factor subunit 1 OS=Mus musculus GN=Etf1 PE=1 SV=4 | 490.81 | 164.40 | 408.45 | 112.21 |
| Q80W14 | 1 (1) | 28.97 | 0.24 | 1.65 |  | PR40B\_MOUSE Pre-mRNA-processing factor 40 homolog B OS=Mus musculus GN=Prpf40b PE=2 SV=2 | 9981.16 | 1.33e+004 | 1.11e+004 | 1.64e+004 |
| P31230 | 1 (1) | 28.96 | 0.12 | 1.60 |  | AIMP1\_MOUSE Aminoacyl tRNA synthase complex-interacting multifunctional protein 1 OS=Mus musculus GN=Aimp1 PE=1 SV=2 | 446.72 | 279.37 | 400.79 | 308.97 |
| Q9D309 | 1 (1) | 28.47 | 0.99 | 1.65 |  | FAM3B\_MOUSE Protein FAM3B OS=Mus musculus GN=Fam3b PE=1 SV=1 | 224.83 | 144.42 | 138.80 | 228.65 |
| Q9WVA4 | 1 (1) | 28.28 | 0.22 | 2.71 |  | TAGL2\_MOUSE Transgelin-2 OS=Mus musculus GN=Tagln2 PE=1 SV=4 | 1242.99 | 459.23 | 900.88 | 710.64 |
| Q9R1P3 | 1 (1) | 28.19 | 6.08e-003 | 4.84 |  | PSB2\_MOUSE Proteasome subunit beta type-2 OS=Mus musculus GN=Psmb2 PE=1 SV=1 | 414.85 | 85.70 | 378.80 | 227.76 |
| P09055 | 1 (1) | 28.19 | 0.36 | 1.37 |  | ITB1\_MOUSE Integrin beta-1 OS=Mus musculus GN=Itgb1 PE=1 SV=1 | 1498.77 | 1592.25 | 1578.60 | 1160.94 |
| Q5RL79 | 1 (1) | 28.16 | 0.13 | 1.55 |  | KTAP2\_MOUSE Keratinocyte-associated protein 2 OS=Mus musculus GN=Krtcap2 PE=2 SV=2 | 306.19 | 197.99 | 258.31 | 198.54 |
| Q91V64 | 1 (1) | 27.82 | 0.26 | 2.81 |  | ISOC1\_MOUSE Isochorismatase domain-containing protein 1 OS=Mus musculus GN=Isoc1 PE=2 SV=1 | 482.53 | 171.98 | 443.14 | 325.85 |
| Q91ZA3 | 1 (1) | 27.12 | 0.08 | 2.41 |  | PCCA\_MOUSE Propionyl-CoA carboxylase alpha chain, mitochondrial OS=Mus musculus GN=Pcca PE=2 SV=2 | 1882.63 | 4543.24 | 2555.36 | 4093.79 |
| Q9D958 | 1 (1) | 27.03 | 0.26 | 2.89 |  | SPCS1\_MOUSE Signal peptidase complex subunit 1 OS=Mus musculus GN=Spcs1 PE=2 SV=2 | 691.32 | 267.15 | 523.46 | 772.37 |
| Q9CR67 | 1 (1) | 26.95 | 0.61 | 2.54 |  | TMM33\_MOUSE Transmembrane protein 33 OS=Mus musculus GN=Tmem33 PE=2 SV=1 | 90.64 | 35.73 | 63.64 | 70.75 |
| Q8BFR5 | 1 (1) | 26.80 | 0.37 | 1.64 |  | EFTU\_MOUSE Elongation factor Tu, mitochondrial OS=Mus musculus GN=Tufm PE=1 SV=1 | 581.04 | 436.76 | 714.51 | 508.95 |
| Q62210 | 1 (1) | 26.64 | 0.47 | 1.37 |  | BIRC2\_MOUSE Baculoviral IAP repeat-containing protein 2 OS=Mus musculus GN=Birc2 PE=1 SV=1 | 3968.10 | 4176.47 | 5441.91 | 5096.20 |
| P80313 | 1 (1) | 26.53 | 0.11 | 2.22 |  | TCPH\_MOUSE T-complex protein 1 subunit eta OS=Mus musculus GN=Cct7 PE=1 SV=1 | 330.12 | 148.81 | 309.02 | 248.96 |
| Q8JZQ9 | 1 (1) | 26.46 | 0.10 | 1.75 |  | EIF3B\_MOUSE Eukaryotic translation initiation factor 3 subunit B OS=Mus musculus GN=Eif3b PE=1 SV=1 | 136.33 | 78.06 | 130.69 | 88.46 |
| Q7TN08 | 1 (1) | 26.29 | 0.26 | 2.53 |  | DACT2\_MOUSE Dapper homolog 2 OS=Mus musculus GN=Dact2 PE=2 SV=2 | 1320.75 | 2616.96 | 2788.10 | 3341.89 |
| Q9DBP5 | 1 (1) | 26.10 | 0.09 | 2.77 |  | KCY\_MOUSE UMP-CMP kinase OS=Mus musculus GN=Cmpk1 PE=1 SV=1 | 456.40 | 164.61 | 390.39 | 171.52 |
| Q9D1R9 | 1 (1) | 25.84 | 0.78 | 1.26 |  | RL34\_MOUSE 60S ribosomal protein L34 OS=Mus musculus GN=Rpl34 PE=3 SV=2 | 881.45 | 841.52 | 730.70 | 698.71 |
| Q80XI3 | 1 (1) | 25.73 | 0.39 | 1.58 |  | IF4G3\_MOUSE Eukaryotic translation initiation factor 4 gamma 3 OS=Mus musculus GN=Eif4g3 PE=1 SV=2 | 716.06 | 1131.96 | 816.75 | 1040.90 |
| Q8BMS1 | 1 (1) | 25.03 | 0.01 | 10.24 |  | ECHA\_MOUSE Trifunctional enzyme subunit alpha, mitochondrial OS=Mus musculus GN=Hadha PE=1 SV=1 | 597.13 | 58.31 | 382.76 | 167.62 |

  

| Tags | |
| --- | --- |
|  | Anova p-value ≤ 0.05 |
|  | Anova p-value ≤ 0.01 |

  

##

## O08638

MYH11\_MOUSE
Myosin-11 OS=Mus musculus GN=Myh11 PE=1 SV=1  
52 peptides

  

| Sequence | Peptide Ion | Score | Hits | Mass | Charge | Tags | Conflicts | Modifications | In quantitation | Average Normalised Abundances | | | |
| --- | --- | --- | --- | --- | --- | --- | --- | --- | --- | --- | --- | --- | --- |
|  |  |  |  |  |  |  |  |  |  | 2m2f | 4m2f | 2m4f | 4m4f |
| ALEEALEAK | 2123 | 48.46 | 3 | 972.5034 | 2 |  | 1 |  | no | 2591.10 | 1893.80 | 2436.67 | 2415.66 |
| ALELDPNLYR | 1691 | 45.85 | 3 | 1202.6272 | 2 |  | 1 |  | no | 3611.49 | 2436.77 | 3308.21 | 2950.42 |
| ATLQAEQLSNELATER | 5239 | 61.01 | 3 | 1772.8935 | 3 |  | 0 |  | no | 1602.43 | 219.02 | 1174.81 | 791.66 |
| DEIFATSK | 2364 | 38.41 | 1 | 909.4364 | 2 |  | 0 |  | no | 3753.04 | 2862.35 | 3390.59 | 3092.90 |
| DLGEELEALK | 3622 | 44.74 | 3 | 1115.5715 | 2 |  | 1 |  | no | 1520.67 | 496.17 | 1599.84 | 914.86 |
| DVASLGSQLQDTQELLQEETR | 2730 | 88.38 | 3 | 2359.1490 | 3 |  | 0 |  | yes | 5827.35 | 1451.39 | 6579.93 | 2169.99 |
| DVASLGSQLQDTQELLQEETR | 16003 | --- | --- | 2359.1564 | 2 |  | 0 |  | yes | 641.05 | 420.97 | 610.48 | 567.89 |
| EDQSILCTGESGAGK | 2805 | 68.63 | 3 | 1550.6829 | 2 |  | 2 |  | no | 4611.77 | 2706.44 | 4055.32 | 4123.34 |
| EELAEELASSLSGR | 13212 | 30.42 | 3 | 1489.7255 | 3 |  | 0 |  | no | 170.10 | 13.62 | 131.55 | 39.92 |
| ELDEATESNEAMGR | 4829 | 90.85 | 3 | 1550.6510 | 2 |  | 0 |  | no | 1911.61 | 475.62 | 1240.56 | 1253.57 |
| ELEGHISDLQEDLDSER | 3485 | 58.03 | 2 | 1983.8952 | 3 |  | 0 |  | no | 3441.20 | 1175.39 | 2808.19 | 1721.23 |
| EMEGLSQQYEEK | 4358 | 72.80 | 3 | 1469.6339 | 2 |  | 0 |  | no | 1975.49 | 756.92 | 1475.14 | 1304.47 |
| EQADFAIEALAK | 1716 | 65.04 | 3 | 1304.6612 | 2 |  | 1 |  | yes | 6797.83 | 6257.95 | 5448.91 | 5039.11 |
| EVLLQVEDER | 3282 | 56.83 | 3 | 1228.6299 | 2 |  | 0 |  | no | 2455.39 | 559.42 | 1675.95 | 1239.30 |
| FDQLLAEEK | 4779 | 58.34 | 3 | 1091.5412 | 2 |  | 2 |  | no | 836.23 | 356.40 | 579.35 | 531.05 |
| FVADLWK | 2338 | 48.92 | 3 | 877.4689 | 2 |  | 0 |  | no | 1398.22 | 737.65 | 1439.65 | 1192.74 |
| GDEVVVELVENGK | 4538 | 31.11 | 2 | 1385.7071 | 2 |  | 1 |  | no | 1844.52 | 2023.86 | 2411.09 | 1912.56 |
| HTQAVEELTEQLEQFK | 3267 | 63.68 | 2 | 1928.9483 | 3 |  | 0 |  | no | 1881.42 | 670.08 | 1668.68 | 817.83 |
| HVSTLNIQLSDSK | 4328 | 57.42 | 3 | 1440.7596 | 3 |  | 0 |  | no | 1721.16 | 2039.97 | 2053.88 | 2034.94 |
| IAQLEEELEEEQGNMEAMSDR | 8696 | 25.78 | 3 | 2450.0567 | 3 |  | 0 |  | no | 776.16 | 175.02 | 1208.92 | 379.38 |
| IAQLEEQVEQEAR | 2770 | 122.66 | 3 | 1541.7686 | 2 |  | 0 |  | no | 4065.64 | 1282.69 | 3466.62 | 2351.59 |
| IVFQEFR | 1970 | 39.97 | 3 | 937.5011 | 2 |  | 1 |  | no | 1901.47 | 623.16 | 1827.83 | 1233.28 |
| KFDQLLAEEK | 9347 | 42.12 | 3 | 1219.6444 | 3 |  | 2 |  | no | 225.59 | 33.67 | 268.76 | 131.26 |
| KQELEEILHEMEAR | 9003 | 49.30 | 3 | 1753.8653 | 3 |  | 0 |  | no | 470.59 | 58.14 | 380.29 | 165.87 |
| LDAFLVLEQLR | 12289 | --- | --- | 1315.7493 | 3 |  | 0 |  | no | 274.24 | 37.51 | 380.76 | 51.14 |
| LDAFLVLEQLR | 3600 | 63.91 | 3 | 1315.7498 | 2 |  | 0 |  | no | 4715.09 | 838.73 | 5778.33 | 1417.58 |
| LDEEIAQK | 2767 | 50.29 | 3 | 944.4807 | 2 |  | 0 |  | no | 1473.81 | 539.16 | 1231.79 | 1072.83 |
| LEDDILVMDDQNSK | 13302 | 44.00 | 2 | 1633.7493 | 2 |  | 0 |  | no | 600.12 | 134.91 | 362.10 | 276.92 |
| LEVNMQALK | 3728 | 62.41 | 3 | 1044.5623 | 2 |  | 0 |  | no | 1060.42 | 324.44 | 798.56 | 575.10 |
| LEVQLQDLQSK | 4939 | 84.51 | 3 | 1299.6950 | 2 |  | 0 |  | no | 1944.50 | 1295.90 | 2266.34 | 1758.65 |
| LKEVLLQVEDER | 10674 | 42.82 | 1 | 1469.8087 | 3 |  | 0 |  | no | 171.43 | 29.83 | 199.60 | 57.32 |
| LQDFASTIEVMEEGK | 11628 | 45.42 | 3 | 1695.8016 | 3 |  | 0 |  | no | 380.57 | 36.35 | 321.85 | 145.22 |
| LQDFASTIEVMEEGK | 4920 | 121.90 | 3 | 1695.8024 | 2 |  | 0 |  | no | 2211.55 | 412.72 | 2538.32 | 928.63 |
| LQEVEGAVK | 2207 | 58.48 | 3 | 971.5274 | 2 |  | 0 |  | no | 2231.49 | 509.94 | 1589.74 | 1295.39 |
| LQNEVESVTGMLNEAEGK | 6105 | 78.38 | 3 | 1946.9165 | 2 |  | 0 |  | no | 1375.36 | 666.49 | 1678.72 | 1217.79 |
| LQQELDDLVVDLDNQR | 3329 | 55.04 | 3 | 1911.9574 | 3 |  | 0 |  | yes | 5699.87 | 2829.77 | 5653.68 | 3366.00 |
| LQQELDDLVVDLDNQR | 10684 | 71.09 | 1 | 1911.9548 | 2 |  | 0 |  | no | 5801.96 | 2693.75 | 4687.21 | 1519.93 |
| MAQQMLDLEEQLEEEEAAR | 9380 | 55.88 | 3 | 2262.0161 | 3 |  | 0 |  | no | 874.39 | 150.55 | 812.88 | 474.48 |
| MTESSLPSASK | 3098 | 57.25 | 3 | 1136.5377 | 2 |  | 0 |  | no | 2334.12 | 1020.21 | 2065.70 | 1701.78 |
| NFMNSPMAQADWVAK | 9054 | 49.56 | 3 | 1708.7694 | 2 |  | 0 |  | no | 738.82 | 228.27 | 662.33 | 367.60 |
| NMDPLNDNVTSLLNASSDK | 6668 | 65.95 | 3 | 2046.9494 | 2 |  | 0 |  | no | 2943.04 | 1636.35 | 2825.59 | 2139.64 |
| NMDPLNDNVTSLLNASSDK | 10392 | --- | --- | 2046.9541 | 3 |  | 0 |  | no | 641.12 | 156.25 | 404.55 | 351.78 |
| NTDQASMPDNTAAQK | 6747 | 80.30 | 3 | 1590.6923 | 2 |  | 1 |  | no | 1604.43 | 648.84 | 1575.83 | 951.88 |
| NWQWWR | 7475 | 29.88 | 3 | 974.4509 | 2 |  | 1 |  | no | 259.23 | 35.40 | 269.30 | 207.07 |
| QLLQANPILEAFGNAK | 10097 | 69.31 | 3 | 1725.9414 | 2 |  | 1 |  | no | 1456.18 | 102.49 | 1479.00 | 375.24 |
| SHEAQVQEMR | 12725 | 42.14 | 3 | 1213.5513 | 3 |  | 0 |  | no | 111.25 | 7.49 | 95.00 | 43.41 |
| SMLQDR | 10607 | 30.02 | 1 | 748.3539 | 2 |  | 0 |  | no | 177.51 | 30.01 | 105.67 | 69.52 |
| TEFSIIHYAGK | 7044 | 31.29 | 3 | 1264.6449 | 3 |  | 0 |  | no | 214.22 | 25.44 | 214.43 | 129.62 |
| TGVLAHLEEER | 2979 | 52.89 | 3 | 1252.6409 | 3 |  | 0 |  | no | 797.26 | 174.00 | 804.82 | 444.03 |
| VCHLVGINVTDFTR | 4376 | 32.95 | 3 | 1629.8281 | 3 |  | 0 |  | no | 932.63 | 186.27 | 1455.25 | 610.73 |
| VCHLVGINVTDFTR | 17497 | 27.41 | 1 | 1629.8316 | 2 |  | 0 |  | no | 163.24 | 9.61 | 195.41 | 29.87 |
| VDYNASAWLTK | 2126 | 58.39 | 3 | 1266.6223 | 2 |  | 0 |  | no | 3332.18 | 1300.11 | 3213.42 | 2802.25 |
| VEDMAELTCLNEASVLHNLR | 5112 | 54.48 | 3 | 2313.1092 | 3 |  | 0 |  | no | 2685.38 | 862.41 | 3151.95 | 1235.57 |
| VIQYLAVVASSHK | 5217 | 43.12 | 3 | 1413.7970 | 3 |  | 0 |  | no | 463.54 | 96.88 | 769.07 | 376.61 |
| VIQYLAVVASSHK | 14043 | --- | --- | 1413.7967 | 2 |  | 0 |  | no | 237.46 | 18.69 | 228.52 | 109.09 |
| VKPLLQVTR | 10591 | --- | --- | 1052.6704 | 2 |  | 1 |  | no | 213.57 | 28.00 | 175.75 | 106.15 |
| VKPLLQVTR | 5102 | 41.43 | 1 | 1052.6709 | 3 |  | 1 |  | no | 368.23 | 347.80 | 516.90 | 464.98 |
| VTVGKDDIQK | 9231 | 28.31 | 3 | 1101.6036 | 3 |  | 0 |  | no | 151.70 | 33.19 | 213.76 | 177.43 |
| VVSSVLQLGNIVFK | 5123 | 61.37 | 3 | 1501.8862 | 2 |  | 0 |  | no | 3485.65 | 475.95 | 3029.73 | 1086.81 |
| VVSSVLQLGNIVFK | 13338 | --- | --- | 1501.8860 | 3 |  | 0 |  | no | 304.30 | 17.76 | 243.99 | 69.56 |
| VVSSVLQLGNIVFKK | 13543 | --- | --- | 1629.9820 | 2 |  | 0 |  | no | 299.50 | 29.50 | 372.20 | 162.20 |
| VVSSVLQLGNIVFKK | 4964 | 27.31 | 3 | 1629.9945 | 3 |  | 0 |  | no | 1448.87 | 1200.17 | 1711.87 | 1424.04 |

  

| Tags | |
| --- | --- |

  

##

## Q6WIZ7

Q6WIZ7\_MOUSE
PSv-2 OS=Mus musculus GN=Svs1 PE=2 SV=1  
40 peptides

  

| Sequence | Peptide Ion | Score | Hits | Mass | Charge | Tags | Conflicts | Modifications | In quantitation | Average Normalised Abundances | | | |
| --- | --- | --- | --- | --- | --- | --- | --- | --- | --- | --- | --- | --- | --- |
|  |  |  |  |  |  |  |  |  |  | 2m2f | 4m2f | 2m4f | 4m4f |
| AKFPMPISK | 487 | 51.06 | 3 | 1017.5677 | 2 |  | 0 |  | no | 1.91e+004 | 2.42e+004 | 1.64e+004 | 2.17e+004 |
| ALCIFEMPLR | 3209 | 37.17 | 1 | 1248.6350 | 3 |  | 0 |  | no | 889.28 | 1718.24 | 1421.13 | 1622.78 |
| ALCIFEMPLR | 62 | 46.15 | 3 | 1248.6354 | 2 |  | 0 |  | no | 2.19e+005 | 4.16e+005 | 3.08e+005 | 3.61e+005 |
| ALCIFEMPLR | 1479 | 51.75 | 3 | 1264.6318 | 2 |  | 0 | [7] Oxidation (M) | no | 4787.25 | 1.50e+004 | 8576.99 | 1.61e+004 |
| ALCIFEMPLR | 11873 | 39.29 | 3 | 1264.6303 | 2 |  | 0 | [7] Oxidation (M) | no | 284.24 | 359.80 | 186.10 | 241.31 |
| EFPMPISK | 81 | 33.09 | 3 | 947.4781 | 2 |  | 0 |  | no | 7.30e+004 | 1.22e+005 | 8.25e+004 | 1.28e+005 |
| ELELQPSTLLTLAK | 19 | 83.78 | 3 | 1554.8858 | 2 |  | 0 |  | yes | 1.46e+006 | 1.96e+006 | 1.96e+006 | 1.96e+006 |
| ELELQPSTLLTLAK | 384 | 44.86 | 3 | 1554.8860 | 3 |  | 0 |  | yes | 3.26e+004 | 4.21e+004 | 4.60e+004 | 4.11e+004 |
| ELELQPSTLLTLAK | 5881 | 36.07 | 3 | 1554.8830 | 2 |  | 0 |  | no | 1607.49 | 1485.63 | 1683.42 | 1629.98 |
| FPIPISK | 44 | 44.99 | 3 | 800.4793 | 2 |  | 0 |  | no | 2.88e+005 | 4.25e+005 | 3.34e+005 | 5.03e+005 |
| FPMPISK | 117 | 40.58 | 3 | 818.4359 | 2 |  | 0 |  | no | 1.15e+005 | 1.82e+005 | 1.18e+005 | 1.79e+005 |
| FYNSPEELAQK | 1894 | 54.84 | 3 | 1324.6299 | 3 |  | 0 |  | yes | 2948.96 | 5278.17 | 3476.91 | 5508.42 |
| FYNSPEELAQK | 22 | 66.50 | 3 | 1324.6293 | 2 |  | 0 |  | yes | 6.89e+005 | 1.12e+006 | 7.01e+005 | 1.02e+006 |
| GAMLPLR | 157 | 41.91 | 3 | 756.4319 | 2 |  | 0 |  | no | 3.32e+004 | 5.55e+004 | 3.38e+004 | 5.29e+004 |
| GGPQIAQPHVPHYR | 806 | --- | --- | 1555.8013 | 4 |  | 0 |  | no | 7861.36 | 5195.84 | 8106.46 | 5239.52 |
| GGPQIAQPHVPHYR | 1120 | 29.99 | 3 | 1555.7996 | 3 |  | 0 |  | no | 8480.18 | 4898.33 | 8186.05 | 5519.99 |
| GGPQVAQPYR | 48 | 73.10 | 3 | 1071.5456 | 2 |  | 0 |  | no | 2.05e+005 | 3.08e+005 | 2.56e+005 | 2.99e+005 |
| GGPQVVQPDPK | 67 | 64.26 | 3 | 1120.5871 | 2 |  | 0 |  | no | 1.63e+005 | 2.50e+005 | 1.85e+005 | 2.21e+005 |
| GGPQVVQPDPK | 6358 | --- | --- | 1120.5877 | 3 |  | 0 |  | no | 353.61 | 374.02 | 276.94 | 239.49 |
| GGQQLSWASRPMSK | 6555 | 50.78 | 3 | 1531.7549 | 3 |  | 0 |  | no | 656.16 | 819.33 | 818.13 | 1065.14 |
| HNTVLYGDWNFFFK | 1338 | 56.04 | 3 | 1786.8458 | 3 |  | 0 |  | no | 1.37e+004 | 1.22e+004 | 1.56e+004 | 1.20e+004 |
| HNTVLYGDWNFFFK | 5738 | 39.37 | 3 | 1786.8468 | 2 |  | 0 |  | no | 3467.00 | 2499.44 | 3773.40 | 3009.15 |
| HSWFLLQR | 628 | 33.49 | 3 | 1085.5774 | 3 |  | 0 |  | no | 4720.83 | 5241.57 | 4510.37 | 6749.26 |
| HSWFLLQR | 94 | 41.77 | 3 | 1085.5766 | 2 |  | 0 |  | no | 9.16e+004 | 1.09e+005 | 8.53e+004 | 1.33e+005 |
| IAYEVGVQEVMALYR | 1089 | 59.50 | 3 | 1739.8906 | 3 |  | 0 |  | no | 1.46e+004 | 1.52e+004 | 1.15e+004 | 5284.34 |
| IAYEVGVQEVMALYR | 709 | 79.41 | 3 | 1739.8918 | 2 |  | 0 |  | no | 4.47e+004 | 4.59e+004 | 3.59e+004 | 1.74e+004 |
| IDLDVAGTK | 29 | 61.41 | 3 | 930.5016 | 2 |  | 0 |  | no | 2.08e+005 | 3.44e+005 | 2.46e+005 | 3.49e+005 |
| KYEVLAFLDK | 8560 | --- | --- | 1224.6681 | 3 |  | 0 |  | no | 167.00 | 279.49 | 209.01 | 272.98 |
| KYEVLAFLDK | 95 | 73.98 | 3 | 1224.6748 | 2 |  | 0 |  | no | 1.25e+005 | 1.46e+005 | 1.29e+005 | 2.01e+005 |
| KYEVLAFLDK | 145 | 42.39 | 3 | 1224.6747 | 3 |  | 0 |  | no | 3.74e+004 | 4.44e+004 | 3.90e+004 | 6.23e+004 |
| KYEVLAFLDK | 7587 | 39.89 | 3 | 1224.6741 | 2 |  | 0 |  | no | 265.33 | 322.30 | 438.59 | 442.19 |
| LENIMDPWSQQVKPILDK | 2540 | 47.21 | 3 | 2153.1245 | 2 |  | 0 |  | no | 6898.71 | 5965.98 | 7239.48 | 6635.74 |
| LENIMDPWSQQVKPILDK | 12362 | --- | --- | 2153.1172 | 4 |  | 0 |  | no | 245.30 | 195.84 | 251.87 | 167.45 |
| LENIMDPWSQQVKPILDK | 127 | 44.44 | 3 | 2153.1186 | 3 |  | 0 |  | no | 1.70e+005 | 1.71e+005 | 1.63e+005 | 1.62e+005 |
| LENIMDPWSQQVKPILDK | 2028 | 38.57 | 3 | 2169.1123 | 3 |  | 0 | [5] Oxidation (M) | no | 8340.18 | 9098.05 | 9196.45 | 7256.43 |
| LHSSYGLQLFNVHFR | 1417 | 44.85 | 3 | 1816.9345 | 4 |  | 0 |  | no | 3984.70 | 4195.40 | 5330.79 | 4981.71 |
| LHSSYGLQLFNVHFR | 1172 | 56.01 | 3 | 1816.9363 | 3 |  | 0 |  | no | 7708.37 | 8004.99 | 9972.56 | 9639.74 |
| LSGPMLVLR | 60 | 76.19 | 2 | 984.5786 | 2 |  | 0 |  | no | 1.14e+005 | 2.09e+005 | 1.17e+005 | 1.84e+005 |
| NSVFLIEMLMPK | 8054 | 55.46 | 3 | 1420.7448 | 3 |  | 0 |  | no | 1443.79 | 1256.29 | 1625.37 | 1101.04 |
| NSVFLIEMLMPK | 206 | 115.18 | 3 | 1420.7454 | 2 |  | 0 |  | no | 3.12e+005 | 2.69e+005 | 3.65e+005 | 2.98e+005 |
| NSVFLIEMLMPK | 1223 | 69.03 | 1 | 1436.7406 | 2 |  | 1 | [8] Oxidation (M) | no | 3.30e+004 | 4.34e+004 | 4.58e+004 | 3.87e+004 |
| NSVFLIEMLMPK | 13960 | --- | --- | 1436.7413 | 3 |  | 1 | [8] Oxidation (M) | no | 293.80 | 276.59 | 321.14 | 252.86 |
| NSVFLIEMLMPK | 13262 | 37.24 | 3 | 1436.7404 | 2 |  | 0 | [8] Oxidation (M) | no | 1533.14 | 307.93 | 882.42 | 1156.23 |
| NSVFLIEMLMPK | 13960 | --- | --- | 1436.7413 | 3 |  | 1 | [10] Oxidation (M) | no | 293.80 | 276.59 | 321.14 | 252.86 |
| NSVFLIEMLMPK | 1223 | 86.90 | 2 | 1436.7406 | 2 |  | 1 | [10] Oxidation (M) | no | 3.30e+004 | 4.34e+004 | 4.58e+004 | 3.87e+004 |
| QAAFHFR | 1426 | 57.01 | 3 | 875.4400 | 2 |  | 0 |  | no | 1900.00 | 2029.12 | 1609.70 | 1809.91 |
| QYFNSNFR | 403 | 30.43 | 3 | 1074.4875 | 2 |  | 0 |  | no | 1.16e+004 | 2.45e+004 | 1.51e+004 | 2.17e+004 |
| RYTASPTHAQCVC | 3037 | 39.85 | 3 | 1549.6712 | 2 |  | 0 |  | no | 947.57 | 1280.63 | 852.26 | 1447.69 |
| RYTASPTHAQCVC | 864 | 35.80 | 3 | 1549.6755 | 3 |  | 0 |  | no | 6310.66 | 9268.31 | 7197.25 | 8546.33 |
| SSFSSYAK | 63 | 60.29 | 3 | 875.4027 | 2 |  | 0 |  | no | 8.22e+004 | 1.10e+005 | 9.55e+004 | 1.07e+005 |
| SVSGLSHSYR | 258 | 27.77 | 3 | 1091.5362 | 3 |  | 0 |  | no | 1.27e+004 | 1.73e+004 | 1.44e+004 | 2.10e+004 |
| SVSGLSHSYR | 417 | 59.99 | 3 | 1091.5364 | 2 |  | 0 |  | no | 1.24e+004 | 1.71e+004 | 1.43e+004 | 2.07e+004 |
| TKPQMLWDLSGEELEAVHNFVMSSK | 12548 | --- | --- | 2875.3882 | 3 |  | 0 |  | no | 859.71 | 951.39 | 1999.87 | 995.99 |
| TKPQMLWDLSGEELEAVHNFVMSSK | 11061 | 33.92 | 3 | 2875.3944 | 4 |  | 0 |  | no | 910.52 | 1060.94 | 1996.65 | 1025.37 |
| TQYSWER | 65 | 33.90 | 3 | 968.4349 | 2 |  | 0 |  | no | 9.30e+004 | 1.73e+005 | 1.32e+005 | 1.62e+005 |
| TTEQFPQFSTYKPYAEFLMPISK | 4045 | 43.03 | 3 | 2768.3438 | 3 |  | 0 | [19] Oxidation (M) | no | 6462.15 | 4461.20 | 5287.21 | 2764.29 |
| VESALLLHTLK | 106 | 67.91 | 3 | 1222.7283 | 2 |  | 0 |  | no | 8.35e+004 | 7.54e+004 | 7.36e+004 | 1.10e+005 |
| VESALLLHTLK | 35 | 45.10 | 3 | 1222.7280 | 3 |  | 0 |  | no | 1.28e+005 | 1.21e+005 | 1.23e+005 | 1.87e+005 |
| VIQLYPEFPMPISK | 195 | 44.40 | 3 | 1660.8891 | 3 |  | 0 |  | yes | 7.52e+004 | 9.07e+004 | 9.48e+004 | 8.56e+004 |
| VIQLYPEFPMPISK | 56 | 47.12 | 2 | 1660.8896 | 2 |  | 0 |  | yes | 5.76e+005 | 7.14e+005 | 7.04e+005 | 6.63e+005 |
| YADGEVDIVVLEDPLPK | 228 | 58.57 | 3 | 1870.9559 | 3 |  | 0 |  | no | 1.10e+005 | 1.36e+005 | 1.35e+005 | 7.08e+004 |
| YADGEVDIVVLEDPLPK | 98 | 53.16 | 3 | 1870.9567 | 2 |  | 0 |  | no | 6.22e+005 | 7.38e+005 | 7.18e+005 | 3.99e+005 |
| YEVLAFLDK | 143 | 58.11 | 3 | 1096.5799 | 2 |  | 0 |  | no | 6.86e+004 | 1.12e+005 | 8.26e+004 | 9.34e+004 |
| YEVLAFLDK | 6311 | 50.37 | 3 | 1096.5790 | 2 |  | 0 |  | no | 649.56 | 939.68 | 799.31 | 760.16 |
| YLLFSNTGK | 32 | 51.76 | 3 | 1041.5488 | 2 |  | 0 |  | no | 1.76e+005 | 3.51e+005 | 2.41e+005 | 3.34e+005 |
| YQLAVTK | 34 | 55.36 | 3 | 821.4645 | 2 |  | 0 |  | no | 1.13e+005 | 2.24e+005 | 1.63e+005 | 2.40e+005 |
| YTASPTHAQCVC | 54 | 58.94 | 3 | 1393.5748 | 2 |  | 0 |  | no | 3.11e+005 | 4.37e+005 | 3.57e+005 | 4.91e+005 |

  

| Tags | |
| --- | --- |

  

##

## Q62216

Q62216\_MOUSE
Semenoclotin OS=Mus musculus GN=Semg1 PE=2 SV=1  
26 peptides

  

| Sequence | Peptide Ion | Score | Hits | Mass | Charge | Tags | Conflicts | Modifications | In quantitation | Average Normalised Abundances | | | |
| --- | --- | --- | --- | --- | --- | --- | --- | --- | --- | --- | --- | --- | --- |
|  |  |  |  |  |  |  |  |  |  | 2m2f | 4m2f | 2m4f | 4m4f |
| GFAMDEGMSQVR | 1079 | 63.16 | 3 | 1326.5694 | 3 |  | 0 |  | no | 4257.13 | 7538.89 | 4215.82 | 7663.82 |
| GFAMDEGMSQVR | 36 | 93.14 | 3 | 1326.5696 | 2 |  | 0 |  | no | 3.72e+005 | 6.75e+005 | 3.68e+005 | 6.77e+005 |
| GFAMDEGMSQVR | 865 | 74.97 | 3 | 1342.5643 | 2 |  | 0 | [4] Oxidation (M) | no | 2.15e+004 | 2.80e+004 | 2.19e+004 | 2.92e+004 |
| GGADLYQAQLK | 2712 | 30.67 | 3 | 1162.5986 | 3 |  | 0 |  | no | 949.06 | 1251.85 | 1113.62 | 971.83 |
| GGADLYQAQLK | 11 | 74.21 | 3 | 1162.5979 | 2 |  | 0 |  | no | 6.29e+005 | 9.65e+005 | 7.53e+005 | 8.84e+005 |
| GGSDEAAEESLFMQSQR | 49 | 106.13 | 3 | 1840.7901 | 2 |  | 0 |  | no | 5.32e+005 | 7.52e+005 | 6.32e+005 | 6.23e+005 |
| GGSDEAAEESLFMQSQR | 73 | 67.15 | 3 | 1840.7893 | 3 |  | 0 |  | no | 1.95e+005 | 2.74e+005 | 2.29e+005 | 2.33e+005 |
| GGSDEAAEESLFMQSQR | 1589 | 46.91 | 3 | 1856.7849 | 2 |  | 0 | [13] Oxidation (M) | no | 1.44e+004 | 2.21e+004 | 1.48e+004 | 1.76e+004 |
| GGSDEAAEESLFMQSQR | 1205 | 109.89 | 3 | 1856.7850 | 3 |  | 0 | [13] Oxidation (M) | no | 1.10e+004 | 1.72e+004 | 1.14e+004 | 1.38e+004 |
| GHFQSSSSEGFMLGQK | 441 | 114.42 | 3 | 1725.7778 | 2 |  | 0 |  | no | 3.82e+004 | 3.47e+004 | 3.41e+004 | 3.22e+004 |
| GHFQSSSSEGFMLGQK | 66 | 48.62 | 3 | 1725.7781 | 3 |  | 0 |  | no | 1.86e+005 | 2.15e+005 | 1.89e+005 | 2.16e+005 |
| GHFQSSSSEGFMLGQK | 8372 | 41.03 | 2 | 1741.7718 | 2 |  | 0 | [12] Oxidation (M) | no | 1053.33 | 538.40 | 739.27 | 628.91 |
| GHFQSSSSEGFMLGQK | 1019 | 69.05 | 3 | 1741.7720 | 3 |  | 0 | [12] Oxidation (M) | no | 1.21e+004 | 9676.65 | 1.03e+004 | 8179.70 |
| GHLSFGIK | 82 | 41.90 | 3 | 857.4755 | 2 |  | 0 |  | no | 6.12e+004 | 8.77e+004 | 8.25e+004 | 2.59e+004 |
| GYLEQYR | 14 | 36.69 | 3 | 927.4444 | 2 |  | 0 |  | no | 2.95e+005 | 4.24e+005 | 3.37e+005 | 4.40e+005 |
| GYLEQYRK | 2236 | 32.60 | 3 | 1055.5362 | 2 |  | 0 |  | no | 1252.81 | 2306.25 | 1420.07 | 2113.97 |
| GYLEQYRK | 1914 | --- | --- | 1055.5415 | 3 |  | 0 |  | no | 689.58 | 1310.42 | 868.58 | 1249.81 |
| KNFNPGNYFTK | 467 | 60.48 | 3 | 1328.6515 | 2 |  | 0 |  | no | 2.36e+004 | 3.07e+004 | 2.66e+004 | 4.00e+004 |
| KNFNPGNYFTK | 262 | 45.51 | 3 | 1328.6508 | 3 |  | 0 |  | no | 1.88e+004 | 2.73e+004 | 2.36e+004 | 3.64e+004 |
| KQFSDDDLSVQQK | 90 | 51.98 | 3 | 1536.7408 | 3 |  | 0 |  | no | 7.75e+004 | 9.26e+004 | 8.53e+004 | 9.77e+004 |
| KQFSDDDLSVQQK | 394 | 87.97 | 3 | 1536.7414 | 2 |  | 0 |  | no | 2.29e+004 | 2.55e+004 | 2.40e+004 | 2.61e+004 |
| KQFSDDDLSVQQK | 7279 | 36.61 | 3 | 1536.7406 | 3 |  | 0 |  | no | 462.21 | 619.10 | 521.62 | 483.57 |
| NFNPGNYFTK | 17 | 48.50 | 3 | 1200.5560 | 2 |  | 0 |  | no | 5.60e+005 | 1.04e+006 | 7.58e+005 | 9.63e+005 |
| QFSDDDLSVQQK | 151 | 76.42 | 3 | 1408.6463 | 2 |  | 0 |  | no | 9.45e+004 | 1.23e+005 | 8.97e+004 | 1.29e+005 |
| QFSDDDLSVQQK | 5584 | 26.62 | 2 | 1408.6469 | 2 |  | 0 |  | no | 2390.29 | 4386.77 | 1979.69 | 4653.78 |
| QFSDDDLSVQQK | 3002 | 27.64 | 3 | 1408.6465 | 3 |  | 0 |  | no | 1522.98 | 2325.36 | 1580.10 | 2814.52 |
| SGGPAFGQVK | 6 | 93.50 | 3 | 946.4864 | 2 |  | 0 |  | no | 5.76e+005 | 8.06e+005 | 6.32e+005 | 7.25e+005 |
| SGGPAFGQVKSQESQIK | 5252 | 26.35 | 3 | 1746.8831 | 3 |  | 0 |  | no | 645.80 | 696.04 | 709.99 | 902.16 |
| SGGSAFGQVK | 1 | 77.15 | 3 | 936.4657 | 2 |  | 0 |  | yes | 9.93e+005 | 1.24e+006 | 1.14e+006 | 1.13e+006 |
| SQESQIK | 872 | 30.63 | 3 | 818.4132 | 2 |  | 0 |  | no | 2.15e+004 | 1.76e+004 | 1.70e+004 | 1.69e+004 |
| SQESQIKSYGQVK | 6017 | 25.30 | 3 | 1480.7513 | 3 |  | 0 |  | no | 561.03 | 742.19 | 744.73 | 885.05 |
| SQQQSSFSQVK | 20 | 53.34 | 3 | 1252.6042 | 2 |  | 0 |  | no | 6.43e+005 | 7.80e+005 | 7.07e+005 | 8.68e+005 |
| SQQQSSFSQVK | 1139 | 54.46 | 3 | 1252.6044 | 2 |  | 0 |  | no | 1.15e+004 | 1.77e+004 | 1.22e+004 | 1.55e+004 |
| SQQQSSFSQVK | 3069 | 31.71 | 3 | 1252.6046 | 3 |  | 0 |  | no | 1697.08 | 1955.73 | 1634.69 | 1777.43 |
| SSVSQIK | 1928 | 52.69 | 3 | 747.4125 | 2 |  | 0 |  | no | 3899.93 | 4142.40 | 3885.87 | 3888.91 |
| SYGEEGQLNSFSQLK | 16 | 94.43 | 3 | 1685.7893 | 2 |  | 0 |  | yes | 9.07e+005 | 1.21e+006 | 9.77e+005 | 1.12e+006 |
| SYGEEGQLNSFSQLK | 130 | 77.66 | 3 | 1685.7891 | 3 |  | 0 |  | yes | 6.35e+004 | 8.13e+004 | 6.67e+004 | 7.64e+004 |
| TEEDLSQFGQQR | 3281 | 69.86 | 3 | 1436.6612 | 2 |  | 0 |  | no | 2368.67 | 3204.51 | 2910.33 | 3959.66 |
| TEEDLSQFGQQR | 10 | 96.25 | 3 | 1436.6524 | 2 |  | 0 |  | yes | 8.33e+005 | 1.32e+006 | 9.62e+005 | 1.16e+006 |
| TEEDLSQFGQQR | 107 | 48.70 | 3 | 1436.6528 | 3 |  | 0 |  | yes | 6.39e+004 | 1.04e+005 | 7.41e+004 | 9.10e+004 |
| TEEDLSQFGQQR | 1002 | 88.56 | 3 | 1436.6531 | 2 |  | 0 |  | no | 1.06e+004 | 2.25e+004 | 1.29e+004 | 2.13e+004 |
| TEEDLSQFGQQR | 7807 | 30.74 | 2 | 1436.6524 | 3 |  | 0 |  | no | 313.39 | 681.12 | 431.86 | 647.51 |
| VSQEHTSVK | 5533 | 61.75 | 3 | 1013.5134 | 2 |  | 0 |  | no | 2335.32 | 2062.78 | 2120.85 | 1825.10 |
| VYGQGGGDMTQTR | 31 | 94.13 | 3 | 1368.6085 | 2 |  | 0 |  | no | 5.16e+005 | 6.50e+005 | 5.82e+005 | 7.31e+005 |
| VYGQGGGDMTQTR | 2052 | 48.48 | 3 | 1368.6068 | 3 |  | 0 |  | no | 2975.44 | 3700.07 | 3036.41 | 4130.03 |
| VYGQGGGDMTQTR | 514 | 101.42 | 3 | 1384.6035 | 2 |  | 0 | [9] Oxidation (M) | no | 3.48e+004 | 3.53e+004 | 3.55e+004 | 3.29e+004 |
| VYGQGGGDMTQTR | 6732 | 59.97 | 1 | 1384.6006 | 2 |  | 0 | [9] Oxidation (M) | no | 877.33 | 1728.76 | 760.36 | 1052.12 |

  

| Tags | |
| --- | --- |

  

##

## Q8BTM8

FLNA\_MOUSE
Filamin-A OS=Mus musculus GN=Flna PE=1 SV=4  
31 peptides

  

| Sequence | Peptide Ion | Score | Hits | Mass | Charge | Tags | Conflicts | Modifications | In quantitation | Average Normalised Abundances | | | |
| --- | --- | --- | --- | --- | --- | --- | --- | --- | --- | --- | --- | --- | --- |
|  |  |  |  |  |  |  |  |  |  | 2m2f | 4m2f | 2m4f | 4m4f |
| AEAGVPAEFGIWTR | 4058 | 70.49 | 3 | 1502.7533 | 2 |  | 0 |  | yes | 2260.29 | 881.97 | 2517.29 | 1434.83 |
| AEGPGLNR | 10487 | 43.69 | 3 | 812.4149 | 2 |  | 0 |  | no | 215.82 | 26.88 | 163.37 | 70.48 |
| AFGPGLQGGNAGSPAR | 5792 | 86.94 | 3 | 1455.7207 | 2 |  | 0 |  | no | 972.55 | 218.26 | 920.94 | 487.98 |
| ANLPQSFQVDTSK | 6740 | 67.79 | 1 | 1433.7145 | 2 |  | 0 |  | no | 1097.99 | 264.04 | 844.25 | 712.60 |
| AWGPGLEGGIVGK | 3969 | 70.45 | 3 | 1239.6596 | 2 |  | 0 |  | yes | 1561.09 | 1535.03 | 1821.47 | 1779.74 |
| DVDIIDHHDNTYTVK | 7866 | 29.08 | 2 | 1783.8368 | 3 |  | 0 |  | no | 763.62 | 95.93 | 620.13 | 386.90 |
| EAGAGGLAIAVEGPSK | 4863 | 94.10 | 3 | 1425.7459 | 2 |  | 0 |  | no | 1436.32 | 379.95 | 1213.53 | 645.23 |
| EATTEFSVDAR | 4164 | 74.63 | 2 | 1224.5586 | 2 |  | 0 |  | no | 1326.22 | 849.56 | 1353.06 | 1276.71 |
| EGSYSISVLYGEEEVPR | 11361 | 76.72 | 3 | 1912.9069 | 2 |  | 0 |  | no | 629.83 | 54.14 | 697.78 | 221.49 |
| GAGTGGLGLAVEGPSEAK | 4802 | 87.64 | 3 | 1569.7993 | 2 |  | 0 |  | no | 1846.43 | 558.36 | 1935.41 | 879.18 |
| GTVEPQLEAR | 6014 | 50.31 | 3 | 1098.5636 | 2 |  | 0 |  | no | 704.93 | 188.92 | 662.49 | 416.34 |
| IANLQTDLSDGLR | 3732 | 93.73 | 3 | 1414.7441 | 2 |  | 0 |  | yes | 2509.36 | 3080.05 | 2525.79 | 3417.50 |
| IECDDKGDGSCDVR | 6720 | 47.40 | 3 | 1624.6449 | 3 |  | 1 |  | no | 659.81 | 205.30 | 867.58 | 493.93 |
| LDVQFSGLAK | 7338 | 53.60 | 3 | 1076.5868 | 2 |  | 0 |  | no | 446.72 | 124.15 | 416.38 | 191.09 |
| LIALLEVLSQK | 5086 | 62.70 | 3 | 1225.7644 | 2 |  | 1 |  | no | 2359.91 | 608.15 | 2757.47 | 869.96 |
| LIALLEVLSQKK | 15161 | 26.90 | 3 | 1353.8585 | 3 |  | 0 |  | no | 100.58 | 24.06 | 185.03 | 70.70 |
| LVSIDSK | 7329 | 45.02 | 3 | 760.4324 | 2 |  | 1 |  | no | 306.54 | 91.13 | 256.42 | 179.20 |
| SAGQGEVLVYVEDPAGHQEEAK | 6646 | 65.85 | 2 | 2312.0924 | 3 |  | 0 |  | no | 1147.29 | 326.61 | 1233.93 | 659.87 |
| SNFTVDCSK | 10153 | 45.47 | 1 | 1056.4550 | 2 |  | 1 |  | no | 379.14 | 28.83 | 332.28 | 134.88 |
| SPFEVYVDK | 5257 | 51.33 | 3 | 1082.5278 | 2 |  | 0 |  | no | 875.69 | 297.70 | 729.83 | 587.12 |
| SPFSVGVSPSLDLSK | 4486 | 81.09 | 3 | 1518.7927 | 2 |  | 0 |  | no | 1574.28 | 488.63 | 1449.99 | 858.78 |
| TFSVWYVPEVTGTHK | 5513 | 49.36 | 3 | 1749.8713 | 3 |  | 0 |  | no | 373.18 | 201.66 | 882.02 | 598.20 |
| TGVAVNKPAEFTVDAK | 28150 | --- | --- | 1645.8665 | 2 |  | 0 |  | no | 156.40 | 14.54 | 90.63 | 61.59 |
| TGVAVNKPAEFTVDAK | 4522 | 28.08 | 1 | 1645.8668 | 3 |  | 0 |  | no | 1081.56 | 253.35 | 1501.28 | 870.13 |
| VATVPQHATSGPGPADVSK | 6200 | 64.59 | 3 | 1817.9262 | 3 |  | 0 |  | no | 1064.68 | 187.81 | 1269.40 | 797.33 |
| VHGPGIQSGTTNKPNK | 12951 | --- | --- | 1633.8538 | 4 |  | 0 |  | no | 224.43 | 12.94 | 204.61 | 98.95 |
| VHGPGIQSGTTNKPNK | 15077 | 29.38 | 2 | 1633.8533 | 3 |  | 0 |  | no | 316.72 | 8.40e-003 | 151.66 | 148.14 |
| VHSPSGALEECYVTEIDQDK | 9127 | 35.84 | 2 | 2276.0270 | 3 |  | 0 |  | no | 663.92 | 71.90 | 649.70 | 135.53 |
| VPVHDVTDASK | 8044 | 65.47 | 3 | 1166.5931 | 2 |  | 0 |  | no | 474.66 | 40.51 | 407.58 | 302.33 |
| VPVHDVTDASK | 4882 | --- | --- | 1166.5939 | 3 |  | 0 |  | no | 572.25 | 150.19 | 596.14 | 451.08 |
| VQVQDNEGCSVEATVK | 10148 | 81.24 | 3 | 1761.8206 | 2 |  | 0 |  | no | 763.27 | 72.74 | 606.84 | 187.60 |
| VTAQGPGLEPSGNIANK | 6569 | 63.62 | 3 | 1651.8525 | 2 |  | 0 |  | no | 1001.57 | 185.54 | 897.81 | 419.01 |
| VTYTPMAPGSYLISIK | 9172 | 31.09 | 2 | 1739.9167 | 2 |  | 0 |  | no | 768.75 | 167.33 | 858.05 | 569.15 |
| VTYTPMAPGSYLISIK | 25228 | --- | --- | 1739.9157 | 3 |  | 0 |  | no | 91.45 | 11.20 | 87.31 | 27.69 |
| WGDEHIPGSPYR | 8341 | 32.50 | 3 | 1412.6453 | 3 |  | 0 |  | no | 221.16 | 112.60 | 272.60 | 159.45 |

  

| Tags | |
| --- | --- |

  

##

## P07724

ALBU\_MOUSE
Serum albumin OS=Mus musculus GN=Alb PE=1 SV=3  
25 peptides

  

| Sequence | Peptide Ion | Score | Hits | Mass | Charge | Tags | Conflicts | Modifications | In quantitation | Average Normalised Abundances | | | |
| --- | --- | --- | --- | --- | --- | --- | --- | --- | --- | --- | --- | --- | --- |
|  |  |  |  |  |  |  |  |  |  | 2m2f | 4m2f | 2m4f | 4m4f |
| AADKDTCFSTEGPNLVTR | 1084 | 49.69 | 3 | 1980.9211 | 3 |  | 0 |  | no | 1.31e+004 | 1.19e+004 | 1.17e+004 | 1.09e+004 |
| AADKDTCFSTEGPNLVTR | 24761 | --- | --- | 1980.9140 | 2 |  | 0 |  | no | 395.03 | 321.90 | 243.93 | 316.00 |
| AETFTFHSDICTLPEK | 1507 | 49.49 | 3 | 1894.8687 | 3 |  | 0 |  | no | 1.08e+004 | 8188.34 | 1.05e+004 | 7932.16 |
| APQVSTPTLVEAAR | 794 | 63.33 | 3 | 1438.7759 | 2 |  | 0 |  | yes | 2.23e+004 | 2.16e+004 | 2.02e+004 | 2.16e+004 |
| DTCFSTEGPNLVTR | 7404 | 37.45 | 3 | 1595.7246 | 2 |  | 0 |  | no | 1054.45 | 864.88 | 840.40 | 679.01 |
| DVFLGTFLYEYSR | 31430 | --- | --- | 1608.7818 | 3 |  | 0 |  | no | 94.19 | 41.73 | 140.68 | 64.99 |
| DVFLGTFLYEYSR | 6052 | 49.30 | 3 | 1608.7839 | 2 |  | 0 |  | no | 3443.06 | 1663.64 | 4325.52 | 1288.96 |
| ECCHGDLLECADDR | 1867 | 52.27 | 3 | 1748.6546 | 3 |  | 0 |  | no | 3977.13 | 3479.31 | 3826.42 | 3279.68 |
| ECCHGDLLECADDRAELAK | 5459 | 46.23 | 3 | 2260.9481 | 4 |  | 0 |  | no | 1027.97 | 537.73 | 1305.63 | 866.82 |
| ECCHGDLLECADDRAELAK | 9630 | --- | --- | 2260.9506 | 3 |  | 0 |  | no | 1505.08 | 465.48 | 870.90 | 805.87 |
| ENYGELADCCTK | 1179 | 61.84 | 3 | 1458.5763 | 2 |  | 0 |  | no | 1.05e+004 | 1.14e+004 | 9762.76 | 1.12e+004 |
| ENYGELADCCTK | 21633 | --- | --- | 1458.5771 | 3 |  | 0 |  | no | 84.02 | 110.38 | 59.85 | 87.30 |
| GLVLIAFSQYLQK | 3423 | 84.35 | 3 | 1478.8496 | 2 |  | 0 |  | no | 8011.16 | 4139.49 | 7550.32 | 2424.57 |
| GLVLIAFSQYLQK | 7536 | 38.20 | 3 | 1478.8490 | 3 |  | 0 |  | no | 1228.88 | 687.51 | 1184.80 | 296.06 |
| HPDYSVSLLLR | 6727 | 57.98 | 3 | 1298.6962 | 3 |  | 0 |  | no | 438.74 | 224.19 | 526.59 | 287.29 |
| KQTALAELVK | 4359 | --- | --- | 1099.6596 | 2 |  | 0 |  | no | 767.61 | 629.66 | 768.17 | 714.96 |
| KQTALAELVK | 11449 | 43.09 | 3 | 1099.6601 | 3 |  | 0 |  | no | 121.32 | 82.88 | 124.41 | 61.07 |
| KYEATLEK | 1814 | 37.24 | 3 | 980.5176 | 2 |  | 0 |  | no | 2503.77 | 2193.92 | 2288.04 | 2090.42 |
| LATDLTK | 892 | 32.97 | 3 | 760.4328 | 2 |  | 0 |  | no | 4762.50 | 4762.84 | 4020.70 | 4451.91 |
| LCAIPNLR | 993 | 38.71 | 3 | 955.5269 | 2 |  | 0 |  | no | 4366.77 | 5125.57 | 4069.88 | 4735.29 |
| LGEYGFQNAILVR | 9421 | 31.12 | 3 | 1478.7898 | 3 |  | 0 |  | yes | 379.04 | 277.17 | 453.40 | 375.45 |
| LGEYGFQNAILVR | 778 | 98.40 | 3 | 1478.7886 | 2 |  | 0 |  | yes | 1.84e+004 | 1.74e+004 | 1.90e+004 | 1.80e+004 |
| LPCVEDYLSAILNR | 4456 | 28.64 | 3 | 1661.8437 | 3 |  | 0 |  | no | 8574.22 | 2529.73 | 5916.71 | 2071.92 |
| LPCVEDYLSAILNR | 5226 | 49.79 | 3 | 1661.8446 | 2 |  | 0 |  | no | 1.26e+004 | 3142.09 | 8290.17 | 2678.93 |
| LQTCCDKPLLK | 1294 | 27.59 | 1 | 1374.7121 | 3 |  | 0 |  | no | 4542.29 | 4114.14 | 3686.13 | 5757.39 |
| LSQTFPNADFAEITK | 1719 | 42.40 | 2 | 1680.8356 | 2 |  | 0 |  | yes | 2.59e+004 | 2.79e+004 | 2.69e+004 | 2.41e+004 |
| LVQEVTDFAK | 508 | 68.10 | 3 | 1148.6072 | 2 |  | 0 |  | no | 1.41e+004 | 1.34e+004 | 1.15e+004 | 1.35e+004 |
| RPCFSALTVDETYVPK | 3818 | 32.95 | 3 | 1881.9304 | 3 |  | 0 |  | no | 2763.99 | 1621.95 | 2481.16 | 1925.50 |
| SLHTLFGDK | 2742 | 26.96 | 3 | 1016.5314 | 2 |  | 0 |  | no | 1672.48 | 1232.73 | 1247.53 | 1128.24 |
| TCVADESAANCDK | 1156 | 93.89 | 3 | 1439.5677 | 2 |  | 0 |  | no | 1.42e+004 | 1.36e+004 | 1.23e+004 | 1.25e+004 |
| TVMDDFAQFLDTCCK | 3767 | 99.86 | 3 | 1849.7691 | 2 |  | 0 |  | no | 1.06e+004 | 5289.25 | 9379.50 | 5943.43 |
| TVMDDFAQFLDTCCK | 4905 | 71.95 | 3 | 1849.7673 | 3 |  | 0 |  | no | 3381.06 | 1741.32 | 3315.76 | 1921.93 |
| YMCENQATISSK | 1626 | 55.46 | 3 | 1430.6134 | 2 |  | 0 |  | no | 8345.35 | 7356.35 | 6740.20 | 6600.42 |
| YNDLGEQHFK | 1222 | 45.11 | 3 | 1249.5728 | 3 |  | 0 |  | no | 2706.65 | 2318.57 | 2409.45 | 2387.76 |
| YNDLGEQHFK | 4136 | 47.31 | 3 | 1249.5714 | 2 |  | 0 |  | no | 1005.49 | 785.24 | 812.29 | 766.82 |

  

| Tags | |
| --- | --- |

  

##

## Q8VI13

Q8VI13\_MOUSE
Seminal vesicle secretion III OS Mus musculus GN Svs3a PE 2 SV 1  
19
peptides

  

| Sequence | Peptide Ion | Score | Hits | Mass | Charge | Tags | Conflicts | Modifications | In quantitation | Average Normalised Abundances | | | |
| --- | --- | --- | --- | --- | --- | --- | --- | --- | --- | --- | --- | --- | --- |
|  |  |  |  |  |  |  |  |  |  | 2m2f | 4m2f | 2m4f | 4m4f |
| EDIPQQVK | 70 | 49.49 | 3 | 955.4972 | 2 |  | 0 |  | no | 1.24e+005 | 1.63e+005 | 1.36e+005 | 1.54e+005 |
| EDIVCEEEDELAQQK | 281 | 77.19 | 3 | 1833.7960 | 3 |  | 0 |  | no | 4.99e+004 | 8.23e+004 | 6.18e+004 | 6.96e+004 |
| EDIVCEEEDELAQQK | 387 | 105.96 | 3 | 1833.7945 | 2 |  | 0 |  | no | 5.90e+004 | 9.14e+004 | 6.70e+004 | 7.34e+004 |
| EEQEEAPEESIFVQTK | 152 | 62.56 | 3 | 1891.8696 | 3 |  | 0 |  | yes | 7.71e+004 | 1.09e+005 | 9.63e+004 | 1.17e+005 |
| EEQEEAPEESIFVQTK | 109 | 81.08 | 3 | 1891.8692 | 2 |  | 0 |  | yes | 2.72e+005 | 3.94e+005 | 3.45e+005 | 4.24e+005 |
| GGVILYQDAFTD | 3051 | 48.68 | 3 | 1297.6272 | 2 |  | 0 |  | no | 2100.48 | 2488.79 | 2532.81 | 2757.41 |
| GGVILYQDAFTD | 78 | 82.01 | 3 | 1297.6191 | 2 |  | 0 |  | yes | 2.34e+005 | 4.04e+005 | 2.83e+005 | 3.47e+005 |
| GHFLVK | 269 | 32.46 | 3 | 699.4074 | 2 |  | 0 |  | no | 1.06e+004 | 1.22e+004 | 1.06e+004 | 1.14e+004 |
| GYGLAEDLAQVR | 593 | 68.05 | 3 | 1290.6569 | 3 |  | 0 |  | no | 6471.63 | 1.24e+004 | 1.06e+004 | 9737.64 |
| HHAYGQDADADMGGALSSQELTSLK | 1990 | 94.00 | 3 | 2601.1807 | 4 |  | 0 |  | no | 5151.17 | 5198.78 | 6462.13 | 4145.90 |
| HHAYGQDADADMGGALSSQELTSLK | 2746 | 144.35 | 3 | 2601.1762 | 3 |  | 0 |  | no | 4719.20 | 3790.56 | 5616.78 | 3612.25 |
| KTAAFYPQFR | 9361 | 25.62 | 3 | 1227.6398 | 3 |  | 0 |  | no | 210.60 | 387.10 | 326.49 | 384.36 |
| LNLREDIPQQVK | 15641 | --- | --- | 1451.8098 | 2 |  | 0 |  | no | 149.81 | 97.18 | 136.90 | 95.38 |
| LNLREDIPQQVK | 2952 | 29.91 | 3 | 1451.8124 | 3 |  | 0 |  | no | 1143.31 | 1773.97 | 1459.18 | 2543.28 |
| NQFLYGHK | 364 | 29.78 | 3 | 1005.5026 | 2 |  | 0 |  | no | 1.40e+004 | 1.45e+004 | 1.33e+004 | 1.60e+004 |
| NQFLYGHKEEQEEAPEESIFVQTK | 2480 | 59.03 | 3 | 2879.3576 | 3 |  | 0 |  | no | 5019.81 | 2516.79 | 5548.29 | 4108.63 |
| NQFLYGHKEEQEEAPEESIFVQTK | 966 | 36.95 | 3 | 2879.3607 | 4 |  | 0 |  | no | 1.10e+004 | 6775.55 | 1.42e+004 | 1.10e+004 |
| SFYPGPGMCYCPR | 1333 | 62.14 | 3 | 1590.6409 | 3 |  | 0 |  | no | 4399.23 | 5300.83 | 4549.88 | 5386.40 |
| SFYPGPGMCYCPR | 140 | 72.73 | 3 | 1590.6419 | 2 |  | 0 |  | no | 1.14e+005 | 1.36e+005 | 1.17e+005 | 1.45e+005 |
| SFYPGPGMCYCPR | 4082 | 29.56 | 3 | 1606.6465 | 2 |  | 0 | [8] Oxidation (M) | no | 3805.78 | 3905.13 | 3347.53 | 4615.14 |
| SQLPSQSQIK | 40 | 37.83 | 3 | 1114.5977 | 2 |  | 0 |  | yes | 2.67e+005 | 2.95e+005 | 2.87e+005 | 2.80e+005 |
| SYAAQLK | 74 | 35.69 | 3 | 779.4177 | 2 |  | 0 |  | no | 7.32e+004 | 1.10e+005 | 8.67e+004 | 1.11e+005 |
| TAAFYPQFR | 64 | 45.86 | 3 | 1099.5447 | 2 |  | 0 |  | no | 1.68e+005 | 2.73e+005 | 2.15e+005 | 2.61e+005 |
| TAAFYPQFR | 2885 | 45.93 | 3 | 1099.5455 | 3 |  | 0 |  | no | 854.44 | 1285.10 | 1089.24 | 1260.82 |
| TSPLMFIGK | 96 | 47.72 | 3 | 992.5362 | 2 |  | 0 |  | no | 9.15e+004 | 1.56e+005 | 1.21e+005 | 1.54e+005 |
| TSPLMFIGK | 3815 | 42.24 | 3 | 1008.5315 | 2 |  | 0 | [5] Oxidation (M) | no | 1805.03 | 2265.91 | 2067.65 | 2506.12 |
| YFVQFQEQLQGSVHHTK | 748 | --- | --- | 2075.0163 | 3 |  | 0 |  | no | 1.80e+004 | 2.00e+004 | 1.73e+004 | 1.44e+004 |
| YFVQFQEQLQGSVHHTK | 609 | 42.72 | 3 | 2075.0209 | 4 |  | 0 |  | no | 1.31e+004 | 9360.19 | 1.09e+004 | 8053.69 |

  

| Tags | |
| --- | --- |

  

##

## P20029

GRP78\_MOUSE
78 kDa glucose-regulated protein OS=Mus musculus GN=Hspa5 PE=1 SV=3  
21
peptides

  

| Sequence | Peptide Ion | Score | Hits | Mass | Charge | Tags | Conflicts | Modifications | In quantitation | Average Normalised Abundances | | | |
| --- | --- | --- | --- | --- | --- | --- | --- | --- | --- | --- | --- | --- | --- |
|  |  |  |  |  |  |  |  |  |  | 2m2f | 4m2f | 2m4f | 4m4f |
| DNHLLGTFDLTGIPPAPR | 9452 | 25.84 | 3 | 1933.0056 | 3 |  | 0 |  | no | 944.73 | 160.35 | 723.62 | 561.30 |
| ELEEIVQPIISK | 10665 | --- | --- | 1396.7800 | 3 |  | 0 |  | yes | 208.12 | 159.30 | 219.98 | 132.53 |
| ELEEIVQPIISK | 856 | 75.15 | 3 | 1396.7805 | 2 |  | 0 |  | yes | 1.15e+004 | 1.30e+004 | 1.44e+004 | 1.21e+004 |
| ETAEAYLGK | 1800 | 32.37 | 3 | 980.4808 | 2 |  | 0 |  | no | 2799.17 | 2750.58 | 2746.36 | 2687.89 |
| FEELNMDLFR | 2287 | 72.83 | 3 | 1312.6116 | 2 |  | 0 |  | no | 4724.69 | 3939.15 | 5154.89 | 4788.12 |
| IEIESFFEGEDFSETLTR | 7615 | --- | --- | 2147.9891 | 3 |  | 0 |  | no | 1353.42 | 738.24 | 1319.60 | 333.89 |
| IEIESFFEGEDFSETLTR | 6126 | 41.23 | 3 | 2147.9909 | 2 |  | 0 |  | no | 6261.51 | 2584.40 | 6112.60 | 1754.88 |
| IINEPTAAAIAYGLDK | 1209 | 111.89 | 3 | 1658.8880 | 2 |  | 1 |  | no | 1.01e+004 | 6877.88 | 8351.44 | 8660.21 |
| IINEPTAAAIAYGLDK | 2500 | 90.89 | 3 | 1658.8861 | 3 |  | 1 |  | no | 2329.95 | 1547.30 | 2056.25 | 2056.09 |
| IINEPTAAAIAYGLDKR | 3674 | 28.38 | 3 | 1814.9888 | 3 |  | 0 |  | no | 1817.17 | 1030.32 | 1366.43 | 1573.03 |
| ITITNDQNR | 1795 | 45.30 | 3 | 1073.5456 | 2 |  | 0 |  | no | 4020.60 | 3127.50 | 3745.42 | 3219.90 |
| ITPSYVAFTPEGER | 5717 | --- | --- | 1565.7721 | 3 |  | 0 |  | no | 897.79 | 774.55 | 857.05 | 651.50 |
| ITPSYVAFTPEGER | 1408 | 53.02 | 3 | 1565.7717 | 2 |  | 0 |  | no | 9695.26 | 9315.94 | 9948.63 | 9962.57 |
| KSDIDEIVLVGGSTR | 5336 | 28.83 | 2 | 1587.8540 | 3 |  | 0 |  | no | 1099.14 | 515.01 | 895.01 | 912.26 |
| NELESYAYSLK | 1613 | 75.71 | 3 | 1315.6296 | 2 |  | 0 |  | no | 4845.00 | 5882.89 | 6349.12 | 5700.55 |
| NQLTSNPENTVFDAK | 5339 | 48.37 | 3 | 1676.7996 | 3 |  | 0 |  | yes | 1004.23 | 912.53 | 934.58 | 919.34 |
| NQLTSNPENTVFDAK | 495 | --- | --- | 1676.7353 | 2 |  | 0 |  | yes | 1.98e+004 | 2.99e+004 | 2.30e+004 | 2.35e+004 |
| SDIDEIVLVGGSTR | 1941 | 101.76 | 3 | 1459.7489 | 2 |  | 0 |  | no | 5304.41 | 8474.06 | 6837.27 | 7393.62 |
| SQIFSTASDNQPTVTIK | 2241 | 72.78 | 3 | 1835.9269 | 2 |  | 0 |  | yes | 8170.54 | 9181.43 | 7531.75 | 8480.36 |
| SQIFSTASDNQPTVTIK | 2715 | 72.07 | 3 | 1835.9257 | 3 |  | 0 |  | yes | 3718.79 | 4367.49 | 3593.26 | 3950.84 |
| TFAPEEISAMVLTK | 1951 | 67.07 | 3 | 1535.7899 | 2 |  | 0 |  | no | 9865.93 | 8095.22 | 1.07e+004 | 1.00e+004 |
| TFAPEEISAMVLTK | 4321 | 68.95 | 3 | 1535.7891 | 3 |  | 0 |  | no | 1662.09 | 1359.91 | 1911.86 | 1657.00 |
| TKPYIQVDIGGGQTK | 3238 | 38.63 | 3 | 1603.8556 | 3 |  | 0 |  | no | 2279.54 | 1449.83 | 2222.93 | 2105.69 |
| TWNDPSVQQDIK | 1346 | 87.44 | 3 | 1429.6854 | 2 |  | 0 |  | no | 9549.82 | 8612.41 | 8618.45 | 1.02e+004 |
| VEIIANDQGNR | 1006 | 60.83 | 3 | 1227.6195 | 2 |  | 2 |  | no | 7512.51 | 4609.63 | 5361.71 | 5790.14 |
| VLEDSDLK | 1501 | 48.99 | 3 | 917.4705 | 2 |  | 0 |  | no | 2821.90 | 2093.09 | 2583.31 | 2388.15 |
| VTHAVVTVPAYFNDAQR | 1552 | 46.16 | 3 | 1886.9626 | 3 |  | 0 |  | no | 3699.85 | 1643.24 | 2697.88 | 3369.19 |
| VYEGERPLTK | 7108 | 43.96 | 3 | 1190.6244 | 2 |  | 0 |  | no | 373.73 | 191.13 | 287.97 | 487.61 |
| VYEGERPLTK | 1745 | --- | --- | 1190.6290 | 3 |  | 0 |  | no | 1772.97 | 1273.71 | 1445.22 | 1910.55 |

  

| Tags | |
| --- | --- |

  

##

## P31001

DESM\_MOUSE
Desmin OS=Mus musculus GN=Des PE=1 SV=3  
16 peptides

  

| Sequence | Peptide Ion | Score | Hits | Mass | Charge | Tags | Conflicts | Modifications | In quantitation | Average Normalised Abundances | | | |
| --- | --- | --- | --- | --- | --- | --- | --- | --- | --- | --- | --- | --- | --- |
|  |  |  |  |  |  |  |  |  |  | 2m2f | 4m2f | 2m4f | 4m4f |
| DGEVVSEATQQQHEVL | 12417 | 32.50 | 1 | 1767.8200 | 3 |  | 0 |  | no | 303.41 | 64.32 | 182.35 | 166.06 |
| DGEVVSEATQQQHEVL | 5222 | 69.89 | 3 | 1767.8269 | 2 |  | 0 |  | no | 1887.96 | 611.82 | 1464.25 | 1339.21 |
| EEAENNLAAFR | 2221 | 74.43 | 3 | 1262.5889 | 2 |  | 0 |  | yes | 2799.33 | 1206.14 | 2343.94 | 2289.69 |
| EYQDLLNVK | 2583 | 55.04 | 3 | 1120.5788 | 2 |  | 1 |  | no | 1847.05 | 960.56 | 1558.44 | 1756.13 |
| FASEANGYQDNIAR | 4381 | 96.70 | 3 | 1554.7061 | 2 |  | 0 |  | no | 2449.46 | 835.80 | 2322.55 | 1372.93 |
| FLEQQNAALAAEVNR | 4311 | 116.57 | 3 | 1672.8519 | 2 |  | 0 |  | no | 2338.83 | 652.59 | 2628.86 | 910.62 |
| FLEQQNAALAAEVNR | 6702 | 57.32 | 3 | 1672.8517 | 3 |  | 0 |  | no | 612.62 | 140.49 | 675.90 | 266.78 |
| HQIQSYTCEIDALK | 6462 | 57.06 | 2 | 1704.8126 | 3 |  | 0 |  | no | 571.55 | 177.27 | 708.74 | 391.19 |
| INLPIQTFSALNFR | 11374 | 37.23 | 3 | 1632.8977 | 3 |  | 0 |  | yes | 603.26 | 129.24 | 827.07 | 218.08 |
| INLPIQTFSALNFR | 6549 | 52.64 | 3 | 1632.8991 | 2 |  | 0 |  | yes | 3599.00 | 924.94 | 4980.80 | 1665.84 |
| LLEGEESR | 1786 | 53.36 | 3 | 931.4603 | 2 |  | 2 |  | no | 2674.46 | 1761.54 | 2395.89 | 2386.46 |
| LQEEIQLR | 2320 | 48.34 | 3 | 1027.5648 | 2 |  | 0 |  | no | 2352.72 | 787.97 | 2238.09 | 1435.09 |
| MALDVEIATYR | 4948 | 73.62 | 3 | 1280.6380 | 2 |  | 0 |  | no | 1079.62 | 371.98 | 832.51 | 277.65 |
| NISEAEEWYK | 2399 | 44.31 | 3 | 1267.5729 | 2 |  | 0 |  | yes | 3377.15 | 1460.75 | 2936.72 | 2470.76 |
| TFGGAPGFSLGSPLSSPVFPR | 8646 | 52.99 | 3 | 2077.0570 | 3 |  | 0 |  | no | 1272.22 | 333.68 | 1671.91 | 550.68 |
| TFGGAPGFSLGSPLSSPVFPR | 13724 | 62.66 | 1 | 2077.0618 | 2 |  | 0 |  | no | 1061.31 | 233.29 | 1292.72 | 311.69 |
| TSGGAGGLGSLR | 3241 | 87.02 | 3 | 1031.5358 | 2 |  | 0 |  | no | 1060.73 | 427.14 | 1036.11 | 717.23 |
| VAELYEEEMR | 3374 | 51.68 | 1 | 1267.5748 | 2 |  | 0 |  | no | 2174.19 | 1507.16 | 2224.15 | 2155.30 |
| VELQELNDR | 1956 | 61.13 | 3 | 1114.5606 | 2 |  | 1 |  | no | 2040.94 | 703.53 | 1978.04 | 1167.96 |
| VSDLTQAANK | 2648 | 60.86 | 3 | 1045.5409 | 2 |  | 0 |  | no | 2730.85 | 981.13 | 2656.39 | 1748.85 |

  

| Tags | |
| --- | --- |

  

##

## Q8BND5

QSOX1\_MOUSE
Sulfhydryl oxidase 1 OS=Mus musculus GN=Qsox1 PE=2 SV=1  
18
peptides

  

| Sequence | Peptide Ion | Score | Hits | Mass | Charge | Tags | Conflicts | Modifications | In quantitation | Average Normalised Abundances | | | |
| --- | --- | --- | --- | --- | --- | --- | --- | --- | --- | --- | --- | --- | --- |
|  |  |  |  |  |  |  |  |  |  | 2m2f | 4m2f | 2m4f | 4m4f |
| ADYLALVFER | 1414 | 68.20 | 3 | 1195.6233 | 2 |  | 0 |  | no | 6469.12 | 8164.60 | 9510.43 | 7459.78 |
| AHFSPANIVIDSSASR | 8539 | --- | --- | 1670.8351 | 2 |  | 0 |  | no | 766.82 | 438.72 | 565.00 | 389.20 |
| AHFSPANIVIDSSASR | 1971 | 64.59 | 3 | 1670.8360 | 3 |  | 0 |  | no | 3744.82 | 2760.92 | 2915.17 | 3202.63 |
| DTWPPACPPLEPAK | 4140 | 33.69 | 3 | 1577.7513 | 2 |  | 0 |  | no | 5150.61 | 6496.55 | 5044.17 | 4816.11 |
| DWRPALNLAVLDCAEETNSAVCR | 6381 | 55.55 | 3 | 2659.2503 | 3 |  | 0 |  | no | 3115.87 | 1337.93 | 1712.29 | 1069.92 |
| EFNIAGFPTVR | 841 | 56.90 | 3 | 1249.6447 | 2 |  | 0 |  | yes | 1.16e+004 | 1.70e+004 | 1.28e+004 | 1.27e+004 |
| EVTLDLSQYHAVAVR | 2835 | 39.15 | 3 | 1699.8879 | 3 |  | 0 |  | no | 3067.11 | 2084.00 | 2100.14 | 1237.26 |
| EVTLDLSQYHAVAVR | 12101 | --- | --- | 1699.8897 | 2 |  | 0 |  | no | 500.16 | 224.32 | 306.00 | 45.77 |
| FGVTDFPSCYLLLR | 4117 | 33.72 | 2 | 1686.8429 | 2 |  | 0 |  | yes | 1.85e+004 | 1.09e+004 | 1.76e+004 | 1.34e+004 |
| IPYSFFK | 1699 | 26.70 | 3 | 900.4738 | 2 |  | 0 |  | no | 2711.01 | 4184.24 | 3064.73 | 4034.81 |
| IYMADLESALHYILR | 12096 | 33.03 | 3 | 1806.9326 | 3 |  | 0 |  | no | 637.15 | 465.68 | 625.65 | 606.94 |
| LIDALESHR | 1762 | 53.30 | 3 | 1052.5618 | 3 |  | 0 |  | no | 987.59 | 1225.97 | 1023.19 | 984.67 |
| LNDIDGFFTR | 747 | 77.77 | 3 | 1196.5826 | 2 |  | 0 |  | no | 9412.35 | 1.61e+004 | 1.11e+004 | 1.31e+004 |
| LSGALSEDPHFPK | 1710 | 56.87 | 3 | 1396.6976 | 3 |  | 0 |  | no | 2620.25 | 3228.83 | 2329.72 | 2951.68 |
| LSGALSEDPHFPK | 5497 | 46.81 | 2 | 1396.6931 | 2 |  | 0 |  | no | 698.73 | 632.64 | 473.63 | 488.28 |
| SPSNAILWLWTSHNR | 4094 | 70.89 | 3 | 1780.9002 | 3 |  | 0 |  | no | 1741.69 | 2092.25 | 2904.70 | 2582.74 |
| SYVQFFFGCR | 2169 | 63.01 | 3 | 1309.5910 | 2 |  | 0 |  | no | 4612.15 | 5722.43 | 6039.20 | 5204.21 |
| VLNTESDLVNK | 621 | 97.88 | 3 | 1230.6446 | 2 |  | 0 |  | yes | 1.18e+004 | 2.24e+004 | 1.41e+004 | 1.89e+004 |
| VNWVGCQGSEPHFR | 3015 | 43.57 | 3 | 1671.7566 | 3 |  | 0 |  | no | 2485.61 | 2104.15 | 1726.83 | 1953.60 |
| VPVLVESR | 603 | 52.14 | 3 | 897.5278 | 2 |  | 0 |  | no | 6850.37 | 1.01e+004 | 7981.59 | 9015.44 |
| YSEAHPQEPADGQEVLQAMR | 2735 | 52.60 | 3 | 2255.0265 | 3 |  | 0 |  | no | 5822.47 | 4509.55 | 4045.80 | 4330.08 |

  

| Tags | |
| --- | --- |

  

##

## P37804

TAGL\_MOUSE
Transgelin OS=Mus musculus GN=Tagln PE=1 SV=3  
14 peptides

  

| Sequence | Peptide Ion | Score | Hits | Mass | Charge | Tags | Conflicts | Modifications | In quantitation | Average Normalised Abundances | | | |
| --- | --- | --- | --- | --- | --- | --- | --- | --- | --- | --- | --- | --- | --- |
|  |  |  |  |  |  |  |  |  |  | 2m2f | 4m2f | 2m4f | 4m4f |
| AAEDYGVIK | 327 | 65.78 | 3 | 964.4859 | 2 |  | 0 |  | no | 1.46e+004 | 1.89e+004 | 1.45e+004 | 1.87e+004 |
| DFTDSQLQEGK | 401 | 79.89 | 3 | 1266.5702 | 2 |  | 0 |  | yes | 2.25e+004 | 3.60e+004 | 2.79e+004 | 3.64e+004 |
| GDPNWFMK | 1189 | 40.92 | 3 | 993.4377 | 2 |  | 0 |  | no | 3350.62 | 4942.92 | 3942.22 | 4132.04 |
| GPSYGMSR | 1470 | 40.05 | 3 | 853.3744 | 2 |  | 0 |  | no | 2453.01 | 3104.04 | 2615.84 | 2980.50 |
| HVIGLQMGSNR | 4335 | 42.55 | 3 | 1210.6240 | 3 |  | 0 |  | no | 472.97 | 570.53 | 381.02 | 321.53 |
| HVIGLQMGSNR | 5486 | 38.59 | 3 | 1210.6201 | 2 |  | 0 |  | no | 1272.64 | 1404.18 | 970.37 | 1192.28 |
| KYDEELEER | 737 | 66.12 | 3 | 1209.5509 | 2 |  | 0 |  | no | 1.03e+004 | 1.51e+004 | 1.12e+004 | 1.41e+004 |
| KYDEELEER | 1166 | 35.70 | 3 | 1209.5515 | 3 |  | 0 |  | no | 3295.36 | 4808.87 | 3619.84 | 4546.79 |
| LGFQVWLK | 716 | 65.60 | 3 | 989.5698 | 2 |  | 0 |  | no | 9274.94 | 8604.41 | 8974.37 | 6866.67 |
| LVEWIVVQCGPDVGRPDR | 2491 | 81.65 | 3 | 2094.0669 | 3 |  | 0 |  | no | 3415.41 | 1771.30 | 3939.50 | 2808.97 |
| LVNSLYPEGSKPVK | 535 | 41.21 | 3 | 1529.8439 | 3 |  | 0 |  | no | 1.33e+004 | 1.23e+004 | 1.25e+004 | 1.34e+004 |
| LVNSLYPEGSKPVK | 2921 | 48.33 | 3 | 1529.8450 | 2 |  | 0 |  | no | 3204.17 | 2383.18 | 2456.20 | 2653.45 |
| NGVILSK | 1181 | 42.63 | 3 | 729.4386 | 2 |  | 0 |  | no | 3483.97 | 3158.90 | 3390.04 | 2912.63 |
| QMEQVAQFLK | 2556 | 59.33 | 3 | 1220.6236 | 2 |  | 0 |  | no | 2106.78 | 2433.69 | 1727.68 | 1949.68 |
| TDMFQTVDLYEGK | 348 | 112.34 | 3 | 1545.7010 | 2 |  | 0 |  | yes | 4.82e+004 | 6.87e+004 | 5.26e+004 | 4.89e+004 |
| TLMALGSLAVTK | 8728 | 37.80 | 3 | 1203.6893 | 3 |  | 0 |  | yes | 184.11 | 221.76 | 201.52 | 100.85 |
| TLMALGSLAVTK | 319 | 73.34 | 3 | 1203.6894 | 2 |  | 0 |  | yes | 2.74e+004 | 4.41e+004 | 2.92e+004 | 2.42e+004 |
| VPENPPSMVFK | 556 | 42.18 | 3 | 1243.6269 | 2 |  | 0 |  | no | 2.74e+004 | 3.36e+004 | 2.46e+004 | 2.88e+004 |

  

| Tags | |
| --- | --- |

  

##

## P62737

ACTA\_MOUSE
Actin, aortic smooth muscle OS=Mus musculus GN=Acta2 PE=1 SV=1  
15
peptides

  

| Sequence | Peptide Ion | Score | Hits | Mass | Charge | Tags | Conflicts | Modifications | In quantitation | Average Normalised Abundances | | | |
| --- | --- | --- | --- | --- | --- | --- | --- | --- | --- | --- | --- | --- | --- |
|  |  |  |  |  |  |  |  |  |  | 2m2f | 4m2f | 2m4f | 4m4f |
| AGFAGDDAPR | 139 | 76.14 | 3 | 975.4377 | 2 |  | 2 |  | no | 5.79e+004 | 4.44e+004 | 5.96e+004 | 6.23e+004 |
| DLTDYLMK | 257 | 45.15 | 3 | 997.4796 | 2 |  | 3 |  | no | 2.12e+004 | 1.57e+004 | 1.93e+004 | 2.56e+004 |
| DLYANNVLSGGTTMYPGIADR | 882 | 54.76 | 3 | 2227.0602 | 3 |  | 1 |  | no | 1.96e+004 | 1.00e+004 | 2.34e+004 | 1.32e+004 |
| DLYANNVLSGGTTMYPGIADR | 2227 | 42.60 | 3 | 2227.0596 | 2 |  | 1 |  | no | 1.19e+004 | 4942.21 | 1.35e+004 | 6822.44 |
| DLYANNVLSGGTTMYPGIADR | 8362 | 31.17 | 2 | 2243.0503 | 3 |  | 1 | [14] Oxidation (M) | no | 873.15 | 160.38 | 751.18 | 486.15 |
| DSYVGDEAQSK | 183 | 72.06 | 3 | 1197.5149 | 2 |  | 2 |  | no | 8.98e+004 | 5.89e+004 | 9.58e+004 | 7.37e+004 |
| EITALAPSTMK | 221 | 50.88 | 3 | 1160.6106 | 2 |  | 2 |  | no | 3.86e+004 | 3.14e+004 | 3.57e+004 | 5.29e+004 |
| GYSFVTTAER | 234 | 60.92 | 3 | 1129.5402 | 2 |  | 1 |  | no | 3.20e+004 | 1.71e+004 | 2.68e+004 | 3.90e+004 |
| IIAPPER | 164 | 32.59 | 3 | 794.4648 | 2 |  | 3 |  | no | 2.79e+004 | 1.34e+004 | 2.56e+004 | 2.12e+004 |
| IWHHSFYNELR | 2231 | 54.44 | 3 | 1500.7249 | 3 |  | 0 |  | yes | 2961.20 | 669.90 | 2149.24 | 2020.92 |
| IWHHSFYNELR | 16218 | --- | --- | 1500.7269 | 2 |  | 0 |  | yes | 389.06 | 14.04 | 89.95 | 66.52 |
| IWHHSFYNELR | 10072 | --- | --- | 1500.7266 | 4 |  | 0 |  | yes | 219.52 | 18.96 | 106.73 | 73.46 |
| KDLYANNVLSGGTTMYPGIADR | 2085 | 58.43 | 3 | 2355.1391 | 3 |  | 1 |  | no | 9310.97 | 3494.08 | 1.05e+004 | 5882.04 |
| LCYVALDFENEMATAASSSSLEK | 632 | 39.75 | 3 | 2535.1505 | 3 |  | 1 |  | no | 7.17e+004 | 1.99e+004 | 5.60e+004 | 1.54e+004 |
| LCYVALDFENEMATAASSSSLEK | 3949 | 54.93 | 3 | 2535.1529 | 2 |  | 1 |  | no | 1.44e+004 | 4385.70 | 1.03e+004 | 4572.48 |
| QEYDEAGPSIVHR | 1250 | 54.65 | 3 | 1499.7029 | 3 |  | 1 |  | no | 5228.41 | 2145.36 | 5600.39 | 4666.00 |
| QEYDEAGPSIVHR | 11060 | 44.28 | 2 | 1499.6994 | 2 |  | 1 |  | no | 743.69 | 98.55 | 467.41 | 377.40 |
| RGILTLK | 1096 | 27.19 | 3 | 799.5279 | 2 |  | 3 |  | no | 1561.95 | 1158.78 | 2123.78 | 1617.99 |
| SYELPDGQVITIGNER | 653 | 64.66 | 3 | 1789.8837 | 3 |  | 3 |  | no | 1.45e+004 | 8872.48 | 1.62e+004 | 1.32e+004 |
| SYELPDGQVITIGNER | 251 | 60.47 | 3 | 1789.8851 | 2 |  | 3 |  | no | 8.21e+004 | 4.91e+004 | 8.77e+004 | 7.29e+004 |
| SYELPDGQVITIGNER | 6364 | 46.14 | 2 | 1789.8846 | 3 |  | 3 |  | no | 402.77 | 125.55 | 125.84 | 177.85 |
| VAPEEHPTLLTEAPLNPK | 4782 | 42.71 | 3 | 1955.0369 | 2 |  | 1 |  | no | 3313.22 | 328.77 | 1816.81 | 1008.48 |
| VAPEEHPTLLTEAPLNPK | 214 | 33.91 | 3 | 1955.0361 | 3 |  | 1 |  | no | 6.36e+004 | 1.34e+004 | 6.08e+004 | 3.70e+004 |
| VAPEEHPTLLTEAPLNPK | 18524 | --- | --- | 1955.0338 | 4 |  | 1 |  | no | 99.11 | 5.39 | 94.90 | 37.76 |

  

| Tags | |
| --- | --- |

  

##

## P68033

ACTC\_MOUSE
Actin, alpha cardiac muscle 1 OS=Mus musculus GN=Actc1 PE=1 SV=1  
15
peptides

  

| Sequence | Peptide Ion | Score | Hits | Mass | Charge | Tags | Conflicts | Modifications | In quantitation | Average Normalised Abundances | | | |
| --- | --- | --- | --- | --- | --- | --- | --- | --- | --- | --- | --- | --- | --- |
|  |  |  |  |  |  |  |  |  |  | 2m2f | 4m2f | 2m4f | 4m4f |
| AGFAGDDAPR | 139 | 76.14 | 3 | 975.4377 | 2 |  | 2 |  | no | 5.79e+004 | 4.44e+004 | 5.96e+004 | 6.23e+004 |
| DLTDYLMK | 257 | 45.15 | 3 | 997.4796 | 2 |  | 3 |  | no | 2.12e+004 | 1.57e+004 | 1.93e+004 | 2.56e+004 |
| DLYANNVLSGGTTMYPGIADR | 882 | 54.76 | 3 | 2227.0602 | 3 |  | 1 |  | no | 1.96e+004 | 1.00e+004 | 2.34e+004 | 1.32e+004 |
| DLYANNVLSGGTTMYPGIADR | 2227 | 42.60 | 3 | 2227.0596 | 2 |  | 1 |  | no | 1.19e+004 | 4942.21 | 1.35e+004 | 6822.44 |
| DLYANNVLSGGTTMYPGIADR | 8362 | 31.17 | 2 | 2243.0503 | 3 |  | 1 | [14] Oxidation (M) | no | 873.15 | 160.38 | 751.18 | 486.15 |
| DSYVGDEAQSK | 183 | 72.06 | 3 | 1197.5149 | 2 |  | 2 |  | no | 8.98e+004 | 5.89e+004 | 9.58e+004 | 7.37e+004 |
| EITALAPSTMK | 221 | 50.88 | 3 | 1160.6106 | 2 |  | 2 |  | no | 3.86e+004 | 3.14e+004 | 3.57e+004 | 5.29e+004 |
| GYSFVTTAER | 234 | 60.92 | 3 | 1129.5402 | 2 |  | 1 |  | no | 3.20e+004 | 1.71e+004 | 2.68e+004 | 3.90e+004 |
| IIAPPER | 164 | 32.59 | 3 | 794.4648 | 2 |  | 3 |  | no | 2.79e+004 | 1.34e+004 | 2.56e+004 | 2.12e+004 |
| IWHHTFYNELR | 3064 | 49.26 | 3 | 1514.7401 | 3 |  | 1 |  | no | 2030.34 | 280.34 | 1446.06 | 1114.07 |
| IWHHTFYNELR | 12476 | --- | --- | 1514.7420 | 4 |  | 1 |  | no | 141.69 | 2.97 | 65.60 | 44.65 |
| KDLYANNVLSGGTTMYPGIADR | 2085 | 58.43 | 3 | 2355.1391 | 3 |  | 1 |  | no | 9310.97 | 3494.08 | 1.05e+004 | 5882.04 |
| LCYVALDFENEMATAASSSSLEK | 632 | 39.75 | 3 | 2535.1505 | 3 |  | 1 |  | no | 7.17e+004 | 1.99e+004 | 5.60e+004 | 1.54e+004 |
| LCYVALDFENEMATAASSSSLEK | 3949 | 54.93 | 3 | 2535.1529 | 2 |  | 1 |  | no | 1.44e+004 | 4385.70 | 1.03e+004 | 4572.48 |
| QEYDEAGPSIVHR | 1250 | 54.65 | 3 | 1499.7029 | 3 |  | 1 |  | no | 5228.41 | 2145.36 | 5600.39 | 4666.00 |
| QEYDEAGPSIVHR | 11060 | 44.28 | 2 | 1499.6994 | 2 |  | 1 |  | no | 743.69 | 98.55 | 467.41 | 377.40 |
| RGILTLK | 1096 | 27.19 | 3 | 799.5279 | 2 |  | 3 |  | no | 1561.95 | 1158.78 | 2123.78 | 1617.99 |
| SYELPDGQVITIGNER | 6364 | 46.14 | 2 | 1789.8846 | 3 |  | 3 |  | no | 402.77 | 125.55 | 125.84 | 177.85 |
| SYELPDGQVITIGNER | 251 | 60.47 | 3 | 1789.8851 | 2 |  | 3 |  | no | 8.21e+004 | 4.91e+004 | 8.77e+004 | 7.29e+004 |
| SYELPDGQVITIGNER | 653 | 64.66 | 3 | 1789.8837 | 3 |  | 3 |  | no | 1.45e+004 | 8872.48 | 1.62e+004 | 1.32e+004 |
| VAPEEHPTLLTEAPLNPK | 4782 | 42.71 | 3 | 1955.0369 | 2 |  | 1 |  | no | 3313.22 | 328.77 | 1816.81 | 1008.48 |
| VAPEEHPTLLTEAPLNPK | 214 | 33.91 | 3 | 1955.0361 | 3 |  | 1 |  | no | 6.36e+004 | 1.34e+004 | 6.08e+004 | 3.70e+004 |
| VAPEEHPTLLTEAPLNPK | 18524 | --- | --- | 1955.0338 | 4 |  | 1 |  | no | 99.11 | 5.39 | 94.90 | 37.76 |

  

| Tags | |
| --- | --- |

  

##

## P02088

HBB1\_MOUSE
Hemoglobin subunit beta-1 OS=Mus musculus GN=Hbb-b1 PE=1 SV=2  
8
peptides

  

| Sequence | Peptide Ion | Score | Hits | Mass | Charge | Tags | Conflicts | Modifications | In quantitation | Average Normalised Abundances | | | |
| --- | --- | --- | --- | --- | --- | --- | --- | --- | --- | --- | --- | --- | --- |
|  |  |  |  |  |  |  |  |  |  | 2m2f | 4m2f | 2m4f | 4m4f |
| AAVSCLWGK | 2635 | 65.03 | 3 | 990.4949 | 2 |  | 0 |  | no | 1639.60 | 4043.80 | 923.16 | 2665.13 |
| GTFASLSELHCDK | 2299 | 90.91 | 3 | 1463.6715 | 2 |  | 1 |  | no | 3361.84 | 2869.32 | 3048.00 | 3364.46 |
| GTFASLSELHCDK | 829 | 50.77 | 3 | 1463.6704 | 3 |  | 1 |  | no | 5867.32 | 5901.61 | 6333.34 | 6252.57 |
| KVITAFNDGLNHLDSLK | 5017 | 54.79 | 3 | 1884.0089 | 3 |  | 0 |  | no | 1658.65 | 751.26 | 1537.69 | 902.67 |
| LHVDPENFR | 2189 | 41.88 | 3 | 1125.5556 | 2 |  | 1 |  | no | 2681.34 | 2011.50 | 2456.02 | 1989.18 |
| LHVDPENFR | 1887 | 38.89 | 3 | 1125.5569 | 3 |  | 1 |  | no | 1551.18 | 1065.25 | 1642.45 | 1162.82 |
| LLVVYPWTQR | 176 | 56.35 | 3 | 1273.7178 | 2 |  | 1 |  | yes | 6.19e+004 | 8.20e+004 | 9.59e+004 | 6.69e+004 |
| LLVVYPWTQR | 4996 | 28.90 | 3 | 1273.7181 | 3 |  | 1 |  | yes | 523.74 | 654.52 | 878.85 | 593.31 |
| VITAFNDGLNHLDSLK | 3846 | 97.36 | 3 | 1755.9159 | 2 |  | 0 |  | yes | 2759.01 | 1799.87 | 2530.89 | 1935.99 |
| VITAFNDGLNHLDSLK | 647 | 37.47 | 3 | 1755.9130 | 3 |  | 0 |  | yes | 1.45e+004 | 1.10e+004 | 1.55e+004 | 1.36e+004 |
| VNSDEVGGEALGR | 1299 | 123.24 | 3 | 1301.6210 | 2 |  | 0 |  | no | 6939.81 | 1.54e+004 | 5772.10 | 1.18e+004 |
| YFDSFGDLSSASAIMGNAK | 826 | 157.58 | 3 | 1979.8937 | 2 |  | 0 |  | yes | 3.84e+004 | 4.01e+004 | 4.84e+004 | 3.23e+004 |
| YFDSFGDLSSASAIMGNAK | 1824 | 60.70 | 3 | 1979.8929 | 3 |  | 0 |  | yes | 1.08e+004 | 1.04e+004 | 1.37e+004 | 8371.12 |

  

| Tags | |
| --- | --- |

  

##

## P18419

SVS4\_MOUSE
Seminal vesicle secretory protein 4 OS=Mus musculus GN=Svs4 PE=1 SV=2  
9
peptides

  

| Sequence | Peptide Ion | Score | Hits | Mass | Charge | Tags | Conflicts | Modifications | In quantitation | Average Normalised Abundances | | | |
| --- | --- | --- | --- | --- | --- | --- | --- | --- | --- | --- | --- | --- | --- |
|  |  |  |  |  |  |  |  |  |  | 2m2f | 4m2f | 2m4f | 4m4f |
| EKFLQSEETVR | 5114 | 29.58 | 3 | 1364.6925 | 3 |  | 0 |  | no | 238.87 | 930.04 | 376.31 | 1030.08 |
| FLQSEETVR | 5 | 69.54 | 3 | 1107.5553 | 2 |  | 0 |  | yes | 5.81e+005 | 1.37e+006 | 7.87e+005 | 1.18e+006 |
| FLQSEETVR | 7547 | --- | --- | 1107.5558 | 3 |  | 2 |  | no | 89.40 | 271.70 | 151.15 | 342.12 |
| FLQSEETVR | 6444 | 34.66 | 2 | 1107.5546 | 2 |  | 2 |  | no | 447.12 | 686.98 | 457.79 | 791.37 |
| FSQDALE | 41 | 48.60 | 3 | 808.3600 | 2 |  | 0 |  | no | 1.69e+005 | 2.85e+005 | 2.20e+005 | 2.67e+005 |
| FSQDALE | 1653 | 38.04 | 3 | 808.3602 | 2 |  | 0 |  | no | 1971.56 | 5242.52 | 2421.69 | 4645.85 |
| IISSSSDGSNMEGESSYSK | 104 | 49.17 | 3 | 1963.8315 | 3 |  | 0 |  | yes | 1.97e+005 | 2.57e+005 | 2.10e+005 | 2.37e+005 |
| IISSSSDGSNMEGESSYSK | 12 | 152.75 | 3 | 1963.8314 | 2 |  | 0 |  | yes | 2.18e+006 | 2.90e+006 | 2.39e+006 | 2.74e+006 |
| IISSSSDGSNMEGESSYSK | 9866 | 26.59 | 1 | 1979.8247 | 2 |  | 0 | [11] Oxidation (M) | no | 1719.22 | 5215.74 | 1561.25 | 1406.30 |
| IISSSSDGSNMEGESSYSK | 528 | 123.92 | 3 | 1979.8268 | 2 |  | 0 | [11] Oxidation (M) | no | 6.41e+004 | 9.37e+004 | 7.08e+004 | 6.90e+004 |
| IISSSSDGSNMEGESSYSK | 1314 | 65.90 | 3 | 1979.8262 | 3 |  | 0 | [11] Oxidation (M) | no | 1.15e+004 | 1.65e+004 | 1.20e+004 | 1.12e+004 |
| IISSSSDGSNMEGESSYSKR | 601 | 53.37 | 3 | 2119.9317 | 3 |  | 0 |  | no | 4.05e+004 | 5.82e+004 | 4.10e+004 | 5.14e+004 |
| SRFSQDALE | 1117 | 45.35 | 3 | 1051.4928 | 2 |  | 0 |  | no | 3842.84 | 6050.47 | 5169.19 | 6871.17 |
| SRFSQDALE | 5095 | 38.57 | 3 | 1051.4932 | 2 |  | 0 |  | no | 812.96 | 939.24 | 1199.03 | 1237.26 |
| SSEPEVFVRPQDSIGDEASEEMSSSSSSR | 190 | 26.76 | 3 | 3115.3529 | 4 |  | 0 |  | yes | 1.22e+005 | 1.43e+005 | 1.33e+005 | 1.15e+005 |
| SSEPEVFVRPQDSIGDEASEEMSSSSSSR | 9 | 87.18 | 3 | 3115.3560 | 3 |  | 0 |  | yes | 2.65e+006 | 3.28e+006 | 2.99e+006 | 2.76e+006 |
| SSEPEVFVRPQDSIGDEASEEMSSSSSSR | 6561 | --- | --- | 3115.3548 | 2 |  | 0 |  | yes | 3786.23 | 2925.59 | 3969.09 | 2854.39 |
| SSEPEVFVRPQDSIGDEASEEMSSSSSSR | 355 | 80.03 | 3 | 3131.3457 | 3 |  | 0 | [22] Oxidation (M) | no | 1.18e+005 | 1.58e+005 | 1.16e+005 | 1.12e+005 |
| SSEPEVFVRPQDSIGDEASEEMSSSSSSR | 3177 | 32.55 | 3 | 3131.3510 | 4 |  | 0 | [22] Oxidation (M) | no | 5177.07 | 5779.73 | 4613.21 | 3676.57 |

  

| Tags | |
| --- | --- |

  

##

## P60710

ACTB\_MOUSE
Actin, cytoplasmic 1 OS=Mus musculus GN=Actb PE=1 SV=1  
13
peptides

  

| Sequence | Peptide Ion | Score | Hits | Mass | Charge | Tags | Conflicts | Modifications | In quantitation | Average Normalised Abundances | | | |
| --- | --- | --- | --- | --- | --- | --- | --- | --- | --- | --- | --- | --- | --- |
|  |  |  |  |  |  |  |  |  |  | 2m2f | 4m2f | 2m4f | 4m4f |
| AGFAGDDAPR | 139 | 76.14 | 3 | 975.4377 | 2 |  | 2 |  | no | 5.79e+004 | 4.44e+004 | 5.96e+004 | 6.23e+004 |
| DLTDYLMK | 257 | 45.15 | 3 | 997.4796 | 2 |  | 3 |  | no | 2.12e+004 | 1.57e+004 | 1.93e+004 | 2.56e+004 |
| DLYANTVLSGGTTMYPGIADR | 3410 | 61.90 | 3 | 2214.0631 | 2 |  | 0 |  | yes | 7800.84 | 2880.67 | 6500.71 | 2731.37 |
| DLYANTVLSGGTTMYPGIADR | 1474 | 61.61 | 3 | 2214.0625 | 3 |  | 0 |  | yes | 1.28e+004 | 6256.58 | 1.12e+004 | 4165.38 |
| DSYVGDEAQSK | 183 | 72.06 | 3 | 1197.5149 | 2 |  | 2 |  | no | 8.98e+004 | 5.89e+004 | 9.58e+004 | 7.37e+004 |
| EITALAPSTMK | 221 | 50.88 | 3 | 1160.6106 | 2 |  | 2 |  | no | 3.86e+004 | 3.14e+004 | 3.57e+004 | 5.29e+004 |
| GYSFTTTAER | 504 | 57.03 | 3 | 1131.5188 | 2 |  | 0 |  | yes | 1.61e+004 | 1.19e+004 | 1.38e+004 | 1.40e+004 |
| IIAPPER | 164 | 32.59 | 3 | 794.4648 | 2 |  | 3 |  | no | 2.79e+004 | 1.34e+004 | 2.56e+004 | 2.12e+004 |
| IWHHTFYNELR | 3064 | 49.26 | 3 | 1514.7401 | 3 |  | 1 |  | no | 2030.34 | 280.34 | 1446.06 | 1114.07 |
| IWHHTFYNELR | 12476 | --- | --- | 1514.7420 | 4 |  | 1 |  | no | 141.69 | 2.97 | 65.60 | 44.65 |
| KDLYANTVLSGGTTMYPGIADR | 6188 | 30.11 | 3 | 2342.1566 | 3 |  | 0 |  | no | 2165.99 | 888.43 | 1948.05 | 1147.56 |
| LCYVALDFEQEMATAASSSSLEK | 9051 | 35.51 | 3 | 2549.1707 | 2 |  | 0 |  | yes | 4702.69 | 1703.27 | 3027.75 | 2307.97 |
| LCYVALDFEQEMATAASSSSLEK | 1415 | 29.26 | 3 | 2549.1665 | 3 |  | 0 |  | yes | 3.41e+004 | 9741.77 | 2.38e+004 | 7860.16 |
| RGILTLK | 1096 | 27.19 | 3 | 799.5279 | 2 |  | 3 |  | no | 1561.95 | 1158.78 | 2123.78 | 1617.99 |
| SYELPDGQVITIGNER | 653 | 64.66 | 3 | 1789.8837 | 3 |  | 3 |  | no | 1.45e+004 | 8872.48 | 1.62e+004 | 1.32e+004 |
| SYELPDGQVITIGNER | 6364 | 46.14 | 2 | 1789.8846 | 3 |  | 3 |  | no | 402.77 | 125.55 | 125.84 | 177.85 |
| SYELPDGQVITIGNER | 251 | 60.47 | 3 | 1789.8851 | 2 |  | 3 |  | no | 8.21e+004 | 4.91e+004 | 8.77e+004 | 7.29e+004 |
| VAPEEHPVLLTEAPLNPK | 555 | 58.19 | 3 | 1953.0561 | 3 |  | 1 |  | no | 2.56e+004 | 7377.57 | 2.24e+004 | 1.38e+004 |

  

| Tags | |
| --- | --- |

  

##

## P09103

PDIA1\_MOUSE
Protein disulfide-isomerase OS=Mus musculus GN=P4hb PE=1 SV=1  
12
peptides

  

| Sequence | Peptide Ion | Score | Hits | Mass | Charge | Tags | Conflicts | Modifications | In quantitation | Average Normalised Abundances | | | |
| --- | --- | --- | --- | --- | --- | --- | --- | --- | --- | --- | --- | --- | --- |
|  |  |  |  |  |  |  |  |  |  | 2m2f | 4m2f | 2m4f | 4m4f |
| ALAPEYAK | 1080 | 33.21 | 3 | 861.4573 | 2 |  | 0 |  | no | 3002.15 | 2742.81 | 3396.16 | 2950.77 |
| EADDIVNWLK | 874 | 58.79 | 3 | 1201.5970 | 2 |  | 0 |  | yes | 1.11e+004 | 1.27e+004 | 1.28e+004 | 1.09e+004 |
| HNQLPLVIEFTEQTAPK | 4766 | 55.41 | 3 | 1964.0347 | 3 |  | 0 |  | no | 3903.27 | 1261.73 | 3315.27 | 2678.95 |
| IFGGEIK | 1891 | 33.44 | 3 | 762.4277 | 2 |  | 0 |  | no | 1397.99 | 1142.20 | 1227.38 | 1215.14 |
| ILEFFGLK | 1153 | 46.34 | 3 | 965.5584 | 2 |  | 0 |  | no | 5866.59 | 4915.21 | 6438.48 | 6513.44 |
| LITLEEEMTK | 1383 | 49.08 | 3 | 1205.6208 | 2 |  | 0 |  | no | 4127.41 | 4539.16 | 3523.75 | 4219.07 |
| LLDFIK | 1199 | 40.23 | 3 | 747.4531 | 2 |  | 0 |  | no | 2151.60 | 2554.68 | 2331.82 | 2941.43 |
| NNFEGEITK | 1486 | 35.87 | 3 | 1050.4961 | 2 |  | 0 |  | no | 2671.35 | 2440.27 | 2621.08 | 2794.24 |
| THILLFLPK | 4698 | 27.19 | 3 | 1080.6692 | 3 |  | 0 |  | no | 493.71 | 250.87 | 340.33 | 354.13 |
| THILLFLPK | 3160 | 62.23 | 3 | 1080.6688 | 2 |  | 0 |  | no | 1407.80 | 841.11 | 1086.22 | 1080.83 |
| VDATEESDLAQQYGVR | 2333 | 91.87 | 3 | 1779.8278 | 2 |  | 0 |  | yes | 6314.10 | 6102.71 | 6249.53 | 5580.31 |
| VDATEESDLAQQYGVR | 3516 | 78.90 | 3 | 1779.8288 | 3 |  | 0 |  | yes | 2060.00 | 1963.74 | 2249.51 | 1657.79 |
| YKPESDELTAEK | 4542 | 35.43 | 3 | 1408.6641 | 2 |  | 0 |  | yes | 2169.41 | 1323.62 | 1779.31 | 1944.53 |
| YKPESDELTAEK | 1259 | 61.35 | 3 | 1408.6713 | 3 |  | 0 |  | yes | 5267.83 | 4799.81 | 4559.68 | 6409.14 |
| YQLDKDGVVLFK | 3517 | 46.93 | 3 | 1423.7701 | 3 |  | 0 |  | no | 1171.87 | 1128.20 | 1313.19 | 1372.53 |
| YQLDKDGVVLFK | 9804 | 43.43 | 3 | 1423.7661 | 2 |  | 0 |  | no | 254.71 | 161.94 | 185.39 | 270.93 |

  

| Tags | |
| --- | --- |

  

##

## P30933

SVS5\_MOUSE
Seminal vesicle secretory protein 5 OS=Mus musculus GN=Svs5 PE=2 SV=1  
9
peptides

  

| Sequence | Peptide Ion | Score | Hits | Mass | Charge | Tags | Conflicts | Modifications | In quantitation | Average Normalised Abundances | | | |
| --- | --- | --- | --- | --- | --- | --- | --- | --- | --- | --- | --- | --- | --- |
|  |  |  |  |  |  |  |  |  |  | 2m2f | 4m2f | 2m4f | 4m4f |
| AGGSGSGSAMEEYSVSENSWSNFK | 39 | 67.94 | 3 | 2467.0234 | 3 |  | 0 |  | yes | 8.34e+005 | 1.72e+006 | 1.01e+006 | 7.84e+005 |
| AGGSGSGSAMEEYSVSENSWSNFK | 209 | 80.34 | 3 | 2467.0263 | 2 |  | 0 |  | yes | 2.10e+005 | 4.02e+005 | 2.47e+005 | 1.90e+005 |
| AGGSGSGSAMEEYSVSENSWSNFK | 3898 | 28.47 | 3 | 2483.0219 | 2 |  | 0 | [10] Oxidation (M) | no | 5209.55 | 1.01e+004 | 5937.28 | 4478.17 |
| AGGSGSGSAMEEYSVSENSWSNFK | 543 | 65.85 | 3 | 2483.0188 | 3 |  | 0 | [10] Oxidation (M) | no | 5.08e+004 | 1.07e+005 | 5.90e+004 | 3.97e+004 |
| FSQSAEDPSSSHMGIK | 8 | 59.77 | 3 | 1706.7564 | 3 |  | 0 |  | yes | 6.64e+005 | 9.03e+005 | 6.45e+005 | 8.36e+005 |
| FSQSAEDPSSSHMGIK | 15364 | 39.36 | 3 | 1706.7558 | 3 |  | 0 |  | no | 156.71 | 216.63 | 168.84 | 144.01 |
| FSQSAEDPSSSHMGIK | 163 | 70.08 | 3 | 1706.7568 | 2 |  | 0 |  | yes | 9.41e+004 | 1.18e+005 | 8.39e+004 | 1.01e+005 |
| FSQSAEDPSSSHMGIK | 3693 | 27.92 | 1 | 1722.7513 | 3 |  | 0 | [13] Oxidation (M) | no | 1805.41 | 3410.46 | 1592.90 | 2128.21 |
| FSQSAEDPSSSHMGIK | 336 | 49.36 | 3 | 1722.7515 | 3 |  | 0 | [13] Oxidation (M) | no | 4.23e+004 | 6.31e+004 | 4.40e+004 | 4.30e+004 |
| FSQSAEDPSSSHMGIK | 5199 | 26.54 | 2 | 1722.7523 | 2 |  | 0 | [13] Oxidation (M) | no | 2290.07 | 2659.45 | 1729.07 | 1615.00 |
| GSQAGGGMSSFK | 7 | 67.23 | 3 | 1112.4915 | 2 |  | 0 |  | yes | 5.40e+005 | 9.89e+005 | 6.48e+005 | 7.92e+005 |
| GSQAGGGMSSFK | 9725 | 43.71 | 3 | 1128.4868 | 2 |  | 0 | [8] Oxidation (M) | no | 1227.87 | 2626.99 | 1322.60 | 1935.57 |
| HPSSISSESFHEESSSSSEMSSSGGHFGLK | 2255 | 61.60 | 3 | 3124.3330 | 3 |  | 0 |  | no | 9783.10 | 6160.01 | 9361.68 | 4877.19 |
| HPSSISSESFHEESSSSSEMSSSGGHFGLK | 146 | 52.34 | 3 | 3124.3310 | 4 |  | 0 |  | no | 2.27e+005 | 1.69e+005 | 2.02e+005 | 1.22e+005 |
| HPSSISSESFHEESSSSSEMSSSGGHFGLK | 2017 | 26.74 | 3 | 3140.3248 | 4 |  | 0 | [20] Oxidation (M) | no | 9323.94 | 1.06e+004 | 1.02e+004 | 5774.26 |
| SKHPSSISSESFHEESSSSSEMSSSGGHFGLK | 1047 | 41.68 | 3 | 3339.4584 | 4 |  | 0 |  | no | 2.67e+004 | 1.68e+004 | 2.85e+004 | 1.23e+004 |

  

| Tags | |
| --- | --- |

  

##

## P11679

K2C8\_MOUSE
Keratin, type II cytoskeletal 8 OS=Mus musculus GN=Krt8 PE=1 SV=4  
11
peptides

  

| Sequence | Peptide Ion | Score | Hits | Mass | Charge | Tags | Conflicts | Modifications | In quantitation | Average Normalised Abundances | | | |
| --- | --- | --- | --- | --- | --- | --- | --- | --- | --- | --- | --- | --- | --- |
|  |  |  |  |  |  |  |  |  |  | 2m2f | 4m2f | 2m4f | 4m4f |
| AQYEDIANR | 3103 | 49.96 | 3 | 1078.5020 | 2 |  | 1 |  | no | 1340.89 | 1091.66 | 1127.04 | 1121.65 |
| ASLEAAIADAEQR | 4167 | 80.91 | 3 | 1343.6666 | 2 |  | 0 |  | no | 2153.08 | 3559.43 | 2826.28 | 2787.18 |
| LALDIEITTYR | 8089 | 75.25 | 3 | 1306.7130 | 2 |  | 0 |  | no | 662.40 | 394.97 | 458.19 | 188.65 |
| LEAELGNMQGLVEDFK | 7091 | 30.78 | 3 | 1791.8718 | 2 |  | 0 |  | no | 2377.31 | 1122.17 | 2142.81 | 1386.05 |
| LEAELGNMQGLVEDFK | 16515 | 41.08 | 2 | 1791.8713 | 3 |  | 0 |  | no | 253.33 | 70.07 | 233.64 | 140.89 |
| LEGLTDEINFLR | 1490 | 70.89 | 3 | 1418.7393 | 2 |  | 0 |  | yes | 8155.56 | 6742.37 | 8194.55 | 7689.10 |
| LLEGEESR | 1786 | 53.36 | 3 | 931.4603 | 2 |  | 2 |  | no | 2674.46 | 1761.54 | 2395.89 | 2386.46 |
| LQAEIEALK | 2569 | 45.99 | 3 | 1013.5744 | 2 |  | 0 |  | no | 2925.09 | 3051.35 | 2934.49 | 3403.06 |
| SLDMDGIIAEVR | 4150 | 96.00 | 3 | 1317.6636 | 2 |  | 0 |  | no | 2022.07 | 2207.70 | 2282.82 | 2019.16 |
| TEMENEFVLIK | 2825 | 73.35 | 3 | 1351.6686 | 2 |  | 0 |  | yes | 3608.60 | 4165.87 | 3390.37 | 3217.01 |
| WSLLQQQK | 4114 | 37.85 | 3 | 1029.5600 | 2 |  | 0 |  | no | 849.50 | 639.18 | 648.98 | 854.10 |
| YEELQTLAGK | 2361 | 78.59 | 3 | 1150.5859 | 2 |  | 0 |  | yes | 3870.45 | 3110.23 | 4806.35 | 4105.01 |

  

| Tags | |
| --- | --- |

  

##

## O08709

PRDX6\_MOUSE
Peroxiredoxin-6 OS=Mus musculus GN=Prdx6 PE=1 SV=3  
13
peptides

  

| Sequence | Peptide Ion | Score | Hits | Mass | Charge | Tags | Conflicts | Modifications | In quantitation | Average Normalised Abundances | | | |
| --- | --- | --- | --- | --- | --- | --- | --- | --- | --- | --- | --- | --- | --- |
|  |  |  |  |  |  |  |  |  |  | 2m2f | 4m2f | 2m4f | 4m4f |
| DINAYNGETPTEK | 2691 | 59.30 | 3 | 1450.6569 | 2 |  | 0 |  | no | 5256.00 | 3699.86 | 3752.78 | 3369.49 |
| DLAILLGMLDPVEK | 5926 | 102.28 | 3 | 1525.8422 | 2 |  | 0 |  | yes | 1.06e+004 | 4125.25 | 7790.95 | 3845.73 |
| DLAILLGMLDPVEK | 40960 | 26.80 | 1 | 1541.8362 | 2 |  | 0 | [8] Oxidation (M) | no | 128.68 | 14.62 | 51.21 | 13.36 |
| GESVMVVPTLSEEEAK | 8445 | 26.61 | 3 | 1703.8278 | 2 |  | 0 |  | no | 1035.20 | 730.42 | 752.91 | 643.52 |
| KGESVMVVPTLSEEEAK | 5071 | 51.97 | 3 | 1831.9237 | 3 |  | 0 |  | no | 1730.19 | 925.98 | 1190.69 | 1062.84 |
| LIALSIDSVEDHLAWSK | 23759 | --- | --- | 1895.9994 | 2 |  | 0 |  | no | 301.29 | 40.83 | 219.91 | 119.12 |
| LIALSIDSVEDHLAWSK | 4827 | 29.86 | 3 | 1895.9957 | 3 |  | 0 |  | no | 3068.96 | 1627.56 | 2733.99 | 1399.32 |
| LPFPIIDDK | 945 | 48.25 | 3 | 1056.5841 | 2 |  | 0 |  | yes | 9039.28 | 6830.78 | 8181.13 | 7512.62 |
| LSILYPATTGR | 1366 | 40.99 | 3 | 1190.6639 | 2 |  | 0 |  | yes | 6475.50 | 6704.47 | 5942.70 | 5892.01 |
| NFDEILR | 1768 | 41.23 | 3 | 905.4603 | 2 |  | 0 |  | no | 1708.34 | 1615.60 | 1481.62 | 1558.24 |
| PGGLLLGDEAPNFEANTTIGR | 6597 | 71.75 | 3 | 2141.0756 | 2 |  | 0 |  | no | 2760.31 | 905.94 | 1738.31 | 1198.97 |
| PGGLLLGDEAPNFEANTTIGR | 2458 | 73.38 | 3 | 2141.0747 | 3 |  | 0 |  | no | 7080.01 | 2845.69 | 4667.87 | 2959.36 |
| VVDSLQLTGTKPVATPVDWK | 1749 | 64.99 | 3 | 2153.1728 | 3 |  | 0 |  | no | 7709.89 | 3880.36 | 5739.81 | 4839.75 |
| VVFIFGPDK | 1740 | 53.58 | 3 | 1020.5635 | 2 |  | 0 |  | no | 3447.78 | 2363.52 | 3116.83 | 2811.27 |
| VVFIFGPDKK | 7081 | --- | --- | 1148.6554 | 2 |  | 0 |  | no | 587.66 | 436.77 | 348.68 | 334.10 |
| VVFIFGPDKK | 3512 | 33.09 | 3 | 1148.6598 | 3 |  | 0 |  | no | 968.19 | 675.69 | 721.79 | 683.49 |

  

| Tags | |
| --- | --- |

  

##

## P81117

NUCB2\_MOUSE
Nucleobindin-2 OS=Mus musculus GN=Nucb2 PE=1 SV=2  
13
peptides

  

| Sequence | Peptide Ion | Score | Hits | Mass | Charge | Tags | Conflicts | Modifications | In quantitation | Average Normalised Abundances | | | |
| --- | --- | --- | --- | --- | --- | --- | --- | --- | --- | --- | --- | --- | --- |
|  |  |  |  |  |  |  |  |  |  | 2m2f | 4m2f | 2m4f | 4m4f |
| AATADLEQYDR | 666 | 75.83 | 3 | 1251.5719 | 2 |  | 0 |  | no | 9732.48 | 1.40e+004 | 1.10e+004 | 1.42e+004 |
| ADIEEIR | 943 | 53.55 | 3 | 844.4289 | 2 |  | 0 |  | no | 4251.85 | 6208.15 | 4387.98 | 5712.34 |
| DLDMLIK | 1650 | 48.99 | 3 | 846.4519 | 2 |  | 0 |  | no | 1836.82 | 3204.32 | 2170.43 | 3119.60 |
| EHVMSEIDNNKDR | 12533 | --- | --- | 1585.7148 | 4 |  | 0 |  | no | 124.38 | 81.67 | 93.94 | 112.48 |
| EHVMSEIDNNKDR | 3820 | 39.10 | 3 | 1585.7142 | 3 |  | 0 |  | no | 1357.44 | 1360.28 | 1176.72 | 1558.54 |
| EVWEETDGLDPNDFDPK | 2465 | 69.67 | 3 | 2004.8598 | 2 |  | 0 |  | yes | 1.02e+004 | 1.60e+004 | 1.15e+004 | 1.13e+004 |
| EVWEETDGLDPNDFDPK | 8030 | --- | --- | 2004.8647 | 3 |  | 0 |  | yes | 859.32 | 780.74 | 852.52 | 545.82 |
| IEPPDTGLYYDEYLK | 833 | 55.90 | 3 | 1814.8628 | 2 |  | 0 |  | yes | 3.04e+004 | 4.20e+004 | 4.05e+004 | 3.66e+004 |
| IEPPDTGLYYDEYLK | 3426 | 50.43 | 3 | 1814.8628 | 3 |  | 0 |  | yes | 2637.17 | 3575.42 | 4051.59 | 3185.89 |
| KLQQGIAPSGPAGELK | 6670 | 29.75 | 3 | 1592.8861 | 3 |  | 0 |  | no | 381.91 | 307.13 | 423.95 | 357.26 |
| LDALQDTGMNHHLLLK | 6843 | 26.71 | 3 | 1817.9450 | 3 |  | 0 |  | no | 1105.68 | 620.13 | 933.41 | 790.86 |
| LDALQDTGMNHHLLLK | 6350 | --- | --- | 1817.9451 | 4 |  | 0 |  | no | 503.37 | 271.75 | 466.73 | 313.71 |
| LHDVNNDGFLDEQELEALFTR | 5269 | 32.79 | 3 | 2474.1728 | 3 |  | 0 |  | no | 5084.33 | 1958.43 | 4185.79 | 3358.33 |
| LQQGIAPSGPAGELK | 2486 | 57.72 | 3 | 1464.7939 | 2 |  | 0 |  | no | 3591.21 | 3736.49 | 3349.36 | 3577.92 |
| LSQELDLVSHK | 894 | 44.22 | 3 | 1267.6774 | 3 |  | 0 |  | no | 6287.54 | 1.17e+004 | 7036.52 | 1.41e+004 |
| LSQELDLVSHK | 2904 | --- | --- | 1267.6762 | 2 |  | 0 |  | no | 2407.75 | 3233.24 | 2400.09 | 2985.93 |
| LVTLEEFLR | 489 | 55.70 | 3 | 1118.6331 | 2 |  | 0 |  | yes | 2.26e+004 | 2.52e+004 | 2.31e+004 | 2.46e+004 |
| VYNPQNAEDDMIEMEEER | 5345 | 35.98 | 1 | 2210.9085 | 3 |  | 0 |  | no | 2093.07 | 1977.33 | 2192.15 | 1613.19 |
| VYNPQNAEDDMIEMEEER | 8221 | --- | --- | 2210.9118 | 2 |  | 0 |  | no | 1924.44 | 1373.47 | 1298.87 | 1162.49 |

  

| Tags | |
| --- | --- |

  

##

## Q91YQ5

RPN1\_MOUSE
Dolichyl-diphosphooligosaccharide--protein glycosyltransferase subunit 1 OS=Mus
musculus GN=Rpn1 PE=2 SV=1  
11 peptides

  

| Sequence | Peptide Ion | Score | Hits | Mass | Charge | Tags | Conflicts | Modifications | In quantitation | Average Normalised Abundances | | | |
| --- | --- | --- | --- | --- | --- | --- | --- | --- | --- | --- | --- | --- | --- |
|  |  |  |  |  |  |  |  |  |  | 2m2f | 4m2f | 2m4f | 4m4f |
| ASSFVLALEPELESR | 3645 | 67.97 | 3 | 1646.8423 | 2 |  | 0 |  | yes | 5031.54 | 3296.65 | 4377.69 | 3995.17 |
| AVTSEIAVLQSR | 3124 | 92.72 | 3 | 1272.7028 | 2 |  | 0 |  | no | 1661.25 | 1426.50 | 1496.33 | 1582.39 |
| FPLFGGWK | 8503 | 25.87 | 3 | 950.5010 | 2 |  | 0 |  | no | 884.30 | 404.14 | 774.67 | 805.42 |
| FVDHVFDEQVIDSLTVK | 6046 | 36.88 | 3 | 1990.0080 | 3 |  | 0 |  | no | 1960.23 | 775.52 | 1175.10 | 747.91 |
| GEDEEDNNLEVR | 3053 | 85.01 | 3 | 1417.5999 | 2 |  | 0 |  | no | 2219.71 | 1899.86 | 1889.84 | 1996.74 |
| IDHILDAL | 4524 | 30.68 | 3 | 908.4969 | 2 |  | 0 |  | no | 561.55 | 640.67 | 601.04 | 628.58 |
| NIQVDSPYDISR | 3832 | 62.25 | 3 | 1405.6820 | 2 |  | 0 |  | no | 2300.61 | 2498.29 | 1965.92 | 1426.12 |
| SEDVLDYGPFK | 4320 | 55.41 | 2 | 1268.5918 | 2 |  | 0 |  | yes | 2181.98 | 2485.38 | 2251.34 | 2306.23 |
| TEGSDLCDR | 7467 | 59.36 | 3 | 1051.4232 | 2 |  | 0 |  | no | 791.80 | 395.11 | 595.11 | 461.62 |
| TILPAAAQDVYYR | 3058 | 88.16 | 3 | 1479.7689 | 2 |  | 0 |  | yes | 3175.62 | 3677.56 | 2749.02 | 3497.90 |
| VTAEVVLVHPGGGSTSR | 5197 | 69.26 | 3 | 1664.8831 | 3 |  | 0 |  | no | 987.86 | 468.65 | 733.15 | 856.45 |

  

| Tags | |
| --- | --- |

  

##

## P63017

HSP7C\_MOUSE
Heat shock cognate 71 kDa protein OS=Mus musculus GN=Hspa8 PE=1 SV=1  
10
peptides

  

| Sequence | Peptide Ion | Score | Hits | Mass | Charge | Tags | Conflicts | Modifications | In quantitation | Average Normalised Abundances | | | |
| --- | --- | --- | --- | --- | --- | --- | --- | --- | --- | --- | --- | --- | --- |
|  |  |  |  |  |  |  |  |  |  | 2m2f | 4m2f | 2m4f | 4m4f |
| FDDAVVQSDMK | 6859 | 50.58 | 3 | 1253.5593 | 2 |  | 0 |  | no | 694.87 | 643.78 | 626.72 | 553.35 |
| FEELNADLFR | 2004 | 63.70 | 3 | 1252.6080 | 2 |  | 0 |  | yes | 4030.28 | 4067.04 | 4423.14 | 3903.95 |
| GTLDPVEK | 5930 | 25.89 | 1 | 857.4488 | 2 |  | 0 |  | no | 579.74 | 306.63 | 361.80 | 228.44 |
| IINEPTAAAIAYGLDK | 2500 | 90.89 | 3 | 1658.8861 | 3 |  | 1 |  | no | 2329.95 | 1547.30 | 2056.25 | 2056.09 |
| IINEPTAAAIAYGLDK | 1209 | 111.89 | 3 | 1658.8880 | 2 |  | 1 |  | no | 1.01e+004 | 6877.88 | 8351.44 | 8660.21 |
| LLQDFFNGK | 4862 | 53.74 | 3 | 1080.5621 | 2 |  | 0 |  | no | 833.27 | 668.44 | 757.79 | 612.82 |
| NQTAEKEEFEHQQK | 8154 | 34.18 | 3 | 1744.8000 | 3 |  | 0 |  | no | 705.48 | 333.17 | 590.88 | 688.55 |
| NQTAEKEEFEHQQK | 9033 | --- | --- | 1744.7961 | 4 |  | 0 |  | no | 379.09 | 131.62 | 349.68 | 387.16 |
| SFYPEEVSSMVLTK | 5200 | 48.65 | 3 | 1615.7802 | 2 |  | 0 |  | no | 3122.50 | 2354.59 | 2987.26 | 2283.34 |
| SFYPEEVSSMVLTK | 14610 | --- | --- | 1615.7791 | 3 |  | 0 |  | no | 257.48 | 108.43 | 181.34 | 144.44 |
| SINPDEAVAYGAAVQAAILSGDK | 4086 | 44.01 | 3 | 2259.1375 | 3 |  | 0 |  | yes | 7725.09 | 2764.82 | 5883.35 | 3289.18 |
| TTPSYVAFTDTER | 1603 | 65.20 | 3 | 1486.6941 | 2 |  | 1 |  | yes | 6267.03 | 4584.84 | 5135.16 | 4726.15 |
| VEIIANDQGNR | 1006 | 60.83 | 3 | 1227.6195 | 2 |  | 2 |  | no | 7512.51 | 4609.63 | 5361.71 | 5790.14 |

  

| Tags | |
| --- | --- |

  

##

## P58774

TPM2\_MOUSE
Tropomyosin beta chain OS=Mus musculus GN=Tpm2 PE=1 SV=1  
9
peptides

  

| Sequence | Peptide Ion | Score | Hits | Mass | Charge | Tags | Conflicts | Modifications | In quantitation | Average Normalised Abundances | | | |
| --- | --- | --- | --- | --- | --- | --- | --- | --- | --- | --- | --- | --- | --- |
|  |  |  |  |  |  |  |  |  |  | 2m2f | 4m2f | 2m4f | 4m4f |
| ATDAEADVASLNR | 4296 | --- | --- | 1331.6311 | 3 |  | 1 |  | yes | 663.01 | 307.09 | 624.75 | 798.12 |
| ATDAEADVASLNR | 640 | 94.12 | 3 | 1331.6319 | 2 |  | 1 |  | yes | 1.32e+004 | 8278.47 | 1.31e+004 | 1.41e+004 |
| EDKYEEEIK | 834 | 25.21 | 3 | 1181.5449 | 3 |  | 1 |  | no | 4541.60 | 2412.08 | 3999.22 | 4069.56 |
| EDKYEEEIK | 1785 | 29.81 | 1 | 1181.5435 | 2 |  | 1 |  | no | 5666.20 | 3678.47 | 3897.89 | 4865.15 |
| IQLVEEELDR | 273 | 52.00 | 3 | 1242.6466 | 2 |  | 1 |  | yes | 2.98e+004 | 1.17e+004 | 2.33e+004 | 2.58e+004 |
| KLVILEGELER | 7168 | 71.10 | 3 | 1297.7602 | 3 |  | 0 |  | no | 242.67 | 69.82 | 376.65 | 234.35 |
| LDKENAIDR | 3458 | 44.44 | 3 | 1072.5505 | 2 |  | 1 |  | no | 944.03 | 298.15 | 805.67 | 692.69 |
| LDKENAIDR | 1508 | 34.16 | 3 | 1072.5516 | 3 |  | 1 |  | no | 1441.75 | 649.94 | 1421.62 | 1358.49 |
| LDKENAIDR | 3372 | 57.33 | 3 | 1072.5505 | 2 |  | 1 |  | no | 1869.75 | 644.43 | 1411.10 | 1185.64 |
| LDKENAIDR | 1391 | 43.38 | 3 | 1072.5514 | 3 |  | 1 |  | no | 2725.63 | 1259.96 | 2434.40 | 2162.06 |
| LKGTEDEVEK | 4341 | 48.57 | 3 | 1146.5761 | 2 |  | 1 |  | no | 1586.56 | 590.36 | 1416.15 | 1302.87 |
| LKGTEDEVEK | 2218 | 44.68 | 3 | 1146.5769 | 3 |  | 1 |  | no | 2190.17 | 914.72 | 2093.25 | 1812.85 |
| LVILEGELER | 619 | 67.21 | 3 | 1169.6664 | 2 |  | 0 |  | yes | 1.01e+004 | 5772.46 | 1.08e+004 | 7223.64 |
| MELQEMQLK | 1591 | 58.85 | 3 | 1148.5591 | 2 |  | 0 |  | no | 3324.79 | 2162.92 | 2995.09 | 3696.01 |
| QLEEEQQALQK | 4732 | 54.12 | 3 | 1342.6652 | 2 |  | 0 |  | no | 1192.21 | 747.20 | 1044.68 | 1345.59 |

  

| Tags | |
| --- | --- |

  

##

## Q07235

GDN\_MOUSE
Glia-derived nexin OS=Mus musculus GN=Serpine2 PE=2 SV=2  
10
peptides

  

| Sequence | Peptide Ion | Score | Hits | Mass | Charge | Tags | Conflicts | Modifications | In quantitation | Average Normalised Abundances | | | |
| --- | --- | --- | --- | --- | --- | --- | --- | --- | --- | --- | --- | --- | --- |
|  |  |  |  |  |  |  |  |  |  | 2m2f | 4m2f | 2m4f | 4m4f |
| ASAATTAILIAR | 1207 | 92.58 | 3 | 1157.6749 | 2 |  | 0 |  | yes | 4985.31 | 8737.35 | 6092.21 | 7799.47 |
| DIVTVANAVFLR | 8206 | --- | --- | 1316.7449 | 3 |  | 0 |  | yes | 467.14 | 443.06 | 460.61 | 285.73 |
| DIVTVANAVFLR | 1242 | 77.55 | 3 | 1316.7449 | 2 |  | 0 |  | yes | 1.32e+004 | 1.39e+004 | 1.37e+004 | 9987.59 |
| FTAVAQTDLK | 1133 | 65.96 | 3 | 1092.5801 | 2 |  | 0 |  | no | 5096.61 | 5660.91 | 5413.10 | 6735.76 |
| GMIDNLLSPNLIDGALTR | 5587 | 67.21 | 2 | 1912.0093 | 2 |  | 0 |  | no | 7244.01 | 3931.01 | 5193.00 | 1393.19 |
| GMIDNLLSPNLIDGALTR | 6996 | --- | --- | 1912.0074 | 3 |  | 0 |  | no | 2638.64 | 1287.43 | 1804.64 | 366.17 |
| HNPTGAILFLGQVNKP | 5551 | 59.27 | 3 | 1704.9278 | 3 |  | 0 |  | no | 1030.47 | 723.69 | 1099.79 | 983.40 |
| LVLVNAVYFK | 1032 | 78.03 | 3 | 1164.6902 | 2 |  | 0 |  | yes | 6372.64 | 1.04e+004 | 8844.75 | 7130.52 |
| MEVPFAVR | 1512 | 38.96 | 3 | 947.4908 | 2 |  | 0 |  | no | 2134.50 | 3452.25 | 2131.42 | 3286.68 |
| SESLHVSHILQK | 3257 | 48.52 | 3 | 1376.7321 | 3 |  | 0 |  | no | 1300.36 | 2302.29 | 1288.24 | 3318.40 |
| SYQVPMLAQLSVFR | 5453 | 41.07 | 3 | 1637.8601 | 2 |  | 0 |  | no | 7204.44 | 3996.12 | 5932.73 | 4385.49 |
| SYQVPMLAQLSVFR | 17474 | 33.86 | 3 | 1637.8593 | 3 |  | 0 |  | no | 485.70 | 130.94 | 283.24 | 165.06 |
| TIDSWMNTMVPK | 4394 | 35.18 | 3 | 1421.6713 | 2 |  | 0 |  | no | 3862.38 | 4171.92 | 3071.22 | 2778.88 |

  

| Tags | |
| --- | --- |

  

##

## Q8VDD5

MYH9\_MOUSE
Myosin-9 OS=Mus musculus GN=Myh9 PE=1 SV=4  
11 peptides

  

| Sequence | Peptide Ion | Score | Hits | Mass | Charge | Tags | Conflicts | Modifications | In quantitation | Average Normalised Abundances | | | |
| --- | --- | --- | --- | --- | --- | --- | --- | --- | --- | --- | --- | --- | --- |
|  |  |  |  |  |  |  |  |  |  | 2m2f | 4m2f | 2m4f | 4m4f |
| DLGEELEALK | 3622 | 44.74 | 3 | 1115.5715 | 2 |  | 1 |  | no | 1520.67 | 496.17 | 1599.84 | 914.86 |
| EDQSILCTGESGAGK | 2805 | 68.63 | 3 | 1550.6829 | 2 |  | 2 |  | no | 4611.77 | 2706.44 | 4055.32 | 4123.34 |
| EQADFAIEALAK | 1716 | 65.04 | 3 | 1304.6612 | 2 |  | 1 |  | yes | 6797.83 | 6257.95 | 5448.91 | 5039.11 |
| FDQLLAEEK | 4779 | 58.34 | 3 | 1091.5412 | 2 |  | 2 |  | no | 836.23 | 356.40 | 579.35 | 531.05 |
| IAQLEEQLDNETK | 16564 | 27.36 | 2 | 1529.7560 | 2 |  | 0 |  | yes | 334.38 | 369.96 | 266.93 | 738.40 |
| KFDQLLAEEK | 9347 | 42.12 | 3 | 1219.6444 | 3 |  | 2 |  | no | 225.59 | 33.67 | 268.76 | 131.26 |
| LQQELDDLLVDLDHQR | 15453 | 28.54 | 1 | 1948.9961 | 3 |  | 0 |  | yes | 401.04 | 73.78 | 217.26 | 123.93 |
| NTDQASMPDNTAAQK | 6747 | 80.30 | 3 | 1590.6923 | 2 |  | 1 |  | no | 1604.43 | 648.84 | 1575.83 | 951.88 |
| NWQWWR | 7475 | 29.88 | 3 | 974.4509 | 2 |  | 1 |  | no | 259.23 | 35.40 | 269.30 | 207.07 |
| QLLQANPILEAFGNAK | 10097 | 69.31 | 3 | 1725.9414 | 2 |  | 1 |  | no | 1456.18 | 102.49 | 1479.00 | 375.24 |
| TQLEELEDELQATEDAK | 5214 | 68.03 | 3 | 1960.9116 | 2 |  | 1 |  | no | 2891.89 | 757.07 | 2819.56 | 995.69 |
| TQLEELEDELQATEDAK | 3799 | 53.55 | 3 | 1960.9107 | 3 |  | 1 |  | no | 2134.98 | 650.25 | 2643.43 | 1006.97 |

  

| Tags | |
| --- | --- |

  

##

## P08113

ENPL\_MOUSE
Endoplasmin OS=Mus musculus GN=Hsp90b1 PE=1 SV=2  
10 peptides

  

| Sequence | Peptide Ion | Score | Hits | Mass | Charge | Tags | Conflicts | Modifications | In quantitation | Average Normalised Abundances | | | |
| --- | --- | --- | --- | --- | --- | --- | --- | --- | --- | --- | --- | --- | --- |
|  |  |  |  |  |  |  |  |  |  | 2m2f | 4m2f | 2m4f | 4m4f |
| DISTNYYASQK | 4505 | 72.59 | 3 | 1288.5907 | 2 |  | 0 |  | no | 1497.51 | 1669.85 | 1368.38 | 1852.20 |
| EAESSPFVER | 4960 | 41.49 | 3 | 1149.5296 | 2 |  | 0 |  | no | 1510.43 | 2989.16 | 1385.04 | 2170.84 |
| EEASDYLELDTIK | 2866 | 84.90 | 2 | 1524.7214 | 2 |  | 0 |  | yes | 6477.84 | 5867.86 | 6033.56 | 5037.63 |
| EFEPLLNWMK | 5181 | 35.66 | 3 | 1305.6419 | 2 |  | 0 |  | no | 2315.17 | 1243.36 | 1892.01 | 2074.80 |
| GVVDSDDLPLNVSR | 2009 | 114.10 | 3 | 1484.7468 | 2 |  | 0 |  | yes | 4152.84 | 3320.91 | 3700.99 | 4121.49 |
| LGVIEDHSNR | 4239 | 33.73 | 3 | 1138.5727 | 3 |  | 0 |  | no | 618.33 | 346.59 | 420.09 | 557.45 |
| SGYLLPDTK | 4425 | 62.32 | 3 | 992.5172 | 2 |  | 0 |  | no | 806.74 | 689.51 | 707.88 | 825.33 |
| SILFVPTSAPR | 2436 | 56.63 | 3 | 1186.6707 | 2 |  | 0 |  | yes | 2102.32 | 2388.69 | 2443.03 | 2926.42 |
| TETVEEPLEEDEAAKEEK | 6576 | 26.02 | 1 | 2074.9413 | 3 |  | 0 |  | no | 1313.52 | 823.56 | 1148.04 | 992.68 |
| YSQFINFPIYVWSSK | 21146 | 25.21 | 3 | 1877.9348 | 3 |  | 0 |  | no | 361.57 | 8.77 | 212.32 | 136.64 |
| YSQFINFPIYVWSSK | 12187 | 49.02 | 3 | 1877.9341 | 2 |  | 0 |  | no | 1936.39 | 155.38 | 1064.86 | 825.82 |

  

| Tags | |
| --- | --- |

  

##

## P58771

TPM1\_MOUSE
Tropomyosin alpha-1 chain OS=Mus musculus GN=Tpm1 PE=1 SV=1  
8
peptides

  

| Sequence | Peptide Ion | Score | Hits | Mass | Charge | Tags | Conflicts | Modifications | In quantitation | Average Normalised Abundances | | | |
| --- | --- | --- | --- | --- | --- | --- | --- | --- | --- | --- | --- | --- | --- |
|  |  |  |  |  |  |  |  |  |  | 2m2f | 4m2f | 2m4f | 4m4f |
| ATDAEADVASLNR | 640 | 94.12 | 3 | 1331.6319 | 2 |  | 1 |  | yes | 1.32e+004 | 8278.47 | 1.31e+004 | 1.41e+004 |
| ATDAEADVASLNR | 4296 | --- | --- | 1331.6311 | 3 |  | 1 |  | yes | 663.01 | 307.09 | 624.75 | 798.12 |
| EDKYEEEIK | 834 | 25.21 | 3 | 1181.5449 | 3 |  | 1 |  | no | 4541.60 | 2412.08 | 3999.22 | 4069.56 |
| EDKYEEEIK | 1785 | 29.81 | 1 | 1181.5435 | 2 |  | 1 |  | no | 5666.20 | 3678.47 | 3897.89 | 4865.15 |
| IQLVEEELDR | 273 | 52.00 | 3 | 1242.6466 | 2 |  | 1 |  | yes | 2.98e+004 | 1.17e+004 | 2.33e+004 | 2.58e+004 |
| KLVIIESDLER | 5254 | 63.03 | 3 | 1313.7536 | 2 |  | 0 |  | no | 1183.52 | 517.56 | 958.65 | 976.30 |
| KLVIIESDLER | 3577 | 35.57 | 3 | 1313.7541 | 3 |  | 0 |  | no | 815.60 | 610.17 | 1052.77 | 1060.04 |
| LDKENALDR | 3372 | 57.33 | 3 | 1072.5505 | 2 |  | 1 |  | no | 1869.75 | 644.43 | 1411.10 | 1185.64 |
| LDKENALDR | 1391 | 43.38 | 3 | 1072.5514 | 3 |  | 1 |  | no | 2725.63 | 1259.96 | 2434.40 | 2162.06 |
| LDKENALDR | 3458 | 44.44 | 3 | 1072.5505 | 2 |  | 1 |  | no | 944.03 | 298.15 | 805.67 | 692.69 |
| LDKENALDR | 1508 | 34.16 | 3 | 1072.5516 | 3 |  | 1 |  | no | 1441.75 | 649.94 | 1421.62 | 1358.49 |
| LKGTEDELDK | 4341 | 37.20 | 3 | 1146.5761 | 2 |  | 1 |  | no | 1586.56 | 590.36 | 1416.15 | 1302.87 |
| LKGTEDELDK | 2218 | 28.97 | 3 | 1146.5769 | 3 |  | 1 |  | no | 2190.17 | 914.72 | 2093.25 | 1812.85 |
| LVIIESDLER | 585 | 59.52 | 3 | 1185.6596 | 2 |  | 0 |  | yes | 1.26e+004 | 9123.89 | 1.03e+004 | 1.30e+004 |
| MEIQEIQLK | 1013 | 61.07 | 3 | 1130.6000 | 2 |  | 0 |  | no | 5622.86 | 3029.71 | 5070.94 | 6510.10 |

  

| Tags | |
| --- | --- |

  

##

## Q9D819

IPYR\_MOUSE
Inorganic pyrophosphatase OS=Mus musculus GN=Ppa1 PE=1 SV=1  
9
peptides

  

| Sequence | Peptide Ion | Score | Hits | Mass | Charge | Tags | Conflicts | Modifications | In quantitation | Average Normalised Abundances | | | |
| --- | --- | --- | --- | --- | --- | --- | --- | --- | --- | --- | --- | --- | --- |
|  |  |  |  |  |  |  |  |  |  | 2m2f | 4m2f | 2m4f | 4m4f |
| AAPFTLEYR | 3527 | 44.34 | 3 | 1066.5435 | 2 |  | 0 |  | no | 1343.39 | 2115.60 | 1237.50 | 1654.62 |
| DFAVDIIK | 3880 | 38.10 | 3 | 919.5012 | 2 |  | 0 |  | no | 674.38 | 1408.66 | 673.77 | 953.93 |
| GISCMNTTVSESPFK | 5282 | 119.44 | 3 | 1656.7465 | 2 |  | 0 |  | no | 2171.58 | 2245.57 | 1327.63 | 1577.95 |
| HTGCCGDNDPIDVCEIGSK | 4891 | 45.73 | 3 | 2132.8552 | 3 |  | 0 |  | yes | 2435.09 | 1577.94 | 1674.11 | 2416.74 |
| LKPGYLEATVDWFR | 11845 | 38.91 | 3 | 1693.8816 | 3 |  | 0 |  | no | 263.46 | 138.59 | 139.25 | 157.17 |
| VIAINVDDPDAANYK | 6855 | 53.84 | 3 | 1616.8037 | 3 |  | 0 |  | yes | 571.30 | 954.29 | 518.01 | 661.86 |
| VIAINVDDPDAANYK | 1843 | 96.81 | 3 | 1616.8042 | 2 |  | 0 |  | yes | 6492.98 | 1.09e+004 | 5913.77 | 8995.51 |
| VLGILAMIDEGETDWK | 12037 | 47.49 | 3 | 1788.8973 | 2 |  | 0 |  | no | 825.92 | 421.80 | 874.26 | 471.27 |
| VPDGKPENEFAFNAEFK | 9030 | 32.27 | 3 | 1937.9159 | 3 |  | 0 |  | no | 886.97 | 312.17 | 461.16 | 588.11 |
| YVANLFPYK | 3624 | 41.13 | 3 | 1113.5853 | 2 |  | 0 |  | yes | 1588.05 | 2862.68 | 1641.92 | 2341.99 |

  

| Tags | |
| --- | --- |

  

##

## Q7TPR4

ACTN1\_MOUSE
Alpha-actinin-1 OS=Mus musculus GN=Actn1 PE=2 SV=1  
9
peptides

  

| Sequence | Peptide Ion | Score | Hits | Mass | Charge | Tags | Conflicts | Modifications | In quantitation | Average Normalised Abundances | | | |
| --- | --- | --- | --- | --- | --- | --- | --- | --- | --- | --- | --- | --- | --- |
|  |  |  |  |  |  |  |  |  |  | 2m2f | 4m2f | 2m4f | 4m4f |
| DLLLDPAWEK | 7924 | 39.91 | 3 | 1198.6230 | 2 |  | 0 |  | no | 636.36 | 185.86 | 476.63 | 265.86 |
| EGLLLWCQR | 10173 | 33.27 | 3 | 1173.5958 | 2 |  | 0 |  | no | 326.76 | 176.86 | 279.74 | 221.77 |
| FAIQDISVEETSAK | 5502 | 67.14 | 3 | 1536.7669 | 2 |  | 0 |  | yes | 1464.65 | 1187.90 | 1407.16 | 1158.02 |
| GYEEWLLNEIR | 10167 | 60.05 | 2 | 1420.7033 | 2 |  | 0 |  | no | 519.34 | 91.45 | 500.56 | 112.37 |
| LASDLLEWIR | 4493 | 89.90 | 3 | 1214.6656 | 2 |  | 0 |  | yes | 2638.85 | 690.74 | 2460.27 | 1339.10 |
| LLETIDQLYLEYAK | 16339 | 39.58 | 3 | 1710.9088 | 2 |  | 0 |  | no | 566.28 | 53.90 | 382.86 | 66.36 |
| LLETIDQLYLEYAK | 30722 | --- | --- | 1710.9062 | 3 |  | 0 |  | no | 73.99 | 28.03 | 93.36 | 8.36 |
| LVSIGAEEIVDGNVK | 7574 | 91.86 | 3 | 1541.8296 | 2 |  | 0 |  | no | 863.37 | 54.78 | 646.04 | 142.51 |
| TINEVENQILTR | 7062 | 59.51 | 1 | 1428.7528 | 2 |  | 0 |  | no | 971.81 | 535.03 | 849.17 | 1034.44 |
| VGWEQLLTTIAR | 6708 | 68.46 | 3 | 1385.7658 | 2 |  | 0 |  | yes | 1996.82 | 380.56 | 1789.99 | 913.79 |

  

| Tags | |
| --- | --- |

  

##

## Q9DBG6

RPN2\_MOUSE
Dolichyl-diphosphooligosaccharide--protein glycosyltransferase subunit 2 OS=Mus
musculus GN=Rpn2 PE=2 SV=1  
9 peptides

  

| Sequence | Peptide Ion | Score | Hits | Mass | Charge | Tags | Conflicts | Modifications | In quantitation | Average Normalised Abundances | | | |
| --- | --- | --- | --- | --- | --- | --- | --- | --- | --- | --- | --- | --- | --- |
|  |  |  |  |  |  |  |  |  |  | 2m2f | 4m2f | 2m4f | 4m4f |
| LQVSNVLSQPLAQAAVK | 8142 | 32.98 | 1 | 1765.0080 | 2 |  | 0 |  | no | 791.97 | 573.82 | 787.11 | 666.64 |
| LQVSNVLSQPLAQAAVK | 6313 | 26.95 | 3 | 1764.9997 | 3 |  | 0 |  | no | 473.37 | 508.34 | 561.90 | 464.05 |
| LSSGYYDFSVR | 3307 | 63.05 | 3 | 1292.6037 | 2 |  | 0 |  | no | 1901.71 | 1520.47 | 1752.74 | 1569.18 |
| NFESLSEAFSVASAAAALSQNR | 8405 | 44.46 | 3 | 2269.0950 | 3 |  | 0 |  | no | 3089.10 | 554.34 | 1626.26 | 604.09 |
| NIVEEIEDLVAR | 4650 | 64.43 | 3 | 1398.7348 | 2 |  | 0 |  | yes | 5711.75 | 1526.58 | 3747.94 | 1501.72 |
| NIVEEIEDLVAR | 15052 | --- | --- | 1398.7340 | 3 |  | 0 |  | yes | 342.91 | 154.72 | 228.82 | 105.19 |
| NPILWNVADVVIK | 4035 | 52.28 | 1 | 1479.8448 | 2 |  | 0 |  | yes | 4516.62 | 2203.26 | 4315.00 | 2373.16 |
| TGQEVVFVAEPDNK | 3216 | 67.67 | 3 | 1531.7494 | 2 |  | 0 |  | yes | 3305.68 | 4114.68 | 3048.34 | 6776.22 |
| TPFSLVGNVFELNFK | 13072 | 53.78 | 3 | 1710.8977 | 2 |  | 0 |  | no | 1051.29 | 223.59 | 1005.07 | 410.99 |
| YHVPVVVVPEGSTSDTQEQAILR | 5755 | 77.10 | 3 | 2523.2971 | 3 |  | 0 |  | no | 1826.16 | 727.67 | 1452.89 | 1443.02 |
| YIANTVELR | 3492 | 65.68 | 3 | 1077.5806 | 2 |  | 0 |  | no | 1272.11 | 1115.84 | 1085.03 | 1069.05 |

  

| Tags | |
| --- | --- |

  

##

## P62806

H4\_MOUSE
Histone H4 OS=Mus musculus GN=Hist1h4a PE=1 SV=2  
7 peptides

  

| Sequence | Peptide Ion | Score | Hits | Mass | Charge | Tags | Conflicts | Modifications | In quantitation | Average Normalised Abundances | | | |
| --- | --- | --- | --- | --- | --- | --- | --- | --- | --- | --- | --- | --- | --- |
|  |  |  |  |  |  |  |  |  |  | 2m2f | 4m2f | 2m4f | 4m4f |
| DAVTYTEHAK | 869 | 56.76 | 3 | 1133.5348 | 2 |  | 0 |  | no | 7059.46 | 6139.18 | 6948.25 | 5978.90 |
| DAVTYTEHAK | 697 | 27.51 | 3 | 1133.5355 | 3 |  | 0 |  | no | 4572.83 | 4089.36 | 5047.16 | 4151.34 |
| DNIQGITKPAIR | 724 | 41.51 | 3 | 1324.7459 | 3 |  | 0 |  | no | 5627.78 | 6142.07 | 5984.44 | 7630.72 |
| DNIQGITKPAIR | 2620 | 46.98 | 3 | 1324.7458 | 2 |  | 0 |  | no | 2732.59 | 1818.02 | 2037.23 | 1830.47 |
| ISGLIYEETR | 289 | 67.62 | 3 | 1179.6127 | 2 |  | 0 |  | yes | 3.24e+004 | 3.24e+004 | 3.01e+004 | 3.55e+004 |
| KTVTAMDVVYALK | 4361 | 40.43 | 3 | 1437.7863 | 3 |  | 0 |  | no | 653.64 | 347.16 | 646.07 | 460.74 |
| TVTAMDVVYALK | 2722 | 54.57 | 3 | 1309.6946 | 3 |  | 0 |  | yes | 2561.52 | 1447.31 | 2441.06 | 1754.98 |
| TVTAMDVVYALK | 317 | 75.78 | 3 | 1309.6949 | 2 |  | 0 |  | yes | 5.59e+004 | 3.83e+004 | 5.10e+004 | 4.16e+004 |
| TVTAMDVVYALK | 7046 | 69.88 | 3 | 1325.6867 | 2 |  | 0 | [5] Oxidation (M) | no | 1217.37 | 522.06 | 835.54 | 910.15 |
| VFLENVIR | 202 | 62.26 | 3 | 988.5701 | 2 |  | 0 |  | yes | 1.89e+004 | 2.19e+004 | 2.73e+004 | 2.80e+004 |

  

| Tags | |
| --- | --- |

  

##

## P45376

ALDR\_MOUSE
Aldose reductase OS=Mus musculus GN=Akr1b1 PE=1 SV=3  
8
peptides

  

| Sequence | Peptide Ion | Score | Hits | Mass | Charge | Tags | Conflicts | Modifications | In quantitation | Average Normalised Abundances | | | |
| --- | --- | --- | --- | --- | --- | --- | --- | --- | --- | --- | --- | --- | --- |
|  |  |  |  |  |  |  |  |  |  | 2m2f | 4m2f | 2m4f | 4m4f |
| EVGVALQEK | 1303 | 26.57 | 2 | 971.5279 | 2 |  | 0 |  | no | 3724.83 | 3809.22 | 3331.46 | 3699.26 |
| HIDCAQVYQNEK | 2832 | 48.30 | 3 | 1503.6797 | 3 |  | 0 |  | no | 1939.53 | 1510.03 | 1665.39 | 2026.87 |
| HIDCAQVYQNEK | 8140 | 41.30 | 3 | 1503.6921 | 2 |  | 0 |  | no | 1094.55 | 1983.74 | 1411.51 | 3578.80 |
| MPTLGLGTWK | 1131 | 53.86 | 3 | 1102.5817 | 2 |  | 0 |  | yes | 8008.58 | 1.01e+004 | 7252.49 | 7636.70 |
| RQDLFIVSK | 3687 | 58.03 | 3 | 1104.6285 | 2 |  | 0 |  | no | 1144.35 | 1054.66 | 880.32 | 1276.71 |
| SPPGQVTEAVK | 1586 | 59.05 | 3 | 1111.5864 | 2 |  | 0 |  | yes | 1.00e+004 | 9373.06 | 8111.70 | 9808.55 |
| TIGVSNFNPLQIER | 990 | 92.00 | 3 | 1586.8405 | 2 |  | 0 |  | yes | 1.55e+004 | 1.33e+004 | 1.41e+004 | 1.56e+004 |
| TIGVSNFNPLQIER | 6466 | 42.07 | 3 | 1586.8412 | 3 |  | 0 |  | yes | 507.64 | 465.26 | 496.84 | 604.28 |
| TTAQVLIR | 891 | 67.48 | 3 | 900.5386 | 2 |  | 0 |  | no | 3734.92 | 3732.59 | 3600.41 | 3875.12 |
| VAIDLGYR | 1009 | 45.26 | 3 | 905.4971 | 2 |  | 0 |  | no | 4106.99 | 4731.28 | 3913.04 | 4865.95 |

  

| Tags | |
| --- | --- |

  

##

## P15626

GSTM2\_MOUSE
Glutathione S-transferase Mu 2 OS=Mus musculus GN=Gstm2 PE=1 SV=2  
10
peptides

  

| Sequence | Peptide Ion | Score | Hits | Mass | Charge | Tags | Conflicts | Modifications | In quantitation | Average Normalised Abundances | | | |
| --- | --- | --- | --- | --- | --- | --- | --- | --- | --- | --- | --- | --- | --- |
|  |  |  |  |  |  |  |  |  |  | 2m2f | 4m2f | 2m4f | 4m4f |
| HNLCGETEEER | 10137 | --- | --- | 1372.5671 | 2 |  | 0 |  | no | 772.24 | 216.89 | 443.21 | 537.31 |
| HNLCGETEEER | 11601 | 27.87 | 1 | 1372.5674 | 3 |  | 0 |  | no | 700.89 | 520.91 | 610.25 | 817.88 |
| IQLAMVCYSPDFEK | 4305 | 73.29 | 3 | 1699.7936 | 2 |  | 0 |  | yes | 5502.31 | 4611.06 | 3989.76 | 3925.52 |
| ITQSNAILR | 1147 | 73.35 | 3 | 1014.5813 | 2 |  | 1 |  | no | 4469.47 | 4836.33 | 3815.89 | 4891.82 |
| KHNLCGETEEER | 15857 | --- | --- | 1500.6623 | 4 |  | 0 |  | no | 137.59 | 33.63 | 94.49 | 97.55 |
| KHNLCGETEEER | 4277 | 52.23 | 3 | 1500.6620 | 3 |  | 0 |  | no | 2342.99 | 1161.54 | 1647.90 | 1757.53 |
| KKPEYLEGLPEK | 3823 | 37.55 | 3 | 1429.7806 | 3 |  | 0 |  | no | 1318.85 | 2426.11 | 2265.29 | 4483.55 |
| LGLDFPNLPYLIDGSHK | 3075 | 41.97 | 3 | 1897.9926 | 3 |  | 0 |  | no | 6440.84 | 2045.84 | 5082.62 | 3329.76 |
| LLLEYTDTSYEDKK | 2586 | 41.41 | 3 | 1716.8441 | 3 |  | 0 |  | no | 3989.32 | 3333.44 | 2889.07 | 3227.08 |
| LYSEFLGK | 1227 | 27.01 | 3 | 955.5009 | 2 |  | 0 |  | no | 2902.20 | 2531.12 | 2478.72 | 2995.00 |
| PMTLGYWDIR | 1976 | 55.47 | 3 | 1250.6128 | 2 |  | 0 |  | yes | 6350.41 | 4242.51 | 3250.92 | 4166.28 |
| VDILENQAMDTR | 1932 | 79.61 | 2 | 1403.6735 | 2 |  | 0 |  | yes | 5776.33 | 6363.55 | 4349.25 | 7882.03 |

  

| Tags | |
| --- | --- |

  

##

## P10126

EF1A1\_MOUSE
Elongation factor 1-alpha 1 OS=Mus musculus GN=Eef1a1 PE=1 SV=3  
8
peptides

  

| Sequence | Peptide Ion | Score | Hits | Mass | Charge | Tags | Conflicts | Modifications | In quantitation | Average Normalised Abundances | | | |
| --- | --- | --- | --- | --- | --- | --- | --- | --- | --- | --- | --- | --- | --- |
|  |  |  |  |  |  |  |  |  |  | 2m2f | 4m2f | 2m4f | 4m4f |
| EHALLAYTLGVK | 3924 | 37.26 | 3 | 1313.7361 | 3 |  | 0 |  | no | 885.52 | 568.21 | 844.63 | 568.56 |
| EHALLAYTLGVK | 3719 | 51.62 | 3 | 1313.7277 | 2 |  | 0 |  | no | 1380.94 | 1059.39 | 1268.90 | 1028.66 |
| EVSTYIK | 1403 | 26.59 | 3 | 838.4434 | 2 |  | 0 |  | no | 2041.60 | 1190.01 | 1518.19 | 1348.65 |
| IGGIGTVPVGR | 777 | 75.47 | 3 | 1024.6019 | 2 |  | 0 |  | yes | 7403.47 | 5971.27 | 6957.01 | 7242.49 |
| LPLQDVYK | 604 | 58.03 | 3 | 974.5418 | 2 |  | 0 |  | yes | 1.19e+004 | 1.17e+004 | 1.25e+004 | 1.30e+004 |
| QTVAVGVIK | 3933 | 37.79 | 3 | 913.5594 | 2 |  | 0 |  | no | 823.71 | 672.68 | 960.07 | 797.22 |
| STTTGHLIYK | 1497 | 25.61 | 3 | 1119.5920 | 3 |  | 0 |  | no | 2303.53 | 1337.63 | 2118.66 | 1986.34 |
| THINIVVIGHVDSGK | 5663 | 71.93 | 3 | 1587.8729 | 3 |  | 0 |  | no | 1058.12 | 318.75 | 732.02 | 463.20 |
| THINIVVIGHVDSGK | 15326 | --- | --- | 1587.8722 | 4 |  | 0 |  | no | 120.76 | 29.14 | 82.46 | 75.15 |
| YYVTIIDAPGHR | 1298 | 57.45 | 3 | 1403.7191 | 3 |  | 0 |  | no | 4198.27 | 2957.95 | 3449.93 | 4367.99 |
| YYVTIIDAPGHR | 2772 | 58.04 | 3 | 1403.7085 | 2 |  | 0 |  | yes | 5675.65 | 5709.43 | 4687.58 | 6251.50 |

  

| Tags | |
| --- | --- |

  

##

## P56480

ATPB\_MOUSE
ATP synthase subunit beta, mitochondrial OS=Mus musculus GN=Atp5b PE=1
SV=2  
9 peptides

  

| Sequence | Peptide Ion | Score | Hits | Mass | Charge | Tags | Conflicts | Modifications | In quantitation | Average Normalised Abundances | | | |
| --- | --- | --- | --- | --- | --- | --- | --- | --- | --- | --- | --- | --- | --- |
|  |  |  |  |  |  |  |  |  |  | 2m2f | 4m2f | 2m4f | 4m4f |
| AIAELGIYPAVDPLDSTSR | 4734 | 26.61 | 3 | 1987.0287 | 3 |  | 0 |  | no | 2551.97 | 2411.22 | 2415.33 | 1646.01 |
| EGNDLYHEMIESGVINLK | 6182 | 46.00 | 3 | 2059.9888 | 3 |  | 0 |  | no | 2001.74 | 703.17 | 1333.02 | 1002.34 |
| FTQAGSEVSALLGR | 2827 | 98.61 | 3 | 1434.7441 | 2 |  | 0 |  | yes | 3003.32 | 2447.71 | 2865.98 | 2897.31 |
| FTQAGSEVSALLGR | 7413 | --- | --- | 1434.7313 | 3 |  | 0 |  | yes | 332.10 | 227.30 | 374.21 | 366.41 |
| IMDPNIVGNEHYDVAR | 4365 | 27.71 | 3 | 1841.8693 | 3 |  | 0 |  | no | 1932.29 | 1252.74 | 1410.92 | 1352.65 |
| LVLEVAQHLGESTVR | 6511 | 79.35 | 3 | 1649.9097 | 2 |  | 0 |  | no | 1388.23 | 541.87 | 929.08 | 391.62 |
| LVLEVAQHLGESTVR | 4350 | --- | --- | 1649.9094 | 3 |  | 0 |  | no | 1873.62 | 1179.61 | 1605.96 | 853.69 |
| MLSLVGR | 9080 | 28.86 | 3 | 774.4382 | 2 |  | 0 |  | no | 182.67 | 46.37 | 173.70 | 136.71 |
| TIAMDGTEGLVR | 3797 | 38.85 | 3 | 1261.6354 | 2 |  | 0 |  | no | 1242.87 | 1068.58 | 922.97 | 1049.03 |
| TVLIMELINNVAK | 4053 | 67.34 | 3 | 1456.8321 | 2 |  | 0 |  | yes | 6327.96 | 2772.10 | 6130.22 | 3395.34 |
| TVLIMELINNVAK | 13760 | --- | --- | 1456.8316 | 3 |  | 0 |  | yes | 296.80 | 130.95 | 339.70 | 114.92 |
| VALTGLTVAEYFR | 2585 | 86.34 | 3 | 1438.7817 | 2 |  | 0 |  | yes | 4425.85 | 2569.59 | 5277.66 | 3003.01 |

  

| Tags | |
| --- | --- |

  

##

## P14152

MDHC\_MOUSE
Malate dehydrogenase, cytoplasmic OS=Mus musculus GN=Mdh1 PE=1 SV=3  
7
peptides

  

| Sequence | Peptide Ion | Score | Hits | Mass | Charge | Tags | Conflicts | Modifications | In quantitation | Average Normalised Abundances | | | |
| --- | --- | --- | --- | --- | --- | --- | --- | --- | --- | --- | --- | --- | --- |
|  |  |  |  |  |  |  |  |  |  | 2m2f | 4m2f | 2m4f | 4m4f |
| DLDVAVLVGSMPR | 14854 | 36.20 | 1 | 1370.7230 | 3 |  | 0 |  | no | 127.22 | 103.81 | 102.42 | 26.93 |
| DLDVAVLVGSMPR | 4519 | 79.45 | 3 | 1370.7220 | 2 |  | 0 |  | no | 1748.22 | 1409.10 | 1708.89 | 618.78 |
| ELTEEKETAFEFLSSA | 8436 | 28.21 | 1 | 1829.8596 | 2 |  | 0 |  | no | 1518.29 | 1282.13 | 1630.92 | 1446.07 |
| ENFSCLTR | 2351 | 48.79 | 3 | 1025.4592 | 2 |  | 0 |  | no | 1798.90 | 1980.84 | 1605.10 | 1788.21 |
| EVGVYEALKDDSWLK | 11412 | 56.52 | 3 | 1750.8774 | 2 |  | 0 |  | no | 715.93 | 294.84 | 478.87 | 341.26 |
| EVGVYEALKDDSWLK | 3296 | 26.97 | 3 | 1750.8770 | 3 |  | 0 |  | no | 2676.37 | 1763.04 | 2193.81 | 1437.97 |
| FVEGLPINDFSR | 1024 | 45.57 | 3 | 1392.7028 | 2 |  | 0 |  | yes | 1.16e+004 | 1.37e+004 | 1.18e+004 | 1.04e+004 |
| GEFITTVQQR | 1905 | 70.86 | 3 | 1177.6097 | 2 |  | 0 |  | yes | 3281.59 | 3321.64 | 2870.71 | 3341.94 |
| VIVVGNPANTNCLTASK | 2046 | 102.58 | 3 | 1756.9140 | 2 |  | 0 |  | yes | 8480.25 | 8653.19 | 7073.31 | 7311.72 |

  

| Tags | |
| --- | --- |

  

##

## P62908

RS3\_MOUSE
40S ribosomal protein S3 OS=Mus musculus GN=Rps3 PE=1 SV=1  
9
peptides

  

| Sequence | Peptide Ion | Score | Hits | Mass | Charge | Tags | Conflicts | Modifications | In quantitation | Average Normalised Abundances | | | |
| --- | --- | --- | --- | --- | --- | --- | --- | --- | --- | --- | --- | --- | --- |
|  |  |  |  |  |  |  |  |  |  | 2m2f | 4m2f | 2m4f | 4m4f |
| AELNEFLTR | 3306 | 56.25 | 3 | 1091.5604 | 2 |  | 0 |  | no | 1821.86 | 1991.29 | 1666.76 | 1964.20 |
| DEILPTTPISEQK | 2849 | 47.12 | 3 | 1469.7603 | 2 |  | 0 |  | yes | 3750.07 | 3959.25 | 3451.95 | 4040.96 |
| ELAEDGYSGVEVR | 2087 | 90.23 | 3 | 1422.6679 | 2 |  | 0 |  | yes | 4578.22 | 4809.49 | 4554.70 | 4323.05 |
| FGFPEGSVELYAEK | 3897 | 64.06 | 3 | 1571.7505 | 2 |  | 0 |  | yes | 4009.54 | 2563.86 | 4086.17 | 2835.03 |
| FVADGIFK | 3312 | 35.25 | 3 | 895.4798 | 2 |  | 0 |  | no | 925.60 | 1087.54 | 987.46 | 1021.16 |
| GCEVVVSGK | 5953 | 48.60 | 3 | 933.4586 | 2 |  | 0 |  | no | 968.59 | 495.52 | 714.23 | 516.31 |
| GLCAIAQAESLR | 4155 | 60.57 | 3 | 1287.6586 | 2 |  | 0 |  | no | 2406.59 | 3184.92 | 1953.78 | 5013.35 |
| IMLPWDPSGK | 6510 | 30.79 | 3 | 1142.5784 | 2 |  | 0 |  | no | 848.15 | 493.70 | 801.66 | 672.52 |
| TEIIILATR | 3558 | 60.30 | 3 | 1028.6214 | 2 |  | 1 |  | no | 989.21 | 967.63 | 1111.79 | 1014.18 |

  

| Tags | |
| --- | --- |

  

##

## P08249

MDHM\_MOUSE
Malate dehydrogenase, mitochondrial OS=Mus musculus GN=Mdh2 PE=1
SV=3  
10 peptides

  

| Sequence | Peptide Ion | Score | Hits | Mass | Charge | Tags | Conflicts | Modifications | In quantitation | Average Normalised Abundances | | | |
| --- | --- | --- | --- | --- | --- | --- | --- | --- | --- | --- | --- | --- | --- |
|  |  |  |  |  |  |  |  |  |  | 2m2f | 4m2f | 2m4f | 4m4f |
| ANTFVAELK | 4351 | 63.30 | 3 | 991.5334 | 2 |  | 0 |  | no | 740.74 | 755.01 | 575.75 | 703.36 |
| EGVVECSFVQSK | 2944 | 51.28 | 1 | 1367.6381 | 2 |  | 0 |  | yes | 4060.48 | 7427.39 | 3596.46 | 6629.69 |
| ETECTYFSTPLLLGK | 4944 | 59.10 | 3 | 1757.8541 | 2 |  | 0 |  | yes | 4024.22 | 3455.62 | 3437.01 | 2880.31 |
| FVFSLVDAMNGK | 9301 | 33.08 | 2 | 1326.6632 | 2 |  | 0 |  | no | 614.26 | 311.69 | 538.71 | 331.40 |
| GCDVVVIPAGVPR | 3981 | 58.92 | 3 | 1337.7112 | 2 |  | 0 |  | no | 1618.19 | 1424.90 | 1458.70 | 1233.16 |
| GYLGPEQLPDCLK | 4966 | 41.03 | 3 | 1488.7280 | 2 |  | 0 |  | no | 1764.13 | 1506.17 | 1552.09 | 1213.24 |
| IFGVTTLDIVR | 2407 | 35.67 | 3 | 1232.7108 | 2 |  | 0 |  | no | 3728.31 | 3082.29 | 3332.96 | 3111.73 |
| IQEAGTEVVK | 3810 | 27.63 | 3 | 1072.5756 | 2 |  | 0 |  | no | 1247.90 | 1137.29 | 950.97 | 1216.36 |
| TIIPLISQCTPK | 7276 | 31.70 | 3 | 1369.7635 | 2 |  | 0 |  | no | 692.28 | 661.72 | 693.49 | 650.80 |
| VAVLGASGGIGQPLSLLLK | 7484 | 64.18 | 3 | 1792.0820 | 2 |  | 0 |  | yes | 6147.22 | 1874.07 | 4548.79 | 1897.16 |
| VAVLGASGGIGQPLSLLLK | 16259 | 26.49 | 3 | 1792.0814 | 3 |  | 0 |  | yes | 671.26 | 119.35 | 530.42 | 143.47 |

  

| Tags | |
| --- | --- |

  

##

## P27773

PDIA3\_MOUSE
Protein disulfide-isomerase A3 OS=Mus musculus GN=Pdia3 PE=1 SV=2  
10
peptides

  

| Sequence | Peptide Ion | Score | Hits | Mass | Charge | Tags | Conflicts | Modifications | In quantitation | Average Normalised Abundances | | | |
| --- | --- | --- | --- | --- | --- | --- | --- | --- | --- | --- | --- | --- | --- |
|  |  |  |  |  |  |  |  |  |  | 2m2f | 4m2f | 2m4f | 4m4f |
| DASVVGFFR | 3077 | 59.13 | 3 | 996.5029 | 2 |  | 0 |  | no | 1395.73 | 1531.10 | 1436.05 | 1485.94 |
| DLLTAYYDVDYEK | 3315 | 68.10 | 3 | 1606.7539 | 2 |  | 0 |  | yes | 6816.85 | 5609.57 | 7080.60 | 3604.19 |
| ELNDFISYLQR | 2033 | 64.92 | 3 | 1396.6985 | 2 |  | 0 |  | yes | 6694.89 | 4290.45 | 5741.82 | 5255.05 |
| FVMQEEFSR | 3297 | 45.53 | 3 | 1171.5311 | 2 |  | 0 |  | no | 2020.62 | 1982.76 | 2100.33 | 2048.76 |
| GFPTIYFSPANK | 3804 | 43.44 | 3 | 1340.6774 | 2 |  | 0 |  | no | 3345.02 | 2674.81 | 3234.06 | 3450.87 |
| LAPEYEAAATR | 1820 | 50.97 | 3 | 1190.5886 | 2 |  | 0 |  | no | 4749.90 | 3535.68 | 3949.50 | 3971.10 |
| LNFAVASR | 4483 | 39.00 | 1 | 876.4812 | 2 |  | 0 |  | no | 1128.59 | 896.83 | 1230.63 | 566.28 |
| MDATANDVPSPYEVK | 4411 | 29.08 | 2 | 1635.7319 | 2 |  | 0 |  | no | 4462.95 | 4713.51 | 4339.50 | 4461.86 |
| VDCTANTNTCNK | 5363 | 51.16 | 3 | 1396.5705 | 2 |  | 0 |  | yes | 6225.36 | 3600.93 | 4236.44 | 3856.36 |
| YGVSGYPTLK | 1962 | 40.05 | 3 | 1083.5529 | 2 |  | 0 |  | no | 2659.72 | 2599.11 | 2637.85 | 2858.14 |

  

| Tags | |
| --- | --- |

  

##

## Q03265

ATPA\_MOUSE
ATP synthase subunit alpha, mitochondrial OS=Mus musculus GN=Atp5a1 PE=1
SV=1  
7 peptides

  

| Sequence | Peptide Ion | Score | Hits | Mass | Charge | Tags | Conflicts | Modifications | In quantitation | Average Normalised Abundances | | | |
| --- | --- | --- | --- | --- | --- | --- | --- | --- | --- | --- | --- | --- | --- |
|  |  |  |  |  |  |  |  |  |  | 2m2f | 4m2f | 2m4f | 4m4f |
| AVDSLVPIGR | 3334 | 69.02 | 3 | 1025.5861 | 2 |  | 0 |  | no | 1094.35 | 735.25 | 1021.54 | 1027.90 |
| EIVTNFLAGFEP | 5928 | 43.50 | 3 | 1335.6705 | 2 |  | 0 |  | yes | 5391.31 | 2212.85 | 4848.65 | 2008.84 |
| EVAAFAQFGSDLDAATQQLLSR | 8864 | 27.16 | 3 | 2337.1599 | 3 |  | 0 |  | no | 2749.77 | 780.67 | 1744.68 | 829.36 |
| ILGADTSVDLEETGR | 2834 | 125.44 | 3 | 1574.7785 | 2 |  | 0 |  | yes | 4472.73 | 4119.35 | 4309.46 | 2753.15 |
| NVQAEEMVEFSSGLK | 6024 | 86.76 | 1 | 1666.7857 | 2 |  | 0 |  | no | 2262.41 | 2558.40 | 1970.64 | 1814.03 |
| TGAIVDVPVGEELLGR | 2041 | 65.00 | 3 | 1623.8833 | 2 |  | 0 |  | yes | 7700.42 | 7007.76 | 8664.68 | 7232.67 |
| TSIAIDTIINQK | 2561 | 70.45 | 3 | 1315.7341 | 2 |  | 0 |  | no | 2629.25 | 2238.67 | 2245.30 | 1575.39 |

  

| Tags | |
| --- | --- |

  

##

## Q04447

KCRB\_MOUSE
Creatine kinase B-type OS=Mus musculus GN=Ckb PE=1 SV=1  
6
peptides

  

| Sequence | Peptide Ion | Score | Hits | Mass | Charge | Tags | Conflicts | Modifications | In quantitation | Average Normalised Abundances | | | |
| --- | --- | --- | --- | --- | --- | --- | --- | --- | --- | --- | --- | --- | --- |
|  |  |  |  |  |  |  |  |  |  | 2m2f | 4m2f | 2m4f | 4m4f |
| DLFDPIIEER | 1334 | 57.23 | 3 | 1245.6235 | 2 |  | 0 |  | yes | 6494.65 | 9259.52 | 8225.06 | 6946.47 |
| FCTGLTQIETLFK | 4061 | 89.03 | 3 | 1556.7911 | 2 |  | 0 |  | yes | 4337.20 | 1989.71 | 4053.13 | 2705.49 |
| GTGGVDTAAVGGVFDVSNADR | 4564 | 38.67 | 3 | 1963.9221 | 3 |  | 0 |  | no | 1429.54 | 1253.56 | 1310.45 | 973.72 |
| LAVEALSSLDGDLSGR | 6298 | 39.28 | 1 | 1601.8246 | 3 |  | 0 |  | yes | 649.46 | 371.17 | 674.75 | 176.00 |
| LAVEALSSLDGDLSGR | 2133 | 121.23 | 3 | 1601.8261 | 2 |  | 0 |  | yes | 5737.66 | 3350.93 | 5536.92 | 2006.22 |
| LGFSEVELVQMVVDGVK | 10577 | 52.90 | 3 | 1847.9710 | 2 |  | 0 |  | no | 2027.44 | 665.77 | 1622.59 | 435.19 |
| LGFSEVELVQMVVDGVK | 11075 | 38.66 | 3 | 1847.9694 | 3 |  | 0 |  | no | 1010.04 | 336.77 | 773.51 | 222.22 |
| TFLVWINEEDHLR | 8561 | 35.10 | 3 | 1670.8397 | 3 |  | 0 |  | no | 710.27 | 311.23 | 561.22 | 237.79 |

  

| Tags | |
| --- | --- |

  

##

## P97315

CSRP1\_MOUSE
Cysteine and glycine-rich protein 1 OS=Mus musculus GN=Csrp1 PE=1
SV=3  
8 peptides

  

| Sequence | Peptide Ion | Score | Hits | Mass | Charge | Tags | Conflicts | Modifications | In quantitation | Average Normalised Abundances | | | |
| --- | --- | --- | --- | --- | --- | --- | --- | --- | --- | --- | --- | --- | --- |
|  |  |  |  |  |  |  |  |  |  | 2m2f | 4m2f | 2m4f | 4m4f |
| CSQAVYAAEK | 6439 | 53.55 | 3 | 1125.5124 | 2 |  | 0 |  | no | 745.67 | 813.68 | 763.62 | 504.39 |
| GFGFGQGAGALVHSE | 979 | 67.57 | 3 | 1432.6738 | 2 |  | 0 |  | no | 7940.80 | 1.11e+004 | 1.14e+004 | 9139.12 |
| GLESTTLADKDGEIYCK | 819 | 55.54 | 3 | 1898.8854 | 3 |  | 0 |  | yes | 1.19e+004 | 1.13e+004 | 1.12e+004 | 1.43e+004 |
| GYGYGQGAGTLSTDK | 1751 | 77.94 | 3 | 1473.6732 | 2 |  | 0 |  | no | 5607.04 | 7068.37 | 6283.76 | 7266.90 |
| GYGYGQGAGTLSTDKGESLGIK | 1433 | 38.43 | 3 | 2158.0404 | 3 |  | 0 |  | yes | 1.45e+004 | 1.35e+004 | 1.41e+004 | 1.46e+004 |
| HEEAPGHRPTTNPNASK | 19003 | --- | --- | 1841.8738 | 3 |  | 0 |  | no | 917.88 | 97.94 | 414.71 | 34.22 |
| HEEAPGHRPTTNPNASK | 6171 | 28.80 | 2 | 1841.8763 | 4 |  | 0 |  | no | 4518.32 | 1252.45 | 3017.54 | 765.86 |
| NLDSTTVAVHGEEIYCK | 811 | 41.06 | 3 | 1934.9032 | 3 |  | 0 |  | yes | 1.45e+004 | 1.53e+004 | 1.79e+004 | 1.15e+004 |
| NLDSTTVAVHGEEIYCK | 6240 | 75.01 | 3 | 1934.9063 | 2 |  | 0 |  | yes | 1506.76 | 1063.27 | 1749.33 | 865.31 |
| TVYFAEEVQCEGNSFHK | 1600 | 28.71 | 3 | 2043.8979 | 3 |  | 0 |  | no | 8461.22 | 3871.73 | 7283.16 | 5164.88 |

  

| Tags | |
| --- | --- |

  

##

## Q61879

MYH10\_MOUSE
Myosin-10 OS=Mus musculus GN=Myh10 PE=1 SV=2  
8 peptides

  

| Sequence | Peptide Ion | Score | Hits | Mass | Charge | Tags | Conflicts | Modifications | In quantitation | Average Normalised Abundances | | | |
| --- | --- | --- | --- | --- | --- | --- | --- | --- | --- | --- | --- | --- | --- |
|  |  |  |  |  |  |  |  |  |  | 2m2f | 4m2f | 2m4f | 4m4f |
| ALEEALEAK | 2123 | 48.46 | 3 | 972.5034 | 2 |  | 1 |  | no | 2591.10 | 1893.80 | 2436.67 | 2415.66 |
| ALELDPNLYR | 1691 | 45.85 | 3 | 1202.6272 | 2 |  | 1 |  | no | 3611.49 | 2436.77 | 3308.21 | 2950.42 |
| EDQSILCTGESGAGK | 2805 | 68.63 | 3 | 1550.6829 | 2 |  | 2 |  | no | 4611.77 | 2706.44 | 4055.32 | 4123.34 |
| FDQLLAEEK | 4779 | 58.34 | 3 | 1091.5412 | 2 |  | 2 |  | no | 836.23 | 356.40 | 579.35 | 531.05 |
| IVFQEFR | 1970 | 39.97 | 3 | 937.5011 | 2 |  | 1 |  | no | 1901.47 | 623.16 | 1827.83 | 1233.28 |
| KFDQLLAEEK | 9347 | 42.12 | 3 | 1219.6444 | 3 |  | 2 |  | no | 225.59 | 33.67 | 268.76 | 131.26 |
| TQLEELEDELQATEDAK | 3799 | 53.55 | 3 | 1960.9107 | 3 |  | 1 |  | no | 2134.98 | 650.25 | 2643.43 | 1006.97 |
| TQLEELEDELQATEDAK | 5214 | 68.03 | 3 | 1960.9116 | 2 |  | 1 |  | no | 2891.89 | 757.07 | 2819.56 | 995.69 |
| VKPLLQVTR | 5102 | 41.43 | 1 | 1052.6709 | 3 |  | 1 |  | no | 368.23 | 347.80 | 516.90 | 464.98 |
| VKPLLQVTR | 10591 | --- | --- | 1052.6704 | 2 |  | 1 |  | no | 213.57 | 28.00 | 175.75 | 106.15 |

  

| Tags | |
| --- | --- |

  

##

## Q08091

CNN1\_MOUSE
Calponin-1 OS=Mus musculus GN=Cnn1 PE=2 SV=1  
9 peptides

  

| Sequence | Peptide Ion | Score | Hits | Mass | Charge | Tags | Conflicts | Modifications | In quantitation | Average Normalised Abundances | | | |
| --- | --- | --- | --- | --- | --- | --- | --- | --- | --- | --- | --- | --- | --- |
|  |  |  |  |  |  |  |  |  |  | 2m2f | 4m2f | 2m4f | 4m4f |
| DGIILCEFINK | 917 | 46.19 | 3 | 1320.6713 | 2 |  | 0 |  | yes | 1.59e+004 | 1.25e+004 | 1.63e+004 | 1.08e+004 |
| EWIEGVTGR | 1039 | 50.87 | 3 | 1045.5188 | 2 |  | 0 |  | yes | 4416.54 | 4705.89 | 3886.77 | 4995.28 |
| GASQAGMTAPGTK | 5001 | 60.70 | 3 | 1175.5625 | 2 |  | 0 |  | no | 1055.68 | 699.21 | 997.56 | 875.77 |
| GMTVYGLPR | 3210 | 43.79 | 3 | 992.5105 | 2 |  | 0 |  | no | 877.77 | 750.34 | 912.16 | 850.83 |
| GPAYGLSAEVK | 694 | 54.00 | 3 | 1090.5679 | 2 |  | 0 |  | yes | 1.41e+004 | 1.50e+004 | 1.44e+004 | 1.51e+004 |
| IGNNFMDGLK | 1980 | 61.38 | 3 | 1107.5375 | 2 |  | 0 |  | no | 3070.10 | 3234.42 | 2935.92 | 3094.51 |
| LGTDQPLDQATISLQMGTNK | 5555 | 53.40 | 1 | 2130.0608 | 3 |  | 0 |  | no | 2527.40 | 2099.84 | 3668.98 | 917.31 |
| LGTDQPLDQATISLQMGTNK | 17167 | --- | --- | 2130.0638 | 2 |  | 0 |  | no | 446.81 | 236.84 | 506.11 | 151.57 |
| NIIGLQMGTNK | 2814 | 60.05 | 3 | 1187.6346 | 2 |  | 0 |  | no | 1654.86 | 1158.35 | 1391.41 | 1559.87 |
| VNESTQNWHQLENIGNFIK | 2788 | 35.48 | 3 | 2270.1070 | 3 |  | 0 |  | no | 5458.55 | 2144.52 | 4079.41 | 2609.83 |

  

| Tags | |
| --- | --- |

  

##

## P58252

EF2\_MOUSE
Elongation factor 2 OS=Mus musculus GN=Eef2 PE=1 SV=2  
6
peptides

  

| Sequence | Peptide Ion | Score | Hits | Mass | Charge | Tags | Conflicts | Modifications | In quantitation | Average Normalised Abundances | | | |
| --- | --- | --- | --- | --- | --- | --- | --- | --- | --- | --- | --- | --- | --- |
|  |  |  |  |  |  |  |  |  |  | 2m2f | 4m2f | 2m4f | 4m4f |
| AYLPVNESFGFTADLR | 5823 | 64.20 | 3 | 1798.8887 | 2 |  | 0 |  | yes | 3184.98 | 1093.78 | 1927.42 | 669.63 |
| AYLPVNESFGFTADLR | 10107 | 29.75 | 3 | 1798.8885 | 3 |  | 0 |  | yes | 638.57 | 268.10 | 410.54 | 186.12 |
| ETVSEESNVLCLSK | 2232 | 96.76 | 3 | 1593.7560 | 2 |  | 0 |  | yes | 5922.06 | 4238.26 | 4914.46 | 4061.61 |
| GEGQLSAAER | 3324 | 84.43 | 3 | 1016.4884 | 2 |  | 0 |  | no | 1333.11 | 1242.03 | 1159.91 | 948.40 |
| STLTDSLVCK | 2600 | 55.78 | 3 | 1122.5591 | 2 |  | 0 |  | no | 1976.37 | 1824.17 | 2081.35 | 1915.76 |
| VFSGVVSTGLK | 2037 | 74.19 | 3 | 1092.6185 | 2 |  | 0 |  | yes | 2513.35 | 2624.95 | 2401.58 | 2744.15 |
| YEWDVAEAR | 2860 | 57.51 | 3 | 1137.5093 | 2 |  | 0 |  | no | 2022.60 | 2015.95 | 1887.08 | 1629.63 |

  

| Tags | |
| --- | --- |

  

##

## Q6ZWY9

H2B1C\_MOUSE
Histone H2B type 1-C/E/G OS=Mus musculus GN=Hist1h2bc PE=1 SV=3  
7
peptides

  

| Sequence | Peptide Ion | Score | Hits | Mass | Charge | Tags | Conflicts | Modifications | In quantitation | Average Normalised Abundances | | | |
| --- | --- | --- | --- | --- | --- | --- | --- | --- | --- | --- | --- | --- | --- |
|  |  |  |  |  |  |  |  |  |  | 2m2f | 4m2f | 2m4f | 4m4f |
| AMGIMNSFVNDIFER | 1927 | 81.72 | 3 | 1742.8119 | 2 |  | 0 |  | yes | 2.47e+004 | 8461.19 | 1.70e+004 | 8179.26 |
| AMGIMNSFVNDIFER | 9370 | 39.60 | 3 | 1742.8113 | 3 |  | 0 |  | yes | 1269.88 | 297.56 | 827.20 | 288.63 |
| AMGIMNSFVNDIFER | 11347 | 54.44 | 3 | 1758.8070 | 2 |  | 0 | [2] Oxidation (M) | no | 2043.18 | 442.29 | 1115.79 | 253.02 |
| EIQTAVR | 485 | 28.25 | 3 | 815.4502 | 2 |  | 0 |  | yes | 1.10e+004 | 1.12e+004 | 1.19e+004 | 8913.76 |
| ESYSVYVYK | 1325 | 47.17 | 3 | 1136.5389 | 2 |  | 0 |  | no | 5517.95 | 4870.21 | 5002.25 | 5399.29 |
| KESYSVYVYK | 1505 | 71.99 | 3 | 1264.6332 | 2 |  | 0 |  | no | 5043.59 | 3697.40 | 4141.70 | 3965.26 |
| KESYSVYVYK | 1402 | 48.40 | 3 | 1264.6339 | 3 |  | 0 |  | no | 2917.55 | 2230.85 | 2552.64 | 2631.63 |
| LLLPGELAK | 198 | 50.57 | 3 | 952.5950 | 2 |  | 0 |  | yes | 2.92e+004 | 3.27e+004 | 2.75e+004 | 3.48e+004 |
| QVHPDTGISSK | 5011 | 31.33 | 3 | 1167.5912 | 2 |  | 0 |  | no | 2002.78 | 658.42 | 1110.85 | 910.31 |
| QVHPDTGISSK | 1359 | --- | --- | 1167.5885 | 3 |  | 0 |  | no | 5412.91 | 3016.01 | 4383.03 | 3193.90 |

  

| Tags | |
| --- | --- |

  

##

## Q922R8

PDIA6\_MOUSE
Protein disulfide-isomerase A6 OS=Mus musculus GN=Pdia6 PE=1 SV=3  
6
peptides

  

| Sequence | Peptide Ion | Score | Hits | Mass | Charge | Tags | Conflicts | Modifications | In quantitation | Average Normalised Abundances | | | |
| --- | --- | --- | --- | --- | --- | --- | --- | --- | --- | --- | --- | --- | --- |
|  |  |  |  |  |  |  |  |  |  | 2m2f | 4m2f | 2m4f | 4m4f |
| DVVELTDDTFDK | 6485 | 30.65 | 3 | 1395.6368 | 2 |  | 0 |  | no | 1440.46 | 1387.79 | 1344.14 | 1412.06 |
| GESPVDYDGGR | 6585 | 40.44 | 2 | 1150.4886 | 2 |  | 0 |  | no | 347.73 | 787.28 | 917.33 | 1141.57 |
| GSTAPVGGGSFPTITPR | 2940 | 70.26 | 3 | 1600.8222 | 2 |  | 0 |  | yes | 3585.34 | 2237.49 | 2754.68 | 2992.79 |
| LAAVDATVNQVLASR | 5782 | 64.38 | 3 | 1526.8454 | 3 |  | 0 |  | yes | 640.13 | 338.99 | 590.57 | 556.51 |
| LAAVDATVNQVLASR | 2954 | 102.06 | 3 | 1526.8403 | 2 |  | 0 |  | yes | 2573.72 | 2070.42 | 2902.99 | 2281.14 |
| NSYLEVLLK | 7973 | 41.44 | 3 | 1077.6064 | 2 |  | 0 |  | no | 465.61 | 242.56 | 368.35 | 233.38 |
| TGEAIVDAALSALR | 2667 | 100.83 | 3 | 1385.7510 | 2 |  | 0 |  | yes | 6869.73 | 3112.20 | 6230.26 | 4666.74 |
| TGEAIVDAALSALR | 5593 | --- | --- | 1385.7514 | 3 |  | 0 |  | yes | 1138.08 | 487.35 | 1102.86 | 699.01 |

  

| Tags | |
| --- | --- |

  

##

## P62962

PROF1\_MOUSE
Profilin-1 OS=Mus musculus GN=Pfn1 PE=1 SV=2  
6 peptides

  

| Sequence | Peptide Ion | Score | Hits | Mass | Charge | Tags | Conflicts | Modifications | In quantitation | Average Normalised Abundances | | | |
| --- | --- | --- | --- | --- | --- | --- | --- | --- | --- | --- | --- | --- | --- |
|  |  |  |  |  |  |  |  |  |  | 2m2f | 4m2f | 2m4f | 4m4f |
| DSLLQDGEFTMDLR | 4280 | 75.12 | 3 | 1638.7571 | 2 |  | 0 |  | yes | 5640.91 | 2556.30 | 3925.92 | 1427.82 |
| DSLLQDGEFTMDLR | 16522 | --- | --- | 1638.7557 | 3 |  | 0 |  | yes | 228.72 | 87.43 | 143.59 | 30.08 |
| DSPSVWAAVPGK | 2110 | 83.77 | 3 | 1212.6136 | 2 |  | 0 |  | no | 2474.32 | 2073.11 | 2297.49 | 2412.87 |
| SSFFVNGLTLGGQK | 2368 | 67.14 | 3 | 1453.7553 | 2 |  | 0 |  | yes | 4610.64 | 4273.34 | 4269.45 | 4540.34 |
| STGGAPTFNVTVTMTAK | 4416 | 65.53 | 3 | 1681.8347 | 2 |  | 0 |  | no | 3549.08 | 2715.18 | 2307.96 | 1566.19 |
| TFVSITPAEVGVLVGK | 8003 | 62.89 | 3 | 1615.9173 | 3 |  | 0 |  | yes | 1088.73 | 649.26 | 1034.10 | 532.21 |
| TFVSITPAEVGVLVGK | 1931 | 27.62 | 3 | 1615.9187 | 2 |  | 0 |  | yes | 1.79e+004 | 1.13e+004 | 1.67e+004 | 9719.05 |
| TLVLLMGK | 2591 | 51.23 | 3 | 873.5350 | 2 |  | 0 |  | no | 1012.03 | 1350.20 | 1037.32 | 1273.65 |

  

| Tags | |
| --- | --- |

  

##

## P51881

ADT2\_MOUSE
ADP/ATP translocase 2 OS=Mus musculus GN=Slc25a5 PE=1 SV=3  
8
peptides

  

| Sequence | Peptide Ion | Score | Hits | Mass | Charge | Tags | Conflicts | Modifications | In quantitation | Average Normalised Abundances | | | |
| --- | --- | --- | --- | --- | --- | --- | --- | --- | --- | --- | --- | --- | --- |
|  |  |  |  |  |  |  |  |  |  | 2m2f | 4m2f | 2m4f | 4m4f |
| AAYFGIYDTAK | 2099 | 46.41 | 3 | 1218.5952 | 2 |  | 0 |  | yes | 4795.50 | 6948.81 | 4828.41 | 6105.08 |
| DFLAGGVAAAISK | 1940 | 94.88 | 3 | 1218.6618 | 2 |  | 1 |  | no | 4291.56 | 3936.71 | 4956.37 | 3547.91 |
| EQGVLSFWR | 2931 | 44.33 | 3 | 1120.5655 | 2 |  | 0 |  | yes | 2019.43 | 1701.95 | 1892.68 | 1734.09 |
| GAWSNVLR | 2797 | 57.99 | 3 | 901.4764 | 2 |  | 1 |  | no | 895.51 | 796.12 | 853.63 | 919.35 |
| GTDIMYTGTLDCWR | 5273 | 46.54 | 3 | 1687.7342 | 2 |  | 0 |  | yes | 1934.30 | 1402.26 | 1728.89 | 1013.71 |
| GTDIMYTGTLDCWR | 8655 | 46.44 | 3 | 1703.7310 | 2 |  | 0 | [5] Oxidation (M) | no | 818.21 | 507.98 | 1000.27 | 428.16 |
| LLLQVQHASK | 3287 | --- | --- | 1135.6718 | 3 |  | 1 |  | no | 713.89 | 666.18 | 695.94 | 855.13 |
| LLLQVQHASK | 7966 | 37.72 | 3 | 1135.6711 | 2 |  | 1 |  | no | 433.23 | 378.22 | 328.28 | 688.06 |
| YFPTQALNFAFK | 4097 | 56.29 | 3 | 1445.7342 | 2 |  | 1 |  | no | 4087.87 | 1815.62 | 3868.63 | 3271.53 |

  

| Tags | |
| --- | --- |

  

##

## Q64356

SVS6\_MOUSE
Seminal vesicle secretory protein 6 OS=Mus musculus GN=Svs6 PE=2 SV=1  
5
peptides

  

| Sequence | Peptide Ion | Score | Hits | Mass | Charge | Tags | Conflicts | Modifications | In quantitation | Average Normalised Abundances | | | |
| --- | --- | --- | --- | --- | --- | --- | --- | --- | --- | --- | --- | --- | --- |
|  |  |  |  |  |  |  |  |  |  | 2m2f | 4m2f | 2m4f | 4m4f |
| ASAGEIER | 1952 | 51.99 | 3 | 831.4086 | 2 |  | 0 |  | no | 4066.41 | 4668.52 | 4640.80 | 4579.46 |
| FSQAIEEFSSESSEANSPK | 71 | 86.81 | 3 | 2072.9184 | 2 |  | 0 |  | yes | 4.22e+005 | 1.22e+006 | 6.57e+005 | 8.34e+005 |
| FSQAIEEFSSESSEANSPK | 10151 | --- | --- | 2072.9176 | 2 |  | 0 |  | yes | 6610.97 | 2.90e+004 | 1.47e+004 | 2.04e+004 |
| FSQAIEEFSSESSEANSPK | 9216 | 35.37 | 3 | 2072.9164 | 3 |  | 0 |  | yes | 5687.21 | 2.53e+004 | 1.32e+004 | 1.74e+004 |
| FSQAIEEFSSESSEANSPK | 53 | 89.97 | 3 | 2072.9175 | 3 |  | 0 |  | yes | 3.15e+005 | 8.87e+005 | 4.95e+005 | 6.21e+005 |
| NMVNGEDGEDSK | 705 | 42.66 | 3 | 1293.5136 | 2 |  | 0 |  | no | 2.59e+004 | 3.13e+004 | 2.45e+004 | 2.76e+004 |
| SIVHEEVYEEK | 51 | 53.10 | 3 | 1360.6495 | 2 |  | 0 |  | yes | 2.06e+005 | 2.82e+005 | 2.32e+005 | 2.74e+005 |
| SIVHEEVYEEK | 27 | 42.52 | 3 | 1360.6507 | 3 |  | 0 |  | yes | 1.82e+005 | 2.64e+005 | 2.18e+005 | 2.69e+005 |
| SIVHEEVYEEKK | 3993 | --- | --- | 1488.7458 | 4 |  | 0 |  | no | 466.32 | 761.94 | 600.71 | 1158.25 |
| SIVHEEVYEEKK | 2253 | 49.72 | 3 | 1488.7460 | 3 |  | 0 |  | no | 1843.15 | 2820.52 | 2212.99 | 3961.65 |

  

| Tags | |
| --- | --- |

  

##

## P68372

TBB2C\_MOUSE
Tubulin beta-2C chain OS=Mus musculus GN=Tubb2c PE=1 SV=1  
8
peptides

  

| Sequence | Peptide Ion | Score | Hits | Mass | Charge | Tags | Conflicts | Modifications | In quantitation | Average Normalised Abundances | | | |
| --- | --- | --- | --- | --- | --- | --- | --- | --- | --- | --- | --- | --- | --- |
|  |  |  |  |  |  |  |  |  |  | 2m2f | 4m2f | 2m4f | 4m4f |
| ALTVPELTQQMFDAK | 8402 | 25.04 | 3 | 1690.8600 | 2 |  | 1 |  | no | 2156.73 | 1131.60 | 2041.37 | 1496.81 |
| EVDEQMLNVQNK | 4065 | 61.26 | 3 | 1445.6826 | 2 |  | 1 |  | no | 2690.96 | 1834.69 | 2463.05 | 2691.48 |
| FPGQLNADLR | 4853 | 42.19 | 1 | 1129.5811 | 2 |  | 2 |  | no | 3425.65 | 960.87 | 1793.12 | 2408.56 |
| GHYTEGAELVDSVLDVVR | 5191 | 56.74 | 3 | 1957.9734 | 3 |  | 2 |  | no | 3212.66 | 859.58 | 2149.77 | 778.45 |
| IMNTFSVVPSPK | 7269 | 58.29 | 3 | 1318.6967 | 2 |  | 2 |  | no | 1171.12 | 647.80 | 1004.31 | 912.93 |
| INVYYNEATGGK | 2743 | 52.71 | 3 | 1327.6404 | 2 |  | 0 |  | yes | 2345.46 | 2356.98 | 2616.51 | 2214.15 |
| LAVNMVPFPR | 3890 | 64.21 | 3 | 1142.6264 | 2 |  | 2 |  | no | 1222.96 | 766.57 | 1129.00 | 883.32 |
| NSSYFVEWIPNNVK | 11452 | --- | --- | 1695.8237 | 3 |  | 2 |  | no | 297.98 | 133.79 | 238.65 | 233.30 |
| NSSYFVEWIPNNVK | 3507 | 46.86 | 3 | 1695.8253 | 2 |  | 2 |  | no | 4303.55 | 2767.68 | 3878.40 | 3339.32 |

  

| Tags | |
| --- | --- |

  

##

## P63101

1433Z\_MOUSE
14-3-3 protein zeta/delta OS=Mus musculus GN=Ywhaz PE=1 SV=1  
7
peptides

  

| Sequence | Peptide Ion | Score | Hits | Mass | Charge | Tags | Conflicts | Modifications | In quantitation | Average Normalised Abundances | | | |
| --- | --- | --- | --- | --- | --- | --- | --- | --- | --- | --- | --- | --- | --- |
|  |  |  |  |  |  |  |  |  |  | 2m2f | 4m2f | 2m4f | 4m4f |
| DICNDVLSLLEK | 47232 | --- | --- | 1417.7111 | 3 |  | 0 |  | yes | 65.71 | 14.82 | 50.13 | 29.33 |
| DICNDVLSLLEK | 4979 | 42.13 | 3 | 1417.7116 | 2 |  | 0 |  | yes | 3951.04 | 1341.48 | 3436.27 | 1719.44 |
| DSTLIMQLLR | 2121 | 76.05 | 3 | 1188.6524 | 2 |  | 3 |  | no | 5173.61 | 3874.97 | 5291.93 | 4679.16 |
| EMQPTHPIR | 6444 | 25.85 | 2 | 1107.5546 | 2 |  | 2 |  | no | 447.12 | 686.98 | 457.79 | 791.37 |
| EMQPTHPIR | 7547 | --- | --- | 1107.5558 | 3 |  | 2 |  | no | 89.40 | 271.70 | 151.15 | 342.12 |
| NLLSVAYK | 1420 | 43.60 | 3 | 906.5168 | 2 |  | 3 |  | no | 1821.69 | 1728.75 | 1828.48 | 2020.75 |
| SVTEQGAELSNEER | 4000 | 94.46 | 3 | 1547.7045 | 2 |  | 0 |  | yes | 2473.73 | 1389.20 | 2031.22 | 1513.15 |
| TAFDEAIAELDTLSEESYK | 3918 | --- | --- | 2130.9840 | 3 |  | 0 |  | yes | 8561.64 | 2609.49 | 7404.39 | 3138.58 |
| TAFDEAIAELDTLSEESYK | 10251 | 67.11 | 3 | 2130.9852 | 2 |  | 0 |  | yes | 3180.97 | 614.72 | 2444.78 | 1087.32 |
| YLAEVAAGDDKK | 10976 | 43.72 | 3 | 1278.6441 | 2 |  | 0 |  | no | 515.46 | 402.31 | 401.71 | 594.43 |

  

| Tags | |
| --- | --- |

  

##

## Q05186

RCN1\_MOUSE
Reticulocalbin-1 OS=Mus musculus GN=Rcn1 PE=1 SV=1  
5
peptides

  

| Sequence | Peptide Ion | Score | Hits | Mass | Charge | Tags | Conflicts | Modifications | In quantitation | Average Normalised Abundances | | | |
| --- | --- | --- | --- | --- | --- | --- | --- | --- | --- | --- | --- | --- | --- |
|  |  |  |  |  |  |  |  |  |  | 2m2f | 4m2f | 2m4f | 4m4f |
| ASDLDGDLTATR | 2095 | 64.64 | 3 | 1233.5828 | 2 |  | 0 |  | yes | 3385.12 | 3782.33 | 3210.95 | 4244.64 |
| EIVVLETLEDIDK | 2749 | 102.01 | 3 | 1514.8078 | 2 |  | 0 |  | yes | 6585.57 | 4132.69 | 7149.11 | 4633.99 |
| HWILPQDYDHAQAEAR | 9999 | 29.88 | 2 | 1948.9166 | 3 |  | 0 |  | no | 791.18 | 161.80 | 578.18 | 515.69 |
| IDSDGDGLVTTEELK | 1849 | 124.19 | 3 | 1590.7624 | 2 |  | 0 |  | yes | 6997.76 | 7689.46 | 7607.66 | 8066.27 |
| TFDQLSPDESK | 2543 | 67.19 | 3 | 1265.5773 | 2 |  | 0 |  | no | 3113.23 | 3549.10 | 3271.76 | 3614.48 |

  

| Tags | |
| --- | --- |

  

##

## P52480

KPYM\_MOUSE
Pyruvate kinase isozymes M1/M2 OS=Mus musculus GN=Pkm2 PE=1 SV=4  
7
peptides

  

| Sequence | Peptide Ion | Score | Hits | Mass | Charge | Tags | Conflicts | Modifications | In quantitation | Average Normalised Abundances | | | |
| --- | --- | --- | --- | --- | --- | --- | --- | --- | --- | --- | --- | --- | --- |
|  |  |  |  |  |  |  |  |  |  | 2m2f | 4m2f | 2m4f | 4m4f |
| DAVLNAWAEDVDLR | 4958 | 85.12 | 3 | 1585.7735 | 2 |  | 0 |  | yes | 2316.55 | 1667.33 | 2410.31 | 1809.84 |
| DAVLNAWAEDVDLR | 22105 | --- | --- | 1585.7724 | 3 |  | 0 |  | yes | 78.10 | 40.69 | 66.75 | 57.62 |
| EATESFASDPILYRPVAVALDTK | 6696 | 33.07 | 2 | 2492.2791 | 3 |  | 0 |  | yes | 4447.07 | 3482.84 | 2892.51 | 3667.18 |
| GADFLVTEVENGGSLGSK | 6817 | 44.81 | 3 | 1778.8691 | 2 |  | 0 |  | no | 1364.56 | 1061.97 | 1364.68 | 1059.81 |
| GVNLPGAAVDLPAVSEK | 6081 | 50.04 | 3 | 1635.8855 | 2 |  | 0 |  | no | 1419.23 | 800.66 | 1416.81 | 965.16 |
| IYVDDGLISLQVK | 5016 | 85.12 | 3 | 1461.8018 | 2 |  | 0 |  | no | 1690.31 | 1273.25 | 1689.68 | 717.53 |
| LAPITSDPTEAAAVGAVEASFK | 5487 | 29.25 | 2 | 2144.0981 | 3 |  | 0 |  | no | 2121.16 | 1257.36 | 1930.13 | 1274.86 |
| NTGIICTIGPASR | 3936 | 56.94 | 3 | 1358.6945 | 2 |  | 0 |  | yes | 2022.44 | 2599.07 | 1947.64 | 3439.13 |

  

| Tags | |
| --- | --- |

  

##

## P99024

TBB5\_MOUSE
Tubulin beta-5 chain OS=Mus musculus GN=Tubb5 PE=1 SV=1  
7
peptides

  

| Sequence | Peptide Ion | Score | Hits | Mass | Charge | Tags | Conflicts | Modifications | In quantitation | Average Normalised Abundances | | | |
| --- | --- | --- | --- | --- | --- | --- | --- | --- | --- | --- | --- | --- | --- |
|  |  |  |  |  |  |  |  |  |  | 2m2f | 4m2f | 2m4f | 4m4f |
| AILVDLEPGTMDSVR | 10310 | 41.17 | 3 | 1614.8297 | 2 |  | 1 |  | no | 696.01 | 412.82 | 740.55 | 529.83 |
| EVDEQMLNVQNK | 4065 | 61.26 | 3 | 1445.6826 | 2 |  | 1 |  | no | 2690.96 | 1834.69 | 2463.05 | 2691.48 |
| FPGQLNADLR | 4853 | 42.19 | 1 | 1129.5811 | 2 |  | 2 |  | no | 3425.65 | 960.87 | 1793.12 | 2408.56 |
| GHYTEGAELVDSVLDVVR | 5191 | 56.74 | 3 | 1957.9734 | 3 |  | 2 |  | no | 3212.66 | 859.58 | 2149.77 | 778.45 |
| IMNTFSVVPSPK | 7269 | 58.29 | 3 | 1318.6967 | 2 |  | 2 |  | no | 1171.12 | 647.80 | 1004.31 | 912.93 |
| LAVNMVPFPR | 3890 | 64.21 | 3 | 1142.6264 | 2 |  | 2 |  | no | 1222.96 | 766.57 | 1129.00 | 883.32 |
| NSSYFVEWIPNNVK | 3507 | 46.86 | 3 | 1695.8253 | 2 |  | 2 |  | no | 4303.55 | 2767.68 | 3878.40 | 3339.32 |
| NSSYFVEWIPNNVK | 11452 | --- | --- | 1695.8237 | 3 |  | 2 |  | no | 297.98 | 133.79 | 238.65 | 233.30 |

  

| Tags | |
| --- | --- |

  

##

## P24369

PPIB\_MOUSE
Peptidyl-prolyl cis-trans isomerase B OS=Mus musculus GN=Ppib PE=2
SV=2  
7 peptides

  

| Sequence | Peptide Ion | Score | Hits | Mass | Charge | Tags | Conflicts | Modifications | In quantitation | Average Normalised Abundances | | | |
| --- | --- | --- | --- | --- | --- | --- | --- | --- | --- | --- | --- | --- | --- |
|  |  |  |  |  |  |  |  |  |  | 2m2f | 4m2f | 2m4f | 4m4f |
| DFMIQGGDFTR | 5909 | 39.89 | 3 | 1285.5769 | 2 |  | 0 |  | no | 736.11 | 1114.16 | 790.68 | 945.42 |
| DTNGSQFFITTVK | 5229 | 59.06 | 3 | 1456.7192 | 2 |  | 0 |  | yes | 1186.65 | 1200.53 | 1306.78 | 1202.81 |
| DVIIVDSGK | 3300 | 31.24 | 3 | 944.5190 | 2 |  | 0 |  | yes | 1456.36 | 1487.32 | 1474.43 | 1452.80 |
| IEVEKPFAIAK | 12938 | 35.04 | 3 | 1243.7172 | 2 |  | 0 |  | no | 423.43 | 338.37 | 242.51 | 509.05 |
| TVDNFVALATGEK | 1844 | 90.33 | 3 | 1363.6976 | 2 |  | 0 |  | yes | 3559.52 | 4868.68 | 4208.09 | 4366.14 |
| VVFGLFGK | 3059 | 37.10 | 3 | 865.5058 | 2 |  | 0 |  | no | 1028.39 | 1173.97 | 1073.95 | 1130.92 |
| VYFDLQIGDESVGR | 7871 | 75.27 | 3 | 1596.7761 | 2 |  | 0 |  | no | 1095.32 | 578.10 | 709.91 | 360.06 |

  

| Tags | |
| --- | --- |

  

##

## Q64727

VINC\_MOUSE
Vinculin OS=Mus musculus GN=Vcl PE=1 SV=4  
7 peptides

  

| Sequence | Peptide Ion | Score | Hits | Mass | Charge | Tags | Conflicts | Modifications | In quantitation | Average Normalised Abundances | | | |
| --- | --- | --- | --- | --- | --- | --- | --- | --- | --- | --- | --- | --- | --- |
|  |  |  |  |  |  |  |  |  |  | 2m2f | 4m2f | 2m4f | 4m4f |
| ALASQLQDSLK | 6825 | 45.49 | 1 | 1172.6363 | 2 |  | 0 |  | yes | 697.67 | 522.51 | 781.77 | 520.19 |
| AVAGNISDPGLQK | 7821 | 47.71 | 2 | 1268.6647 | 2 |  | 0 |  | no | 585.58 | 459.36 | 622.11 | 479.67 |
| MSAEINEIIR | 12327 | 54.78 | 2 | 1174.5955 | 2 |  | 0 |  | no | 327.31 | 114.59 | 198.81 | 128.69 |
| MTGLVDEAIDTK | 6463 | 65.76 | 2 | 1291.6325 | 2 |  | 0 |  | yes | 808.20 | 613.04 | 857.94 | 574.90 |
| SLGEIAALTSK | 6586 | 50.87 | 2 | 1088.6067 | 2 |  | 0 |  | no | 582.12 | 391.33 | 648.75 | 489.19 |
| VLQLTSWDEDAWASK | 8446 | 67.40 | 3 | 1747.8415 | 2 |  | 0 |  | yes | 1069.57 | 366.88 | 1175.04 | 689.02 |
| WIDNPTVDDR | 6317 | 32.34 | 2 | 1229.5667 | 2 |  | 0 |  | no | 467.16 | 537.34 | 524.78 | 847.22 |

  

| Tags | |
| --- | --- |

  

##

## Q9CQ19

MYL9\_MOUSE
Myosin regulatory light polypeptide 9 OS=Mus musculus GN=Myl9 PE=1
SV=3  
6 peptides

  

| Sequence | Peptide Ion | Score | Hits | Mass | Charge | Tags | Conflicts | Modifications | In quantitation | Average Normalised Abundances | | | |
| --- | --- | --- | --- | --- | --- | --- | --- | --- | --- | --- | --- | --- | --- |
|  |  |  |  |  |  |  |  |  |  | 2m2f | 4m2f | 2m4f | 4m4f |
| ATSNVFAMFDQSQIQEFK | 7613 | 47.18 | 3 | 2089.9767 | 3 |  | 0 |  | no | 1202.13 | 178.72 | 1233.90 | 468.30 |
| ATSNVFAMFDQSQIQEFK | 11701 | 29.92 | 3 | 2089.9800 | 2 |  | 0 |  | no | 1031.38 | 234.52 | 1102.73 | 412.30 |
| DGFIDKEDLHDMLASLGK | 13024 | 34.03 | 1 | 2002.9674 | 3 |  | 0 |  | no | 358.39 | 42.81 | 260.64 | 109.47 |
| EAFNMIDQNR | 4552 | 65.79 | 3 | 1236.5554 | 2 |  | 0 |  | no | 1798.29 | 291.54 | 1224.30 | 667.69 |
| FTDEEVDEMYR | 3598 | 71.95 | 3 | 1432.5812 | 2 |  | 0 |  | yes | 2235.78 | 825.37 | 2091.18 | 1813.92 |
| GNFNYVEFTR | 2738 | 59.02 | 3 | 1245.5788 | 2 |  | 0 |  | yes | 2420.75 | 1023.32 | 2271.26 | 1581.16 |
| LNGTDPEDVIR | 2949 | 55.67 | 3 | 1227.6093 | 2 |  | 0 |  | yes | 3436.72 | 1815.82 | 2821.32 | 2986.30 |

  

| Tags | |
| --- | --- |

  

##

## P19001

K1C19\_MOUSE
Keratin, type I cytoskeletal 19 OS=Mus musculus GN=Krt19 PE=2 SV=1  
6
peptides

  

| Sequence | Peptide Ion | Score | Hits | Mass | Charge | Tags | Conflicts | Modifications | In quantitation | Average Normalised Abundances | | | |
| --- | --- | --- | --- | --- | --- | --- | --- | --- | --- | --- | --- | --- | --- |
|  |  |  |  |  |  |  |  |  |  | 2m2f | 4m2f | 2m4f | 4m4f |
| ALEQANGELEVK | 4676 | 67.52 | 3 | 1299.6637 | 2 |  | 0 |  | yes | 1566.37 | 1072.46 | 1255.96 | 1972.53 |
| GGSFSGTLAVSDGLLSGNEK | 10447 | 77.17 | 2 | 1894.9315 | 2 |  | 0 |  | no | 718.29 | 200.65 | 605.91 | 154.78 |
| IVLQIDNAR | 1700 | 42.43 | 1 | 1040.5936 | 2 |  | 1 |  | yes | 3347.26 | 3544.67 | 3095.19 | 3529.25 |
| LAADDFR | 2306 | 38.29 | 3 | 806.3962 | 2 |  | 1 |  | no | 1748.71 | 1860.41 | 1598.20 | 1781.20 |
| LEQEIATYR | 4279 | 63.95 | 3 | 1121.5710 | 2 |  | 0 |  | no | 1116.82 | 1230.84 | 1060.52 | 1271.71 |
| VLDELTLAR | 3112 | 69.72 | 3 | 1028.5885 | 2 |  | 0 |  | yes | 1452.69 | 1053.21 | 1081.44 | 1450.56 |

  

| Tags | |
| --- | --- |

  

##

## P68369

TBA1A\_MOUSE
Tubulin alpha-1A chain OS=Mus musculus GN=Tuba1a PE=1 SV=1  
6
peptides

  

| Sequence | Peptide Ion | Score | Hits | Mass | Charge | Tags | Conflicts | Modifications | In quantitation | Average Normalised Abundances | | | |
| --- | --- | --- | --- | --- | --- | --- | --- | --- | --- | --- | --- | --- | --- |
|  |  |  |  |  |  |  |  |  |  | 2m2f | 4m2f | 2m4f | 4m4f |
| AVCMLSNTTAIAEAWAR | 10219 | 30.97 | 1 | 1863.8979 | 3 |  | 2 |  | no | 489.45 | 154.15 | 459.53 | 174.45 |
| AVFVDLEPTVIDEVR | 2682 | 70.69 | 3 | 1700.8976 | 2 |  | 1 |  | no | 5851.99 | 3681.06 | 6002.92 | 4706.44 |
| AVFVDLEPTVIDEVR | 4611 | 42.52 | 3 | 1700.8970 | 3 |  | 1 |  | no | 1328.90 | 771.41 | 1477.24 | 1105.80 |
| EDAANNYAR | 5692 | 63.88 | 3 | 1022.4410 | 2 |  | 2 |  | no | 809.45 | 505.77 | 667.00 | 489.55 |
| TIGGGDDSFNTFFSETGAGK | 4419 | 33.11 | 3 | 2006.8863 | 2 |  | 1 |  | no | 3267.06 | 1979.74 | 3420.21 | 2609.16 |
| TIQFVDWCPTGFK | 4545 | 59.40 | 3 | 1597.7595 | 2 |  | 0 |  | yes | 2810.92 | 1938.93 | 2219.70 | 2730.08 |
| VGINYQPPTVVPGGDLAK | 6184 | --- | --- | 1823.9775 | 3 |  | 2 |  | no | 1272.27 | 776.10 | 1234.45 | 968.21 |
| VGINYQPPTVVPGGDLAK | 3505 | 55.61 | 3 | 1823.9780 | 2 |  | 2 |  | no | 5432.91 | 3727.29 | 4970.77 | 4486.65 |

  

| Tags | |
| --- | --- |

  

##

## P05213

TBA1B\_MOUSE
Tubulin alpha-1B chain OS=Mus musculus GN=Tuba1b PE=1 SV=2  
6
peptides

  

| Sequence | Peptide Ion | Score | Hits | Mass | Charge | Tags | Conflicts | Modifications | In quantitation | Average Normalised Abundances | | | |
| --- | --- | --- | --- | --- | --- | --- | --- | --- | --- | --- | --- | --- | --- |
|  |  |  |  |  |  |  |  |  |  | 2m2f | 4m2f | 2m4f | 4m4f |
| AVCMLSNTTAIAEAWAR | 10219 | 30.97 | 1 | 1863.8979 | 3 |  | 2 |  | no | 489.45 | 154.15 | 459.53 | 174.45 |
| AVFVDLEPTVIDEVR | 2682 | 70.69 | 3 | 1700.8976 | 2 |  | 1 |  | no | 5851.99 | 3681.06 | 6002.92 | 4706.44 |
| AVFVDLEPTVIDEVR | 4611 | 42.52 | 3 | 1700.8970 | 3 |  | 1 |  | no | 1328.90 | 771.41 | 1477.24 | 1105.80 |
| EDAANNYAR | 5692 | 63.88 | 3 | 1022.4410 | 2 |  | 2 |  | no | 809.45 | 505.77 | 667.00 | 489.55 |
| SIQFVDWCPTGFK | 4066 | 58.42 | 3 | 1583.7403 | 2 |  | 1 |  | no | 3563.58 | 1848.50 | 2984.33 | 2759.19 |
| TIGGGDDSFNTFFSETGAGK | 4419 | 33.11 | 3 | 2006.8863 | 2 |  | 1 |  | no | 3267.06 | 1979.74 | 3420.21 | 2609.16 |
| VGINYQPPTVVPGGDLAK | 6184 | --- | --- | 1823.9775 | 3 |  | 2 |  | no | 1272.27 | 776.10 | 1234.45 | 968.21 |
| VGINYQPPTVVPGGDLAK | 3505 | 55.61 | 3 | 1823.9780 | 2 |  | 2 |  | no | 5432.91 | 3727.29 | 4970.77 | 4486.65 |

  

| Tags | |
| --- | --- |

  

##

## O54734

OST48\_MOUSE
Dolichyl-diphosphooligosaccharide--protein glycosyltransferase 48 kDa subunit
OS=Mus musculus GN=Ddost PE=1 SV=1  
5 peptides

  

| Sequence | Peptide Ion | Score | Hits | Mass | Charge | Tags | Conflicts | Modifications | In quantitation | Average Normalised Abundances | | | |
| --- | --- | --- | --- | --- | --- | --- | --- | --- | --- | --- | --- | --- | --- |
|  |  |  |  |  |  |  |  |  |  | 2m2f | 4m2f | 2m4f | 4m4f |
| ELGSECGIEFDEEK | 3851 | 92.88 | 3 | 1640.6873 | 2 |  | 0 |  | yes | 2988.55 | 2527.66 | 2692.77 | 2496.80 |
| SSLNPILFR | 2823 | 68.74 | 3 | 1045.5919 | 2 |  | 0 |  | no | 1319.89 | 1373.24 | 1695.93 | 1532.99 |
| TLVLLDNLNVR | 2766 | 75.13 | 3 | 1268.7433 | 2 |  | 0 |  | yes | 3123.34 | 2225.95 | 3183.42 | 1566.73 |
| WVPFDGDDIQLEFVR | 10880 | 32.66 | 3 | 1834.8880 | 2 |  | 0 |  | yes | 2467.86 | 702.64 | 1953.38 | 914.49 |
| YSQTGNYELAVALSR | 8855 | 83.63 | 3 | 1670.8240 | 2 |  | 0 |  | no | 945.32 | 546.51 | 883.34 | 283.14 |

  

| Tags | |
| --- | --- |

  

##

## Q60605

MYL6\_MOUSE
Myosin light polypeptide 6 OS=Mus musculus GN=Myl6 PE=1 SV=3  
6
peptides

  

| Sequence | Peptide Ion | Score | Hits | Mass | Charge | Tags | Conflicts | Modifications | In quantitation | Average Normalised Abundances | | | |
| --- | --- | --- | --- | --- | --- | --- | --- | --- | --- | --- | --- | --- | --- |
|  |  |  |  |  |  |  |  |  |  | 2m2f | 4m2f | 2m4f | 4m4f |
| ALGQNPTNAEVLK | 1062 | 62.69 | 3 | 1353.7225 | 2 |  | 0 |  | yes | 8927.92 | 4110.54 | 8672.41 | 6836.50 |
| DQGTYEDYVEGLR | 2690 | 81.45 | 3 | 1543.6823 | 2 |  | 0 |  | yes | 4919.47 | 2382.56 | 3780.99 | 3181.12 |
| EAFQLFDR | 2290 | 53.81 | 3 | 1024.4990 | 2 |  | 0 |  | no | 1845.46 | 1126.91 | 2175.94 | 1842.50 |
| HVLVTLGEK | 1695 | 58.38 | 3 | 994.5807 | 2 |  | 0 |  | no | 2123.62 | 742.64 | 2184.54 | 1157.23 |
| ILYSQCGDVMR | 3032 | 65.75 | 3 | 1340.6216 | 2 |  | 0 |  | yes | 3848.58 | 2099.93 | 3329.18 | 3367.43 |
| VLDFEHFLPMLQTVAK | 10424 | 30.17 | 3 | 1886.9956 | 3 |  | 0 |  | no | 1705.70 | 179.86 | 1800.38 | 501.47 |

  

| Tags | |
| --- | --- |

  

##

## P17751

TPIS\_MOUSE
Triosephosphate isomerase OS=Mus musculus GN=Tpi1 PE=1 SV=3  
7
peptides

  

| Sequence | Peptide Ion | Score | Hits | Mass | Charge | Tags | Conflicts | Modifications | In quantitation | Average Normalised Abundances | | | |
| --- | --- | --- | --- | --- | --- | --- | --- | --- | --- | --- | --- | --- | --- |
|  |  |  |  |  |  |  |  |  |  | 2m2f | 4m2f | 2m4f | 4m4f |
| DLGATWVVLGHSER | 6218 | 31.24 | 3 | 1538.7826 | 3 |  | 0 |  | no | 559.54 | 254.05 | 544.53 | 408.30 |
| HVFGESDELIGQK | 6287 | 43.49 | 2 | 1457.7134 | 3 |  | 0 |  | no | 789.51 | 487.95 | 519.41 | 654.54 |
| IIYGGSVTGATCK | 2584 | 83.83 | 3 | 1325.6664 | 2 |  | 0 |  | yes | 2996.61 | 2797.56 | 2787.94 | 2253.70 |
| SNVNDGVAQSTR | 3222 | 65.10 | 3 | 1246.5899 | 2 |  | 0 |  | yes | 3841.82 | 2462.24 | 2808.36 | 2163.57 |
| TATPQQAQEVHEK | 25039 | --- | --- | 1465.7160 | 2 |  | 0 |  | no | 89.34 | 5.94 | 56.96 | 19.94 |
| TATPQQAQEVHEK | 3937 | 25.37 | 3 | 1465.7151 | 3 |  | 0 |  | no | 1700.23 | 1153.52 | 1362.44 | 1340.74 |
| VTNGAFTGEISPGMIK | 7725 | 39.28 | 3 | 1620.8169 | 2 |  | 0 |  | no | 978.03 | 1024.71 | 982.45 | 905.80 |
| VVLAYEPVWAIGTGK | 2078 | 63.26 | 3 | 1601.8814 | 2 |  | 0 |  | yes | 7954.56 | 6292.32 | 7228.44 | 6805.55 |
| VVLAYEPVWAIGTGK | 5779 | --- | --- | 1601.8805 | 3 |  | 0 |  | yes | 1029.85 | 684.85 | 945.87 | 834.91 |

  

| Tags | |
| --- | --- |

  

##

## P02089

HBB2\_MOUSE
Hemoglobin subunit beta-2 OS=Mus musculus GN=Hbb-b2 PE=1 SV=2  
4
peptides

  

| Sequence | Peptide Ion | Score | Hits | Mass | Charge | Tags | Conflicts | Modifications | In quantitation | Average Normalised Abundances | | | |
| --- | --- | --- | --- | --- | --- | --- | --- | --- | --- | --- | --- | --- | --- |
|  |  |  |  |  |  |  |  |  |  | 2m2f | 4m2f | 2m4f | 4m4f |
| GTFASLSELHCDK | 829 | 50.77 | 3 | 1463.6704 | 3 |  | 1 |  | yes | 5867.32 | 5901.61 | 6333.34 | 6252.57 |
| GTFASLSELHCDK | 2299 | 90.91 | 3 | 1463.6715 | 2 |  | 1 |  | yes | 3361.84 | 2869.32 | 3048.00 | 3364.46 |
| LHVDPENFR | 1887 | 38.89 | 3 | 1125.5569 | 3 |  | 1 |  | no | 1551.18 | 1065.25 | 1642.45 | 1162.82 |
| LHVDPENFR | 2189 | 41.88 | 3 | 1125.5556 | 2 |  | 1 |  | no | 2681.34 | 2011.50 | 2456.02 | 1989.18 |
| LLVVYPWTQR | 4996 | 28.90 | 3 | 1273.7181 | 3 |  | 1 |  | yes | 523.74 | 654.52 | 878.85 | 593.31 |
| LLVVYPWTQR | 176 | 56.35 | 3 | 1273.7178 | 2 |  | 1 |  | yes | 6.19e+004 | 8.20e+004 | 9.59e+004 | 6.69e+004 |
| YFDSFGDLSSASAIMGNPK | 18391 | --- | --- | 2005.9085 | 3 |  | 0 |  | yes | 116.55 | 336.36 | 71.57 | 174.06 |
| YFDSFGDLSSASAIMGNPK | 11150 | 43.05 | 3 | 2005.9093 | 2 |  | 0 |  | yes | 866.75 | 1422.50 | 648.95 | 1291.69 |

  

| Tags | |
| --- | --- |

  

##

## P35980

RL18\_MOUSE
60S ribosomal protein L18 OS=Mus musculus GN=Rpl18 PE=2 SV=3  
5
peptides

  

| Sequence | Peptide Ion | Score | Hits | Mass | Charge | Tags | Conflicts | Modifications | In quantitation | Average Normalised Abundances | | | |
| --- | --- | --- | --- | --- | --- | --- | --- | --- | --- | --- | --- | --- | --- |
|  |  |  |  |  |  |  |  |  |  | 2m2f | 4m2f | 2m4f | 4m4f |
| GTVLLSGPR | 1023 | 41.06 | 3 | 898.5233 | 2 |  | 0 |  | yes | 5982.91 | 8027.16 | 7554.05 | 7975.24 |
| ILTFDQLALESPK | 1731 | 118.53 | 3 | 1473.8077 | 2 |  | 0 |  | yes | 7171.55 | 6628.29 | 8224.47 | 6454.20 |
| TAVVVGTVTDDVR | 1846 | 96.37 | 3 | 1330.7103 | 2 |  | 0 |  | yes | 3939.95 | 4120.17 | 3963.39 | 4244.41 |
| TNRPPLSLSR | 6508 | 34.61 | 3 | 1139.6411 | 3 |  | 0 |  | no | 412.08 | 252.13 | 339.00 | 406.30 |
| TNSTFNQVVLK | 2547 | 57.39 | 2 | 1249.6596 | 2 |  | 0 |  | no | 4103.57 | 4149.84 | 3706.45 | 4295.75 |

  

| Tags | |
| --- | --- |

  

##

## Q9ERD7

TBB3\_MOUSE
Tubulin beta-3 chain OS=Mus musculus GN=Tubb3 PE=1 SV=1  
7
peptides

  

| Sequence | Peptide Ion | Score | Hits | Mass | Charge | Tags | Conflicts | Modifications | In quantitation | Average Normalised Abundances | | | |
| --- | --- | --- | --- | --- | --- | --- | --- | --- | --- | --- | --- | --- | --- |
|  |  |  |  |  |  |  |  |  |  | 2m2f | 4m2f | 2m4f | 4m4f |
| AILVDLEPGTMDSVR | 10310 | 41.17 | 3 | 1614.8297 | 2 |  | 1 |  | no | 696.01 | 412.82 | 740.55 | 529.83 |
| ALTVPELTQQMFDAK | 8402 | 25.04 | 3 | 1690.8600 | 2 |  | 1 |  | no | 2156.73 | 1131.60 | 2041.37 | 1496.81 |
| FPGQLNADLR | 4853 | 42.19 | 1 | 1129.5811 | 2 |  | 2 |  | no | 3425.65 | 960.87 | 1793.12 | 2408.56 |
| GHYTEGAELVDSVLDVVR | 5191 | 56.74 | 3 | 1957.9734 | 3 |  | 2 |  | no | 3212.66 | 859.58 | 2149.77 | 778.45 |
| IMNTFSVVPSPK | 7269 | 58.29 | 3 | 1318.6967 | 2 |  | 2 |  | no | 1171.12 | 647.80 | 1004.31 | 912.93 |
| LAVNMVPFPR | 3890 | 64.21 | 3 | 1142.6264 | 2 |  | 2 |  | no | 1222.96 | 766.57 | 1129.00 | 883.32 |
| NSSYFVEWIPNNVK | 3507 | 46.86 | 3 | 1695.8253 | 2 |  | 2 |  | no | 4303.55 | 2767.68 | 3878.40 | 3339.32 |
| NSSYFVEWIPNNVK | 11452 | --- | --- | 1695.8237 | 3 |  | 2 |  | no | 297.98 | 133.79 | 238.65 | 233.30 |

  

| Tags | |
| --- | --- |

  

##

## P16858

G3P\_MOUSE
Glyceraldehyde-3-phosphate dehydrogenase OS=Mus musculus GN=Gapdh PE=1
SV=2  
6 peptides

  

| Sequence | Peptide Ion | Score | Hits | Mass | Charge | Tags | Conflicts | Modifications | In quantitation | Average Normalised Abundances | | | |
| --- | --- | --- | --- | --- | --- | --- | --- | --- | --- | --- | --- | --- | --- |
|  |  |  |  |  |  |  |  |  |  | 2m2f | 4m2f | 2m4f | 4m4f |
| GAAQNIIPASTGAAK | 1723 | 33.63 | 3 | 1368.7331 | 2 |  | 0 |  | yes | 5804.70 | 6987.41 | 6705.51 | 6062.93 |
| IVSNASCTTNCLAPLAK | 9085 | --- | --- | 1818.8944 | 3 |  | 0 |  | no | 745.47 | 332.43 | 395.79 | 293.83 |
| IVSNASCTTNCLAPLAK | 6692 | 51.45 | 3 | 1818.8890 | 2 |  | 0 |  | no | 1817.75 | 1579.85 | 1669.43 | 1518.23 |
| LISWYDNEYGYSNR | 3585 | 65.96 | 3 | 1778.7899 | 2 |  | 0 |  | yes | 4766.55 | 5099.45 | 6168.81 | 3931.64 |
| LVINGKPITIFQER | 4242 | 45.26 | 3 | 1626.9449 | 3 |  | 0 |  | no | 2613.08 | 3360.36 | 4233.33 | 4757.26 |
| LVINGKPITIFQER | 27383 | --- | --- | 1626.9434 | 2 |  | 0 |  | no | 165.65 | 0.00 | 81.66 | 57.82 |
| VPTPNVSVVDLTCR | 8098 | --- | --- | 1555.8021 | 3 |  | 0 |  | yes | 373.45 | 227.64 | 374.40 | 297.27 |
| VPTPNVSVVDLTCR | 2409 | 84.27 | 3 | 1555.8024 | 2 |  | 0 |  | yes | 4191.42 | 3952.25 | 4497.60 | 4174.91 |
| WGEAGAEYVVESTGVFTTMEK | 6104 | 50.83 | 3 | 2290.0447 | 3 |  | 0 |  | no | 2371.74 | 909.57 | 2393.20 | 549.50 |

  

| Tags | |
| --- | --- |

  

##

## Q9R0P5

DEST\_MOUSE
Destrin OS=Mus musculus GN=Dstn PE=1 SV=3  
5 peptides

  

| Sequence | Peptide Ion | Score | Hits | Mass | Charge | Tags | Conflicts | Modifications | In quantitation | Average Normalised Abundances | | | |
| --- | --- | --- | --- | --- | --- | --- | --- | --- | --- | --- | --- | --- | --- |
|  |  |  |  |  |  |  |  |  |  | 2m2f | 4m2f | 2m4f | 4m4f |
| AVIFCLSADK | 3299 | 84.84 | 3 | 1122.5704 | 2 |  | 0 |  | no | 1768.82 | 1583.64 | 1875.72 | 1401.62 |
| EILVGDVGATITDPFK | 6149 | --- | --- | 1673.8878 | 3 |  | 0 |  | yes | 1646.42 | 900.19 | 1580.95 | 468.37 |
| EILVGDVGATITDPFK | 1969 | 84.82 | 2 | 1673.8881 | 2 |  | 0 |  | yes | 1.50e+004 | 9325.31 | 1.50e+004 | 5167.31 |
| HEYQANGPEDLNR | 4422 | 52.96 | 2 | 1541.6803 | 3 |  | 0 |  | yes | 3668.20 | 3896.59 | 3768.42 | 5123.73 |
| HFVGMLPEK | 3092 | 40.44 | 3 | 1056.5427 | 2 |  | 0 |  | no | 2084.23 | 1331.56 | 1414.83 | 1335.43 |
| YALYDASFETK | 1103 | 66.52 | 3 | 1306.6064 | 2 |  | 0 |  | yes | 7549.54 | 7153.61 | 7155.17 | 8361.49 |

  

| Tags | |
| --- | --- |

  

##

## Q99KI0

ACON\_MOUSE
Aconitate hydratase, mitochondrial OS=Mus musculus GN=Aco2 PE=1 SV=1  
6
peptides

  

| Sequence | Peptide Ion | Score | Hits | Mass | Charge | Tags | Conflicts | Modifications | In quantitation | Average Normalised Abundances | | | |
| --- | --- | --- | --- | --- | --- | --- | --- | --- | --- | --- | --- | --- | --- |
|  |  |  |  |  |  |  |  |  |  | 2m2f | 4m2f | 2m4f | 4m4f |
| DINQEVYNFLATAGAK | 10127 | 31.96 | 3 | 1752.8676 | 3 |  | 0 |  | yes | 852.44 | 238.10 | 634.13 | 394.26 |
| DINQEVYNFLATAGAK | 14256 | 54.14 | 2 | 1752.8742 | 2 |  | 0 |  | yes | 842.00 | 141.07 | 571.78 | 367.20 |
| DLEDLQILIK | 7950 | 54.59 | 3 | 1198.6740 | 2 |  | 0 |  | no | 980.41 | 612.29 | 939.17 | 432.20 |
| IVYGHLDDPANQEIER | 8073 | 38.41 | 2 | 1867.9056 | 3 |  | 0 |  | no | 680.93 | 465.05 | 567.35 | 456.59 |
| SQFTITPGSEQIR | 5761 | 31.67 | 2 | 1462.7421 | 2 |  | 0 |  | no | 1022.63 | 915.03 | 953.84 | 667.76 |
| TDIANLAEEFK | 5259 | 64.65 | 3 | 1249.6187 | 2 |  | 0 |  | yes | 1132.65 | 1062.08 | 1030.36 | 946.13 |
| WVVIGDENYGEGSSR | 7403 | 44.05 | 3 | 1666.7586 | 2 |  | 0 |  | yes | 1504.44 | 1559.38 | 1249.55 | 957.94 |

  

| Tags | |
| --- | --- |

  

##

## Q8VCT3

AMPB\_MOUSE
Aminopeptidase B OS=Mus musculus GN=Rnpep PE=2 SV=1  
5
peptides

  

| Sequence | Peptide Ion | Score | Hits | Mass | Charge | Tags | Conflicts | Modifications | In quantitation | Average Normalised Abundances | | | |
| --- | --- | --- | --- | --- | --- | --- | --- | --- | --- | --- | --- | --- | --- |
|  |  |  |  |  |  |  |  |  |  | 2m2f | 4m2f | 2m4f | 4m4f |
| AFFPCFDTPAVK | 4516 | 52.10 | 3 | 1398.6698 | 2 |  | 0 |  | yes | 2088.62 | 1627.02 | 1885.04 | 1965.53 |
| EEYSGVIEEFLATGEK | 8761 | 63.99 | 3 | 1799.8484 | 2 |  | 0 |  | yes | 2095.88 | 777.31 | 1723.08 | 1220.82 |
| EEYSGVIEEFLATGEK | 12442 | 34.30 | 2 | 1799.8539 | 3 |  | 0 |  | yes | 535.99 | 274.31 | 483.28 | 325.20 |
| ISNAQNAELR | 6841 | 83.08 | 3 | 1114.5722 | 2 |  | 0 |  | no | 562.28 | 507.73 | 491.18 | 528.02 |
| TYQLVYFLDK | 6693 | 48.02 | 3 | 1288.6723 | 2 |  | 0 |  | yes | 2007.73 | 2994.64 | 2043.46 | 3081.54 |
| VWAEPCLIEAAK | 4538 | 29.73 | 3 | 1385.7071 | 2 |  | 1 |  | no | 1844.52 | 2023.86 | 2411.09 | 1912.56 |

  

| Tags | |
| --- | --- |

  

##

## Q8CEK3

SPIKL\_MOUSE
Serine protease inhibitor kazal-like protein, minor form OS=Mus musculus PE=1
SV=1  
3 peptides

  

| Sequence | Peptide Ion | Score | Hits | Mass | Charge | Tags | Conflicts | Modifications | In quantitation | Average Normalised Abundances | | | |
| --- | --- | --- | --- | --- | --- | --- | --- | --- | --- | --- | --- | --- | --- |
|  |  |  |  |  |  |  |  |  |  | 2m2f | 4m2f | 2m4f | 4m4f |
| NTYYNECYFCIEK | 91 | 87.03 | 3 | 1802.7279 | 2 |  | 0 |  | yes | 2.47e+005 | 5.15e+005 | 3.38e+005 | 3.63e+005 |
| SECSNIAENPVCADDR | 247 | 66.05 | 3 | 1835.7408 | 3 |  | 0 |  | yes | 5.02e+004 | 8.61e+004 | 5.32e+004 | 8.30e+004 |
| SECSNIAENPVCADDR | 114 | 96.03 | 3 | 1835.7416 | 2 |  | 0 |  | yes | 2.18e+005 | 3.94e+005 | 2.42e+005 | 3.69e+005 |
| SKSECSNIAENPVCADDR | 763 | 44.73 | 3 | 2050.8675 | 3 |  | 0 |  | yes | 1.82e+004 | 2.57e+004 | 1.90e+004 | 2.45e+004 |

  

| Tags | |
| --- | --- |

  

##

## Q68FD5

CLH\_MOUSE
Clathrin heavy chain 1 OS=Mus musculus GN=Cltc PE=1 SV=3  
7
peptides

  

| Sequence | Peptide Ion | Score | Hits | Mass | Charge | Tags | Conflicts | Modifications | In quantitation | Average Normalised Abundances | | | |
| --- | --- | --- | --- | --- | --- | --- | --- | --- | --- | --- | --- | --- | --- |
|  |  |  |  |  |  |  |  |  |  | 2m2f | 4m2f | 2m4f | 4m4f |
| ALEHFTDLYDIK | 13937 | 26.28 | 1 | 1463.7290 | 3 |  | 0 |  | no | 139.94 | 37.93 | 83.25 | 114.39 |
| GQFSTDELVAEVEK | 8267 | 45.11 | 1 | 1550.7493 | 2 |  | 0 |  | yes | 677.37 | 334.24 | 660.01 | 500.83 |
| ISGETIFVTAPHEATAGIIGVNR | 16332 | 43.38 | 1 | 2352.2399 | 3 |  | 0 |  | no | 307.79 | 2.38 | 233.74 | 117.50 |
| LPVVIGGLLDVDCSEDVIK | 16488 | 41.50 | 2 | 2040.0822 | 2 |  | 0 |  | yes | 884.60 | 202.83 | 500.92 | 295.92 |
| LPVVIGGLLDVDCSEDVIK | 16282 | --- | --- | 2040.0797 | 3 |  | 0 |  | yes | 467.44 | 68.07 | 308.18 | 129.39 |
| NLQNLLILTAIK | 9129 | 56.55 | 3 | 1352.8386 | 2 |  | 0 |  | yes | 955.61 | 331.13 | 812.78 | 585.38 |
| NNLAGAEELFAR | 9519 | 35.60 | 3 | 1303.6505 | 2 |  | 0 |  | no | 501.47 | 359.73 | 505.85 | 429.59 |
| VGYTPDWIFLLR | 15531 | 35.04 | 1 | 1478.7908 | 2 |  | 0 |  | no | 506.71 | 69.84 | 429.28 | 322.07 |

  

| Tags | |
| --- | --- |

  

##

## Q9JKR6

HYOU1\_MOUSE
Hypoxia up-regulated protein 1 OS=Mus musculus GN=Hyou1 PE=1 SV=1  
6
peptides

  

| Sequence | Peptide Ion | Score | Hits | Mass | Charge | Tags | Conflicts | Modifications | In quantitation | Average Normalised Abundances | | | |
| --- | --- | --- | --- | --- | --- | --- | --- | --- | --- | --- | --- | --- | --- |
|  |  |  |  |  |  |  |  |  |  | 2m2f | 4m2f | 2m4f | 4m4f |
| DAVIYPILVEFTR | 17679 | --- | --- | 1534.8381 | 3 |  | 0 |  | yes | 536.52 | 120.35 | 477.56 | 309.34 |
| DAVIYPILVEFTR | 9738 | 31.10 | 3 | 1534.8390 | 2 |  | 0 |  | yes | 3467.81 | 1099.14 | 2920.75 | 2117.97 |
| LGNTISSLFGGGTSSDAK | 6406 | 57.09 | 3 | 1710.8414 | 2 |  | 0 |  | yes | 1606.03 | 1009.91 | 1608.77 | 1498.33 |
| SLAEDFAEQPIK | 6059 | 47.73 | 1 | 1346.6718 | 2 |  | 0 |  | no | 1222.83 | 1121.30 | 1123.90 | 1333.33 |
| VEFEELCADLFDR | 10243 | 76.38 | 3 | 1641.7346 | 2 |  | 0 |  | no | 1116.90 | 258.35 | 967.86 | 433.42 |
| VESVFETLVEDSPEEESTLTK | 4038 | 26.26 | 3 | 2367.1206 | 3 |  | 0 |  | yes | 2360.84 | 1641.76 | 3062.04 | 1978.78 |
| VLQLINDNTATALSYGVFR | 8328 | 43.60 | 3 | 2094.1116 | 3 |  | 0 |  | no | 1291.05 | 501.75 | 1037.79 | 419.90 |
| VLQLINDNTATALSYGVFR | 18412 | --- | --- | 2094.1137 | 2 |  | 0 |  | no | 465.36 | 158.57 | 369.12 | 186.25 |

  

| Tags | |
| --- | --- |

  

##

## Q99K85

SERC\_MOUSE
Phosphoserine aminotransferase OS=Mus musculus GN=Psat1 PE=1 SV=1  
5
peptides

  

| Sequence | Peptide Ion | Score | Hits | Mass | Charge | Tags | Conflicts | Modifications | In quantitation | Average Normalised Abundances | | | |
| --- | --- | --- | --- | --- | --- | --- | --- | --- | --- | --- | --- | --- | --- |
|  |  |  |  |  |  |  |  |  |  | 2m2f | 4m2f | 2m4f | 4m4f |
| ASLYNAVTTEDVEK | 5186 | 59.88 | 3 | 1538.7444 | 2 |  | 0 |  | yes | 1518.47 | 1773.15 | 1515.46 | 1210.80 |
| ELLAVPNNYK | 6985 | 33.90 | 3 | 1159.6235 | 2 |  | 0 |  | yes | 652.36 | 715.24 | 628.58 | 526.41 |
| IIGNTENLVR | 4699 | 43.42 | 3 | 1127.6293 | 2 |  | 0 |  | yes | 910.20 | 855.45 | 806.67 | 717.11 |
| NVGSAGVTVVIVR | 7300 | 74.30 | 3 | 1269.7398 | 2 |  | 0 |  | no | 430.96 | 492.71 | 624.75 | 284.33 |
| SADYVVTGAWSAK | 6949 | 67.75 | 3 | 1353.6565 | 2 |  | 0 |  | no | 544.29 | 558.95 | 600.58 | 406.30 |

  

| Tags | |
| --- | --- |

  

##

## P61205

ARF3\_MOUSE
ADP-ribosylation factor 3 OS=Mus musculus GN=Arf3 PE=2 SV=2  
5
peptides

  

| Sequence | Peptide Ion | Score | Hits | Mass | Charge | Tags | Conflicts | Modifications | In quantitation | Average Normalised Abundances | | | |
| --- | --- | --- | --- | --- | --- | --- | --- | --- | --- | --- | --- | --- | --- |
|  |  |  |  |  |  |  |  |  |  | 2m2f | 4m2f | 2m4f | 4m4f |
| DAVLLVFANK | 2810 | 63.96 | 3 | 1088.6223 | 2 |  | 1 |  | no | 1536.42 | 1734.65 | 2197.54 | 1683.10 |
| HYFQNTQGLIFVVDSNDR | 9786 | 33.31 | 2 | 2152.0324 | 3 |  | 1 |  | no | 513.36 | 170.07 | 623.46 | 228.03 |
| ILMVGLDAAGK | 2146 | 59.52 | 3 | 1086.6100 | 2 |  | 1 |  | yes | 2364.09 | 2405.79 | 2932.69 | 2010.72 |
| LGEIVTTIPTIGFNVETVEYK | 3105 | 49.10 | 3 | 2322.2352 | 3 |  | 1 |  | yes | 7810.86 | 2676.54 | 6734.56 | 1989.72 |
| LGEIVTTIPTIGFNVETVEYK | 9817 | --- | --- | 2322.2375 | 2 |  | 1 |  | yes | 2476.51 | 820.06 | 2136.25 | 996.88 |
| NISFTVWDVGGQDK | 5036 | 69.64 | 3 | 1564.7488 | 2 |  | 0 |  | yes | 2238.19 | 3141.16 | 2797.68 | 1582.25 |

  

| Tags | |
| --- | --- |

  

##

## P09411

PGK1\_MOUSE
Phosphoglycerate kinase 1 OS=Mus musculus GN=Pgk1 PE=1 SV=4  
6
peptides

  

| Sequence | Peptide Ion | Score | Hits | Mass | Charge | Tags | Conflicts | Modifications | In quantitation | Average Normalised Abundances | | | |
| --- | --- | --- | --- | --- | --- | --- | --- | --- | --- | --- | --- | --- | --- |
|  |  |  |  |  |  |  |  |  |  | 2m2f | 4m2f | 2m4f | 4m4f |
| ALESPERPFLAILGGAK | 8155 | 31.14 | 3 | 1767.9861 | 3 |  | 0 |  | no | 1108.59 | 252.77 | 769.70 | 498.06 |
| DCVGPEVENACANPAAGTVILLENLR | 7499 | 28.97 | 3 | 2781.3441 | 3 |  | 0 |  | yes | 7716.79 | 1206.55 | 5981.49 | 2330.47 |
| GCITIIGGGDTATCCAK | 6556 | 58.39 | 3 | 1753.7793 | 2 |  | 0 |  | no | 1523.45 | 957.64 | 1085.79 | 846.55 |
| ITLPVDFVTADK | 5526 | 48.13 | 3 | 1317.7171 | 2 |  | 0 |  | no | 1972.68 | 1642.22 | 1722.36 | 1162.72 |
| VLPGVDALSNV | 2596 | 57.70 | 3 | 1082.5968 | 2 |  | 0 |  | yes | 2858.30 | 3790.10 | 2560.29 | 2742.16 |
| YSLEPVAAELK | 2449 | 48.64 | 3 | 1218.6491 | 2 |  | 0 |  | yes | 2147.01 | 3690.80 | 2854.51 | 2077.09 |

  

| Tags | |
| --- | --- |

  

##

## Q61400

CEAMA\_MOUSE
Carcinoembryonic antigen-related cell adhesion molecule 10 OS=Mus musculus
GN=Ceacam10 PE=1 SV=1  
5 peptides

  

| Sequence | Peptide Ion | Score | Hits | Mass | Charge | Tags | Conflicts | Modifications | In quantitation | Average Normalised Abundances | | | |
| --- | --- | --- | --- | --- | --- | --- | --- | --- | --- | --- | --- | --- | --- |
|  |  |  |  |  |  |  |  |  |  | 2m2f | 4m2f | 2m4f | 4m4f |
| AIYWYR | 111 | 26.69 | 3 | 870.4386 | 2 |  | 0 |  | no | 5.71e+004 | 6.09e+004 | 3.82e+004 | 5.86e+004 |
| EIIYNNGSLFFQGVTK | 26475 | --- | --- | 1828.9356 | 3 |  | 0 |  | no | 42.44 | 56.43 | 119.09 | 97.24 |
| EIIYNNGSLFFQGVTK | 11139 | 52.00 | 2 | 1828.9359 | 2 |  | 0 |  | no | 702.54 | 523.95 | 841.10 | 483.50 |
| FVTSINR | 57 | 35.93 | 3 | 835.4551 | 2 |  | 0 |  | yes | 1.01e+005 | 1.73e+005 | 1.11e+005 | 1.55e+005 |
| IILGPAHSDR | 113 | 52.51 | 3 | 1077.5931 | 3 |  | 0 |  | yes | 3.71e+004 | 4.39e+004 | 3.43e+004 | 4.02e+004 |
| IILGPAHSDR | 241 | 66.84 | 3 | 1077.5925 | 2 |  | 0 |  | yes | 2.65e+004 | 3.05e+004 | 2.40e+004 | 2.74e+004 |
| VFYWYK | 77 | 37.52 | 3 | 904.4482 | 2 |  | 0 |  | yes | 9.26e+004 | 1.94e+005 | 1.16e+005 | 2.10e+005 |

  

| Tags | |
| --- | --- |

  

##

## P20152

VIME\_MOUSE
Vimentin OS=Mus musculus GN=Vim PE=1 SV=3  
5 peptides

  

| Sequence | Peptide Ion | Score | Hits | Mass | Charge | Tags | Conflicts | Modifications | In quantitation | Average Normalised Abundances | | | |
| --- | --- | --- | --- | --- | --- | --- | --- | --- | --- | --- | --- | --- | --- |
|  |  |  |  |  |  |  |  |  |  | 2m2f | 4m2f | 2m4f | 4m4f |
| EEAESTLQSFR | 15140 | 34.97 | 2 | 1295.5996 | 2 |  | 0 |  | yes | 351.00 | 232.40 | 258.67 | 319.22 |
| EYQDLLNVK | 2583 | 55.04 | 3 | 1120.5788 | 2 |  | 1 |  | yes | 1847.05 | 960.56 | 1558.44 | 1756.13 |
| ILLAELEQLK | 5430 | 66.48 | 3 | 1168.7064 | 2 |  | 0 |  | yes | 893.08 | 323.22 | 792.36 | 717.87 |
| LLEGEESR | 1786 | 53.36 | 3 | 931.4603 | 2 |  | 2 |  | no | 2674.46 | 1761.54 | 2395.89 | 2386.46 |
| VELQELNDR | 1956 | 61.13 | 3 | 1114.5606 | 2 |  | 1 |  | no | 2040.94 | 703.53 | 1978.04 | 1167.96 |

  

| Tags | |
| --- | --- |

  

##

## P17742

PPIA\_MOUSE
Peptidyl-prolyl cis-trans isomerase A OS=Mus musculus GN=Ppia PE=1
SV=2  
5 peptides

  

| Sequence | Peptide Ion | Score | Hits | Mass | Charge | Tags | Conflicts | Modifications | In quantitation | Average Normalised Abundances | | | |
| --- | --- | --- | --- | --- | --- | --- | --- | --- | --- | --- | --- | --- | --- |
|  |  |  |  |  |  |  |  |  |  | 2m2f | 4m2f | 2m4f | 4m4f |
| EGMNIVEAMER | 2411 | 54.13 | 3 | 1277.5766 | 2 |  | 0 |  | yes | 2976.78 | 4138.35 | 2792.90 | 3605.28 |
| FEDENFILK | 848 | 60.73 | 3 | 1153.5650 | 2 |  | 0 |  | yes | 8313.82 | 1.04e+004 | 7094.71 | 1.06e+004 |
| KITISDCGQL | 2906 | 60.17 | 3 | 1133.5737 | 2 |  | 0 |  | no | 1856.63 | 2100.51 | 1674.01 | 1883.64 |
| TEWLDGK | 2285 | 35.69 | 3 | 847.4075 | 2 |  | 0 |  | no | 1708.38 | 1301.33 | 1283.50 | 1268.35 |
| VSFELFADK | 953 | 59.32 | 3 | 1054.5324 | 2 |  | 0 |  | yes | 6995.84 | 7834.57 | 7521.48 | 6760.70 |

  

| Tags | |
| --- | --- |

  

##

## Q8BZH1

TGM4\_MOUSE
Protein-glutamine gamma-glutamyltransferase 4 OS=Mus musculus GN=Tgm4 PE=1
SV=2  
5 peptides

  

| Sequence | Peptide Ion | Score | Hits | Mass | Charge | Tags | Conflicts | Modifications | In quantitation | Average Normalised Abundances | | | |
| --- | --- | --- | --- | --- | --- | --- | --- | --- | --- | --- | --- | --- | --- |
|  |  |  |  |  |  |  |  |  |  | 2m2f | 4m2f | 2m4f | 4m4f |
| FIWLVK | 12624 | 31.81 | 3 | 804.4896 | 2 |  | 0 |  | no | 121.17 | 28.74 | 28.73 | 113.28 |
| FSVESLGLANMK | 14180 | 65.14 | 3 | 1294.6550 | 2 |  | 0 |  | yes | 594.22 | 408.02 | 322.73 | 559.93 |
| FTNTLPIPLTNIK | 12557 | 38.78 | 3 | 1470.8445 | 2 |  | 0 |  | yes | 606.37 | 159.62 | 162.62 | 651.15 |
| GQIFTLK | 36430 | 28.37 | 2 | 805.4695 | 2 |  | 0 |  | no | 42.57 | 2.36 | 7.35 | 17.86 |
| NILIAVETASLGK | 13247 | 104.44 | 1 | 1327.7711 | 2 |  | 0 |  | yes | 261.64 | 63.49 | 35.49 | 129.51 |

  

| Tags | |
| --- | --- |

  

##

## P62983

RS27A\_MOUSE
Ubiquitin-40S ribosomal protein S27a OS=Mus musculus GN=Rps27a PE=1
SV=2  
4 peptides

  

| Sequence | Peptide Ion | Score | Hits | Mass | Charge | Tags | Conflicts | Modifications | In quantitation | Average Normalised Abundances | | | |
| --- | --- | --- | --- | --- | --- | --- | --- | --- | --- | --- | --- | --- | --- |
|  |  |  |  |  |  |  |  |  |  | 2m2f | 4m2f | 2m4f | 4m4f |
| ESTLHLVLR | 1798 | 59.54 | 3 | 1066.6091 | 2 |  | 0 |  | yes | 2226.73 | 2050.98 | 1660.58 | 2548.46 |
| ESTLHLVLR | 2154 | --- | --- | 1066.6137 | 3 |  | 0 |  | yes | 913.52 | 667.42 | 728.83 | 854.42 |
| MQIFVK | 1187 | 40.27 | 3 | 764.4254 | 2 |  | 0 |  | no | 2136.50 | 2400.62 | 1882.85 | 2194.53 |
| TITLEVEPSDTIENVK | 2796 | 58.83 | 3 | 1786.9256 | 3 |  | 0 |  | yes | 3349.56 | 3040.44 | 3714.42 | 2323.02 |
| TITLEVEPSDTIENVK | 591 | 64.33 | 3 | 1786.9201 | 2 |  | 0 |  | yes | 3.49e+004 | 3.72e+004 | 3.59e+004 | 2.64e+004 |
| TLSDYNIQK | 818 | 45.22 | 3 | 1080.5414 | 2 |  | 0 |  | yes | 8183.16 | 8924.20 | 8174.80 | 9026.36 |

  

| Tags | |
| --- | --- |

  

##

## Q8BFZ3

ACTBL\_MOUSE
Beta-actin-like protein 2 OS=Mus musculus GN=Actbl2 PE=1 SV=1  
5
peptides

  

| Sequence | Peptide Ion | Score | Hits | Mass | Charge | Tags | Conflicts | Modifications | In quantitation | Average Normalised Abundances | | | |
| --- | --- | --- | --- | --- | --- | --- | --- | --- | --- | --- | --- | --- | --- |
|  |  |  |  |  |  |  |  |  |  | 2m2f | 4m2f | 2m4f | 4m4f |
| DLTDYLMK | 257 | 45.15 | 3 | 997.4796 | 2 |  | 3 |  | no | 2.12e+004 | 1.57e+004 | 1.93e+004 | 2.56e+004 |
| IIAPPER | 164 | 32.59 | 3 | 794.4648 | 2 |  | 3 |  | no | 2.79e+004 | 1.34e+004 | 2.56e+004 | 2.12e+004 |
| RGILTLK | 1096 | 27.19 | 3 | 799.5279 | 2 |  | 3 |  | no | 1561.95 | 1158.78 | 2123.78 | 1617.99 |
| SYELPDGQVITIGNER | 6364 | 46.14 | 2 | 1789.8846 | 3 |  | 3 |  | no | 402.77 | 125.55 | 125.84 | 177.85 |
| SYELPDGQVITIGNER | 251 | 60.47 | 3 | 1789.8851 | 2 |  | 3 |  | no | 8.21e+004 | 4.91e+004 | 8.77e+004 | 7.29e+004 |
| SYELPDGQVITIGNER | 653 | 64.66 | 3 | 1789.8837 | 3 |  | 3 |  | no | 1.45e+004 | 8872.48 | 1.62e+004 | 1.32e+004 |
| VAPDEHPILLTEAPLNPK | 555 | 31.43 | 3 | 1953.0561 | 3 |  | 1 |  | no | 2.56e+004 | 7377.57 | 2.24e+004 | 1.38e+004 |

  

| Tags | |
| --- | --- |

  

##

## P62259

1433E\_MOUSE
14-3-3 protein epsilon OS=Mus musculus GN=Ywhae PE=1 SV=1  
5
peptides

  

| Sequence | Peptide Ion | Score | Hits | Mass | Charge | Tags | Conflicts | Modifications | In quantitation | Average Normalised Abundances | | | |
| --- | --- | --- | --- | --- | --- | --- | --- | --- | --- | --- | --- | --- | --- |
|  |  |  |  |  |  |  |  |  |  | 2m2f | 4m2f | 2m4f | 4m4f |
| AAFDDAIAELDTLSEESYK | 7049 | --- | --- | 2086.9607 | 2 |  | 0 |  | yes | 4958.40 | 1646.94 | 4019.11 | 2637.76 |
| AAFDDAIAELDTLSEESYK | 3712 | 38.72 | 3 | 2086.9574 | 3 |  | 0 |  | yes | 6977.62 | 2715.93 | 6298.66 | 3789.50 |
| DSTLIMQLLR | 2121 | 76.05 | 3 | 1188.6524 | 2 |  | 3 |  | yes | 5173.61 | 3874.97 | 5291.93 | 4679.16 |
| LICCDILDVLDK | 3161 | 74.26 | 3 | 1475.7360 | 2 |  | 0 |  | yes | 6179.55 | 3282.61 | 5621.01 | 4956.78 |
| NLLSVAYK | 1420 | 43.60 | 3 | 906.5168 | 2 |  | 3 |  | no | 1821.69 | 1728.75 | 1828.48 | 2020.75 |
| VAGMDVELTVEER | 8318 | 28.60 | 1 | 1446.7036 | 2 |  | 0 |  | no | 1101.22 | 887.15 | 866.90 | 999.98 |

  

| Tags | |
| --- | --- |

  

##

## P68254

1433T\_MOUSE
14-3-3 protein theta OS=Mus musculus GN=Ywhaq PE=1 SV=1  
5
peptides

  

| Sequence | Peptide Ion | Score | Hits | Mass | Charge | Tags | Conflicts | Modifications | In quantitation | Average Normalised Abundances | | | |
| --- | --- | --- | --- | --- | --- | --- | --- | --- | --- | --- | --- | --- | --- |
|  |  |  |  |  |  |  |  |  |  | 2m2f | 4m2f | 2m4f | 4m4f |
| AVTEQGAELSNEER | 8521 | 72.58 | 2 | 1531.7111 | 2 |  | 0 |  | yes | 953.22 | 511.67 | 593.33 | 1142.04 |
| DSTLIMQLLR | 2121 | 76.05 | 3 | 1188.6524 | 2 |  | 3 |  | yes | 5173.61 | 3874.97 | 5291.93 | 4679.16 |
| EMQPTHPIR | 6444 | 25.85 | 2 | 1107.5546 | 2 |  | 2 |  | no | 447.12 | 686.98 | 457.79 | 791.37 |
| EMQPTHPIR | 7547 | --- | --- | 1107.5558 | 3 |  | 2 |  | no | 89.40 | 271.70 | 151.15 | 342.12 |
| NLLSVAYK | 1420 | 43.60 | 3 | 906.5168 | 2 |  | 3 |  | no | 1821.69 | 1728.75 | 1828.48 | 2020.75 |
| VISSIEQK | 3333 | 42.93 | 3 | 902.5062 | 2 |  | 1 |  | yes | 1123.73 | 851.37 | 1004.75 | 878.00 |

  

| Tags | |
| --- | --- |

  

##

## P14211

CALR\_MOUSE
Calreticulin OS=Mus musculus GN=Calr PE=1 SV=1  
4 peptides

  

| Sequence | Peptide Ion | Score | Hits | Mass | Charge | Tags | Conflicts | Modifications | In quantitation | Average Normalised Abundances | | | |
| --- | --- | --- | --- | --- | --- | --- | --- | --- | --- | --- | --- | --- | --- |
|  |  |  |  |  |  |  |  |  |  | 2m2f | 4m2f | 2m4f | 4m4f |
| DMHGDSEYNIMFGPDICGPGTK | 7779 | 36.66 | 3 | 2440.0154 | 3 |  | 0 |  | no | 1508.63 | 314.53 | 1113.31 | 709.22 |
| EQFLDGDAWTNR | 1304 | 75.70 | 3 | 1450.6513 | 2 |  | 0 |  | yes | 1.14e+004 | 1.33e+004 | 1.04e+004 | 1.23e+004 |
| GQTLVVQFTVK | 3026 | 58.17 | 3 | 1218.6963 | 2 |  | 0 |  | yes | 1879.38 | 1206.07 | 1539.24 | 852.93 |
| HEQNIDCGGGYVK | 6069 | 50.98 | 3 | 1475.6444 | 2 |  | 0 |  | yes | 1289.52 | 1353.03 | 943.77 | 2130.22 |
| HEQNIDCGGGYVK | 2572 | 39.12 | 3 | 1475.6461 | 3 |  | 0 |  | yes | 2313.18 | 2877.69 | 2098.13 | 3648.96 |

  

| Tags | |
| --- | --- |

  

##

## P68368

TBA4A\_MOUSE
Tubulin alpha-4A chain OS=Mus musculus GN=Tuba4a PE=1 SV=1  
5
peptides

  

| Sequence | Peptide Ion | Score | Hits | Mass | Charge | Tags | Conflicts | Modifications | In quantitation | Average Normalised Abundances | | | |
| --- | --- | --- | --- | --- | --- | --- | --- | --- | --- | --- | --- | --- | --- |
|  |  |  |  |  |  |  |  |  |  | 2m2f | 4m2f | 2m4f | 4m4f |
| AVCMLSNTTAIAEAWAR | 10219 | 30.97 | 1 | 1863.8979 | 3 |  | 2 |  | no | 489.45 | 154.15 | 459.53 | 174.45 |
| AVFVDLEPTVIDEIR | 9780 | 50.56 | 3 | 1714.9135 | 2 |  | 0 |  | yes | 1389.73 | 470.95 | 1312.43 | 601.31 |
| AVFVDLEPTVIDEIR | 15855 | --- | --- | 1714.9123 | 3 |  | 0 |  | yes | 263.70 | 112.16 | 227.22 | 124.69 |
| EDAANNYAR | 5692 | 63.88 | 3 | 1022.4410 | 2 |  | 2 |  | no | 809.45 | 505.77 | 667.00 | 489.55 |
| SIQFVDWCPTGFK | 4066 | 58.42 | 3 | 1583.7403 | 2 |  | 1 |  | no | 3563.58 | 1848.50 | 2984.33 | 2759.19 |
| VGINYQPPTVVPGGDLAK | 3505 | 55.61 | 3 | 1823.9780 | 2 |  | 2 |  | no | 5432.91 | 3727.29 | 4970.77 | 4486.65 |
| VGINYQPPTVVPGGDLAK | 6184 | --- | --- | 1823.9775 | 3 |  | 2 |  | no | 1272.27 | 776.10 | 1234.45 | 968.21 |

  

| Tags | |
| --- | --- |

  

##

## Q6ZWN5

RS9\_MOUSE
40S ribosomal protein S9 OS=Mus musculus GN=Rps9 PE=2 SV=3  
6
peptides

  

| Sequence | Peptide Ion | Score | Hits | Mass | Charge | Tags | Conflicts | Modifications | In quantitation | Average Normalised Abundances | | | |
| --- | --- | --- | --- | --- | --- | --- | --- | --- | --- | --- | --- | --- | --- |
|  |  |  |  |  |  |  |  |  |  | 2m2f | 4m2f | 2m4f | 4m4f |
| IEDFLER | 2983 | 44.11 | 3 | 920.4600 | 2 |  | 0 |  | no | 1009.13 | 862.06 | 897.08 | 824.29 |
| IGVLDEGK | 3669 | 39.50 | 3 | 829.4545 | 2 |  | 0 |  | no | 720.87 | 599.37 | 661.85 | 564.22 |
| LDYILGLK | 3435 | 52.45 | 3 | 933.5527 | 2 |  | 0 |  | yes | 1033.10 | 970.69 | 1033.49 | 1019.34 |
| LFEGNALLR | 3136 | 62.89 | 3 | 1031.5757 | 2 |  | 0 |  | yes | 1327.13 | 1438.74 | 1304.64 | 1631.29 |
| LIGEYGLR | 3510 | 34.82 | 3 | 919.5125 | 2 |  | 0 |  | yes | 1001.36 | 859.38 | 864.38 | 905.20 |
| QVVNIPSFIVR | 12857 | 25.64 | 3 | 1270.7336 | 2 |  | 0 |  | no | 242.15 | 135.00 | 214.57 | 235.96 |

  

| Tags | |
| --- | --- |

  

##

## P84084

ARF5\_MOUSE
ADP-ribosylation factor 5 OS=Mus musculus GN=Arf5 PE=2 SV=2  
5
peptides

  

| Sequence | Peptide Ion | Score | Hits | Mass | Charge | Tags | Conflicts | Modifications | In quantitation | Average Normalised Abundances | | | |
| --- | --- | --- | --- | --- | --- | --- | --- | --- | --- | --- | --- | --- | --- |
|  |  |  |  |  |  |  |  |  |  | 2m2f | 4m2f | 2m4f | 4m4f |
| DAVLLVFANK | 2810 | 63.96 | 3 | 1088.6223 | 2 |  | 1 |  | no | 1536.42 | 1734.65 | 2197.54 | 1683.10 |
| HYFQNTQGLIFVVDSNDR | 9786 | 33.31 | 2 | 2152.0324 | 3 |  | 1 |  | no | 513.36 | 170.07 | 623.46 | 228.03 |
| ILMVGLDAAGK | 2146 | 59.52 | 3 | 1086.6100 | 2 |  | 1 |  | yes | 2364.09 | 2405.79 | 2932.69 | 2010.72 |
| LGEIVTTIPTIGFNVETVEYK | 3105 | 49.10 | 3 | 2322.2352 | 3 |  | 1 |  | yes | 7810.86 | 2676.54 | 6734.56 | 1989.72 |
| LGEIVTTIPTIGFNVETVEYK | 9817 | --- | --- | 2322.2375 | 2 |  | 1 |  | yes | 2476.51 | 820.06 | 2136.25 | 996.88 |
| NICFTVWDVGGQDK | 8886 | 50.48 | 3 | 1637.7510 | 2 |  | 0 |  | yes | 1069.39 | 476.15 | 1077.84 | 502.38 |

  

| Tags | |
| --- | --- |

  

##

## P62242

RS8\_MOUSE
40S ribosomal protein S8 OS=Mus musculus GN=Rps8 PE=1 SV=2  
4
peptides

  

| Sequence | Peptide Ion | Score | Hits | Mass | Charge | Tags | Conflicts | Modifications | In quantitation | Average Normalised Abundances | | | |
| --- | --- | --- | --- | --- | --- | --- | --- | --- | --- | --- | --- | --- | --- |
|  |  |  |  |  |  |  |  |  |  | 2m2f | 4m2f | 2m4f | 4m4f |
| IIDVVYNASNNELVR | 4091 | 59.50 | 3 | 1717.8881 | 2 |  | 0 |  | yes | 2046.81 | 1584.17 | 1629.48 | 1476.97 |
| ISSLLEEQFQQGK | 3266 | 81.33 | 3 | 1505.7721 | 2 |  | 0 |  | yes | 2935.71 | 2835.25 | 3054.93 | 2356.33 |
| LDVGNFSWGSECCTR | 6473 | 36.98 | 3 | 1786.7410 | 2 |  | 0 |  | yes | 1683.98 | 1609.42 | 1591.64 | 1528.83 |
| NCIVLIDSTPYR | 5566 | 77.65 | 3 | 1449.7282 | 2 |  | 0 |  | no | 1237.44 | 909.28 | 1492.17 | 1166.81 |

  

| Tags | |
| --- | --- |

  

##

## P09036

ISK3\_MOUSE
Serine protease inhibitor Kazal-type 3 OS=Mus musculus GN=Spink3 PE=1
SV=1  
4 peptides

  

| Sequence | Peptide Ion | Score | Hits | Mass | Charge | Tags | Conflicts | Modifications | In quantitation | Average Normalised Abundances | | | |
| --- | --- | --- | --- | --- | --- | --- | --- | --- | --- | --- | --- | --- | --- |
|  |  |  |  |  |  |  |  |  |  | 2m2f | 4m2f | 2m4f | 4m4f |
| EASCHDAVAGCPR | 980 | 64.20 | 3 | 1428.5871 | 2 |  | 0 |  | yes | 1.76e+004 | 1.83e+004 | 1.47e+004 | 1.93e+004 |
| EASCHDAVAGCPR | 119 | 53.33 | 3 | 1428.5869 | 3 |  | 0 |  | yes | 9.58e+004 | 1.15e+005 | 9.41e+004 | 1.09e+005 |
| IEPVLIR | 76 | 40.40 | 3 | 838.5279 | 2 |  | 0 |  | yes | 6.54e+004 | 1.32e+005 | 6.78e+004 | 1.43e+005 |
| IYDPVCGTDGITYANECVLCFENR | 8299 | --- | --- | 2865.2391 | 4 |  | 0 |  | yes | 1192.74 | 682.68 | 1293.75 | 500.80 |
| IYDPVCGTDGITYANECVLCFENR | 5029 | --- | --- | 2865.2462 | 2 |  | 0 |  | yes | 1.01e+004 | 7241.48 | 8444.26 | 6952.19 |
| IYDPVCGTDGITYANECVLCFENR | 274 | 58.67 | 3 | 2865.2411 | 3 |  | 0 |  | yes | 2.03e+005 | 1.52e+005 | 1.80e+005 | 9.73e+004 |
| RIEPVLIR | 343 | 36.03 | 3 | 994.6284 | 2 |  | 0 |  | no | 1.32e+004 | 1.64e+004 | 1.25e+004 | 1.87e+004 |

  

| Tags | |
| --- | --- |

  

##

## P17182

ENOA\_MOUSE
Alpha-enolase OS=Mus musculus GN=Eno1 PE=1 SV=3  
4 peptides

  

| Sequence | Peptide Ion | Score | Hits | Mass | Charge | Tags | Conflicts | Modifications | In quantitation | Average Normalised Abundances | | | |
| --- | --- | --- | --- | --- | --- | --- | --- | --- | --- | --- | --- | --- | --- |
|  |  |  |  |  |  |  |  |  |  | 2m2f | 4m2f | 2m4f | 4m4f |
| AAVPSGASTGIYEALELR | 5758 | 61.82 | 3 | 1803.9368 | 2 |  | 0 |  | yes | 2718.87 | 1662.90 | 2493.79 | 780.54 |
| FTASAGIQVVGDDLTVTNPK | 6953 | 34.23 | 3 | 2032.0370 | 3 |  | 0 |  | no | 1859.95 | 754.97 | 1393.55 | 442.21 |
| VNQIGSVTESLQACK | 5581 | 89.43 | 3 | 1632.8101 | 2 |  | 0 |  | yes | 1925.19 | 2045.95 | 1853.96 | 1200.06 |
| YITPDQLADLYK | 3035 | 67.02 | 3 | 1438.7311 | 2 |  | 0 |  | yes | 3604.25 | 3633.67 | 3804.46 | 2708.01 |

  

| Tags | |
| --- | --- |

  

##

## Q8BG05

ROA3\_MOUSE
Heterogeneous nuclear ribonucleoprotein A3 OS=Mus musculus GN=Hnrnpa3 PE=1
SV=1  
4 peptides

  

| Sequence | Peptide Ion | Score | Hits | Mass | Charge | Tags | Conflicts | Modifications | In quantitation | Average Normalised Abundances | | | |
| --- | --- | --- | --- | --- | --- | --- | --- | --- | --- | --- | --- | --- | --- |
|  |  |  |  |  |  |  |  |  |  | 2m2f | 4m2f | 2m4f | 4m4f |
| EDTEEYNLR | 5789 | 41.85 | 3 | 1167.5037 | 2 |  | 0 |  | yes | 931.54 | 793.03 | 812.84 | 925.06 |
| IETIEVMEDR | 4576 | 73.06 | 3 | 1233.5978 | 2 |  | 0 |  | yes | 1988.25 | 3002.33 | 1916.45 | 4348.97 |
| LFIGGLSFETTDDSLR | 6840 | 85.94 | 3 | 1769.8834 | 2 |  | 0 |  | yes | 1663.09 | 643.12 | 2157.03 | 548.05 |
| WGTLTDCVVMR | 5962 | 45.06 | 2 | 1336.6263 | 2 |  | 0 |  | no | 757.47 | 689.39 | 947.84 | 695.51 |

  

| Tags | |
| --- | --- |

  

##

## P99027

RLA2\_MOUSE
60S acidic ribosomal protein P2 OS=Mus musculus GN=Rplp2 PE=1 SV=3  
4
peptides

  

| Sequence | Peptide Ion | Score | Hits | Mass | Charge | Tags | Conflicts | Modifications | In quantitation | Average Normalised Abundances | | | |
| --- | --- | --- | --- | --- | --- | --- | --- | --- | --- | --- | --- | --- | --- |
|  |  |  |  |  |  |  |  |  |  | 2m2f | 4m2f | 2m4f | 4m4f |
| ILDSVGIEADDDRLNK | 22057 | --- | --- | 1771.8937 | 2 |  | 0 |  | no | 283.63 | 62.59 | 74.85 | 85.35 |
| ILDSVGIEADDDRLNK | 2752 | 39.81 | 3 | 1771.8949 | 3 |  | 0 |  | no | 2425.32 | 2007.23 | 2437.76 | 2416.73 |
| LASVPAGGAVAVSAAPGSAAPAAGSAPAAAEEK | 2726 | 38.14 | 3 | 2773.4252 | 3 |  | 0 |  | yes | 7876.00 | 2966.77 | 6594.49 | 4568.15 |
| NIEDVIAQGVGK | 1448 | 88.00 | 3 | 1241.6736 | 2 |  | 0 |  | yes | 5680.68 | 6528.90 | 5661.62 | 6783.03 |
| YVASYLLAALGGNSSPSAK | 4893 | 34.59 | 3 | 1867.9680 | 2 |  | 0 |  | yes | 2634.62 | 1652.89 | 3254.94 | 1951.38 |
| YVASYLLAALGGNSSPSAK | 4461 | 42.78 | 3 | 1867.9676 | 3 |  | 0 |  | yes | 1686.02 | 1124.95 | 2069.25 | 1278.29 |

  

| Tags | |
| --- | --- |

  

##

## P51410

RL9\_MOUSE
60S ribosomal protein L9 OS=Mus musculus GN=Rpl9 PE=2 SV=2  
5
peptides

  

| Sequence | Peptide Ion | Score | Hits | Mass | Charge | Tags | Conflicts | Modifications | In quantitation | Average Normalised Abundances | | | |
| --- | --- | --- | --- | --- | --- | --- | --- | --- | --- | --- | --- | --- | --- |
|  |  |  |  |  |  |  |  |  |  | 2m2f | 4m2f | 2m4f | 4m4f |
| DELILEGNDIELVSNSAALIQQATTVK | 8404 | 44.80 | 3 | 2883.5078 | 3 |  | 0 |  | yes | 5774.22 | 1768.46 | 3724.52 | 1629.69 |
| DFNHINVELSLLGK | 11328 | 34.89 | 3 | 1597.8455 | 3 |  | 0 |  | no | 215.70 | 77.25 | 159.86 | 69.98 |
| FLDGIYVSEK | 3269 | 57.44 | 3 | 1169.5945 | 2 |  | 0 |  | no | 1261.20 | 1421.14 | 1396.22 | 1295.24 |
| TGVACSVSQAQK | 4677 | 67.18 | 3 | 1234.5968 | 2 |  | 0 |  | yes | 1811.37 | 2416.65 | 2187.12 | 2277.47 |
| TILSNQTVDIPENVEITLK | 5747 | 28.95 | 3 | 2126.1459 | 3 |  | 0 |  | yes | 2077.34 | 1153.12 | 2104.75 | 727.00 |

  

| Tags | |
| --- | --- |

  

##

## P14206

RSSA\_MOUSE
40S ribosomal protein SA OS=Mus musculus GN=Rpsa PE=1 SV=4  
5
peptides

  

| Sequence | Peptide Ion | Score | Hits | Mass | Charge | Tags | Conflicts | Modifications | In quantitation | Average Normalised Abundances | | | |
| --- | --- | --- | --- | --- | --- | --- | --- | --- | --- | --- | --- | --- | --- |
|  |  |  |  |  |  |  |  |  |  | 2m2f | 4m2f | 2m4f | 4m4f |
| AIVAIENPADVSVISSR | 7620 | --- | --- | 1739.9414 | 3 |  | 0 |  | yes | 619.87 | 396.66 | 754.28 | 633.77 |
| AIVAIENPADVSVISSR | 2753 | 45.61 | 3 | 1739.9365 | 2 |  | 0 |  | yes | 4245.19 | 3454.26 | 5031.08 | 4007.66 |
| DPEEIEKEEQAAAEK | 6428 | 28.99 | 3 | 1714.8024 | 3 |  | 0 |  | no | 1228.22 | 820.27 | 1217.12 | 985.16 |
| FAAATGATPIAGR | 2681 | 58.36 | 3 | 1202.6407 | 2 |  | 0 |  | yes | 2270.56 | 1785.78 | 2074.63 | 2237.67 |
| FTPGTFTNQIQAAFR | 13176 | --- | --- | 1697.8507 | 3 |  | 0 |  | yes | 341.10 | 94.96 | 264.44 | 216.14 |
| FTPGTFTNQIQAAFR | 6424 | 46.88 | 3 | 1697.8531 | 2 |  | 0 |  | yes | 2235.13 | 914.77 | 1738.81 | 1707.93 |
| SDGIYIINLK | 4111 | 52.61 | 3 | 1134.6282 | 2 |  | 0 |  | no | 1175.68 | 1279.22 | 1112.00 | 1187.19 |

  

| Tags | |
| --- | --- |

  

##

## P25444

RS2\_MOUSE
40S ribosomal protein S2 OS=Mus musculus GN=Rps2 PE=1 SV=3  
5
peptides

  

| Sequence | Peptide Ion | Score | Hits | Mass | Charge | Tags | Conflicts | Modifications | In quantitation | Average Normalised Abundances | | | |
| --- | --- | --- | --- | --- | --- | --- | --- | --- | --- | --- | --- | --- | --- |
|  |  |  |  |  |  |  |  |  |  | 2m2f | 4m2f | 2m4f | 4m4f |
| AEDKEWIPVTK | 3919 | 31.52 | 3 | 1314.6854 | 3 |  | 0 |  | no | 1250.00 | 1064.03 | 1103.02 | 1407.56 |
| GCTATLGNFAK | 3848 | 39.85 | 3 | 1138.5336 | 2 |  | 0 |  | yes | 1624.05 | 1411.95 | 1559.27 | 1339.02 |
| SLEEIYLFSLPIK | 3976 | 74.67 | 3 | 1550.8594 | 2 |  | 0 |  | yes | 6239.38 | 2384.74 | 6233.95 | 4185.60 |
| SLEEIYLFSLPIK | 40409 | --- | --- | 1550.8572 | 3 |  | 0 |  | yes | 78.99 | 22.99 | 106.96 | 51.82 |
| SPYQEFTDHLVK | 6136 | 34.72 | 3 | 1462.7088 | 3 |  | 0 |  | no | 678.21 | 261.78 | 480.54 | 533.36 |
| TYSYLTPDLWK | 3067 | 44.25 | 3 | 1385.6857 | 2 |  | 0 |  | yes | 3238.77 | 3071.16 | 3551.60 | 2785.36 |

  

| Tags | |
| --- | --- |

  

##

## P62754

RS6\_MOUSE
40S ribosomal protein S6 OS=Mus musculus GN=Rps6 PE=1 SV=1  
3
peptides

  

| Sequence | Peptide Ion | Score | Hits | Mass | Charge | Tags | Conflicts | Modifications | In quantitation | Average Normalised Abundances | | | |
| --- | --- | --- | --- | --- | --- | --- | --- | --- | --- | --- | --- | --- | --- |
|  |  |  |  |  |  |  |  |  |  | 2m2f | 4m2f | 2m4f | 4m4f |
| DIPGLTDTTVPR | 4024 | 49.24 | 3 | 1283.6715 | 2 |  | 0 |  | yes | 2152.33 | 1236.68 | 1617.57 | 1710.95 |
| GCIVDANLSVLNLVIVK | 16387 | 53.53 | 1 | 1826.0334 | 2 |  | 0 |  | yes | 521.92 | 336.24 | 642.82 | 155.80 |
| MATEVAADALGEEWK | 4759 | 87.08 | 3 | 1619.7501 | 2 |  | 0 |  | yes | 2102.49 | 2101.99 | 2436.11 | 1876.31 |
| MATEVAADALGEEWK | 7146 | 31.93 | 3 | 1619.7486 | 3 |  | 0 |  | yes | 437.19 | 420.53 | 657.39 | 489.59 |

  

| Tags | |
| --- | --- |

  

##

## P68040

GBLP\_MOUSE
Guanine nucleotide-binding protein subunit beta-2-like 1 OS=Mus musculus
GN=Gnb2l1 PE=1 SV=3  
5 peptides

  

| Sequence | Peptide Ion | Score | Hits | Mass | Charge | Tags | Conflicts | Modifications | In quantitation | Average Normalised Abundances | | | |
| --- | --- | --- | --- | --- | --- | --- | --- | --- | --- | --- | --- | --- | --- |
|  |  |  |  |  |  |  |  |  |  | 2m2f | 4m2f | 2m4f | 4m4f |
| DGQAMLWDLNEGK | 7019 | 44.88 | 2 | 1475.6715 | 2 |  | 0 |  | yes | 1183.27 | 921.62 | 1174.58 | 885.36 |
| HLYTLDGGDIINALCFSPNR | 13011 | 25.09 | 2 | 2275.1032 | 3 |  | 0 |  | no | 631.56 | 162.67 | 430.75 | 242.45 |
| LWDLTTGTTTR | 2516 | 55.59 | 3 | 1263.6451 | 2 |  | 0 |  | yes | 3156.17 | 4393.23 | 3115.09 | 4150.34 |
| LWNTLGVCK | 4981 | 51.66 | 3 | 1089.5637 | 2 |  | 0 |  | no | 879.17 | 670.55 | 700.34 | 841.81 |
| YWLCAATGPSIK | 3793 | 43.17 | 3 | 1365.6740 | 2 |  | 0 |  | yes | 1884.79 | 1525.01 | 1827.09 | 2034.04 |

  

| Tags | |
| --- | --- |

  

##

## P62204

CALM\_MOUSE
Calmodulin OS=Mus musculus GN=Calm1 PE=1 SV=2  
4 peptides

  

| Sequence | Peptide Ion | Score | Hits | Mass | Charge | Tags | Conflicts | Modifications | In quantitation | Average Normalised Abundances | | | |
| --- | --- | --- | --- | --- | --- | --- | --- | --- | --- | --- | --- | --- | --- |
|  |  |  |  |  |  |  |  |  |  | 2m2f | 4m2f | 2m4f | 4m4f |
| DGNGYISAAELR | 3741 | 73.79 | 3 | 1264.6172 | 2 |  | 0 |  | yes | 3939.90 | 3217.61 | 2918.88 | 3093.30 |
| DTDSEEEIR | 3090 | 48.10 | 3 | 1092.4567 | 2 |  | 0 |  | yes | 3224.16 | 2733.16 | 2873.31 | 2436.85 |
| MKDTDSEEEIR | 47290 | 32.63 | 2 | 1351.5916 | 3 |  | 0 |  | no | 216.13 | 296.39 | 288.21 | 285.59 |
| VFDKDGNGYISAAELR | 4434 | 61.15 | 3 | 1753.8544 | 3 |  | 0 |  | yes | 1518.74 | 795.52 | 1556.03 | 1309.98 |

  

| Tags | |
| --- | --- |

  

##

## P05064

ALDOA\_MOUSE
Fructose-bisphosphate aldolase A OS=Mus musculus GN=Aldoa PE=1 SV=2  
4
peptides

  

| Sequence | Peptide Ion | Score | Hits | Mass | Charge | Tags | Conflicts | Modifications | In quantitation | Average Normalised Abundances | | | |
| --- | --- | --- | --- | --- | --- | --- | --- | --- | --- | --- | --- | --- | --- |
|  |  |  |  |  |  |  |  |  |  | 2m2f | 4m2f | 2m4f | 4m4f |
| ALANSLACQGK | 4817 | 48.10 | 3 | 1131.5705 | 2 |  | 0 |  | yes | 936.17 | 753.04 | 815.02 | 783.63 |
| ALQASALK | 5144 | 40.41 | 3 | 800.4754 | 2 |  | 1 |  | no | 623.33 | 586.53 | 626.01 | 593.69 |
| GILAADESTGSIAK | 2562 | 71.01 | 3 | 1331.6865 | 2 |  | 0 |  | yes | 4686.77 | 4254.05 | 3206.95 | 3902.85 |
| LQSIGTENTEENR | 8096 | 52.45 | 1 | 1489.7001 | 2 |  | 0 |  | yes | 687.42 | 770.62 | 743.95 | 644.57 |

  

| Tags | |
| --- | --- |

  

##

## P47911

RL6\_MOUSE
60S ribosomal protein L6 OS=Mus musculus GN=Rpl6 PE=1 SV=3  
4
peptides

  

| Sequence | Peptide Ion | Score | Hits | Mass | Charge | Tags | Conflicts | Modifications | In quantitation | Average Normalised Abundances | | | |
| --- | --- | --- | --- | --- | --- | --- | --- | --- | --- | --- | --- | --- | --- |
|  |  |  |  |  |  |  |  |  |  | 2m2f | 4m2f | 2m4f | 4m4f |
| AVDLQILPK | 3509 | 66.33 | 3 | 995.6012 | 2 |  | 0 |  | yes | 1153.12 | 942.40 | 1203.09 | 1168.58 |
| AVPQLQGYLR | 3039 | 27.70 | 2 | 1143.6455 | 2 |  | 1 |  | no | 2138.39 | 2835.69 | 2200.81 | 3114.53 |
| HLTDAYFK | 4300 | 35.35 | 3 | 993.4915 | 2 |  | 0 |  | yes | 646.73 | 853.70 | 727.50 | 935.56 |
| SSITPGTVLIILTGR | 7755 | 44.73 | 3 | 1526.9022 | 2 |  | 0 |  | yes | 3358.30 | 2890.10 | 2778.88 | 1628.68 |
| SSITPGTVLIILTGR | 7616 | 33.85 | 3 | 1526.9019 | 3 |  | 0 |  | yes | 1646.05 | 467.27 | 1520.17 | 835.66 |

  

| Tags | |
| --- | --- |

  

##

## P09405

NUCL\_MOUSE
Nucleolin OS=Mus musculus GN=Ncl PE=1 SV=2  
4 peptides

  

| Sequence | Peptide Ion | Score | Hits | Mass | Charge | Tags | Conflicts | Modifications | In quantitation | Average Normalised Abundances | | | |
| --- | --- | --- | --- | --- | --- | --- | --- | --- | --- | --- | --- | --- | --- |
|  |  |  |  |  |  |  |  |  |  | 2m2f | 4m2f | 2m4f | 4m4f |
| FAISELFAK | 7850 | 52.30 | 3 | 1024.5583 | 2 |  | 0 |  | no | 444.46 | 319.09 | 447.96 | 344.68 |
| FGYVDFESAEDLEK | 10617 | 60.61 | 1 | 1647.7318 | 2 |  | 0 |  | yes | 571.41 | 336.52 | 624.22 | 222.66 |
| GFGFVDFNSEEDAK | 7155 | 25.79 | 3 | 1560.6755 | 2 |  | 0 |  | yes | 1445.71 | 3280.47 | 1725.97 | 3185.89 |
| TLVLSNLSYSATK | 7830 | 69.16 | 3 | 1395.7590 | 2 |  | 0 |  | yes | 669.48 | 783.78 | 646.23 | 574.35 |

  

| Tags | |
| --- | --- |

  

##

## P08228

SODC\_MOUSE
Superoxide dismutase [Cu-Zn] OS=Mus musculus GN=Sod1 PE=1 SV=2  
4
peptides

  

| Sequence | Peptide Ion | Score | Hits | Mass | Charge | Tags | Conflicts | Modifications | In quantitation | Average Normalised Abundances | | | |
| --- | --- | --- | --- | --- | --- | --- | --- | --- | --- | --- | --- | --- | --- |
|  |  |  |  |  |  |  |  |  |  | 2m2f | 4m2f | 2m4f | 4m4f |
| DGVANVSIEDR | 1365 | 77.72 | 3 | 1173.5623 | 2 |  | 0 |  | yes | 4089.86 | 7040.13 | 5054.05 | 5757.08 |
| GDGPVQGTIHFEQK | 1663 | 26.67 | 3 | 1511.7362 | 3 |  | 0 |  | no | 2023.35 | 2081.02 | 1775.08 | 2445.11 |
| HVGDLGNVTAGK | 1203 | 68.52 | 3 | 1166.6039 | 2 |  | 0 |  | yes | 5394.70 | 5068.89 | 4802.46 | 4894.13 |
| VISLSGEHSIIGR | 1419 | 33.96 | 3 | 1366.7564 | 3 |  | 0 |  | yes | 2602.54 | 2639.47 | 2238.00 | 2312.47 |
| VISLSGEHSIIGR | 5543 | --- | --- | 1366.7548 | 2 |  | 0 |  | yes | 893.88 | 708.00 | 682.00 | 523.96 |

  

| Tags | |
| --- | --- |

  

##

## P61982

1433G\_MOUSE
14-3-3 protein gamma OS=Mus musculus GN=Ywhag PE=1 SV=2  
4
peptides

  

| Sequence | Peptide Ion | Score | Hits | Mass | Charge | Tags | Conflicts | Modifications | In quantitation | Average Normalised Abundances | | | |
| --- | --- | --- | --- | --- | --- | --- | --- | --- | --- | --- | --- | --- | --- |
|  |  |  |  |  |  |  |  |  |  | 2m2f | 4m2f | 2m4f | 4m4f |
| DSTLIMQLLR | 2121 | 76.05 | 3 | 1188.6524 | 2 |  | 3 |  | yes | 5173.61 | 3874.97 | 5291.93 | 4679.16 |
| NLLSVAYK | 1420 | 43.60 | 3 | 906.5168 | 2 |  | 3 |  | no | 1821.69 | 1728.75 | 1828.48 | 2020.75 |
| NVTELNEPLSNEER | 7942 | 41.62 | 2 | 1642.7800 | 2 |  | 0 |  | yes | 1107.12 | 645.83 | 826.77 | 749.26 |
| VISSIEQK | 3333 | 42.93 | 3 | 902.5062 | 2 |  | 1 |  | yes | 1123.73 | 851.37 | 1004.75 | 878.00 |

  

| Tags | |
| --- | --- |

  

##

## P12032

TIMP1\_MOUSE
Metalloproteinase inhibitor 1 OS=Mus musculus GN=Timp1 PE=2 SV=2  
4
peptides

  

| Sequence | Peptide Ion | Score | Hits | Mass | Charge | Tags | Conflicts | Modifications | In quantitation | Average Normalised Abundances | | | |
| --- | --- | --- | --- | --- | --- | --- | --- | --- | --- | --- | --- | --- | --- |
|  |  |  |  |  |  |  |  |  |  | 2m2f | 4m2f | 2m4f | 4m4f |
| LESDTHCLWTDQVLVGSEDYQSR | 5333 | 52.95 | 3 | 2737.2286 | 3 |  | 0 |  | yes | 3031.74 | 1856.48 | 2357.67 | 836.40 |
| NPGLCTWR | 4455 | 43.01 | 2 | 1002.4706 | 2 |  | 0 |  | yes | 922.04 | 812.26 | 714.61 | 421.93 |
| SEEFLITGR | 2864 | 76.98 | 3 | 1050.5335 | 2 |  | 0 |  | yes | 1357.31 | 1423.76 | 1097.25 | 1006.62 |
| YAYTPVMESLCGYAHK | 6851 | 30.59 | 3 | 1888.8474 | 3 |  | 0 |  | no | 906.88 | 573.02 | 762.91 | 307.52 |

  

| Tags | |
| --- | --- |

  

##

## P35564

CALX\_MOUSE
Calnexin OS=Mus musculus GN=Canx PE=1 SV=1  
3 peptides

  

| Sequence | Peptide Ion | Score | Hits | Mass | Charge | Tags | Conflicts | Modifications | In quantitation | Average Normalised Abundances | | | |
| --- | --- | --- | --- | --- | --- | --- | --- | --- | --- | --- | --- | --- | --- |
|  |  |  |  |  |  |  |  |  |  | 2m2f | 4m2f | 2m4f | 4m4f |
| APVPTGEVYFADSFDR | 5559 | 61.72 | 3 | 1769.8287 | 2 |  | 0 |  | yes | 2179.16 | 1199.91 | 2309.15 | 1530.92 |
| GSLSGWILSK | 6143 | 48.24 | 1 | 1046.5740 | 2 |  | 0 |  | yes | 704.92 | 526.90 | 718.65 | 588.94 |
| VVDDWANDGWGLK | 5473 | 89.91 | 3 | 1473.6892 | 2 |  | 0 |  | yes | 1770.32 | 1322.28 | 1754.82 | 1370.07 |

  

| Tags | |
| --- | --- |

  

##

## P48962

ADT1\_MOUSE
ADP/ATP translocase 1 OS=Mus musculus GN=Slc25a4 PE=1 SV=4  
4
peptides

  

| Sequence | Peptide Ion | Score | Hits | Mass | Charge | Tags | Conflicts | Modifications | In quantitation | Average Normalised Abundances | | | |
| --- | --- | --- | --- | --- | --- | --- | --- | --- | --- | --- | --- | --- | --- |
|  |  |  |  |  |  |  |  |  |  | 2m2f | 4m2f | 2m4f | 4m4f |
| DFLAGGIAAAVSK | 1940 | 43.45 | 3 | 1218.6618 | 2 |  | 1 |  | no | 4291.56 | 3936.71 | 4956.37 | 3547.91 |
| GAWSNVLR | 2797 | 57.99 | 3 | 901.4764 | 2 |  | 1 |  | no | 895.51 | 796.12 | 853.63 | 919.35 |
| LLLQVQHASK | 7966 | 37.72 | 3 | 1135.6711 | 2 |  | 1 |  | no | 433.23 | 378.22 | 328.28 | 688.06 |
| LLLQVQHASK | 3287 | --- | --- | 1135.6718 | 3 |  | 1 |  | no | 713.89 | 666.18 | 695.94 | 855.13 |
| YFPTQALNFAFK | 4097 | 56.29 | 3 | 1445.7342 | 2 |  | 1 |  | no | 4087.87 | 1815.62 | 3868.63 | 3271.53 |

  

| Tags | |
| --- | --- |

  

##

## P48036

ANXA5\_MOUSE
Annexin A5 OS=Mus musculus GN=Anxa5 PE=1 SV=1  
3 peptides

  

| Sequence | Peptide Ion | Score | Hits | Mass | Charge | Tags | Conflicts | Modifications | In quantitation | Average Normalised Abundances | | | |
| --- | --- | --- | --- | --- | --- | --- | --- | --- | --- | --- | --- | --- | --- |
|  |  |  |  |  |  |  |  |  |  | 2m2f | 4m2f | 2m4f | 4m4f |
| ETSGNLEQLLLAVVK | 19974 | 65.73 | 3 | 1612.9032 | 2 |  | 0 |  | yes | 603.49 | 44.45 | 293.96 | 71.45 |
| GLGTDEDSILNLLTSR | 35173 | --- | --- | 1702.8738 | 3 |  | 0 |  | yes | 101.36 | 0.00 | 86.78 | 19.18 |
| GLGTDEDSILNLLTSR | 8583 | 74.51 | 2 | 1702.8727 | 2 |  | 0 |  | yes | 2116.34 | 383.05 | 1543.81 | 513.93 |
| SEIDLFNIR | 7791 | 53.78 | 3 | 1105.5765 | 2 |  | 0 |  | yes | 428.55 | 398.71 | 433.57 | 370.37 |

  

| Tags | |
| --- | --- |

  

##

## Q09098

PATE4\_MOUSE
Prostate and testis expressed protein 4 OS=Mus musculus GN=Pate4 PE=1
SV=3  
3 peptides

  

| Sequence | Peptide Ion | Score | Hits | Mass | Charge | Tags | Conflicts | Modifications | In quantitation | Average Normalised Abundances | | | |
| --- | --- | --- | --- | --- | --- | --- | --- | --- | --- | --- | --- | --- | --- |
|  |  |  |  |  |  |  |  |  |  | 2m2f | 4m2f | 2m4f | 4m4f |
| CVAKPGESCSTVSHFVGTK | 1787 | 65.17 | 3 | 2049.9588 | 3 |  | 0 |  | yes | 5987.57 | 4613.34 | 6394.45 | 5315.15 |
| CVAKPGESCSTVSHFVGTK | 1286 | 53.01 | 3 | 2049.9598 | 4 |  | 0 |  | yes | 4358.44 | 4104.14 | 5190.44 | 4210.99 |
| LIYIMCCEK | 126 | 48.54 | 3 | 1228.5642 | 2 |  | 0 |  | yes | 7.03e+004 | 2.15e+005 | 1.03e+005 | 1.94e+005 |
| LIYIMCCEK | 2235 | 26.59 | 3 | 1244.5604 | 2 |  | 0 | [5] Oxidation (M) | yes | 2946.15 | 4558.61 | 2708.61 | 4603.42 |

  

| Tags | |
| --- | --- |

  

##

## P02762

MUP6\_MOUSE
Major urinary protein 6 OS=Mus musculus GN=Mup6 PE=1 SV=2  
3
peptides

  

| Sequence | Peptide Ion | Score | Hits | Mass | Charge | Tags | Conflicts | Modifications | In quantitation | Average Normalised Abundances | | | |
| --- | --- | --- | --- | --- | --- | --- | --- | --- | --- | --- | --- | --- | --- |
|  |  |  |  |  |  |  |  |  |  | 2m2f | 4m2f | 2m4f | 4m4f |
| DEECSELSMVADK | 10297 | 96.65 | 2 | 1511.6085 | 2 |  | 0 |  | yes | 659.23 | 776.39 | 820.22 | 1646.71 |
| ENIIDLSNANR | 5368 | 55.67 | 3 | 1257.6308 | 2 |  | 0 |  | yes | 749.71 | 125.74 | 1534.80 | 82.65 |
| FAQLCEEHGILR | 9460 | 38.17 | 2 | 1471.7235 | 3 |  | 0 |  | yes | 200.12 | 7.00 | 315.53 | 13.90 |

  

| Tags | |
| --- | --- |

  

##

## Q9D8E6

RL4\_MOUSE
60S ribosomal protein L4 OS=Mus musculus GN=Rpl4 PE=1 SV=3  
4
peptides

  

| Sequence | Peptide Ion | Score | Hits | Mass | Charge | Tags | Conflicts | Modifications | In quantitation | Average Normalised Abundances | | | |
| --- | --- | --- | --- | --- | --- | --- | --- | --- | --- | --- | --- | --- | --- |
|  |  |  |  |  |  |  |  |  |  | 2m2f | 4m2f | 2m4f | 4m4f |
| IEEVPELPLVVEDK | 2563 | 33.22 | 3 | 1607.8660 | 2 |  | 0 |  | yes | 7267.07 | 6452.33 | 7482.29 | 5489.10 |
| LDELYGTWR | 5281 | 57.45 | 2 | 1151.5605 | 2 |  | 0 |  | no | 755.72 | 704.17 | 669.63 | 686.13 |
| NIPGITLLNVSK | 2719 | 51.99 | 3 | 1267.7499 | 2 |  | 0 |  | yes | 2855.99 | 2845.08 | 3124.21 | 2773.38 |
| YAICSALAASALPALVMSK | 13425 | 47.36 | 3 | 1936.0146 | 2 |  | 0 |  | yes | 1120.45 | 312.91 | 913.90 | 395.35 |
| YAICSALAASALPALVMSK | 13113 | --- | --- | 1936.0179 | 3 |  | 0 |  | yes | 623.76 | 149.83 | 543.39 | 197.58 |

  

| Tags | |
| --- | --- |

  

##

## Q01853

TERA\_MOUSE
Transitional endoplasmic reticulum ATPase OS=Mus musculus GN=Vcp PE=1
SV=4  
4 peptides

  

| Sequence | Peptide Ion | Score | Hits | Mass | Charge | Tags | Conflicts | Modifications | In quantitation | Average Normalised Abundances | | | |
| --- | --- | --- | --- | --- | --- | --- | --- | --- | --- | --- | --- | --- | --- |
|  |  |  |  |  |  |  |  |  |  | 2m2f | 4m2f | 2m4f | 4m4f |
| DVDLEFLAK | 5974 | 34.28 | 3 | 1048.5498 | 2 |  | 0 |  | no | 739.20 | 625.88 | 728.91 | 714.28 |
| IVSQLLTLMDGLK | 9113 | 46.99 | 2 | 1429.8134 | 2 |  | 0 |  | yes | 2674.18 | 459.55 | 1151.16 | 960.83 |
| LGDVISIQPCPDVK | 5151 | 74.78 | 3 | 1539.7962 | 2 |  | 0 |  | yes | 1446.38 | 701.35 | 1181.30 | 930.07 |
| NAPAIIFIDELDAIAPK | 19065 | 30.51 | 3 | 1809.9866 | 3 |  | 0 |  | yes | 871.77 | 96.82 | 595.18 | 268.26 |
| NAPAIIFIDELDAIAPK | 13936 | --- | --- | 1809.9879 | 2 |  | 0 |  | yes | 3307.93 | 432.14 | 2323.89 | 1184.99 |

  

| Tags | |
| --- | --- |

  

##

## P21460

CYTC\_MOUSE
Cystatin-C OS=Mus musculus GN=Cst3 PE=2 SV=2  
3 peptides

  

| Sequence | Peptide Ion | Score | Hits | Mass | Charge | Tags | Conflicts | Modifications | In quantitation | Average Normalised Abundances | | | |
| --- | --- | --- | --- | --- | --- | --- | --- | --- | --- | --- | --- | --- | --- |
|  |  |  |  |  |  |  |  |  |  | 2m2f | 4m2f | 2m4f | 4m4f |
| ALCSFQIYSVPWK | 4866 | 63.05 | 3 | 1597.7970 | 2 |  | 0 |  | yes | 1976.03 | 2676.65 | 2212.48 | 2095.84 |
| ALDFAVSEYNK | 2289 | 66.35 | 3 | 1255.6080 | 2 |  | 0 |  | yes | 2960.73 | 4282.39 | 2697.91 | 3564.46 |
| MLGAPEEADANEEGVR | 3922 | 56.31 | 2 | 1686.7518 | 2 |  | 0 |  | yes | 2703.28 | 4081.85 | 2446.83 | 2889.47 |

  

| Tags | |
| --- | --- |

  

##

## Q9D1G1

RAB1B\_MOUSE
Ras-related protein Rab-1B OS=Mus musculus GN=Rab1b PE=1 SV=1  
4
peptides

  

| Sequence | Peptide Ion | Score | Hits | Mass | Charge | Tags | Conflicts | Modifications | In quantitation | Average Normalised Abundances | | | |
| --- | --- | --- | --- | --- | --- | --- | --- | --- | --- | --- | --- | --- | --- |
|  |  |  |  |  |  |  |  |  |  | 2m2f | 4m2f | 2m4f | 4m4f |
| EFADSLGVPFLETSAK | 9661 | 32.91 | 2 | 1709.8507 | 2 |  | 0 |  | yes | 1454.65 | 723.81 | 1295.24 | 868.16 |
| FADDTYTESYISTIGVDFK | 12372 | 44.06 | 2 | 2170.9959 | 2 |  | 1 |  | yes | 1213.51 | 403.50 | 875.51 | 253.74 |
| FADDTYTESYISTIGVDFK | 9087 | 28.13 | 3 | 2170.9948 | 3 |  | 1 |  | yes | 946.70 | 395.22 | 850.73 | 148.13 |
| LLLIGDSGVGK | 2022 | 43.69 | 3 | 1070.6324 | 2 |  | 2 |  | yes | 2121.19 | 2152.22 | 1924.28 | 2150.47 |
| TITSSYYR | 5531 | 36.86 | 3 | 989.4816 | 2 |  | 1 |  | no | 711.12 | 484.20 | 603.13 | 484.43 |

  

| Tags | |
| --- | --- |

  

##

## Q8VHX6

FLNC\_MOUSE
Filamin-C OS=Mus musculus GN=Flnc PE=1 SV=3  
4 peptides

  

| Sequence | Peptide Ion | Score | Hits | Mass | Charge | Tags | Conflicts | Modifications | In quantitation | Average Normalised Abundances | | | |
| --- | --- | --- | --- | --- | --- | --- | --- | --- | --- | --- | --- | --- | --- |
|  |  |  |  |  |  |  |  |  |  | 2m2f | 4m2f | 2m4f | 4m4f |
| IECDDKGDGSCDVR | 6720 | 47.40 | 3 | 1624.6449 | 3 |  | 1 |  | no | 659.81 | 205.30 | 867.58 | 493.93 |
| LIALLEVLSQK | 5086 | 62.70 | 3 | 1225.7644 | 2 |  | 1 |  | no | 2359.91 | 608.15 | 2757.47 | 869.96 |
| LVSIDSK | 7329 | 45.02 | 3 | 760.4324 | 2 |  | 1 |  | no | 306.54 | 91.13 | 256.42 | 179.20 |
| NSFTVDCSK | 10153 | 30.38 | 1 | 1056.4550 | 2 |  | 1 |  | no | 379.14 | 28.83 | 332.28 | 134.88 |

  

| Tags | |
| --- | --- |

  

##

## P62821

RAB1A\_MOUSE
Ras-related protein Rab-1A OS=Mus musculus GN=Rab1A PE=1 SV=3  
4
peptides

  

| Sequence | Peptide Ion | Score | Hits | Mass | Charge | Tags | Conflicts | Modifications | In quantitation | Average Normalised Abundances | | | |
| --- | --- | --- | --- | --- | --- | --- | --- | --- | --- | --- | --- | --- | --- |
|  |  |  |  |  |  |  |  |  |  | 2m2f | 4m2f | 2m4f | 4m4f |
| EFADSLGIPFLETSAK | 4592 | 32.26 | 3 | 1723.8669 | 2 |  | 0 |  | yes | 7550.89 | 3762.78 | 6897.80 | 5261.10 |
| FADDTYTESYISTIGVDFK | 9087 | 28.13 | 3 | 2170.9948 | 3 |  | 1 |  | yes | 946.70 | 395.22 | 850.73 | 148.13 |
| FADDTYTESYISTIGVDFK | 12372 | 44.06 | 2 | 2170.9959 | 2 |  | 1 |  | yes | 1213.51 | 403.50 | 875.51 | 253.74 |
| LLLIGDSGVGK | 2022 | 43.69 | 3 | 1070.6324 | 2 |  | 2 |  | yes | 2121.19 | 2152.22 | 1924.28 | 2150.47 |
| TITSSYYR | 5531 | 36.86 | 3 | 989.4816 | 2 |  | 1 |  | no | 711.12 | 484.20 | 603.13 | 484.43 |

  

| Tags | |
| --- | --- |

  

##

## Q9QY48

DNS2B\_MOUSE
Deoxyribonuclease-2-beta OS=Mus musculus GN=Dnase2b PE=2 SV=1  
4
peptides

  

| Sequence | Peptide Ion | Score | Hits | Mass | Charge | Tags | Conflicts | Modifications | In quantitation | Average Normalised Abundances | | | |
| --- | --- | --- | --- | --- | --- | --- | --- | --- | --- | --- | --- | --- | --- |
|  |  |  |  |  |  |  |  |  |  | 2m2f | 4m2f | 2m4f | 4m4f |
| NEYGEAVDWFIFYK | 20921 | 43.48 | 3 | 1779.8135 | 2 |  | 0 |  | no | 280.03 | 140.45 | 483.16 | 134.54 |
| SSFYTDDIFTGWIAQK | 16633 | --- | --- | 1877.8829 | 3 |  | 0 |  | yes | 246.98 | 188.40 | 356.14 | 122.33 |
| SSFYTDDIFTGWIAQK | 6948 | 45.90 | 3 | 1877.8849 | 2 |  | 0 |  | yes | 2764.73 | 1894.52 | 3187.19 | 1180.08 |
| TQGFWLIHSVPK | 5125 | 34.91 | 3 | 1411.7603 | 3 |  | 0 |  | yes | 576.93 | 447.27 | 409.23 | 503.82 |
| WTCIGDLNR | 3190 | 59.80 | 3 | 1133.5284 | 2 |  | 0 |  | yes | 1295.29 | 1428.27 | 1448.37 | 1466.51 |

  

| Tags | |
| --- | --- |

  

##

## Q78PY7

SND1\_MOUSE
Staphylococcal nuclease domain-containing protein 1 OS=Mus musculus GN=Snd1 PE=1
SV=1  
3 peptides

  

| Sequence | Peptide Ion | Score | Hits | Mass | Charge | Tags | Conflicts | Modifications | In quantitation | Average Normalised Abundances | | | |
| --- | --- | --- | --- | --- | --- | --- | --- | --- | --- | --- | --- | --- | --- |
|  |  |  |  |  |  |  |  |  |  | 2m2f | 4m2f | 2m4f | 4m4f |
| EVEVEVESMDK | 13349 | 30.30 | 3 | 1292.5832 | 2 |  | 0 |  | yes | 556.03 | 139.14 | 330.45 | 233.20 |
| SEAVVEYVFSGSR | 6112 | 96.75 | 3 | 1428.6860 | 2 |  | 0 |  | yes | 990.17 | 815.82 | 952.00 | 841.38 |
| VITEYLNAQESAK | 5775 | 55.19 | 3 | 1464.7461 | 2 |  | 0 |  | yes | 3194.64 | 3148.54 | 2896.76 | 2765.36 |

  

| Tags | |
| --- | --- |

  

##

## Q921I1

TRFE\_MOUSE
Serotransferrin OS=Mus musculus GN=Tf PE=1 SV=1  
4 peptides

  

| Sequence | Peptide Ion | Score | Hits | Mass | Charge | Tags | Conflicts | Modifications | In quantitation | Average Normalised Abundances | | | |
| --- | --- | --- | --- | --- | --- | --- | --- | --- | --- | --- | --- | --- | --- |
|  |  |  |  |  |  |  |  |  |  | 2m2f | 4m2f | 2m4f | 4m4f |
| DFQLFSSPLGK | 7374 | 53.75 | 3 | 1237.6334 | 2 |  | 0 |  | yes | 1045.69 | 732.80 | 819.22 | 740.63 |
| DGGGDVAFVK | 11617 | 67.99 | 2 | 963.4658 | 2 |  | 0 |  | yes | 332.39 | 257.61 | 256.99 | 173.40 |
| EDLIWEILK | 14276 | 26.38 | 3 | 1157.6324 | 2 |  | 0 |  | no | 348.30 | 105.52 | 294.68 | 158.95 |
| SAGWVIPIGLLFCK | 12170 | 33.44 | 3 | 1559.8532 | 2 |  | 0 |  | yes | 1633.75 | 552.97 | 1629.92 | 551.47 |

  

| Tags | |
| --- | --- |

  

##

## Q01768

NDKB\_MOUSE
Nucleoside diphosphate kinase B OS=Mus musculus GN=Nme2 PE=1 SV=1  
4
peptides

  

| Sequence | Peptide Ion | Score | Hits | Mass | Charge | Tags | Conflicts | Modifications | In quantitation | Average Normalised Abundances | | | |
| --- | --- | --- | --- | --- | --- | --- | --- | --- | --- | --- | --- | --- | --- |
|  |  |  |  |  |  |  |  |  |  | 2m2f | 4m2f | 2m4f | 4m4f |
| DRPFFPGLVK | 7651 | --- | --- | 1174.6500 | 3 |  | 0 |  | yes | 186.53 | 86.90 | 191.44 | 157.56 |
| DRPFFPGLVK | 7849 | 29.49 | 2 | 1174.6454 | 2 |  | 0 |  | yes | 711.21 | 964.33 | 663.94 | 1232.82 |
| GDFCIQVGR | 3248 | 66.63 | 3 | 1050.4915 | 2 |  | 1 |  | no | 1396.99 | 1334.28 | 1327.05 | 1243.88 |
| NIIHGSDSVESAEK | 2036 | 54.55 | 3 | 1484.7151 | 3 |  | 0 |  | yes | 3204.00 | 3779.56 | 2913.55 | 5119.24 |
| NIIHGSDSVESAEK | 8884 | --- | --- | 1484.7099 | 2 |  | 0 |  | yes | 1080.97 | 1240.02 | 968.79 | 1827.10 |
| TFIAIKPDGVQR | 9037 | --- | --- | 1343.7547 | 2 |  | 1 |  | yes | 470.22 | 248.59 | 223.75 | 266.09 |
| TFIAIKPDGVQR | 2397 | 30.00 | 3 | 1343.7550 | 3 |  | 1 |  | yes | 1523.75 | 1115.27 | 1105.12 | 1235.05 |

  

| Tags | |
| --- | --- |

  

##

## Q3SXH3

Q3SXH3\_MOUSE
Seminal vesicle antigen OS=Mus musculus GN=Sva PE=2 SV=1  
3
peptides

  

| Sequence | Peptide Ion | Score | Hits | Mass | Charge | Tags | Conflicts | Modifications | In quantitation | Average Normalised Abundances | | | |
| --- | --- | --- | --- | --- | --- | --- | --- | --- | --- | --- | --- | --- | --- |
|  |  |  |  |  |  |  |  |  |  | 2m2f | 4m2f | 2m4f | 4m4f |
| ITPSTSITNK | 26 | 59.69 | 3 | 1060.5759 | 2 |  | 0 |  | yes | 3.09e+005 | 4.79e+005 | 3.54e+005 | 4.11e+005 |
| ITPSTSITNKK | 1464 | --- | --- | 1188.6708 | 3 |  | 0 |  | yes | 1909.24 | 3786.00 | 2552.64 | 3230.13 |
| ITPSTSITNKK | 4879 | 31.97 | 1 | 1188.6712 | 2 |  | 0 |  | yes | 736.18 | 1402.04 | 919.85 | 1239.90 |
| QCQEVEINTEHER | 12315 | 25.74 | 3 | 1670.7325 | 2 |  | 0 |  | yes | 304.93 | 684.27 | 339.67 | 29.64 |
| QCQEVEINTEHER | 1015 | 58.81 | 3 | 1670.7296 | 3 |  | 0 |  | yes | 8341.51 | 8619.44 | 6778.63 | 3538.80 |

  

| Tags | |
| --- | --- |

  

##

## P01942

HBA\_MOUSE
Hemoglobin subunit alpha OS=Mus musculus GN=Hba PE=1 SV=2  
3
peptides

  

| Sequence | Peptide Ion | Score | Hits | Mass | Charge | Tags | Conflicts | Modifications | In quantitation | Average Normalised Abundances | | | |
| --- | --- | --- | --- | --- | --- | --- | --- | --- | --- | --- | --- | --- | --- |
|  |  |  |  |  |  |  |  |  |  | 2m2f | 4m2f | 2m4f | 4m4f |
| IGGHGAEYGAEALER | 448 | 55.50 | 3 | 1528.7260 | 3 |  | 0 |  | yes | 1.29e+004 | 1.35e+004 | 1.39e+004 | 1.41e+004 |
| IGGHGAEYGAEALER | 3504 | 56.37 | 3 | 1528.7267 | 2 |  | 0 |  | yes | 2096.45 | 2069.65 | 2088.91 | 2364.31 |
| MFASFPTTK | 392 | 36.37 | 3 | 1028.4996 | 2 |  | 0 |  | yes | 1.18e+004 | 1.67e+004 | 1.59e+004 | 1.39e+004 |
| TYFPHFDVSHGSAQVK | 21515 | --- | --- | 1818.8629 | 2 |  | 0 |  | yes | 221.33 | 23.31 | 93.65 | 40.95 |
| TYFPHFDVSHGSAQVK | 1217 | --- | --- | 1818.8689 | 4 |  | 0 |  | yes | 4647.13 | 2577.52 | 4989.54 | 2862.24 |
| TYFPHFDVSHGSAQVK | 1069 | 27.93 | 3 | 1818.8810 | 3 |  | 0 |  | yes | 8857.74 | 7592.81 | 8401.67 | 8792.78 |

  

| Tags | |
| --- | --- |

  

##

## P29341

PABP1\_MOUSE
Polyadenylate-binding protein 1 OS=Mus musculus GN=Pabpc1 PE=1 SV=1  
4
peptides

  

| Sequence | Peptide Ion | Score | Hits | Mass | Charge | Tags | Conflicts | Modifications | In quantitation | Average Normalised Abundances | | | |
| --- | --- | --- | --- | --- | --- | --- | --- | --- | --- | --- | --- | --- | --- |
|  |  |  |  |  |  |  |  |  |  | 2m2f | 4m2f | 2m4f | 4m4f |
| ALDTMNFDVIK | 6625 | 58.92 | 3 | 1265.6245 | 2 |  | 0 |  | yes | 897.93 | 979.32 | 1153.49 | 862.26 |
| ALYDTFSAFGNILSCK | 10312 | 57.51 | 3 | 1805.8773 | 2 |  | 0 |  | yes | 3016.56 | 800.02 | 1752.65 | 1299.93 |
| EFSPFGTITSAK | 6816 | 28.31 | 2 | 1283.6392 | 2 |  | 0 |  | yes | 845.23 | 649.64 | 676.73 | 797.69 |
| GFGFVSFER | 7777 | 25.86 | 3 | 1044.5023 | 2 |  | 0 |  | no | 426.77 | 462.13 | 453.38 | 413.75 |

  

| Tags | |
| --- | --- |

  

##

## P0C0S6

H2AZ\_MOUSE
Histone H2A.Z OS=Mus musculus GN=H2afz PE=1 SV=2  
3 peptides

  

| Sequence | Peptide Ion | Score | Hits | Mass | Charge | Tags | Conflicts | Modifications | In quantitation | Average Normalised Abundances | | | |
| --- | --- | --- | --- | --- | --- | --- | --- | --- | --- | --- | --- | --- | --- |
|  |  |  |  |  |  |  |  |  |  | 2m2f | 4m2f | 2m4f | 4m4f |
| AGLQFPVGR | 456 | 75.71 | 3 | 943.5236 | 2 |  | 1 |  | yes | 1.22e+004 | 7299.51 | 1.01e+004 | 9960.86 |
| GDEELDSLIK | 3189 | 56.44 | 3 | 1117.5503 | 2 |  | 0 |  | yes | 1575.59 | 1899.70 | 1759.72 | 1696.96 |
| HLQLAIR | 581 | 38.31 | 3 | 849.5181 | 2 |  | 1 |  | yes | 6087.71 | 4628.89 | 5237.33 | 4379.96 |

  

| Tags | |
| --- | --- |

  

##

## Q9DCD0

6PGD\_MOUSE
6-phosphogluconate dehydrogenase, decarboxylating OS=Mus musculus GN=Pgd PE=2
SV=3  
3 peptides

  

| Sequence | Peptide Ion | Score | Hits | Mass | Charge | Tags | Conflicts | Modifications | In quantitation | Average Normalised Abundances | | | |
| --- | --- | --- | --- | --- | --- | --- | --- | --- | --- | --- | --- | --- | --- |
|  |  |  |  |  |  |  |  |  |  | 2m2f | 4m2f | 2m4f | 4m4f |
| GILFVGSGVSGGEEGAR | 6106 | 74.56 | 3 | 1590.7965 | 2 |  | 0 |  | yes | 1147.23 | 1048.56 | 1012.14 | 820.15 |
| LVPLLDTGDIIIDGGNSEYR | 13274 | 25.17 | 2 | 2159.1100 | 2 |  | 0 |  | yes | 1392.78 | 574.08 | 1101.15 | 441.69 |
| LVPLLDTGDIIIDGGNSEYR | 6895 | --- | --- | 2159.1112 | 3 |  | 0 |  | yes | 2871.51 | 1493.29 | 2558.85 | 939.12 |
| NPELQNLLLDDFFK | 10093 | 68.09 | 3 | 1704.8725 | 2 |  | 0 |  | yes | 2078.65 | 495.91 | 1486.08 | 655.91 |

  

| Tags | |
| --- | --- |

  

##

## P47963

RL13\_MOUSE
60S ribosomal protein L13 OS=Mus musculus GN=Rpl13 PE=2 SV=3  
3
peptides

  

| Sequence | Peptide Ion | Score | Hits | Mass | Charge | Tags | Conflicts | Modifications | In quantitation | Average Normalised Abundances | | | |
| --- | --- | --- | --- | --- | --- | --- | --- | --- | --- | --- | --- | --- | --- |
|  |  |  |  |  |  |  |  |  |  | 2m2f | 4m2f | 2m4f | 4m4f |
| GFSLEELR | 4036 | 36.97 | 3 | 949.4862 | 2 |  | 0 |  | yes | 770.53 | 640.69 | 733.93 | 654.60 |
| LATQLTGPVMPIR | 4872 | 55.57 | 3 | 1395.7874 | 2 |  | 0 |  | yes | 1910.58 | 1419.45 | 1718.13 | 1548.95 |
| STESLQANVQR | 3586 | 75.04 | 3 | 1231.6157 | 2 |  | 0 |  | yes | 1786.73 | 1899.87 | 1630.53 | 2591.28 |

  

| Tags | |
| --- | --- |

  

##

## P14824

ANXA6\_MOUSE
Annexin A6 OS=Mus musculus GN=Anxa6 PE=1 SV=2  
3 peptides

  

| Sequence | Peptide Ion | Score | Hits | Mass | Charge | Tags | Conflicts | Modifications | In quantitation | Average Normalised Abundances | | | |
| --- | --- | --- | --- | --- | --- | --- | --- | --- | --- | --- | --- | --- | --- |
|  |  |  |  |  |  |  |  |  |  | 2m2f | 4m2f | 2m4f | 4m4f |
| DAFVAIVQSVK | 9377 | 57.53 | 3 | 1175.6552 | 2 |  | 0 |  | yes | 456.15 | 988.21 | 797.19 | 819.28 |
| ESILELITSR | 10444 | 49.94 | 3 | 1159.6441 | 2 |  | 0 |  | yes | 583.55 | 98.16 | 398.56 | 292.55 |
| TLIEILATR | 3558 | 27.04 | 3 | 1028.6214 | 2 |  | 1 |  | no | 989.21 | 967.63 | 1111.79 | 1014.18 |
| TLIEILATR | 9465 | 58.81 | 3 | 1028.6217 | 2 |  | 0 |  | yes | 274.48 | 123.03 | 249.40 | 122.85 |

  

| Tags | |
| --- | --- |

  

##

## P14148

RL7\_MOUSE
60S ribosomal protein L7 OS=Mus musculus GN=Rpl7 PE=2 SV=2  
3
peptides

  

| Sequence | Peptide Ion | Score | Hits | Mass | Charge | Tags | Conflicts | Modifications | In quantitation | Average Normalised Abundances | | | |
| --- | --- | --- | --- | --- | --- | --- | --- | --- | --- | --- | --- | --- | --- |
|  |  |  |  |  |  |  |  |  |  | 2m2f | 4m2f | 2m4f | 4m4f |
| EANNFLWPFK | 3776 | 46.02 | 3 | 1264.6235 | 2 |  | 0 |  | yes | 4961.87 | 2818.20 | 4430.79 | 4284.08 |
| IALTDNSLIAR | 2205 | 78.27 | 2 | 1185.6675 | 2 |  | 0 |  | yes | 2965.50 | 2973.58 | 2436.05 | 3841.35 |
| SVNELIYK | 3323 | 38.80 | 3 | 964.5231 | 2 |  | 0 |  | yes | 1224.54 | 1320.46 | 1133.49 | 1057.21 |

  

| Tags | |
| --- | --- |

  

##

## Q9DBF1

AL7A1\_MOUSE
Alpha-aminoadipic semialdehyde dehydrogenase OS=Mus musculus GN=Aldh7a1 PE=1
SV=4  
2 peptides

  

| Sequence | Peptide Ion | Score | Hits | Mass | Charge | Tags | Conflicts | Modifications | In quantitation | Average Normalised Abundances | | | |
| --- | --- | --- | --- | --- | --- | --- | --- | --- | --- | --- | --- | --- | --- |
|  |  |  |  |  |  |  |  |  |  | 2m2f | 4m2f | 2m4f | 4m4f |
| AWNIWADIPAPK | 10760 | 57.99 | 3 | 1380.7154 | 2 |  | 0 |  | yes | 724.21 | 125.12 | 511.74 | 327.24 |
| VNLLSFTGSTQVGK | 4994 | 104.27 | 3 | 1449.7813 | 2 |  | 0 |  | yes | 1588.54 | 903.41 | 1835.42 | 718.08 |

  

| Tags | |
| --- | --- |

  

##

## P05202

AATM\_MOUSE
Aspartate aminotransferase, mitochondrial OS=Mus musculus GN=Got2 PE=1
SV=1  
3 peptides

  

| Sequence | Peptide Ion | Score | Hits | Mass | Charge | Tags | Conflicts | Modifications | In quantitation | Average Normalised Abundances | | | |
| --- | --- | --- | --- | --- | --- | --- | --- | --- | --- | --- | --- | --- | --- |
|  |  |  |  |  |  |  |  |  |  | 2m2f | 4m2f | 2m4f | 4m4f |
| ASAELALGENNEVLK | 4889 | 34.14 | 2 | 1556.8019 | 2 |  | 0 |  | yes | 1960.06 | 1374.85 | 1978.96 | 1210.48 |
| EYLPIGGLAEFCK | 6180 | 49.54 | 3 | 1495.7378 | 2 |  | 0 |  | yes | 1378.15 | 934.93 | 1142.63 | 1143.63 |
| TCGFDFSGALEDISK | 4379 | 77.77 | 3 | 1645.7294 | 2 |  | 0 |  | yes | 3729.80 | 2497.19 | 3398.57 | 2669.82 |

  

| Tags | |
| --- | --- |

  

##

## P35700

PRDX1\_MOUSE
Peroxiredoxin-1 OS=Mus musculus GN=Prdx1 PE=1 SV=1  
3
peptides

  

| Sequence | Peptide Ion | Score | Hits | Mass | Charge | Tags | Conflicts | Modifications | In quantitation | Average Normalised Abundances | | | |
| --- | --- | --- | --- | --- | --- | --- | --- | --- | --- | --- | --- | --- | --- |
|  |  |  |  |  |  |  |  |  |  | 2m2f | 4m2f | 2m4f | 4m4f |
| GLFIIDDK | 2273 | 49.97 | 3 | 919.5014 | 2 |  | 0 |  | yes | 1271.49 | 1521.23 | 1380.22 | 1630.96 |
| LVQAFQFTDK | 2529 | 60.95 | 3 | 1195.6232 | 2 |  | 0 |  | yes | 2247.57 | 2413.01 | 2278.38 | 2388.83 |
| TIAQDYGVLK | 2179 | 50.13 | 3 | 1106.5966 | 2 |  | 0 |  | yes | 2638.78 | 2769.66 | 2465.55 | 2688.96 |

  

| Tags | |
| --- | --- |

  

##

## P22752

H2A1\_MOUSE
Histone H2A type 1 OS=Mus musculus GN=Hist1h2ab PE=1 SV=3  
3
peptides

  

| Sequence | Peptide Ion | Score | Hits | Mass | Charge | Tags | Conflicts | Modifications | In quantitation | Average Normalised Abundances | | | |
| --- | --- | --- | --- | --- | --- | --- | --- | --- | --- | --- | --- | --- | --- |
|  |  |  |  |  |  |  |  |  |  | 2m2f | 4m2f | 2m4f | 4m4f |
| AGLQFPVGR | 456 | 75.71 | 3 | 943.5236 | 2 |  | 1 |  | yes | 1.22e+004 | 7299.51 | 1.01e+004 | 9960.86 |
| HLQLAIR | 581 | 38.31 | 3 | 849.5181 | 2 |  | 1 |  | yes | 6087.71 | 4628.89 | 5237.33 | 4379.96 |
| VTIAQGGVLPNIQAVLLPK | 1457 | --- | --- | 1930.1618 | 2 |  | 0 |  | yes | 5.04e+004 | 1.58e+004 | 4.13e+004 | 2.21e+004 |
| VTIAQGGVLPNIQAVLLPK | 1005 | 46.09 | 3 | 1930.1609 | 3 |  | 0 |  | yes | 4.36e+004 | 1.49e+004 | 3.75e+004 | 1.92e+004 |

  

| Tags | |
| --- | --- |

  

##

## P17879

HS71B\_MOUSE
Heat shock 70 kDa protein 1B OS=Mus musculus GN=Hspa1b PE=1 SV=3  
3
peptides

  

| Sequence | Peptide Ion | Score | Hits | Mass | Charge | Tags | Conflicts | Modifications | In quantitation | Average Normalised Abundances | | | |
| --- | --- | --- | --- | --- | --- | --- | --- | --- | --- | --- | --- | --- | --- |
|  |  |  |  |  |  |  |  |  |  | 2m2f | 4m2f | 2m4f | 4m4f |
| SFFPEEISSMVLTK | 17008 | 31.95 | 1 | 1613.8003 | 2 |  | 0 |  | yes | 503.00 | 65.59 | 473.22 | 214.96 |
| TTPSYVAFTDTER | 1603 | 65.20 | 3 | 1486.6941 | 2 |  | 1 |  | yes | 6267.03 | 4584.84 | 5135.16 | 4726.15 |
| VEIIANDQGNR | 1006 | 60.83 | 3 | 1227.6195 | 2 |  | 2 |  | yes | 7512.51 | 4609.63 | 5361.71 | 5790.14 |

  

| Tags | |
| --- | --- |

  

##

## P43274

H14\_MOUSE
Histone H1.4 OS=Mus musculus GN=Hist1h1e PE=1 SV=2  
2
peptides

  

| Sequence | Peptide Ion | Score | Hits | Mass | Charge | Tags | Conflicts | Modifications | In quantitation | Average Normalised Abundances | | | |
| --- | --- | --- | --- | --- | --- | --- | --- | --- | --- | --- | --- | --- | --- |
|  |  |  |  |  |  |  |  |  |  | 2m2f | 4m2f | 2m4f | 4m4f |
| SGVSLAALK | 926 | 64.96 | 3 | 844.5011 | 2 |  | 0 |  | yes | 4487.26 | 4949.57 | 4407.79 | 4524.93 |
| TSGPPVSELITK | 1100 | 88.95 | 3 | 1227.6704 | 2 |  | 0 |  | yes | 1.07e+004 | 1.21e+004 | 9782.86 | 1.16e+004 |

  

| Tags | |
| --- | --- |

  

##

## Q6PDN3

MYLK\_MOUSE
Myosin light chain kinase, smooth muscle OS=Mus musculus GN=Mylk PE=1
SV=3  
3 peptides

  

| Sequence | Peptide Ion | Score | Hits | Mass | Charge | Tags | Conflicts | Modifications | In quantitation | Average Normalised Abundances | | | |
| --- | --- | --- | --- | --- | --- | --- | --- | --- | --- | --- | --- | --- | --- |
|  |  |  |  |  |  |  |  |  |  | 2m2f | 4m2f | 2m4f | 4m4f |
| IEGYPDPEVVWFK | 2937 | 35.70 | 3 | 1577.7768 | 2 |  | 0 |  | yes | 8771.64 | 6671.48 | 1.05e+004 | 8586.74 |
| IIDEDFELTER | 7461 | 68.55 | 3 | 1378.6584 | 2 |  | 0 |  | yes | 1195.67 | 2664.03 | 1232.64 | 3074.49 |
| SSTGSPTSPINAEK | 4294 | 48.87 | 3 | 1374.6601 | 2 |  | 0 |  | yes | 2215.51 | 1598.74 | 2173.02 | 2519.76 |

  

| Tags | |
| --- | --- |

  

##

## P01887

B2MG\_MOUSE
Beta-2-microglobulin OS=Mus musculus GN=B2m PE=1 SV=1  
3
peptides

  

| Sequence | Peptide Ion | Score | Hits | Mass | Charge | Tags | Conflicts | Modifications | In quantitation | Average Normalised Abundances | | | |
| --- | --- | --- | --- | --- | --- | --- | --- | --- | --- | --- | --- | --- | --- |
|  |  |  |  |  |  |  |  |  |  | 2m2f | 4m2f | 2m4f | 4m4f |
| TPQIQVYSR | 759 | 57.76 | 3 | 1090.5762 | 2 |  | 0 |  | yes | 4670.08 | 1.26e+004 | 7645.60 | 1.05e+004 |
| TVYWDR | 118 | 30.22 | 3 | 838.3973 | 2 |  | 0 |  | yes | 3.48e+004 | 6.01e+004 | 4.94e+004 | 5.57e+004 |
| VEMSDMSFSK | 227 | 64.25 | 3 | 1159.4887 | 2 |  | 0 |  | yes | 3.29e+004 | 6.46e+004 | 3.84e+004 | 6.82e+004 |

  

| Tags | |
| --- | --- |

  

##

## P62702

RS4X\_MOUSE
40S ribosomal protein S4, X isoform OS=Mus musculus GN=Rps4x PE=2
SV=2  
3 peptides

  

| Sequence | Peptide Ion | Score | Hits | Mass | Charge | Tags | Conflicts | Modifications | In quantitation | Average Normalised Abundances | | | |
| --- | --- | --- | --- | --- | --- | --- | --- | --- | --- | --- | --- | --- | --- |
|  |  |  |  |  |  |  |  |  |  | 2m2f | 4m2f | 2m4f | 4m4f |
| ECLPLIIFLR | 8522 | 44.14 | 3 | 1272.7262 | 2 |  | 0 |  | yes | 1873.34 | 609.67 | 1693.16 | 1529.73 |
| HPGSFDVVHVK | 15392 | 38.48 | 1 | 1220.6287 | 3 |  | 0 |  | yes | 108.52 | 12.73 | 45.82 | 55.49 |
| LSNIFVIGK | 3407 | 68.07 | 3 | 989.5918 | 2 |  | 0 |  | yes | 937.93 | 1021.33 | 1027.04 | 1127.17 |

  

| Tags | |
| --- | --- |

  

##

## P62270

RS18\_MOUSE
40S ribosomal protein S18 OS=Mus musculus GN=Rps18 PE=2 SV=3  
3
peptides

  

| Sequence | Peptide Ion | Score | Hits | Mass | Charge | Tags | Conflicts | Modifications | In quantitation | Average Normalised Abundances | | | |
| --- | --- | --- | --- | --- | --- | --- | --- | --- | --- | --- | --- | --- | --- |
|  |  |  |  |  |  |  |  |  |  | 2m2f | 4m2f | 2m4f | 4m4f |
| AGELTEDEVER | 2780 | 69.04 | 3 | 1246.5670 | 2 |  | 0 |  | yes | 2259.07 | 1627.69 | 1756.10 | 1435.95 |
| IAFAITAIK | 5570 | 44.11 | 3 | 946.5848 | 2 |  | 0 |  | yes | 360.29 | 270.45 | 354.20 | 312.86 |
| IPDWFLNR | 3521 | 36.45 | 3 | 1059.5495 | 2 |  | 0 |  | yes | 1654.40 | 1361.42 | 1640.70 | 1740.52 |

  

| Tags | |
| --- | --- |

  

##

## P32921

SYWC\_MOUSE
Tryptophanyl-tRNA synthetase, cytoplasmic OS=Mus musculus GN=Wars PE=1
SV=2  
4 peptides

  

| Sequence | Peptide Ion | Score | Hits | Mass | Charge | Tags | Conflicts | Modifications | In quantitation | Average Normalised Abundances | | | |
| --- | --- | --- | --- | --- | --- | --- | --- | --- | --- | --- | --- | --- | --- |
|  |  |  |  |  |  |  |  |  |  | 2m2f | 4m2f | 2m4f | 4m4f |
| ASEDFVDPWTVR | 5683 | 48.41 | 3 | 1420.6634 | 2 |  | 0 |  | yes | 934.42 | 1023.20 | 1121.67 | 925.01 |
| DLTLEQAYSYTVENAK | 14822 | 29.38 | 1 | 1843.8822 | 3 |  | 0 |  | yes | 303.39 | 135.28 | 335.07 | 135.27 |
| DLTLEQAYSYTVENAK | 11291 | --- | --- | 1843.8848 | 2 |  | 0 |  | yes | 669.96 | 253.83 | 765.40 | 315.56 |
| DMNQILDAYENK | 12835 | 25.56 | 1 | 1452.6546 | 2 |  | 0 |  | no | 339.86 | 179.18 | 277.69 | 170.14 |
| GIFGFTDSDCIGK | 6516 | 44.43 | 3 | 1415.6385 | 2 |  | 0 |  | yes | 1465.95 | 3150.03 | 1631.57 | 2289.07 |

  

| Tags | |
| --- | --- |

  

##

## P00329

ADH1\_MOUSE
Alcohol dehydrogenase 1 OS=Mus musculus GN=Adh1 PE=2 SV=2  
2
peptides

  

| Sequence | Peptide Ion | Score | Hits | Mass | Charge | Tags | Conflicts | Modifications | In quantitation | Average Normalised Abundances | | | |
| --- | --- | --- | --- | --- | --- | --- | --- | --- | --- | --- | --- | --- | --- |
|  |  |  |  |  |  |  |  |  |  | 2m2f | 4m2f | 2m4f | 4m4f |
| INEAFDLLR | 2946 | 64.21 | 3 | 1089.5817 | 2 |  | 0 |  | yes | 1342.95 | 1346.09 | 1411.46 | 1351.30 |
| VCLIGCGFSTGYGSAVK | 6533 | 82.98 | 3 | 1774.8475 | 2 |  | 0 |  | yes | 1648.19 | 1206.06 | 1547.96 | 1276.44 |

  

| Tags | |
| --- | --- |

  

##

## P70296

PEBP1\_MOUSE
Phosphatidylethanolamine-binding protein 1 OS=Mus musculus GN=Pebp1 PE=1
SV=3  
2 peptides

  

| Sequence | Peptide Ion | Score | Hits | Mass | Charge | Tags | Conflicts | Modifications | In quantitation | Average Normalised Abundances | | | |
| --- | --- | --- | --- | --- | --- | --- | --- | --- | --- | --- | --- | --- | --- |
|  |  |  |  |  |  |  |  |  |  | 2m2f | 4m2f | 2m4f | 4m4f |
| LYTLVLTDPDAPSR | 3361 | 69.79 | 3 | 1559.8149 | 2 |  | 0 |  | yes | 2376.87 | 2288.06 | 2416.37 | 1945.11 |
| VDYAGVTVDELGK | 2888 | 74.81 | 3 | 1364.6795 | 2 |  | 0 |  | yes | 2653.59 | 2387.63 | 2083.85 | 2569.02 |

  

| Tags | |
| --- | --- |

  

##

## Q61171

PRDX2\_MOUSE
Peroxiredoxin-2 OS=Mus musculus GN=Prdx2 PE=1 SV=3  
2
peptides

  

| Sequence | Peptide Ion | Score | Hits | Mass | Charge | Tags | Conflicts | Modifications | In quantitation | Average Normalised Abundances | | | |
| --- | --- | --- | --- | --- | --- | --- | --- | --- | --- | --- | --- | --- | --- |
|  |  |  |  |  |  |  |  |  |  | 2m2f | 4m2f | 2m4f | 4m4f |
| EGGLGPLNIPLLADVTK | 14615 | 43.12 | 2 | 1705.9608 | 2 |  | 0 |  | yes | 1656.83 | 126.88 | 1120.51 | 517.50 |
| SAPDFTATAVVDGAFK | 4270 | 100.24 | 3 | 1595.7825 | 2 |  | 0 |  | yes | 2630.81 | 2449.27 | 2933.41 | 1941.41 |

  

| Tags | |
| --- | --- |

  

##

## Q8BGZ7

K2C75\_MOUSE
Keratin, type II cytoskeletal 75 OS=Mus musculus GN=Krt75 PE=1 SV=1  
2
peptides

  

| Sequence | Peptide Ion | Score | Hits | Mass | Charge | Tags | Conflicts | Modifications | In quantitation | Average Normalised Abundances | | | |
| --- | --- | --- | --- | --- | --- | --- | --- | --- | --- | --- | --- | --- | --- |
|  |  |  |  |  |  |  |  |  |  | 2m2f | 4m2f | 2m4f | 4m4f |
| AQYEDIANR | 3103 | 49.96 | 3 | 1078.5020 | 2 |  | 1 |  | yes | 1340.89 | 1091.66 | 1127.04 | 1121.65 |
| SLDLDSIIAEVK | 4472 | 92.30 | 3 | 1301.7075 | 2 |  | 0 |  | yes | 2303.04 | 1859.68 | 1891.13 | 1397.89 |

  

| Tags | |
| --- | --- |

  

##

## P08003

PDIA4\_MOUSE
Protein disulfide-isomerase A4 OS=Mus musculus GN=Pdia4 PE=1 SV=2  
3
peptides

  

| Sequence | Peptide Ion | Score | Hits | Mass | Charge | Tags | Conflicts | Modifications | In quantitation | Average Normalised Abundances | | | |
| --- | --- | --- | --- | --- | --- | --- | --- | --- | --- | --- | --- | --- | --- |
|  |  |  |  |  |  |  |  |  |  | 2m2f | 4m2f | 2m4f | 4m4f |
| FDVSGYPTIK | 5715 | 28.40 | 1 | 1125.5704 | 2 |  | 0 |  | yes | 852.57 | 434.41 | 676.09 | 607.90 |
| IDATSASMLASK | 9890 | 48.26 | 1 | 1193.5954 | 2 |  | 0 |  | yes | 471.04 | 174.34 | 304.54 | 236.57 |
| VDATEQTDLAK | 4903 | 62.65 | 3 | 1189.5806 | 2 |  | 0 |  | yes | 1212.26 | 896.22 | 1031.46 | 1047.57 |

  

| Tags | |
| --- | --- |

  

##

## P62082

RS7\_MOUSE
40S ribosomal protein S7 OS=Mus musculus GN=Rps7 PE=2 SV=1  
3
peptides

  

| Sequence | Peptide Ion | Score | Hits | Mass | Charge | Tags | Conflicts | Modifications | In quantitation | Average Normalised Abundances | | | |
| --- | --- | --- | --- | --- | --- | --- | --- | --- | --- | --- | --- | --- | --- |
|  |  |  |  |  |  |  |  |  |  | 2m2f | 4m2f | 2m4f | 4m4f |
| AIIIFVPVPQLK | 3940 | 56.75 | 3 | 1336.8480 | 2 |  | 0 |  | yes | 4783.43 | 2329.24 | 4419.87 | 4136.62 |
| DVNFEFPEFQL | 6548 | 38.42 | 3 | 1383.6344 | 2 |  | 0 |  | yes | 3788.85 | 2226.01 | 3844.40 | 2715.08 |
| VETFSGVYK | 3753 | 42.74 | 3 | 1028.5178 | 2 |  | 0 |  | yes | 1123.31 | 1007.37 | 1061.36 | 1073.54 |

  

| Tags | |
| --- | --- |

  

##

## P07356

ANXA2\_MOUSE
Annexin A2 OS=Mus musculus GN=Anxa2 PE=1 SV=2  
2 peptides

  

| Sequence | Peptide Ion | Score | Hits | Mass | Charge | Tags | Conflicts | Modifications | In quantitation | Average Normalised Abundances | | | |
| --- | --- | --- | --- | --- | --- | --- | --- | --- | --- | --- | --- | --- | --- |
|  |  |  |  |  |  |  |  |  |  | 2m2f | 4m2f | 2m4f | 4m4f |
| GVDEVTIVNILTNR | 16325 | --- | --- | 1541.8404 | 3 |  | 0 |  | yes | 176.79 | 44.54 | 135.08 | 77.29 |
| GVDEVTIVNILTNR | 7053 | 87.93 | 3 | 1541.8422 | 2 |  | 0 |  | yes | 1254.54 | 557.10 | 1205.65 | 694.37 |
| TNQELQEINR | 8526 | 49.91 | 1 | 1243.6137 | 2 |  | 0 |  | yes | 745.68 | 495.43 | 670.93 | 647.21 |

  

| Tags | |
| --- | --- |

  

##

## Q60854

SPB6\_MOUSE
Serpin B6 OS=Mus musculus GN=Serpinb6 PE=2 SV=1  
3 peptides

  

| Sequence | Peptide Ion | Score | Hits | Mass | Charge | Tags | Conflicts | Modifications | In quantitation | Average Normalised Abundances | | | |
| --- | --- | --- | --- | --- | --- | --- | --- | --- | --- | --- | --- | --- | --- |
|  |  |  |  |  |  |  |  |  |  | 2m2f | 4m2f | 2m4f | 4m4f |
| GTTASQMAQALALDK | 8773 | 48.90 | 3 | 1504.7561 | 2 |  | 0 |  | yes | 1006.68 | 576.04 | 847.61 | 709.80 |
| MTYIGEIFTK | 5415 | 37.27 | 2 | 1201.6038 | 2 |  | 0 |  | yes | 1422.49 | 1046.39 | 1374.52 | 892.64 |
| TCDLLASFK | 6034 | 49.60 | 3 | 1053.5164 | 2 |  | 0 |  | yes | 605.98 | 516.81 | 615.78 | 648.85 |

  

| Tags | |
| --- | --- |

  

##

## O70251

EF1B\_MOUSE
Elongation factor 1-beta OS=Mus musculus GN=Eef1b PE=1 SV=5  
2
peptides

  

| Sequence | Peptide Ion | Score | Hits | Mass | Charge | Tags | Conflicts | Modifications | In quantitation | Average Normalised Abundances | | | |
| --- | --- | --- | --- | --- | --- | --- | --- | --- | --- | --- | --- | --- | --- |
|  |  |  |  |  |  |  |  |  |  | 2m2f | 4m2f | 2m4f | 4m4f |
| SIQADGLVWGSSK | 4857 | 65.47 | 3 | 1346.6820 | 2 |  | 0 |  | yes | 1599.90 | 2205.92 | 1794.31 | 1729.17 |
| TPAGLQVLNDYLADK | 3631 | 70.19 | 3 | 1616.8407 | 2 |  | 0 |  | yes | 4696.92 | 2594.15 | 3479.56 | 3523.53 |

  

| Tags | |
| --- | --- |

  

##

## P07901

HS90A\_MOUSE
Heat shock protein HSP 90-alpha OS=Mus musculus GN=Hsp90aa1 PE=1 SV=4  
3
peptides

  

| Sequence | Peptide Ion | Score | Hits | Mass | Charge | Tags | Conflicts | Modifications | In quantitation | Average Normalised Abundances | | | |
| --- | --- | --- | --- | --- | --- | --- | --- | --- | --- | --- | --- | --- | --- |
|  |  |  |  |  |  |  |  |  |  | 2m2f | 4m2f | 2m4f | 4m4f |
| EDQTEYLEER | 4373 | 39.74 | 3 | 1310.5657 | 2 |  | 0 |  | yes | 1611.99 | 1265.09 | 1388.41 | 1355.60 |
| ELISNSSDALDK | 7086 | 47.97 | 3 | 1290.6346 | 2 |  | 0 |  | yes | 686.96 | 429.61 | 519.41 | 543.52 |
| YIDQEELNK | 4875 | 45.73 | 3 | 1150.5487 | 2 |  | 0 |  | yes | 857.11 | 674.87 | 662.53 | 575.55 |

  

| Tags | |
| --- | --- |

  

##

## Q61598

GDIB\_MOUSE
Rab GDP dissociation inhibitor beta OS=Mus musculus GN=Gdi2 PE=1 SV=1  
2
peptides

  

| Sequence | Peptide Ion | Score | Hits | Mass | Charge | Tags | Conflicts | Modifications | In quantitation | Average Normalised Abundances | | | |
| --- | --- | --- | --- | --- | --- | --- | --- | --- | --- | --- | --- | --- | --- |
|  |  |  |  |  |  |  |  |  |  | 2m2f | 4m2f | 2m4f | 4m4f |
| DLGTDSQIFISR | 6886 | 60.47 | 2 | 1350.6766 | 2 |  | 0 |  | yes | 864.57 | 470.30 | 699.30 | 400.87 |
| FVSISDLFVPK | 4131 | 72.18 | 3 | 1250.6884 | 2 |  | 0 |  | yes | 2931.23 | 1777.46 | 2832.17 | 2995.97 |

  

| Tags | |
| --- | --- |

  

##

## P05784

K1C18\_MOUSE
Keratin, type I cytoskeletal 18 OS=Mus musculus GN=Krt18 PE=1 SV=5  
3
peptides

  

| Sequence | Peptide Ion | Score | Hits | Mass | Charge | Tags | Conflicts | Modifications | In quantitation | Average Normalised Abundances | | | |
| --- | --- | --- | --- | --- | --- | --- | --- | --- | --- | --- | --- | --- | --- |
|  |  |  |  |  |  |  |  |  |  | 2m2f | 4m2f | 2m4f | 4m4f |
| IVLQIDNAR | 1700 | 42.43 | 1 | 1040.5936 | 2 |  | 1 |  | yes | 3347.26 | 3544.67 | 3095.19 | 3529.25 |
| LAADDFR | 2306 | 38.29 | 3 | 806.3962 | 2 |  | 1 |  | yes | 1748.71 | 1860.41 | 1598.20 | 1781.20 |
| TLQTLEIDLDSMK | 7594 | 50.33 | 3 | 1505.7625 | 2 |  | 0 |  | yes | 681.59 | 392.94 | 736.33 | 399.10 |

  

| Tags | |
| --- | --- |

  

##

## Q9D1D4

TMEDA\_MOUSE
Transmembrane emp24 domain-containing protein 10 OS=Mus musculus GN=Tmed10 PE=2
SV=1  
2 peptides

  

| Sequence | Peptide Ion | Score | Hits | Mass | Charge | Tags | Conflicts | Modifications | In quantitation | Average Normalised Abundances | | | |
| --- | --- | --- | --- | --- | --- | --- | --- | --- | --- | --- | --- | --- | --- |
|  |  |  |  |  |  |  |  |  |  | 2m2f | 4m2f | 2m4f | 4m4f |
| IPDQLVILDMK | 3938 | 81.62 | 3 | 1283.7160 | 2 |  | 0 |  | yes | 1992.76 | 1293.63 | 1802.22 | 1412.33 |
| LEDLSESIVNDFAYMK | 24846 | --- | --- | 1872.8949 | 3 |  | 0 |  | yes | 353.71 | 162.65 | 287.86 | 167.97 |
| LEDLSESIVNDFAYMK | 11618 | 49.30 | 3 | 1872.8897 | 2 |  | 0 |  | yes | 1420.07 | 404.18 | 1239.59 | 447.31 |

  

| Tags | |
| --- | --- |

  

##

## P15532

NDKA\_MOUSE
Nucleoside diphosphate kinase A OS=Mus musculus GN=Nme1 PE=1 SV=1  
3
peptides

  

| Sequence | Peptide Ion | Score | Hits | Mass | Charge | Tags | Conflicts | Modifications | In quantitation | Average Normalised Abundances | | | |
| --- | --- | --- | --- | --- | --- | --- | --- | --- | --- | --- | --- | --- | --- |
|  |  |  |  |  |  |  |  |  |  | 2m2f | 4m2f | 2m4f | 4m4f |
| EISLWFQPEELVEYK | 10186 | 34.27 | 3 | 1908.9514 | 2 |  | 0 |  | yes | 2438.04 | 499.97 | 1758.28 | 636.20 |
| GDFCIQVGR | 3248 | 66.63 | 3 | 1050.4915 | 2 |  | 1 |  | yes | 1396.99 | 1334.28 | 1327.05 | 1243.88 |
| TFIAIKPDGVQR | 2397 | 30.00 | 3 | 1343.7550 | 3 |  | 1 |  | yes | 1523.75 | 1115.27 | 1105.12 | 1235.05 |
| TFIAIKPDGVQR | 9037 | --- | --- | 1343.7547 | 2 |  | 1 |  | yes | 470.22 | 248.59 | 223.75 | 266.09 |

  

| Tags | |
| --- | --- |

  

##

## Q8BP67

RL24\_MOUSE
60S ribosomal protein L24 OS=Mus musculus GN=Rpl24 PE=2 SV=2  
2
peptides

  

| Sequence | Peptide Ion | Score | Hits | Mass | Charge | Tags | Conflicts | Modifications | In quantitation | Average Normalised Abundances | | | |
| --- | --- | --- | --- | --- | --- | --- | --- | --- | --- | --- | --- | --- | --- |
|  |  |  |  |  |  |  |  |  |  | 2m2f | 4m2f | 2m4f | 4m4f |
| AITGASLADIMAK | 4946 | 77.07 | 3 | 1260.6729 | 2 |  | 0 |  | yes | 1106.32 | 1082.28 | 1226.82 | 944.81 |
| VFQFLNAK | 3704 | 52.82 | 3 | 965.5325 | 2 |  | 0 |  | yes | 673.25 | 719.67 | 977.55 | 923.79 |

  

| Tags | |
| --- | --- |

  

##

## P97351

RS3A\_MOUSE
40S ribosomal protein S3a OS=Mus musculus GN=Rps3a PE=1 SV=3  
3
peptides

  

| Sequence | Peptide Ion | Score | Hits | Mass | Charge | Tags | Conflicts | Modifications | In quantitation | Average Normalised Abundances | | | |
| --- | --- | --- | --- | --- | --- | --- | --- | --- | --- | --- | --- | --- | --- |
|  |  |  |  |  |  |  |  |  |  | 2m2f | 4m2f | 2m4f | 4m4f |
| ACQSIYPLHDVFVR | 6823 | 35.60 | 3 | 1703.8446 | 3 |  | 0 |  | yes | 821.40 | 293.07 | 664.54 | 600.97 |
| TTDGYLLR | 5104 | 44.33 | 3 | 937.4874 | 2 |  | 0 |  | yes | 596.67 | 482.32 | 561.25 | 425.07 |
| VFEVSLADLQNDEVAFR | 9462 | 48.87 | 3 | 1950.9687 | 2 |  | 0 |  | yes | 2780.83 | 665.52 | 1387.33 | 528.47 |

  

| Tags | |
| --- | --- |

  

##

## Q9QZE5

COPG\_MOUSE
Coatomer subunit gamma OS=Mus musculus GN=Copg PE=2 SV=1  
3
peptides

  

| Sequence | Peptide Ion | Score | Hits | Mass | Charge | Tags | Conflicts | Modifications | In quantitation | Average Normalised Abundances | | | |
| --- | --- | --- | --- | --- | --- | --- | --- | --- | --- | --- | --- | --- | --- |
|  |  |  |  |  |  |  |  |  |  | 2m2f | 4m2f | 2m4f | 4m4f |
| AIVDCIISIIEENSESK | 22887 | 32.17 | 3 | 1918.9547 | 3 |  | 0 |  | yes | 298.71 | 70.12 | 219.40 | 140.50 |
| SIATLAITTLLK | 5941 | 70.19 | 3 | 1243.7745 | 2 |  | 0 |  | yes | 2206.55 | 747.97 | 1994.71 | 1031.57 |
| TLEEAVGNIVK | 6057 | 25.51 | 2 | 1171.6483 | 2 |  | 1 |  | no | 1107.55 | 1263.05 | 955.12 | 1045.99 |

  

| Tags | |
| --- | --- |

  

##

## P62889

RL30\_MOUSE
60S ribosomal protein L30 OS=Mus musculus GN=Rpl30 PE=2 SV=2  
2
peptides

  

| Sequence | Peptide Ion | Score | Hits | Mass | Charge | Tags | Conflicts | Modifications | In quantitation | Average Normalised Abundances | | | |
| --- | --- | --- | --- | --- | --- | --- | --- | --- | --- | --- | --- | --- | --- |
|  |  |  |  |  |  |  |  |  |  | 2m2f | 4m2f | 2m4f | 4m4f |
| LVILANNCPALR | 3455 | 75.10 | 3 | 1352.7588 | 2 |  | 0 |  | yes | 1955.50 | 1672.25 | 1656.23 | 1784.67 |
| VCTLAIIDPGDSDIIR | 3396 | 52.52 | 3 | 1756.8973 | 2 |  | 0 |  | yes | 5095.00 | 4851.02 | 5279.95 | 3153.83 |

  

| Tags | |
| --- | --- |

  

##

## Q91YR9

PTGR1\_MOUSE
Prostaglandin reductase 1 OS=Mus musculus GN=Ptgr1 PE=2 SV=2  
3
peptides

  

| Sequence | Peptide Ion | Score | Hits | Mass | Charge | Tags | Conflicts | Modifications | In quantitation | Average Normalised Abundances | | | |
| --- | --- | --- | --- | --- | --- | --- | --- | --- | --- | --- | --- | --- | --- |
|  |  |  |  |  |  |  |  |  |  | 2m2f | 4m2f | 2m4f | 4m4f |
| ALTELMNWVSEGK | 6659 | 61.42 | 3 | 1476.7284 | 2 |  | 0 |  | yes | 1728.48 | 801.86 | 1177.79 | 881.36 |
| LGFDVAFNYK | 9280 | 31.88 | 3 | 1172.5866 | 2 |  | 0 |  | yes | 522.84 | 324.94 | 367.30 | 109.62 |
| MEGFIVNR | 11620 | 30.63 | 3 | 964.4801 | 2 |  | 0 |  | yes | 214.88 | 139.85 | 152.56 | 120.98 |

  

| Tags | |
| --- | --- |

  

##

## P14131

RS16\_MOUSE
40S ribosomal protein S16 OS=Mus musculus GN=Rps16 PE=2 SV=4  
2
peptides

  

| Sequence | Peptide Ion | Score | Hits | Mass | Charge | Tags | Conflicts | Modifications | In quantitation | Average Normalised Abundances | | | |
| --- | --- | --- | --- | --- | --- | --- | --- | --- | --- | --- | --- | --- | --- |
|  |  |  |  |  |  |  |  |  |  | 2m2f | 4m2f | 2m4f | 4m4f |
| GPLQSVQVFGR | 4952 | 77.46 | 1 | 1186.6425 | 2 |  | 0 |  | yes | 1358.92 | 1670.77 | 1070.42 | 1733.74 |
| LLEPVLLLGK | 1715 | 46.09 | 3 | 1093.7106 | 2 |  | 0 |  | yes | 5927.00 | 4595.79 | 6178.53 | 5970.83 |

  

| Tags | |
| --- | --- |

  

##

## P63038

CH60\_MOUSE
60 kDa heat shock protein, mitochondrial OS=Mus musculus GN=Hspd1 PE=1
SV=1  
2 peptides

  

| Sequence | Peptide Ion | Score | Hits | Mass | Charge | Tags | Conflicts | Modifications | In quantitation | Average Normalised Abundances | | | |
| --- | --- | --- | --- | --- | --- | --- | --- | --- | --- | --- | --- | --- | --- |
|  |  |  |  |  |  |  |  |  |  | 2m2f | 4m2f | 2m4f | 4m4f |
| TLNDELEIIEGMK | 11588 | 52.15 | 3 | 1503.7491 | 2 |  | 0 |  | yes | 676.26 | 181.92 | 427.20 | 229.24 |
| VGGTSDVEVNEK | 7598 | 71.37 | 3 | 1232.5876 | 2 |  | 0 |  | yes | 750.49 | 798.91 | 789.25 | 1113.04 |

  

| Tags | |
| --- | --- |

  

##

## Q9CY50

SSRA\_MOUSE
Translocon-associated protein subunit alpha OS=Mus musculus GN=Ssr1 PE=1
SV=1  
2 peptides

  

| Sequence | Peptide Ion | Score | Hits | Mass | Charge | Tags | Conflicts | Modifications | In quantitation | Average Normalised Abundances | | | |
| --- | --- | --- | --- | --- | --- | --- | --- | --- | --- | --- | --- | --- | --- |
|  |  |  |  |  |  |  |  |  |  | 2m2f | 4m2f | 2m4f | 4m4f |
| FLVGFTNK | 4984 | 30.22 | 3 | 924.5066 | 2 |  | 0 |  | yes | 529.70 | 412.45 | 432.73 | 469.60 |
| GTEDFIVESLDASFR | 13559 | --- | --- | 1684.7926 | 3 |  | 0 |  | yes | 300.29 | 105.39 | 228.88 | 70.82 |
| GTEDFIVESLDASFR | 8538 | 93.12 | 3 | 1684.7930 | 2 |  | 0 |  | yes | 2461.02 | 1107.06 | 1686.65 | 773.49 |

  

| Tags | |
| --- | --- |

  

##

## P62855

RS26\_MOUSE
40S ribosomal protein S26 OS=Mus musculus GN=Rps26 PE=2 SV=3  
2
peptides

  

| Sequence | Peptide Ion | Score | Hits | Mass | Charge | Tags | Conflicts | Modifications | In quantitation | Average Normalised Abundances | | | |
| --- | --- | --- | --- | --- | --- | --- | --- | --- | --- | --- | --- | --- | --- |
|  |  |  |  |  |  |  |  |  |  | 2m2f | 4m2f | 2m4f | 4m4f |
| DISEASVFDAYVLPK | 2988 | 62.88 | 3 | 1652.8301 | 2 |  | 0 |  | yes | 1.03e+004 | 6244.13 | 1.07e+004 | 6527.30 |
| NIVEAAAVR | 3049 | 60.31 | 3 | 941.5287 | 2 |  | 0 |  | yes | 1060.11 | 989.75 | 1068.77 | 979.46 |

  

| Tags | |
| --- | --- |

  

##

## P45878

FKBP2\_MOUSE
Peptidyl-prolyl cis-trans isomerase FKBP2 OS=Mus musculus GN=Fkbp2 PE=1
SV=1  
3 peptides

  

| Sequence | Peptide Ion | Score | Hits | Mass | Charge | Tags | Conflicts | Modifications | In quantitation | Average Normalised Abundances | | | |
| --- | --- | --- | --- | --- | --- | --- | --- | --- | --- | --- | --- | --- | --- |
|  |  |  |  |  |  |  |  |  |  | 2m2f | 4m2f | 2m4f | 4m4f |
| GWDQGLLGMCEGEK | 7928 | 25.02 | 2 | 1578.6853 | 2 |  | 0 |  | yes | 841.16 | 451.86 | 697.96 | 659.71 |
| IPGGATLVFEVELLK | 15831 | 52.26 | 3 | 1584.9117 | 2 |  | 0 |  | yes | 595.49 | 329.52 | 549.54 | 128.00 |
| LVIPSELGYGER | 2048 | 44.76 | 3 | 1331.7080 | 2 |  | 0 |  | yes | 4225.64 | 4980.09 | 3864.51 | 4598.13 |

  

| Tags | |
| --- | --- |

  

##

## Q9CXW4

RL11\_MOUSE
60S ribosomal protein L11 OS=Mus musculus GN=Rpl11 PE=1 SV=4  
2
peptides

  

| Sequence | Peptide Ion | Score | Hits | Mass | Charge | Tags | Conflicts | Modifications | In quantitation | Average Normalised Abundances | | | |
| --- | --- | --- | --- | --- | --- | --- | --- | --- | --- | --- | --- | --- | --- |
|  |  |  |  |  |  |  |  |  |  | 2m2f | 4m2f | 2m4f | 4m4f |
| VLEQLTGQTPVFSK | 9409 | --- | --- | 1545.8439 | 3 |  | 0 |  | yes | 423.25 | 257.25 | 370.60 | 261.30 |
| VLEQLTGQTPVFSK | 4323 | 75.88 | 3 | 1545.8391 | 2 |  | 0 |  | yes | 2215.48 | 2003.91 | 2296.08 | 2151.90 |
| YDGIILPGK | 5332 | 45.58 | 3 | 974.5436 | 2 |  | 0 |  | yes | 617.21 | 452.08 | 545.89 | 441.83 |

  

| Tags | |
| --- | --- |

  

##

## P62830

RL23\_MOUSE
60S ribosomal protein L23 OS=Mus musculus GN=Rpl23 PE=2 SV=1  
3
peptides

  

| Sequence | Peptide Ion | Score | Hits | Mass | Charge | Tags | Conflicts | Modifications | In quantitation | Average Normalised Abundances | | | |
| --- | --- | --- | --- | --- | --- | --- | --- | --- | --- | --- | --- | --- | --- |
|  |  |  |  |  |  |  |  |  |  | 2m2f | 4m2f | 2m4f | 4m4f |
| ISLGLPVGAVINCADNTGAK | 4264 | 41.87 | 3 | 1969.0315 | 3 |  | 0 |  | yes | 2611.39 | 1727.26 | 2741.15 | 1880.66 |
| ISLGLPVGAVINCADNTGAK | 5163 | --- | --- | 1969.0315 | 2 |  | 0 |  | yes | 3371.36 | 1989.74 | 2931.73 | 2289.68 |
| LPAAGVGDMVMATVK | 6475 | 46.10 | 3 | 1458.7588 | 2 |  | 0 |  | yes | 1384.30 | 745.74 | 1104.82 | 866.07 |
| LPAAGVGDMVMATVK | 24704 | --- | --- | 1458.7598 | 3 |  | 0 |  | yes | 122.21 | 26.20 | 59.67 | 32.79 |
| NLYIISVK | 3573 | 32.82 | 3 | 948.5640 | 2 |  | 0 |  | yes | 972.88 | 832.85 | 902.91 | 882.83 |

  

| Tags | |
| --- | --- |

  

##

## Q9EQ20

MMSA\_MOUSE
Methylmalonate-semialdehyde dehydrogenase [acylating], mitochondrial OS=Mus
musculus GN=Aldh6a1 PE=1 SV=1  
3 peptides

  

| Sequence | Peptide Ion | Score | Hits | Mass | Charge | Tags | Conflicts | Modifications | In quantitation | Average Normalised Abundances | | | |
| --- | --- | --- | --- | --- | --- | --- | --- | --- | --- | --- | --- | --- | --- |
|  |  |  |  |  |  |  |  |  |  | 2m2f | 4m2f | 2m4f | 4m4f |
| AISFVGSNQAGEYIFER | 11104 | 27.15 | 3 | 1886.9149 | 2 |  | 0 |  | yes | 714.49 | 258.15 | 611.98 | 314.09 |
| EGASILLDGR | 12159 | 59.37 | 2 | 1029.5462 | 2 |  | 0 |  | yes | 534.92 | 731.23 | 656.13 | 605.87 |
| ENTLNQLVGAAFGAAGQR | 17748 | 32.83 | 1 | 1815.9265 | 3 |  | 0 |  | yes | 281.65 | 45.33 | 166.41 | 52.90 |

  

| Tags | |
| --- | --- |

  

##

## P67984

RL22\_MOUSE
60S ribosomal protein L22 OS=Mus musculus GN=Rpl22 PE=2 SV=2  
2
peptides

  

| Sequence | Peptide Ion | Score | Hits | Mass | Charge | Tags | Conflicts | Modifications | In quantitation | Average Normalised Abundances | | | |
| --- | --- | --- | --- | --- | --- | --- | --- | --- | --- | --- | --- | --- | --- |
|  |  |  |  |  |  |  |  |  |  | 2m2f | 4m2f | 2m4f | 4m4f |
| AGNLGGGVVTIER | 3054 | 51.98 | 3 | 1241.6725 | 2 |  | 0 |  | yes | 1697.31 | 1535.61 | 1496.01 | 1781.33 |
| ITVTSEVPFSK | 2489 | 65.16 | 3 | 1206.6432 | 2 |  | 0 |  | yes | 4159.34 | 5382.03 | 5953.91 | 5267.20 |

  

| Tags | |
| --- | --- |

  

##

## Q9JIF7

COPB\_MOUSE
Coatomer subunit beta OS=Mus musculus GN=Copb1 PE=1 SV=1  
2
peptides

  

| Sequence | Peptide Ion | Score | Hits | Mass | Charge | Tags | Conflicts | Modifications | In quantitation | Average Normalised Abundances | | | |
| --- | --- | --- | --- | --- | --- | --- | --- | --- | --- | --- | --- | --- | --- |
|  |  |  |  |  |  |  |  |  |  | 2m2f | 4m2f | 2m4f | 4m4f |
| TLQLALDLVSSR | 10103 | 62.28 | 3 | 1314.7429 | 2 |  | 0 |  | yes | 758.78 | 302.10 | 499.56 | 250.52 |
| VLQDLVMDILR | 11610 | 54.30 | 3 | 1313.7367 | 2 |  | 0 |  | yes | 756.60 | 218.20 | 592.51 | 267.21 |

  

| Tags | |
| --- | --- |

  

##

## P35979

RL12\_MOUSE
60S ribosomal protein L12 OS=Mus musculus GN=Rpl12 PE=1 SV=2  
1
peptide

  

| Sequence | Peptide Ion | Score | Hits | Mass | Charge | Tags | Conflicts | Modifications | In quantitation | Average Normalised Abundances | | | |
| --- | --- | --- | --- | --- | --- | --- | --- | --- | --- | --- | --- | --- | --- |
|  |  |  |  |  |  |  |  |  |  | 2m2f | 4m2f | 2m4f | 4m4f |
| EILGTAQSVGCNVDGR | 6778 | 40.19 | 3 | 1674.7985 | 3 |  | 0 |  | yes | 635.45 | 455.22 | 575.65 | 514.15 |
| EILGTAQSVGCNVDGR | 5633 | 75.92 | 3 | 1674.7995 | 2 |  | 0 |  | yes | 1880.59 | 1044.80 | 1643.84 | 1458.25 |

  

| Tags | |
| --- | --- |

  

##

## Q60930

VDAC2\_MOUSE
Voltage-dependent anion-selective channel protein 2 OS=Mus musculus GN=Vdac2
PE=1 SV=2  
2 peptides

  

| Sequence | Peptide Ion | Score | Hits | Mass | Charge | Tags | Conflicts | Modifications | In quantitation | Average Normalised Abundances | | | |
| --- | --- | --- | --- | --- | --- | --- | --- | --- | --- | --- | --- | --- | --- |
|  |  |  |  |  |  |  |  |  |  | 2m2f | 4m2f | 2m4f | 4m4f |
| LTFDTTFSPNTGK | 7634 | 41.61 | 3 | 1427.6925 | 2 |  | 0 |  | yes | 837.41 | 1121.57 | 859.22 | 1122.61 |
| LTLSALVDGK | 7836 | 73.37 | 3 | 1015.5910 | 2 |  | 0 |  | yes | 348.09 | 264.73 | 384.66 | 206.17 |

  

| Tags | |
| --- | --- |

  

##

## Q99PL5

RRBP1\_MOUSE
Ribosome-binding protein 1 OS=Mus musculus GN=Rrbp1 PE=2 SV=2  
3
peptides

  

| Sequence | Peptide Ion | Score | Hits | Mass | Charge | Tags | Conflicts | Modifications | In quantitation | Average Normalised Abundances | | | |
| --- | --- | --- | --- | --- | --- | --- | --- | --- | --- | --- | --- | --- | --- |
|  |  |  |  |  |  |  |  |  |  | 2m2f | 4m2f | 2m4f | 4m4f |
| LLATEQEDAAVAK | 7612 | 43.72 | 3 | 1357.7083 | 2 |  | 0 |  | yes | 998.17 | 1807.00 | 1139.18 | 2533.98 |
| TEATLEAEQTR | 21891 | 36.73 | 1 | 1247.5975 | 2 |  | 0 |  | yes | 320.18 | 198.47 | 186.53 | 196.69 |
| TILAETEGMLK | 10703 | 33.66 | 3 | 1204.6291 | 2 |  | 0 |  | yes | 376.16 | 140.81 | 271.62 | 223.11 |

  

| Tags | |
| --- | --- |

  

##

## Q06890

CLUS\_MOUSE
Clusterin OS=Mus musculus GN=Clu PE=1 SV=1  
2 peptides

  

| Sequence | Peptide Ion | Score | Hits | Mass | Charge | Tags | Conflicts | Modifications | In quantitation | Average Normalised Abundances | | | |
| --- | --- | --- | --- | --- | --- | --- | --- | --- | --- | --- | --- | --- | --- |
|  |  |  |  |  |  |  |  |  |  | 2m2f | 4m2f | 2m4f | 4m4f |
| ASGIIDTLFQDR | 6300 | 57.92 | 1 | 1334.6812 | 2 |  | 0 |  | yes | 1366.09 | 1050.21 | 921.55 | 718.68 |
| LFDSDPITVVLPEEVSK | 24656 | --- | --- | 1886.9853 | 3 |  | 0 |  | yes | 181.34 | 152.42 | 151.95 | 69.59 |
| LFDSDPITVVLPEEVSK | 10616 | 55.84 | 3 | 1886.9885 | 2 |  | 0 |  | yes | 1601.83 | 1263.37 | 1273.62 | 690.33 |

  

| Tags | |
| --- | --- |

  

##

## P07759

SPA3K\_MOUSE
Serine protease inhibitor A3K OS=Mus musculus GN=Serpina3k PE=1 SV=2  
3
peptides

  

| Sequence | Peptide Ion | Score | Hits | Mass | Charge | Tags | Conflicts | Modifications | In quantitation | Average Normalised Abundances | | | |
| --- | --- | --- | --- | --- | --- | --- | --- | --- | --- | --- | --- | --- | --- |
|  |  |  |  |  |  |  |  |  |  | 2m2f | 4m2f | 2m4f | 4m4f |
| AVLDVAETGTEAAAATGVIGGIR | 5078 | 59.82 | 3 | 2141.1330 | 3 |  | 0 |  | yes | 2421.24 | 2130.75 | 3084.91 | 957.71 |
| EVFTEQADLSGITETK | 10795 | 25.89 | 1 | 1766.8574 | 2 |  | 0 |  | yes | 672.05 | 551.15 | 889.43 | 509.12 |
| LEEDVLPEMGIK | 6348 | 27.37 | 3 | 1371.6940 | 2 |  | 0 |  | yes | 997.75 | 1108.98 | 1061.04 | 913.36 |

  

| Tags | |
| --- | --- |

  

##

## P62849

RS24\_MOUSE
40S ribosomal protein S24 OS=Mus musculus GN=Rps24 PE=1 SV=1  
2
peptides

  

| Sequence | Peptide Ion | Score | Hits | Mass | Charge | Tags | Conflicts | Modifications | In quantitation | Average Normalised Abundances | | | |
| --- | --- | --- | --- | --- | --- | --- | --- | --- | --- | --- | --- | --- | --- |
|  |  |  |  |  |  |  |  |  |  | 2m2f | 4m2f | 2m4f | 4m4f |
| TTGFGMIYDSLDYAK | 5503 | 57.02 | 3 | 1680.7708 | 2 |  | 0 |  | yes | 2631.22 | 1761.79 | 2452.82 | 2006.03 |
| TTPDVIFVFGFR | 6127 | 55.98 | 3 | 1397.7340 | 2 |  | 0 |  | yes | 1983.08 | 991.70 | 2349.19 | 1511.99 |

  

| Tags | |
| --- | --- |

  

##

## P84099

RL19\_MOUSE
60S ribosomal protein L19 OS=Mus musculus GN=Rpl19 PE=1 SV=1  
2
peptides

  

| Sequence | Peptide Ion | Score | Hits | Mass | Charge | Tags | Conflicts | Modifications | In quantitation | Average Normalised Abundances | | | |
| --- | --- | --- | --- | --- | --- | --- | --- | --- | --- | --- | --- | --- | --- |
|  |  |  |  |  |  |  |  |  |  | 2m2f | 4m2f | 2m4f | 4m4f |
| LLADQAEAR | 3135 | 60.66 | 3 | 985.5189 | 2 |  | 0 |  | yes | 1388.46 | 984.59 | 1243.22 | 1278.51 |
| VWLDPNETNEIANANSR | 6700 | 51.09 | 3 | 1941.9179 | 2 |  | 0 |  | yes | 1582.20 | 1079.53 | 1434.62 | 1079.62 |

  

| Tags | |
| --- | --- |

  

##

## Q64433

CH10\_MOUSE
10 kDa heat shock protein, mitochondrial OS=Mus musculus GN=Hspe1 PE=1
SV=2  
2 peptides

  

| Sequence | Peptide Ion | Score | Hits | Mass | Charge | Tags | Conflicts | Modifications | In quantitation | Average Normalised Abundances | | | |
| --- | --- | --- | --- | --- | --- | --- | --- | --- | --- | --- | --- | --- | --- |
|  |  |  |  |  |  |  |  |  |  | 2m2f | 4m2f | 2m4f | 4m4f |
| VLQATVVAVGSGGK | 5225 | 79.79 | 3 | 1284.7393 | 2 |  | 0 |  | yes | 1144.59 | 856.08 | 763.98 | 693.32 |
| VVLDDKDYFLFR | 11145 | 31.50 | 3 | 1528.7913 | 3 |  | 0 |  | yes | 290.84 | 58.46 | 172.29 | 109.11 |

  

| Tags | |
| --- | --- |

  

##

## Q3UN54

SSLP1\_MOUSE
Secreted seminal-vesicle Ly-6 protein 1 OS=Mus musculus GN=Sslp1 PE=1
SV=1  
2 peptides

  

| Sequence | Peptide Ion | Score | Hits | Mass | Charge | Tags | Conflicts | Modifications | In quantitation | Average Normalised Abundances | | | |
| --- | --- | --- | --- | --- | --- | --- | --- | --- | --- | --- | --- | --- | --- |
|  |  |  |  |  |  |  |  |  |  | 2m2f | 4m2f | 2m4f | 4m4f |
| CCDHQNLCNKP | 9189 | 26.85 | 3 | 1444.5550 | 2 |  | 0 |  | yes | 771.45 | 744.02 | 768.89 | 1091.15 |
| CCDHQNLCNKP | 1202 | 37.87 | 3 | 1444.5655 | 3 |  | 0 |  | yes | 6203.17 | 7875.38 | 6491.94 | 8679.45 |
| LNSSGICETAETSCEATNNR | 33042 | --- | --- | 2212.9276 | 2 |  | 0 |  | yes | 178.21 | 268.43 | 191.74 | 203.10 |
| LNSSGICETAETSCEATNNR | 4442 | 46.48 | 2 | 2212.9318 | 3 |  | 0 |  | yes | 2269.95 | 3093.69 | 2828.58 | 2534.11 |

  

| Tags | |
| --- | --- |

  

##

## Q9DBJ1

PGAM1\_MOUSE
Phosphoglycerate mutase 1 OS=Mus musculus GN=Pgam1 PE=1 SV=3  
3
peptides

  

| Sequence | Peptide Ion | Score | Hits | Mass | Charge | Tags | Conflicts | Modifications | In quantitation | Average Normalised Abundances | | | |
| --- | --- | --- | --- | --- | --- | --- | --- | --- | --- | --- | --- | --- | --- |
|  |  |  |  |  |  |  |  |  |  | 2m2f | 4m2f | 2m4f | 4m4f |
| ALPFWNEEIVPQIK | 16598 | --- | --- | 1682.9024 | 3 |  | 0 |  | yes | 290.26 | 54.05 | 311.64 | 125.81 |
| ALPFWNEEIVPQIK | 5515 | 39.19 | 3 | 1682.8993 | 2 |  | 0 |  | yes | 5497.52 | 2436.32 | 4498.61 | 3183.72 |
| DAGYEFDICFTSVQK | 16584 | 30.83 | 1 | 1778.7804 | 2 |  | 0 |  | yes | 276.95 | 112.17 | 218.08 | 62.06 |
| FSGWYDADLSPAGHEEAK | 5801 | 40.05 | 3 | 1978.8678 | 3 |  | 0 |  | yes | 1594.23 | 1092.56 | 1322.26 | 1132.18 |

  

| Tags | |
| --- | --- |

  

##

## P57776

EF1D\_MOUSE
Elongation factor 1-delta OS=Mus musculus GN=Eef1d PE=1 SV=3  
2
peptides

  

| Sequence | Peptide Ion | Score | Hits | Mass | Charge | Tags | Conflicts | Modifications | In quantitation | Average Normalised Abundances | | | |
| --- | --- | --- | --- | --- | --- | --- | --- | --- | --- | --- | --- | --- | --- |
|  |  |  |  |  |  |  |  |  |  | 2m2f | 4m2f | 2m4f | 4m4f |
| GVVQDLQQAISK | 4163 | 71.19 | 3 | 1284.6999 | 2 |  | 0 |  | yes | 1687.11 | 1232.43 | 1720.65 | 1765.09 |
| SIQLDGLVWGASK | 1362 | --- | --- | 1372.7420 | 3 |  | 0 |  | yes | 5470.86 | 6422.71 | 7436.63 | 9372.28 |
| SIQLDGLVWGASK | 3163 | 38.66 | 3 | 1372.7268 | 2 |  | 0 |  | yes | 4641.00 | 3834.26 | 5441.15 | 5326.67 |

  

| Tags | |
| --- | --- |

  

##

## P13020

GELS\_MOUSE
Gelsolin OS=Mus musculus GN=Gsn PE=1 SV=3  
2 peptides

  

| Sequence | Peptide Ion | Score | Hits | Mass | Charge | Tags | Conflicts | Modifications | In quantitation | Average Normalised Abundances | | | |
| --- | --- | --- | --- | --- | --- | --- | --- | --- | --- | --- | --- | --- | --- |
|  |  |  |  |  |  |  |  |  |  | 2m2f | 4m2f | 2m4f | 4m4f |
| EVQGFESSTFSGYFK | 8540 | 60.79 | 3 | 1711.7713 | 2 |  | 0 |  | yes | 1011.19 | 568.17 | 945.82 | 476.58 |
| TPSAAYLWVGAGASEAEK | 7442 | 48.44 | 3 | 1806.8772 | 2 |  | 0 |  | yes | 1587.50 | 895.22 | 1221.25 | 1002.06 |

  

| Tags | |
| --- | --- |

  

##

## P61255

RL26\_MOUSE
60S ribosomal protein L26 OS=Mus musculus GN=Rpl26 PE=2 SV=1  
3
peptides

  

| Sequence | Peptide Ion | Score | Hits | Mass | Charge | Tags | Conflicts | Modifications | In quantitation | Average Normalised Abundances | | | |
| --- | --- | --- | --- | --- | --- | --- | --- | --- | --- | --- | --- | --- | --- |
|  |  |  |  |  |  |  |  |  |  | 2m2f | 4m2f | 2m4f | 4m4f |
| FNPFVTSDR | 6293 | 34.68 | 3 | 1081.5200 | 2 |  | 0 |  | yes | 466.15 | 394.88 | 354.85 | 490.80 |
| HFNAPSHIR | 9678 | 41.53 | 1 | 1077.5475 | 3 |  | 0 |  | yes | 259.70 | 36.12 | 124.52 | 105.43 |
| KDDEVQVVR | 15230 | 31.45 | 2 | 1086.5666 | 2 |  | 0 |  | yes | 182.35 | 82.07 | 143.03 | 210.79 |
| KDDEVQVVR | 13788 | --- | --- | 1086.5667 | 3 |  | 0 |  | yes | 144.11 | 193.19 | 114.42 | 296.24 |

  

| Tags | |
| --- | --- |

  

##

## Q8CGC7

SYEP\_MOUSE
Bifunctional aminoacyl-tRNA synthetase OS=Mus musculus GN=Eprs PE=2
SV=3  
2 peptides

  

| Sequence | Peptide Ion | Score | Hits | Mass | Charge | Tags | Conflicts | Modifications | In quantitation | Average Normalised Abundances | | | |
| --- | --- | --- | --- | --- | --- | --- | --- | --- | --- | --- | --- | --- | --- |
|  |  |  |  |  |  |  |  |  |  | 2m2f | 4m2f | 2m4f | 4m4f |
| VTEAVECLLSLK | 11713 | 67.36 | 3 | 1360.7272 | 2 |  | 0 |  | yes | 314.04 | 198.87 | 360.32 | 223.60 |
| VYEELLAIPVVR | 10000 | 39.37 | 3 | 1399.8071 | 2 |  | 0 |  | yes | 555.91 | 368.30 | 585.34 | 537.66 |

  

| Tags | |
| --- | --- |

  

##

## Q9CZX8

RS19\_MOUSE
40S ribosomal protein S19 OS=Mus musculus GN=Rps19 PE=1 SV=3  
2
peptides

  

| Sequence | Peptide Ion | Score | Hits | Mass | Charge | Tags | Conflicts | Modifications | In quantitation | Average Normalised Abundances | | | |
| --- | --- | --- | --- | --- | --- | --- | --- | --- | --- | --- | --- | --- | --- |
|  |  |  |  |  |  |  |  |  |  | 2m2f | 4m2f | 2m4f | 4m4f |
| ELAPYDENWFYTR | 4593 | 60.34 | 3 | 1702.7631 | 2 |  | 0 |  | yes | 3223.40 | 2620.50 | 3527.06 | 2790.50 |
| VLQALEGLK | 6213 | 45.34 | 3 | 969.5846 | 2 |  | 0 |  | yes | 528.93 | 306.84 | 452.69 | 349.50 |

  

| Tags | |
| --- | --- |

  

##

## P40142

TKT\_MOUSE
Transketolase OS=Mus musculus GN=Tkt PE=1 SV=1  
2 peptides

  

| Sequence | Peptide Ion | Score | Hits | Mass | Charge | Tags | Conflicts | Modifications | In quantitation | Average Normalised Abundances | | | |
| --- | --- | --- | --- | --- | --- | --- | --- | --- | --- | --- | --- | --- | --- |
|  |  |  |  |  |  |  |  |  |  | 2m2f | 4m2f | 2m4f | 4m4f |
| LDNLVAIFDINR | 7667 | 78.51 | 3 | 1401.7607 | 2 |  | 0 |  | yes | 1163.50 | 590.83 | 1210.78 | 483.84 |
| TVPFCSTFAAFFTR | 18107 | 26.15 | 2 | 1650.7866 | 2 |  | 0 |  | yes | 439.36 | 76.81 | 392.79 | 194.93 |

  

| Tags | |
| --- | --- |

  

##

## P00687

AMY1\_MOUSE
Alpha-amylase 1 OS=Mus musculus GN=Amy1 PE=1 SV=1  
2 peptides

  

| Sequence | Peptide Ion | Score | Hits | Mass | Charge | Tags | Conflicts | Modifications | In quantitation | Average Normalised Abundances | | | |
| --- | --- | --- | --- | --- | --- | --- | --- | --- | --- | --- | --- | --- | --- |
|  |  |  |  |  |  |  |  |  |  | 2m2f | 4m2f | 2m4f | 4m4f |
| LSGLLDLALEK | 4288 | 72.76 | 3 | 1170.6853 | 2 |  | 0 |  | yes | 1393.20 | 2397.58 | 1392.43 | 1488.05 |
| NWGEGWGLMPSDR | 15871 | 31.53 | 1 | 1503.6599 | 2 |  | 0 |  | yes | 112.58 | 422.37 | 103.61 | 230.94 |

  

| Tags | |
| --- | --- |

  

##

## P48774

GSTM5\_MOUSE
Glutathione S-transferase Mu 5 OS=Mus musculus GN=Gstm5 PE=1 SV=1  
2
peptides

  

| Sequence | Peptide Ion | Score | Hits | Mass | Charge | Tags | Conflicts | Modifications | In quantitation | Average Normalised Abundances | | | |
| --- | --- | --- | --- | --- | --- | --- | --- | --- | --- | --- | --- | --- | --- |
|  |  |  |  |  |  |  |  |  |  | 2m2f | 4m2f | 2m4f | 4m4f |
| ITQSNAILR | 1147 | 73.35 | 3 | 1014.5813 | 2 |  | 1 |  | yes | 4469.47 | 4836.33 | 3815.89 | 4891.82 |
| VDIMENQIMDFR | 19747 | 30.84 | 1 | 1509.6953 | 2 |  | 0 |  | yes | 117.87 | 37.43 | 77.38 | 57.46 |

  

| Tags | |
| --- | --- |

  

##

## P14115

RL27A\_MOUSE
60S ribosomal protein L27a OS=Mus musculus GN=Rpl27a PE=2 SV=5  
2
peptides

  

| Sequence | Peptide Ion | Score | Hits | Mass | Charge | Tags | Conflicts | Modifications | In quantitation | Average Normalised Abundances | | | |
| --- | --- | --- | --- | --- | --- | --- | --- | --- | --- | --- | --- | --- | --- |
|  |  |  |  |  |  |  |  |  |  | 2m2f | 4m2f | 2m4f | 4m4f |
| NQSFCPTVNLDK | 4044 | 36.34 | 3 | 1421.6607 | 2 |  | 0 |  | yes | 2413.10 | 3083.99 | 2169.79 | 5205.72 |
| TGVAPIIDVVR | 1995 | 67.29 | 3 | 1138.6696 | 2 |  | 0 |  | yes | 2654.99 | 2721.71 | 2921.41 | 3383.93 |

  

| Tags | |
| --- | --- |

  

##

## P14869

RLA0\_MOUSE
60S acidic ribosomal protein P0 OS=Mus musculus GN=Rplp0 PE=1 SV=3  
2
peptides

  

| Sequence | Peptide Ion | Score | Hits | Mass | Charge | Tags | Conflicts | Modifications | In quantitation | Average Normalised Abundances | | | |
| --- | --- | --- | --- | --- | --- | --- | --- | --- | --- | --- | --- | --- | --- |
|  |  |  |  |  |  |  |  |  |  | 2m2f | 4m2f | 2m4f | 4m4f |
| AFLADPSAFAAAAPAAAATTAAPAAAAAPAK | 7516 | 34.28 | 2 | 2695.3936 | 3 |  | 0 |  | yes | 2053.16 | 264.15 | 1712.91 | 824.55 |
| GTIEILSDVQLIK | 3959 | 69.05 | 3 | 1427.8219 | 2 |  | 0 |  | yes | 4235.43 | 2057.70 | 3079.15 | 996.58 |

  

| Tags | |
| --- | --- |

  

##

## Q9D8V7

SC11C\_MOUSE
Signal peptidase complex catalytic subunit SEC11C OS=Mus musculus GN=Sec11c PE=2
SV=3  
2 peptides

  

| Sequence | Peptide Ion | Score | Hits | Mass | Charge | Tags | Conflicts | Modifications | In quantitation | Average Normalised Abundances | | | |
| --- | --- | --- | --- | --- | --- | --- | --- | --- | --- | --- | --- | --- | --- |
|  |  |  |  |  |  |  |  |  |  | 2m2f | 4m2f | 2m4f | 4m4f |
| AGAVGTHLPTSSLDIFGDLR | 11133 | 35.68 | 3 | 2026.0467 | 3 |  | 0 |  | yes | 600.61 | 159.42 | 396.99 | 207.17 |
| GDLLFLTNFR | 5869 | 67.23 | 3 | 1194.6391 | 2 |  | 0 |  | yes | 1091.43 | 582.26 | 1166.20 | 1029.12 |

  

| Tags | |
| --- | --- |

  

##

## P11087

CO1A1\_MOUSE
Collagen alpha-1(I) chain OS=Mus musculus GN=Col1a1 PE=1 SV=4  
1
peptide

  

| Sequence | Peptide Ion | Score | Hits | Mass | Charge | Tags | Conflicts | Modifications | In quantitation | Average Normalised Abundances | | | |
| --- | --- | --- | --- | --- | --- | --- | --- | --- | --- | --- | --- | --- | --- |
|  |  |  |  |  |  |  |  |  |  | 2m2f | 4m2f | 2m4f | 4m4f |
| GETGPAGPAGPIGPAGAR | 5772 | 102.90 | 3 | 1531.7734 | 2 |  | 0 |  | yes | 2096.20 | 778.01 | 1567.66 | 2361.68 |

  

| Tags | |
| --- | --- |

  

##

## P62717

RL18A\_MOUSE
60S ribosomal protein L18a OS=Mus musculus GN=Rpl18a PE=1 SV=1  
2
peptides

  

| Sequence | Peptide Ion | Score | Hits | Mass | Charge | Tags | Conflicts | Modifications | In quantitation | Average Normalised Abundances | | | |
| --- | --- | --- | --- | --- | --- | --- | --- | --- | --- | --- | --- | --- | --- |
|  |  |  |  |  |  |  |  |  |  | 2m2f | 4m2f | 2m4f | 4m4f |
| DLTTAGAVTQCYR | 4658 | 59.53 | 3 | 1454.6811 | 2 |  | 0 |  | yes | 1668.16 | 1353.17 | 1521.78 | 1380.51 |
| SSGEIVYCGQVFEK | 5207 | 42.71 | 3 | 1601.7392 | 2 |  | 0 |  | yes | 1973.07 | 1659.89 | 1833.31 | 1206.43 |

  

| Tags | |
| --- | --- |

  

##

## P15105

GLNA\_MOUSE
Glutamine synthetase OS=Mus musculus GN=Glul PE=1 SV=6  
2
peptides

  

| Sequence | Peptide Ion | Score | Hits | Mass | Charge | Tags | Conflicts | Modifications | In quantitation | Average Normalised Abundances | | | |
| --- | --- | --- | --- | --- | --- | --- | --- | --- | --- | --- | --- | --- | --- |
|  |  |  |  |  |  |  |  |  |  | 2m2f | 4m2f | 2m4f | 4m4f |
| LTGFHETSNINDFSAGVANR | 10398 | 50.01 | 1 | 2149.0218 | 3 |  | 0 |  | yes | 969.85 | 726.17 | 813.96 | 761.85 |
| TCLLNETGDEPFQYK | 9953 | 50.91 | 3 | 1813.8190 | 2 |  | 0 |  | yes | 753.80 | 710.87 | 749.50 | 670.06 |

  

| Tags | |
| --- | --- |

  

##

## P18242

CATD\_MOUSE
Cathepsin D OS=Mus musculus GN=Ctsd PE=1 SV=1  
3 peptides

  

| Sequence | Peptide Ion | Score | Hits | Mass | Charge | Tags | Conflicts | Modifications | In quantitation | Average Normalised Abundances | | | |
| --- | --- | --- | --- | --- | --- | --- | --- | --- | --- | --- | --- | --- | --- |
|  |  |  |  |  |  |  |  |  |  | 2m2f | 4m2f | 2m4f | 4m4f |
| GGCEAIVDTGTSLLVGPVEEVK | 13168 | --- | --- | 2229.1205 | 2 |  | 0 |  | yes | 1465.57 | 473.26 | 1128.53 | 466.38 |
| GGCEAIVDTGTSLLVGPVEEVK | 3272 | 29.08 | 3 | 2229.1296 | 3 |  | 0 |  | yes | 9917.94 | 5194.35 | 9961.84 | 3507.13 |
| NIFSFYLNR | 4129 | 33.35 | 3 | 1172.5976 | 2 |  | 0 |  | yes | 2181.82 | 1231.44 | 2100.19 | 1477.21 |
| VSSLPTVYLK | 1864 | 38.29 | 3 | 1105.6315 | 2 |  | 0 |  | yes | 2501.68 | 3272.89 | 3237.48 | 2501.66 |

  

| Tags | |
| --- | --- |

  

##

## Q9CPU0

LGUL\_MOUSE
Lactoylglutathione lyase OS=Mus musculus GN=Glo1 PE=1 SV=3  
2
peptides

  

| Sequence | Peptide Ion | Score | Hits | Mass | Charge | Tags | Conflicts | Modifications | In quantitation | Average Normalised Abundances | | | |
| --- | --- | --- | --- | --- | --- | --- | --- | --- | --- | --- | --- | --- | --- |
|  |  |  |  |  |  |  |  |  |  | 2m2f | 4m2f | 2m4f | 4m4f |
| DFLLQQTMLR | 7095 | 56.53 | 3 | 1263.6642 | 2 |  | 0 |  | yes | 1103.73 | 799.82 | 763.08 | 521.15 |
| VLGLTLLQK | 4357 | 43.49 | 3 | 983.6376 | 2 |  | 0 |  | yes | 879.81 | 645.61 | 674.26 | 563.39 |

  

| Tags | |
| --- | --- |

  

##

## Q9CR57

RL14\_MOUSE
60S ribosomal protein L14 OS=Mus musculus GN=Rpl14 PE=2 SV=3  
1
peptide

  

| Sequence | Peptide Ion | Score | Hits | Mass | Charge | Tags | Conflicts | Modifications | In quantitation | Average Normalised Abundances | | | |
| --- | --- | --- | --- | --- | --- | --- | --- | --- | --- | --- | --- | --- | --- |
|  |  |  |  |  |  |  |  |  |  | 2m2f | 4m2f | 2m4f | 4m4f |
| LVAIVDVIDQNR | 2530 | 99.94 | 3 | 1353.7610 | 2 |  | 0 |  | yes | 3646.51 | 3017.74 | 4439.28 | 2406.34 |

  

| Tags | |
| --- | --- |

  

##

## P63028

TCTP\_MOUSE
Translationally-controlled tumor protein OS=Mus musculus GN=Tpt1 PE=1
SV=1  
2 peptides

  

| Sequence | Peptide Ion | Score | Hits | Mass | Charge | Tags | Conflicts | Modifications | In quantitation | Average Normalised Abundances | | | |
| --- | --- | --- | --- | --- | --- | --- | --- | --- | --- | --- | --- | --- | --- |
|  |  |  |  |  |  |  |  |  |  | 2m2f | 4m2f | 2m4f | 4m4f |
| DLISHDELFSDIYK | 18183 | --- | --- | 1693.8219 | 2 |  | 0 |  | yes | 370.34 | 109.31 | 184.98 | 217.89 |
| DLISHDELFSDIYK | 6347 | 31.51 | 3 | 1693.8190 | 3 |  | 0 |  | yes | 976.93 | 389.11 | 886.21 | 700.53 |
| EIADGLCLEVEGK | 5741 | 67.25 | 3 | 1431.6904 | 2 |  | 0 |  | yes | 1410.25 | 854.49 | 911.12 | 356.01 |

  

| Tags | |
| --- | --- |

  

##

## P61027

RAB10\_MOUSE
Ras-related protein Rab-10 OS=Mus musculus GN=Rab10 PE=1 SV=1  
2
peptides

  

| Sequence | Peptide Ion | Score | Hits | Mass | Charge | Tags | Conflicts | Modifications | In quantitation | Average Normalised Abundances | | | |
| --- | --- | --- | --- | --- | --- | --- | --- | --- | --- | --- | --- | --- | --- |
|  |  |  |  |  |  |  |  |  |  | 2m2f | 4m2f | 2m4f | 4m4f |
| AFLTLAEDILR | 10301 | 54.45 | 3 | 1260.7072 | 2 |  | 0 |  | yes | 741.52 | 176.40 | 651.29 | 285.97 |
| LLLIGDSGVGK | 2022 | 43.69 | 3 | 1070.6324 | 2 |  | 2 |  | yes | 2121.19 | 2152.22 | 1924.28 | 2150.47 |

  

| Tags | |
| --- | --- |

  

##

## P99029

PRDX5\_MOUSE
Peroxiredoxin-5, mitochondrial OS=Mus musculus GN=Prdx5 PE=1 SV=2  
2
peptides

  

| Sequence | Peptide Ion | Score | Hits | Mass | Charge | Tags | Conflicts | Modifications | In quantitation | Average Normalised Abundances | | | |
| --- | --- | --- | --- | --- | --- | --- | --- | --- | --- | --- | --- | --- | --- |
|  |  |  |  |  |  |  |  |  |  | 2m2f | 4m2f | 2m4f | 4m4f |
| ATDLLLDDSLVSLFGNR | 18154 | 25.95 | 2 | 1847.9615 | 3 |  | 0 |  | yes | 425.06 | 99.94 | 210.06 | 60.74 |
| ATDLLLDDSLVSLFGNR | 11199 | 34.18 | 3 | 1847.9642 | 2 |  | 0 |  | yes | 2093.85 | 367.53 | 1086.23 | 407.64 |
| GVLFGVPGAFTPGCSK | 10481 | 33.08 | 3 | 1592.8023 | 2 |  | 0 |  | yes | 1065.28 | 331.72 | 674.76 | 675.29 |

  

| Tags | |
| --- | --- |

  

##

## Q62186

SSRD\_MOUSE
Translocon-associated protein subunit delta OS=Mus musculus GN=Ssr4 PE=2
SV=1  
2 peptides

  

| Sequence | Peptide Ion | Score | Hits | Mass | Charge | Tags | Conflicts | Modifications | In quantitation | Average Normalised Abundances | | | |
| --- | --- | --- | --- | --- | --- | --- | --- | --- | --- | --- | --- | --- | --- |
|  |  |  |  |  |  |  |  |  |  | 2m2f | 4m2f | 2m4f | 4m4f |
| FFDEESYSLLR | 3760 | 63.04 | 3 | 1404.6665 | 2 |  | 0 |  | yes | 2932.77 | 2814.27 | 2968.27 | 2717.28 |
| NNEDVSIIPPLFTVSVDHR | 6795 | 30.12 | 3 | 2151.0956 | 3 |  | 0 |  | yes | 2848.59 | 619.73 | 2469.88 | 1306.14 |

  

| Tags | |
| --- | --- |

  

##

## Q921F2

TADBP\_MOUSE
TAR DNA-binding protein 43 OS=Mus musculus GN=Tardbp PE=1 SV=1  
2
peptides

  

| Sequence | Peptide Ion | Score | Hits | Mass | Charge | Tags | Conflicts | Modifications | In quantitation | Average Normalised Abundances | | | |
| --- | --- | --- | --- | --- | --- | --- | --- | --- | --- | --- | --- | --- | --- |
|  |  |  |  |  |  |  |  |  |  | 2m2f | 4m2f | 2m4f | 4m4f |
| AFAFVTFADDK | 17637 | 37.73 | 3 | 1230.5944 | 2 |  | 0 |  | yes | 85.45 | 92.10 | 113.64 | 105.42 |
| TSDLIVLGLPWK | 13950 | 53.94 | 3 | 1340.7692 | 2 |  | 0 |  | yes | 479.43 | 67.86 | 387.74 | 244.63 |

  

| Tags | |
| --- | --- |

  

##

## P47962

RL5\_MOUSE
60S ribosomal protein L5 OS=Mus musculus GN=Rpl5 PE=1 SV=3  
1
peptide

  

| Sequence | Peptide Ion | Score | Hits | Mass | Charge | Tags | Conflicts | Modifications | In quantitation | Average Normalised Abundances | | | |
| --- | --- | --- | --- | --- | --- | --- | --- | --- | --- | --- | --- | --- | --- |
|  |  |  |  |  |  |  |  |  |  | 2m2f | 4m2f | 2m4f | 4m4f |
| VGLTNYAAAYCTGLLLAR | 11553 | 44.93 | 3 | 1926.0043 | 2 |  | 0 |  | yes | 1007.12 | 260.60 | 771.54 | 455.41 |
| VGLTNYAAAYCTGLLLAR | 10704 | 46.59 | 3 | 1926.0018 | 3 |  | 0 |  | yes | 773.38 | 320.01 | 628.56 | 312.27 |

  

| Tags | |
| --- | --- |

  

##

## Q9CYN2

SPCS2\_MOUSE
Signal peptidase complex subunit 2 OS=Mus musculus GN=Spcs2 PE=2 SV=1  
2
peptides

  

| Sequence | Peptide Ion | Score | Hits | Mass | Charge | Tags | Conflicts | Modifications | In quantitation | Average Normalised Abundances | | | |
| --- | --- | --- | --- | --- | --- | --- | --- | --- | --- | --- | --- | --- | --- |
|  |  |  |  |  |  |  |  |  |  | 2m2f | 4m2f | 2m4f | 4m4f |
| SGGGGGSSGAGGGPSCGTSSSR | 21382 | 36.94 | 1 | 1782.7162 | 2 |  | 0 |  | yes | 642.45 | 353.87 | 360.68 | 413.69 |
| YVENFGLIDGR | 3974 | 54.28 | 3 | 1281.6340 | 2 |  | 0 |  | yes | 1351.58 | 1542.83 | 1433.80 | 1503.92 |

  

| Tags | |
| --- | --- |

  

##

## P42125

D3D2\_MOUSE
3,2-trans-enoyl-CoA isomerase, mitochondrial OS=Mus musculus GN=Dci PE=2
SV=1  
2 peptides

  

| Sequence | Peptide Ion | Score | Hits | Mass | Charge | Tags | Conflicts | Modifications | In quantitation | Average Normalised Abundances | | | |
| --- | --- | --- | --- | --- | --- | --- | --- | --- | --- | --- | --- | --- | --- |
|  |  |  |  |  |  |  |  |  |  | 2m2f | 4m2f | 2m4f | 4m4f |
| EADIQNFTSFISK | 10696 | 43.86 | 3 | 1498.7309 | 2 |  | 0 |  | yes | 508.01 | 430.13 | 573.40 | 385.58 |
| VGVVDEVVPEDQVHSK | 12046 | 47.05 | 1 | 1734.8791 | 3 |  | 0 |  | yes | 356.16 | 146.79 | 250.63 | 327.99 |

  

| Tags | |
| --- | --- |

  

##

## P18760

COF1\_MOUSE
Cofilin-1 OS=Mus musculus GN=Cfl1 PE=1 SV=3  
2 peptides

  

| Sequence | Peptide Ion | Score | Hits | Mass | Charge | Tags | Conflicts | Modifications | In quantitation | Average Normalised Abundances | | | |
| --- | --- | --- | --- | --- | --- | --- | --- | --- | --- | --- | --- | --- | --- |
|  |  |  |  |  |  |  |  |  |  | 2m2f | 4m2f | 2m4f | 4m4f |
| EILVGDVGQTVDDPYTTFVK | 7706 | 39.85 | 1 | 2195.1015 | 2 |  | 0 |  | yes | 1684.61 | 1474.14 | 1809.99 | 1323.82 |
| EILVGDVGQTVDDPYTTFVK | 4818 | --- | --- | 2195.1004 | 3 |  | 0 |  | yes | 2224.65 | 2088.67 | 2757.27 | 1733.49 |
| YALYDATYETK | 2876 | 50.79 | 3 | 1336.6204 | 2 |  | 0 |  | yes | 3063.14 | 2400.23 | 2507.66 | 2935.77 |

  

| Tags | |
| --- | --- |

  

##

## O55029

COPB2\_MOUSE
Coatomer subunit beta' OS=Mus musculus GN=Copb2 PE=2 SV=2  
2
peptides

  

| Sequence | Peptide Ion | Score | Hits | Mass | Charge | Tags | Conflicts | Modifications | In quantitation | Average Normalised Abundances | | | |
| --- | --- | --- | --- | --- | --- | --- | --- | --- | --- | --- | --- | --- | --- |
|  |  |  |  |  |  |  |  |  |  | 2m2f | 4m2f | 2m4f | 4m4f |
| EAFVVEEWVK | 7952 | 32.86 | 3 | 1234.6232 | 2 |  | 0 |  | yes | 664.90 | 708.90 | 740.24 | 470.87 |
| FELALQLGELK | 15276 | 55.73 | 3 | 1259.7040 | 2 |  | 0 |  | yes | 783.92 | 223.36 | 333.31 | 314.79 |

  

| Tags | |
| --- | --- |

  

##

## Q8VDN2

AT1A1\_MOUSE
Sodium/potassium-transporting ATPase subunit alpha-1 OS=Mus musculus GN=Atp1a1
PE=1 SV=1  
2 peptides

  

| Sequence | Peptide Ion | Score | Hits | Mass | Charge | Tags | Conflicts | Modifications | In quantitation | Average Normalised Abundances | | | |
| --- | --- | --- | --- | --- | --- | --- | --- | --- | --- | --- | --- | --- | --- |
|  |  |  |  |  |  |  |  |  |  | 2m2f | 4m2f | 2m4f | 4m4f |
| AVAGDASESALLK | 10679 | 54.87 | 3 | 1230.6411 | 2 |  | 0 |  | yes | 519.73 | 179.45 | 317.50 | 189.43 |
| SPDFTNENPLETR | 11812 | 33.21 | 1 | 1518.6940 | 2 |  | 0 |  | yes | 457.47 | 153.46 | 406.42 | 225.12 |

  

| Tags | |
| --- | --- |

  

##

## Q8R086

SUOX\_MOUSE
Sulfite oxidase, mitochondrial OS=Mus musculus GN=Suox PE=1 SV=2  
2
peptides

  

| Sequence | Peptide Ion | Score | Hits | Mass | Charge | Tags | Conflicts | Modifications | In quantitation | Average Normalised Abundances | | | |
| --- | --- | --- | --- | --- | --- | --- | --- | --- | --- | --- | --- | --- | --- |
|  |  |  |  |  |  |  |  |  |  | 2m2f | 4m2f | 2m4f | 4m4f |
| TGVWVTLGSEVFDVTK | 12812 | 49.17 | 3 | 1736.8977 | 2 |  | 0 |  | yes | 905.46 | 299.63 | 773.71 | 187.12 |
| VSVESEESYSHWQR | 7223 | 38.01 | 3 | 1721.7649 | 3 |  | 0 |  | yes | 605.17 | 324.77 | 590.00 | 410.99 |

  

| Tags | |
| --- | --- |

  

##

## Q9QYB1

CLIC4\_MOUSE
Chloride intracellular channel protein 4 OS=Mus musculus GN=Clic4 PE=1
SV=3  
1 peptide

  

| Sequence | Peptide Ion | Score | Hits | Mass | Charge | Tags | Conflicts | Modifications | In quantitation | Average Normalised Abundances | | | |
| --- | --- | --- | --- | --- | --- | --- | --- | --- | --- | --- | --- | --- | --- |
|  |  |  |  |  |  |  |  |  |  | 2m2f | 4m2f | 2m4f | 4m4f |
| GVVFSVTTVDLK | 17044 | 85.98 | 3 | 1263.7050 | 2 |  | 0 |  | yes | 261.19 | 260.41 | 332.53 | 175.34 |

  

| Tags | |
| --- | --- |

  

##

## P15327

PMGE\_MOUSE
Bisphosphoglycerate mutase OS=Mus musculus GN=Bpgm PE=2 SV=2  
2
peptides

  

| Sequence | Peptide Ion | Score | Hits | Mass | Charge | Tags | Conflicts | Modifications | In quantitation | Average Normalised Abundances | | | |
| --- | --- | --- | --- | --- | --- | --- | --- | --- | --- | --- | --- | --- | --- |
|  |  |  |  |  |  |  |  |  |  | 2m2f | 4m2f | 2m4f | 4m4f |
| AVGPHQFLGNQEAIQAAIK | 5262 | 39.82 | 2 | 1991.0546 | 3 |  | 0 |  | yes | 2858.69 | 1353.89 | 2950.86 | 1481.76 |
| FCSWVDQK | 5035 | 45.03 | 3 | 1068.4708 | 2 |  | 0 |  | yes | 1213.43 | 1449.61 | 1242.35 | 1272.18 |

  

| Tags | |
| --- | --- |

  

##

## Q64310

SURF4\_MOUSE
Surfeit locus protein 4 OS=Mus musculus GN=Surf4 PE=2 SV=1  
1
peptide

  

| Sequence | Peptide Ion | Score | Hits | Mass | Charge | Tags | Conflicts | Modifications | In quantitation | Average Normalised Abundances | | | |
| --- | --- | --- | --- | --- | --- | --- | --- | --- | --- | --- | --- | --- | --- |
|  |  |  |  |  |  |  |  |  |  | 2m2f | 4m2f | 2m4f | 4m4f |
| LCLISTFLEDGIR | 18517 | 28.42 | 3 | 1535.8012 | 3 |  | 0 |  | yes | 198.97 | 58.59 | 182.56 | 68.03 |
| LCLISTFLEDGIR | 6326 | 56.16 | 3 | 1535.8014 | 2 |  | 0 |  | yes | 3194.68 | 1192.49 | 2988.93 | 1596.25 |

  

| Tags | |
| --- | --- |

  

##

## Q91V61

SFXN3\_MOUSE
Sideroflexin-3 OS=Mus musculus GN=Sfxn3 PE=1 SV=1  
2 peptides

  

| Sequence | Peptide Ion | Score | Hits | Mass | Charge | Tags | Conflicts | Modifications | In quantitation | Average Normalised Abundances | | | |
| --- | --- | --- | --- | --- | --- | --- | --- | --- | --- | --- | --- | --- | --- |
|  |  |  |  |  |  |  |  |  |  | 2m2f | 4m2f | 2m4f | 4m4f |
| AGVATPGLTEDQLWR | 8175 | 39.64 | 3 | 1612.8181 | 2 |  | 0 |  | yes | 1168.27 | 852.22 | 1029.55 | 1080.67 |
| NLLLSGEQLEASR | 5704 | 44.45 | 3 | 1428.7567 | 2 |  | 0 |  | yes | 1418.07 | 1694.09 | 1662.23 | 1478.41 |

  

| Tags | |
| --- | --- |

  

##

## P26040

EZRI\_MOUSE
Ezrin OS=Mus musculus GN=Ezr PE=1 SV=3  
2 peptides

  

| Sequence | Peptide Ion | Score | Hits | Mass | Charge | Tags | Conflicts | Modifications | In quantitation | Average Normalised Abundances | | | |
| --- | --- | --- | --- | --- | --- | --- | --- | --- | --- | --- | --- | --- | --- |
|  |  |  |  |  |  |  |  |  |  | 2m2f | 4m2f | 2m4f | 4m4f |
| IGFPWSEIR | 4648 | 41.26 | 3 | 1103.5760 | 2 |  | 0 |  | yes | 1302.06 | 885.71 | 1171.50 | 1153.60 |
| LFFLQVK | 3675 | 41.50 | 2 | 893.5372 | 2 |  | 0 |  | yes | 1097.12 | 937.88 | 1110.43 | 1171.98 |

  

| Tags | |
| --- | --- |

  

##

## Q9D8N0

EF1G\_MOUSE
Elongation factor 1-gamma OS=Mus musculus GN=Eef1g PE=1 SV=3  
1
peptide

  

| Sequence | Peptide Ion | Score | Hits | Mass | Charge | Tags | Conflicts | Modifications | In quantitation | Average Normalised Abundances | | | |
| --- | --- | --- | --- | --- | --- | --- | --- | --- | --- | --- | --- | --- | --- |
|  |  |  |  |  |  |  |  |  |  | 2m2f | 4m2f | 2m4f | 4m4f |
| ALIAAQYSGAQVR | 4402 | 82.42 | 3 | 1346.7299 | 2 |  | 0 |  | yes | 1606.99 | 1875.71 | 1405.61 | 2302.53 |

  

| Tags | |
| --- | --- |

  

##

## P62301

RS13\_MOUSE
40S ribosomal protein S13 OS=Mus musculus GN=Rps13 PE=1 SV=2  
2
peptides

  

| Sequence | Peptide Ion | Score | Hits | Mass | Charge | Tags | Conflicts | Modifications | In quantitation | Average Normalised Abundances | | | |
| --- | --- | --- | --- | --- | --- | --- | --- | --- | --- | --- | --- | --- | --- |
|  |  |  |  |  |  |  |  |  |  | 2m2f | 4m2f | 2m4f | 4m4f |
| GLTPSQIGVILR | 6514 | 43.29 | 3 | 1252.7495 | 2 |  | 0 |  | yes | 650.26 | 379.57 | 531.92 | 502.95 |
| LILIESR | 2773 | 38.82 | 3 | 842.5226 | 2 |  | 0 |  | yes | 1151.51 | 1105.13 | 1077.52 | 1259.53 |

  

| Tags | |
| --- | --- |

  

##

## Q9CYH2

CJ058\_MOUSE
UPF0765 protein C10orf58 homolog OS=Mus musculus PE=1 SV=2  
2
peptides

  

| Sequence | Peptide Ion | Score | Hits | Mass | Charge | Tags | Conflicts | Modifications | In quantitation | Average Normalised Abundances | | | |
| --- | --- | --- | --- | --- | --- | --- | --- | --- | --- | --- | --- | --- | --- |
|  |  |  |  |  |  |  |  |  |  | 2m2f | 4m2f | 2m4f | 4m4f |
| LDELGVPLYAVVK | 6468 | 54.63 | 3 | 1414.8054 | 2 |  | 0 |  | yes | 1735.28 | 1069.09 | 895.72 | 563.76 |
| LGVWYNSFR | 22739 | 27.42 | 2 | 1140.5672 | 2 |  | 0 |  | yes | 117.96 | 81.06 | 52.27 | 45.34 |

  

| Tags | |
| --- | --- |

  

##

## P62858

RS28\_MOUSE
40S ribosomal protein S28 OS=Mus musculus GN=Rps28 PE=2 SV=1  
1
peptide

  

| Sequence | Peptide Ion | Score | Hits | Mass | Charge | Tags | Conflicts | Modifications | In quantitation | Average Normalised Abundances | | | |
| --- | --- | --- | --- | --- | --- | --- | --- | --- | --- | --- | --- | --- | --- |
|  |  |  |  |  |  |  |  |  |  | 2m2f | 4m2f | 2m4f | 4m4f |
| EGDVLTLLESER | 2139 | 80.16 | 3 | 1359.6872 | 2 |  | 0 |  | yes | 5362.69 | 4503.51 | 4991.21 | 3785.22 |

  

| Tags | |
| --- | --- |

  

##

## P62267

RS23\_MOUSE
40S ribosomal protein S23 OS=Mus musculus GN=Rps23 PE=2 SV=3  
1
peptide

  

| Sequence | Peptide Ion | Score | Hits | Mass | Charge | Tags | Conflicts | Modifications | In quantitation | Average Normalised Abundances | | | |
| --- | --- | --- | --- | --- | --- | --- | --- | --- | --- | --- | --- | --- | --- |
|  |  |  |  |  |  |  |  |  |  | 2m2f | 4m2f | 2m4f | 4m4f |
| VANVSLLALYK | 3041 | 79.62 | 3 | 1189.7021 | 2 |  | 0 |  | yes | 3098.69 | 4762.69 | 3717.96 | 4007.35 |

  

| Tags | |
| --- | --- |

  

##

## Q91YW3

DNJC3\_MOUSE
DnaJ homolog subfamily C member 3 OS=Mus musculus GN=Dnajc3 PE=1 SV=1  
2
peptides

  

| Sequence | Peptide Ion | Score | Hits | Mass | Charge | Tags | Conflicts | Modifications | In quantitation | Average Normalised Abundances | | | |
| --- | --- | --- | --- | --- | --- | --- | --- | --- | --- | --- | --- | --- | --- |
|  |  |  |  |  |  |  |  |  |  | 2m2f | 4m2f | 2m4f | 4m4f |
| ILEVCVWDAELR | 16625 | 28.20 | 1 | 1501.7595 | 2 |  | 0 |  | yes | 153.45 | 121.01 | 149.98 | 119.40 |
| LIESAEELIR | 6057 | 50.18 | 1 | 1171.6483 | 2 |  | 1 |  | no | 1107.55 | 1263.05 | 955.12 | 1045.99 |

  

| Tags | |
| --- | --- |

  

##

## Q9DB20

ATPO\_MOUSE
ATP synthase subunit O, mitochondrial OS=Mus musculus GN=Atp5o PE=1
SV=1  
2 peptides

  

| Sequence | Peptide Ion | Score | Hits | Mass | Charge | Tags | Conflicts | Modifications | In quantitation | Average Normalised Abundances | | | |
| --- | --- | --- | --- | --- | --- | --- | --- | --- | --- | --- | --- | --- | --- |
|  |  |  |  |  |  |  |  |  |  | 2m2f | 4m2f | 2m4f | 4m4f |
| GEVPCTVTTASPLDDAVLSELK | 8069 | 29.47 | 2 | 2301.1411 | 3 |  | 0 |  | yes | 1570.86 | 764.86 | 1247.29 | 1002.79 |
| VSLAVLNPYIK | 7128 | 48.48 | 2 | 1215.7221 | 2 |  | 0 |  | yes | 753.06 | 448.35 | 743.15 | 705.81 |

  

| Tags | |
| --- | --- |

  

##

## O88569

ROA2\_MOUSE
Heterogeneous nuclear ribonucleoproteins A2/B1 OS=Mus musculus GN=Hnrnpa2b1 PE=1
SV=2  
1 peptide

  

| Sequence | Peptide Ion | Score | Hits | Mass | Charge | Tags | Conflicts | Modifications | In quantitation | Average Normalised Abundances | | | |
| --- | --- | --- | --- | --- | --- | --- | --- | --- | --- | --- | --- | --- | --- |
|  |  |  |  |  |  |  |  |  |  | 2m2f | 4m2f | 2m4f | 4m4f |
| LFIGGLSFETTEESLR | 11168 | 77.88 | 3 | 1797.9154 | 2 |  | 0 |  | yes | 1512.83 | 400.88 | 1170.66 | 340.27 |

  

| Tags | |
| --- | --- |

  

##

## Q9D0I9

SYRC\_MOUSE
Arginyl-tRNA synthetase, cytoplasmic OS=Mus musculus GN=Rars PE=2
SV=2  
2 peptides

  

| Sequence | Peptide Ion | Score | Hits | Mass | Charge | Tags | Conflicts | Modifications | In quantitation | Average Normalised Abundances | | | |
| --- | --- | --- | --- | --- | --- | --- | --- | --- | --- | --- | --- | --- | --- |
|  |  |  |  |  |  |  |  |  |  | 2m2f | 4m2f | 2m4f | 4m4f |
| DIVKEFEDK | 438 | 26.64 | 3 | 1121.5705 | 2 |  | 0 |  | yes | 2.69e+004 | 3.40e+004 | 3.27e+004 | 3.48e+004 |
| LNDYIFSFDK | 10680 | 50.55 | 3 | 1260.6016 | 2 |  | 0 |  | yes | 296.58 | 225.58 | 304.62 | 211.38 |

  

| Tags | |
| --- | --- |

  

##

## P60867

RS20\_MOUSE
40S ribosomal protein S20 OS=Mus musculus GN=Rps20 PE=1 SV=1  
2
peptides

  

| Sequence | Peptide Ion | Score | Hits | Mass | Charge | Tags | Conflicts | Modifications | In quantitation | Average Normalised Abundances | | | |
| --- | --- | --- | --- | --- | --- | --- | --- | --- | --- | --- | --- | --- | --- |
|  |  |  |  |  |  |  |  |  |  | 2m2f | 4m2f | 2m4f | 4m4f |
| LIDLHSPSEIVK | 14274 | --- | --- | 1349.7517 | 2 |  | 0 |  | yes | 235.08 | 158.67 | 147.54 | 166.33 |
| LIDLHSPSEIVK | 3682 | 26.80 | 3 | 1349.7551 | 3 |  | 0 |  | yes | 1024.45 | 695.67 | 868.70 | 1109.54 |
| TPVEPEVAIHR | 4445 | 50.37 | 3 | 1246.6669 | 3 |  | 0 |  | yes | 651.34 | 379.78 | 499.40 | 492.08 |

  

| Tags | |
| --- | --- |

  

##

## Q99MN1

SYK\_MOUSE
Lysyl-tRNA synthetase OS=Mus musculus GN=Kars PE=1 SV=1  
2
peptides

  

| Sequence | Peptide Ion | Score | Hits | Mass | Charge | Tags | Conflicts | Modifications | In quantitation | Average Normalised Abundances | | | |
| --- | --- | --- | --- | --- | --- | --- | --- | --- | --- | --- | --- | --- | --- |
|  |  |  |  |  |  |  |  |  |  | 2m2f | 4m2f | 2m4f | 4m4f |
| LIFYDLR | 11247 | 38.51 | 3 | 938.5215 | 2 |  | 0 |  | yes | 160.17 | 149.65 | 211.70 | 134.29 |
| YLDLILNDFVR | 13930 | 37.94 | 3 | 1379.7410 | 2 |  | 0 |  | yes | 575.76 | 284.30 | 513.03 | 274.87 |

  

| Tags | |
| --- | --- |

  

##

## Q99LX0

PARK7\_MOUSE
Protein DJ-1 OS=Mus musculus GN=Park7 PE=1 SV=1  
2 peptides

  

| Sequence | Peptide Ion | Score | Hits | Mass | Charge | Tags | Conflicts | Modifications | In quantitation | Average Normalised Abundances | | | |
| --- | --- | --- | --- | --- | --- | --- | --- | --- | --- | --- | --- | --- | --- |
|  |  |  |  |  |  |  |  |  |  | 2m2f | 4m2f | 2m4f | 4m4f |
| DGLILTSR | 5935 | 45.87 | 3 | 873.4916 | 2 |  | 0 |  | yes | 452.79 | 397.87 | 390.44 | 416.93 |
| GAEEMETVIPVDVMR | 9828 | 30.26 | 1 | 1674.7952 | 2 |  | 0 |  | yes | 1057.47 | 918.71 | 917.62 | 871.95 |

  

| Tags | |
| --- | --- |

  

##

## P68433

H31\_MOUSE
Histone H3.1 OS=Mus musculus GN=Hist1h3a PE=1 SV=2  
2
peptides

  

| Sequence | Peptide Ion | Score | Hits | Mass | Charge | Tags | Conflicts | Modifications | In quantitation | Average Normalised Abundances | | | |
| --- | --- | --- | --- | --- | --- | --- | --- | --- | --- | --- | --- | --- | --- |
|  |  |  |  |  |  |  |  |  |  | 2m2f | 4m2f | 2m4f | 4m4f |
| STELLIR | 314 | 45.32 | 3 | 830.4851 | 2 |  | 0 |  | yes | 1.48e+004 | 1.65e+004 | 1.40e+004 | 1.53e+004 |
| YRPGTVALR | 1220 | 30.37 | 3 | 1031.5868 | 2 |  | 0 |  | yes | 3962.50 | 2268.32 | 2546.38 | 2740.98 |

  

| Tags | |
| --- | --- |

  

##

## O55143

AT2A2\_MOUSE
Sarcoplasmic/endoplasmic reticulum calcium ATPase 2 OS=Mus musculus GN=Atp2a2
PE=1 SV=2  
2 peptides

  

| Sequence | Peptide Ion | Score | Hits | Mass | Charge | Tags | Conflicts | Modifications | In quantitation | Average Normalised Abundances | | | |
| --- | --- | --- | --- | --- | --- | --- | --- | --- | --- | --- | --- | --- | --- |
|  |  |  |  |  |  |  |  |  |  | 2m2f | 4m2f | 2m4f | 4m4f |
| ISLPVILMDETLK | 16883 | 25.73 | 3 | 1470.8360 | 2 |  | 0 |  | yes | 600.79 | 51.24 | 618.52 | 138.43 |
| VGEATETALTCLVEK | 11202 | 49.75 | 3 | 1619.8085 | 2 |  | 0 |  | yes | 528.66 | 139.30 | 432.13 | 290.84 |

  

| Tags | |
| --- | --- |

  

##

## Q9CZU6

CISY\_MOUSE
Citrate synthase, mitochondrial OS=Mus musculus GN=Cs PE=1 SV=1  
2
peptides

  

| Sequence | Peptide Ion | Score | Hits | Mass | Charge | Tags | Conflicts | Modifications | In quantitation | Average Normalised Abundances | | | |
| --- | --- | --- | --- | --- | --- | --- | --- | --- | --- | --- | --- | --- | --- |
|  |  |  |  |  |  |  |  |  |  | 2m2f | 4m2f | 2m4f | 4m4f |
| EGSSIGAIDSR | 8356 | 42.63 | 2 | 1090.5267 | 2 |  | 0 |  | yes | 444.72 | 261.71 | 268.45 | 246.87 |
| GLVYETSVLDPDEGIR | 6657 | 32.76 | 3 | 1761.8799 | 2 |  | 0 |  | yes | 1185.11 | 1006.20 | 1256.53 | 922.65 |

  

| Tags | |
| --- | --- |

  

##

## Q61735

CD47\_MOUSE
Leukocyte surface antigen CD47 OS=Mus musculus GN=Cd47 PE=1 SV=2  
1
peptide

  

| Sequence | Peptide Ion | Score | Hits | Mass | Charge | Tags | Conflicts | Modifications | In quantitation | Average Normalised Abundances | | | |
| --- | --- | --- | --- | --- | --- | --- | --- | --- | --- | --- | --- | --- | --- |
|  |  |  |  |  |  |  |  |  |  | 2m2f | 4m2f | 2m4f | 4m4f |
| ISVSDLINGIASLK | 12968 | 75.35 | 3 | 1428.8182 | 2 |  | 0 |  | yes | 830.71 | 80.95 | 556.32 | 193.92 |

  

| Tags | |
| --- | --- |

  

##

## P61804

DAD1\_MOUSE
Dolichyl-diphosphooligosaccharide--protein glycosyltransferase subunit DAD1
OS=Mus musculus GN=Dad1 PE=2 SV=3  
1 peptide

  

| Sequence | Peptide Ion | Score | Hits | Mass | Charge | Tags | Conflicts | Modifications | In quantitation | Average Normalised Abundances | | | |
| --- | --- | --- | --- | --- | --- | --- | --- | --- | --- | --- | --- | --- | --- |
|  |  |  |  |  |  |  |  |  |  | 2m2f | 4m2f | 2m4f | 4m4f |
| FLEEYLSSTPQR | 12034 | --- | --- | 1468.7229 | 3 |  | 0 |  | yes | 264.54 | 226.73 | 307.62 | 221.66 |
| FLEEYLSSTPQR | 4448 | 75.18 | 3 | 1468.7201 | 2 |  | 0 |  | yes | 2529.23 | 2739.70 | 2667.12 | 2370.12 |

  

| Tags | |
| --- | --- |

  

##

## P12970

RL7A\_MOUSE
60S ribosomal protein L7a OS=Mus musculus GN=Rpl7a PE=2 SV=2  
2
peptides

  

| Sequence | Peptide Ion | Score | Hits | Mass | Charge | Tags | Conflicts | Modifications | In quantitation | Average Normalised Abundances | | | |
| --- | --- | --- | --- | --- | --- | --- | --- | --- | --- | --- | --- | --- | --- |
|  |  |  |  |  |  |  |  |  |  | 2m2f | 4m2f | 2m4f | 4m4f |
| LKVPPAINQFTQALDR | 14404 | 26.47 | 3 | 1810.0100 | 3 |  | 0 |  | yes | 206.77 | 25.95 | 176.65 | 170.92 |
| NFGIGQDIQPK | 3743 | 47.79 | 3 | 1215.6237 | 2 |  | 0 |  | yes | 1743.63 | 1605.52 | 1593.55 | 1348.43 |

  

| Tags | |
| --- | --- |

  

##

## P06745

G6PI\_MOUSE
Glucose-6-phosphate isomerase OS=Mus musculus GN=Gpi PE=1 SV=4  
2
peptides

  

| Sequence | Peptide Ion | Score | Hits | Mass | Charge | Tags | Conflicts | Modifications | In quantitation | Average Normalised Abundances | | | |
| --- | --- | --- | --- | --- | --- | --- | --- | --- | --- | --- | --- | --- | --- |
|  |  |  |  |  |  |  |  |  |  | 2m2f | 4m2f | 2m4f | 4m4f |
| ELFEADPER | 10062 | 43.13 | 3 | 1104.5082 | 2 |  | 0 |  | yes | 468.40 | 389.90 | 322.10 | 212.76 |
| TLASLSPETSLFIIASK | 6190 | 30.27 | 2 | 1776.9874 | 2 |  | 0 |  | yes | 3521.30 | 1917.70 | 3544.50 | 1097.84 |

  

| Tags | |
| --- | --- |

  

##

## Q8BH04

PCKGM\_MOUSE
Phosphoenolpyruvate carboxykinase [GTP], mitochondrial OS=Mus musculus GN=Pck2
PE=2 SV=1  
1 peptide

  

| Sequence | Peptide Ion | Score | Hits | Mass | Charge | Tags | Conflicts | Modifications | In quantitation | Average Normalised Abundances | | | |
| --- | --- | --- | --- | --- | --- | --- | --- | --- | --- | --- | --- | --- | --- |
|  |  |  |  |  |  |  |  |  |  | 2m2f | 4m2f | 2m4f | 4m4f |
| EVLAELEALEGR | 10488 | 73.37 | 3 | 1327.6980 | 2 |  | 0 |  | yes | 453.95 | 125.86 | 327.90 | 254.79 |

  

| Tags | |
| --- | --- |

  

##

## Q62095

DDX3Y\_MOUSE
ATP-dependent RNA helicase DDX3Y OS=Mus musculus GN=Ddx3y PE=1 SV=2  
2
peptides

  

| Sequence | Peptide Ion | Score | Hits | Mass | Charge | Tags | Conflicts | Modifications | In quantitation | Average Normalised Abundances | | | |
| --- | --- | --- | --- | --- | --- | --- | --- | --- | --- | --- | --- | --- | --- |
|  |  |  |  |  |  |  |  |  |  | 2m2f | 4m2f | 2m4f | 4m4f |
| DLLDLLVEAK | 9776 | 45.90 | 3 | 1127.6432 | 2 |  | 0 |  | yes | 712.33 | 262.51 | 710.44 | 259.42 |
| VGNLGLATSFFNER | 11478 | 26.85 | 3 | 1523.7730 | 2 |  | 0 |  | yes | 526.39 | 186.52 | 388.32 | 322.85 |

  

| Tags | |
| --- | --- |

  

##

## Q8R2E9

ERO1B\_MOUSE
ERO1-like protein beta OS=Mus musculus GN=Ero1lb PE=2 SV=1  
2
peptides

  

| Sequence | Peptide Ion | Score | Hits | Mass | Charge | Tags | Conflicts | Modifications | In quantitation | Average Normalised Abundances | | | |
| --- | --- | --- | --- | --- | --- | --- | --- | --- | --- | --- | --- | --- | --- |
|  |  |  |  |  |  |  |  |  |  | 2m2f | 4m2f | 2m4f | 4m4f |
| SIVDLYTGNVEDDADTK | 11961 | 33.47 | 1 | 1853.8530 | 2 |  | 0 |  | yes | 447.96 | 922.54 | 707.52 | 532.54 |
| TLLLSIFQDTK | 6019 | 39.14 | 3 | 1277.7176 | 2 |  | 0 |  | yes | 1385.66 | 1185.11 | 1843.99 | 1389.23 |

  

| Tags | |
| --- | --- |

  

##

## P13707

GPDA\_MOUSE
Glycerol-3-phosphate dehydrogenase [NAD+], cytoplasmic OS=Mus musculus GN=Gpd1
PE=1 SV=3  
2 peptides

  

| Sequence | Peptide Ion | Score | Hits | Mass | Charge | Tags | Conflicts | Modifications | In quantitation | Average Normalised Abundances | | | |
| --- | --- | --- | --- | --- | --- | --- | --- | --- | --- | --- | --- | --- | --- |
|  |  |  |  |  |  |  |  |  |  | 2m2f | 4m2f | 2m4f | 4m4f |
| VAEAFAR | 17828 | 36.21 | 3 | 762.3989 | 2 |  | 0 |  | yes | 223.59 | 125.58 | 157.42 | 121.40 |
| VCIVGSGNWGSAIAK | 13802 | 36.32 | 1 | 1517.7646 | 2 |  | 0 |  | yes | 401.48 | 390.60 | 372.19 | 293.42 |

  

| Tags | |
| --- | --- |

  

##

## P06869

UROK\_MOUSE
Urokinase-type plasminogen activator OS=Mus musculus GN=Plau PE=1
SV=1  
2 peptides

  

| Sequence | Peptide Ion | Score | Hits | Mass | Charge | Tags | Conflicts | Modifications | In quantitation | Average Normalised Abundances | | | |
| --- | --- | --- | --- | --- | --- | --- | --- | --- | --- | --- | --- | --- | --- |
|  |  |  |  |  |  |  |  |  |  | 2m2f | 4m2f | 2m4f | 4m4f |
| ESESDYLYPK | 12032 | 37.95 | 3 | 1229.5470 | 2 |  | 0 |  | yes | 148.40 | 595.13 | 301.75 | 439.29 |
| FEVEQLILHEYYR | 12732 | 34.36 | 3 | 1737.8669 | 3 |  | 0 |  | yes | 173.61 | 124.16 | 214.64 | 206.81 |

  

| Tags | |
| --- | --- |

  

##

## P02468

LAMC1\_MOUSE
Laminin subunit gamma-1 OS=Mus musculus GN=Lamc1 PE=1 SV=2  
1
peptide

  

| Sequence | Peptide Ion | Score | Hits | Mass | Charge | Tags | Conflicts | Modifications | In quantitation | Average Normalised Abundances | | | |
| --- | --- | --- | --- | --- | --- | --- | --- | --- | --- | --- | --- | --- | --- |
|  |  |  |  |  |  |  |  |  |  | 2m2f | 4m2f | 2m4f | 4m4f |
| LSAEDLVLEGAGLR | 15103 | 72.18 | 3 | 1441.7803 | 2 |  | 0 |  | yes | 452.39 | 52.38 | 286.25 | 294.99 |

  

| Tags | |
| --- | --- |

  

##

## Q921U8

SMTN\_MOUSE
Smoothelin OS=Mus musculus GN=Smtn PE=2 SV=2  
1 peptide

  

| Sequence | Peptide Ion | Score | Hits | Mass | Charge | Tags | Conflicts | Modifications | In quantitation | Average Normalised Abundances | | | |
| --- | --- | --- | --- | --- | --- | --- | --- | --- | --- | --- | --- | --- | --- |
|  |  |  |  |  |  |  |  |  |  | 2m2f | 4m2f | 2m4f | 4m4f |
| SPLSAEELTAIEDEGVLDK | 20907 | 72.15 | 1 | 2014.9914 | 2 |  | 0 |  | yes | 278.51 | 41.25 | 188.60 | 186.82 |

  

| Tags | |
| --- | --- |

  

##

## Q922Q4

P5CR2\_MOUSE
Pyrroline-5-carboxylate reductase 2 OS=Mus musculus GN=Pycr2 PE=2
SV=1  
1 peptide

  

| Sequence | Peptide Ion | Score | Hits | Mass | Charge | Tags | Conflicts | Modifications | In quantitation | Average Normalised Abundances | | | |
| --- | --- | --- | --- | --- | --- | --- | --- | --- | --- | --- | --- | --- | --- |
|  |  |  |  |  |  |  |  |  |  | 2m2f | 4m2f | 2m4f | 4m4f |
| SLLINAVEASCIR | 15093 | 71.31 | 2 | 1444.7814 | 2 |  | 0 |  | yes | 553.73 | 384.78 | 353.84 | 442.98 |

  

| Tags | |
| --- | --- |

  

##

## P97447

FHL1\_MOUSE
Four and a half LIM domains protein 1 OS=Mus musculus GN=Fhl1 PE=2
SV=3  
1 peptide

  

| Sequence | Peptide Ion | Score | Hits | Mass | Charge | Tags | Conflicts | Modifications | In quantitation | Average Normalised Abundances | | | |
| --- | --- | --- | --- | --- | --- | --- | --- | --- | --- | --- | --- | --- | --- |
|  |  |  |  |  |  |  |  |  |  | 2m2f | 4m2f | 2m4f | 4m4f |
| AIVAGDQNVEYK | 8007 | 71.02 | 3 | 1305.6569 | 2 |  | 0 |  | yes | 796.44 | 208.97 | 456.14 | 295.33 |

  

| Tags | |
| --- | --- |

  

##

## P26039

TLN1\_MOUSE
Talin-1 OS=Mus musculus GN=Tln1 PE=1 SV=1  
2 peptides

  

| Sequence | Peptide Ion | Score | Hits | Mass | Charge | Tags | Conflicts | Modifications | In quantitation | Average Normalised Abundances | | | |
| --- | --- | --- | --- | --- | --- | --- | --- | --- | --- | --- | --- | --- | --- |
|  |  |  |  |  |  |  |  |  |  | 2m2f | 4m2f | 2m4f | 4m4f |
| GLAGAVSELLR | 14883 | 26.35 | 2 | 1084.6225 | 2 |  | 0 |  | yes | 100.37 | 33.82 | 112.52 | 73.08 |
| TLAESALQLLYTAK | 11423 | 44.11 | 3 | 1520.8317 | 2 |  | 0 |  | yes | 776.49 | 171.77 | 736.49 | 322.74 |
| TLAESALQLLYTAK | 37128 | --- | --- | 1520.8429 | 3 |  | 0 |  | yes | 31.58 | 1.55 | 45.63 | 8.83 |

  

| Tags | |
| --- | --- |

  

##

## P27659

RL3\_MOUSE
60S ribosomal protein L3 OS=Mus musculus GN=Rpl3 PE=2 SV=2  
2
peptides

  

| Sequence | Peptide Ion | Score | Hits | Mass | Charge | Tags | Conflicts | Modifications | In quantitation | Average Normalised Abundances | | | |
| --- | --- | --- | --- | --- | --- | --- | --- | --- | --- | --- | --- | --- | --- |
|  |  |  |  |  |  |  |  |  |  | 2m2f | 4m2f | 2m4f | 4m4f |
| NNASTDYDLSDK | 4569 | 39.59 | 3 | 1341.5685 | 2 |  | 0 |  | yes | 1544.05 | 1435.25 | 1383.20 | 1334.55 |
| TVFAEHISDECK | 4937 | 29.30 | 3 | 1434.6377 | 3 |  | 0 |  | yes | 628.05 | 443.51 | 553.12 | 552.59 |

  

| Tags | |
| --- | --- |

  

##

## P57759

ERP29\_MOUSE
Endoplasmic reticulum resident protein 29 OS=Mus musculus GN=Erp29 PE=1
SV=2  
2 peptides

  

| Sequence | Peptide Ion | Score | Hits | Mass | Charge | Tags | Conflicts | Modifications | In quantitation | Average Normalised Abundances | | | |
| --- | --- | --- | --- | --- | --- | --- | --- | --- | --- | --- | --- | --- | --- |
|  |  |  |  |  |  |  |  |  |  | 2m2f | 4m2f | 2m4f | 4m4f |
| ESYPVFYLFR | 13567 | 25.71 | 3 | 1319.6554 | 2 |  | 0 |  | yes | 1057.72 | 284.79 | 816.14 | 1002.60 |
| GALPLDTVTFYK | 4873 | 42.19 | 3 | 1323.7070 | 2 |  | 0 |  | yes | 1476.19 | 1422.37 | 1623.34 | 1329.52 |

  

| Tags | |
| --- | --- |

  

##

## Q922Q8

LRC59\_MOUSE
Leucine-rich repeat-containing protein 59 OS=Mus musculus GN=Lrrc59 PE=2
SV=1  
2 peptides

  

| Sequence | Peptide Ion | Score | Hits | Mass | Charge | Tags | Conflicts | Modifications | In quantitation | Average Normalised Abundances | | | |
| --- | --- | --- | --- | --- | --- | --- | --- | --- | --- | --- | --- | --- | --- |
|  |  |  |  |  |  |  |  |  |  | 2m2f | 4m2f | 2m4f | 4m4f |
| DKLDGNELDLSLSDLNEVPVK | 8515 | 31.72 | 2 | 2312.1679 | 3 |  | 0 |  | yes | 923.36 | 297.15 | 843.08 | 440.17 |
| LVTLPVSFAQLK | 3983 | 35.96 | 3 | 1314.7905 | 2 |  | 0 |  | yes | 2160.93 | 1563.44 | 2305.91 | 2308.06 |

  

| Tags | |
| --- | --- |

  

##

## O09159

MA2B1\_MOUSE
Lysosomal alpha-mannosidase OS=Mus musculus GN=Man2b1 PE=2 SV=3  
1
peptide

  

| Sequence | Peptide Ion | Score | Hits | Mass | Charge | Tags | Conflicts | Modifications | In quantitation | Average Normalised Abundances | | | |
| --- | --- | --- | --- | --- | --- | --- | --- | --- | --- | --- | --- | --- | --- |
|  |  |  |  |  |  |  |  |  |  | 2m2f | 4m2f | 2m4f | 4m4f |
| FLQDTFGSDGLPR | 10576 | 67.58 | 2 | 1451.7040 | 2 |  | 0 |  | yes | 514.24 | 472.66 | 429.41 | 491.68 |

  

| Tags | |
| --- | --- |

  

##

## Q91V04

TRAM1\_MOUSE
Translocating chain-associated membrane protein 1 OS=Mus musculus GN=Tram1 PE=1
SV=3  
1 peptide

  

| Sequence | Peptide Ion | Score | Hits | Mass | Charge | Tags | Conflicts | Modifications | In quantitation | Average Normalised Abundances | | | |
| --- | --- | --- | --- | --- | --- | --- | --- | --- | --- | --- | --- | --- | --- |
|  |  |  |  |  |  |  |  |  |  | 2m2f | 4m2f | 2m4f | 4m4f |
| LDFSTGNFNVLAVR | 6839 | 67.16 | 2 | 1551.8032 | 2 |  | 0 |  | yes | 1263.51 | 727.69 | 1049.09 | 617.86 |

  

| Tags | |
| --- | --- |

  

##

## Q9CQR2

RS21\_MOUSE
40S ribosomal protein S21 OS=Mus musculus GN=Rps21 PE=2 SV=1  
1
peptide

  

| Sequence | Peptide Ion | Score | Hits | Mass | Charge | Tags | Conflicts | Modifications | In quantitation | Average Normalised Abundances | | | |
| --- | --- | --- | --- | --- | --- | --- | --- | --- | --- | --- | --- | --- | --- |
|  |  |  |  |  |  |  |  |  |  | 2m2f | 4m2f | 2m4f | 4m4f |
| MGESDDSILR | 3830 | 66.95 | 3 | 1121.5010 | 2 |  | 0 |  | yes | 2350.06 | 2502.82 | 2213.05 | 2196.20 |

  

| Tags | |
| --- | --- |

  

##

## P60334

CDO1\_MOUSE
Cysteine dioxygenase type 1 OS=Mus musculus GN=Cdo1 PE=1 SV=1  
2
peptides

  

| Sequence | Peptide Ion | Score | Hits | Mass | Charge | Tags | Conflicts | Modifications | In quantitation | Average Normalised Abundances | | | |
| --- | --- | --- | --- | --- | --- | --- | --- | --- | --- | --- | --- | --- | --- |
|  |  |  |  |  |  |  |  |  |  | 2m2f | 4m2f | 2m4f | 4m4f |
| ENQCAYINDSIGLHR | 4707 | 34.79 | 2 | 1788.8249 | 3 |  | 0 |  | yes | 953.56 | 789.97 | 893.15 | 932.78 |
| ETLFDWPDKK | 5135 | 31.23 | 3 | 1277.6283 | 3 |  | 0 |  | yes | 548.42 | 577.83 | 617.29 | 766.23 |

  

| Tags | |
| --- | --- |

  

##

## Q8VEM8

MPCP\_MOUSE
Phosphate carrier protein, mitochondrial OS=Mus musculus GN=Slc25a3 PE=1
SV=1  
1 peptide

  

| Sequence | Peptide Ion | Score | Hits | Mass | Charge | Tags | Conflicts | Modifications | In quantitation | Average Normalised Abundances | | | |
| --- | --- | --- | --- | --- | --- | --- | --- | --- | --- | --- | --- | --- | --- |
|  |  |  |  |  |  |  |  |  |  | 2m2f | 4m2f | 2m4f | 4m4f |
| ALYSNILGEENTYLWR | 19454 | 65.83 | 1 | 1940.9648 | 2 |  | 0 |  | yes | 468.00 | 62.00 | 259.66 | 84.57 |

  

| Tags | |
| --- | --- |

  

##

## Q99LC5

ETFA\_MOUSE
Electron transfer flavoprotein subunit alpha, mitochondrial OS=Mus musculus
GN=Etfa PE=1 SV=2  
2 peptides

  

| Sequence | Peptide Ion | Score | Hits | Mass | Charge | Tags | Conflicts | Modifications | In quantitation | Average Normalised Abundances | | | |
| --- | --- | --- | --- | --- | --- | --- | --- | --- | --- | --- | --- | --- | --- |
|  |  |  |  |  |  |  |  |  |  | 2m2f | 4m2f | 2m4f | 4m4f |
| GLLPEELTPLILETQK | 35694 | --- | --- | 1793.0142 | 3 |  | 0 |  | yes | 141.19 | 7.44 | 103.61 | 43.77 |
| GLLPEELTPLILETQK | 15706 | 34.45 | 3 | 1793.0188 | 2 |  | 0 |  | yes | 875.14 | 90.25 | 641.91 | 461.79 |
| LNVAPVSDIIEIK | 8113 | 31.31 | 1 | 1409.8133 | 2 |  | 0 |  | yes | 529.52 | 313.46 | 671.77 | 481.67 |

  

| Tags | |
| --- | --- |

  

##

## Q00896

A1AT3\_MOUSE
Alpha-1-antitrypsin 1-3 OS=Mus musculus GN=Serpina1c PE=1 SV=2  
2
peptides

  

| Sequence | Peptide Ion | Score | Hits | Mass | Charge | Tags | Conflicts | Modifications | In quantitation | Average Normalised Abundances | | | |
| --- | --- | --- | --- | --- | --- | --- | --- | --- | --- | --- | --- | --- | --- |
|  |  |  |  |  |  |  |  |  |  | 2m2f | 4m2f | 2m4f | 4m4f |
| LSISGEYNLK | 5251 | 32.65 | 3 | 1122.5956 | 2 |  | 0 |  | yes | 824.44 | 693.29 | 870.53 | 573.75 |
| VINDFVEK | 3761 | 32.83 | 3 | 962.5057 | 2 |  | 0 |  | yes | 778.99 | 909.26 | 845.89 | 669.81 |

  

| Tags | |
| --- | --- |

  

##

## Q8BP47

SYNC\_MOUSE
Asparaginyl-tRNA synthetase, cytoplasmic OS=Mus musculus GN=Nars PE=1
SV=2  
2 peptides

  

| Sequence | Peptide Ion | Score | Hits | Mass | Charge | Tags | Conflicts | Modifications | In quantitation | Average Normalised Abundances | | | |
| --- | --- | --- | --- | --- | --- | --- | --- | --- | --- | --- | --- | --- | --- |
|  |  |  |  |  |  |  |  |  |  | 2m2f | 4m2f | 2m4f | 4m4f |
| LMTDTINEPILLCR | 12318 | 26.09 | 2 | 1687.8578 | 2 |  | 0 |  | yes | 557.05 | 319.29 | 635.57 | 369.55 |
| SWDSEEILEGYK | 11914 | 38.89 | 1 | 1454.6569 | 2 |  | 0 |  | yes | 403.49 | 307.54 | 486.86 | 391.57 |

  

| Tags | |
| --- | --- |

  

##

## P62900

RL31\_MOUSE
60S ribosomal protein L31 OS=Mus musculus GN=Rpl31 PE=2 SV=1  
1
peptide

  

| Sequence | Peptide Ion | Score | Hits | Mass | Charge | Tags | Conflicts | Modifications | In quantitation | Average Normalised Abundances | | | |
| --- | --- | --- | --- | --- | --- | --- | --- | --- | --- | --- | --- | --- | --- |
|  |  |  |  |  |  |  |  |  |  | 2m2f | 4m2f | 2m4f | 4m4f |
| LYTLVTYVPVTTFK | 2846 | 64.03 | 3 | 1643.9180 | 2 |  | 0 |  | yes | 7721.79 | 6182.24 | 7534.07 | 6176.39 |

  

| Tags | |
| --- | --- |

  

##

## P61979

HNRPK\_MOUSE
Heterogeneous nuclear ribonucleoprotein K OS=Mus musculus GN=Hnrnpk PE=1
SV=1  
1 peptide

  

| Sequence | Peptide Ion | Score | Hits | Mass | Charge | Tags | Conflicts | Modifications | In quantitation | Average Normalised Abundances | | | |
| --- | --- | --- | --- | --- | --- | --- | --- | --- | --- | --- | --- | --- | --- |
|  |  |  |  |  |  |  |  |  |  | 2m2f | 4m2f | 2m4f | 4m4f |
| IILDLISESPIK | 5501 | 63.65 | 3 | 1339.7962 | 2 |  | 0 |  | yes | 2144.90 | 1199.78 | 2050.55 | 1372.46 |

  

| Tags | |
| --- | --- |

  

##

## A3KMP2

TTC38\_MOUSE
Tetratricopeptide repeat protein 38 OS=Mus musculus GN=Ttc38 PE=2
SV=2  
1 peptide

  

| Sequence | Peptide Ion | Score | Hits | Mass | Charge | Tags | Conflicts | Modifications | In quantitation | Average Normalised Abundances | | | |
| --- | --- | --- | --- | --- | --- | --- | --- | --- | --- | --- | --- | --- | --- |
|  |  |  |  |  |  |  |  |  |  | 2m2f | 4m2f | 2m4f | 4m4f |
| VLELLLPIR | 20017 | 62.81 | 3 | 1064.6952 | 2 |  | 0 |  | yes | 125.26 | 66.09 | 138.57 | 93.90 |

  

| Tags | |
| --- | --- |

  

##

## P62911

RL32\_MOUSE
60S ribosomal protein L32 OS=Mus musculus GN=Rpl32 PE=2 SV=2  
1
peptide

  

| Sequence | Peptide Ion | Score | Hits | Mass | Charge | Tags | Conflicts | Modifications | In quantitation | Average Normalised Abundances | | | |
| --- | --- | --- | --- | --- | --- | --- | --- | --- | --- | --- | --- | --- | --- |
|  |  |  |  |  |  |  |  |  |  | 2m2f | 4m2f | 2m4f | 4m4f |
| ELEVLLMCNK | 9075 | 62.02 | 3 | 1247.6241 | 2 |  | 0 |  | yes | 356.19 | 345.08 | 421.37 | 249.31 |

  

| Tags | |
| --- | --- |

  

##

## Q9Z1Z0

USO1\_MOUSE
General vesicular transport factor p115 OS=Mus musculus GN=Uso1 PE=1
SV=2  
1 peptide

  

| Sequence | Peptide Ion | Score | Hits | Mass | Charge | Tags | Conflicts | Modifications | In quantitation | Average Normalised Abundances | | | |
| --- | --- | --- | --- | --- | --- | --- | --- | --- | --- | --- | --- | --- | --- |
|  |  |  |  |  |  |  |  |  |  | 2m2f | 4m2f | 2m4f | 4m4f |
| NDGVLLLQALTR | 15246 | 61.22 | 3 | 1311.7446 | 2 |  | 0 |  | yes | 455.31 | 76.84 | 344.17 | 263.07 |

  

| Tags | |
| --- | --- |

  

##

## P49312

ROA1\_MOUSE
Heterogeneous nuclear ribonucleoprotein A1 OS=Mus musculus GN=Hnrnpa1 PE=1
SV=2  
1 peptide

  

| Sequence | Peptide Ion | Score | Hits | Mass | Charge | Tags | Conflicts | Modifications | In quantitation | Average Normalised Abundances | | | |
| --- | --- | --- | --- | --- | --- | --- | --- | --- | --- | --- | --- | --- | --- |
|  |  |  |  |  |  |  |  |  |  | 2m2f | 4m2f | 2m4f | 4m4f |
| LFIGGLSFETTDESLR | 18956 | 61.21 | 2 | 1783.8982 | 2 |  | 0 |  | yes | 325.44 | 109.05 | 297.08 | 92.45 |

  

| Tags | |
| --- | --- |

  

##

## Q99JI6

RAP1B\_MOUSE
Ras-related protein Rap-1b OS=Mus musculus GN=Rap1b PE=2 SV=2  
1
peptide

  

| Sequence | Peptide Ion | Score | Hits | Mass | Charge | Tags | Conflicts | Modifications | In quantitation | Average Normalised Abundances | | | |
| --- | --- | --- | --- | --- | --- | --- | --- | --- | --- | --- | --- | --- | --- |
|  |  |  |  |  |  |  |  |  |  | 2m2f | 4m2f | 2m4f | 4m4f |
| INVNEIFYDLVR | 9874 | 60.41 | 3 | 1493.7877 | 2 |  | 0 |  | yes | 1600.60 | 593.02 | 1187.10 | 303.80 |

  

| Tags | |
| --- | --- |

  

##

## Q61024

ASNS\_MOUSE
Asparagine synthetase [glutamine-hydrolyzing] OS=Mus musculus GN=Asns PE=2
SV=3  
2 peptides

  

| Sequence | Peptide Ion | Score | Hits | Mass | Charge | Tags | Conflicts | Modifications | In quantitation | Average Normalised Abundances | | | |
| --- | --- | --- | --- | --- | --- | --- | --- | --- | --- | --- | --- | --- | --- |
|  |  |  |  |  |  |  |  |  |  | 2m2f | 4m2f | 2m4f | 4m4f |
| ELYLFDVLR | 10502 | 32.87 | 3 | 1166.6334 | 2 |  | 0 |  | yes | 480.41 | 212.72 | 591.04 | 458.66 |
| IGCLLSGGLDSSLVAASLLK | 18367 | 26.96 | 1 | 1973.0823 | 2 |  | 0 |  | yes | 874.25 | 146.07 | 452.58 | 177.28 |

  

| Tags | |
| --- | --- |

  

##

## Q5XJY5

COPD\_MOUSE
Coatomer subunit delta OS=Mus musculus GN=Arcn1 PE=2 SV=1  
1
peptide

  

| Sequence | Peptide Ion | Score | Hits | Mass | Charge | Tags | Conflicts | Modifications | In quantitation | Average Normalised Abundances | | | |
| --- | --- | --- | --- | --- | --- | --- | --- | --- | --- | --- | --- | --- | --- |
|  |  |  |  |  |  |  |  |  |  | 2m2f | 4m2f | 2m4f | 4m4f |
| LFTAESLIGLK | 5381 | 59.74 | 3 | 1190.6906 | 2 |  | 0 |  | yes | 998.06 | 625.80 | 838.89 | 919.98 |

  

| Tags | |
| --- | --- |

  

##

## P62852

RS25\_MOUSE
40S ribosomal protein S25 OS=Mus musculus GN=Rps25 PE=2 SV=1  
1
peptide

  

| Sequence | Peptide Ion | Score | Hits | Mass | Charge | Tags | Conflicts | Modifications | In quantitation | Average Normalised Abundances | | | |
| --- | --- | --- | --- | --- | --- | --- | --- | --- | --- | --- | --- | --- | --- |
|  |  |  |  |  |  |  |  |  |  | 2m2f | 4m2f | 2m4f | 4m4f |
| LITPAVVSER | 2974 | 58.98 | 3 | 1083.6272 | 2 |  | 0 |  | yes | 2573.62 | 2236.10 | 2308.64 | 2508.71 |

  

| Tags | |
| --- | --- |

  

##

## Q62425

NDUA4\_MOUSE
NADH dehydrogenase [ubiquinone] 1 alpha subcomplex subunit 4 OS=Mus musculus
GN=Ndufa4 PE=1 SV=2  
1 peptide

  

| Sequence | Peptide Ion | Score | Hits | Mass | Charge | Tags | Conflicts | Modifications | In quantitation | Average Normalised Abundances | | | |
| --- | --- | --- | --- | --- | --- | --- | --- | --- | --- | --- | --- | --- | --- |
|  |  |  |  |  |  |  |  |  |  | 2m2f | 4m2f | 2m4f | 4m4f |
| LALFNPDVSWDR | 5213 | 57.94 | 3 | 1431.7144 | 2 |  | 0 |  | yes | 2245.51 | 1095.95 | 2121.18 | 1892.27 |

  

| Tags | |
| --- | --- |

  

##

## P10107

ANXA1\_MOUSE
Annexin A1 OS=Mus musculus GN=Anxa1 PE=1 SV=2  
1 peptide

  

| Sequence | Peptide Ion | Score | Hits | Mass | Charge | Tags | Conflicts | Modifications | In quantitation | Average Normalised Abundances | | | |
| --- | --- | --- | --- | --- | --- | --- | --- | --- | --- | --- | --- | --- | --- |
|  |  |  |  |  |  |  |  |  |  | 2m2f | 4m2f | 2m4f | 4m4f |
| GVDEATIIDILTK | 15479 | 57.50 | 3 | 1386.7590 | 2 |  | 0 |  | yes | 357.50 | 50.16 | 245.25 | 75.71 |

  

| Tags | |
| --- | --- |

  

##

## Q9R0P6

SC11A\_MOUSE
Signal peptidase complex catalytic subunit SEC11A OS=Mus musculus GN=Sec11a PE=2
SV=1  
1 peptide

  

| Sequence | Peptide Ion | Score | Hits | Mass | Charge | Tags | Conflicts | Modifications | In quantitation | Average Normalised Abundances | | | |
| --- | --- | --- | --- | --- | --- | --- | --- | --- | --- | --- | --- | --- | --- |
|  |  |  |  |  |  |  |  |  |  | 2m2f | 4m2f | 2m4f | 4m4f |
| MLSLDFLDDVR | 8964 | 57.37 | 3 | 1322.6537 | 2 |  | 0 |  | yes | 1059.60 | 392.94 | 860.74 | 427.16 |

  

| Tags | |
| --- | --- |

  

##

## P97429

ANXA4\_MOUSE
Annexin A4 OS=Mus musculus GN=Anxa4 PE=2 SV=3  
1 peptide

  

| Sequence | Peptide Ion | Score | Hits | Mass | Charge | Tags | Conflicts | Modifications | In quantitation | Average Normalised Abundances | | | |
| --- | --- | --- | --- | --- | --- | --- | --- | --- | --- | --- | --- | --- | --- |
|  |  |  |  |  |  |  |  |  |  | 2m2f | 4m2f | 2m4f | 4m4f |
| SETSGSFEDALLAIVK | 13472 | 57.08 | 3 | 1665.8485 | 2 |  | 0 |  | yes | 1994.56 | 245.13 | 921.61 | 505.44 |

  

| Tags | |
| --- | --- |

  

##

## Q8CIE6

COPA\_MOUSE
Coatomer subunit alpha OS=Mus musculus GN=Copa PE=1 SV=1  
1
peptide

  

| Sequence | Peptide Ion | Score | Hits | Mass | Charge | Tags | Conflicts | Modifications | In quantitation | Average Normalised Abundances | | | |
| --- | --- | --- | --- | --- | --- | --- | --- | --- | --- | --- | --- | --- | --- |
|  |  |  |  |  |  |  |  |  |  | 2m2f | 4m2f | 2m4f | 4m4f |
| SILLSVPLLVVDNK | 11090 | 56.57 | 3 | 1508.9173 | 2 |  | 0 |  | yes | 1157.26 | 259.71 | 898.12 | 553.84 |

  

| Tags | |
| --- | --- |

  

##

## P34884

MIF\_MOUSE
Macrophage migration inhibitory factor OS=Mus musculus GN=Mif PE=1
SV=2  
1 peptide

  

| Sequence | Peptide Ion | Score | Hits | Mass | Charge | Tags | Conflicts | Modifications | In quantitation | Average Normalised Abundances | | | |
| --- | --- | --- | --- | --- | --- | --- | --- | --- | --- | --- | --- | --- | --- |
|  |  |  |  |  |  |  |  |  |  | 2m2f | 4m2f | 2m4f | 4m4f |
| PMFIVNTNVPR | 6538 | 56.53 | 3 | 1286.6781 | 2 |  | 0 |  | yes | 792.64 | 673.37 | 648.30 | 566.93 |

  

| Tags | |
| --- | --- |

  

##

## P49817

CAV1\_MOUSE
Caveolin-1 OS=Mus musculus GN=Cav1 PE=1 SV=1  
1 peptide

  

| Sequence | Peptide Ion | Score | Hits | Mass | Charge | Tags | Conflicts | Modifications | In quantitation | Average Normalised Abundances | | | |
| --- | --- | --- | --- | --- | --- | --- | --- | --- | --- | --- | --- | --- | --- |
|  |  |  |  |  |  |  |  |  |  | 2m2f | 4m2f | 2m4f | 4m4f |
| IDFEDVIAEPEGTHSFDGIWK | 11422 | 56.33 | 2 | 2404.1199 | 3 |  | 0 |  | yes | 869.78 | 9.55 | 886.21 | 199.14 |

  

| Tags | |
| --- | --- |

  

##

## Q01149

CO1A2\_MOUSE
Collagen alpha-2(I) chain OS=Mus musculus GN=Col1a2 PE=2 SV=2  
1
peptide

  

| Sequence | Peptide Ion | Score | Hits | Mass | Charge | Tags | Conflicts | Modifications | In quantitation | Average Normalised Abundances | | | |
| --- | --- | --- | --- | --- | --- | --- | --- | --- | --- | --- | --- | --- | --- |
|  |  |  |  |  |  |  |  |  |  | 2m2f | 4m2f | 2m4f | 4m4f |
| GEAGAAGPSGPAGPR | 7921 | 56.29 | 3 | 1250.6004 | 2 |  | 0 |  | yes | 942.27 | 13.06 | 538.81 | 188.13 |

  

| Tags | |
| --- | --- |

  

##

## P10630

IF4A2\_MOUSE
Eukaryotic initiation factor 4A-II OS=Mus musculus GN=Eif4a2 PE=2
SV=2  
1 peptide

  

| Sequence | Peptide Ion | Score | Hits | Mass | Charge | Tags | Conflicts | Modifications | In quantitation | Average Normalised Abundances | | | |
| --- | --- | --- | --- | --- | --- | --- | --- | --- | --- | --- | --- | --- | --- |
|  |  |  |  |  |  |  |  |  |  | 2m2f | 4m2f | 2m4f | 4m4f |
| VLITTDLLAR | 2420 | 56.16 | 3 | 1113.6742 | 2 |  | 0 |  | yes | 2201.13 | 2160.16 | 2863.66 | 2227.50 |

  

| Tags | |
| --- | --- |

  

##

## P14602

HSPB1\_MOUSE
Heat shock protein beta-1 OS=Mus musculus GN=Hspb1 PE=1 SV=3  
1
peptide

  

| Sequence | Peptide Ion | Score | Hits | Mass | Charge | Tags | Conflicts | Modifications | In quantitation | Average Normalised Abundances | | | |
| --- | --- | --- | --- | --- | --- | --- | --- | --- | --- | --- | --- | --- | --- |
|  |  |  |  |  |  |  |  |  |  | 2m2f | 4m2f | 2m4f | 4m4f |
| LFDQAFGVPR | 5995 | 56.13 | 3 | 1148.5963 | 2 |  | 0 |  | yes | 872.95 | 1199.00 | 1073.29 | 2053.48 |

  

| Tags | |
| --- | --- |

  

##

## P80316

TCPE\_MOUSE
T-complex protein 1 subunit epsilon OS=Mus musculus GN=Cct5 PE=1 SV=1  
1
peptide

  

| Sequence | Peptide Ion | Score | Hits | Mass | Charge | Tags | Conflicts | Modifications | In quantitation | Average Normalised Abundances | | | |
| --- | --- | --- | --- | --- | --- | --- | --- | --- | --- | --- | --- | --- | --- |
|  |  |  |  |  |  |  |  |  |  | 2m2f | 4m2f | 2m4f | 4m4f |
| LGFAGVVQEISFGTTK | 19485 | 56.08 | 1 | 1652.8764 | 2 |  | 0 |  | yes | 208.01 | 32.63 | 164.77 | 84.73 |

  

| Tags | |
| --- | --- |

  

##

## P47955

RLA1\_MOUSE
60S acidic ribosomal protein P1 OS=Mus musculus GN=Rplp1 PE=1 SV=1  
1
peptide

  

| Sequence | Peptide Ion | Score | Hits | Mass | Charge | Tags | Conflicts | Modifications | In quantitation | Average Normalised Abundances | | | |
| --- | --- | --- | --- | --- | --- | --- | --- | --- | --- | --- | --- | --- | --- |
|  |  |  |  |  |  |  |  |  |  | 2m2f | 4m2f | 2m4f | 4m4f |
| AAGVSVEPFWPGLFAK | 14242 | 30.30 | 1 | 1674.8762 | 3 |  | 0 |  | yes | 460.06 | 51.92 | 379.45 | 208.71 |
| AAGVSVEPFWPGLFAK | 2748 | 25.76 | 3 | 1674.8746 | 2 |  | 0 |  | yes | 1.81e+004 | 6939.45 | 1.57e+004 | 1.27e+004 |

  

| Tags | |
| --- | --- |

  

##

## Q60932

VDAC1\_MOUSE
Voltage-dependent anion-selective channel protein 1 OS=Mus musculus GN=Vdac1
PE=1 SV=3  
1 peptide

  

| Sequence | Peptide Ion | Score | Hits | Mass | Charge | Tags | Conflicts | Modifications | In quantitation | Average Normalised Abundances | | | |
| --- | --- | --- | --- | --- | --- | --- | --- | --- | --- | --- | --- | --- | --- |
|  |  |  |  |  |  |  |  |  |  | 2m2f | 4m2f | 2m4f | 4m4f |
| WTEYGLTFTEK | 5461 | 55.81 | 3 | 1373.6487 | 2 |  | 0 |  | yes | 1046.21 | 1050.66 | 1147.19 | 1046.34 |

  

| Tags | |
| --- | --- |

  

##

## P20108

PRDX3\_MOUSE
Thioredoxin-dependent peroxide reductase, mitochondrial OS=Mus musculus GN=Prdx3
PE=1 SV=1  
1 peptide

  

| Sequence | Peptide Ion | Score | Hits | Mass | Charge | Tags | Conflicts | Modifications | In quantitation | Average Normalised Abundances | | | |
| --- | --- | --- | --- | --- | --- | --- | --- | --- | --- | --- | --- | --- | --- |
|  |  |  |  |  |  |  |  |  |  | 2m2f | 4m2f | 2m4f | 4m4f |
| GLFIIDPNGVVK | 6703 | 55.45 | 3 | 1270.7278 | 2 |  | 0 |  | yes | 912.26 | 554.75 | 772.65 | 736.81 |

  

| Tags | |
| --- | --- |

  

##

## P62264

RS14\_MOUSE
40S ribosomal protein S14 OS=Mus musculus GN=Rps14 PE=2 SV=3  
1
peptide

  

| Sequence | Peptide Ion | Score | Hits | Mass | Charge | Tags | Conflicts | Modifications | In quantitation | Average Normalised Abundances | | | |
| --- | --- | --- | --- | --- | --- | --- | --- | --- | --- | --- | --- | --- | --- |
|  |  |  |  |  |  |  |  |  |  | 2m2f | 4m2f | 2m4f | 4m4f |
| IEDVTPIPSDSTR | 3057 | 54.34 | 3 | 1428.7094 | 2 |  | 0 |  | yes | 3318.66 | 2199.87 | 2647.98 | 2706.08 |

  

| Tags | |
| --- | --- |

  

##

## Q62465

VAT1\_MOUSE
Synaptic vesicle membrane protein VAT-1 homolog OS=Mus musculus GN=Vat1 PE=1
SV=3  
1 peptide

  

| Sequence | Peptide Ion | Score | Hits | Mass | Charge | Tags | Conflicts | Modifications | In quantitation | Average Normalised Abundances | | | |
| --- | --- | --- | --- | --- | --- | --- | --- | --- | --- | --- | --- | --- | --- |
|  |  |  |  |  |  |  |  |  |  | 2m2f | 4m2f | 2m4f | 4m4f |
| VVTYGMANLLTGPK | 11794 | 54.20 | 2 | 1462.7848 | 2 |  | 0 |  | yes | 494.63 | 296.33 | 471.06 | 392.43 |

  

| Tags | |
| --- | --- |

  

##

## Q78XF5

OSTC\_MOUSE
Oligosaccharyltransferase complex subunit OSTC OS=Mus musculus GN=Ostc PE=2
SV=1  
1 peptide

  

| Sequence | Peptide Ion | Score | Hits | Mass | Charge | Tags | Conflicts | Modifications | In quantitation | Average Normalised Abundances | | | |
| --- | --- | --- | --- | --- | --- | --- | --- | --- | --- | --- | --- | --- | --- |
|  |  |  |  |  |  |  |  |  |  | 2m2f | 4m2f | 2m4f | 4m4f |
| VPFLVLECPNLK | 6018 | 54.09 | 3 | 1427.7838 | 2 |  | 0 |  | yes | 1292.27 | 761.80 | 992.57 | 1120.78 |

  

| Tags | |
| --- | --- |

  

##

## Q6ZWY3

RS27L\_MOUSE
40S ribosomal protein S27-like OS=Mus musculus GN=Rps27l PE=2 SV=3  
2
peptides

  

| Sequence | Peptide Ion | Score | Hits | Mass | Charge | Tags | Conflicts | Modifications | In quantitation | Average Normalised Abundances | | | |
| --- | --- | --- | --- | --- | --- | --- | --- | --- | --- | --- | --- | --- | --- |
|  |  |  |  |  |  |  |  |  |  | 2m2f | 4m2f | 2m4f | 4m4f |
| DLLHPSLEEEK | 6616 | 25.44 | 3 | 1308.6561 | 3 |  | 0 |  | yes | 413.19 | 282.20 | 322.50 | 325.95 |
| LTEGCSFR | 1679 | 28.11 | 3 | 968.4379 | 2 |  | 0 |  | yes | 2420.51 | 3730.49 | 2414.16 | 2819.89 |

  

| Tags | |
| --- | --- |

  

##

## P10922

H10\_MOUSE
Histone H1.0 OS=Mus musculus GN=H1f0 PE=2 SV=4  
1 peptide

  

| Sequence | Peptide Ion | Score | Hits | Mass | Charge | Tags | Conflicts | Modifications | In quantitation | Average Normalised Abundances | | | |
| --- | --- | --- | --- | --- | --- | --- | --- | --- | --- | --- | --- | --- | --- |
|  |  |  |  |  |  |  |  |  |  | 2m2f | 4m2f | 2m4f | 4m4f |
| VGENADSQIK | 3551 | 53.11 | 3 | 1059.5194 | 2 |  | 0 |  | yes | 2134.76 | 1134.46 | 1679.12 | 1038.06 |

  

| Tags | |
| --- | --- |

  

##

## Q7TMK9

HNRPQ\_MOUSE
Heterogeneous nuclear ribonucleoprotein Q OS=Mus musculus GN=Syncrip PE=1
SV=2  
1 peptide

  

| Sequence | Peptide Ion | Score | Hits | Mass | Charge | Tags | Conflicts | Modifications | In quantitation | Average Normalised Abundances | | | |
| --- | --- | --- | --- | --- | --- | --- | --- | --- | --- | --- | --- | --- | --- |
|  |  |  |  |  |  |  |  |  |  | 2m2f | 4m2f | 2m4f | 4m4f |
| DLFEDELVPLFEK | 12348 | 52.73 | 3 | 1592.7971 | 2 |  | 0 |  | yes | 939.87 | 224.51 | 895.12 | 524.43 |

  

| Tags | |
| --- | --- |

  

##

## P50580

PA2G4\_MOUSE
Proliferation-associated protein 2G4 OS=Mus musculus GN=Pa2g4 PE=1
SV=3  
1 peptide

  

| Sequence | Peptide Ion | Score | Hits | Mass | Charge | Tags | Conflicts | Modifications | In quantitation | Average Normalised Abundances | | | |
| --- | --- | --- | --- | --- | --- | --- | --- | --- | --- | --- | --- | --- | --- |
|  |  |  |  |  |  |  |  |  |  | 2m2f | 4m2f | 2m4f | 4m4f |
| SLVEASSSGVSVLSLCEK | 11734 | 51.99 | 3 | 1850.9228 | 2 |  | 0 |  | yes | 954.81 | 367.48 | 763.78 | 353.87 |

  

| Tags | |
| --- | --- |

  

##

## P29387

GBB4\_MOUSE
Guanine nucleotide-binding protein subunit beta-4 OS=Mus musculus GN=Gnb4 PE=2
SV=4  
1 peptide

  

| Sequence | Peptide Ion | Score | Hits | Mass | Charge | Tags | Conflicts | Modifications | In quantitation | Average Normalised Abundances | | | |
| --- | --- | --- | --- | --- | --- | --- | --- | --- | --- | --- | --- | --- | --- |
|  |  |  |  |  |  |  |  |  |  | 2m2f | 4m2f | 2m4f | 4m4f |
| LLVSASQDGK | 10908 | 51.61 | 3 | 1016.5503 | 2 |  | 0 |  | yes | 265.67 | 49.57 | 142.32 | 82.02 |

  

| Tags | |
| --- | --- |

  

##

## Q8BH64

EHD2\_MOUSE
EH domain-containing protein 2 OS=Mus musculus GN=Ehd2 PE=1 SV=1  
1
peptide

  

| Sequence | Peptide Ion | Score | Hits | Mass | Charge | Tags | Conflicts | Modifications | In quantitation | Average Normalised Abundances | | | |
| --- | --- | --- | --- | --- | --- | --- | --- | --- | --- | --- | --- | --- | --- |
|  |  |  |  |  |  |  |  |  |  | 2m2f | 4m2f | 2m4f | 4m4f |
| LFELEEQDLFR | 16059 | 51.31 | 1 | 1437.7124 | 2 |  | 0 |  | yes | 353.07 | 95.48 | 362.57 | 138.51 |

  

| Tags | |
| --- | --- |

  

##

## P14069

S10A6\_MOUSE
Protein S100-A6 OS=Mus musculus GN=S100a6 PE=1 SV=3  
1
peptide

  

| Sequence | Peptide Ion | Score | Hits | Mass | Charge | Tags | Conflicts | Modifications | In quantitation | Average Normalised Abundances | | | |
| --- | --- | --- | --- | --- | --- | --- | --- | --- | --- | --- | --- | --- | --- |
|  |  |  |  |  |  |  |  |  |  | 2m2f | 4m2f | 2m4f | 4m4f |
| LQDAEIAR | 1218 | 51.27 | 3 | 914.4824 | 2 |  | 0 |  | yes | 3161.94 | 3838.94 | 3315.63 | 3711.59 |

  

| Tags | |
| --- | --- |

  

##

## Q9CZM2

RL15\_MOUSE
60S ribosomal protein L15 OS=Mus musculus GN=Rpl15 PE=2 SV=4  
1
peptide

  

| Sequence | Peptide Ion | Score | Hits | Mass | Charge | Tags | Conflicts | Modifications | In quantitation | Average Normalised Abundances | | | |
| --- | --- | --- | --- | --- | --- | --- | --- | --- | --- | --- | --- | --- | --- |
|  |  |  |  |  |  |  |  |  |  | 2m2f | 4m2f | 2m4f | 4m4f |
| VLNSYWVGEDSTYK | 4219 | 51.14 | 3 | 1659.7776 | 2 |  | 0 |  | yes | 2540.17 | 2704.16 | 2011.93 | 2228.79 |

  

| Tags | |
| --- | --- |

  

##

## Q8K2B3

DHSA\_MOUSE
Succinate dehydrogenase [ubiquinone] flavoprotein subunit, mitochondrial OS=Mus
musculus GN=Sdha PE=1 SV=1  
1 peptide

  

| Sequence | Peptide Ion | Score | Hits | Mass | Charge | Tags | Conflicts | Modifications | In quantitation | Average Normalised Abundances | | | |
| --- | --- | --- | --- | --- | --- | --- | --- | --- | --- | --- | --- | --- | --- |
|  |  |  |  |  |  |  |  |  |  | 2m2f | 4m2f | 2m4f | 4m4f |
| LGANSLLDLVVFGR | 34175 | 50.52 | 2 | 1472.8346 | 2 |  | 0 |  | yes | 255.35 | 36.83 | 164.37 | 63.55 |

  

| Tags | |
| --- | --- |

  

##

## Q9JJI8

RL38\_MOUSE
60S ribosomal protein L38 OS=Mus musculus GN=Rpl38 PE=2 SV=3  
1
peptide

  

| Sequence | Peptide Ion | Score | Hits | Mass | Charge | Tags | Conflicts | Modifications | In quantitation | Average Normalised Abundances | | | |
| --- | --- | --- | --- | --- | --- | --- | --- | --- | --- | --- | --- | --- | --- |
|  |  |  |  |  |  |  |  |  |  | 2m2f | 4m2f | 2m4f | 4m4f |
| YLYTLVITDK | 4224 | 50.43 | 3 | 1227.6739 | 2 |  | 0 |  | yes | 1269.02 | 1233.27 | 1590.41 | 1097.98 |

  

| Tags | |
| --- | --- |

  

##

## P16254

SRP14\_MOUSE
Signal recognition particle 14 kDa protein OS=Mus musculus GN=Srp14 PE=1
SV=1  
1 peptide

  

| Sequence | Peptide Ion | Score | Hits | Mass | Charge | Tags | Conflicts | Modifications | In quantitation | Average Normalised Abundances | | | |
| --- | --- | --- | --- | --- | --- | --- | --- | --- | --- | --- | --- | --- | --- |
|  |  |  |  |  |  |  |  |  |  | 2m2f | 4m2f | 2m4f | 4m4f |
| VLLESEQFLTELTR | 13146 | 50.39 | 3 | 1676.8975 | 2 |  | 0 |  | yes | 624.59 | 178.60 | 517.37 | 197.36 |

  

| Tags | |
| --- | --- |

  

##

## P00920

CAH2\_MOUSE
Carbonic anhydrase 2 OS=Mus musculus GN=Ca2 PE=1 SV=4  
1
peptide

  

| Sequence | Peptide Ion | Score | Hits | Mass | Charge | Tags | Conflicts | Modifications | In quantitation | Average Normalised Abundances | | | |
| --- | --- | --- | --- | --- | --- | --- | --- | --- | --- | --- | --- | --- | --- |
|  |  |  |  |  |  |  |  |  |  | 2m2f | 4m2f | 2m4f | 4m4f |
| AVQQPDGLAVLGIFLK | 16140 | --- | --- | 1667.9603 | 3 |  | 0 |  | yes | 294.99 | 123.61 | 273.27 | 108.31 |
| AVQQPDGLAVLGIFLK | 11070 | 50.05 | 3 | 1667.9614 | 2 |  | 0 |  | yes | 1306.56 | 541.00 | 1192.68 | 790.98 |

  

| Tags | |
| --- | --- |

  

##

## P48678

LMNA\_MOUSE
Prelamin-A/C OS=Mus musculus GN=Lmna PE=1 SV=2  
1 peptide

  

| Sequence | Peptide Ion | Score | Hits | Mass | Charge | Tags | Conflicts | Modifications | In quantitation | Average Normalised Abundances | | | |
| --- | --- | --- | --- | --- | --- | --- | --- | --- | --- | --- | --- | --- | --- |
|  |  |  |  |  |  |  |  |  |  | 2m2f | 4m2f | 2m4f | 4m4f |
| AAYEAELGDAR | 9756 | 49.80 | 3 | 1164.5433 | 2 |  | 0 |  | yes | 426.81 | 248.12 | 293.29 | 273.27 |

  

| Tags | |
| --- | --- |

  

##

## Q9CXS4

CENPV\_MOUSE
Centromere protein V OS=Mus musculus GN=Cenpv PE=2 SV=2  
1
peptide

  

| Sequence | Peptide Ion | Score | Hits | Mass | Charge | Tags | Conflicts | Modifications | In quantitation | Average Normalised Abundances | | | |
| --- | --- | --- | --- | --- | --- | --- | --- | --- | --- | --- | --- | --- | --- |
|  |  |  |  |  |  |  |  |  |  | 2m2f | 4m2f | 2m4f | 4m4f |
| SGATGGLSGGESR | 26406 | 49.51 | 2 | 1134.5257 | 2 |  | 0 |  | yes | 237.18 | 224.66 | 208.13 | 193.53 |

  

| Tags | |
| --- | --- |

  

##

## Q8R5C5

ACTY\_MOUSE
Beta-centractin OS=Mus musculus GN=Actr1b PE=1 SV=1  
1
peptide

  

| Sequence | Peptide Ion | Score | Hits | Mass | Charge | Tags | Conflicts | Modifications | In quantitation | Average Normalised Abundances | | | |
| --- | --- | --- | --- | --- | --- | --- | --- | --- | --- | --- | --- | --- | --- |
|  |  |  |  |  |  |  |  |  |  | 2m2f | 4m2f | 2m4f | 4m4f |
| TLFSNIVLSGGSTLFK | 18181 | 49.45 | 1 | 1682.9224 | 2 |  | 0 |  | yes | 292.47 | 42.05 | 207.41 | 59.69 |

  

| Tags | |
| --- | --- |

  

##

## P26638

SYSC\_MOUSE
Seryl-tRNA synthetase, cytoplasmic OS=Mus musculus GN=Sars PE=2 SV=3  
1
peptide

  

| Sequence | Peptide Ion | Score | Hits | Mass | Charge | Tags | Conflicts | Modifications | In quantitation | Average Normalised Abundances | | | |
| --- | --- | --- | --- | --- | --- | --- | --- | --- | --- | --- | --- | --- | --- |
|  |  |  |  |  |  |  |  |  |  | 2m2f | 4m2f | 2m4f | 4m4f |
| VLDLDLFR | 4075 | 49.45 | 3 | 989.5538 | 2 |  | 0 |  | yes | 1735.90 | 1291.25 | 1896.05 | 1466.38 |

  

| Tags | |
| --- | --- |

  

##

## P16125

LDHB\_MOUSE
L-lactate dehydrogenase B chain OS=Mus musculus GN=Ldhb PE=1 SV=2  
1
peptide

  

| Sequence | Peptide Ion | Score | Hits | Mass | Charge | Tags | Conflicts | Modifications | In quantitation | Average Normalised Abundances | | | |
| --- | --- | --- | --- | --- | --- | --- | --- | --- | --- | --- | --- | --- | --- |
|  |  |  |  |  |  |  |  |  |  | 2m2f | 4m2f | 2m4f | 4m4f |
| SLADELALVDVLEDK | 12151 | 49.45 | 2 | 1628.8523 | 2 |  | 0 |  | yes | 1048.71 | 104.97 | 757.74 | 257.37 |

  

| Tags | |
| --- | --- |

  

##

## Q9Z1Q5

CLIC1\_MOUSE
Chloride intracellular channel protein 1 OS=Mus musculus GN=Clic1 PE=1
SV=3  
1 peptide

  

| Sequence | Peptide Ion | Score | Hits | Mass | Charge | Tags | Conflicts | Modifications | In quantitation | Average Normalised Abundances | | | |
| --- | --- | --- | --- | --- | --- | --- | --- | --- | --- | --- | --- | --- | --- |
|  |  |  |  |  |  |  |  |  |  | 2m2f | 4m2f | 2m4f | 4m4f |
| LAALNPESNTSGLDIFAK | 9633 | 49.29 | 3 | 1859.9608 | 2 |  | 0 |  | yes | 926.49 | 455.45 | 917.59 | 578.25 |

  

| Tags | |
| --- | --- |

  

##

## P54071

IDHP\_MOUSE
Isocitrate dehydrogenase [NADP], mitochondrial OS=Mus musculus GN=Idh2 PE=1
SV=3  
1 peptide

  

| Sequence | Peptide Ion | Score | Hits | Mass | Charge | Tags | Conflicts | Modifications | In quantitation | Average Normalised Abundances | | | |
| --- | --- | --- | --- | --- | --- | --- | --- | --- | --- | --- | --- | --- | --- |
|  |  |  |  |  |  |  |  |  |  | 2m2f | 4m2f | 2m4f | 4m4f |
| LIDDMVAQVLK | 8574 | 49.04 | 3 | 1243.6831 | 2 |  | 0 |  | yes | 453.25 | 352.36 | 404.91 | 367.12 |

  

| Tags | |
| --- | --- |

  

##

## Q99020

ROAA\_MOUSE
Heterogeneous nuclear ribonucleoprotein A/B OS=Mus musculus GN=Hnrnpab PE=1
SV=1  
1 peptide

  

| Sequence | Peptide Ion | Score | Hits | Mass | Charge | Tags | Conflicts | Modifications | In quantitation | Average Normalised Abundances | | | |
| --- | --- | --- | --- | --- | --- | --- | --- | --- | --- | --- | --- | --- | --- |
|  |  |  |  |  |  |  |  |  |  | 2m2f | 4m2f | 2m4f | 4m4f |
| IFVGGLNPEATEEK | 5860 | 48.01 | 3 | 1502.7616 | 2 |  | 0 |  | yes | 1366.86 | 974.80 | 1158.96 | 1486.80 |

  

| Tags | |
| --- | --- |

  

##

## Q9CXI5

MANF\_MOUSE
Mesencephalic astrocyte-derived neurotrophic factor OS=Mus musculus GN=Manf PE=1
SV=1  
1 peptide

  

| Sequence | Peptide Ion | Score | Hits | Mass | Charge | Tags | Conflicts | Modifications | In quantitation | Average Normalised Abundances | | | |
| --- | --- | --- | --- | --- | --- | --- | --- | --- | --- | --- | --- | --- | --- |
|  |  |  |  |  |  |  |  |  |  | 2m2f | 4m2f | 2m4f | 4m4f |
| DVTFSPATIEEELIK | 5674 | 47.97 | 3 | 1690.8641 | 2 |  | 0 |  | yes | 6296.17 | 3539.29 | 5743.15 | 4991.23 |

  

| Tags | |
| --- | --- |

  

##

## Q61207

SAP\_MOUSE
Sulfated glycoprotein 1 OS=Mus musculus GN=Psap PE=1 SV=2  
1
peptide

  

| Sequence | Peptide Ion | Score | Hits | Mass | Charge | Tags | Conflicts | Modifications | In quantitation | Average Normalised Abundances | | | |
| --- | --- | --- | --- | --- | --- | --- | --- | --- | --- | --- | --- | --- | --- |
|  |  |  |  |  |  |  |  |  |  | 2m2f | 4m2f | 2m4f | 4m4f |
| TVVTEAGNLLK | 3039 | 47.96 | 1 | 1143.6455 | 2 |  | 1 |  | no | 2138.39 | 2835.69 | 2200.81 | 3114.53 |

  

| Tags | |
| --- | --- |

  

##

## P63323

RS12\_MOUSE
40S ribosomal protein S12 OS=Mus musculus GN=Rps12 PE=1 SV=2  
1
peptide

  

| Sequence | Peptide Ion | Score | Hits | Mass | Charge | Tags | Conflicts | Modifications | In quantitation | Average Normalised Abundances | | | |
| --- | --- | --- | --- | --- | --- | --- | --- | --- | --- | --- | --- | --- | --- |
|  |  |  |  |  |  |  |  |  |  | 2m2f | 4m2f | 2m4f | 4m4f |
| LGEWVGLCK | 4289 | 47.93 | 3 | 1060.5344 | 2 |  | 0 |  | yes | 1100.57 | 1044.71 | 936.84 | 1020.56 |

  

| Tags | |
| --- | --- |

  

##

## Q9CZD3

SYG\_MOUSE
Glycyl-tRNA synthetase OS=Mus musculus GN=Gars PE=1 SV=1  
1
peptide

  

| Sequence | Peptide Ion | Score | Hits | Mass | Charge | Tags | Conflicts | Modifications | In quantitation | Average Normalised Abundances | | | |
| --- | --- | --- | --- | --- | --- | --- | --- | --- | --- | --- | --- | --- | --- |
|  |  |  |  |  |  |  |  |  |  | 2m2f | 4m2f | 2m4f | 4m4f |
| TFFSFPAVVAPFK | 13857 | 47.76 | 3 | 1456.7710 | 2 |  | 0 |  | yes | 635.24 | 184.07 | 611.72 | 409.08 |

  

| Tags | |
| --- | --- |

  

##

## Q61753

SERA\_MOUSE
D-3-phosphoglycerate dehydrogenase OS=Mus musculus GN=Phgdh PE=1 SV=3  
1
peptide

  

| Sequence | Peptide Ion | Score | Hits | Mass | Charge | Tags | Conflicts | Modifications | In quantitation | Average Normalised Abundances | | | |
| --- | --- | --- | --- | --- | --- | --- | --- | --- | --- | --- | --- | --- | --- |
|  |  |  |  |  |  |  |  |  |  | 2m2f | 4m2f | 2m4f | 4m4f |
| AGTGVDNVDLEAATR | 10255 | 47.46 | 3 | 1487.7226 | 2 |  | 0 |  | yes | 786.43 | 338.03 | 657.39 | 499.62 |

  

| Tags | |
| --- | --- |

  

##

## Q8CHP8

PGP\_MOUSE
Phosphoglycolate phosphatase OS=Mus musculus GN=Pgp PE=2 SV=1  
1
peptide

  

| Sequence | Peptide Ion | Score | Hits | Mass | Charge | Tags | Conflicts | Modifications | In quantitation | Average Normalised Abundances | | | |
| --- | --- | --- | --- | --- | --- | --- | --- | --- | --- | --- | --- | --- | --- |
|  |  |  |  |  |  |  |  |  |  | 2m2f | 4m2f | 2m4f | 4m4f |
| TILTLTGVSSLEDVK | 7910 | 46.41 | 3 | 1574.8765 | 2 |  | 0 |  | yes | 901.65 | 614.10 | 910.16 | 532.63 |

  

| Tags | |
| --- | --- |

  

##

## O88844

IDHC\_MOUSE
Isocitrate dehydrogenase [NADP] cytoplasmic OS=Mus musculus GN=Idh1 PE=1
SV=1  
1 peptide

  

| Sequence | Peptide Ion | Score | Hits | Mass | Charge | Tags | Conflicts | Modifications | In quantitation | Average Normalised Abundances | | | |
| --- | --- | --- | --- | --- | --- | --- | --- | --- | --- | --- | --- | --- | --- |
|  |  |  |  |  |  |  |  |  |  | 2m2f | 4m2f | 2m4f | 4m4f |
| LVTGWVKPIIIGR | 16983 | 46.39 | 1 | 1450.9017 | 3 |  | 0 |  | yes | 110.78 | 55.82 | 67.56 | 28.21 |

  

| Tags | |
| --- | --- |

  

##

## Q9QUI0

RHOA\_MOUSE
Transforming protein RhoA OS=Mus musculus GN=Rhoa PE=1 SV=1  
1
peptide

  

| Sequence | Peptide Ion | Score | Hits | Mass | Charge | Tags | Conflicts | Modifications | In quantitation | Average Normalised Abundances | | | |
| --- | --- | --- | --- | --- | --- | --- | --- | --- | --- | --- | --- | --- | --- |
|  |  |  |  |  |  |  |  |  |  | 2m2f | 4m2f | 2m4f | 4m4f |
| LVIVGDGACGK | 5858 | 46.16 | 3 | 1087.5697 | 2 |  | 0 |  | yes | 572.82 | 660.06 | 605.39 | 560.29 |

  

| Tags | |
| --- | --- |

  

##

## Q8CIB5

FERM2\_MOUSE
Fermitin family homolog 2 OS=Mus musculus GN=Fermt2 PE=1 SV=1  
1
peptide

  

| Sequence | Peptide Ion | Score | Hits | Mass | Charge | Tags | Conflicts | Modifications | In quantitation | Average Normalised Abundances | | | |
| --- | --- | --- | --- | --- | --- | --- | --- | --- | --- | --- | --- | --- | --- |
|  |  |  |  |  |  |  |  |  |  | 2m2f | 4m2f | 2m4f | 4m4f |
| YYSFFDLNPK | 14184 | 46.15 | 2 | 1292.6074 | 2 |  | 0 |  | yes | 295.76 | 86.51 | 334.05 | 179.36 |

  

| Tags | |
| --- | --- |

  

##

## Q99KK7

DPP3\_MOUSE
Dipeptidyl peptidase 3 OS=Mus musculus GN=Dpp3 PE=2 SV=1  
1
peptide

  

| Sequence | Peptide Ion | Score | Hits | Mass | Charge | Tags | Conflicts | Modifications | In quantitation | Average Normalised Abundances | | | |
| --- | --- | --- | --- | --- | --- | --- | --- | --- | --- | --- | --- | --- | --- |
|  |  |  |  |  |  |  |  |  |  | 2m2f | 4m2f | 2m4f | 4m4f |
| NVSLGNVLAVAYAAK | 16917 | 46.10 | 2 | 1488.8302 | 2 |  | 0 |  | yes | 335.03 | 94.64 | 225.91 | 133.67 |

  

| Tags | |
| --- | --- |

  

##

## Q99PT1

GDIR1\_MOUSE
Rho GDP-dissociation inhibitor 1 OS=Mus musculus GN=Arhgdia PE=1 SV=3  
1
peptide

  

| Sequence | Peptide Ion | Score | Hits | Mass | Charge | Tags | Conflicts | Modifications | In quantitation | Average Normalised Abundances | | | |
| --- | --- | --- | --- | --- | --- | --- | --- | --- | --- | --- | --- | --- | --- |
|  |  |  |  |  |  |  |  |  |  | 2m2f | 4m2f | 2m4f | 4m4f |
| AEEYEFLTPMEEAPK | 6604 | 45.59 | 3 | 1782.8029 | 2 |  | 0 |  | yes | 1570.44 | 1564.56 | 1366.23 | 1125.33 |

  

| Tags | |
| --- | --- |

  

##

## Q93092

TALDO\_MOUSE
Transaldolase OS=Mus musculus GN=Taldo1 PE=1 SV=2  
1 peptide

  

| Sequence | Peptide Ion | Score | Hits | Mass | Charge | Tags | Conflicts | Modifications | In quantitation | Average Normalised Abundances | | | |
| --- | --- | --- | --- | --- | --- | --- | --- | --- | --- | --- | --- | --- | --- |
|  |  |  |  |  |  |  |  |  |  | 2m2f | 4m2f | 2m4f | 4m4f |
| VSTEVDAR | 7330 | 45.19 | 3 | 875.4346 | 2 |  | 0 |  | yes | 632.74 | 301.24 | 370.46 | 253.95 |

  

| Tags | |
| --- | --- |

  

##

## Q9CY27

TECR\_MOUSE
Trans-2,3-enoyl-CoA reductase OS=Mus musculus GN=Tecr PE=1 SV=1  
1
peptide

  

| Sequence | Peptide Ion | Score | Hits | Mass | Charge | Tags | Conflicts | Modifications | In quantitation | Average Normalised Abundances | | | |
| --- | --- | --- | --- | --- | --- | --- | --- | --- | --- | --- | --- | --- | --- |
|  |  |  |  |  |  |  |  |  |  | 2m2f | 4m2f | 2m4f | 4m4f |
| LPVGTTATLYFR | 8038 | 45.07 | 1 | 1337.7336 | 2 |  | 0 |  | yes | 440.60 | 269.44 | 423.63 | 398.74 |

  

| Tags | |
| --- | --- |

  

##

## Q9CZ13

QCR1\_MOUSE
Cytochrome b-c1 complex subunit 1, mitochondrial OS=Mus musculus GN=Uqcrc1 PE=1
SV=1  
1 peptide

  

| Sequence | Peptide Ion | Score | Hits | Mass | Charge | Tags | Conflicts | Modifications | In quantitation | Average Normalised Abundances | | | |
| --- | --- | --- | --- | --- | --- | --- | --- | --- | --- | --- | --- | --- | --- |
|  |  |  |  |  |  |  |  |  |  | 2m2f | 4m2f | 2m4f | 4m4f |
| VVELLADIVQNSSLEDSQIEK | 9441 | 44.86 | 3 | 2328.2045 | 3 |  | 0 |  | yes | 1752.97 | 535.89 | 1350.70 | 335.47 |

  

| Tags | |
| --- | --- |

  

##

## Q9DCX2

ATP5H\_MOUSE
ATP synthase subunit d, mitochondrial OS=Mus musculus GN=Atp5h PE=1
SV=3  
1 peptide

  

| Sequence | Peptide Ion | Score | Hits | Mass | Charge | Tags | Conflicts | Modifications | In quantitation | Average Normalised Abundances | | | |
| --- | --- | --- | --- | --- | --- | --- | --- | --- | --- | --- | --- | --- | --- |
|  |  |  |  |  |  |  |  |  |  | 2m2f | 4m2f | 2m4f | 4m4f |
| SCAEFVSGSQLR | 10104 | 43.99 | 1 | 1339.6189 | 2 |  | 0 |  | yes | 390.94 | 202.10 | 367.30 | 253.95 |

  

| Tags | |
| --- | --- |

  

##

## Q9D1M7

FKB11\_MOUSE
Peptidyl-prolyl cis-trans isomerase FKBP11 OS=Mus musculus GN=Fkbp11 PE=2
SV=1  
1 peptide

  

| Sequence | Peptide Ion | Score | Hits | Mass | Charge | Tags | Conflicts | Modifications | In quantitation | Average Normalised Abundances | | | |
| --- | --- | --- | --- | --- | --- | --- | --- | --- | --- | --- | --- | --- | --- |
|  |  |  |  |  |  |  |  |  |  | 2m2f | 4m2f | 2m4f | 4m4f |
| DPLVIELGQK | 6730 | 43.94 | 3 | 1110.6275 | 2 |  | 0 |  | yes | 516.76 | 288.28 | 443.88 | 331.83 |

  

| Tags | |
| --- | --- |

  

##

## Q3UPH1

PRRC1\_MOUSE
Protein PRRC1 OS=Mus musculus GN=Prrc1 PE=2 SV=1  
1 peptide

  

| Sequence | Peptide Ion | Score | Hits | Mass | Charge | Tags | Conflicts | Modifications | In quantitation | Average Normalised Abundances | | | |
| --- | --- | --- | --- | --- | --- | --- | --- | --- | --- | --- | --- | --- | --- |
|  |  |  |  |  |  |  |  |  |  | 2m2f | 4m2f | 2m4f | 4m4f |
| GQDDAPAGGIWGFIK | 12698 | 43.77 | 3 | 1530.7513 | 2 |  | 0 |  | yes | 263.81 | 119.50 | 241.94 | 214.44 |

  

| Tags | |
| --- | --- |

  

##

## P19253

RL13A\_MOUSE
60S ribosomal protein L13a OS=Mus musculus GN=Rpl13a PE=1 SV=4  
1
peptide

  

| Sequence | Peptide Ion | Score | Hits | Mass | Charge | Tags | Conflicts | Modifications | In quantitation | Average Normalised Abundances | | | |
| --- | --- | --- | --- | --- | --- | --- | --- | --- | --- | --- | --- | --- | --- |
|  |  |  |  |  |  |  |  |  |  | 2m2f | 4m2f | 2m4f | 4m4f |
| YQAVTATLEEK | 2437 | 43.74 | 3 | 1251.6343 | 2 |  | 0 |  | yes | 2348.49 | 1805.42 | 1788.91 | 1766.46 |

  

| Tags | |
| --- | --- |

  

##

## P56391

CX6B1\_MOUSE
Cytochrome c oxidase subunit 6B1 OS=Mus musculus GN=Cox6b1 PE=1 SV=2  
1
peptide

  

| Sequence | Peptide Ion | Score | Hits | Mass | Charge | Tags | Conflicts | Modifications | In quantitation | Average Normalised Abundances | | | |
| --- | --- | --- | --- | --- | --- | --- | --- | --- | --- | --- | --- | --- | --- |
|  |  |  |  |  |  |  |  |  |  | 2m2f | 4m2f | 2m4f | 4m4f |
| SLCPVSWVSAWDDR | 10222 | 43.72 | 3 | 1676.7615 | 2 |  | 0 |  | yes | 1466.84 | 244.87 | 1152.02 | 622.45 |

  

| Tags | |
| --- | --- |

  

##

## P42932

TCPQ\_MOUSE
T-complex protein 1 subunit theta OS=Mus musculus GN=Cct8 PE=1 SV=3  
1
peptide

  

| Sequence | Peptide Ion | Score | Hits | Mass | Charge | Tags | Conflicts | Modifications | In quantitation | Average Normalised Abundances | | | |
| --- | --- | --- | --- | --- | --- | --- | --- | --- | --- | --- | --- | --- | --- |
|  |  |  |  |  |  |  |  |  |  | 2m2f | 4m2f | 2m4f | 4m4f |
| LATNAAVTVLR | 7371 | 43.62 | 3 | 1127.6662 | 2 |  | 1 |  | no | 538.11 | 342.50 | 309.91 | 461.18 |

  

| Tags | |
| --- | --- |

  

##

## Q9R0Q3

TMED2\_MOUSE
Transmembrane emp24 domain-containing protein 2 OS=Mus musculus GN=Tmed2 PE=1
SV=1  
1 peptide

  

| Sequence | Peptide Ion | Score | Hits | Mass | Charge | Tags | Conflicts | Modifications | In quantitation | Average Normalised Abundances | | | |
| --- | --- | --- | --- | --- | --- | --- | --- | --- | --- | --- | --- | --- | --- |
|  |  |  |  |  |  |  |  |  |  | 2m2f | 4m2f | 2m4f | 4m4f |
| LEEMINELAVAMTAVK | 23599 | 43.47 | 3 | 1760.9043 | 3 |  | 0 |  | yes | 227.78 | 27.77 | 133.73 | 59.83 |

  

| Tags | |
| --- | --- |

  

##

## Q9CQC9

SAR1B\_MOUSE
GTP-binding protein SAR1b OS=Mus musculus GN=Sar1b PE=1 SV=1  
1
peptide

  

| Sequence | Peptide Ion | Score | Hits | Mass | Charge | Tags | Conflicts | Modifications | In quantitation | Average Normalised Abundances | | | |
| --- | --- | --- | --- | --- | --- | --- | --- | --- | --- | --- | --- | --- | --- |
|  |  |  |  |  |  |  |  |  |  | 2m2f | 4m2f | 2m4f | 4m4f |
| LVFLGLDNAGK | 3921 | 43.42 | 3 | 1145.6432 | 2 |  | 0 |  | yes | 1245.84 | 1152.35 | 1675.82 | 1817.88 |

  

| Tags | |
| --- | --- |

  

##

## P26350

PTMA\_MOUSE
Prothymosin alpha OS=Mus musculus GN=Ptma PE=1 SV=2  
1
peptide

  

| Sequence | Peptide Ion | Score | Hits | Mass | Charge | Tags | Conflicts | Modifications | In quantitation | Average Normalised Abundances | | | |
| --- | --- | --- | --- | --- | --- | --- | --- | --- | --- | --- | --- | --- | --- |
|  |  |  |  |  |  |  |  |  |  | 2m2f | 4m2f | 2m4f | 4m4f |
| VAEDDEDDDVDTKK | 5124 | 43.16 | 3 | 1592.6680 | 3 |  | 0 |  | yes | 1589.14 | 1157.97 | 1310.99 | 1078.55 |

  

| Tags | |
| --- | --- |

  

##

## P06151

LDHA\_MOUSE
L-lactate dehydrogenase A chain OS=Mus musculus GN=Ldha PE=1 SV=3  
1
peptide

  

| Sequence | Peptide Ion | Score | Hits | Mass | Charge | Tags | Conflicts | Modifications | In quantitation | Average Normalised Abundances | | | |
| --- | --- | --- | --- | --- | --- | --- | --- | --- | --- | --- | --- | --- | --- |
|  |  |  |  |  |  |  |  |  |  | 2m2f | 4m2f | 2m4f | 4m4f |
| DQLIVNLLK | 5739 | 43.14 | 3 | 1054.6375 | 2 |  | 0 |  | yes | 641.57 | 669.54 | 715.04 | 503.47 |

  

| Tags | |
| --- | --- |

  

##

## O35350

CAN1\_MOUSE
Calpain-1 catalytic subunit OS=Mus musculus GN=Capn1 PE=2 SV=1  
1
peptide

  

| Sequence | Peptide Ion | Score | Hits | Mass | Charge | Tags | Conflicts | Modifications | In quantitation | Average Normalised Abundances | | | |
| --- | --- | --- | --- | --- | --- | --- | --- | --- | --- | --- | --- | --- | --- |
|  |  |  |  |  |  |  |  |  |  | 2m2f | 4m2f | 2m4f | 4m4f |
| APSDLYQIILK | 11766 | 43.03 | 2 | 1259.7111 | 2 |  | 0 |  | yes | 255.23 | 129.76 | 192.13 | 265.73 |

  

| Tags | |
| --- | --- |

  

##

## P10639

THIO\_MOUSE
Thioredoxin OS=Mus musculus GN=Txn PE=1 SV=3  
1 peptide

  

| Sequence | Peptide Ion | Score | Hits | Mass | Charge | Tags | Conflicts | Modifications | In quantitation | Average Normalised Abundances | | | |
| --- | --- | --- | --- | --- | --- | --- | --- | --- | --- | --- | --- | --- | --- |
|  |  |  |  |  |  |  |  |  |  | 2m2f | 4m2f | 2m4f | 4m4f |
| VGEFSGANK | 3320 | 42.89 | 3 | 907.4407 | 2 |  | 0 |  | yes | 2273.33 | 1828.15 | 2078.52 | 1673.44 |

  

| Tags | |
| --- | --- |

  

##

## Q8VDJ3

VIGLN\_MOUSE
Vigilin OS=Mus musculus GN=Hdlbp PE=1 SV=1  
1 peptide

  

| Sequence | Peptide Ion | Score | Hits | Mass | Charge | Tags | Conflicts | Modifications | In quantitation | Average Normalised Abundances | | | |
| --- | --- | --- | --- | --- | --- | --- | --- | --- | --- | --- | --- | --- | --- |
|  |  |  |  |  |  |  |  |  |  | 2m2f | 4m2f | 2m4f | 4m4f |
| DLANIAEVEVSIPAK | 12310 | 42.86 | 1 | 1567.8450 | 2 |  | 0 |  | yes | 623.87 | 255.70 | 624.92 | 250.44 |

  

| Tags | |
| --- | --- |

  

##

## P50431

GLYC\_MOUSE
Serine hydroxymethyltransferase, cytosolic OS=Mus musculus GN=Shmt1 PE=1
SV=2  
1 peptide

  

| Sequence | Peptide Ion | Score | Hits | Mass | Charge | Tags | Conflicts | Modifications | In quantitation | Average Normalised Abundances | | | |
| --- | --- | --- | --- | --- | --- | --- | --- | --- | --- | --- | --- | --- | --- |
|  |  |  |  |  |  |  |  |  |  | 2m2f | 4m2f | 2m4f | 4m4f |
| ISATSIFFESMPYK | 17919 | 42.84 | 3 | 1619.7944 | 2 |  | 0 |  | yes | 496.95 | 70.01 | 368.50 | 217.19 |

  

| Tags | |
| --- | --- |

  

##

## Q9JLR1

S61A2\_MOUSE
Protein transport protein Sec61 subunit alpha isoform 2 OS=Mus musculus
GN=Sec61a2 PE=2 SV=3  
1 peptide

  

| Sequence | Peptide Ion | Score | Hits | Mass | Charge | Tags | Conflicts | Modifications | In quantitation | Average Normalised Abundances | | | |
| --- | --- | --- | --- | --- | --- | --- | --- | --- | --- | --- | --- | --- | --- |
|  |  |  |  |  |  |  |  |  |  | 2m2f | 4m2f | 2m4f | 4m4f |
| IIEVGDTPK | 4319 | 42.69 | 3 | 970.5321 | 2 |  | 0 |  | yes | 1098.69 | 1236.69 | 955.78 | 1101.05 |

  

| Tags | |
| --- | --- |

  

##

## P61514

RL37A\_MOUSE
60S ribosomal protein L37a OS=Mus musculus GN=Rpl37a PE=2 SV=2  
1
peptide

  

| Sequence | Peptide Ion | Score | Hits | Mass | Charge | Tags | Conflicts | Modifications | In quantitation | Average Normalised Abundances | | | |
| --- | --- | --- | --- | --- | --- | --- | --- | --- | --- | --- | --- | --- | --- |
|  |  |  |  |  |  |  |  |  |  | 2m2f | 4m2f | 2m4f | 4m4f |
| TVAGGAWTYNTTSAVTVK | 6004 | 42.61 | 2 | 1825.9210 | 2 |  | 0 |  | yes | 1752.80 | 1155.32 | 1458.49 | 1045.46 |

  

| Tags | |
| --- | --- |

  

##

## P49962

SRP09\_MOUSE
Signal recognition particle 9 kDa protein OS=Mus musculus GN=Srp9 PE=1
SV=2  
1 peptide

  

| Sequence | Peptide Ion | Score | Hits | Mass | Charge | Tags | Conflicts | Modifications | In quantitation | Average Normalised Abundances | | | |
| --- | --- | --- | --- | --- | --- | --- | --- | --- | --- | --- | --- | --- | --- |
|  |  |  |  |  |  |  |  |  |  | 2m2f | 4m2f | 2m4f | 4m4f |
| VTDDLVCLVYR | 11101 | 41.45 | 1 | 1351.6805 | 2 |  | 0 |  | yes | 410.06 | 308.43 | 524.52 | 339.35 |

  

| Tags | |
| --- | --- |

  

##

## Q9R099

TBL2\_MOUSE
Transducin beta-like protein 2 OS=Mus musculus GN=Tbl2 PE=2 SV=1  
1
peptide

  

| Sequence | Peptide Ion | Score | Hits | Mass | Charge | Tags | Conflicts | Modifications | In quantitation | Average Normalised Abundances | | | |
| --- | --- | --- | --- | --- | --- | --- | --- | --- | --- | --- | --- | --- | --- |
|  |  |  |  |  |  |  |  |  |  | 2m2f | 4m2f | 2m4f | 4m4f |
| LQQQLTQAQEALK | 19229 | 40.92 | 1 | 1497.8138 | 2 |  | 0 |  | yes | 278.73 | 125.17 | 298.24 | 128.37 |

  

| Tags | |
| --- | --- |

  

##

## Q5SYD0

MYO1D\_MOUSE
Myosin-Id OS=Mus musculus GN=Myo1d PE=1 SV=1  
1 peptide

  

| Sequence | Peptide Ion | Score | Hits | Mass | Charge | Tags | Conflicts | Modifications | In quantitation | Average Normalised Abundances | | | |
| --- | --- | --- | --- | --- | --- | --- | --- | --- | --- | --- | --- | --- | --- |
|  |  |  |  |  |  |  |  |  |  | 2m2f | 4m2f | 2m4f | 4m4f |
| VVSVIAELLSTK | 14280 | 40.79 | 3 | 1257.7548 | 2 |  | 0 |  | yes | 453.04 | 83.34 | 297.64 | 169.18 |

  

| Tags | |
| --- | --- |

  

##

## Q3TDQ1

STT3B\_MOUSE
Dolichyl-diphosphooligosaccharide--protein glycosyltransferase subunit STT3B
OS=Mus musculus GN=Stt3b PE=1 SV=2  
1 peptide

  

| Sequence | Peptide Ion | Score | Hits | Mass | Charge | Tags | Conflicts | Modifications | In quantitation | Average Normalised Abundances | | | |
| --- | --- | --- | --- | --- | --- | --- | --- | --- | --- | --- | --- | --- | --- |
|  |  |  |  |  |  |  |  |  |  | 2m2f | 4m2f | 2m4f | 4m4f |
| FYSLWDTGYAK | 6761 | 40.52 | 3 | 1349.6289 | 2 |  | 0 |  | yes | 932.77 | 909.28 | 1198.33 | 1072.77 |

  

| Tags | |
| --- | --- |

  

##

## P62862

RS30\_MOUSE
40S ribosomal protein S30 OS=Mus musculus GN=Fau PE=3 SV=1  
1
peptide

  

| Sequence | Peptide Ion | Score | Hits | Mass | Charge | Tags | Conflicts | Modifications | In quantitation | Average Normalised Abundances | | | |
| --- | --- | --- | --- | --- | --- | --- | --- | --- | --- | --- | --- | --- | --- |
|  |  |  |  |  |  |  |  |  |  | 2m2f | 4m2f | 2m4f | 4m4f |
| FVNVVPTFGK | 7351 | 40.39 | 3 | 1106.6088 | 2 |  | 0 |  | yes | 666.15 | 641.80 | 491.03 | 714.81 |

  

| Tags | |
| --- | --- |

  

##

## Q9CPY7

AMPL\_MOUSE
Cytosol aminopeptidase OS=Mus musculus GN=Lap3 PE=1 SV=3  
1
peptide

  

| Sequence | Peptide Ion | Score | Hits | Mass | Charge | Tags | Conflicts | Modifications | In quantitation | Average Normalised Abundances | | | |
| --- | --- | --- | --- | --- | --- | --- | --- | --- | --- | --- | --- | --- | --- |
|  |  |  |  |  |  |  |  |  |  | 2m2f | 4m2f | 2m4f | 4m4f |
| GITFDSGGISIK | 12378 | 39.82 | 3 | 1193.6296 | 2 |  | 0 |  | yes | 250.20 | 193.78 | 214.20 | 191.10 |

  

| Tags | |
| --- | --- |

  

##

## P62827

RAN\_MOUSE
GTP-binding nuclear protein Ran OS=Mus musculus GN=Ran PE=1 SV=3  
1
peptide

  

| Sequence | Peptide Ion | Score | Hits | Mass | Charge | Tags | Conflicts | Modifications | In quantitation | Average Normalised Abundances | | | |
| --- | --- | --- | --- | --- | --- | --- | --- | --- | --- | --- | --- | --- | --- |
|  |  |  |  |  |  |  |  |  |  | 2m2f | 4m2f | 2m4f | 4m4f |
| NLQYYDISAK | 7235 | 39.57 | 3 | 1213.5989 | 2 |  | 0 |  | yes | 863.39 | 946.65 | 664.35 | 886.06 |

  

| Tags | |
| --- | --- |

  

##

## P61961

UFM1\_MOUSE
Ubiquitin-fold modifier 1 OS=Mus musculus GN=Ufm1 PE=1 SV=1  
1
peptide

  

| Sequence | Peptide Ion | Score | Hits | Mass | Charge | Tags | Conflicts | Modifications | In quantitation | Average Normalised Abundances | | | |
| --- | --- | --- | --- | --- | --- | --- | --- | --- | --- | --- | --- | --- | --- |
|  |  |  |  |  |  |  |  |  |  | 2m2f | 4m2f | 2m4f | 4m4f |
| VLSVPESTPFTAVLK | 4773 | 39.37 | 3 | 1586.8918 | 2 |  | 0 |  | yes | 3209.92 | 2248.50 | 2787.29 | 2498.22 |

  

| Tags | |
| --- | --- |

  

##

## Q91VR2

ATPG\_MOUSE
ATP synthase subunit gamma, mitochondrial OS=Mus musculus GN=Atp5c1 PE=1
SV=1  
1 peptide

  

| Sequence | Peptide Ion | Score | Hits | Mass | Charge | Tags | Conflicts | Modifications | In quantitation | Average Normalised Abundances | | | |
| --- | --- | --- | --- | --- | --- | --- | --- | --- | --- | --- | --- | --- | --- |
|  |  |  |  |  |  |  |  |  |  | 2m2f | 4m2f | 2m4f | 4m4f |
| NEVAALTAAGK | 8969 | 39.22 | 1 | 1043.5628 | 2 |  | 0 |  | yes | 308.51 | 241.60 | 330.97 | 303.76 |

  

| Tags | |
| --- | --- |

  

##

## P00405

COX2\_MOUSE
Cytochrome c oxidase subunit 2 OS=Mus musculus GN=Mtco2 PE=1 SV=1  
1
peptide

  

| Sequence | Peptide Ion | Score | Hits | Mass | Charge | Tags | Conflicts | Modifications | In quantitation | Average Normalised Abundances | | | |
| --- | --- | --- | --- | --- | --- | --- | --- | --- | --- | --- | --- | --- | --- |
|  |  |  |  |  |  |  |  |  |  | 2m2f | 4m2f | 2m4f | 4m4f |
| VVLPMELPIR | 7735 | 39.16 | 3 | 1165.6887 | 2 |  | 0 |  | yes | 614.15 | 325.69 | 519.96 | 396.99 |

  

| Tags | |
| --- | --- |

  

##

## P47758

SRPRB\_MOUSE
Signal recognition particle receptor subunit beta OS=Mus musculus GN=Srprb PE=1
SV=1  
1 peptide

  

| Sequence | Peptide Ion | Score | Hits | Mass | Charge | Tags | Conflicts | Modifications | In quantitation | Average Normalised Abundances | | | |
| --- | --- | --- | --- | --- | --- | --- | --- | --- | --- | --- | --- | --- | --- |
|  |  |  |  |  |  |  |  |  |  | 2m2f | 4m2f | 2m4f | 4m4f |
| EFEFSQLPLK | 10758 | 38.05 | 1 | 1236.6373 | 2 |  | 0 |  | yes | 296.21 | 138.74 | 239.42 | 261.89 |

  

| Tags | |
| --- | --- |

  

##

## P47738

ALDH2\_MOUSE
Aldehyde dehydrogenase, mitochondrial OS=Mus musculus GN=Aldh2 PE=1
SV=1  
1 peptide

  

| Sequence | Peptide Ion | Score | Hits | Mass | Charge | Tags | Conflicts | Modifications | In quantitation | Average Normalised Abundances | | | |
| --- | --- | --- | --- | --- | --- | --- | --- | --- | --- | --- | --- | --- | --- |
|  |  |  |  |  |  |  |  |  |  | 2m2f | 4m2f | 2m4f | 4m4f |
| GYFIQPTVFGDVK | 9763 | 38.05 | 3 | 1469.7560 | 2 |  | 0 |  | yes | 514.00 | 305.09 | 506.82 | 344.09 |

  

| Tags | |
| --- | --- |

  

##

## Q62393

TPD52\_MOUSE
Tumor protein D52 OS=Mus musculus GN=Tpd52 PE=1 SV=2  
1
peptide

  

| Sequence | Peptide Ion | Score | Hits | Mass | Charge | Tags | Conflicts | Modifications | In quantitation | Average Normalised Abundances | | | |
| --- | --- | --- | --- | --- | --- | --- | --- | --- | --- | --- | --- | --- | --- |
|  |  |  |  |  |  |  |  |  |  | 2m2f | 4m2f | 2m4f | 4m4f |
| VEEEIQTLSQVLAAK | 17623 | --- | --- | 1656.8923 | 3 |  | 0 |  | yes | 124.97 | 89.99 | 114.21 | 128.85 |
| VEEEIQTLSQVLAAK | 11814 | 37.43 | 1 | 1656.8934 | 2 |  | 0 |  | yes | 470.33 | 235.36 | 504.96 | 348.12 |

  

| Tags | |
| --- | --- |

  

##

## Q60597

ODO1\_MOUSE
2-oxoglutarate dehydrogenase, mitochondrial OS=Mus musculus GN=Ogdh PE=1
SV=3  
1 peptide

  

| Sequence | Peptide Ion | Score | Hits | Mass | Charge | Tags | Conflicts | Modifications | In quantitation | Average Normalised Abundances | | | |
| --- | --- | --- | --- | --- | --- | --- | --- | --- | --- | --- | --- | --- | --- |
|  |  |  |  |  |  |  |  |  |  | 2m2f | 4m2f | 2m4f | 4m4f |
| LEAADEGSGDMK | 38549 | 37.39 | 1 | 1237.5123 | 2 |  | 0 | [11] Oxidation (M) | yes | 114.97 | 22.06 | 29.27 | 51.99 |

  

| Tags | |
| --- | --- |

  

##

## Q91W90

TXND5\_MOUSE
Thioredoxin domain-containing protein 5 OS=Mus musculus GN=Txndc5 PE=1
SV=2  
1 peptide

  

| Sequence | Peptide Ion | Score | Hits | Mass | Charge | Tags | Conflicts | Modifications | In quantitation | Average Normalised Abundances | | | |
| --- | --- | --- | --- | --- | --- | --- | --- | --- | --- | --- | --- | --- | --- |
|  |  |  |  |  |  |  |  |  |  | 2m2f | 4m2f | 2m4f | 4m4f |
| GYPTLLLFR | 11364 | 37.31 | 2 | 1078.6166 | 2 |  | 0 |  | yes | 479.42 | 184.28 | 388.65 | 339.02 |

  

| Tags | |
| --- | --- |

  

##

## Q63805

A1AG3\_MOUSE
Alpha-1-acid glycoprotein 3 OS=Mus musculus GN=Orm3 PE=2 SV=1  
1
peptide

  

| Sequence | Peptide Ion | Score | Hits | Mass | Charge | Tags | Conflicts | Modifications | In quantitation | Average Normalised Abundances | | | |
| --- | --- | --- | --- | --- | --- | --- | --- | --- | --- | --- | --- | --- | --- |
|  |  |  |  |  |  |  |  |  |  | 2m2f | 4m2f | 2m4f | 4m4f |
| AVTHVGMDESEIIFVDWK | 19001 | 37.16 | 1 | 2075.0026 | 3 |  | 0 |  | yes | 209.10 | 146.89 | 338.03 | 71.21 |

  

| Tags | |
| --- | --- |

  

##

## Q9JII6

AK1A1\_MOUSE
Alcohol dehydrogenase [NADP+] OS=Mus musculus GN=Akr1a1 PE=1 SV=3  
1
peptide

  

| Sequence | Peptide Ion | Score | Hits | Mass | Charge | Tags | Conflicts | Modifications | In quantitation | Average Normalised Abundances | | | |
| --- | --- | --- | --- | --- | --- | --- | --- | --- | --- | --- | --- | --- | --- |
|  |  |  |  |  |  |  |  |  |  | 2m2f | 4m2f | 2m4f | 4m4f |
| MPLIGLGTWK | 9743 | 36.92 | 3 | 1114.6198 | 2 |  | 0 |  | yes | 333.07 | 205.07 | 245.30 | 284.30 |

  

| Tags | |
| --- | --- |

  

##

## Q9Z0J0

NPC2\_MOUSE
Epididymal secretory protein E1 OS=Mus musculus GN=Npc2 PE=2 SV=1  
1
peptide

  

| Sequence | Peptide Ion | Score | Hits | Mass | Charge | Tags | Conflicts | Modifications | In quantitation | Average Normalised Abundances | | | |
| --- | --- | --- | --- | --- | --- | --- | --- | --- | --- | --- | --- | --- | --- |
|  |  |  |  |  |  |  |  |  |  | 2m2f | 4m2f | 2m4f | 4m4f |
| VPFPIPEPDGCK | 7963 | 36.82 | 2 | 1354.6585 | 2 |  | 0 |  | yes | 927.16 | 595.02 | 671.64 | 540.51 |

  

| Tags | |
| --- | --- |

  

##

## Q6ZWV3

RL10\_MOUSE
60S ribosomal protein L10 OS=Mus musculus GN=Rpl10 PE=2 SV=3  
1
peptide

  

| Sequence | Peptide Ion | Score | Hits | Mass | Charge | Tags | Conflicts | Modifications | In quantitation | Average Normalised Abundances | | | |
| --- | --- | --- | --- | --- | --- | --- | --- | --- | --- | --- | --- | --- | --- |
|  |  |  |  |  |  |  |  |  |  | 2m2f | 4m2f | 2m4f | 4m4f |
| MLSCAGADR | 8322 | 36.80 | 3 | 979.4210 | 2 |  | 0 |  | yes | 548.59 | 341.12 | 430.03 | 363.40 |

  

| Tags | |
| --- | --- |

  

##

## Q9CPT4

CS010\_MOUSE
UPF0556 protein C19orf10 homolog OS=Mus musculus GN=D17Wsu104e PE=2
SV=1  
1 peptide

  

| Sequence | Peptide Ion | Score | Hits | Mass | Charge | Tags | Conflicts | Modifications | In quantitation | Average Normalised Abundances | | | |
| --- | --- | --- | --- | --- | --- | --- | --- | --- | --- | --- | --- | --- | --- |
|  |  |  |  |  |  |  |  |  |  | 2m2f | 4m2f | 2m4f | 4m4f |
| SYLYFTQFK | 9046 | 36.53 | 3 | 1195.5911 | 2 |  | 0 |  | yes | 461.10 | 404.16 | 444.52 | 389.24 |

  

| Tags | |
| --- | --- |

  

##

## Q9CQS8

SC61B\_MOUSE
Protein transport protein Sec61 subunit beta OS=Mus musculus GN=Sec61b PE=1
SV=3  
1 peptide

  

| Sequence | Peptide Ion | Score | Hits | Mass | Charge | Tags | Conflicts | Modifications | In quantitation | Average Normalised Abundances | | | |
| --- | --- | --- | --- | --- | --- | --- | --- | --- | --- | --- | --- | --- | --- |
|  |  |  |  |  |  |  |  |  |  | 2m2f | 4m2f | 2m4f | 4m4f |
| FYTEDSPGLK | 2928 | 36.22 | 3 | 1155.5448 | 2 |  | 0 |  | yes | 2462.58 | 2359.34 | 2269.41 | 2284.75 |

  

| Tags | |
| --- | --- |

  

##

## Q64442

DHSO\_MOUSE
Sorbitol dehydrogenase OS=Mus musculus GN=Sord PE=1 SV=3  
1
peptide

  

| Sequence | Peptide Ion | Score | Hits | Mass | Charge | Tags | Conflicts | Modifications | In quantitation | Average Normalised Abundances | | | |
| --- | --- | --- | --- | --- | --- | --- | --- | --- | --- | --- | --- | --- | --- |
|  |  |  |  |  |  |  |  |  |  | 2m2f | 4m2f | 2m4f | 4m4f |
| AVEAFETAK | 13112 | 35.67 | 3 | 964.4921 | 2 |  | 0 |  | yes | 362.66 | 223.02 | 303.57 | 221.55 |

  

| Tags | |
| --- | --- |

  

##

## Q8BMF4

ODP2\_MOUSE
Dihydrolipoyllysine-residue acetyltransferase component of pyruvate
dehydrogenase complex, mitochondrial OS=Mus musculus GN=Dlat PE=1
SV=2  
1 peptide

  

| Sequence | Peptide Ion | Score | Hits | Mass | Charge | Tags | Conflicts | Modifications | In quantitation | Average Normalised Abundances | | | |
| --- | --- | --- | --- | --- | --- | --- | --- | --- | --- | --- | --- | --- | --- |
|  |  |  |  |  |  |  |  |  |  | 2m2f | 4m2f | 2m4f | 4m4f |
| GLETIASDVVSLASK | 17342 | 35.52 | 1 | 1488.8030 | 2 |  | 0 |  | yes | 399.86 | 43.42 | 287.02 | 131.01 |

  

| Tags | |
| --- | --- |

  

##

## P41105

RL28\_MOUSE
60S ribosomal protein L28 OS=Mus musculus GN=Rpl28 PE=1 SV=2  
1
peptide

  

| Sequence | Peptide Ion | Score | Hits | Mass | Charge | Tags | Conflicts | Modifications | In quantitation | Average Normalised Abundances | | | |
| --- | --- | --- | --- | --- | --- | --- | --- | --- | --- | --- | --- | --- | --- |
|  |  |  |  |  |  |  |  |  |  | 2m2f | 4m2f | 2m4f | 4m4f |
| TVGVEPAADGK | 4029 | 35.37 | 3 | 1042.5285 | 2 |  | 0 |  | yes | 1479.09 | 1241.80 | 1425.99 | 1584.84 |

  

| Tags | |
| --- | --- |

  

##

## P60335

PCBP1\_MOUSE
Poly(rC)-binding protein 1 OS=Mus musculus GN=Pcbp1 PE=1 SV=1  
1
peptide

  

| Sequence | Peptide Ion | Score | Hits | Mass | Charge | Tags | Conflicts | Modifications | In quantitation | Average Normalised Abundances | | | |
| --- | --- | --- | --- | --- | --- | --- | --- | --- | --- | --- | --- | --- | --- |
|  |  |  |  |  |  |  |  |  |  | 2m2f | 4m2f | 2m4f | 4m4f |
| LVVPATQCGSLIGK | 6075 | 35.15 | 3 | 1441.7956 | 2 |  | 0 |  | yes | 878.50 | 544.97 | 863.97 | 676.61 |

  

| Tags | |
| --- | --- |

  

##

## P24527

LKHA4\_MOUSE
Leukotriene A-4 hydrolase OS=Mus musculus GN=Lta4h PE=1 SV=3  
1
peptide

  

| Sequence | Peptide Ion | Score | Hits | Mass | Charge | Tags | Conflicts | Modifications | In quantitation | Average Normalised Abundances | | | |
| --- | --- | --- | --- | --- | --- | --- | --- | --- | --- | --- | --- | --- | --- |
|  |  |  |  |  |  |  |  |  |  | 2m2f | 4m2f | 2m4f | 4m4f |
| WEEAIPLALK | 15072 | 35.05 | 3 | 1168.6481 | 2 |  | 0 |  | yes | 311.72 | 197.30 | 248.25 | 239.45 |

  

| Tags | |
| --- | --- |

  

##

## O88487

DC1I2\_MOUSE
Cytoplasmic dynein 1 intermediate chain 2 OS=Mus musculus GN=Dync1i2 PE=2
SV=1  
1 peptide

  

| Sequence | Peptide Ion | Score | Hits | Mass | Charge | Tags | Conflicts | Modifications | In quantitation | Average Normalised Abundances | | | |
| --- | --- | --- | --- | --- | --- | --- | --- | --- | --- | --- | --- | --- | --- |
|  |  |  |  |  |  |  |  |  |  | 2m2f | 4m2f | 2m4f | 4m4f |
| ADAEEEAATR | 4250 | 34.98 | 2 | 1061.4660 | 2 |  | 0 |  | yes | 1551.34 | 2036.63 | 2022.79 | 1963.08 |

  

| Tags | |
| --- | --- |

  

##

## Q9D051

ODPB\_MOUSE
Pyruvate dehydrogenase E1 component subunit beta, mitochondrial OS=Mus musculus
GN=Pdhb PE=1 SV=1  
1 peptide

  

| Sequence | Peptide Ion | Score | Hits | Mass | Charge | Tags | Conflicts | Modifications | In quantitation | Average Normalised Abundances | | | |
| --- | --- | --- | --- | --- | --- | --- | --- | --- | --- | --- | --- | --- | --- |
|  |  |  |  |  |  |  |  |  |  | 2m2f | 4m2f | 2m4f | 4m4f |
| VFLLGEEVAQYDGAYK | 18001 | 34.75 | 1 | 1800.8961 | 2 |  | 0 |  | yes | 437.12 | 213.19 | 294.86 | 269.39 |

  

| Tags | |
| --- | --- |

  

##

## Q9EPC1

PARVA\_MOUSE
Alpha-parvin OS=Mus musculus GN=Parva PE=1 SV=1  
1 peptide

  

| Sequence | Peptide Ion | Score | Hits | Mass | Charge | Tags | Conflicts | Modifications | In quantitation | Average Normalised Abundances | | | |
| --- | --- | --- | --- | --- | --- | --- | --- | --- | --- | --- | --- | --- | --- |
|  |  |  |  |  |  |  |  |  |  | 2m2f | 4m2f | 2m4f | 4m4f |
| VLIDWINDVLVGER | 22755 | 34.01 | 3 | 1639.8927 | 2 |  | 0 |  | yes | 349.65 | 48.91 | 340.42 | 92.92 |

  

| Tags | |
| --- | --- |

  

##

## Q9R1P0

PSA4\_MOUSE
Proteasome subunit alpha type-4 OS=Mus musculus GN=Psma4 PE=1 SV=1  
1
peptide

  

| Sequence | Peptide Ion | Score | Hits | Mass | Charge | Tags | Conflicts | Modifications | In quantitation | Average Normalised Abundances | | | |
| --- | --- | --- | --- | --- | --- | --- | --- | --- | --- | --- | --- | --- | --- |
|  |  |  |  |  |  |  |  |  |  | 2m2f | 4m2f | 2m4f | 4m4f |
| LLDEVFFSEK | 5437 | 33.71 | 3 | 1225.6287 | 2 |  | 0 |  | yes | 691.73 | 355.12 | 720.70 | 464.10 |

  

| Tags | |
| --- | --- |

  

##

## Q9D662

SC23B\_MOUSE
Protein transport protein Sec23B OS=Mus musculus GN=Sec23b PE=2 SV=1  
1
peptide

  

| Sequence | Peptide Ion | Score | Hits | Mass | Charge | Tags | Conflicts | Modifications | In quantitation | Average Normalised Abundances | | | |
| --- | --- | --- | --- | --- | --- | --- | --- | --- | --- | --- | --- | --- | --- |
|  |  |  |  |  |  |  |  |  |  | 2m2f | 4m2f | 2m4f | 4m4f |
| MVVPLACLLTPLK | 11225 | 33.51 | 3 | 1453.8399 | 2 |  | 0 |  | yes | 1251.38 | 188.30 | 1096.09 | 657.50 |

  

| Tags | |
| --- | --- |

  

##

## Q91YL7

PG2IP\_MOUSE
PGAP2-interacting protein OS=Mus musculus GN=Cwh43 PE=1 SV=1  
1
peptide

  

| Sequence | Peptide Ion | Score | Hits | Mass | Charge | Tags | Conflicts | Modifications | In quantitation | Average Normalised Abundances | | | |
| --- | --- | --- | --- | --- | --- | --- | --- | --- | --- | --- | --- | --- | --- |
|  |  |  |  |  |  |  |  |  |  | 2m2f | 4m2f | 2m4f | 4m4f |
| GAPATVVSAAIWPFR | 28860 | 33.43 | 1 | 1541.8347 | 2 |  | 0 |  | yes | 418.37 | 56.82 | 309.91 | 172.51 |

  

| Tags | |
| --- | --- |

  

##

## P23591

FCL\_MOUSE
GDP-L-fucose synthase OS=Mus musculus GN=Tsta3 PE=2 SV=3  
1
peptide

  

| Sequence | Peptide Ion | Score | Hits | Mass | Charge | Tags | Conflicts | Modifications | In quantitation | Average Normalised Abundances | | | |
| --- | --- | --- | --- | --- | --- | --- | --- | --- | --- | --- | --- | --- | --- |
|  |  |  |  |  |  |  |  |  |  | 2m2f | 4m2f | 2m4f | 4m4f |
| ILVTGGSGLVGR | 7371 | 33.42 | 3 | 1127.6662 | 2 |  | 1 |  | no | 538.11 | 342.50 | 309.91 | 461.18 |

  

| Tags | |
| --- | --- |

  

##

## Q3UPL0

SC31A\_MOUSE
Protein transport protein Sec31A OS=Mus musculus GN=Sec31a PE=1 SV=2  
1
peptide

  

| Sequence | Peptide Ion | Score | Hits | Mass | Charge | Tags | Conflicts | Modifications | In quantitation | Average Normalised Abundances | | | |
| --- | --- | --- | --- | --- | --- | --- | --- | --- | --- | --- | --- | --- | --- |
|  |  |  |  |  |  |  |  |  |  | 2m2f | 4m2f | 2m4f | 4m4f |
| AQDGSSPLSLQDLIEK | 10882 | 32.23 | 2 | 1699.8635 | 2 |  | 0 |  | yes | 658.60 | 423.33 | 668.85 | 482.61 |

  

| Tags | |
| --- | --- |

  

##

## P63276

RS17\_MOUSE
40S ribosomal protein S17 OS=Mus musculus GN=Rps17 PE=1 SV=2  
1
peptide

  

| Sequence | Peptide Ion | Score | Hits | Mass | Charge | Tags | Conflicts | Modifications | In quantitation | Average Normalised Abundances | | | |
| --- | --- | --- | --- | --- | --- | --- | --- | --- | --- | --- | --- | --- | --- |
|  |  |  |  |  |  |  |  |  |  | 2m2f | 4m2f | 2m4f | 4m4f |
| LLDFGSLSNLQVTQPTVGMNFK | 13564 | 32.10 | 1 | 2408.2399 | 3 |  | 0 |  | yes | 1157.71 | 87.65 | 790.21 | 223.07 |

  

| Tags | |
| --- | --- |

  

##

## P01868

IGHG1\_MOUSE
Ig gamma-1 chain C region secreted form OS=Mus musculus GN=Ighg1 PE=1
SV=1  
1 peptide

  

| Sequence | Peptide Ion | Score | Hits | Mass | Charge | Tags | Conflicts | Modifications | In quantitation | Average Normalised Abundances | | | |
| --- | --- | --- | --- | --- | --- | --- | --- | --- | --- | --- | --- | --- | --- |
|  |  |  |  |  |  |  |  |  |  | 2m2f | 4m2f | 2m4f | 4m4f |
| VNSAAFPAPIEK | 11637 | 31.73 | 2 | 1242.6637 | 2 |  | 0 |  | yes | 444.07 | 473.72 | 331.39 | 351.43 |

  

| Tags | |
| --- | --- |

  

##

## P15501

SPBP\_MOUSE
Prostatic spermine-binding protein OS=Mus musculus GN=Sbp PE=2 SV=1  
1
peptide

  

| Sequence | Peptide Ion | Score | Hits | Mass | Charge | Tags | Conflicts | Modifications | In quantitation | Average Normalised Abundances | | | |
| --- | --- | --- | --- | --- | --- | --- | --- | --- | --- | --- | --- | --- | --- |
|  |  |  |  |  |  |  |  |  |  | 2m2f | 4m2f | 2m4f | 4m4f |
| VATFGVR | 16349 | 31.73 | 1 | 748.4225 | 2 |  | 0 |  | yes | 12.87 | 0.54 | 64.32 | 6.26 |

  

| Tags | |
| --- | --- |

  

##

## P80314

TCPB\_MOUSE
T-complex protein 1 subunit beta OS=Mus musculus GN=Cct2 PE=1 SV=4  
1
peptide

  

| Sequence | Peptide Ion | Score | Hits | Mass | Charge | Tags | Conflicts | Modifications | In quantitation | Average Normalised Abundances | | | |
| --- | --- | --- | --- | --- | --- | --- | --- | --- | --- | --- | --- | --- | --- |
|  |  |  |  |  |  |  |  |  |  | 2m2f | 4m2f | 2m4f | 4m4f |
| LSSFIGAIAIGDLVK | 33787 | 31.39 | 1 | 1502.8717 | 2 |  | 0 |  | yes | 145.26 | 33.53 | 138.81 | 41.84 |

  

| Tags | |
| --- | --- |

  

##

## Q02053

UBA1\_MOUSE
Ubiquitin-like modifier-activating enzyme 1 OS=Mus musculus GN=Uba1 PE=1
SV=1  
1 peptide

  

| Sequence | Peptide Ion | Score | Hits | Mass | Charge | Tags | Conflicts | Modifications | In quantitation | Average Normalised Abundances | | | |
| --- | --- | --- | --- | --- | --- | --- | --- | --- | --- | --- | --- | --- | --- |
|  |  |  |  |  |  |  |  |  |  | 2m2f | 4m2f | 2m4f | 4m4f |
| AENYDISPADR | 14147 | 30.78 | 3 | 1249.5571 | 2 |  | 0 |  | yes | 491.10 | 117.96 | 371.78 | 242.05 |

  

| Tags | |
| --- | --- |

  

##

## Q9DC29

ABCB6\_MOUSE
ATP-binding cassette sub-family B member 6, mitochondrial OS=Mus musculus
GN=Abcb6 PE=1 SV=1  
1 peptide

  

| Sequence | Peptide Ion | Score | Hits | Mass | Charge | Tags | Conflicts | Modifications | In quantitation | Average Normalised Abundances | | | |
| --- | --- | --- | --- | --- | --- | --- | --- | --- | --- | --- | --- | --- | --- |
|  |  |  |  |  |  |  |  |  |  | 2m2f | 4m2f | 2m4f | 4m4f |
| AIQASLAK | 5144 | 30.26 | 3 | 800.4754 | 2 |  | 1 |  | no | 623.33 | 586.53 | 626.01 | 593.69 |

  

| Tags | |
| --- | --- |

  

##

## Q8BGY2

IF5A2\_MOUSE
Eukaryotic translation initiation factor 5A-2 OS=Mus musculus GN=Eif5a2 PE=2
SV=3  
1 peptide

  

| Sequence | Peptide Ion | Score | Hits | Mass | Charge | Tags | Conflicts | Modifications | In quantitation | Average Normalised Abundances | | | |
| --- | --- | --- | --- | --- | --- | --- | --- | --- | --- | --- | --- | --- | --- |
| 2m2f | 4m2f | 2m4f | 4m4f |
[truncated: 101,508 more chars]
